# Metabolomic Profiling of *Leptadenia reticulata*: Unveiling Therapeutic Potential for Inflammatory Diseases through Network Pharmacology and Docking Studies

**DOI:** 10.3390/ph17040423

**Published:** 2024-03-26

**Authors:** Yashaswini Mallepura Adinarayanaswamy, Deepthi Padmanabhan, Purushothaman Natarajan, Senthilkumar Palanisamy

**Affiliations:** 1 Department of Genetic Engineering, School of Bioengineering, SRM Institute of Science and Technology, Kattankulathur 603203, Tamil Nadu, India; yashaswinima2000@gmail.com (Y.M.A.); deepthipadmanabhan2010@gmail.com (D.P.); 2 Department of Biology, West Virginia State University, Institute, WV 25112-1000, USA; pnatarajan@wvstateu.edu

**Keywords:** drug discovery, medicinal plants, network pharmacology, phytocompounds, small molecules

## Abstract

Medicinal plants have been utilized since ancient times for their therapeutic properties, offering potential solutions for various ailments, including epidemics. Among these, *Leptadenia reticulata*, a member of the *Asclepiadaceae* family, has been traditionally employed to address numerous conditions such as diarrhea, cancer, and fever. In this study, employing HR-LCMS/MS(Q-TOF) analysis, we identified 113 compounds from the methanolic extract of *L. reticulata*. Utilizing Lipinski’s rule of five, we evaluated the drug-likeness of these compounds using SwissADME and ProTox II. SwissTarget Prediction facilitated the identification of potential inflammatory targets, and these targets were discerned through the Genecard, TTD, and CTD databases. A network pharmacology analysis unveiled hub proteins including CCR2, ICAM1, KIT, MPO, NOS2, and STAT3. Molecular docking studies identified various constituents of *L. reticulata*, exhibiting high binding affinity scores. Further investigations involving in vivo testing and genomic analyses of metabolite-encoding genes will be pivotal in developing efficacious natural-source drugs. Additionally, the potential of molecular dynamics simulations warrants exploration, offering insights into the dynamic behavior of protein–compound interactions and guiding the design of novel therapeutics.

## 1. Introduction

Inflammation is a painful redness or swelling of a body part brought on by an injury, disease, or infection. Inflammation may not always have a beneficial impact on the body. Autoimmune diseases arise whenever the immune system’s response inappropriately attacks the human body’s native cells, leading to harmful inflammation [1]. The production of cytokines, cell trafficking, mediator synthesis, fibrolysis, coagulation, extravasation changes in hemodynamic properties, and ultimately microvascular death-causing permeability are all part of a cascade of events which leads to inflammation [2]. Normally, the process results in healing and recovery from infection. Nevertheless, if targeted destruction and assistance with repair are not properly designed, inflammation may result in lasting damage to tissue by affecting collagen, leukocytes, or lymphocytes [3]. Inflammation is categorized as acute when marked by swift and intense onset due to factors like toxins or trauma, with symptoms lasting a short time, whereas chronic inflammation, lasting for months to years, depends on the body’s healing capacity and the nature of the initial injury [4]. The WHO says chronic illnesses are the largest health concern. Researchers expect long-term inflammation-related disorders to grow in the US during the next 30 years. In 2000, 125 million people had chronic diseases, and 61 million (21%) had several disorders [5]. Chronic inflammatory diseases such as stroke, lung, heart, cancer, obesity, and diabetes affect three in five people worldwide [6]. The activity of inflammatory-related cytokines, chemokines, adhesion proteins, and pro-inflammatory enzymes has been associated with chronic inflammation [7]. Nonsteroidal anti-inflammatory medicines (NSAIDs) are widely used pharmaceuticals for treating inflammation and associated illnesses, with a global consumption estimated to exceed 30 million per day [8,9,10]. Regrettably, the therapeutic use of NSAIDs is restricted due to the severe adverse effects they may cause, including gastrointestinal (GI) ulcers, perforation, obstruction, and bleeding, despite their strong anti-inflammatory efficacy [10]. NSAIDs may also elevate the likelihood of experiencing falls, heighten the occurrence of geriatric mental episodes, and amplify the danger of stroke [11].

Over 80,000 plants have medicinal properties, most of which have been utilized for centuries [12]. Traditional medicinal plants are receiving more attention from medical research and healthcare. India possesses a total of 45,000 medicinal plants in the Andaman and Nicobar Islands, Western Ghats, and Eastern Himalayas [13]. There are approximately 3000 therapeutic herbs that have received regulatory approval, although traditional practitioners utilize roughly 6000 herbs [14]. Approximately 75–80% of the global population residing in underdeveloped nations depend on natural products for their fundamental healthcare needs, owing to their greater cultural acceptance, compatibility with the human body, and lack of adverse side effects. Traditional medicinal plants produce compounds with anti-inflammatory, antioxidant, and antimicrobial effects [15]. Medicinal plants include a significant abundance of secondary metabolites, which play a crucial role in the identification of novel drugs. Medicinal plants yield a diverse array of secondary metabolites, including flavonoids, terpenoids, tannins, steroids, quinones, coumarins, and alkaloids. These compounds exhibit a broad spectrum of pharmacological properties [16]. *Leptadenia reticulata* is one of the traditional medicinal plants; it belongs to the family *Apocynaceae* [17]. It is commonly known as Jivanti (Sanskrit) and Palaikkodi (Tamil). It is a branched shrub which has greenish yellow flowers, the leaves’ length and width are between 2 and 5 cm, and it has an ovate form. It is widely grown in warm subtropical and tropical areas [18]. The whole plant is used in many Ayurvedic remedies and possesses several pharmaceutical properties such as antimicrobial, anti-inflammatory, antipyretic, hepatoprotective, wound healing, diuretic activity, antioxidative, analgesic activity, cytotoxic activity, and an immunomodulatory effect [19]. Our study pioneers an integrated approach combining metabolomics, network pharmacology, molecular docking, and molecular dynamics simulations to elucidate complex biochemical interactions and therapeutic targets. Metabolomics provides insights into physiological states and perturbations [20]. Network pharmacology maps interactions within biological networks, highlighting potential drug targets [21]. Molecular docking identifies promising ligand–receptor interactions, which are then refined through molecular dynamics simulations to assess the stability and dynamics of these complexes in a realistic environment [22]. This comprehensive framework enhances our understanding of molecular mechanisms and drug action, offering a robust strategy for drug discovery and development, with potential applications in personalized medicine and disease treatment. The objective of this study was to characterize the bioactive compounds present in the methanolic extract of *L. reticulata* using high-resolution liquid chromatography–quadrupole time-of-flight mass spectrometry (HR-LCMS/MS(Q-TOF)). The identified phytochemicals went through ADMET (absorption, distribution, metabolism, excretion, and toxicity) testing, utilizing online tools. Additionally, a network pharmacology analysis was conducted to elucidate the component–target–pathway interactions, thereby uncovering potential molecular mechanisms of action for the identified phytocompounds. Subsequent assessment of the therapeutic potential of these compounds involved molecular docking and molecular dynamics simulation experiments, facilitating the development of novel therapies for inflammatory conditions.

## 2. Results

### 2.1. Identification of Phytochemicals Using HR-LCMS/MS(Q-TOF)

Phytocompound separation and analysis were conducted using Q-TOF in both negative and positive modes, in conjunction with HR-LCMS/MS. Table 1 presents a comprehensive list of 113 identified phytocompounds detected in the methanolic extract. Notable among these compounds are kaempferol, known for its diverse medicinal properties such as anticarcinogenic, antibacterial, antifungal, antiprotozoal, anti-inflammatory, and antioxidant activities [23,24]; luteolin, which boasts numerous health benefits including cancer prevention, mitigation of oxidative stress, management of behavioral issues, neuroinflammation, inflammation, cardiovascular diseases, and its role in preventing metabolic disorders like diabetes, hepatic steatosis, and obesity [25]; ferulic acid, recognized for its versatility as a bioactive molecule with anti-inflammatory and antioxidant properties, offering some degree of protection against cardiovascular and renal diseases [26]; quercitrin, which exhibits a wide range of bioactivities including antioxidant effects, anti-inflammatory properties, antimicrobial activity, immune system regulation, pain reduction, wound healing promotion, and vasodilation [27]; catechin, known for its antibacterial, antitumor, antihypertensive, anticoagulant, and antiulcer properties [28]; and ellagic acid, possessing significant anti-inflammatory, anti-mutagenic, anti-proliferative, and antioxidant properties, showing promise in the treatment of various human ailments. Additionally, several other phytocompounds were identified, consistent with findings from previous studies [29].

### 2.2. ADMET Profiling

SwissADME, an online tool, was used to assess the phytocompounds’ pharmacokinetic and drug-likeness characteristics as well as their distribution, metabolism, excretion, and absorption capabilities. We predicted ADME profiling based on Lipinski’s rule of five, where compounds with a molecular weight less than 500, topological surface area (TSA) < 150, number of hydrogen bond donors < 150, quantity of donors for hydrogen bonds < 5, quantity of donors for hydrogen acceptors < 10, and two breaches of the rules are permitted. Except for 2,3,5,7,9-Pentathiadecane 2,2-dioxide, beta-D-3[5-Deoxy-5-(dimethylarsinyl)ribofuranosyloxy]-2-hydroxy-1-propanesulfonic acid, and Methyl(3×,10R)-dihydroxy-11-dodecene-6,8-diynoate 10-glucoside, all phytocompounds which cleared both ADME and toxicity exhibited excellent gastrointestinal absorption. It was predicted that certain compounds cannot pass through the blood–brain barrier (BBB), including Neotussilagine, 1-Pyrenylsulfate, 2,3,5,7,9-Pentathiadecane 2,2-dioxide, Kaempferol, Malic acid, (Z)-3-(1-Formyl-1-propenyl)pentanedioic acid, (1S,4R)-10-Hydroxyfenchone glucoside, beta-D-3[5-Deoxy-5-(dimethylarsinyl)ribofuranosyloxy]-2-hydroxy-1-propanesulfonic acid, and Albuterol, Methyl(3×,10R)-dihydroxy-11-dodecene-6,8-diynoate 10-glucoside. Pro Tox II was used to determine the toxicity, and apart from Neotussilagine, the majority of the phytocompounds would not be mutagenic, cytotoxic, carcinogenic, immunotoxic, or hepatotoxic. The substances with LD50 values greater than 2000 mg/kg indicated potential safety for usage as future medicines in in vivo research. Table 2 shows the list of compounds with the best ADME properties. Table 3 shows the list of compounds with the best toxicity profiling.

### 2.3. Network Pharmacology Analysis for Potential Active Compound Targets and Anti-Inflammatory Targets

**Figure 1:**
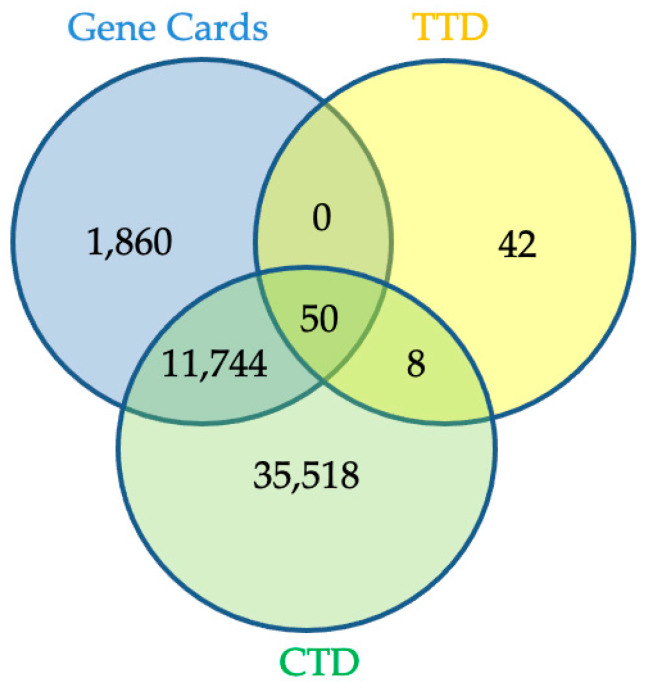
Target proteins for inflammation from different databases.

**Figure 2:**
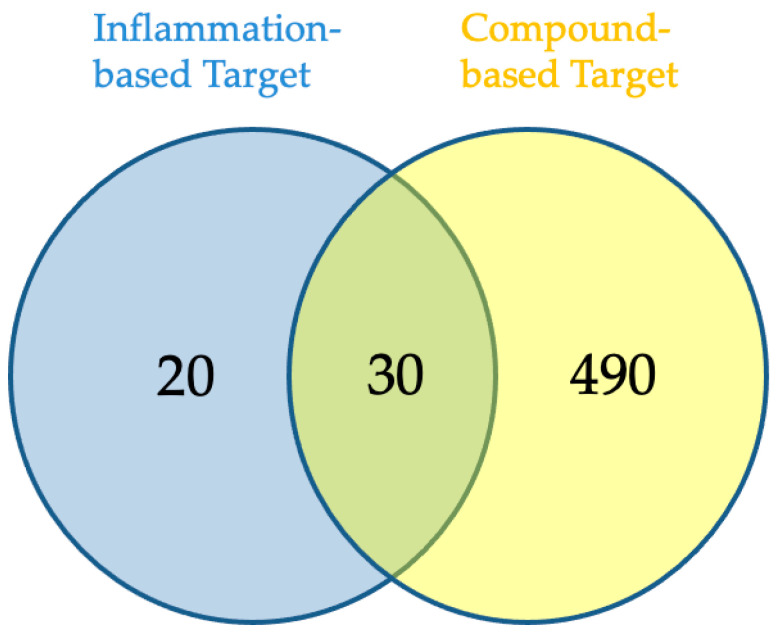
The common target proteins from compound-based and disease-based databases.

**Figure 3:**
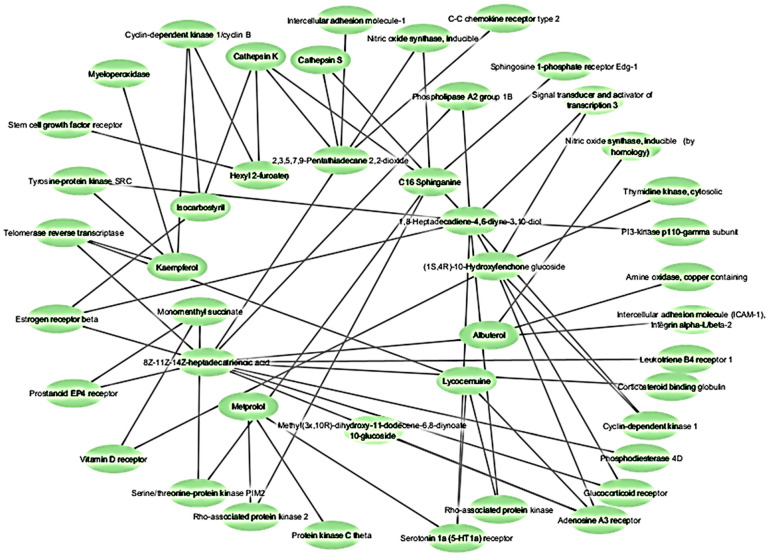
Interaction network between active components and intersection targets in *L. reticulata*.

Eighteen of the eighty compounds in the plant *L. reticulata* were chosen using Lipinski’s rule of five and ADME. A total of 520 potential targets could be found for 18 of the components combined using SwissTarget Prediction of having a likelihood greater than zero. Using the term “inflammation”, the associated genes were selected from the disease gene databases (GeneCards, CTD, TTD). After pooling the findings and removing duplicates, 50 records remained for screening, as shown in Figure 1. The Venn diagram (Figure 2) revealed 30 crossovers between the active compounds and inflammation-related targets. To create a compound–target network diagram, the targets and corresponding phytocompounds were loaded into Cytoscape 3.9.1. This network (Figure 3) shows the synergistic multi-component and multitargeted effects of the *L. reticulata* contributing to their anti-inflammatory activities. Table 4 shows the list of target proteins which play vital roles in inflammation.

### 2.4. Protein–Protein Interaction

**Figure 4:**
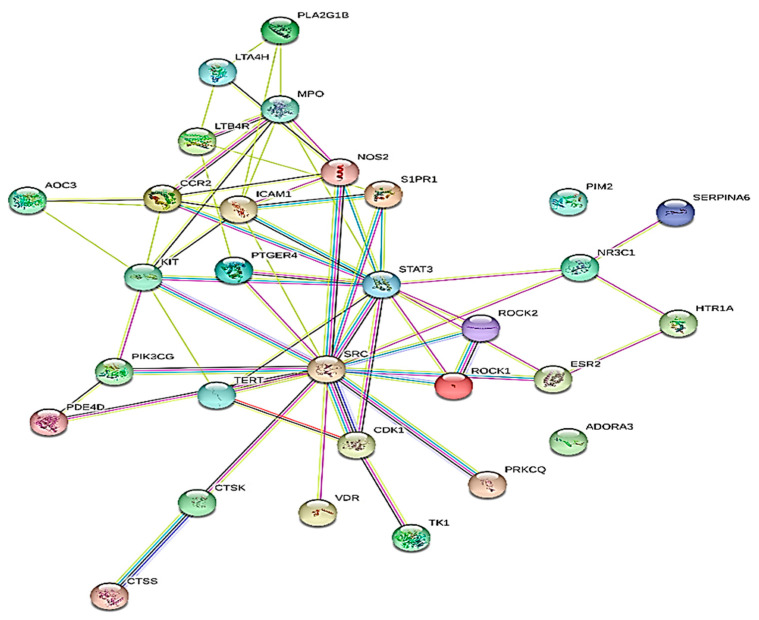
Protein–protein interaction (PPI) network of proteins as a target for anti-inflammation treatment.

**Figure 5:**
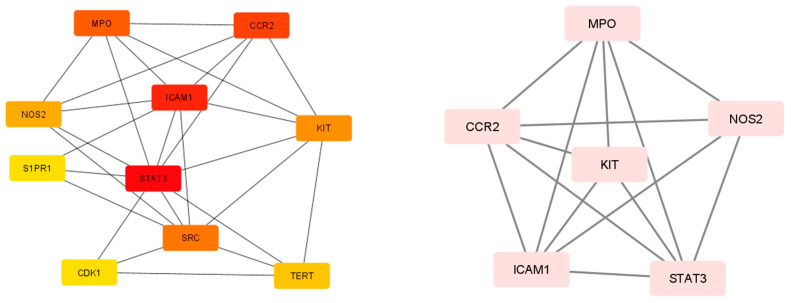
Modules in the PPI network of hub target proteins for anti-inflammatory treatment (The left Figure represents the interaction in Cytohuba plugin where the darker color represents that the protein has highest interaction score with the other proteins. The right figure represents the interaction using MCC algorithm).

Using the STRING database, a PPI network analysis was carried out to recognize the hub genes in critical modules. The selected results required an overall score of at least 0.4. Figure 4 shows the protein–protein interaction (PPI) network, which serves as a therapeutic target for the reduction of inflammation. Within the PPI network, the top 10 hub genes were selected using the MCC algorithm and the CytoHubba plugin. The top functional clusters of the module were chosen, as shown in Figure 5. By examining the MCC and M-CODE junction targets, six gene hubs were found (CCR2, ICAM1, KIT, MPO, NOS2, and STAT3). The eighteen metabolites showed anti-inflammatory effects, namely, receptors (C-C chemokine receptor type 2, stem cell growth factor receptor, and others), enzymes (myeloperoxidase, nitric oxide synthase, and others), and proteins (intercellular adhesion molecule-1, signal transducer and activator of transcription 3, and others).

### 2.5. GO Enrichment and KEGG Analysis

**Figure 6:**
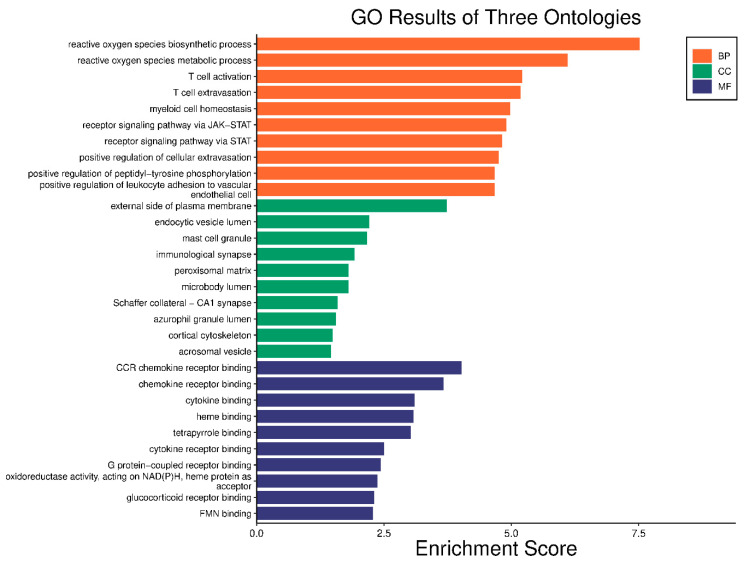
Biological process analysis of key hub targets.

**Figure 7:**
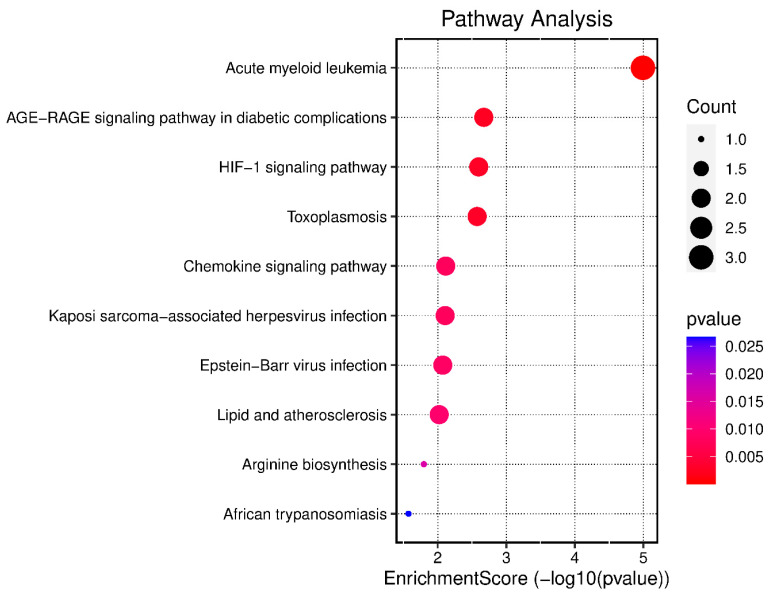
KEGG pathway enrichment analysis of key hub targets.

KEGG pathway enrichment and GO analysis were carried out on the six major targets using the DAVID 6.8 database. With the use of GO analysis, 24 GO items with *p* < 0.05 were found; they included entries for 17 biological processes, five cell component entries, and two molecular functions; Figure 6 shows the biological processes, cellular components, and gene molecular functions. The biological processes included a reactive oxygen species biosynthesis process, a reactive oxygen species metabolic process, T cell activation, and T cell extravasation. The cellular component enrichment included the external side of the plasma membrane, mast cell granules, immunological synapses, endocytic vesicle lumen, and microbody lumen. The molecular functions included CCR chemokine receptor binding, chemokine receptor binding, cytokine binding, and heme binding. Afterwards, an enrichment analysis of the KEGG pathway was carried out (Figure 7). Every route that had a *p* value less than 0.05 was screened and then sorted based on the *p* value. Acute myeloid leukemia, the AGE–RAGE signaling route in diabetic compilations, and the HIF-1 signaling pathway are the top three mechanisms.

### 2.6. Molecular Docking of Active Compounds and Key Targets

**Figure 8:**
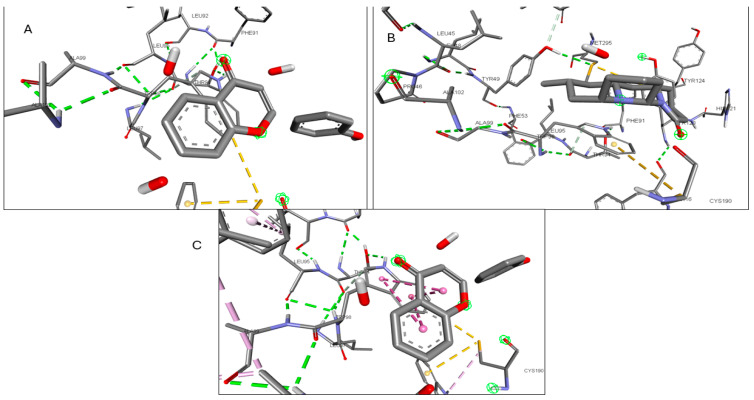
(**A**) 3D interaction of Kaempferol with CCR; (**B**) 3D interaction of Lycocernuine with CCR2; (**C**) 3D interaction of (1S,4R)-10-Hydroxyfenchone glucoside with CCR.

**Figure 9:**
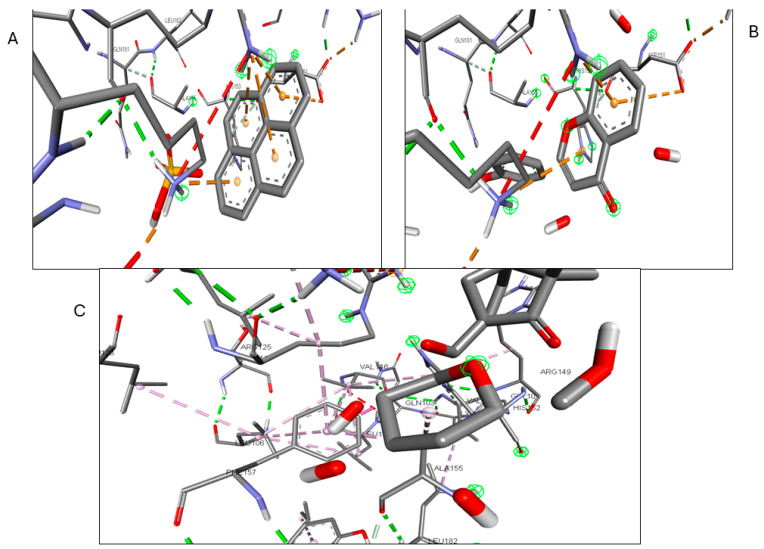
(**A**) 3D interaction of 1-Pyrenylsulfate with ICAM-1; (**B**) 3D interaction of Kaempferol with ICAM-1; (**C**) 3D interaction of (1S,4R)-10-Hydroxyfenchone glucoside with ICAM-1.

**Figure 10:**
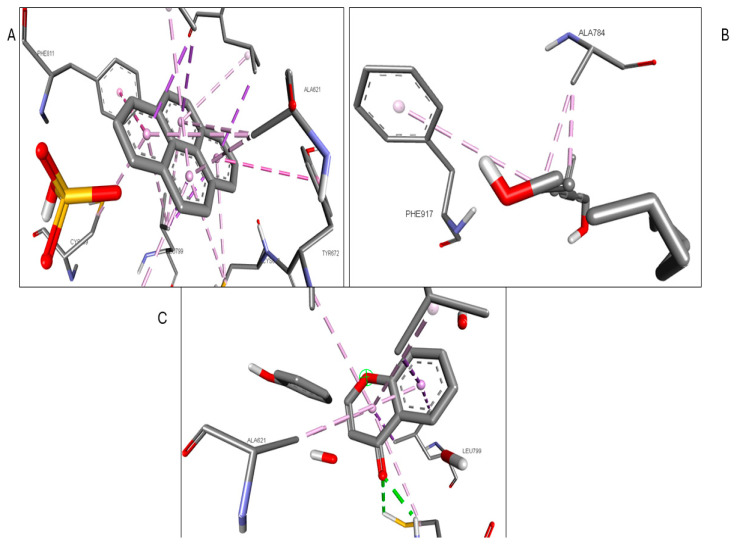
(**A**) 3D interaction of 1-Pyrenylsulfate with KIT; (**B**) 3D interaction of 1,8-Heptadecadiene-4,6-diyne-3,10-diol with KIT; (**C**) 3D interaction of Kaempferol with KIT.

**Figure 11:**
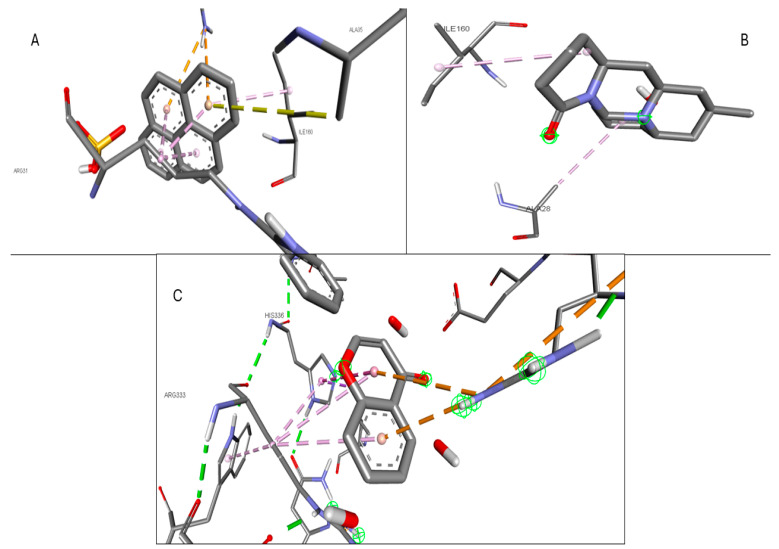
(**A**) 3D interaction of 1-Pyrenylsulfate with MPO; (**B**) 3D interaction of Lycocernuine with MPO; (**C**) Interaction residue of Kaempferol with MPO.

**Figure 12:**
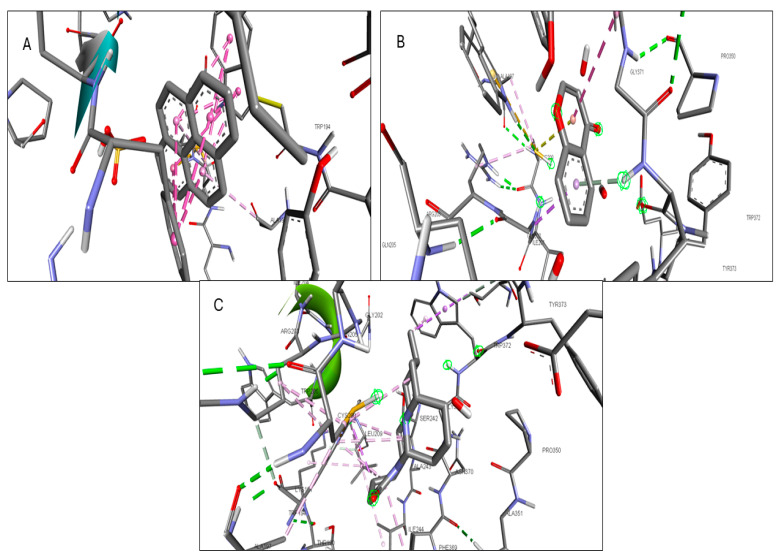
(**A**) 3D interaction of 1-Pyrenylsulfate with NOS2; (**B**) 3D interaction of Kaempferol with NOS2; (**C**) 3D interaction of Lycocernuine with NOS2.

**Figure 13:**
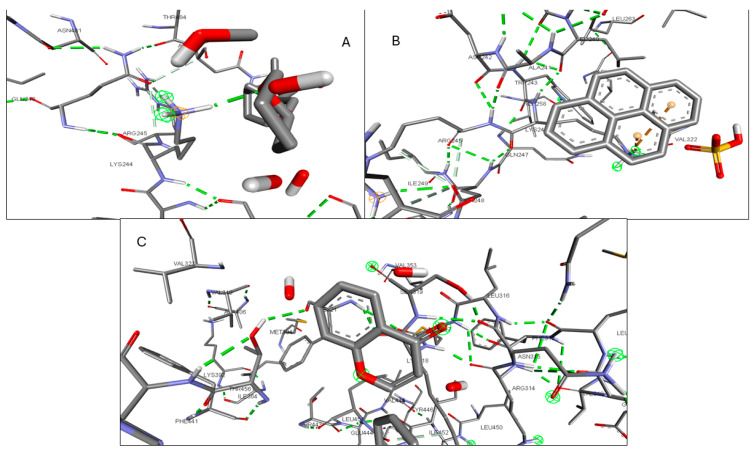
(**A**) 3D interaction of (1S,4R)-10-Hydroxyfenchone with STAT3; (**B**) 3D interaction of glucoside1-Pyrenylsulfate with STAT3; (**C**) 3D interaction of Kaempferol with STAT3.

Validation of the docking software was conducted by removing the crystallized ligand from the protein and then rebinding it to the same pocket. Supplementary Figure S1 illustrates the three-dimensional interaction between the ligand and the target proteins. The top six targets, ranked by degree, along with eighteen active components, were selected for molecular docking. According to convention, a binding energy score higher than 4.25 indicates a binding capability between the compounds and proteins. Scores exceeding 5.0 denote a relatively high binding affinity, while scores exceeding 7.0 indicate a strong ligand–receptor interaction. Table 5 presents the optimal binding affinities of the phytocompounds and proteins. Using the BIOVIA Discovery Studio 2024 Client Visualizer, interactions between amino acid residues and the ligand, as well as the types of forces involved, were investigated in both three-dimensional (Figure 8, Figure 9, Figure 10, Figure 11, Figure 12 and Figure 13) and two-dimensional formats (Supplementary Figures S2–S7).

### 2.7. Molecular Dynamics Simulation

**Figure 14:**
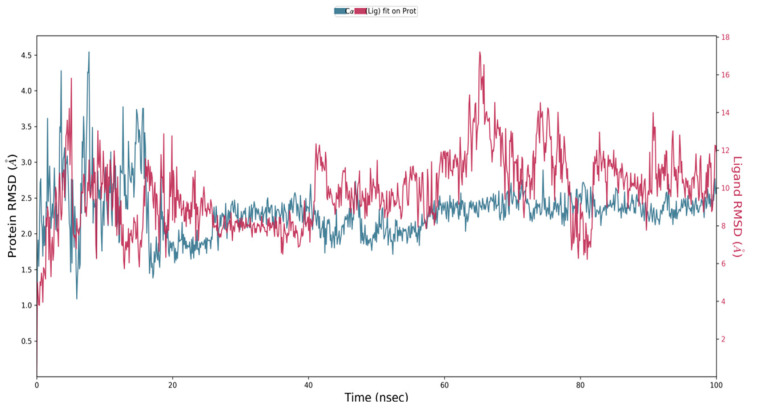
RMSD plot for (1S,4R)-10-Hydroxyfenchone glucoside–ICAM1 complex with the RMSD of the protein backbone and the molecular dynamics trajectory of 100 ns.

**Figure 15:**
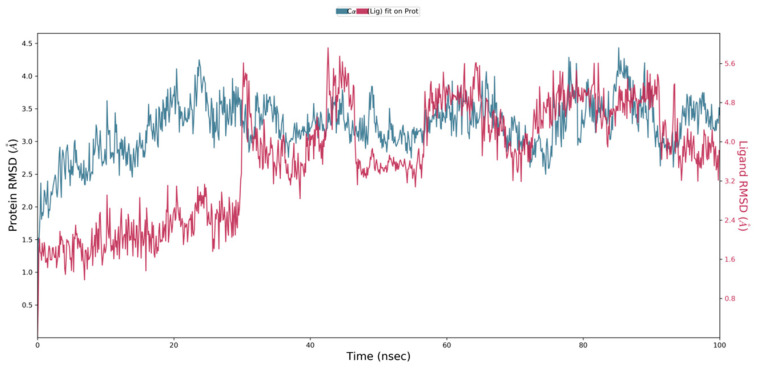
RMSD plot for (1S,4R)-10-Hydroxyfenchone glucoside–CCR2 complex with the RMSD of the protein backbone and the molecular dynamics trajectory of 100 ns.

**Figure 16:**
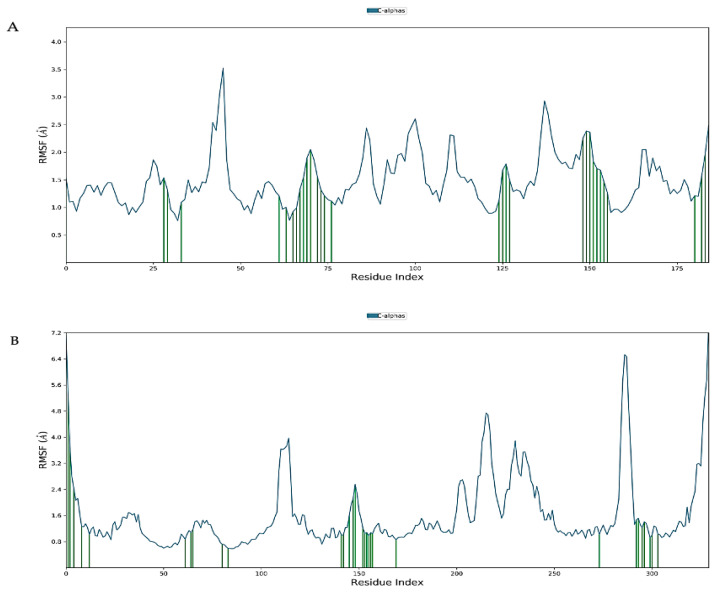
(**A**) RMSF plot of ICAM-1 protein chain with the ligand-bound state; (**B**) RMSF plot of CCR2 protein chain with the ligand-bound state (Blue color represents the residues or atoms with higher fluctuation and green color represents the lower residues or atoms fluctuating in the RMSF plot).

**Figure 17:**
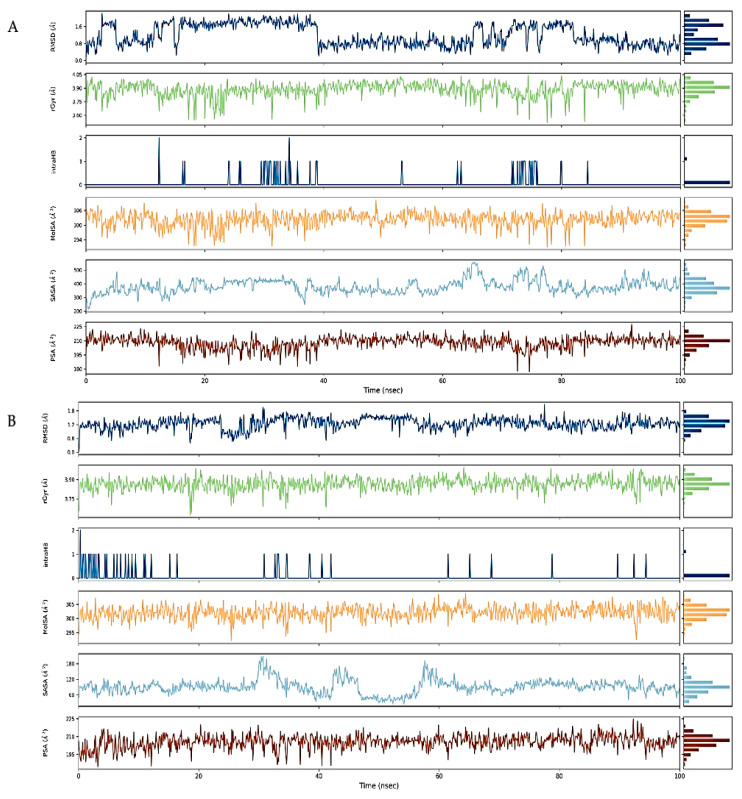
(**A**) RMSD, radius of gyration (rGyr), intramolecular hydrogen bond (intraHB), molecular surface area (MolSA), solvent accessible surface area (SASA), polar surface area (PSA) of the ligand–ICAM-1 protein complex as calculated during the 100 ns of MD simulation; (**B**) RMSD, radius of gyration (rGyr), intramolecular hydrogen bond (intraHB), molecular surface area (MolSA), solvent accessible surface area (SASA), polar surface area (PSA) of the ligand–CCR2 protein complex as calculated during the 100 ns of MD simulation.

**Figure 18:**
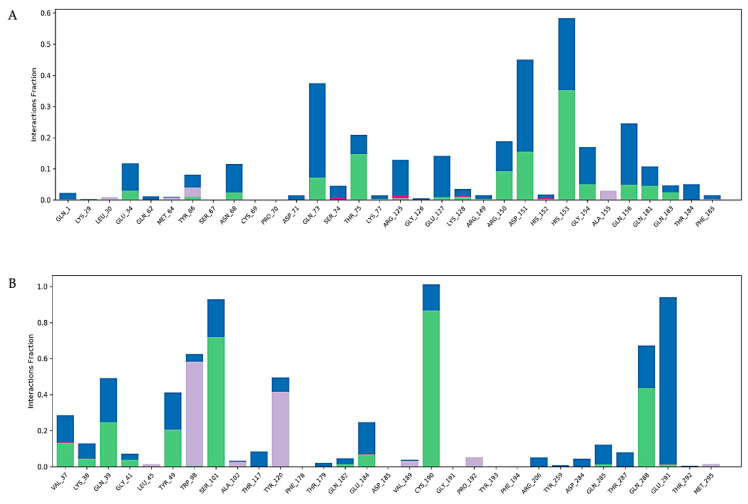
(**A**) The bar graph represents the interactions between (1S,4R)-10-Hydroxyfenchone glucoside–ICAM1 throughout the simulations of 100 ns, with different colors signifying the type of interactions between the amino acids and the ligand; (**B**) the bar graph represents the interactions between (1S,4R)-10-Hydroxyfenchone glucoside–CCR2 throughout the simulations of 100 ns, with different colors signifying the type of interactions between the amino acids and the ligand (Green color represents the hydrogen bond, lavender color represents hydrophobic bond and blue represents water bridges).

Virtual screening of phytocompounds from *L. reticulata* identified fifteen potent hub protein antagonists. Among these, (1S,4R)-10-Hydroxyfenchone glucoside, 1-Pyrenylsulfate, Kaempferol, and Lycocernuine exhibited the highest binding affinities. Consequently, the conformational stability of (1S,4R)-10-Hydroxyfenchone glucoside–ICAM1 and (1S,4R)-10-Hydroxyfenchone glucoside–CCR2 complexes were assessed through 100-nanosecond dynamics simulations. The structural integrity of the ligand–protein complex was evaluated using the root mean square deviation (RMSD) obtained from the MD simulation trajectory. For the (1S,4R)-10-Hydroxyfenchone glucoside–ICAM1 complex (depicted in Figure 14), there was initial stability observed between 0 and 40 nanoseconds, followed by fluctuation from 40 to nearly 80 nanoseconds. The ligand exhibited fluctuations ranging from 2.5 Å to 4.5 Å, while the protein showed lesser fluctuations, between 2.0 Å and 2.5 Å. The stability of the ligand within the binding site of the target persisted throughout the simulation, indicating prolonged interaction, which correlates with the observed high binding affinity from docking analysis. The simulation of the (1S,4R)-10-Hydroxyfenchone glucoside–CCR2 complex (illustrated in Figure 15) also showed minimal fluctuation, with stability observed from 30 nanoseconds onwards, exhibiting a deviation of 0.5 Å throughout the 100-nanosecond simulation period. A root-mean-square fluctuation (RMSF) analysis was employed to assess variations within the CCR2 and ICAM1 proteins. Peaks in the RMSF plot represent the regions of the protein with the highest degree of fluctuation during the simulations. Figure 16 illustrates the backbone atoms of the protein moiety (shown in blue) and the side chain atoms (in green), with RMSF values quantifying protein flexibility and fluctuation. Further evaluation of the protein–ligand complex during the 100-nanosecond simulation included analysis of the root mean square deviation (RMSD), polar surface area (PSA), molecular surface area (MolSA), radius of gyration (rGyr), and solvent-accessible surface area (SASA), as depicted in Figure 17. Supplementary Figure S8 illustrates the hydrogen bond formation process, while Figure 18 visualizes the interaction between protein–ligand complexes during molecular dynamics simulations. Supplementary Figure S9 represents the RMSF of protein ICAM 1 and CCR2. Supplementary Figure S10 provides a timeline depiction of the protein’s residues interactions with the ligand molecule.

## 3. Discussion

Inflammation serves as the body’s innate defense mechanism against various harmful substances and unpleasant stimuli. However, conventional anti-inflammatory medications often come with a host of adverse effects. Natural remedies have long been employed to alleviate inflammation, reflecting ancient practices. Throughout history, the use of medicinal herbs has been widely accepted as safe, cost effective, and prevalent. In Serbia, traditional medicine reigns as the predominant form of therapy, rooted in a steadfast belief in the healing properties of herbs. Numerous studies have highlighted the potent anti-inflammatory properties of several components found in *L. reticulata* [29].

The methanolic extract derived from *L. reticulata* underwent analysis through high-resolution liquid chromatography–mass spectrometry (HR-LCMS/MS) coupled with Q-TOF analysis, revealing a total of 260 compounds. Upon comparison of the high-resolution liquid chromatography and mass spectrum data with the MassHunter library, 113 compounds were successfully characterized and identified. These identifications relied on factors such as retention time, molecular formula, and mass. The chromatograms (Supplementary Figures S3–S8) provide significant insights into the relative amounts of various bioactive chemicals. Prominent phytocompounds confirmed through the HR-LCMS/MS(Q-TOF) analysis included Methyl N-methylanthranilate, Brassilexin, Kaempferol, Ferulic acid, Ellagic acid, Neuraminic acid, Hydroquinidine, Catechin, Madasiatic acid, Luteolin, Caulerpin, 2-Hexaprenyl-3-methyl-6-methoxy-1,4 benzoquinone, Malic acid, Quercitrin, Albuterol, Colnelenic acid, Lamprolobine, 1-Pyrenylsulfate, 14,19-Dihydroaspidospermatine, Muricatalin, Hexazinone, Tetradecyl sulfate, 9-HOTE, Isocarbostyril, 6-Methylquinoline, L-Tryptophan, and 2,4,6-Triethyl-1,3,5-trioxane. In a similar study on *Alangium salviifolium* bark, LC/Q-TOF analysis led to the identification of 449 compounds using the METLIN library.

ADMET studies play a crucial role in pharmaceutical research, providing a comprehensive assessment of a medication’s pharmacokinetics, including absorption, distribution, metabolism, excretion, and toxicity. Predicting a medication’s fate and its physiological impacts, such as oral and gastrointestinal absorption, is essential for drug development. Inadequate absorption can adversely affect distribution and metabolism, potentially leading to neurotoxicity and nephrotoxicity. Understanding a medication molecule’s distribution within an organism is a primary objective of research, making ADMET studies vital in computational drug design. In the current study, 18 phytocompounds from *L. reticulata* successfully underwent ADMET profiling. The observations indicated satisfactory oral absorption, along with appropriate solubility and absorption qualities, aligning with drug-like principles.

Network pharmacology was utilized to pinpoint the compounds primarily responsible for the anti-inflammatory effects of the phytocompounds. After undergoing the ADME filter procedure and adhering to Lipinski’s rule of five, eighteen compounds were chosen for target prediction. Analysis of the protein–protein interaction (PPI) network using MCODE revealed hub genes, including CCR2, ICAM1, KIT, MPO, NOS2, and STAT3, which are pivotal in inflammation, guiding immune cell migration, adhesion, and activation. Their roles range from facilitating leukocyte endothelial transmigration (ICAM1) to regulating inflammatory responses and signaling (STAT3). Targeting these proteins could significantly impact treating inflammatory and autoimmune diseases by modulating immune responses [30,31,32,33,34,35].

The investigation culminated in molecular docking and molecular dynamic simulations to elucidate the precise medicinal mechanism of phytocompounds found in the plant. The docking studies identified key interactions between proteins and compounds, suggesting paths for further investigation into their therapeutic effects. We found that the 1S,4R)-10-Hydroxyfenchone glucoside phytocompound had a great interaction with CCR2 and ICAM1. Due to this interaction, we took this complex forward for a molecular dynamics simulation study. Despite their complexity, the molecular dynamics simulations offered insights into the receptor–ligand interactions, emphasizing the role of water molecules in drug development. Changes in the root mean square deviation (RMSD) highlighted deviations in protein conformation, while the root mean square fluctuation (RMSF) values pointed to stability and strong hydrogen bonding, crucial for understanding compound efficacy.

Other phytocompounds with notable binding affinities with different targets include (1S,4R)-10-Hydroxyfenchone glucoside and Lycocernuin. Kaempferol, known for its anti-inflammatory properties, has been validated in various studies as a safe and effective natural dietary anti-inflammatory agent. Conversely, (1S,4R)-10-Hydroxyfenchone glucoside boasts antioxidant, antimicrobial, and other bioactive effects, warranting further investigation to confirm its connection and underlying mechanisms.

Overall, this research underscores the anti-inflammatory properties of *Leptadenia reticulata* methanolic extract, mediated through the synergy of multiple components, targets, and pathways. Network pharmacology played a pivotal role in elucidating this mechanism of action, offering valuable insights for future therapeutic development.

## 4. Materials and Methods

### 4.1. Plant Material

Plant samples were collected from Hosur, Tamil Nadu and were duly authenticated by Dr. Senthilkumar Umapathy (https://mcc.edu.in/; accessed on 9 September 2023; senthilumapathy@mcc.edu.in), Assistant Professor, Department of Botany at Madras Christian College campus.

### 4.2. Extraction of Phytochemicals from L. reticulata

The leaves, stem, and root of *Leptadenia reticulata* were shade dried for two weeks at room temperature. The dried samples were ground finely using liquid nitrogen and 1 mL of 99.9% methanol was added to 100 mg of sample and stored at −20 °C overnight. The sample was subjected to water bath sonication for 15 min at 35 °C, and then stored at −4 °C overnight [36,37]. After 24 h, the sample was centrifuged at 10,000 rpm for 10 min. The centrifuged sample was then filtered using a syringe filter (PVDF/L 0.22 µm) and stored at −20 °C for further analysis.

### 4.3. Identification of Phytochemicals Using HR-LCMS/MS(Q-TOF)

Identification of secondary metabolites from *Leptadenia reticulata* was achieved using an Agilent 6550 iFunnel Q-TOF (Agilent Technologies, Santa Clara, CA, USA), with both positive and negative modes [38]. Hypersil Gold C-18 (3 µm particle size, 2.1 mm internal diameter, and 100 mm length) was used for the separation of secondary metabolites. A flow rate of 300 µL/min was used. An aliquot of 3 µL was injected independently. 100% acetonitrile with 100% methanol made up mobile phase B, whereas 0.1% formic acid in water made up mobile phase A [39]. With the following parameters, a complete scan mode was attained in the 100–1000 amu range: capillary voltage (3500 V); nozzle voltage (1000 V); 13 L/min gas flow rate at 300 °C; and nebulization set at 35 psi. Mass Hunter Workstation was used for identification of secondary metabolites based on the *m*/*z* (mass/charge) values and spectrum graph.

### 4.4. Active Ingredient Screening

The SwissADME web server (http://www.swissadme.ch/; accessed on 29 September 2023) was used to conduct the in silico ADME toxicity and drug-likeness assessments on the discovered phytocompounds. To evaluate the drug-likeness of the compound various parameters were computed, including number of hydrogen bond donors and acceptors, molecular weight, molecule polar surface area, Veber’s rule, the logarithm of the n-octanol/water partition coefficient(logP), and Lipinski’s rule of five [40]. Additionally, ADMET prediction was carried out while considering elements including cytochrome P450(CYP) 2D6 inhibition, plasma membrane binding, blood–brain barrier penetration, aqueous solubility, and hepatotoxicity [16]. The ProTox II (https://tox-new.charite.de/; accessed on 2 October 2023) online server was used to calculate the LD50 value of the phytocompounds and to predict organ toxicity.

### 4.5. Inflammation-Related Target and Associated Drug Target Screening

The SwissTargetPrediction tool (http://www.swisstargetprediction.ch; accessed on 7 October 2023) was used to screen the target corresponding to the component and screen the targets based on the possible criteria before (probability > 0) merging the targets and removing repeated values [41]. In order to find relevant targets, we used the search term “inflammation” to gather and combine targets from the Human Gene Database (GeneCards; https://www.genecards.org/; accessed on 9 October 2023) [42], Therapeutic Target Database (TTD; https://db.idrblab.net/ttd/; accessed on 9 October 2023) [43], and Comparative Toxicogenomics Database (CTD; http://ctdbase.org/; accessed on 9 October 2023) [44]. Then, the target dataset was imported into the tool called Venny2.1.0 (https://bioinfogp.cnb.csic.es/tools/venny/index.html; accessed on 12 October 2023) to create a Venn diagram that showed the intersection of the identified drug targets and disease, i.e., the potential targets of phytocompounds against inflammation-related diseases.

### 4.6. Protein–Protein Interaction

To build a protein–protein interaction (PPI) network, the common targets were imported into the STRING 12.0 database (https://www.string-db.org; accessed on 13 October 2023) [45]. Subsequently, the acquired data were transferred into Cytoscape 3.9.1. (http://cytoscape.org; accessed on 13 October 2023) for topological analysis to screen out the primary targets of diseases associated with inflammation [46].

### 4.7. GO Enrichment and KEGG Analysis

GO function and KEGG (https://www.kegg.jp/kegg/kegg1.html; accessed on 14 October 2023) pathway enrichment analysis were carried out using DAVID (https://david.ncifcrf.gov/; accessed on 14 October 2023) [47] using *Homo sapiens* as the selected species. SRplots (https://www.bioinformatics.com.cn/en; accessed on 14 October 2023) was used to visualize the GO function and KEGG pathway enrichment. The threshold point was set at *p* < 0.05 for all GO enrichment and pathway studies [48]. The final pathway map was created by integrating and plotting the top-ranked paths.

### 4.8. Molecular Docking

The interactions between the target protein and the bioactive phytocompounds identified in the HR-LCMS/MS(Q-TOF) investigation of *L. reticulata* were determined using the PyRx-0.8 software. The RCBS Protein Data Bank (https://www.rcsb.org/; accessed on 17 October 2023) was accessed to obtain the target protein’s 3D structures (PDB ID: 1IAM, 1T46, 4NOS, 4C1M, 6NJS, 6GPS) [49]. Using BIOVIA Discovery Studio, the protein structures were altered by removing water and hydrogen atoms; later the Chimera 1.16 tool was used to add polar hydrogen bonds. Subsequently, PyRx was utilized to convert the structures into PDBQT format [50,51,52]. The SDF format of the found compounds’ structures were downloaded from PubChem (https://pubchem.ncbi.nlm.nih.gov/; accessed on 17 October 2023) [53] and PyRx was used to minimize the energy and convert it into the PDBQT format. Table 6 gives the list of phytocompounds downloaded to perform molecular docking. PyRx was used to carry out the molecular docking. The validation of the docking software was achieved by removing the crystallized ligand from the pocket and rebinding it to the same pocket. For docking studies, the size of the grid box was selected to encompass the active site of the protein. The best docked posture for the ligand and protein was chosen based on the binding affinity, and it was then displayed in 2D analysis using Discovery Studio to identify the residues interacting with different bonds.

### 4.9. Molecular Docking Simulations

Molecular dynamics (MD) simulation is a computer technique used in drug design to generate a trajectory that monitors the movements of molecules over time [54]. In this study, we studied the interactions of the highest-scoring protein–ligand complexes using Maestro Schrodinger 2017v1 during a period of 100 ns.

The simulation model utilized an explicit solvent system, employing the four-point TIP4P rigid water model. Additionally, a crystalline system with a unit cell in the form of a right prism with a rectangular base, known as an orthorhombic box, was used for model preparation. Sodium and chloride ions (Na^+^ and Cl^−^) were introduced at a pressure of 1.01325 bar and a temperature of 310 K to neutralize the model. The simulation was conducted using the OPLS-2005 force field [52]. The simulation was run for 100 nanoseconds. The trajectory created was evaluated to determine the root mean square deviation (RMSD), root mean square fluctuation (RMSF), hydrogen mapping, and radius of gyration [51].

## 5. Conclusions

Traditional medicine often relies on plants like *L. reticulata*, which contain a myriad of bioactive compounds yet we lack a comprehensive understanding of their therapeutic mechanisms. To address this gap, our study employed HR-LCMS/MS(Q-TOF), unveiling 113 constituents within the methanolic extract of *L. reticulata*’s leaves, stems, and roots. Among these compounds, flavonoids, organic acids, and polyphenols emerged as predominant components, hinting at their potential therapeutic significance. Through network pharmacology, we identified 18 compounds and six targets implicated in the plant’s bioactivity, notably targeting inflammation. Among these, neotussilagine, kaempferol, and (1S,4R)-10-Hydroxyfenchone glucoside stood out for their potential anti-inflammatory properties. These findings align with the traditional use of *L. reticulata* in managing inflammatory conditions, shedding light on its molecular underpinnings. Of particular interest was the strong binding affinity demonstrated by (1S,4R)-10-Hydroxyfenchone glucoside towards CCR2 and ICAM-1, key players in inflammatory pathways. This suggests a promising avenue for targeted medication design against inflammatory disorders, potentially paving the way for novel therapeutics. Moreover, our investigation highlighted several signaling pathways implicated in the anti-inflammatory effects of *L. reticulata* compounds. Notably, the HIF-1 and AGE–RAGE signaling pathways, along with acute myeloid leukemia pathways, emerged as potential targets for modulating inflammation. These insights provide a roadmap for future research into the mechanisms underlying *L. reticulata*’s anti-inflammatory properties, offering new avenues for drug development and therapeutic intervention. However, while molecular docking studies revealed promising interactions between *L. reticulata* compounds and their protein targets, further validation through in vitro and in vivo studies is essential. These experiments will provide a deeper understanding of the efficacy and safety profiles of these compounds, ultimately translating into tangible clinical benefits for individuals suffering from inflammatory conditions. In summary, our study provides valuable insights into the bioactive potential of *L. reticulata* and lays the groundwork for future research aimed at harnessing its anti-inflammatory properties for therapeutic purposes.

## Figures and Tables

**Table 1:** List of phytocompounds identified by HR-LCMS/MS(Q-TOF) from root, stem, and leaves of *Leptadenia reticulata*.

Serial No	Compound Name	*m*/*z*	Molecular Formula	Expressed in Root (R), Leaf (L), Stem (S)
1	Brassilexin	175.03	C_9_ H_6_ N_2_ S	S
2	1-Pyrenylsulfate	299.04	C_16_ H_10_ O_4_ S	R
3	12-Tridecene-4,6,8,10tetraynal	203.048	C_13_ H_8_ O	S
4	Methyl N-methylanthranilate	188.066	C_9_ H_11_ N O₂	S, L, R
5	Fenapanil	254.169	C_16_ H_19_ N_3_	S
6	2,4,6-Triethyl-1,3,5-trioxane	197.113	C_9_ H_18_ O_3_	S, L
7	11-Methoxy-vinorine	387.172	C_22_ H_24_ N_2_ O_3_	S
8	Naltrindole	415.203	C_26_ H_26_ N_2_ O_3_	S
9	Hydroxyprolyl-Alanine	203.102	C_8_ H_14_ N_2_ O_4_	S
10	5-Acetoxydihydrotheaespirane	277.174	C_15_ H_26_ O_3_	S, R
11	Monomenthyl succinate	279.153	C_14_ H_24_ O_4_	S
12	Grossamide	625.254	C_36_ H_36_ N_2_ O_8_	S
13	Somniferine	609.259	C_36_ H_36_ N_2_ O_7_	S
14	Campestanol	425.369	C_28_ H_50_ O	S
15	Octadecyl fumarate	391.277	C_22_ H_40_ O_4_	S
16	Archaeol	675.663	C4_3_ H_88_ O_3_	S
17	1-Eicosanol	321.308	C_20_ H_42_ O	S
18	Chinomethionat	256.979	C_10_ H_6_ N_2_ O S_2_	L
19	Neotussilagine	222.111	C_10_ H_17_ N O_3_	L, R
20	Neuraminic acid	268.102	C_9_ H_17_ N O_8_	L, R
21	Gabapentin	172.132	C_9_ H_17_ N O_2_	L, R
22	1-Phenylbiguanide	200.09	C_8_ H_11_ N_5_	L
23	L-Tryptophan	205.095	C_11_ H_12_ N_2_ O_2_	L, R
24	6-Methylquinoline	144.08	C_10_ H_9_ N	L, R
25	Isocarbostyril	146.059	C_9_ H_7_ N O	L, R
26	Methyprylon	206.116	C_10_ H_17_ N O_2_	L
27	Pirbuterol	263.137	C_12_ H_20_ N_2_ O_3_	L
28	2-Ethyl-5-methylpyridine	144.079	C_8_ H_11_ N	L
29	Citrinin	273.073	C_13_ H_14_ O_5_	L
30	[2,2-bis (2-methylpropoxy) ethyl]benzene	273.178	C_16_ H_26_ O_2_	L
31	Hexyl 2-furoate	197.116	C_11_ H_16_ O_3_	L
32	Maritimetin	287.053	C_15_ H_10_ O_6_	L
33	Ismine	258.113	C_15_ H_15_ N O_3_	L
34	Lenacil	257.126	C_13_ H_18_ N_2_ O_2_	L
35	3-Hydroxynonyl acetate	225.147	C_11_ H_22_ O_3_	L, R
36	C16 Sphinganine	274.273	C_16_ H_35_ N O_2_	L, R
37	Symlandine	404.203	C_20_ H_31_ N O_6_	L
38	Lauroyl diethanolamide	288.251	C_16_ H_33_ N O_3_	L, R
39	Gibberellin A74	387.177	C_20_ H_28_ O_6_	L, R
40	Thiamylal	255.12	C_12_ H_18_ N_2_ O_2_ S	L
41	2,6-Di-tert-butyl-4-ethylphenol	257.188	C_16_ H_26_ O	L
42	Nigakilactone B	415.208	C_22_ H_32_ O_6_	L, R
43	Sphinganine	302.303	C_18_ H_39_ N O_2_	L, R
44	18-Nor-4(19),8,11,13abietatetraene	277.195	C_19_ H_26_	L
45	Oxidized dinoflagellate luciferin	625.261	C_33_ H_38_ N_4_ O_7_	L
46	Irinotecan	609.266	C_33_ H_38_ N_4_ O_6_	L
47	3-[(3-Methylbutyl) nitrosoamino]-2butanone	187.142	C_9_ H_18_ N_2_ O_2_	R
48	Metoprolol	268.188	C_15_ H_25_ N O_3_	R
49	(Z)-3-(1-Formyl-1propenyl) pentanedioic acid	223.058	C_9_ H_12_ O_5_	R
50	Luciduline	208.168	C_13_ H_21_ N O	R
51	methyl (2E,6E,10R,11S)-10,11epoxy-3,7,11-trimethyltrideca-2,6-dienoate	303.193	C_17_ H_28_ O_3_	R
52	3-Hydroxy-4-deoxypaxilline	444.246	C_27_ H_35_ N O_3_	R
53	Licoagrodione	357.131	C_20_ H_20_ O_6_	R
54	Gibberellin A91	387.14	C_19_ H_24_ O_7_	R
55	Riesling acetal	249.147	C_13_ H_22_ O_3_	R
56	11-Hydroxy-9-tridecenoic acid	251.162	C_13_ H_24_ O_3_	R
57	Ginsenoyne D	285.183	C_17_ H_26_ O_2_	R
58	Sulfadimidine	279.091	C_12_ H_14_ N_4_ O_2_ S	R
59	8Z,11Z,14Z-heptadecatrienoic acid	287.198	C_17_ H_28_ O_2_	R
60	1,8-Heptadecadiene-4,6-diyne-3,10-diol	283.167	C_17_ H_24_ O_2_	R
61	Albuterol	262.142	C_13_ H_21_ N O_3_	R
62	Caffeic aldehyde	163.042	C_9_ H_8_ O_3_	L
63	Quercitrin	447.096	C_21_ H_20_ O_11_	L
64	Kaempferol	285.042	C_15_ H_10_ O_6_	L
65	Luteolin	285.042	C_15_ H_10_ O_6_	L
66	Colnelenic acid	291.199	C_18_ H_28_ O_3_	L
67	9-HOTE	293.214	C_18_ H_30_ O_3_	L, R
68	Ferulic acid	193.052	C_10_ H_10_ O_4_	L
69	Ellagic acid	301.001	C_14_ H_6_ O_8_	L
70	Malic acid	133.015	C₄ H₆ O₅	L
71	Ribose-1-arsenate	272.961	C_5_ H_11_ As O_8_	L, S
72	2,3,5,7,9-Pentathiadecane 2,2-dioxide	262.932	C_5_ H_12_ O_2_ S_5_	L
73	Apigenin 7-[rhamnosyl-(1->2)-galacturonide]	591.139	C_27_ H_28_ O_15_	L
74	CMP-N-glycoloylneuraminate	629.132	C_20_ H_31_ N_4_ O_17_ P	L
75	Nicotiflorin	593.156	C_27_ H_30_ O_15_	L
76	Genistein 8-C-glucoside	431.102	C_21_ H_20_ O_10_	L
77	Biorobin	593.156	C_27_ H_30_ O_15_	L
78	Glafenine	431.102	C_19_ H_17_ Cl N_2_ O_4_	L
79	Tetradecyl sulfate	293.178	C_14_ H_30_ O_4_ S	L, R
80	Hexazinone	297.156	C_12_ H_20_ N_4_ O_2_	L, S
81	Magnesium protoporphyrin monomethyl ester		C_35_ H_34_ Mg N_4_ O_4_	L
82	Lamprolobine	309.177	C_15_ H_24_ N_2_ O_2_	L, R
83	Kanokoside D	623.257	C_27_ H_44_ O_16_	L
84	19-Hydroxycinnzeylanol 19-glucoside	607.261	C_26_ H_42_ O_13_	L
85	Muricatalin	671.471	C_35_ H_64_ O_8_	L, S
86	14,19-Dihydroaspidospermatine	339.203	C_21_ H_28_ N_2_ O_2_	L, R
87	Lycocernuine	337.209	C_16_ H_26_ N_2_ O_2_	L
88	Malvalic acid	339.256	C_18_ H_32_ O_2_	R
89	6-Feruloylglucose 2,3,4-trihydroxy-3-methylbutylglycoside	473.168	C_21_ H_30_ O_12_	R
90	Lusitanicoside	487.184	C_21_ H_30_ O_10_	R
91	Thiazopyr	455.106	C_16_ H_17_ F_5_ N_2_ O_2_ S	R
92	beta-D-3-[5-Deoxy-5-(dimethylarsinyl)ribofuranosyl oxy]-2-hydroxy-1-propanesulfonic acid	451.023	C_10_ H_21_ As O_9_ S	R
93	Tosyllysine chloromethyl ketone	377.09	C_14_ H_21_ Cl N_2_ O_3_ S	R
94	Dictyoquinazol C	341.113	C_18_ H_18_ N_2_ O_5_	R
95	Methyl (3×,10R)-dihydroxy-11-dodecene-6,8-diynoate 10-glucoside	443.16	C_19_ H_26_ O_9_	R
96	Haemocorin	687.199	C_32_ H_34_ O_14_	R
97	Sudachiin A	521.136	C_24_ H_26_ O_13_	R
98	4-(4-Hydroxyphenyl)-2-butanone O-[2-galloyl-6-p-coumaroylglucoside]	669.189	C_32_ H_32_ O_13_	R
99	Phytolaccoside E	825.437	C_42_ H_66_ O_16_	R
100	(1S,4R)-10-Hydroxyfenchone glucoside	329.157	C_16_ H_26_ O_7_	R
101	Madasiatic acid	487.348	C_30_ H_48_ O_5_	R, S
102	Provincialin	517.207	C_27_ H_34_ O_10_	R
103	2-Hexaprenyl-3-methyl-6-methoxy-1,4 benzoquinone	605.411	C_38_ H_56_ O_3_	R
104	Omega-hydroxy behenic acid	335.326	C_22_ H_44_ O_3_	R
105	Catechin	349.094	C_15_ H_14_ O_6_	S
106	Cauleprin	457.141	C_24_ H_18_ N_2_ O_4_	S
107	Dracorubin	487.152	C_32_ H_24_ O_5_	S
108	1,4-beta-D-Glucan	595.174	C_18_ H_32_ O_18_	S
109	Daidzin 4′-O-glucuronide	591.143	C_27_ H_28_ O_15_	S
110	Aurasperone C	591.144	C_31_ H_28_ O_12_	S
111	Nb-Stearoyltryptamine	471.355	C_28_ H_46_ N_2_ O	S
112	Tetrahexosylceramide (d18:1/24:0)	668.442	C_68_ H_126_ N_2_ O_23_	S
113	Hydroquinidine	325.19	C_20_ H_26_ N_2_ O_2_	S

**Table 2:** List of compounds with the best ADME profiling.

Molecule	Molecular Formula	MW g/mol	No. of H-Bond Acceptors	No. of H-Bond Donors	TPSA	GI Absorption	BBB Permeant	No. of Lipinski Violations
Neotussilagine	C_10_ H_17_ NO_3_	199.25	4	1	49.77	High	No	0
Isocarbostyril	C_9_ H_7_ N O	145.16	1	1	32.86	High	Yes	0
Hexyl 2-furoateṇ	C_11_ H_16_ O_3_	196.24	3	0	39.44	High	Yes	0
1-Pyrenylsulfate	C_16_ H_10_ O_4_ S	298.31	4	1	71.98	High	No	0
C16 Sphinganine	C_16_ H_35_ N O_2_	273.45	3	3	66.48	High	Yes	0
2,3,5,7,9-Pentathiadecane 2,2-dioxide	C_5_ H_12_ O_2_ S_5_	264.47	2	0	143.72	Low	No	0
Lycocernuine	C_16_ H_26_ N_2_ O_2_	278.39	3	1	43.78	High	Yes	0
Kaempferol	C_15_ H_10_ O_6_	286.24	6	4	111.13	High	No	0
Malic acid	C_4_ H_6_ O_5_	134.09	5	3	94.83	High	No	0
Metoprolol	C_15_ H_25_ N O_3_	267.36	4	2	50.72	High	Yes	0
(Z)-3-(1-Formyl-1-propenyl)pentanedioic acid	C_9_ H_12_ O_5_	200.19	5	2	91.67	High	No	0
(1S,4R)-10-Hydroxyfenchone glucoside	C_16_ H_26_ O_7_	330.37	7	4	116.45	High	No	0
beta-D-3[5-Deoxy-5-(dimethylarsinyl)ribofuranosyloxy]-2-hydroxy-1-propanesulfonic acid	C_10_ H_21_ As O_9_ S	392.25	9	4	161.96	Low	No	0
Albuterol	C_13_ H_21_ N O_3_	239.31	4	4	72.72	High	No	0
8Z-11Z-14Z-heptadecatrienoic acid	C_17_ H_28_ O_2_	264.4	2	1	37.3	High	Yes	0
Methyl(3×,10R)-dihydroxy-11-dodecene-6,8-diynoate 10-glucoside	C_19_ H_26_ O_9_	398.4	9	5	145.91	Low	No	0
Monomenthyl succinate	C_14_ H_24_ O_4_	256.34	4	1	63.6	High	Yes	0
1,8-Heptadecadiene-4,6-diyne-3,10-diol	C_17_ H_24_ O_2_	260.37	2	2	40.46	High	Yes	0

**Table 3:** List of compounds with best toxicity profiling.

Compound Name	Oral LD50 Value (mg/kg)	Predicted Toxicity Class	Hepatotoxicity	Carcinogenicity	Immunotoxicity	Mutagenicity	Cytotoxicity
Neotussilagine	1240	4	Inactive (−0.92)	Active (−0.59)	Inactive (−0.98)	Inactive (−0.75)	Inactive (−0.72)
Isocarbostyril	360	4	Inactive (−0.51)	Inactive (−0.58)	Inactive (−0.99)	Inactive (−0.66)	Inactive (−0.85)
Hexyl 2-furoateṇ	1500	4	Inactive (−0.8)	Active (−0.51)	Inactive (−0.9)	Inactive (−0.84)	Inactive (−0.74)
1-Pyrenylsulfate	2793	5	Inactive (−0.73)	Inactive (−0.73)	Inactive (−0.83)	Inactive (−0.79)	Inactive (−0.83)
C16 Sphinganine	3500	5	Inactive (−0.76)	Inactive (−0.54)	Inactive (−0.99)	Inactive (−0.9)	Inactive (−0.71)
2,3,5,7,9-Pentathiadecane 2,2-dioxide	3200	5	Inactive (−0.69)	Inactive (−0.64)	Inactive (−0.99)	Inactive (−0.62)	Inactive (−0.78)
Lycocernuine	4000	5	Inactive (−0.73)	Inactive (−0.61)	Inactive (−0.84)	Inactive (−0.74)	Inactive (−0.74)
Kaempferol	3919	5	Inactive (−0.68)	Inactive (−0.72)	Inactive (−0.96)	Inactive (−0.52)	Inactive (−0.98)
Malic acid	2497	5	Inactive (−0.9)	Inactive (−0.71)	Inactive (−0.99)	Inactive (−0.97)	Inactive (−0.74)
Metprolol	1050	4	Inactive (−0.94)	Inactive (−0.82)	Inactive (−0.88)	Inactive (−0.93)	Inactive (−0.73)
(Z)-3-(1-Formyl-1-propenyl)pentanedioic acid	2140	5	Inactive (−0.73)	Inactive (−0.73)	Inactive (−0.99)	Inactive (−0.9)	Inactive (−0.69)
1,8-Heptadecadiene-4,6-diyne-3,10-diol	5600	6	Inactive (−0.7)	Inactive (−0.65)	Inactive (−0.95)	Inactive (−0.95)	Inactive (−0.79)
8Z-11Z-14Z-heptadecatrienoic acid	10,000	6	Inactive (−0.54)	Inactive (−0.63)	Inactive (−0.99)	Inactive (−0.95)	Inactive (−0.71)
Albuterol	660	4	Inactive (−0.98)	Inactive (−0.86)	Inactive (−0.88)	Inactive (−0.75)	Inactive (−0.66)
beta-D-3[5-Deoxy-5-(dimethylarsinyl)ribofuranosyloxy]-2-hydroxy-1-propanesulfonic acid	8000	6	Inactive (−0.78)	Inactive (−0.68)	Inactive (−0.84)	Inactive (−0.54)	Inactive (−0.72)
(1S,4R)-10-Hydroxyfenchone glucoside	190	3	Inactive (−0.9)	Inactive (−0.83)	Inactive (−0.96)	Inactive (−0.7)	Inactive (−0.63)
Methyl(3×,10R)-dihydroxy-11-dodecene-6,8-diynoate 10-glucoside	10,000	6	Inactive (−0.87)	Inactive (−0.83)	Inactive (−0.99)	Inactive (−0.69)	Inactive (−0.76)
Monomenthyl succinate	930	4	Inactive (−0.63)	Inactive (−0.66)	Inactive (−0.99)	Inactive (−0.86)	Inactive (−0.81)

**Table 4:** List of target proteins which plays vital role in inflammation.

Serial No.	Target	Common Name	Uniport ID
1	Intercellular adhesion molecule 1	ICAM1	P05362
2	Signal transducer and activator of transcription 3	STAT3	P40763
3	Myeloperoxidase	MPO	P05164
4	Nitric oxide synthase, inducible	NOS2	P35228
5	PI3-kinase p110-gamma subunit	PIK3CG	P48736
6	Tyrosine-protein kinase SRC	SRC	P12931
7	Vitamin D receptor	VDR	P11473
8	Glucocorticoid receptor	NR3C1	P04150
9	Leukotriene B4 receptor 1	LTB4R	Q15722
10	C-C chemokine receptor type 2	CCR2	P41597
11	Telomerase reverse transcriptase	TERT	O14746
12	Protein kinase C theta	PRKCQ	Q04759
13	Leukotriene A4 hydrolase	LTA4H	
14	Prostanoid EP4 receptor	PTGER4	P35408
15	Amine oxidase, copper containing	AOC3	Q16853
16	Estrogen receptor beta	ESR2	Q92731
17	Corticosteroid binding globulin	SERPINA6	P08185
18	Serotonin 1a (5-HT1a) receptor	HTR1A	P08908
19	Adenosine A3 receptor	ADORA3	P0DMS8
20	Phospholipase A2 group 1B	PLA2G1B	P04054
21	Sphingosine 1-phosphate receptor Edg-1	S1PR1	P21453
22	Stem cell growth factor receptor	KIT	P10721
23	Cathepsin S	CTSS	P25774
24	Phosphodiesterase 4D	PDE4D	Q08499
25	Rho-associated protein kinase	ROCK1	Q13464
26	Cathepsin K	CTSK	P43235
27	Rho-associated protein kinase 2	ROCK2	O75116
28	Serine/threonine-protein kinase PIM2	PIM2	Q9P1W9
29	Cyclin-dependent kinase 1/cyclin B	CDK1	P06493
30	Thymidine kinase, cytosolic	TK1	P04183

**Table 5:** Molecular docking results of active components from *L. reticulata* and potential targets of inflammation.

Gene	Phytocompounds	Binding Affinity kcal/mol	Interactions
CCR2	Lycocernuine	−8	
	(1S,4R)-10-Hydroxyfenchone glucoside	−7.8	TRP A:98, SER A:101
	Kaempferol	−7.7	THR A:179, SER A:101
ICAM1	1-Pyrenylsulfate	−5.9	LYS A:128, GLN A:156, HIS A:153
	Kaempferol	−5.5	GLN A:156, HIS A:152
	(1S,4R)-10-Hydroxyfenchone glucoside	−5.2	GLN A:156, HIS A:153, LYS A:128, GLY A:154, HIS A:152
KIT	Kaempferol	−9.9	CYSA:673
	1-Pyrenylsulfate	−9.4	LYS A:623
	1,8-Heptadecadiene-4,6-diyne-3,10-diol	−7.4	
MPO	Kaempferol	−8.5	ARG C:323, ARG D:161
	1-Pyrenylsulfate	−8.3	ARG C:323
	Lycocernuine	−8	ARG C:323
NOS2	1-Pyrenylsulfate	−10.3	GLY D:371, GLU D:377
	Kaempferol	−9.5	TRP D:372
	Lycocernuine	−8.3	
STAT3	1-Pyrenylsulfate	−7	THR A:456, LYS A:318, LYS A:244
	Kaempferol	−6.7	PHE A:321, THR A:456, LYS A:318
	(1S,4R)-10-Hydroxyfenchone glucoside	−6.6	SER A:319, PHE A:321, GLU A:455, THR A:456, LYS A:244

**Table 6:** List of the phytocompounds acquired for molecular docking.

S. No	Compound Name	PubChem ID	Molecular Weight (g/mol)	Molecular Formula
1	(1S,4R)-10-Hydroxyfenchone glucoside	85257992	330.37	C_16_ H_26_ O_7_
2	(Z)-3-(1-Formyl-1-propenyl)pentanedioic acid	22394751	200.19	C_9_ H_12_ O_5_
3	1,8-Heptadecadiene-4,6-diyne-3,10-diol	5318010	260.399	C_17_ H_24_ O_2_
4	1-Pyrenylsulfate	9543290	298.3	C_16_ H_10_ O_4_ S
5	2,3,5,7,9-Pentathiadecane 2,2-dioxide	11777600	264.5	C_5_ H_12_ O_2_ S_2_
6	8Z-11Z-14Z-Heptadecatrienoic acid	16061034	264.4	C_17_ H_28_ O_2_
7	Albuterol	2083	239.31	C_13_ H_21_ N O_3_
8	C16 Sphinganine	656816	273.45	C_16_ H_35_ N O_2_
9	Isocarbostyril	10284	145.16	C_9_ H_7_ N O
10	Kaempferol	5280863	286.24	C_15_ H_10_ O_6_
11	Lycocernuine	442481	278.39	C_16_ H_26_ N_2_ O_2_
12	Malic acid	525	134.09	C_4_ H_6_ O_5_
13	Methyl(3×,10R)-dihydroxy-11-dodecene-6,8-diynoate 10-glucoside	131752977	398.4	C_19_ H_26_ O_9_
14	Metprolol	4171	267.36	C_15_ H_25_ N O_3_
15	Monomenthyl succinate	10199004	256.339	C_14_ H_24_ O_4_
16	Neotussilagine	4484216	199.25	C_10_ H_7_ N O_3_
17	Hexyl 2-furoate	61984	196.24	C_11_ H_16_ O_3_
18	beta-D-3[5-Deoxy-5-(dimethylarsinyl)ribofuranosyloxy]-2-hydroxy-1-propanesulfonic acid	131751282	392.26	C_10_ H_21_ As O_9_ S

## Data Availability

Data is contained within the article and Supplementary Materials.

## Supplementary Materials

The following supporting information can be downloaded at: https://www.mdpi.com/article/10.3390/ph17040423/s1, Figure S1: (A) Interaction of CCR2 and ligand; (B) Interaction of ICAM-1 and ligand; (C) Interaction of KIT and ligand; (D) Interaction of MPO and ligand; (E) Interaction of NOS2 and ligand; (F) Interaction of STAT3 and ligand. Figure S2: (A) Interaction residue of (1S,4R)-10-Hydroxyfenchone glucoside with CCR2; (B) Interaction residue of Kaempferol with CCR2; (C) Interaction residue of Lycocernuine with CCR2. Figure S3: (A) Interaction residue of (1S,4R)-10-Hydroxyfenchone glucoside with ICAM-1; (B) Interaction residue of 1-Pyrenylsulfate with ICAM-1; (C) Interaction residue of Kaempferol with ICAM-1. Figure S4: (A) Interaction residue of 1,8-Heptadecadiene-4,6-diyne-3,10-diol with KIT; (B) Interaction residue of 1-Pyrenylsulfate with KIT; (C) Interaction residue of Kaempferol with KIT. Figure S5: (A) Interaction residue of 1-Pyrenylsulfate with MPO; (B) Interaction residue of Kaempferol with MPO; (C) Interaction residue of Lycocernuine with MPO. Figure S6: (A) Interaction residue of 1-Pyrenylsulfate with NOS2; (B) Interaction residue of Kaempferol with NOS2; (C) Interaction residue of Lycocernuine with NOS2. Figure S7: (A) Interaction residue of (1S,4R)-10-Hydroxyfenchone with STAT3; (B) Interaction residue of glucoside1-Pyrenylsulfate with STAT3; (C) Interaction residue of Kaempferol with STAT3. Figure S8: (A) Interactions of (1S,4R)-10-Hydroxyfenchone glucoside with ICAM1; (B) Interactions of (1S,4R)-10-Hydroxyfenchone glucoside with CCR2. Figure S9: (A) ICAM1 Protein RMSF; (B) CCR2 Protein RMSF. Figure S10: (A) The number of ligand contacts with ICAM1 protein and the amino acid sites; (B) The number of ligand contacts with CCR2 protein and the amino acid sites.
