# Supplementary material for: Metabolomic Profiling of Leptadenia reticulata: Unveiling Therapeutic Potential for Inflammatory Diseases through Network Pharmacology and Docking Studies
Source: Pharmaceuticals (Basel). 2024 Mar 26;17(4):423. doi: 10.3390/ph17040423 (PMC11054655; doi:10.3390/ph17040423)

**Sample Name**	L	**Position**	P1-A3	**Instrument Name**	QTOF	**User Name**	
**Inj Vol**	3	**InjPosition**		**SampleType**	Sample	**IRM Calibration Status**	Success
**Data Filename**	L.d	**ACQ Method**	metabolite_ESI_+VE_M	**Comment**		**Acquired Time**	5/4/2023 10:15:15 AM

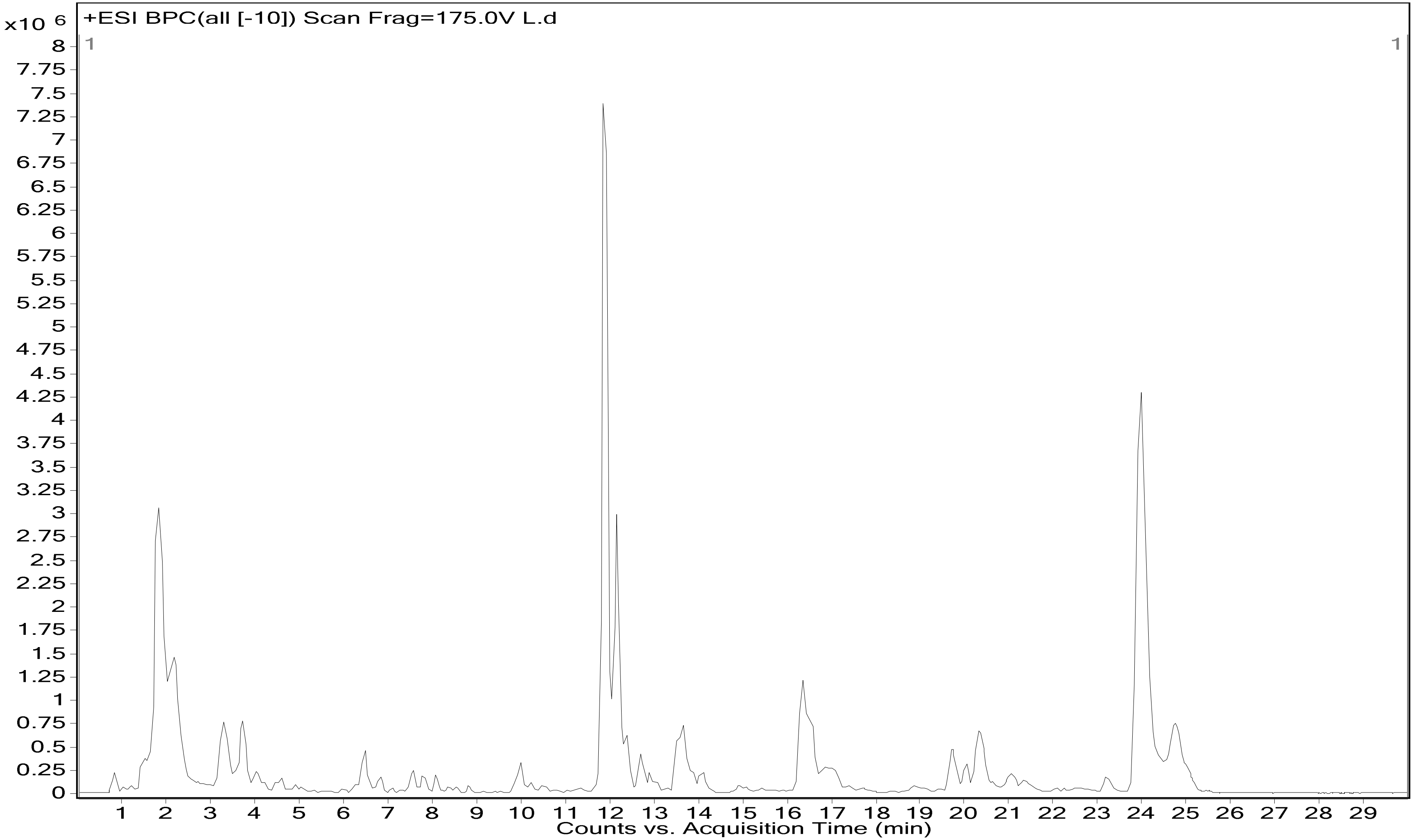

**Sample Name**	M	**Position**	P1-A6	**Instrument Name**	QTOF	**User Name**	
**Inj Vol**	3	**InjPosition**		**SampleType**	Sample	**IRM Calibration Status**	Success
**Data Filename**	M.d	**ACQ Method**	metabolite_ESI_+VE_M	**Comment**		**Acquired Time**	5/7/2023 7:10:20 PM

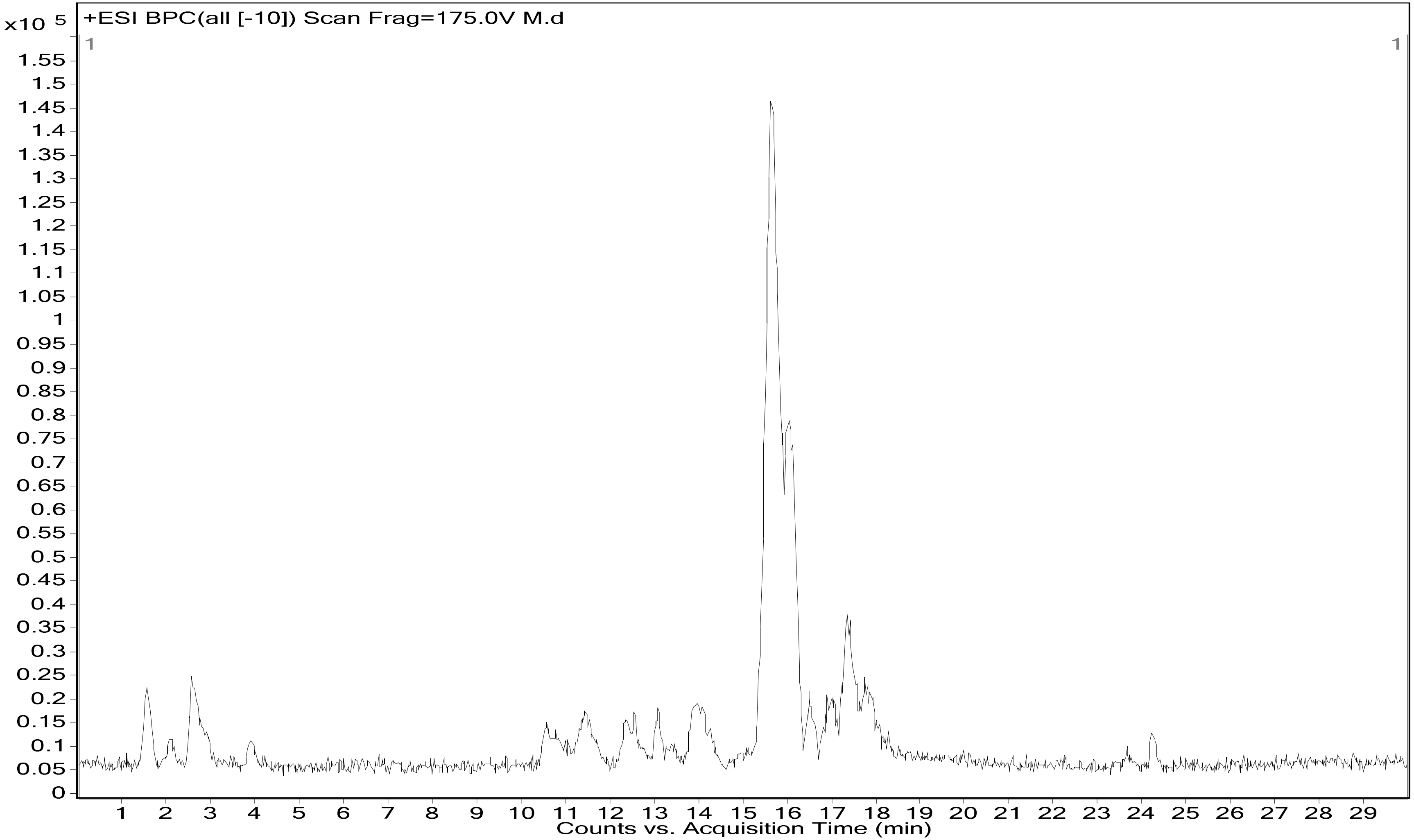

**Sample Name**	R	**Position**	P1-A4	**Instrument Name**	QTOF	**User Name**	
**Inj Vol**	3	**InjPosition**		**SampleType**	Sample	**IRM Calibration Status**	Success
**Data Filename**	R.d	**ACQ Method**	metabolite_ESI_+VE_M	**Comment**		**Acquired Time**	5/4/2023 11:26:43 AM

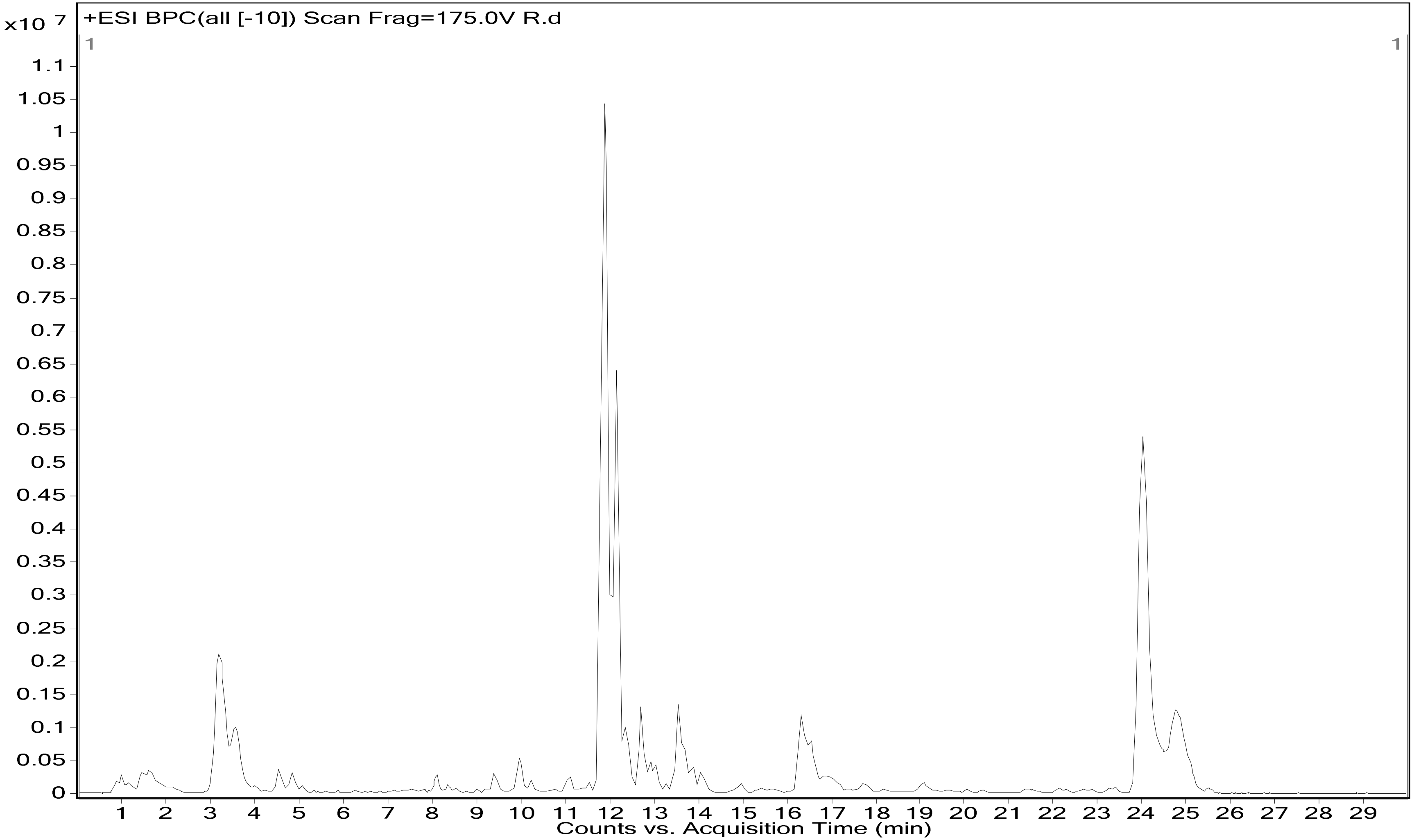

**Sample Name**	S	**Position**	P1-A5	**Instrument Name**	QTOF	**User Name**	
**Inj Vol**	3	**InjPosition**		**SampleType**	Sample	**IRM Calibration Status**	Success
**Data Filename**	S-r.d	**ACQ Method**	metabolite_ESI_+VE_M	**Comment**		**Acquired Time**	5/7/2023 5:58:52 PM

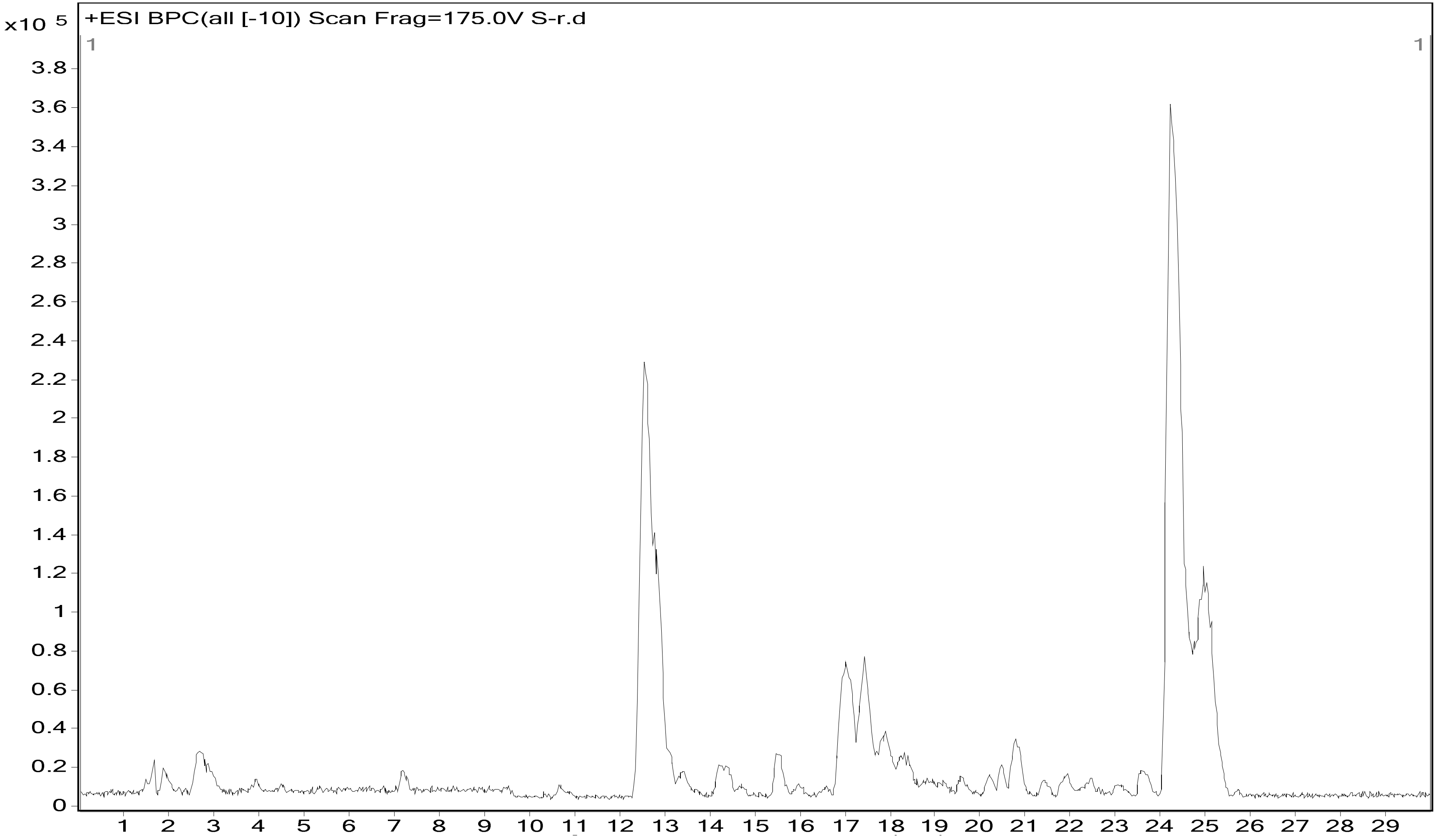

Supplement: Supplementary file 1 [file pharmaceuticals-17-00423-s001.zip › HR LCMS chromatogram/chromatogram_+ve.pdf]
