# Supplementary material for: Metabolomic Profiling of Leptadenia reticulata: Unveiling Therapeutic Potential for Inflammatory Diseases through Network Pharmacology and Docking Studies
Source: Pharmaceuticals (Basel). 2024 Mar 26;17(4):423. doi: 10.3390/ph17040423 (PMC11054655; doi:10.3390/ph17040423)

**Sample Name**	L	**Position**	P1-A3	**Instrument Name**	QTOF	**User Name**	
**Inj Vol**	3	**InjPosition**		**SampleType**	Sample	**IRM Calibration Status**	Success
**Data Filename**	L_-VE.d	**ACQ Method**	metabolite_ESI_-VE_M	**Comment**		**Acquired Time**	5/8/2023 1:08:19 AM

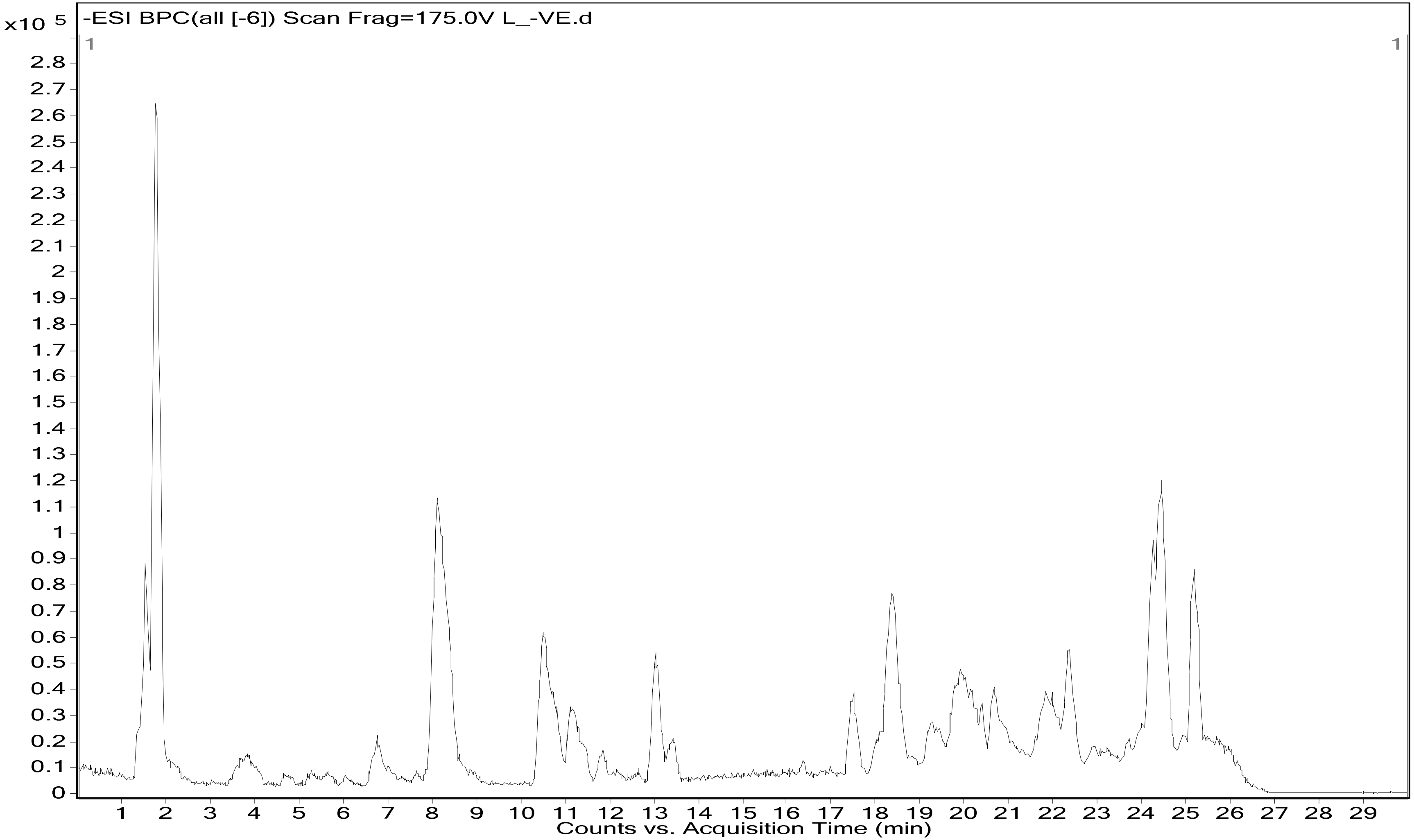

**Sample Name**	M	**Position**	P1-A6	**Instrument Name**	QTOF	**User Name**	
**Inj Vol**	3	**InjPosition**		**SampleType**	Sample	**IRM Calibration Status**	Success
**Data Filename**	M_-VE.d	**ACQ Method**	metabolite_ESI_-VE_M	**Comment**		**Acquired Time**	5/8/2023 4:42:44 AM

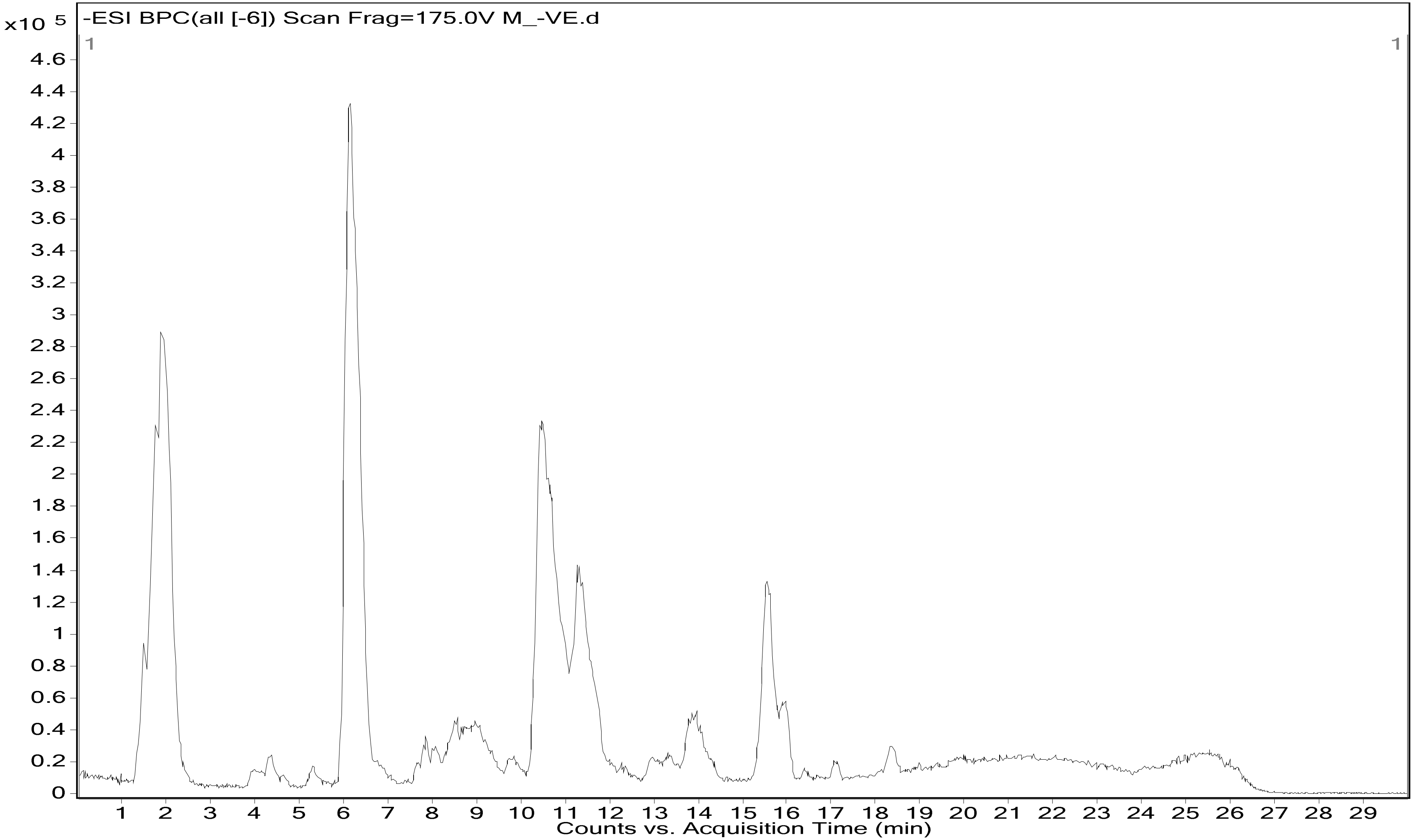

**Sample Name**	R	**Position**	P1-A4	**Instrument Name**	QTOF	**User Name**	
**Inj Vol**	3	**InjPosition**		**SampleType**	Sample	**IRM Calibration Status**	Success
**Data Filename**	R_-VE.d	**ACQ Method**	metabolite_ESI_-VE_M	**Comment**		**Acquired Time**	5/8/2023 2:19:48 AM

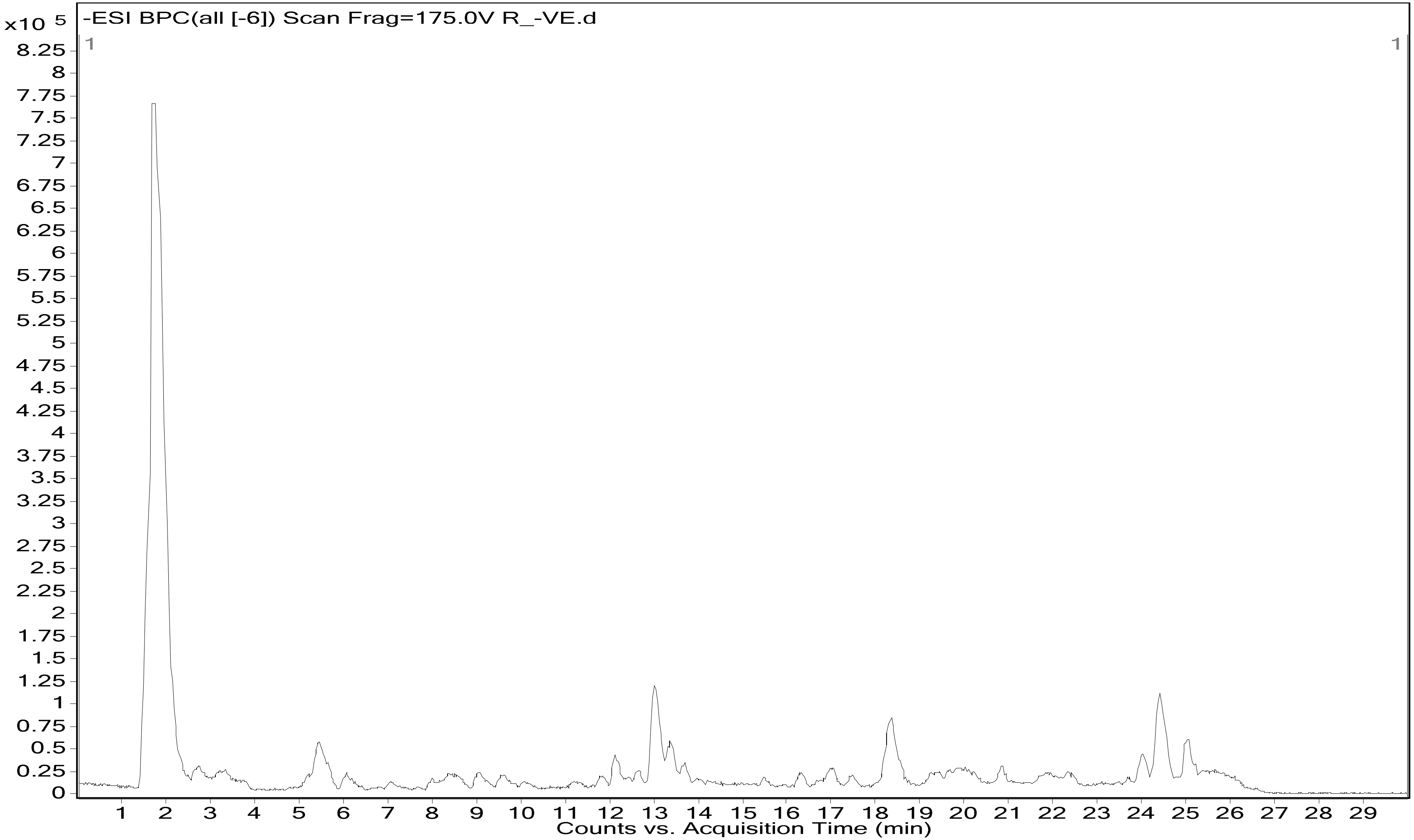

**Sample Name**	S	**Position**	P1-A5	**Instrument Name**	QTOF	**User Name**	
**Inj Vol**	3	**InjPosition**		**SampleType**	Sample	**IRM Calibration Status**	Success
**Data Filename**	S_-VE.d	**ACQ Method**	metabolite_ESI_-VE_M	**Comment**		**Acquired Time**	5/8/2023 3:31:17 AM

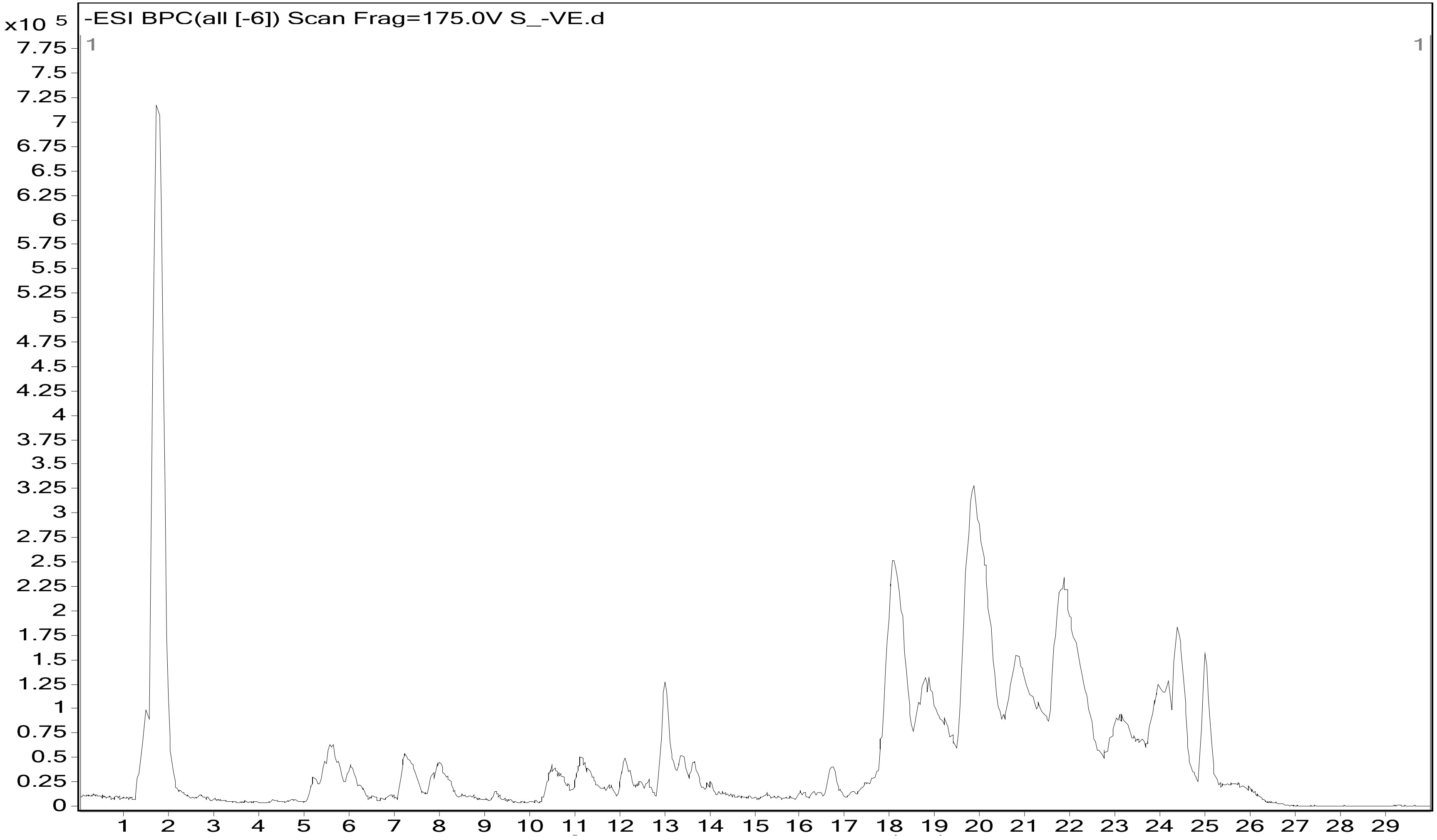

Supplement: Supplementary file 1 [file pharmaceuticals-17-00423-s001.zip › HR LCMS chromatogram/chromatogram_-ve.pdf]
