# Supplementary material for: Metabolomic Profiling of Leptadenia reticulata: Unveiling Therapeutic Potential for Inflammatory Diseases through Network Pharmacology and Docking Studies
Source: Pharmaceuticals (Basel). 2024 Mar 26;17(4):423. doi: 10.3390/ph17040423 (PMC11054655; doi:10.3390/ph17040423)
Supplement: Supplementary file 1 [file pharmaceuticals-17-00423-s001.zip › HR LCMS chromatogram/L_-VE_CompoundReport.pdf]

Qualitative Compound Report

**Data File**	L_-VE.d	**Sample Name**	L
**Sample Type**	Sample	**Position**	P1-A3
**Instrument Name**	QTOF	**User Name**	
**Acq Method**	metabolite_ESI_-VE_MSMS.m	**Acquired Time**	5/8/2023 1:08:19 AM
**IRM Calibration Status**	Success	**DA Method**	default.m
**Comment**			

**Sample Group**		**Info.**
**Acquisition SW Version**	6200 series TOF/6500 series Q-TOF B.05.01 (B5125.3)	

Compound Table

Compound Label	RT	Mass	Abund	Name	Formula	Tgt Mass	Diff (ppm)	MFG Formula	DB Formula	DB Diff (ppm)	Hits (DB)
Cpd 1: Caffeic aldehyde; C9 H8 O3	6.679	164.0487	21783	Caffeic aldehyde	C9 H8 O3			C9 H8 O3	C9 H8 O3	-8.51	
Cpd 2: Quercitrin; C21 H20 O11	7.651	448.1036	4536	Quercitrin	C21 H20 O11			C21 H20 O11	C21 H20 O11	-6.75	
Cpd 3: Quercitrin; C21 H20 O11	7.651	448.1036	4536	Quercitrin	C21 H20 O11			C21 H20 O11	C21 H20 O11	-6.75	
Cpd 4: Kaempferol; C15 H10 O6	8.245	286.0493	1136	Kaempferol	C15 H10 O6			C15 H10 O6	C15 H10 O6	-5.42	
Cpd 5: Luteolin; C15 H10 O6	8.245	286.0493	1136	Luteolin	C15 H10 O6			C15 H10 O6	C15 H10 O6	-5.42	
Cpd 6: Colnelenic acid; C18 H28 O3	16.007	292.206	1013	Colnelenic acid	C18 H28 O3			C18 H28 O3	C18 H28 O3	-7.46	
Cpd 7: 9-HOTE; C18 H30 O3	16.911	294.2213	1198	9-HOTE	C18 H30 O3			C18 H30 O3	C18 H30 O3	-6.07	
Cpd 8: Caffeic aldehyde; C9 H8 O3	6.679	164.0487	21783	Caffeic aldehyde	C9 H8 O3			C9 H8 O3	C9 H8 O3	-8.51	
Cpd 9: Ferulic acid; C10 H10 O4	7.055	194.0593	1647	Ferulic acid	C10 H10 O4			C10 H10 O4	C10 H10 O4	-7.07	
Cpd 10: Ferulic acid; C10 H10 O4	7.055	194.0593	1647	Ferulic acid	C10 H10 O4			C10 H10 O4	C10 H10 O4	-7.07	
Cpd 11: C9 H6 O2	8.782	146.0373	782		C9 H6 O2	146.0368	3.48	C9 H6 O2	C9 H6 O2		
Cpd 12: Ellagic acid; C14 H6 O8	7.584	302.0087	2557	Ellagic acid	C14 H6 O8			C14 H6 O8	C14 H6 O8	-7.96	
Cpd 13: C14 H12 O3	2.084	228.078	750		C14 H12 O3	228.0786	-2.73	C14 H12 O3	C14 H12 O3		
Cpd 14: Caffeic aldehyde; C9 H8 O3	6.679	164.0487	21783	Caffeic aldehyde	C9 H8 O3			C9 H8 O3	C9 H8 O3	-8.51	
Cpd 15: Malic acid; C4 H6 O5	6.775	134.0227	10108	Malic acid	C4 H6 O5			C4 H6 O5	C4 H6 O5	-8.67	
Cpd 16: C6 H8 O7	2.134	192.0272	940		C6 H8 O7	192.027	0.91	C6 H8 O7	C6 H8 O7		
Cpd 17: Ribose-1-arsenate; C5 H11 As O8	1.505	273.9661	46846	Ribose-1-arsenate	C5 H11 As O8			C5 H11 As O8	C5 H11 As O8	3.14	2
Compound 18	1.523										
Cpd 19: 2,3,5,7,9-Pentathiadecane 2,2-dioxide; C5 H12 O2 S5	1.544	263.9397	19559	2,3,5,7,9-Pentathiadecane 2,2-dioxide	C5 H12 O2 S5			C5 H12 O2 S5	C5 H12 O2 S5	16.53	1
Cpd 20: DuP-697; C17 H12 Br F O2 S2	1.582	409.9425	11063	DuP-697	C17 H12 Br F O2 S2			C17 H12 Br F O2 S2	C17 H12 Br F O2 S2	5.1	1
Cpd 21: Apigenin 7-[rhamnosyl-(1->2)-galacturonide]; C27 H28 O15	8.028	592.1465		Apigenin 7-[rhamnosyl-(1->2)-galacturonide]	C27 H28 O15			C27 H28 O15	C27 H28 O15	-6.22	10
Cpd 22: CMP-N-glycoloylneuraminate; C20 H31 N4 O17 P	8.096	630.1376		CMP-N-glycoloylneuraminate	C20 H31 N4 O17 P			C20 H31 N4 O17 P	C20 H31 N4 O17 P	7.26	1
Cpd 23: Nicotiflorin; C27 H30 O15	8.277	594.1631	88374	Nicotiflorin	C27 H30 O15			C27 H30 O15	C27 H30 O15	-7.77	10
Cpd 24: Apigenin 7-[rhamnosyl-(1->2)-galacturonide]; C27 H28 O15	8.321	592.1464		Apigenin 7-[rhamnosyl-(1->2)-galacturonide]	C27 H28 O15			C27 H28 O15	C27 H28 O15	-6.08	10
Cpd 25: Genistein 8-C-glucoside; C21 H20 O10	8.378	432.1092		Genistein 8-C-glucoside	C21 H20 O10			C21 H20 O10	C21 H20 O10	-8.33	10
Cpd 26: CMP-N-glycoloylneuraminate; C20 H31 N4 O17 P	8.461	630.1377		CMP-N-glycoloylneuraminate	C20 H31 N4 O17 P			C20 H31 N4 O17 P	C20 H31 N4 O17 P	7.05	1
Cpd 27: Biorobin; C27 H30 O15	8.543	594.1627	26423	Biorobin	C27 H30 O15			C27 H30 O15	C27 H30 O15	-7.1	10
Cpd 28: Glafenine; C19 H17 Cl N2 O4	8.66	372.0889		Glafenine	C19 H17 Cl N2 O4			C19 H17 Cl N2 O4	C19 H17 Cl N2 O4	-3.36	10
Cpd 29: Tetradecyl sulfate; C14 H30 O4 S	13.01	294.1858		Tetradecyl sulfate	C14 H30 O4 S			C14 H30 O4 S	C14 H30 O4 S	2.39	10
Cpd 30: Hexazinone; C12 H20 N4 O2	18.039	252.1576	17400	Hexazinone	C12 H20 N4 O2			C12 H20 N4 O2	C12 H20 N4 O2	4.03	4
Compound 31	19.193										
Compound 32	19.49										
Cpd 33: Hexazinone; C12 H20 N4 O2	19.915	252.1576	42076	Hexazinone	C12 H20 N4 O2			C12 H20 N4 O2	C12 H20 N4 O2	4.27	6
Compound 34	20.072										
Cpd 35: Magnesium protoporphyrin monomethyl ester; C35 H34 Mg N4 O4	20.121	598.2395	11004	Magnesium protoporphyrin monomethyl ester	C35 H34 Mg N4 O4			C35 H34 Mg N4 O4	C35 H34 Mg N4 O4	6	2
Cpd 36: Lamprolobine; C15 H24 N2 O2	20.351	264.1787		Lamprolobine	C15 H24 N2 O2			C15 H24 N2 O2	C15 H24 N2 O2	19.05	8
Cpd 37: Kanokoside D; C27 H44 O16	20.386	624.2638		Kanokoside D	C27 H44 O16			C27 H44 O16	C27 H44 O16	-1.43	4
Cpd 38: 19-Hydroxycinnzeylanol 19-glucoside; C26 H42 O13	20.645	562.2633		19-Hydroxycinnzeylanol 19-glucoside	C26 H42 O13			C26 H42 O13	C26 H42 O13	-1.3	4
Cpd 39: Magnesium protoporphyrin monomethyl ester; C35 H34 Mg N4 O4	20.74	598.2393	14210	Magnesium protoporphyrin monomethyl ester	C35 H34 Mg N4 O4			C35 H34 Mg N4 O4	C35 H34 Mg N4 O4	6.34	2
Cpd 40: Hexazinone; C12 H20 N4 O2	21.05	252.1575	22450	Hexazinone	C12 H20 N4 O2			C12 H20 N4 O2	C12 H20 N4 O2	4.57	6
Compound 41	22.051										
Cpd 42: Muricatalin; C35 H64 O8	22.572	612.4638	5517	Muricatalin	C35 H64 O8			C35 H64 O8	C35 H64 O8	-6.07	10
Cpd 43: 14,19-Dihydroaspidospermatine; C21 H28 N2 O2	23.787	340.2102		14,19-Dihydroaspidospermatine	C21 H28 N2 O2			C21 H28 N2 O2	C21 H28 N2 O2	14.31	10

Cpd 44: 14,19-Dihydroaspidospermatine; C21 H28 N2 O2	24.069	340.2105	25299	14,19-Dihydroaspidospermatine	C21 H28 N2 O2			C21 H28 N2 O2	C21 H28 N2 O2	13.35	6
Cpd 45: Lycocernuine; C16 H26 N2 O2	24.227	278.1948		Lycocernuine	C16 H26 N2 O2			C16 H26 N2 O2	C16 H26 N2 O2	16.71	6
Compound 46	24.236		11503								

Compound Label	Name	*m/z*	RT	Algorithm	Mass
Cpd 1: Caffeic aldehyde; C9 H8 O3	**Caffeic aldehyde**	163.0415	6.679	Find By Formula	164.0487

MS Spectrum

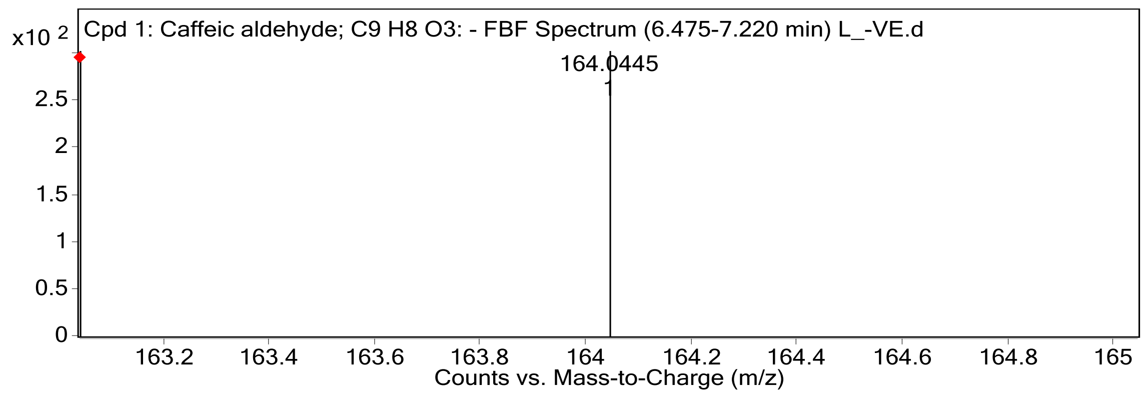

MS Zoomed Spectrum

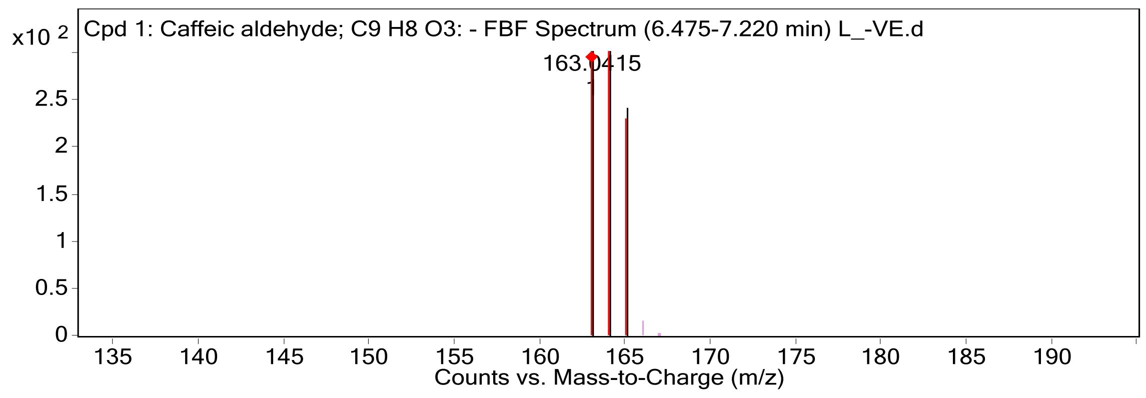

MS Spectrum Peak List

m/z	z	Abund	Formula	Ion
163.0415	1	21782.78	C9 H8 O3	(M-H)-
164.0445	1	2080.31	C9 H8 O3	(M-H)-
165.0468	1	242.24	C9 H8 O3	(M-H)-

MSMS Spectrum

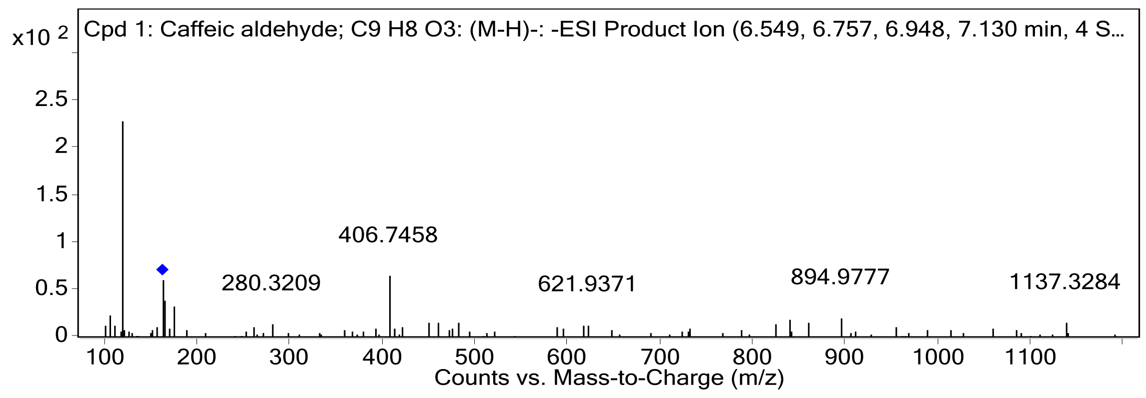

MS/MS Spectrum Peak List

m/z	z	Abund
106.0405		22.68
119.0496	1	228.56
163.0342	2	60.21
164.8359		39.52
174.5847		33.31
406.7458		65.13
449.7887		15.81
458.7679		16.16
838.3779		18
894.9777		20.56

Compound Structure

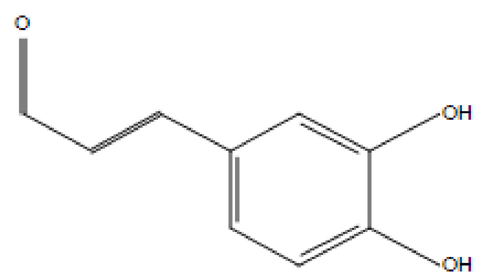

Compound Label	Name	*m/z*	RT	Algorithm	Mass
Cpd 2: Quercitrin; C21 H20 O11	**Quercitrin**	447.0962	7.651	Find By Formula	448.1036

MS Spectrum

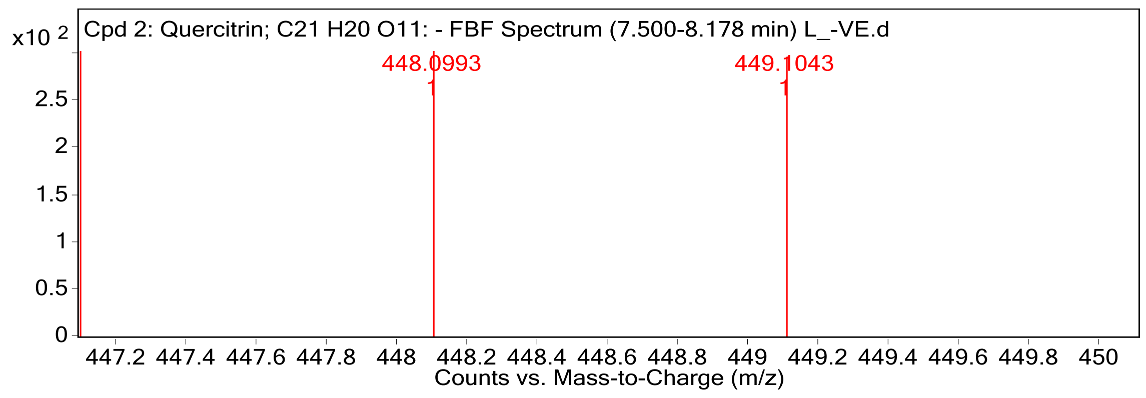

MS Zoomed Spectrum

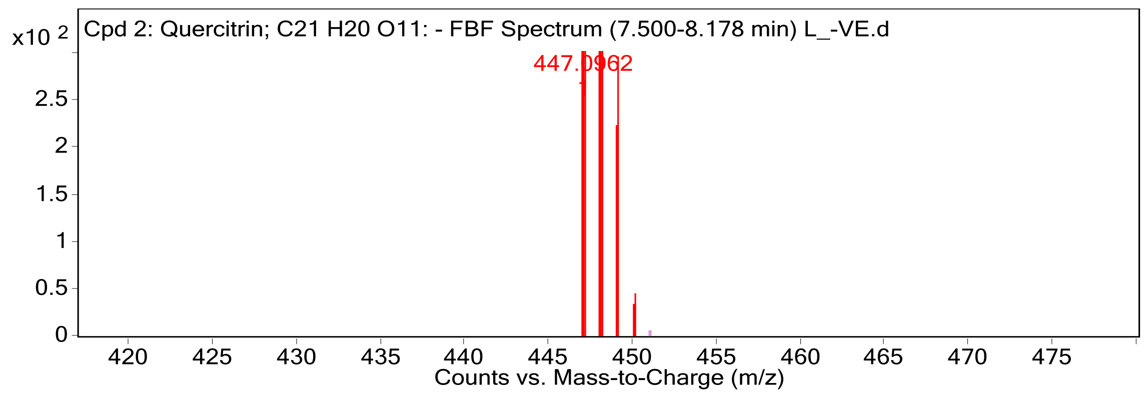

MS Spectrum Peak List

m/z	z	Abund	Formula	Ion
447.0962	1	4536.32	C21 H20 O11	(M-H)-
448.0993	1	1019.91	C21 H20 O11	(M-H)-
449.1043	1	296.85	C21 H20 O11	(M-H)-
450.1109	1	46.23	C21 H20 O11	(M-H)-

Compound Structure

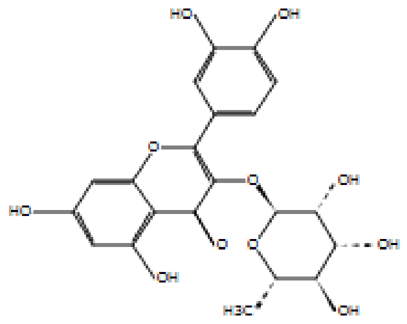

Compound Label	Name	*m/z*	RT	Algorithm	Mass
Cpd 3: Quercitrin; C21 H20 O11	**Quercitrin**	447.0962	7.651	Find By Formula	448.1036

MS Spectrum

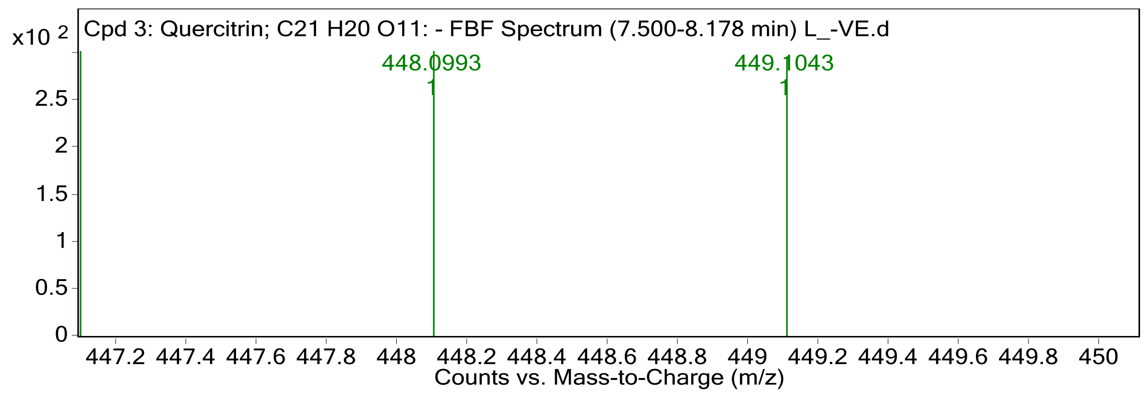

MS Zoomed Spectrum

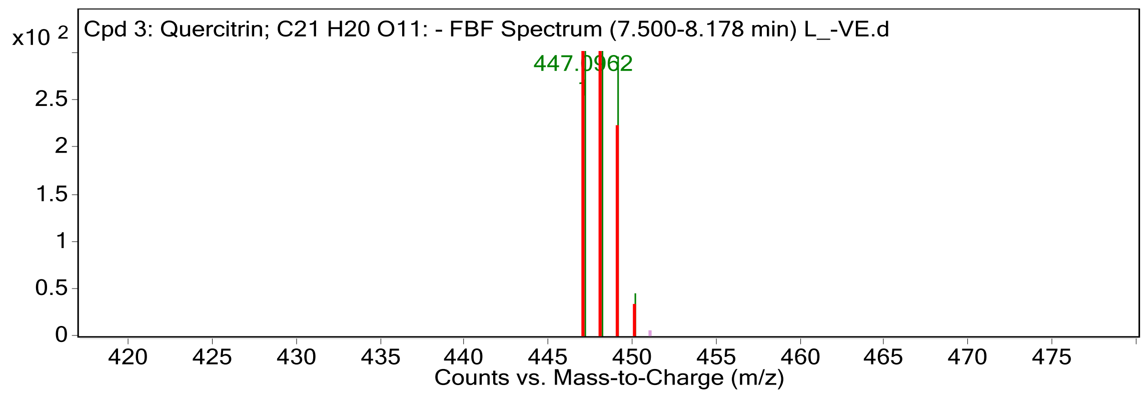

MS Spectrum Peak List

m/z	z	Abund	Formula	Ion
447.0962	1	4536.32	C21 H20 O11	(M-H)-
448.0993	1	1019.91	C21 H20 O11	(M-H)-
449.1043	1	296.85	C21 H20 O11	(M-H)-
450.1109	1	46.23	C21 H20 O11	(M-H)-

Compound Structure

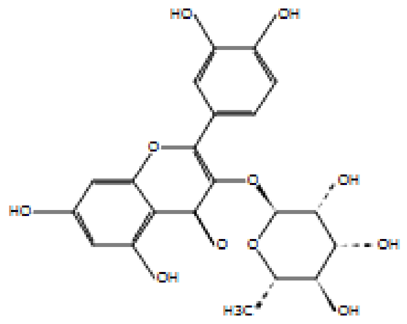

Compound Label	Name	*m/z*	RT	Algorithm	Mass
Cpd 4: Kaempferol; C15 H10 O6	**Kaempferol**	285.0422	8.245	Find By Formula	286.0493

MS Spectrum

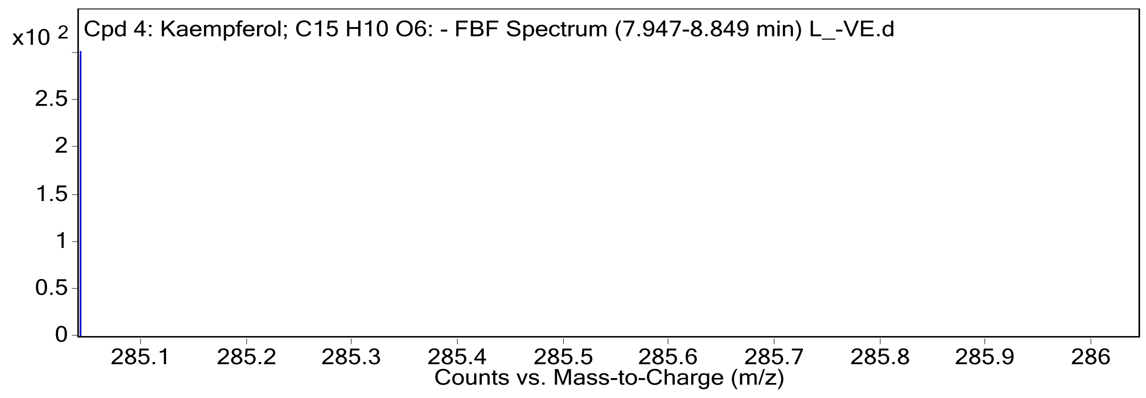

MS Zoomed Spectrum

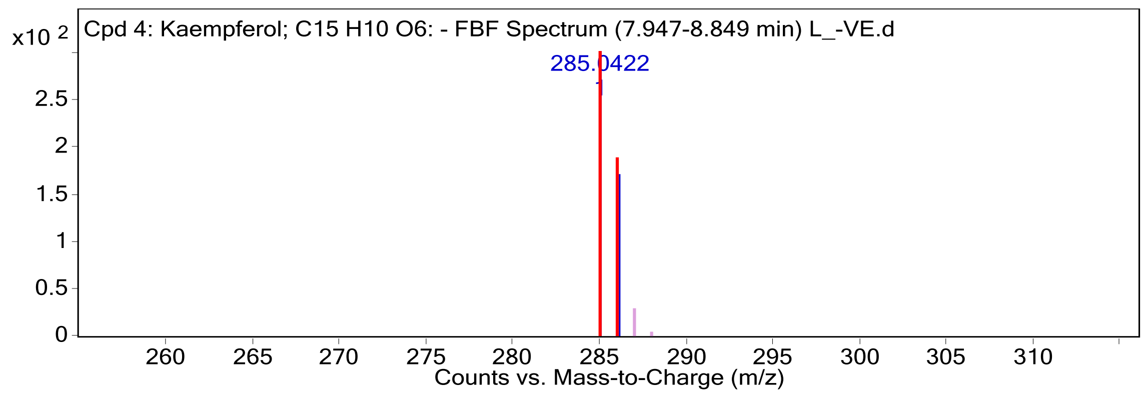

MS Spectrum Peak List

m/z	z	Abund	Formula	Ion
285.0422	1	1136.44	C15 H10 O6	(M-H)-
286.0443	1	172.42	C15 H10 O6	(M-H)-

Compound Structure

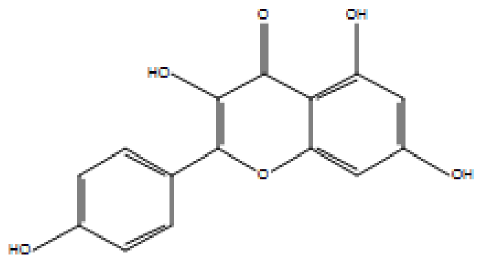

Compound Label	Name	*m/z*	RT	Algorithm	Mass
Cpd 5: Luteolin; C15 H10 O6	**Luteolin**	285.0422	8.245	Find By Formula	286.0493

MS Spectrum

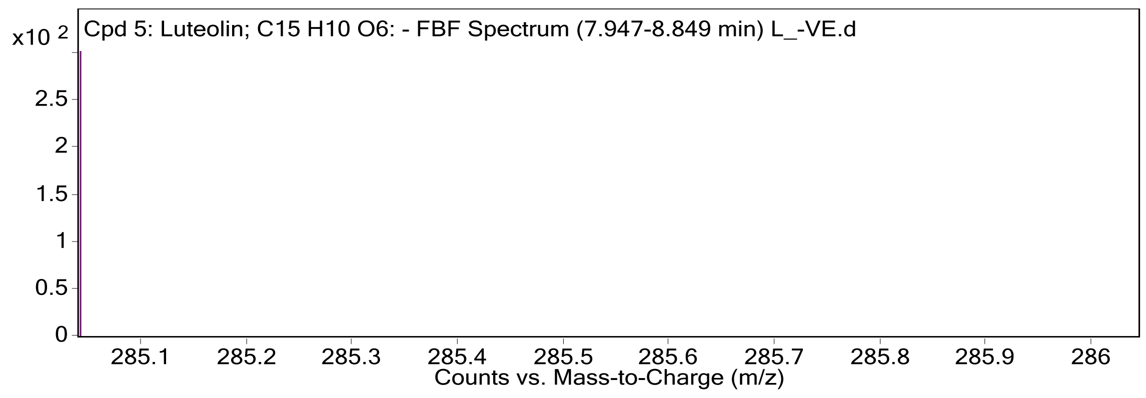

MS Zoomed Spectrum

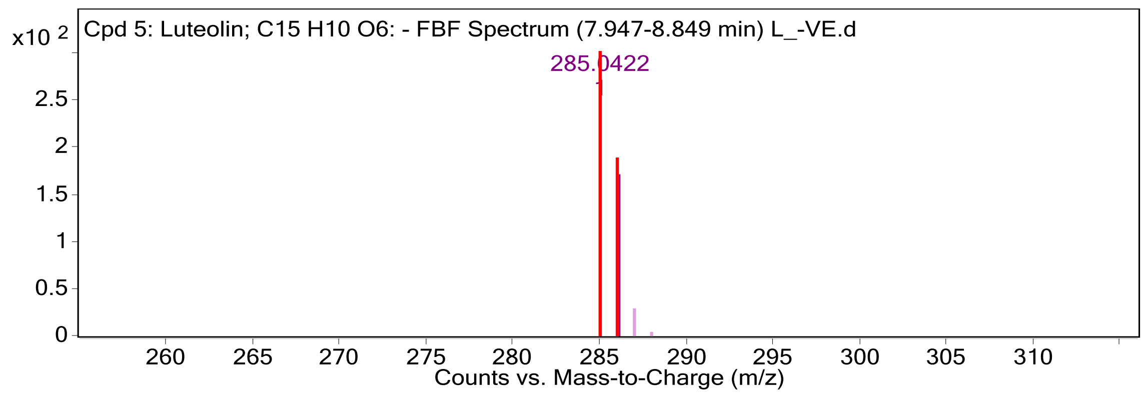

MS Spectrum Peak List

m/z	z	Abund	Formula	Ion
285.0422	1	1136.44	C15 H10 O6	(M-H)-
286.0443	1	172.42	C15 H10 O6	(M-H)-

Compound Structure

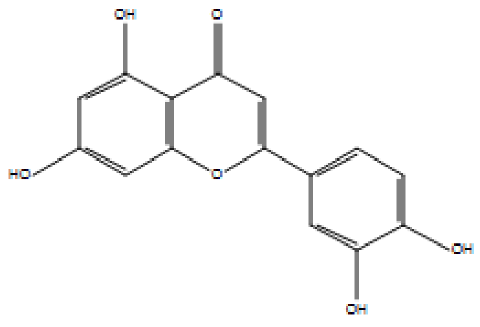

Compound Label	Name	*m/z*	RT	Algorithm	Mass
Cpd 6: Colnelenic acid; C18 H28 O3	**Colnelenic acid**	291.1987	16.007	Find By Formula	292.206

MS Spectrum

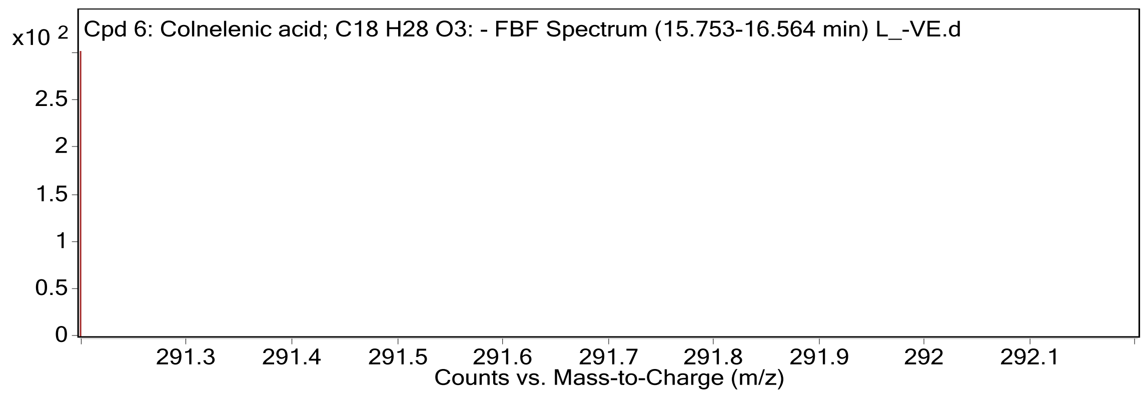

MS Zoomed Spectrum

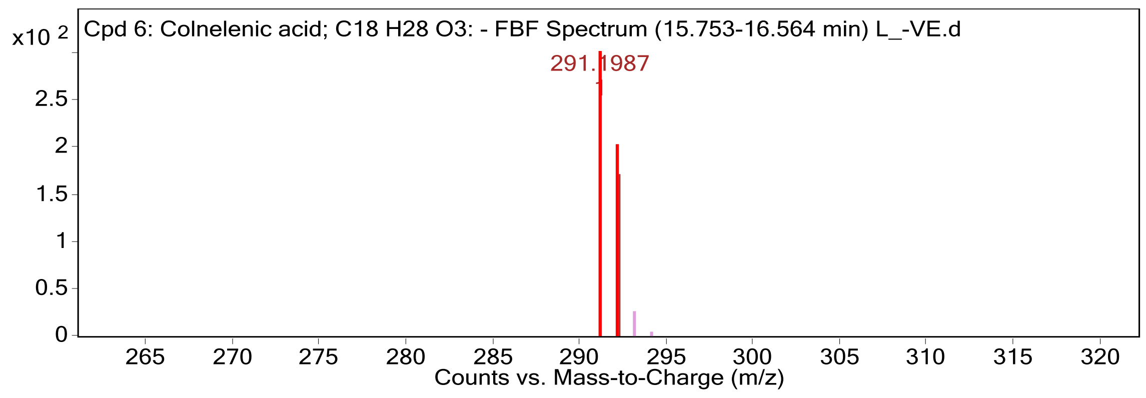

MS Spectrum Peak List

m/z	z	Abund	Formula	Ion
291.1987	1	1012.92	C18 H28 O3	(M-H)-
292.2022	1	172.5	C18 H28 O3	(M-H)-

Compound Structure

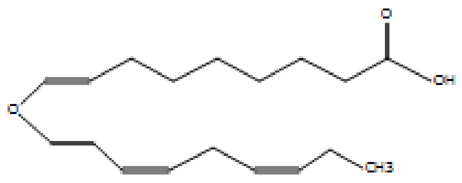

Compound Label	Name	*m/z*	RT	Algorithm	Mass
Cpd 7: 9-HOTE; C18 H30 O3	**9-HOTE**	293.2141	16.911	Find By Formula	294.2213

MS Spectrum

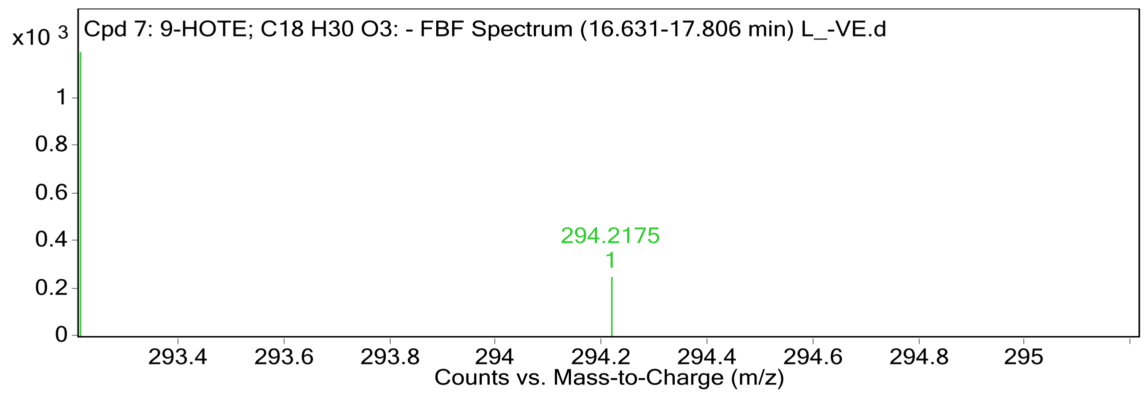

MS Zoomed Spectrum

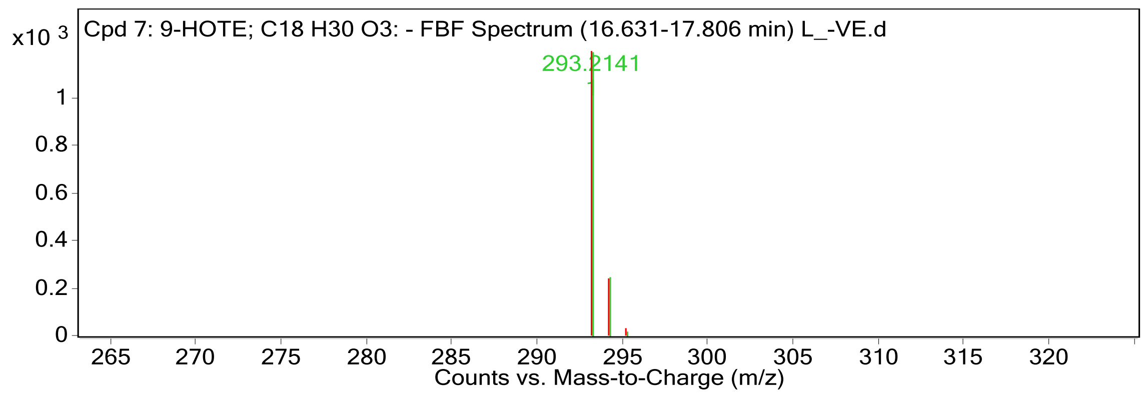

MS Spectrum Peak List

m/z	z	Abund	Formula	Ion
293.2141	1	1198.27	C18 H30 O3	(M-H)-
294.2175	1	254.4	C18 H30 O3	(M-H)-
295.2142	1	27.41	C18 H30 O3	(M-H)-

Compound Structure

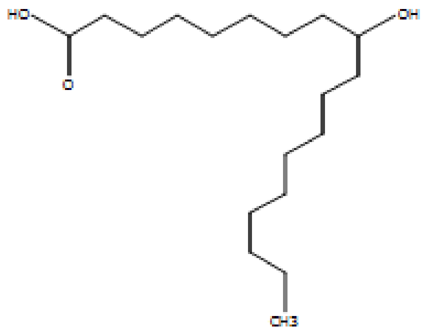

Compound Label	Name	*m/z*	RT	Algorithm	Mass
Cpd 8: Caffeic aldehyde; C9 H8 O3	**Caffeic aldehyde**	163.0415	6.679	Find By Formula	164.0487

MS Spectrum

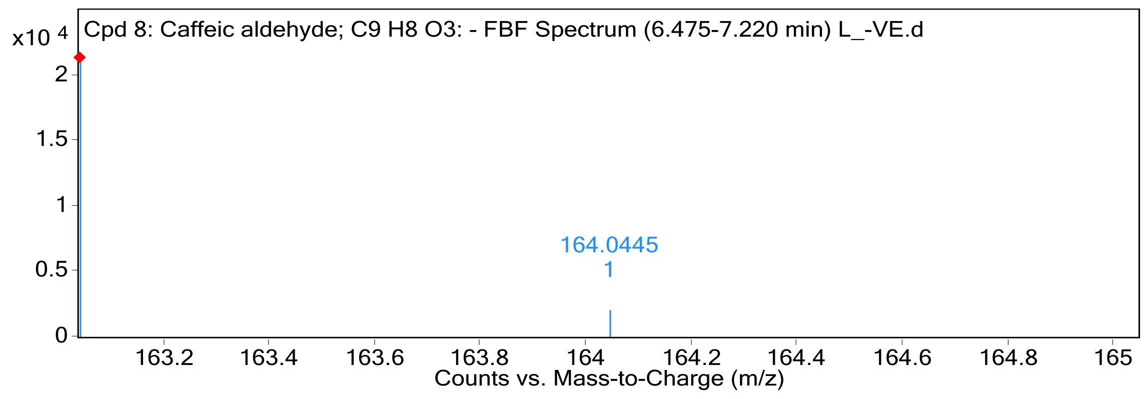

MS Zoomed Spectrum

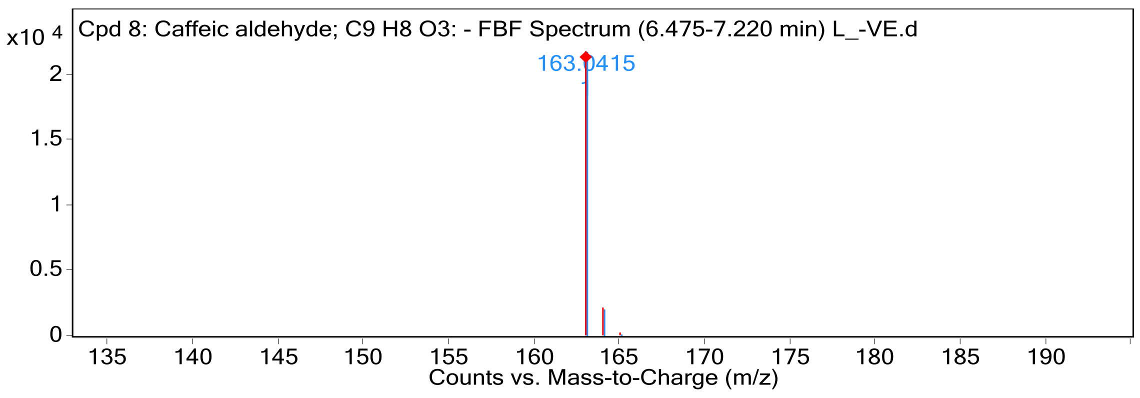

MS Spectrum Peak List

m/z	z	Abund	Formula	Ion
163.0415	1	21782.78	C9 H8 O3	(M-H)-
164.0445	1	2080.31	C9 H8 O3	(M-H)-

m/z	z	Abund	Formula	Ion
165.0468	1	242.24	C9 H8 O3	(M-H)-

MSMS Spectrum

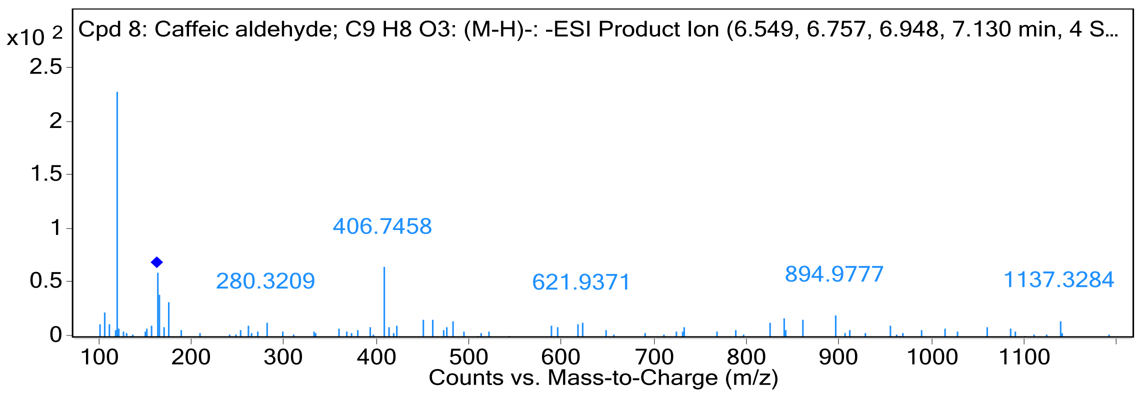

MS/MS Spectrum Peak List

m/z	z	Abund
106.0405		22.68
119.0496	1	228.56
163.0342	2	60.21
164.8359		39.52
174.5847		33.31
406.7458		65.13
449.7887		15.81
458.7679		16.16
838.3779		18
894.9777		20.56

Compound Structure

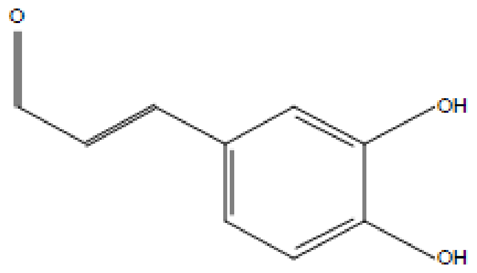

Compound Label	Name	*m/z*	RT	Algorithm	Mass
Cpd 9: Ferulic acid; C10 H10 O4	**Ferulic acid**	193.052	7.055	Find By Formula	194.0593

MS Spectrum

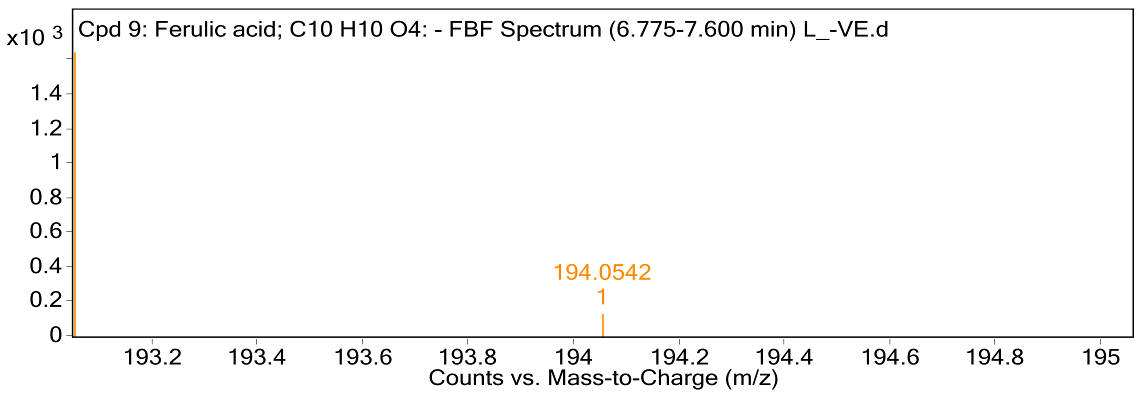

MS Zoomed Spectrum

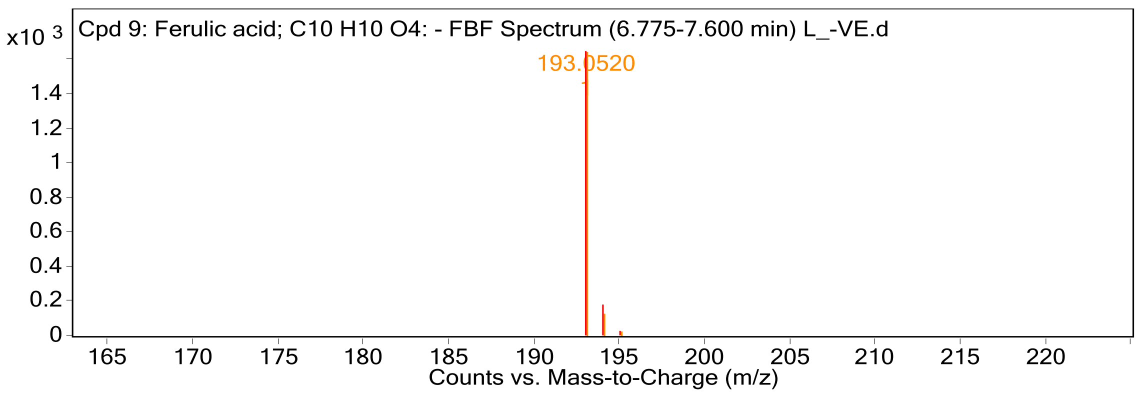

MS Spectrum Peak List

m/z	z	Abund	Formula	Ion
193.052	1	1646.52	C10 H10 O4	(M-H)-
194.0542	1	139.12	C10 H10 O4	(M-H)-
195.0606	1	34	C10 H10 O4	(M-H)-

Compound Structure

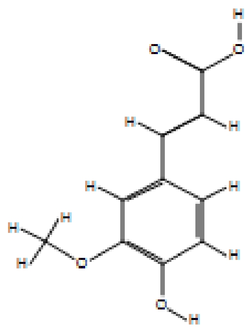

Compound Label	Name	*m/z*	RT	Algorithm	Mass
Cpd 10: Ferulic acid; C10 H10 O4	**Ferulic acid**	193.052	7.055	Find By Formula	194.0593

MS Spectrum

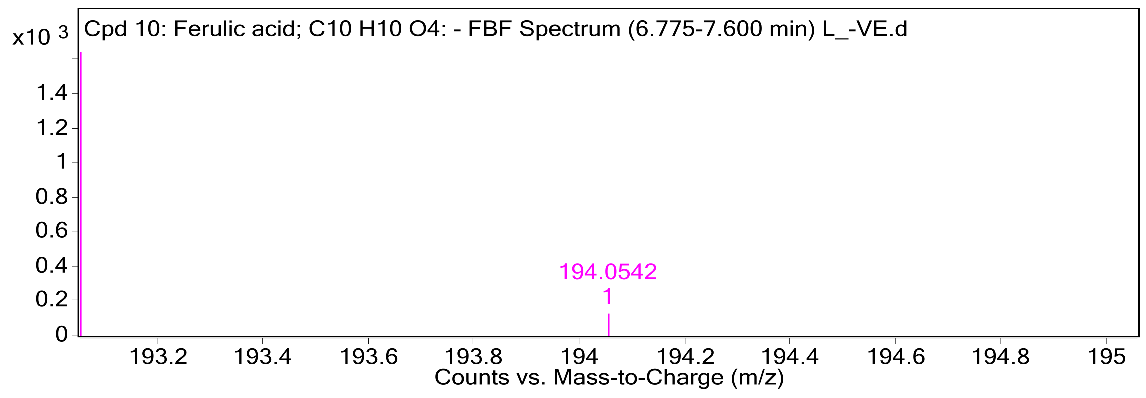

MS Zoomed Spectrum

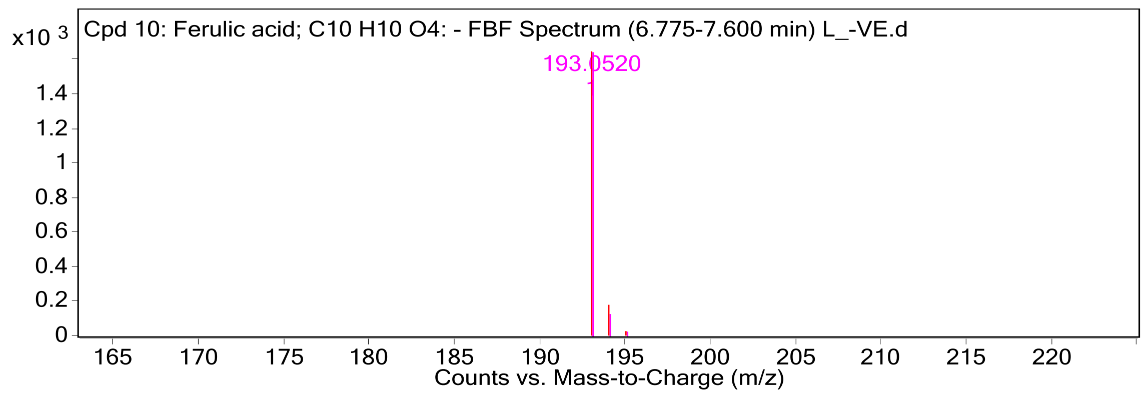

MS Spectrum Peak List

m/z	z	Abund	Formula	Ion
193.052	1	1646.52	C10 H10 O4	(M-H)-
194.0542	1	139.12	C10 H10 O4	(M-H)-
195.0606	1	34	C10 H10 O4	(M-H)-

Compound Structure

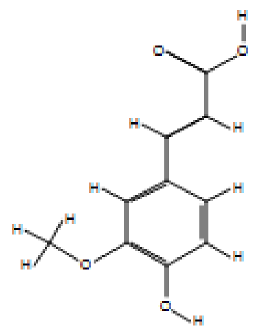

Compound Label	*m/z*	RT	Algorithm	Mass
Cpd 11: C9 H6 O2	145.031	8.782	Find By Formula	146.0373

MS Spectrum

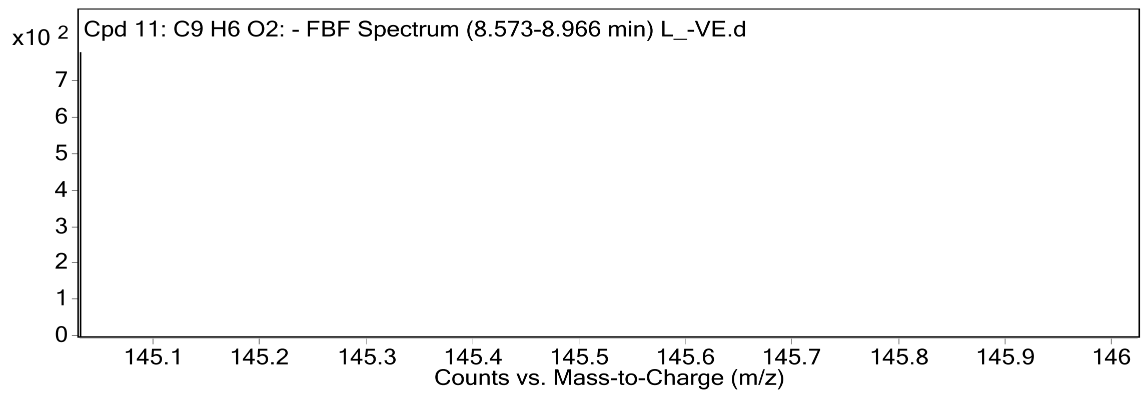

MS Zoomed Spectrum

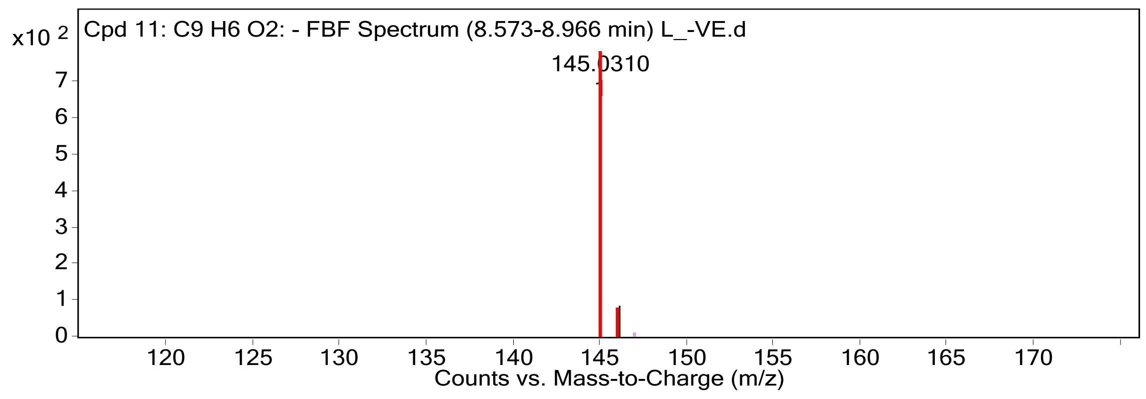

MS Spectrum Peak List

m/z	z	Abund	Formula	Ion
145.031	1	781.82	C9 H6 O2	(M-H)-
146.0246	1	87.4	C9 H6 O2	(M-H)-

Compound Label	Name	*m/z*	RT	Algorithm	Mass
Cpd 12: Ellagic acid; C14 H6 O8	**Ellagic acid**	301.0014	7.584	Find By Formula	302.0087

MS Spectrum

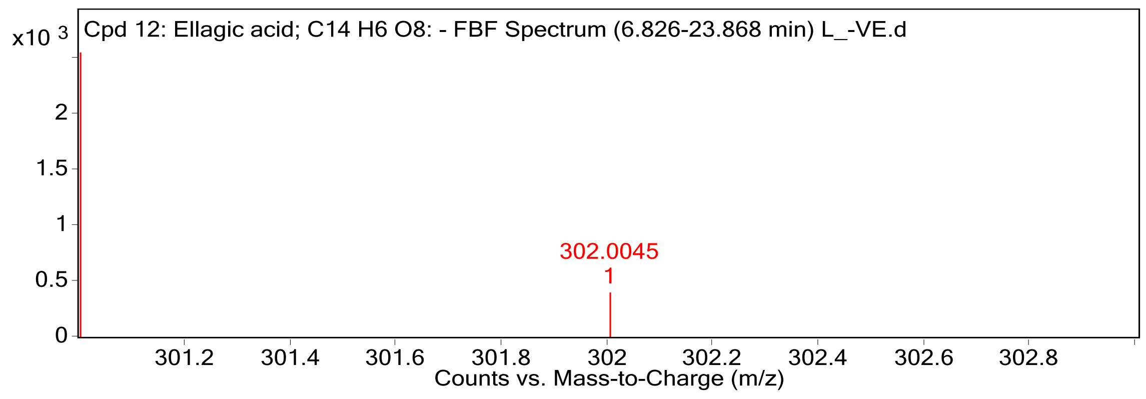

MS Zoomed Spectrum

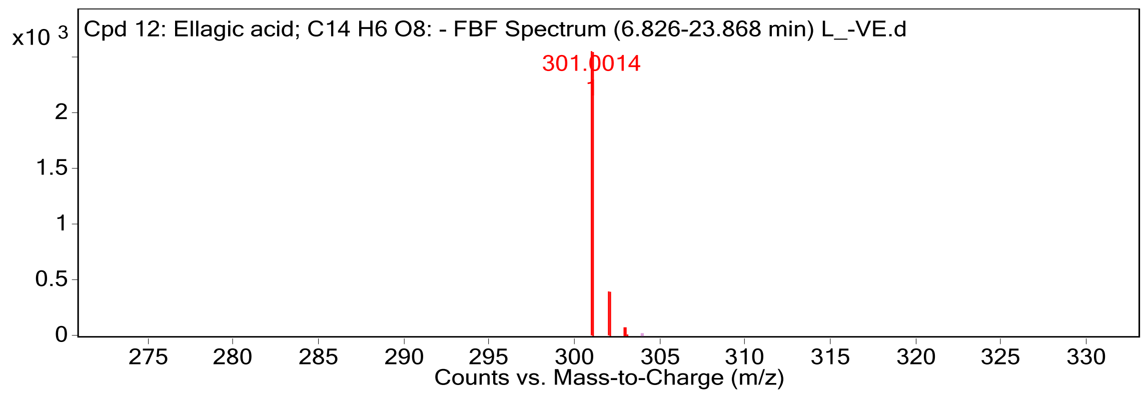

MS Spectrum Peak List

m/z	z	Abund	Formula	Ion
301.0014	1	2557.15	C14 H6 O8	(M-H)-
302.0045	1	408.14	C14 H6 O8	(M-H)-
303.0058	1	29.47	C14 H6 O8	(M-H)-

Compound Structure

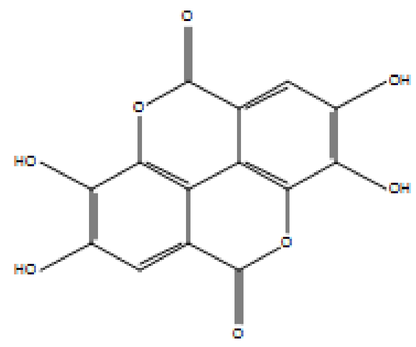

Compound Label	*m/z*	RT	Algorithm	Mass
Cpd 13: C14 H12 O3	287.092	2.084	Find By Formula	228.078

MS Spectrum

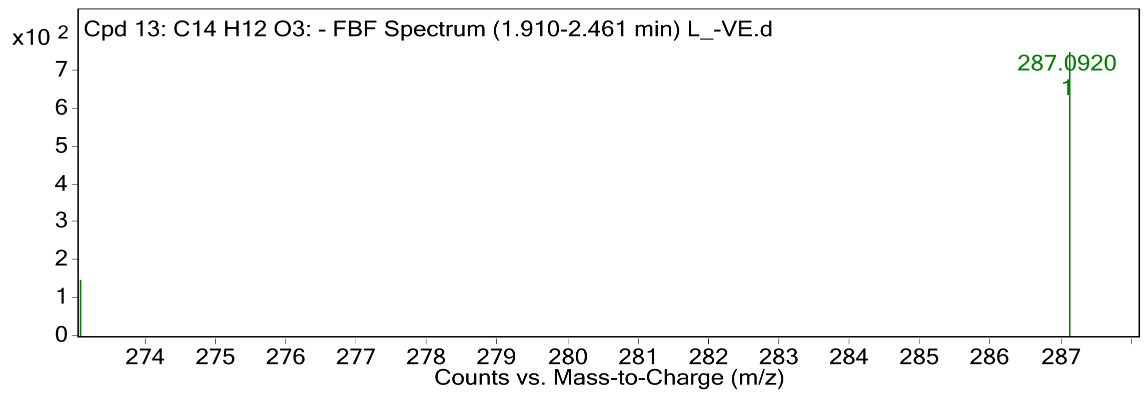

MS Zoomed Spectrum

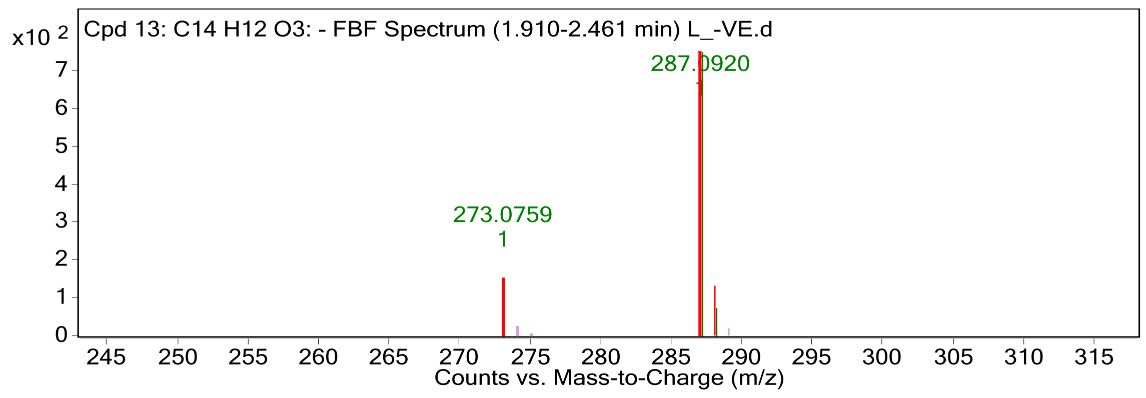

MS Spectrum Peak List

m/z	z	Abund	Formula	Ion
273.0759	1	150.34	C14 H12 O3	(M+HCOO)-
287.092	1	750.45	C14 H12 O3	(M+CH3COO)-
288.095	1	77.91	C14 H12 O3	(M+CH3COO)-

Compound Label	Name	*m/z*	RT	Algorithm	Mass
Cpd 14: Caffeic aldehyde; C9 H8 O3	**Caffeic aldehyde**	163.0415	6.679	Find By Formula	164.0487

MS Spectrum

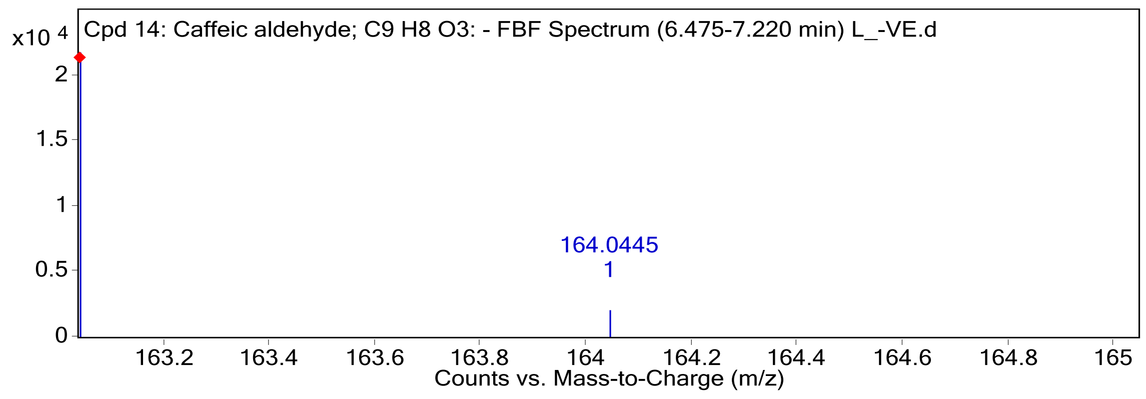

MS Zoomed Spectrum

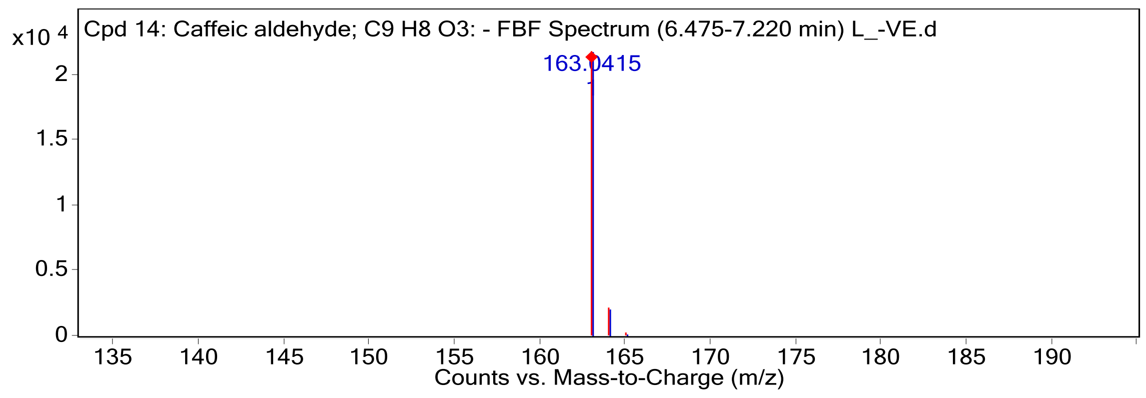

MS Spectrum Peak List

m/z	z	Abund	Formula	Ion
163.0415	1	21782.78	C9 H8 O3	(M-H)-
164.0445	1	2080.31	C9 H8 O3	(M-H)-
165.0468	1	242.24	C9 H8 O3	(M-H)-

MSMS Spectrum

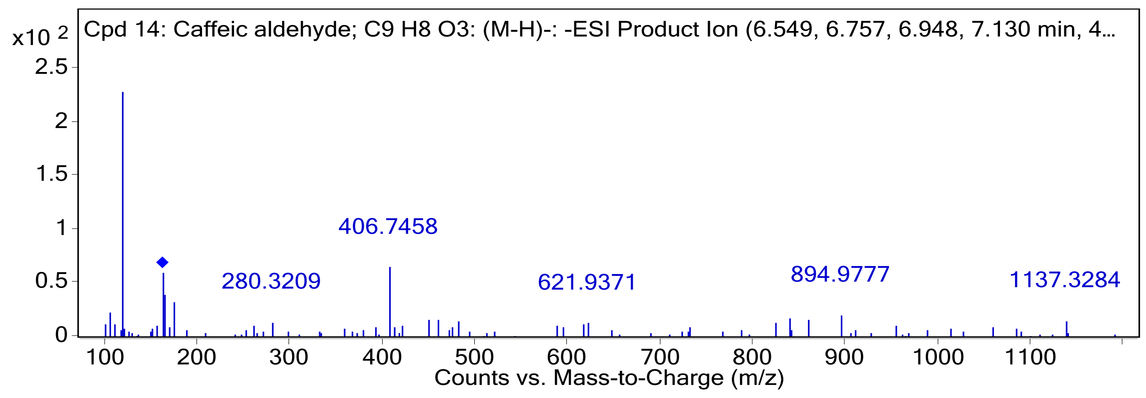

MS/MS Spectrum Peak List

m/z	z	Abund
106.0405		22.68
119.0496	1	228.56
163.0342	2	60.21
164.8359		39.52
174.5847		33.31
406.7458		65.13
449.7887		15.81
458.7679		16.16
838.3779		18
894.9777		20.56

Compound Structure

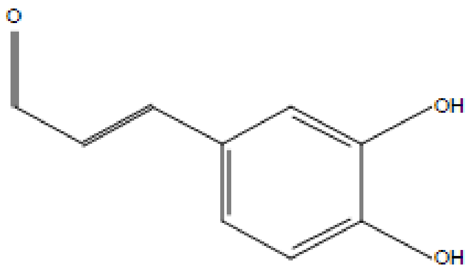

Compound Label	Name	*m/z*	RT	Algorithm	Mass
Cpd 15: Malic acid; C4 H6 O5	**Malic acid**	133.0154	6.775	Find By Formula	134.0227

MS Spectrum

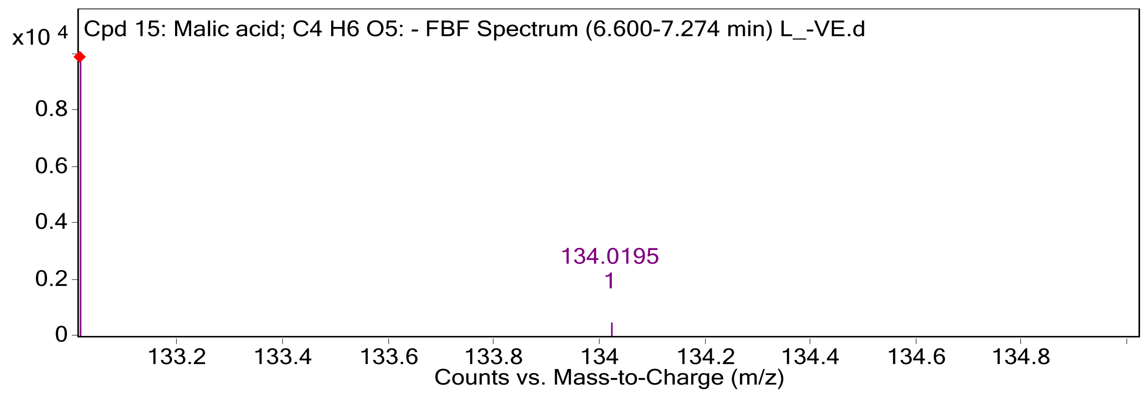

MS Zoomed Spectrum

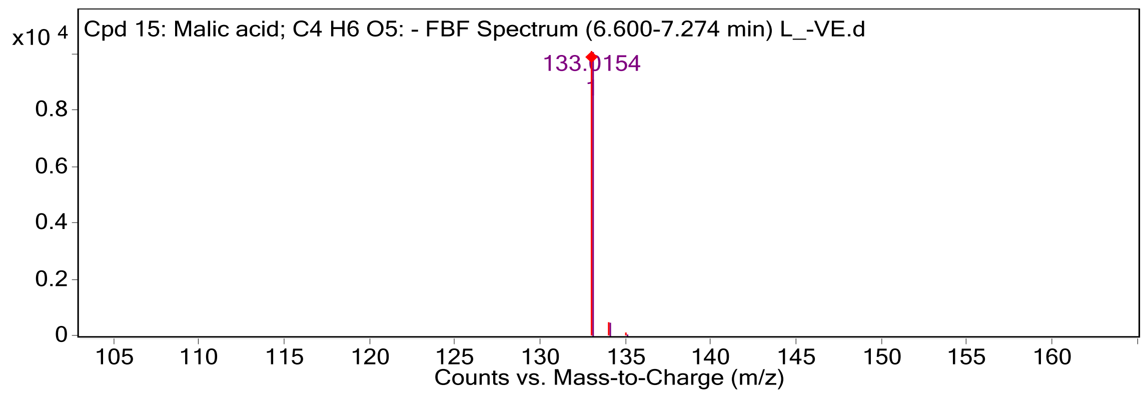

MS Spectrum Peak List

m/z	z	Abund	Formula	Ion
133.0154	1	10108.19	C4 H6 O5	(M-H)-
134.0195	1	532.73	C4 H6 O5	(M-H)-
135.0205	1	113.33	C4 H6 O5	(M-H)-

MSMS Spectrum

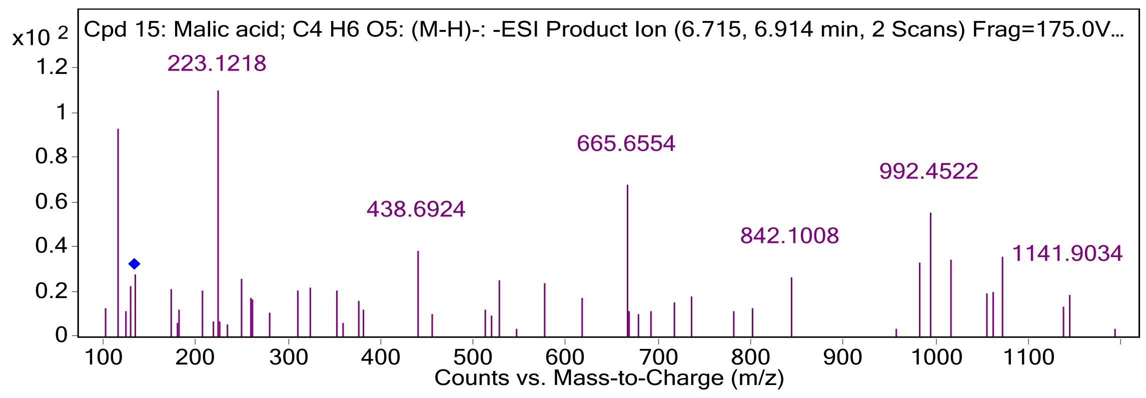

MS/MS Spectrum Peak List

m/z	Abund
114.9775	92.88
134.0488	28.26
223.1218	110.29
438.6924	39.04
665.6554	68.48
842.1008	27.16
980.0083	33.51
992.4522	56.04
1014.7449	35.02
1069.0696	35.82

Compound Structure

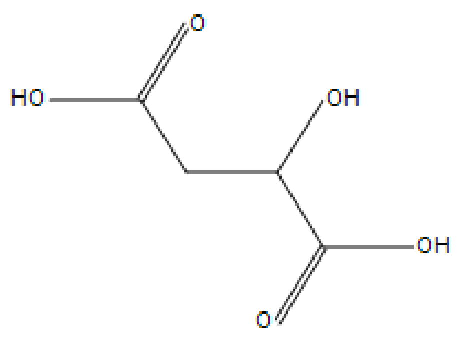

Compound Label	*m/z*	RT	Algorithm	Mass
Cpd 16: C6 H8 O7	191.02	2.134	Find By Formula	192.0272

MS Spectrum

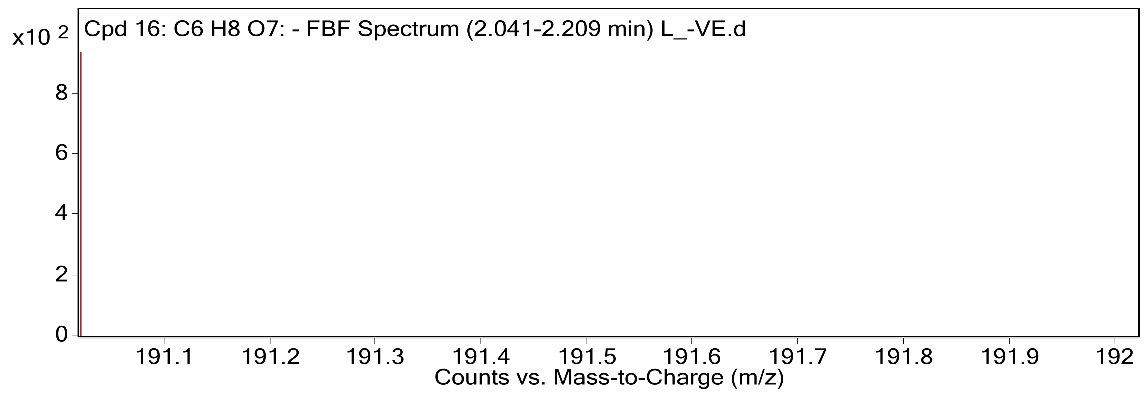

MS Zoomed Spectrum

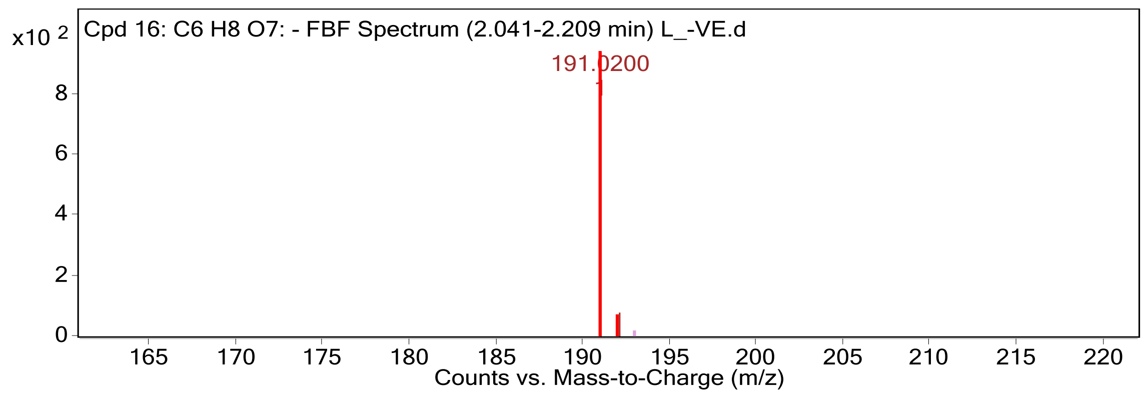

MS Spectrum Peak List

m/z	z	Abund	Formula	Ion
191.02	1	939.87	C6 H8 O7	(M-H)-
192.0219	1	82.53	C6 H8 O7	(M-H)-

Compound Label	Name	*m/z*	RT	Algorithm	Mass
Cpd 17: Ribose-1-arsenate; C5 H11 As O8	**Ribose-1-arsenate**	272.961	1.505	Auto MS/MS	273.9661

MS Spectrum

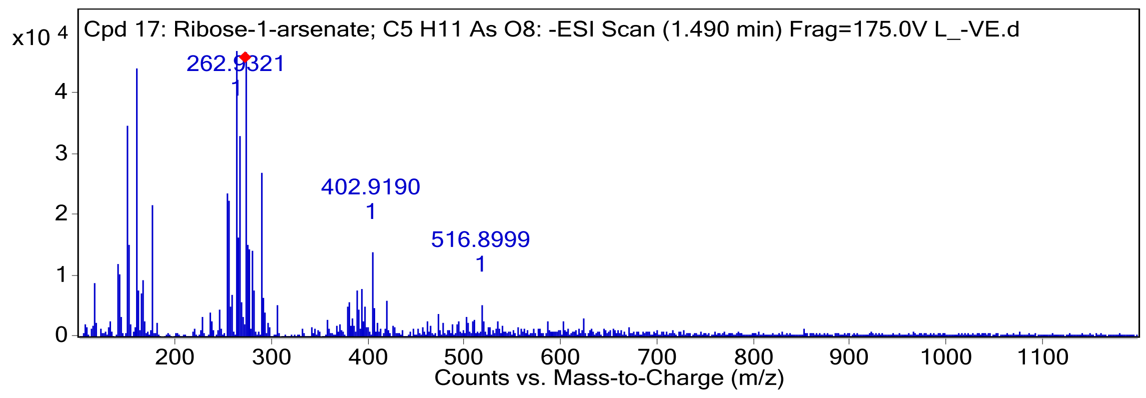

MS Zoomed Spectrum

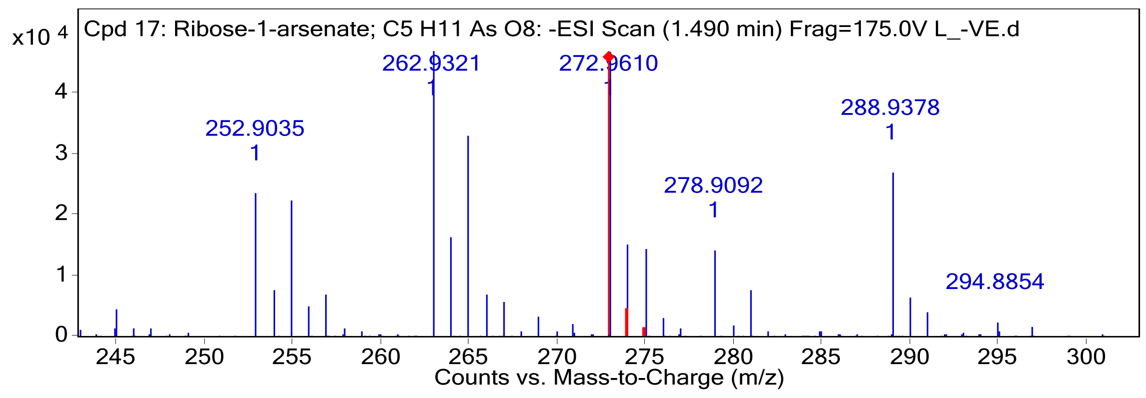

MS Spectrum Peak List

m/z	*Calc m/z*	Diff(ppm)	z	Abund	Formula	Ion
148.9509			1	34775.92		
158.9797			1	44141.77		
252.9035			1	23563.42		
262.9321			1	49265.35		
264.9295			1	32998.34		
272.961	272.9597	-4.6	1	46846.44	C5 H11 As O8	(M-H)-
273.9614	273.9632	6.34	1	15155.31	C5 H11 As O8	(M-H)-
274.9588	274.9642	19.5	1	14468.44	C5 H11 As O8	(M-H)-
275.96	275.9675	27.14	1	3069.12	C5 H11 As O8	(M-H)-
288.9378			1	27001.31		

MSMS Spectrum

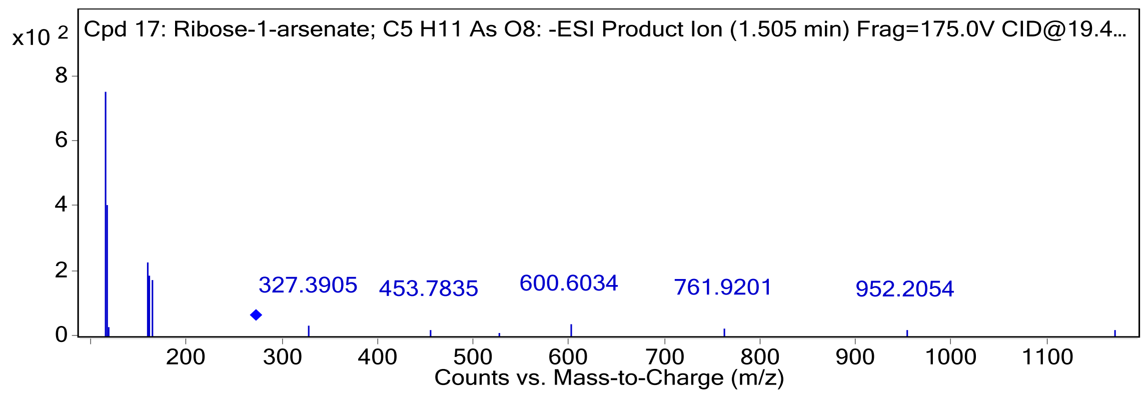

MS/MS Spectrum Peak List

m/z	Abund
114.9696	361.87
114.9904	755.71
115.991	306.41
116.9809	404.59
158.951	90.74
158.977	229.41
160.9768	190.26
162.9633	177.42
327.3905	34.11
600.6034	40.17

Qualitative Compound Report

Compound Structure

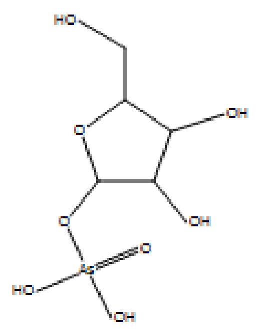

Compound Label	*m/z*	RT	Algorithm
Compound 18	402.919	1.523	Auto MS/MS

MS Spectrum

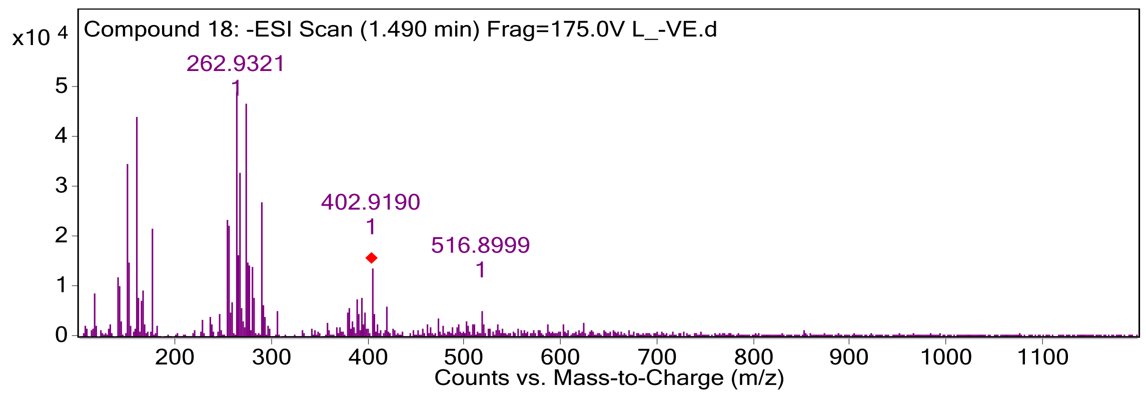

MS Zoomed Spectrum

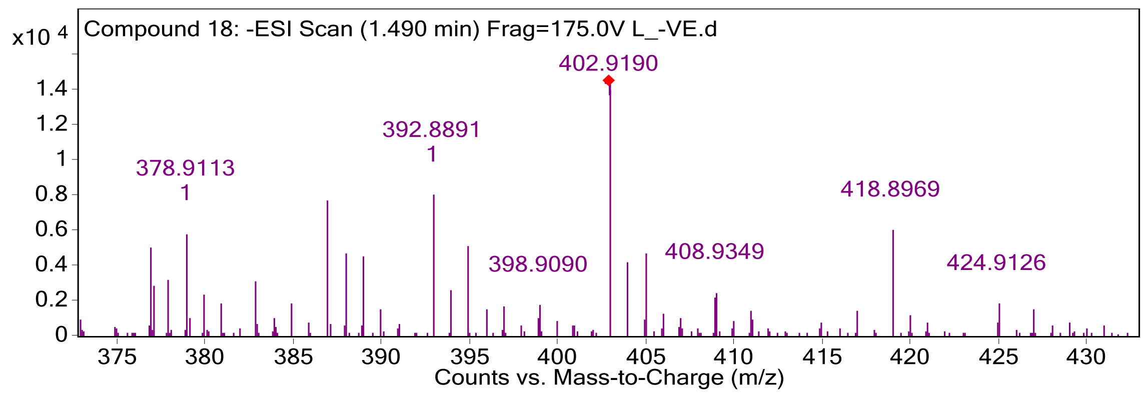

MS Spectrum Peak List

m/z	z	Abund
148.9509	1	34775.92
158.9797	1	44141.77
262.9321	1	49265.35
264.9295	1	32998.34
272.961	1	46846.44
402.919	1	13961.72
403.9188	1	4239.2
404.9184	1	4781.79
405.9212	1	1310.83
406.915	1	1081.12

MSMS Spectrum

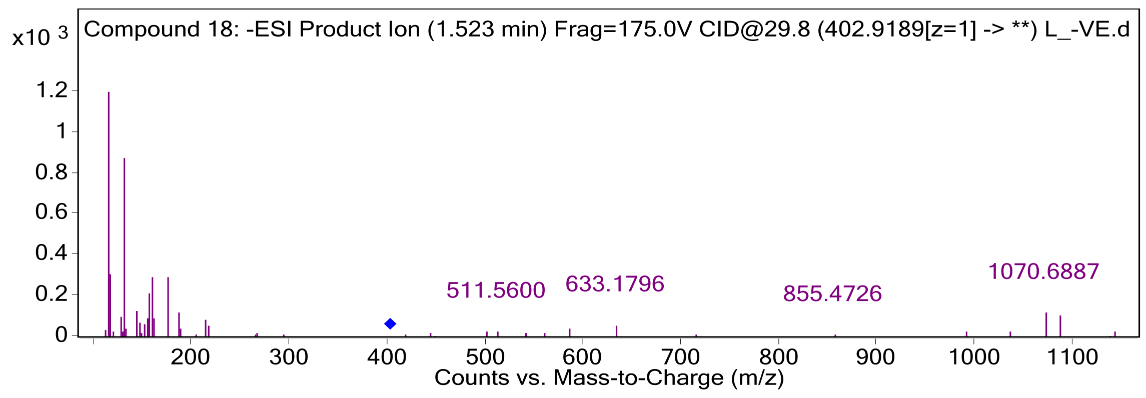

MS/MS Spectrum Peak List

m/z	Abund
114.9898	1202.08
116.9875	311.43
130.9507	173.97
130.9673	877.83
156.0082	216.03
158.9526	207.3
158.98	144.08
159.9828	292.82
174.9278	215.73
174.9496	297.91

Compound Label	Name	*m/z*	RT	Algorithm	Mass
Cpd 19: 2,3,5,7,9-Pentathiadecane 2,2-dioxide; C5 H12 O2 S5	**2,3,5,7,9-Pentathiadecane 2,2-dioxide**	262.9316	1.544	Auto MS/MS	263.9397

MS Spectrum

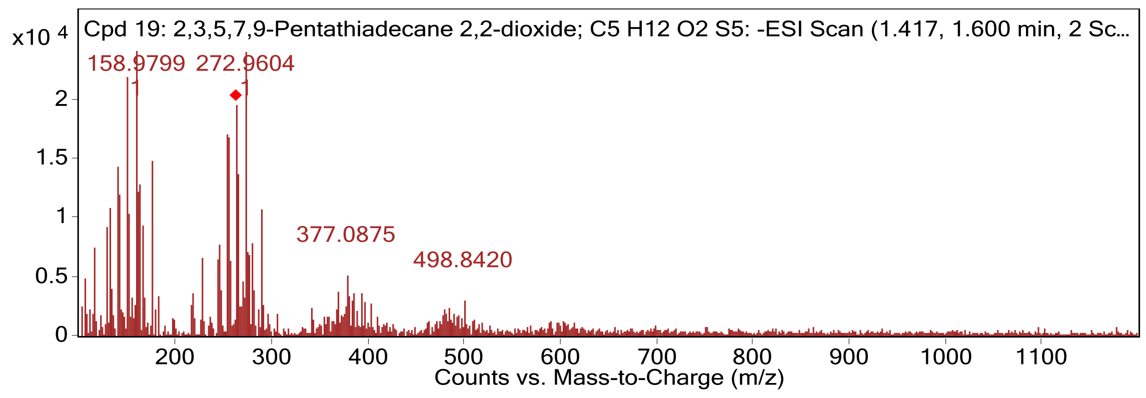

MS Zoomed Spectrum

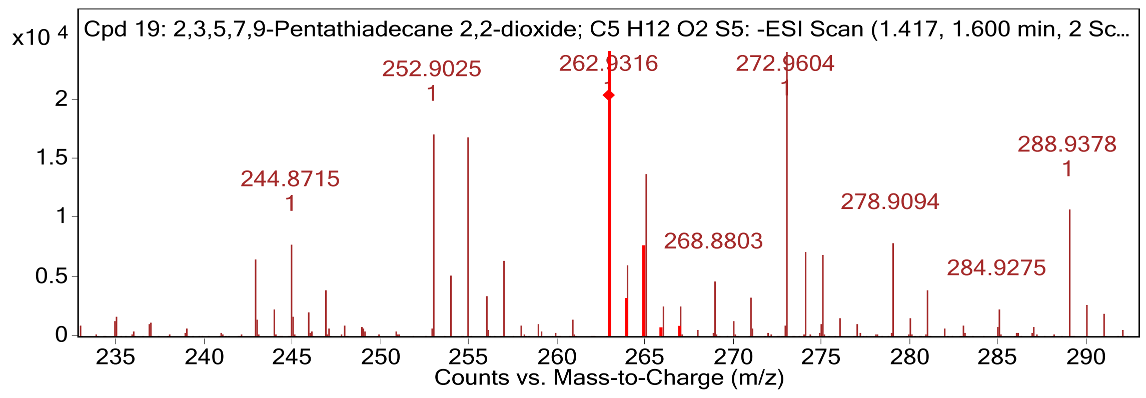

MS Spectrum Peak List

m/z	*Calc m/z*	Diff(ppm)	z	Abund	Formula	Ion
148.951			1	21960.83		
158.9799			1	35244.39		
252.9025			1	17170.21		
254.9001			1	16839.69		
262.9316	262.9368	19.72	1	19558.99	C5 H12 O2 S5	(M-H)-
263.9337	263.9386	18.26	1	6049.09	C5 H12 O2 S5	(M-H)-
264.9294	264.9329	13.2	1	13720.38	C5 H12 O2 S5	(M-H)-
265.9314	265.9348	12.81	1	2647.02	C5 H12 O2 S5	(M-H)-
266.9266	266.9291	9.32	1	2586.1	C5 H12 O2 S5	(M-H)-
272.9604			1	24080.01		

MSMS Spectrum

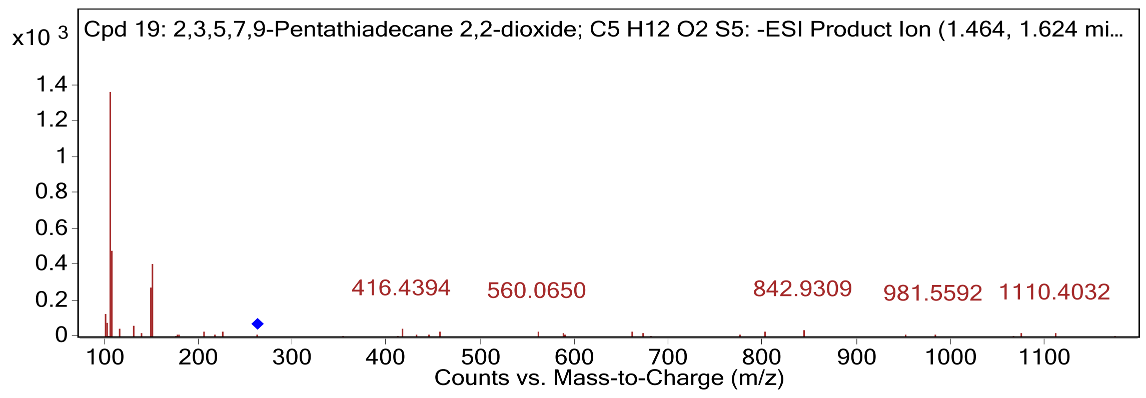

MS/MS Spectrum Peak List

m/z	z	Abund
100.9401		127.57
101.9629		78.65
104.9613	1	1368.95
105.9603	1	158.71
106.9385		90.06
106.956	1	479.34
131.0482		64.12
148.9512		274.41
149.9526		141.75
150.9472		408.4

Compound Structure

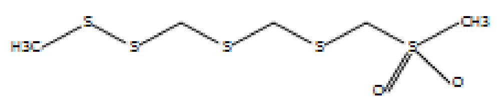

Compound Label	Name	*m/z*	RT	Algorithm	Mass
Cpd 20: DuP-697; C17 H12 Br F O2 S2	**DuP-697**	408.9363	1.582	Auto MS/MS	409.9425

MS Spectrum

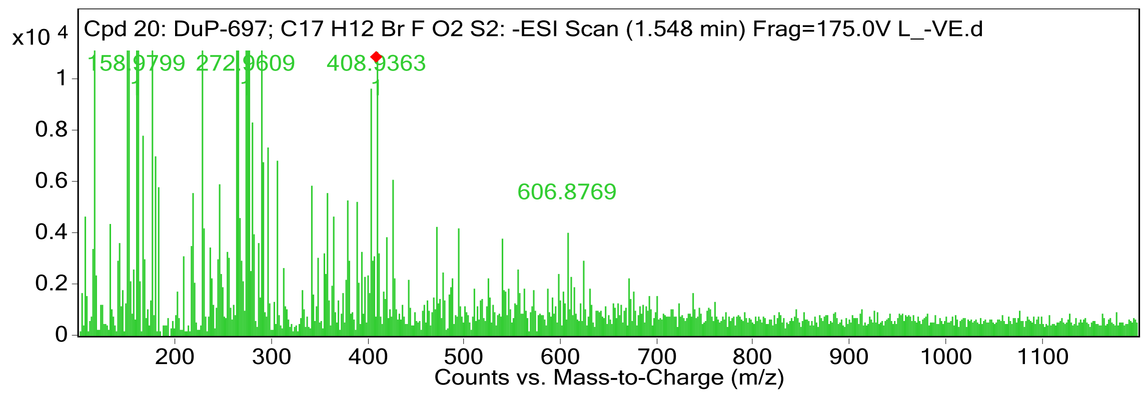

MS Zoomed Spectrum

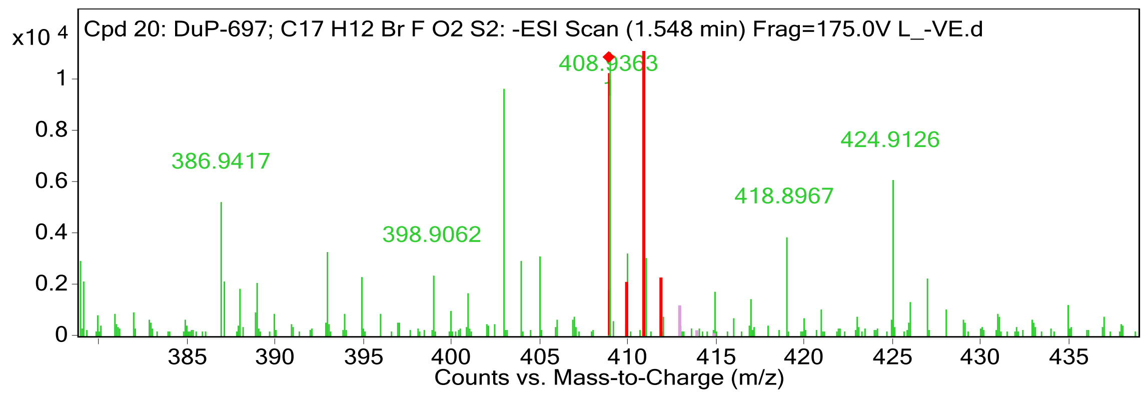

MS Spectrum Peak List

m/z	*Calc m/z*	Diff(ppm)	z	Abund	Formula	Ion
158.9799			1	88468.8		
174.9576				32115.77		
262.9322			1	28583.47		
272.9609			1	84733.77		
274.9592			1	28204.88		
288.9383			1	36578.25		
408.9363	408.9373	2.57	1	11062.71	C17 H12 Br F O2 S2	(M-H)-
409.9383	409.9404	5.02	1	3265.95	C17 H12 Br F O2 S2	(M-H)-

410.93	410.9353	12.84	1	3096.18	C17 H12 Br F O2 S2	(M-H)-
411.934	411.9383	10.4	1	774.8	C17 H12 Br F O2 S2	(M-H)-

MSMS Spectrum

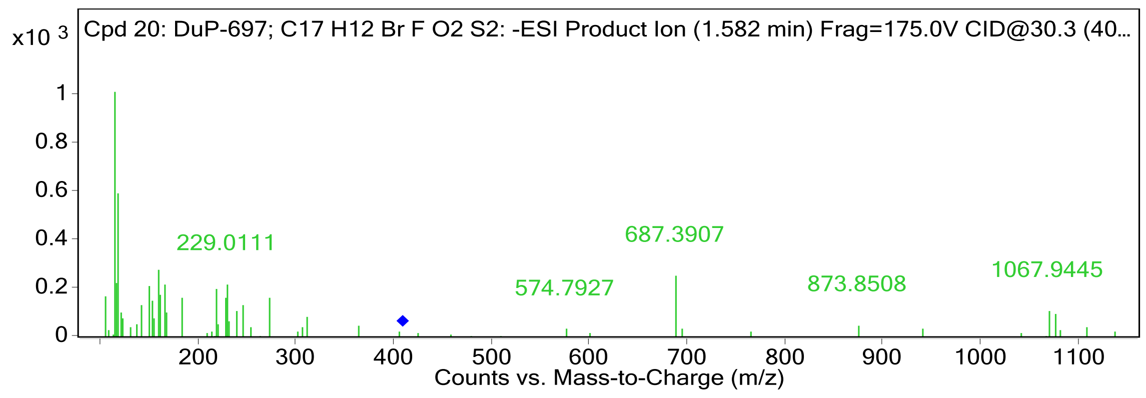

MS/MS Spectrum Peak List

m/z	z	Abund
114.9702		255.35
114.9894	1	1012.72
115.9916	1	224.56
116.9873	1	593.01
149.9247		210.08
158.9769		278.65
164.9264		219.67
218.0059		198.16
229.0111		220.94
687.3907		255.63

Compound Structure

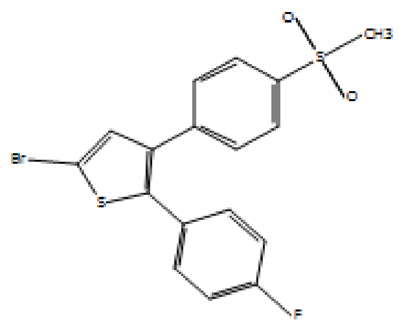

Compound Label	Name	*m/z*	RT	Algorithm	Mass
Cpd 21: Apigenin 7-[rhamnosyl-(1->2)-galacturonide]; C27 H28	**Apigenin 7-[rhamnosyl-(1->2)-galacturonide]**	591.1392	8.028	Auto MS/MS	592.1465

MS Spectrum

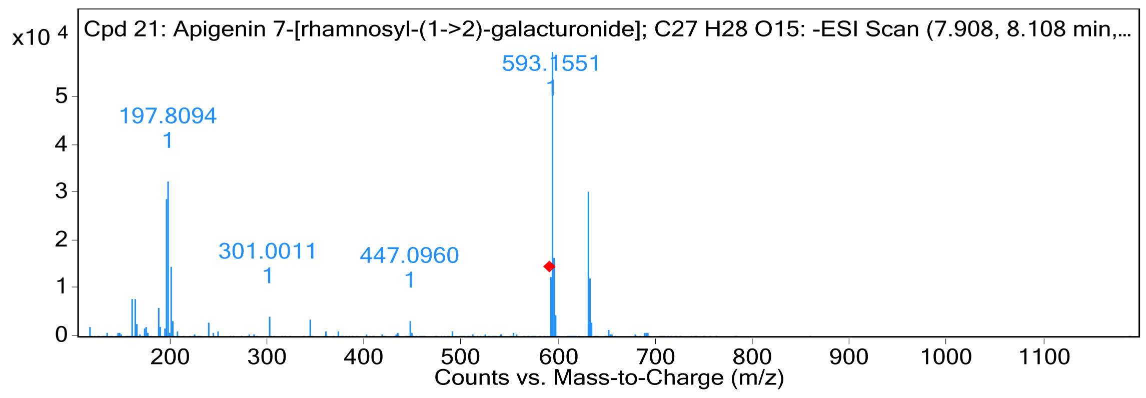

MS Zoomed Spectrum

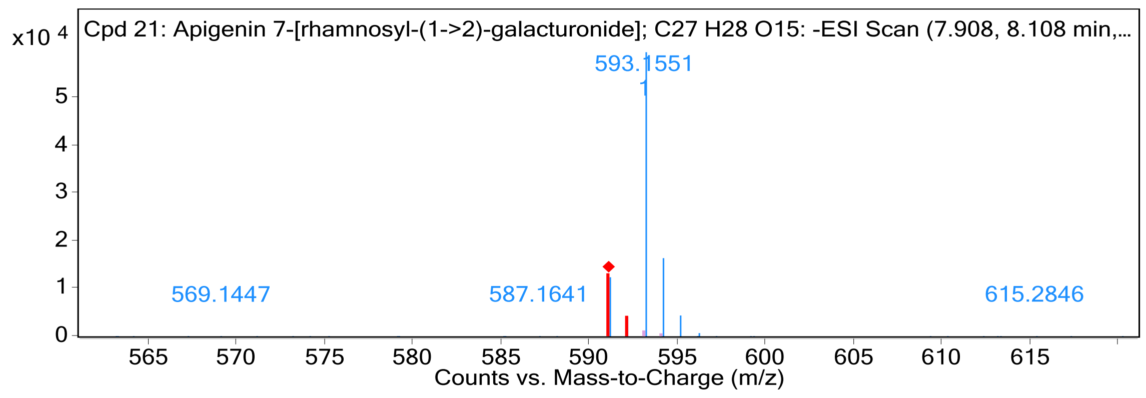

MS Spectrum Peak List

m/z	*Calc m/z*	Diff(ppm)	z	Abund	Formula	Ion
195.8128			1	28914.28		
197.8094			1	32587.03		
199.8064			1	14667.26		
591.1392	591.1355	-6.27	1	12520.66	C27 H28 O15	(M-H)-
592.1426	592.1389	-6.21	1	4274.16	C27 H28 O15	(M-H)-
593.1551			1	59538.25		
594.1583			1	16484.41		
629.1324			1	30411.37		
630.1351			1	8823.93		
631.1304			1	12185.68		

MSMS Spectrum

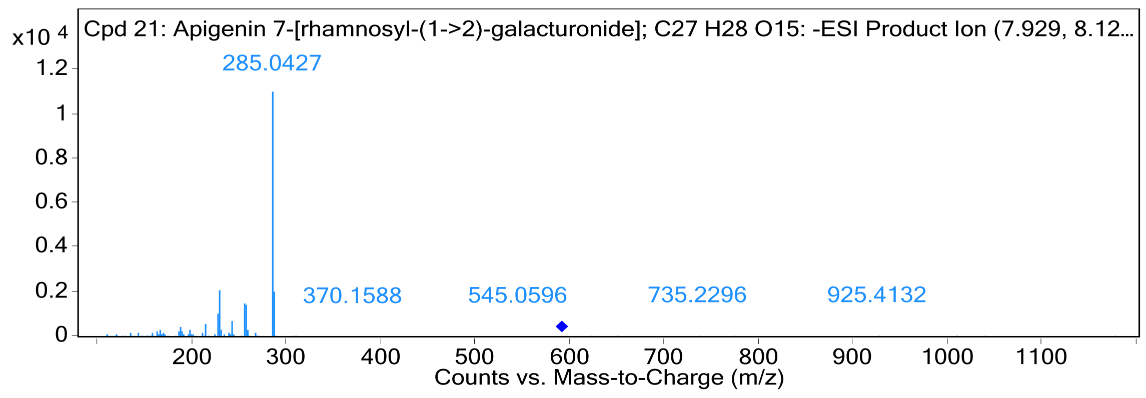

MS/MS Spectrum Peak List

m/z	z	Abund
187.0364	1	476.89
213.0575	1	618.27
227.0366	1	1086.45
229.0503	1	2117.09

241.0509	1	709.93
255.0324	1	1503.74
257.0462	1	1437.21
284.0341		2366.01
285.0427	1	11046.75
286.0458	1	2064.34

Compound Structure

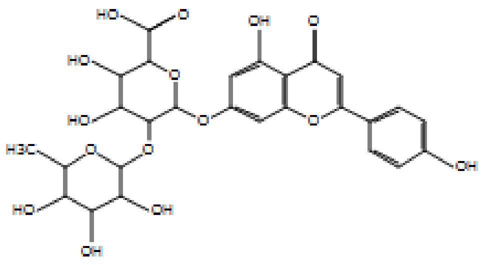

Compound Label	Name	m/z	RT	Algorithm	Mass
Cpd 22: CMP-N-glycoloylneuraminate; C20 H31 N4 O17 P	**CMP-N-glycoloylneuraminate**	629.1318	8.096	Auto MS/MS	630.1376

MS Spectrum

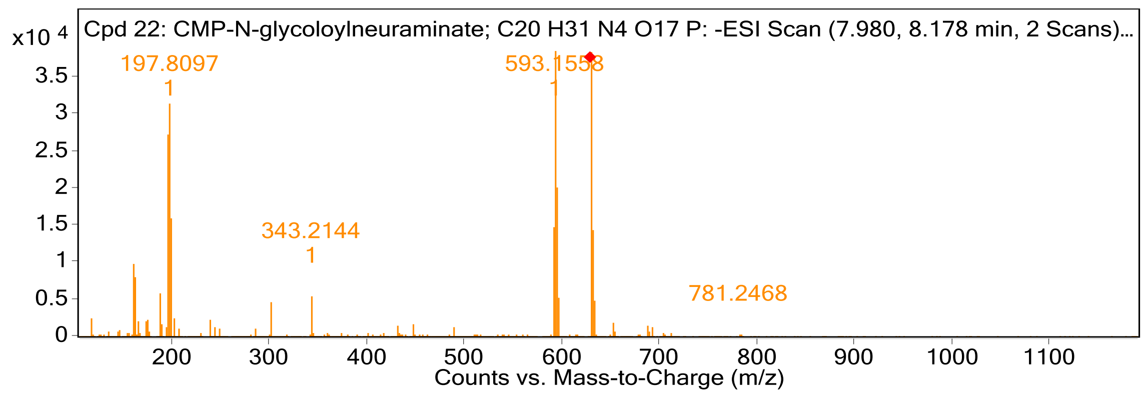

MS Zoomed Spectrum

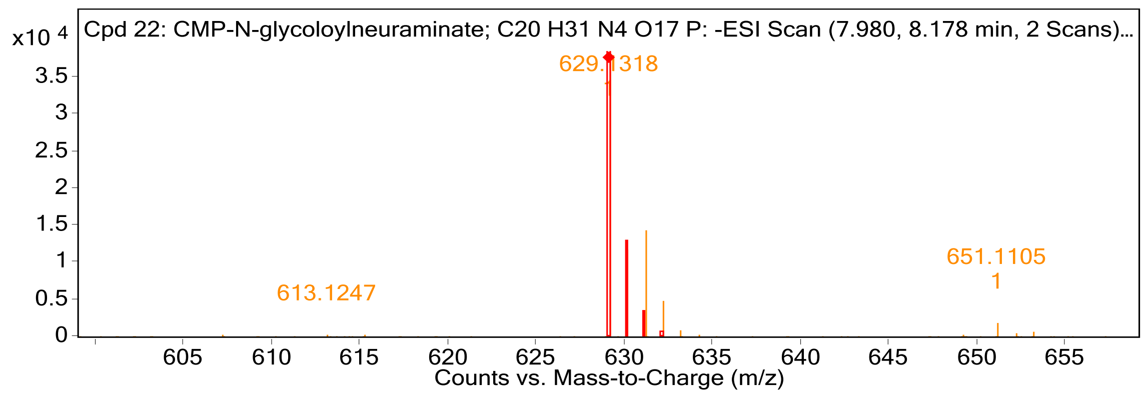

MS Spectrum Peak List

m/z	*Calc m/z*	Diff(ppm)	z	Abund	Formula	Ion
195.8128			1	27398.82		
197.8097			1	31491.51		
199.8068			1	15958.57		
593.1558			1	71946.37		
594.1591			1	20198.75		
629.1318	629.1349	4.98	1	38379.7	C20 H31 N4 O17 P	(M-H)-
630.1362	630.1379	2.76	1	11777.72	C20 H31 N4 O17 P	(M-H)-
631.1308	631.14	14.44	1	14457.44	C20 H31 N4 O17 P	(M-H)-
632.1335	632.1425	14.34	1	4850.65	C20 H31 N4 O17 P	(M-H)-
633.1377	633.1447	11.09	1	902.99	C20 H31 N4 O17 P	(M-H)-

MSMS Spectrum

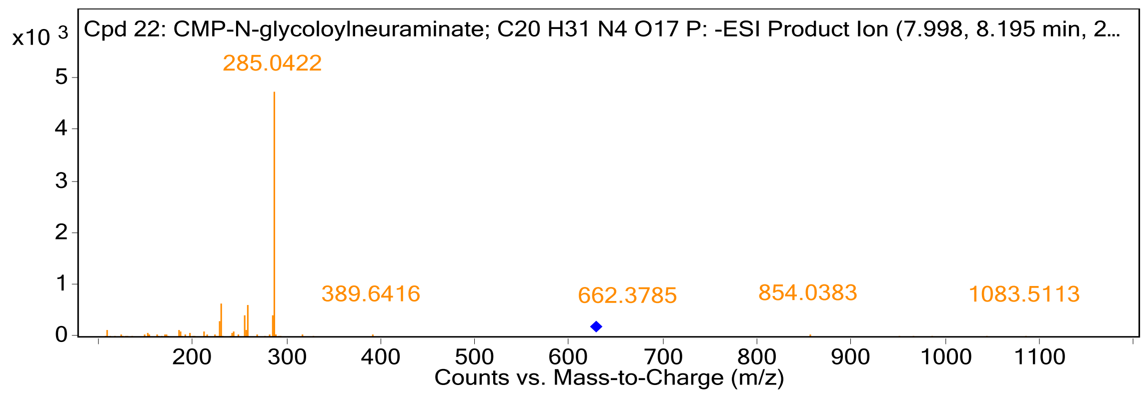

MS/MS Spectrum Peak List

m/z	z	Abund
185.0588		155.68
227.037		319.66
229.0518	1	668.35
255.0286	1	420.78
257.0486		638
258.0522		295.07
283.9964		157.93
284.0362		433.42
285.0422	1	4757.22
286.0447	1	1143.88

Compound Structure

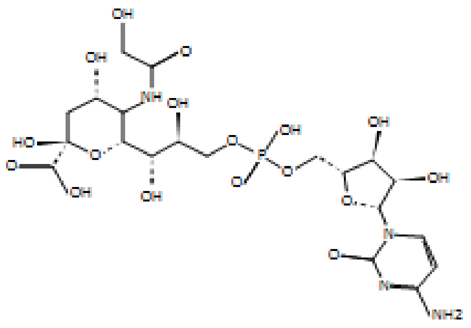

Compound Label	Name	*m/z*	RT	Algorithm	Mass
Cpd 23: Nicotiflorin; C27 H30 O15	**Nicotiflorin**	593.1559	8.277	Auto MS/MS	594.1631

MS Spectrum

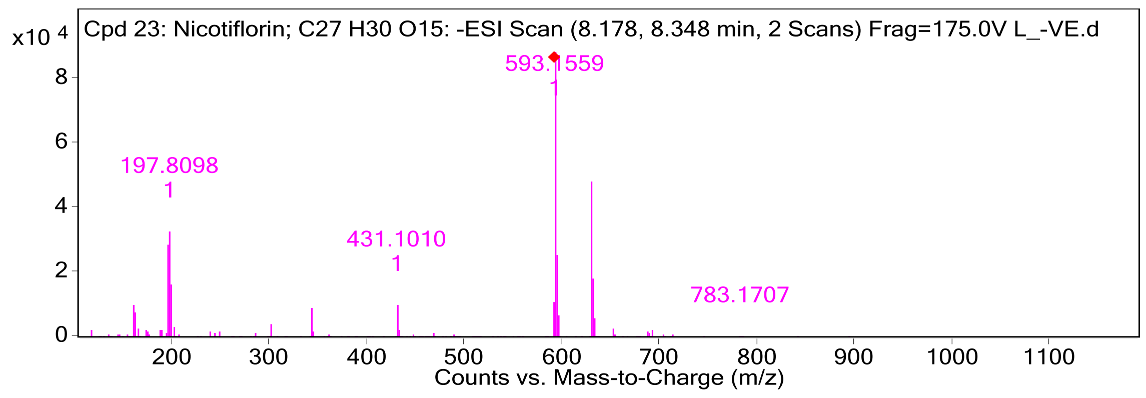

MS Zoomed Spectrum

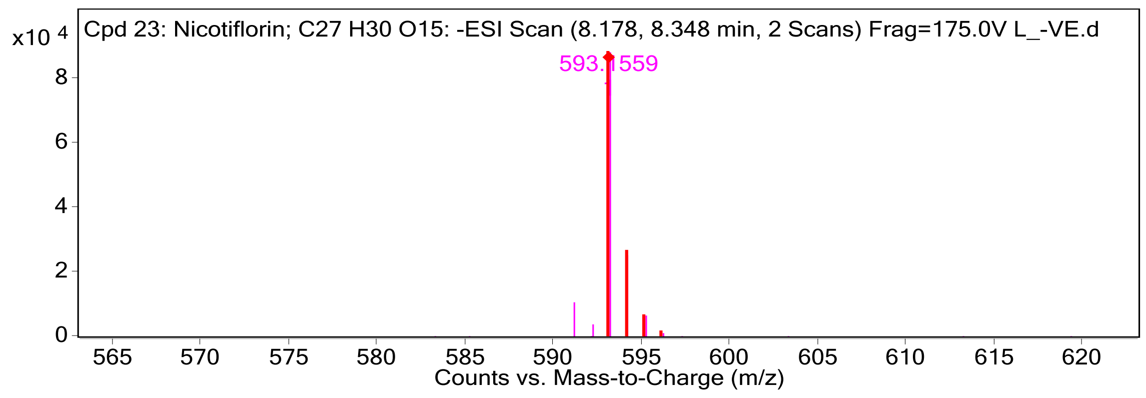

MS Spectrum Peak List

m/z	*Calc m/z*	Diff(ppm)	z	Abund	Formula	Ion
195.8129			1	28539.63		
197.8098			1	32959.77		
199.8068			1	16551.73		
593.1559	593.1512	-7.91	1	88374.2	C27 H30 O15	(M-H)-
594.1589	594.1546	-7.22	1	25389.11	C27 H30 O15	(M-H)-
595.1619	595.1569	-8.41	1	6776.24	C27 H30 O15	(M-H)-
596.1638	596.1596	-7.07	1	1510.47	C27 H30 O15	(M-H)-
629.1321			1	48407.21		
630.136			1	14829.23		
631.1311			1	18047.67		

MSMS Spectrum

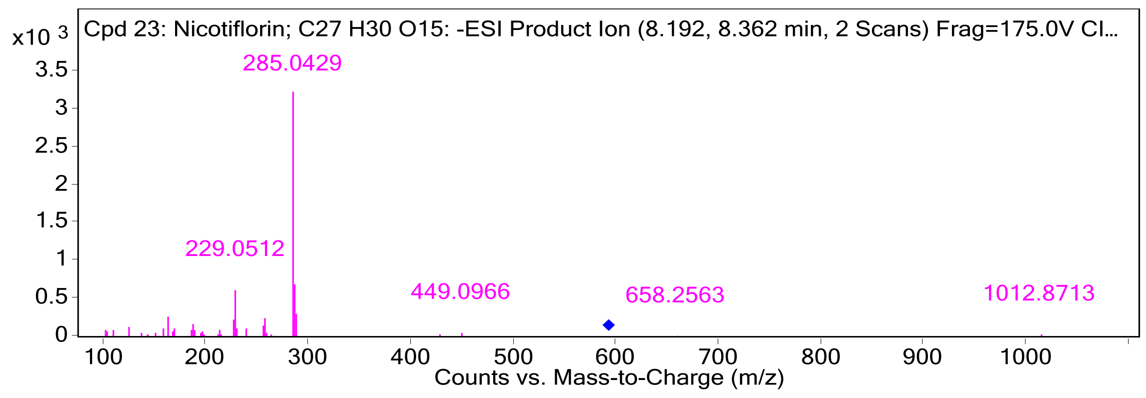

MS/MS Spectrum Peak List

m/z	z	Abund
163.0038		271.67
187.0388		170.69
227.0356	1	238.55
229.0512	1	625.51
255.0221		149.53
257.0477	1	258.37
284.035		403.61
285.0429	1	3232.32
286.0453	1	705.17
287.0447	1	302.63

Compound Structure

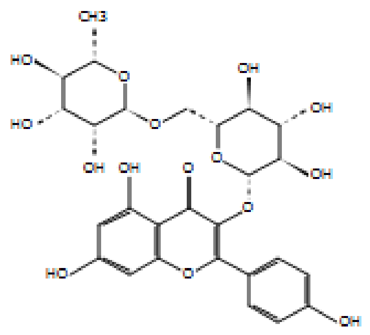

Compound Label	Name	*m/z*	RT	Algorithm	Mass
Cpd 24: Apigenin 7-[rhamnosyl-(1->2)-galacturonide]; C27 H28	**Apigenin 7-[rhamnosyl-(1->2)-galacturonide]**	591.14	8.321	Auto MS/MS	592.1464

MS Spectrum

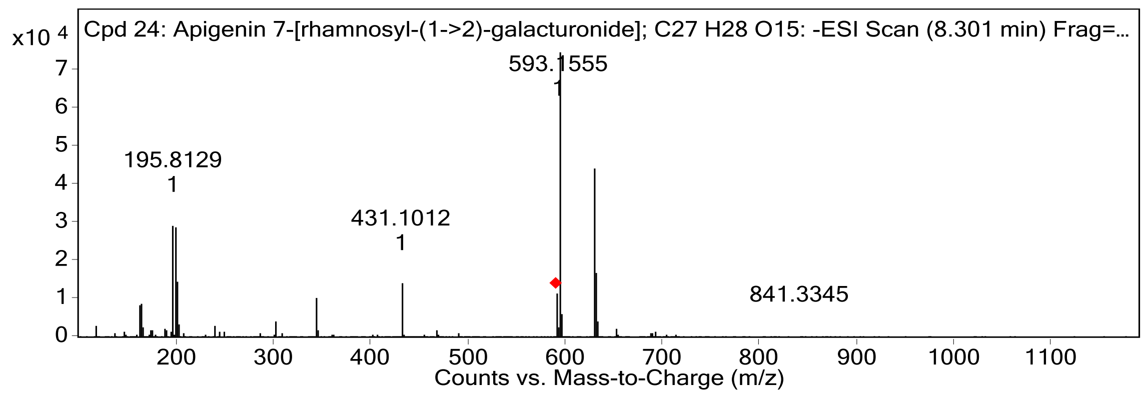

MS Zoomed Spectrum

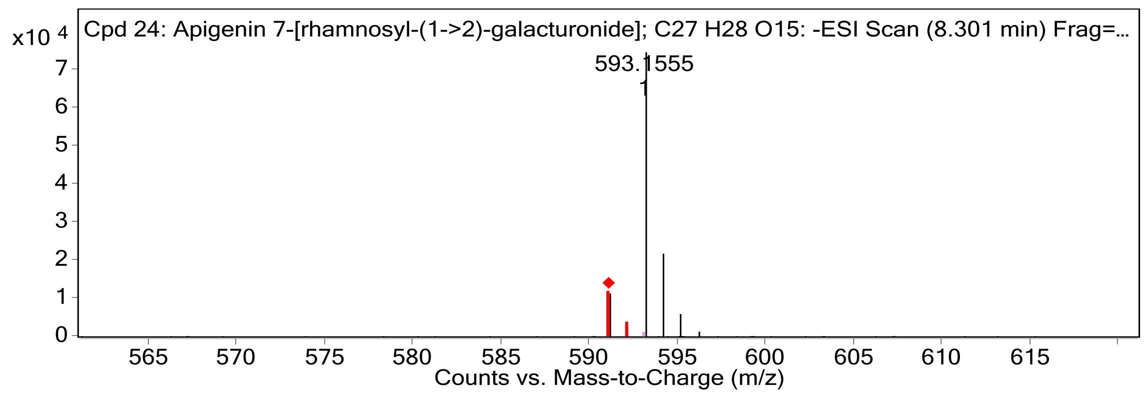

MS Spectrum Peak List

m/z	*Calc m/z*	Diff(ppm)	z	Abund	Formula	Ion
195.8129			1	29445.77		
197.8095			1	28858.39		
199.8064			1	14506.09		
431.1012			1	14114.16		
591.14	591.1355	-7.54	1	11579.25	C27 H28 O15	(M-H)-
592.1386	592.1389	0.67	1	2524.9	C27 H28 O15	(M-H)-
593.1555			1	74789.33		
594.1583			1	22166.38		
629.1329			1	44182.34		
631.1319			1	16826.33		

MSMS Spectrum

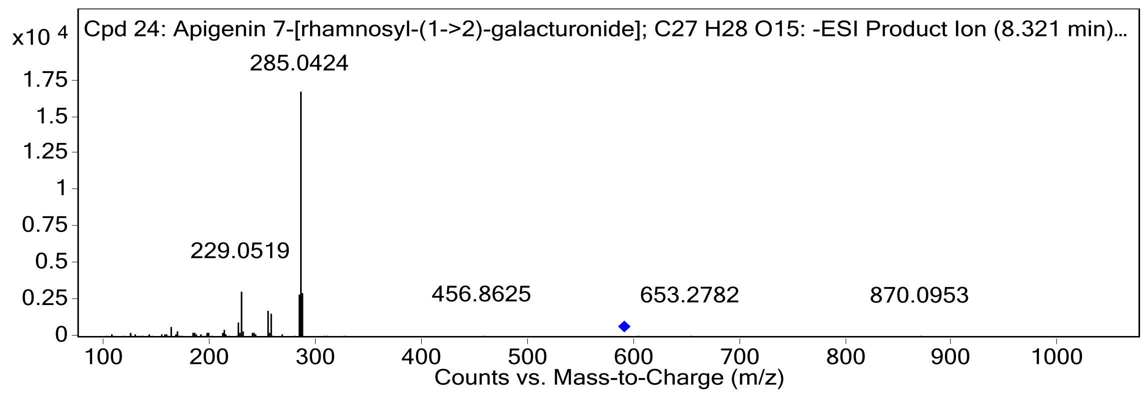

MS/MS Spectrum Peak List

m/z	z	Abund
163.0053		660.44
169.0676		400.97
213.0575		492.12
227.0375		1046.68
229.0519	1	3099.5
255.0334	1	1846.68
257.0437	2	1557.81
284.0364		2943.12
285.0424	1	16780.4
286.0459	1	3060.13

Compound Structure

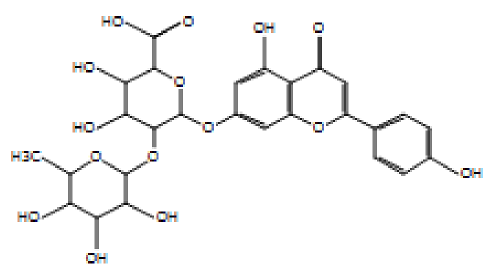

Compound Label	Name	*m/z*	RT	Algorithm	Mass
Cpd 25: Genistein 8-C-glucoside; C21 H20 O10	**Genistein 8-C-glucoside**	431.1016	8.378	Auto MS/MS	432.1092

MS Spectrum

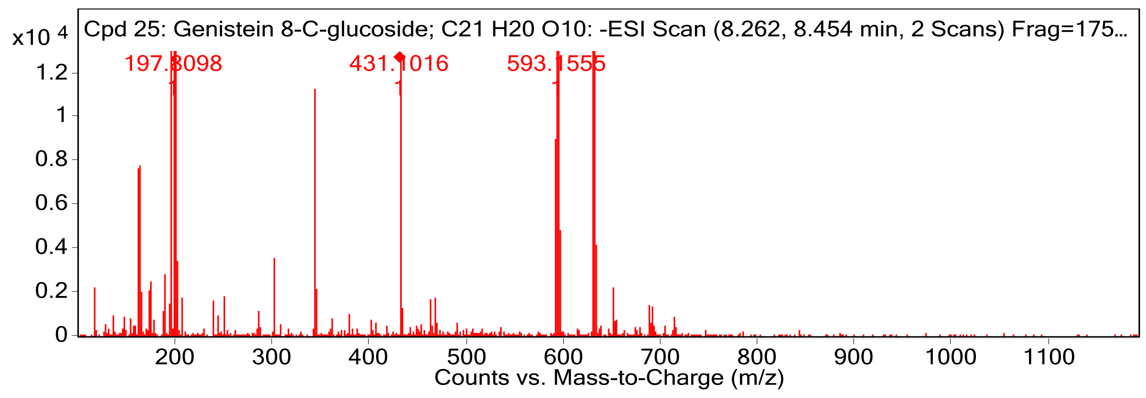

MS Zoomed Spectrum

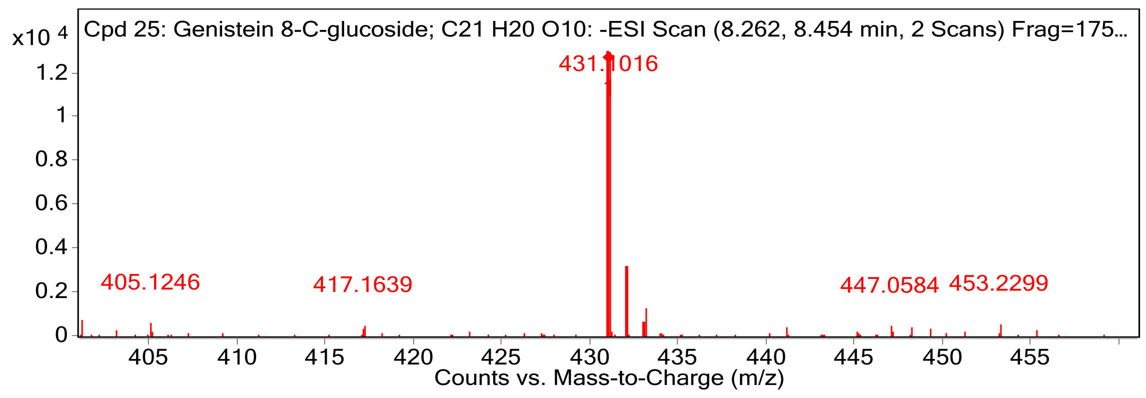

MS Spectrum Peak List

m/z	*Calc m/z*	Diff(ppm)	z	Abund	Formula	Ion
195.8126				28192.44		
197.8098			1	32228.85		
199.8068			1	14226.08		
431.1016	431.0984	-7.39	1	12973.82	C21 H20 O10	(M-H)-
432.1048	432.1018	-7.13	1	3028.02	C21 H20 O10	(M-H)-
433.1124	433.104	-19.29	1	1336.56	C21 H20 O10	(M-H)-
434.113	434.1067	-14.56	1	165.5	C21 H20 O10	(M-H)-

593.1555			1	65421.46		
594.1591			1	20038.67		
629.1319			1	38077.45		

MSMS Spectrum

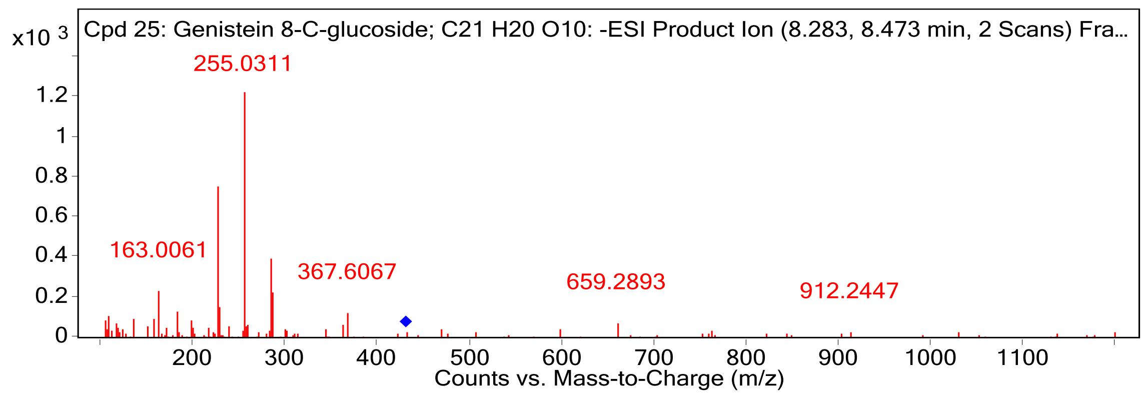

MS/MS Spectrum Peak List

m/z	z	Abund
163.0061		234.31
183.0415	1	134.1
227.0096	1	274.97
227.0377	1	754.85
228.037	1	153.41
229.0206		153.89
255.0311	1	1227.14
256.0309	1	374.1
284.0352	2	397.12
285.0413	2	229.68

Compound Structure

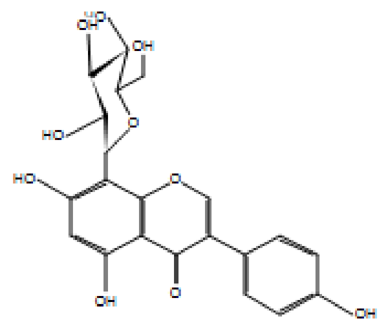

Compound Label	Name	*m/z*	RT	Algorithm	Mass
Cpd 26: CMP-N-glycoloylneuraminate; C20 H31 N4 O17 P	**CMP-N-glycoloylneuraminate**	629.132	8.461	Auto MS/MS	630.1377

MS Spectrum

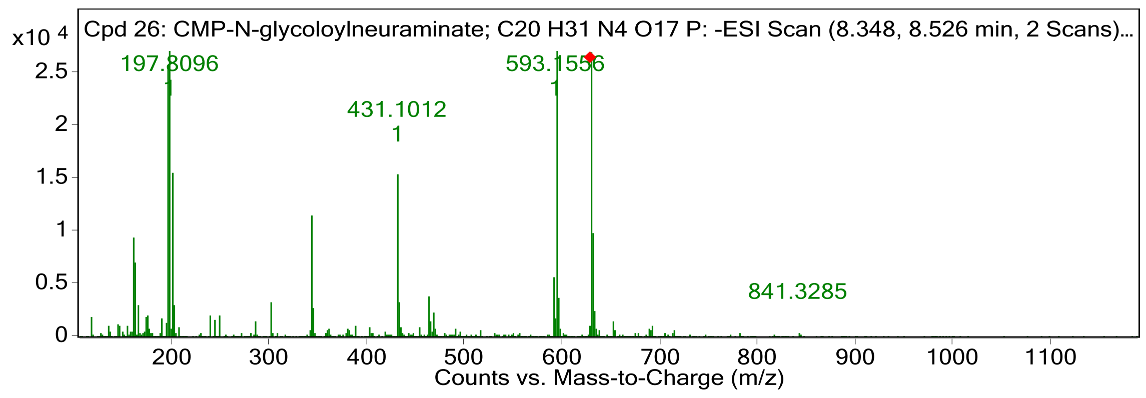

MS Zoomed Spectrum

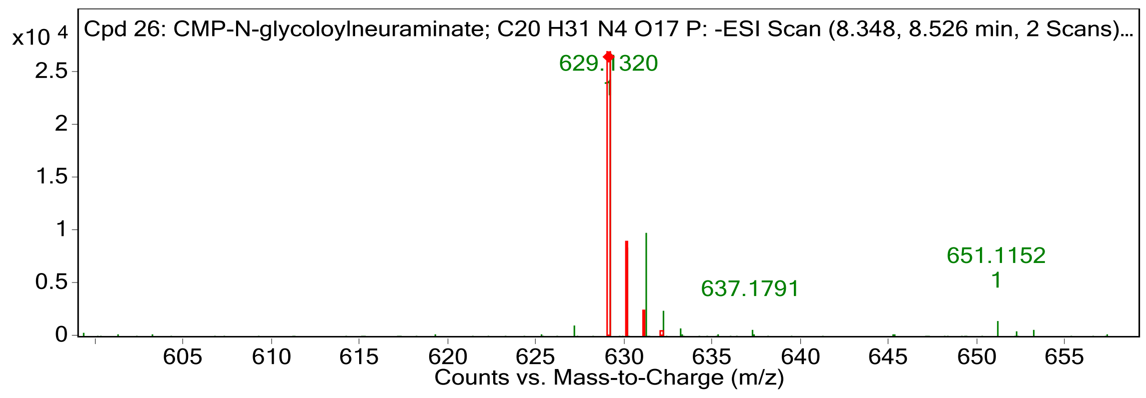

MS Spectrum Peak List

m/z	*Calc m/z*	Diff(ppm)	z	Abund	Formula	Ion
195.8128			1	25747.72		
197.8096			1	32029.9		
199.8065			1	15584.13		
431.1012			1	15402.72		
593.1556			1	47768.59		
629.132	629.1349	4.68	1	26991.68	C20 H31 N4 O17 P	(M-H)-
630.1357	630.1379	3.58	1	8484.31	C20 H31 N4 O17 P	(M-H)-
631.1309	631.14	14.4	1	9813.64	C20 H31 N4 O17 P	(M-H)-
632.1331	632.1425	14.95	1	2492.9	C20 H31 N4 O17 P	(M-H)-
633.1394	633.1447	8.35	1	770.46	C20 H31 N4 O17 P	(M-H)-

MSMS Spectrum

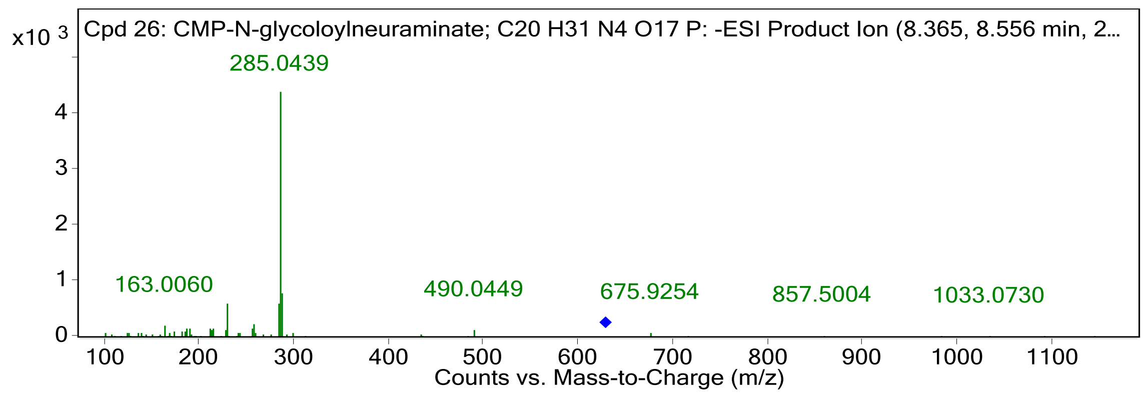

MS/MS Spectrum Peak List

m/z	z	Abund
163.006	1	210.54
187.0343		156.35
214.0273		158.79

229.0525	1	594.9
257.0147		155.55
257.0502	1	236.32
284.0327		607.32
284.9985		184.66
285.0439	1	4401.2
286.0469	1	788.19

Compound Structure

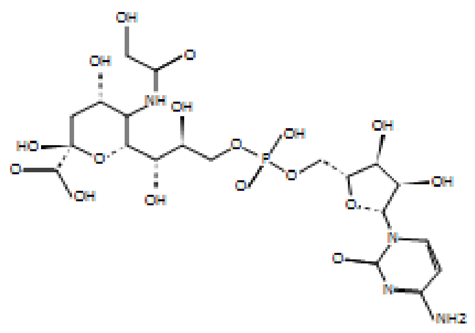

Compound Label	Name	*m/z*	RT	Algorithm	Mass
Cpd 27: Biorobin; C27 H30 O15	**Biorobin**	593.1556	8.543	Auto MS/MS	594.1627

MS Spectrum

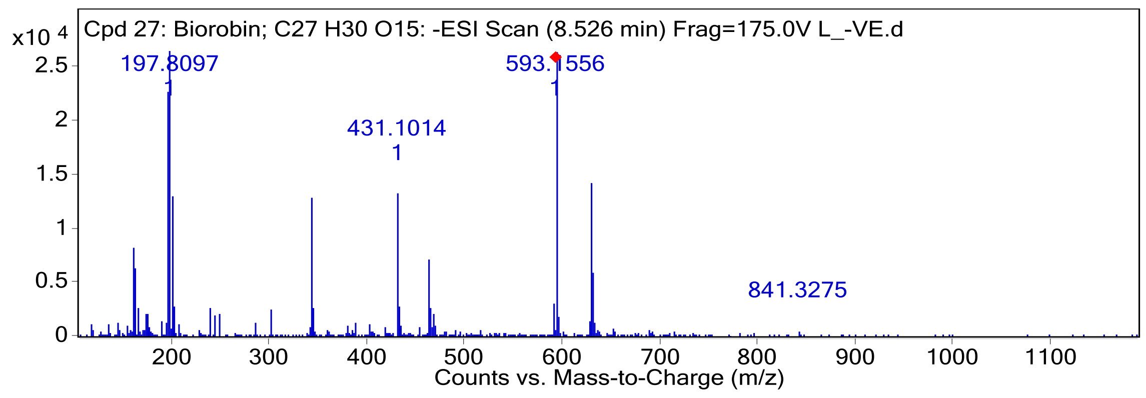

MS Zoomed Spectrum

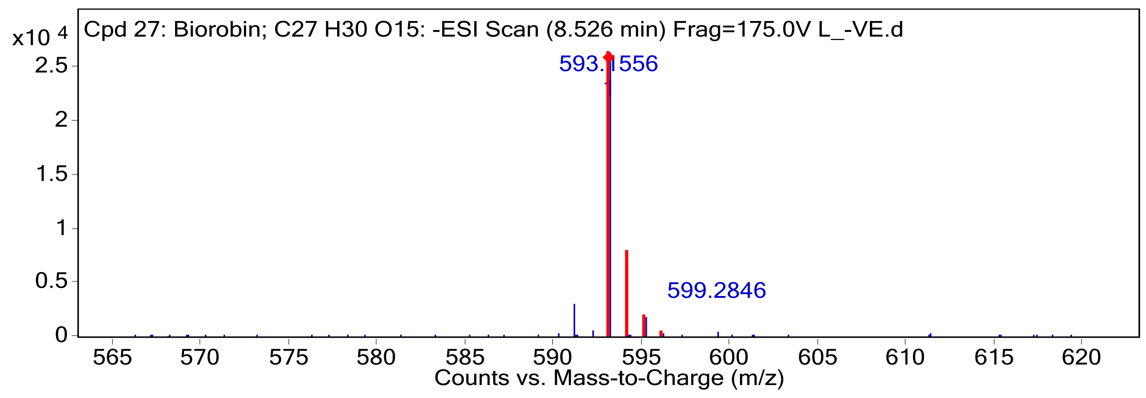

MS Spectrum Peak List

m/z	*Calc m/z*	Diff(ppm)	z	Abund	Formula	Ion
195.8127			1	22782.23		
197.8097			1	31888.38		
199.8064			1	13130.22		
343.2156			1	12961.3		
431.1014			1	13337.36		
593.1556	593.1512	-7.38	1	26422.77	C27 H30 O15	(M-H)-
594.1585	594.1546	-6.62	1	7546.19	C27 H30 O15	(M-H)-
595.1596	595.1569	-4.38	1	1967.85	C27 H30 O15	(M-H)-
596.1663	596.1596	-11.26	1	442.01	C27 H30 O15	(M-H)-
629.1304			1	14286.48		

MSMS Spectrum

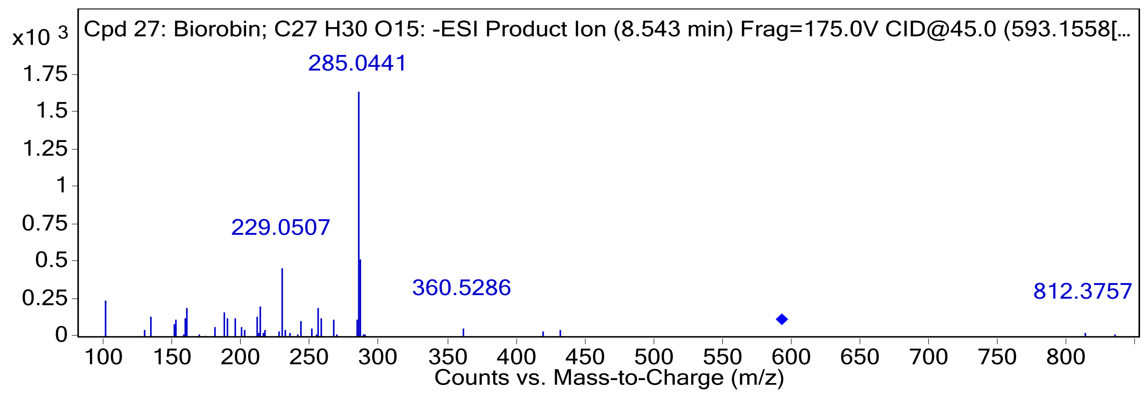

MS/MS Spectrum Peak List

m/z	z	Abund
101.0265		247.93
160.0288		198.97
187.0424		168.23
211.0437		138.31
213.0458		201.91
229.0507		460.33
255.0061		201.12
285.0016		226.33
285.0441	1	1642.7
286.045	1	518.63

Compound Structure

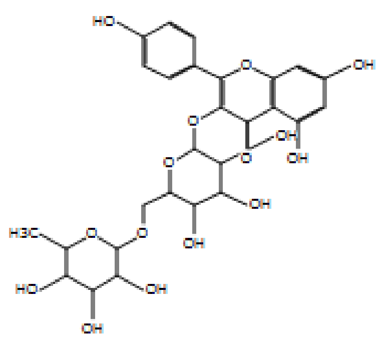

Compound Label	Name	*m/z*	RT	Algorithm	Mass
Cpd 28: Glafenine; C19 H17 Cl N2 O4	**Glafenine**	431.1023	8.66	Auto MS/MS	372.0889

MS Spectrum

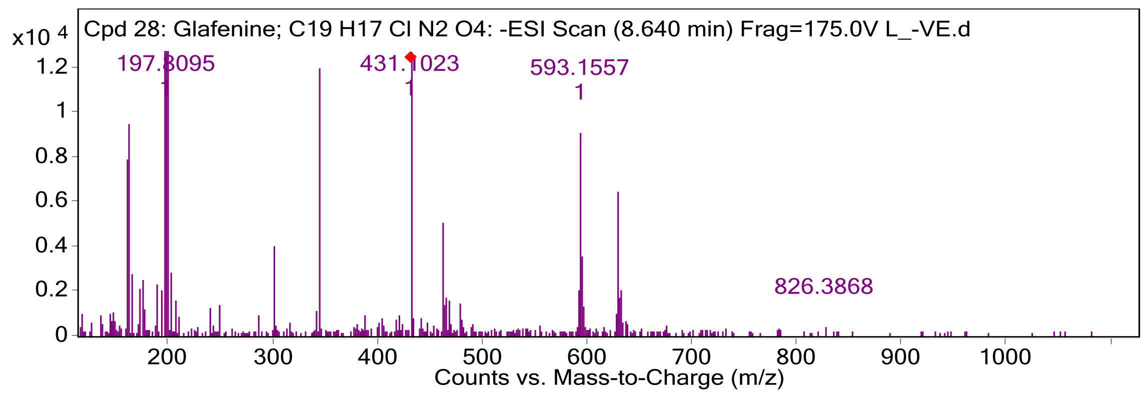

MS Zoomed Spectrum

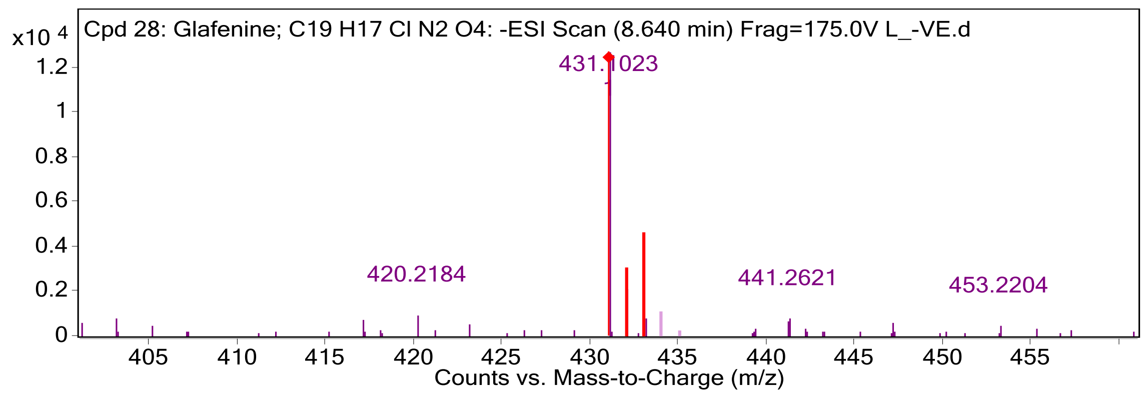

MS Spectrum Peak List

m/z	*Calc m/z*	Diff(ppm)	z	Abund	Formula	Ion
160.8428				7893.48		
162.8409				9502.07		
195.8125			1	25676.25		
197.8095			1	31810.9		
199.8068			1	14804.28		
343.214			1	11981.47		
431.1023	431.1015	-1.82	1	12693.95	C19 H17 Cl N2 O4	(M+CH3COO)-
432.1027	432.1047	4.74	1	2186.33	C19 H17 Cl N2 O4	(M+CH3COO)-
433.1161	433.0995	-38.12	1	855.65	C19 H17 Cl N2 O4	(M+CH3COO)-
593.1557			1	9110.8		

MSMS Spectrum

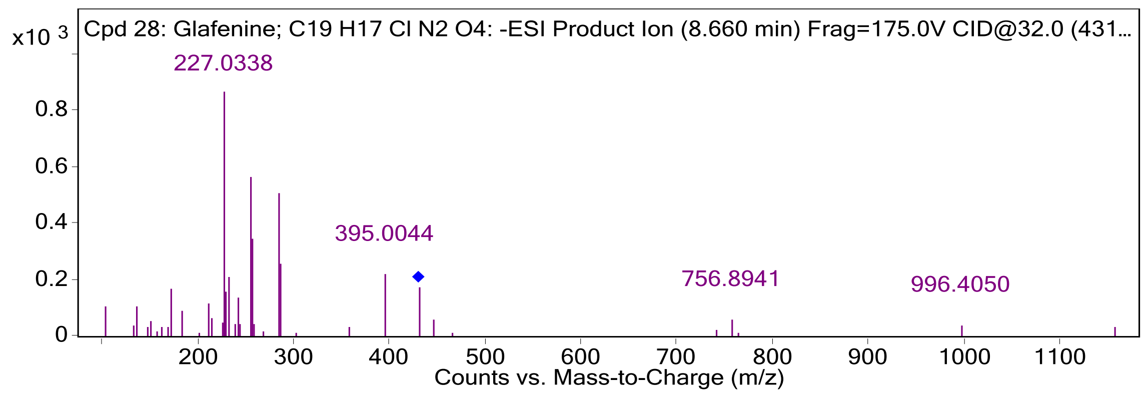

MS/MS Spectrum Peak List

m/z	z	Abund
172.0167		171.68
227.0338	1	870.62
231.0635		214.87
254.999	1	252.59
255.0309		565.02
256.0346		347.16
284.038		508.67
285.0415		262.16
395.0044		222.38
431.2348		178.29

Compound Structure

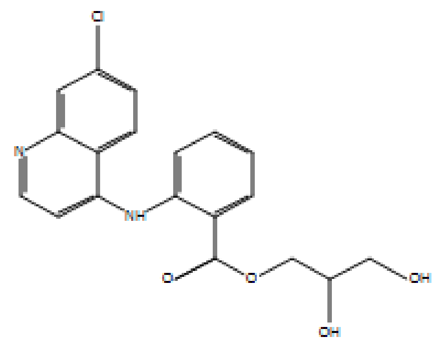

Compound Label	Name	*m/z*	RT	Algorithm	Mass
Cpd 29: Tetradecyl sulfate; C14 H30 O4 S	**Tetradecyl sulfate**	293.1783	13.01	Auto MS/MS	294.1858

MS Spectrum

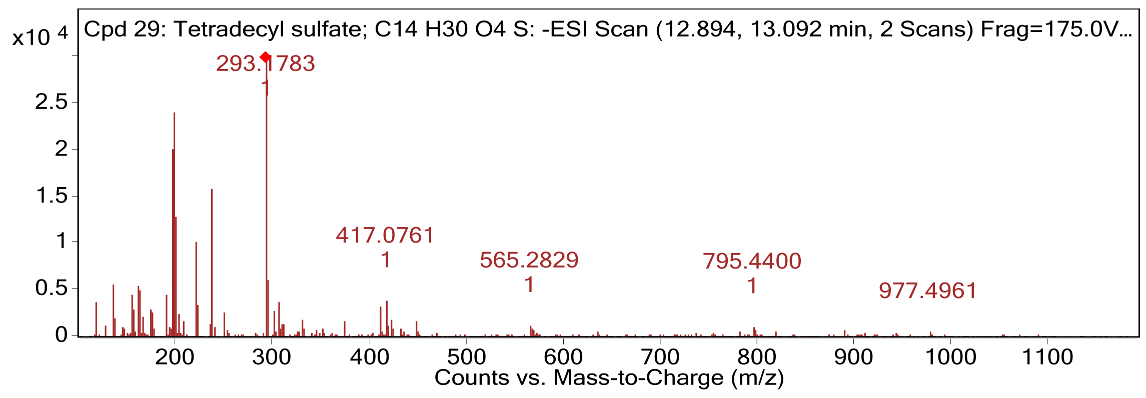

MS Zoomed Spectrum

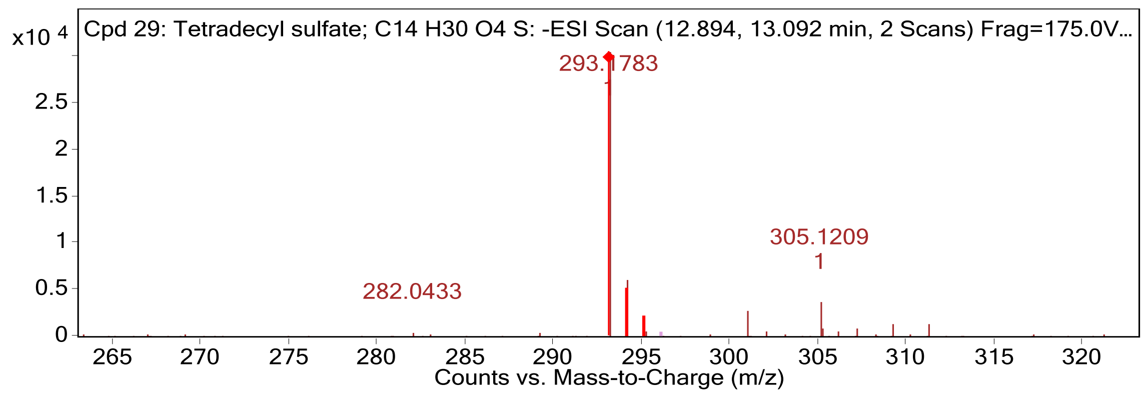

MS Spectrum Peak List

m/z	*Calc m/z*	Diff(ppm)	z	Abund	Formula	Ion
134.896			1	5653.19		
160.8434				5548.62		
195.8128			1	20074.15		
197.8098			1	24079.56		
199.8067			1	12818.22		
220.1491			1	10255.82		
236.1076			1	15922.76		
293.1783	293.1792	2.98	1	30490.9	C14 H30 O4 S	(M-H)-
294.182	294.1824	1.47	1	6091.44	C14 H30 O4 S	(M-H)-
295.1843	295.1781	-20.83	1	582.85	C14 H30 O4 S	(M-H)-

MSMS Spectrum

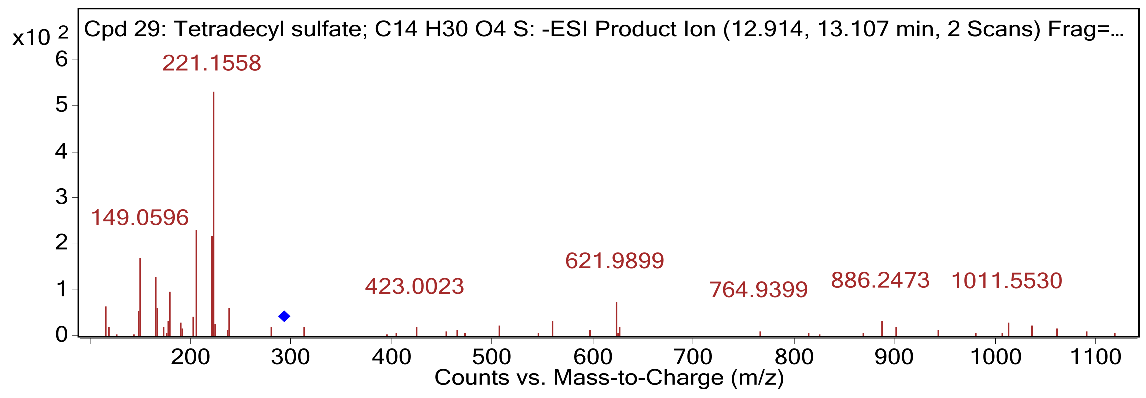

MS/MS Spectrum Peak List

m/z	z	Abund
114.9024		68.01
148.0531		132.83
149.0596		170.61
164.0842		131.27
178.0987		99.13
205.1208		232.87
220.1509		219.22
221.1558	1	533.33
222.1511	1	132.81
621.9899		76.56

Compound Structure

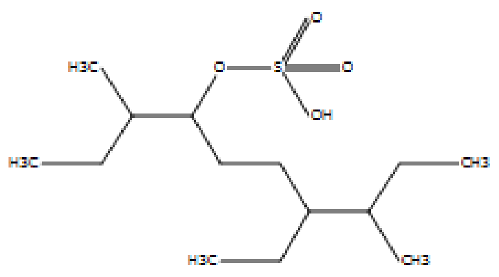

Compound Label	Name	*m/z*	RT	Algorithm	Mass
Cpd 30: Hexazinone; C12 H20 N4 O2	**Hexazinone**	297.1559	18.039	Auto MS/MS	252.1576

MS Spectrum

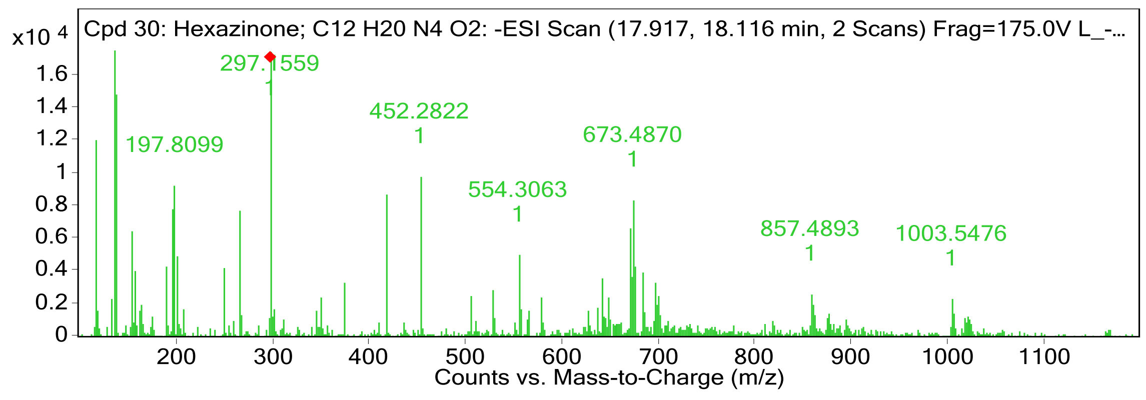

MS Zoomed Spectrum

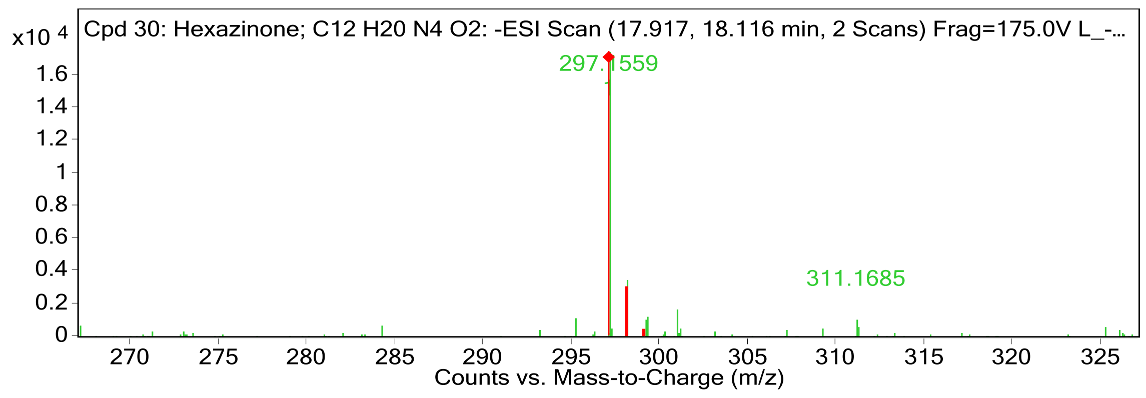

MS Spectrum Peak List

m/z	*Calc m/z*	Diff(ppm)	z	Abund	Formula	Ion
116.9301			1	12019.67		
134.8964			1	44937.31		
136.8937			1	14813.91		
197.8099				9264.05		
297.1559	297.1568	2.97	1	17399.51	C12 H20 N4 O2	(M+HCOO)-
298.1592	298.1597	1.47	1	3488.83	C12 H20 N4 O2	(M+HCOO)-
299.1569	299.1619	16.56	1	1085.84	C12 H20 N4 O2	(M+HCOO)-
417.0767			1	8707.31		

452.2822			1	9807.24		
673.487			1	8371.28		

MSMS Spectrum

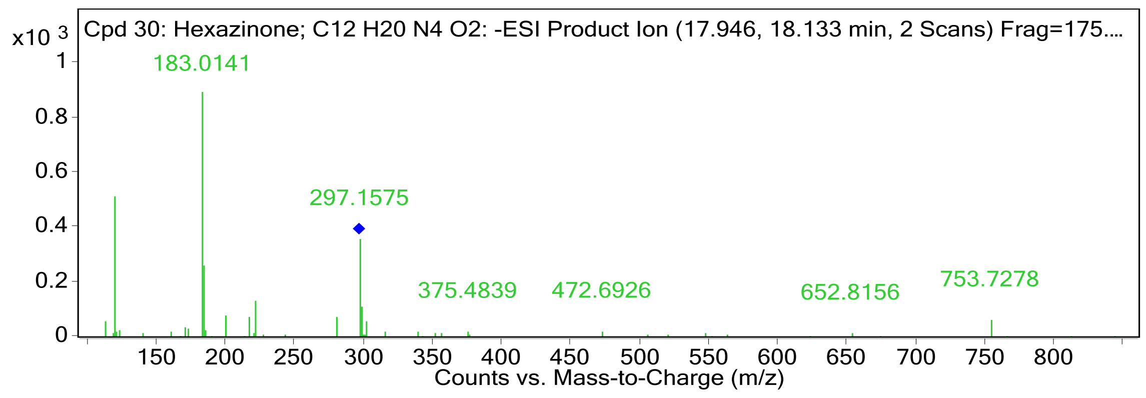

MS/MS Spectrum Peak List

m/z	z	Abund
119.0318		90.82
119.0515	2	512.96
120.0558	2	251.02
183.0141	1	896.48
183.9905		77.75
184.0221	1	262.49
200.0125		80.19
221.1584		135.13
297.1575	1	360.57
298.1572	1	113.06

Compound Structure

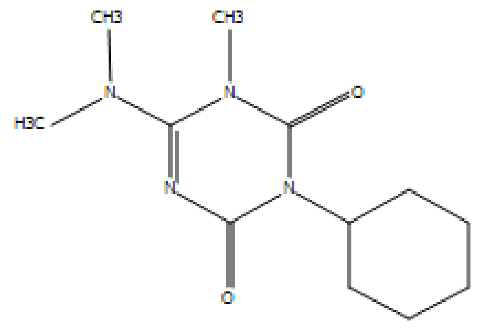

Compound Label	m/z	RT	Algorithm
Compound 31	530.3066	19.193	Auto MS/MS

MS Spectrum

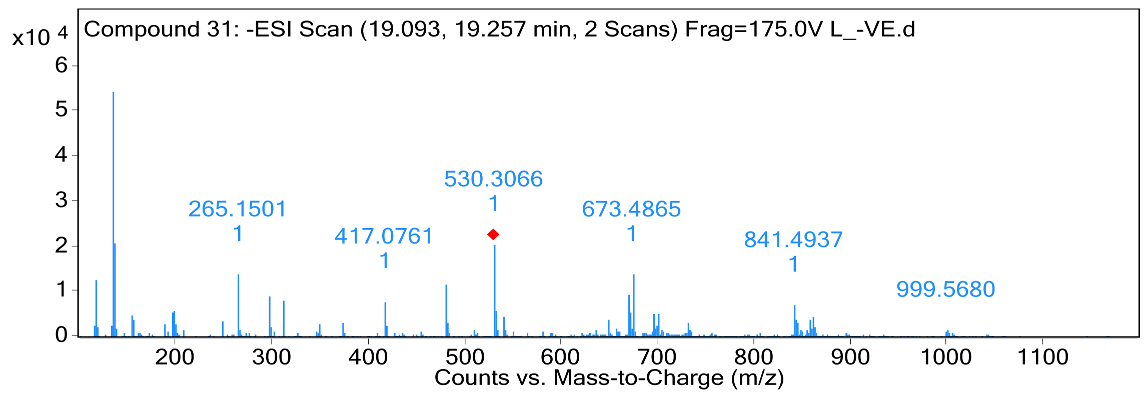

MS Zoomed Spectrum

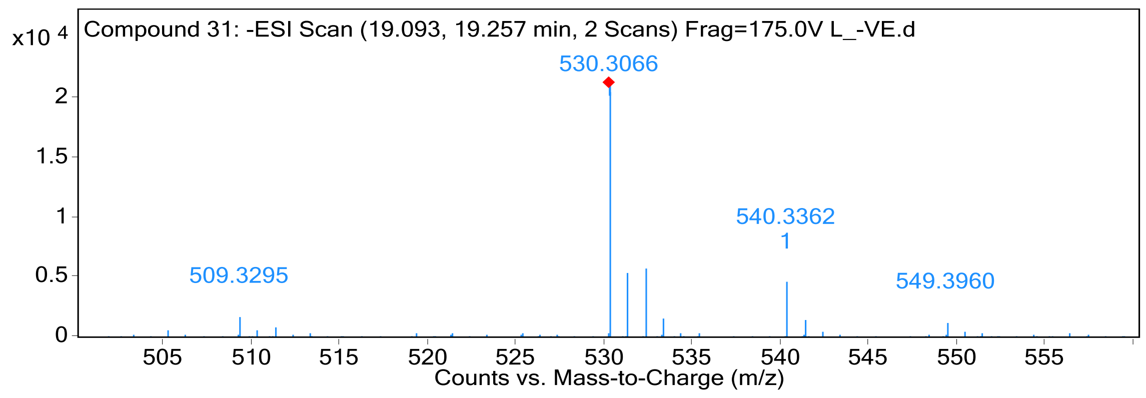

MS Spectrum Peak List

m/z	z	Abund
116.9299	1	12701.59
134.8963	1	54404.23
136.8937	1	20765.34
265.1501	1	13912.61
530.3066	1	20562.43
531.3112	1	5391.74
532.3033	1	5727.32
533.3074	1	1644.78
534.3091	1	335.15
673.4865	1	13882.47

MSMS Spectrum

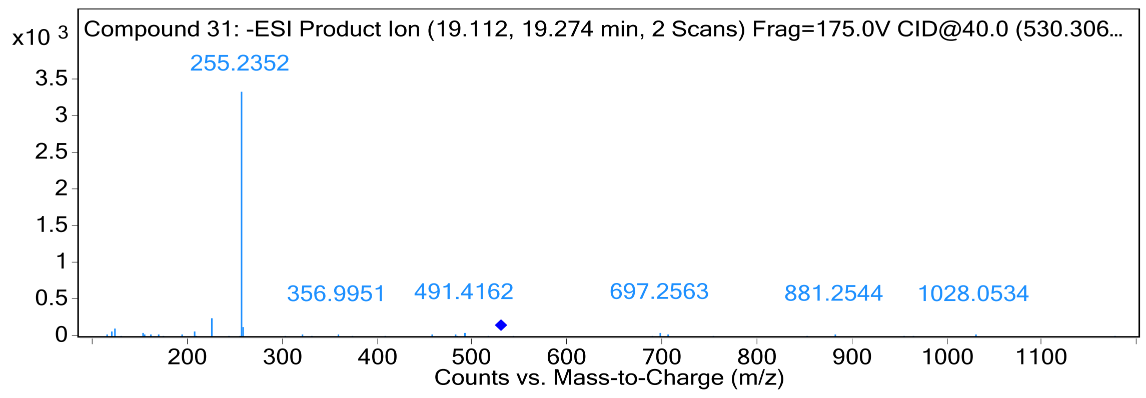

MS/MS Spectrum Peak List

m/z	z	Abund
118.9956		88.33
123.288		129.58
207.1009		83.9
224.0715		266.36
255.2085	1	302.25
255.2352	1	3349.96
256.2167	1	92.95
256.2407	1	662.82

257.2309	1	73.58
258.2452		137.45

Compound Label	*m/z*	RT	Algorithm
Compound 32	530.3069	19.49	Auto MS/MS

MS Spectrum

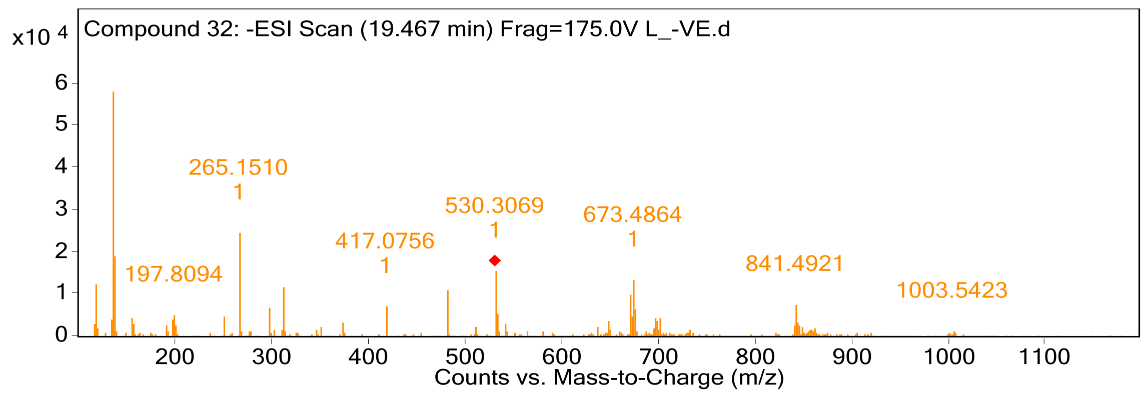

MS Zoomed Spectrum

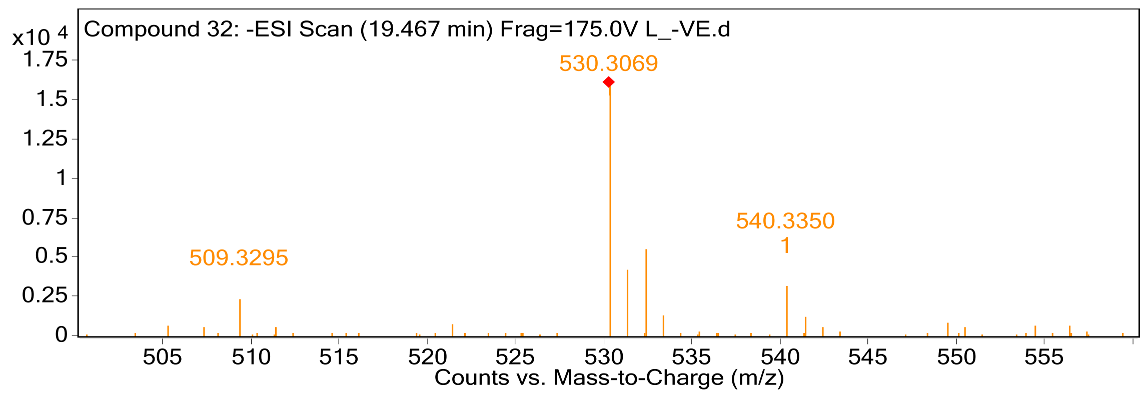

MS Spectrum Peak List

m/z	z	Abund
116.9297	1	12432.29
134.8963	1	58184.21
136.8935	1	19072.5
265.151	1	24685.67
530.3069	1	15622.62
531.3099	1	4294.96
532.3044	1	5573.74
533.3097	1	1435.14
534.3133	1	285.24
673.4864	1	13487.23

MSMS Spectrum

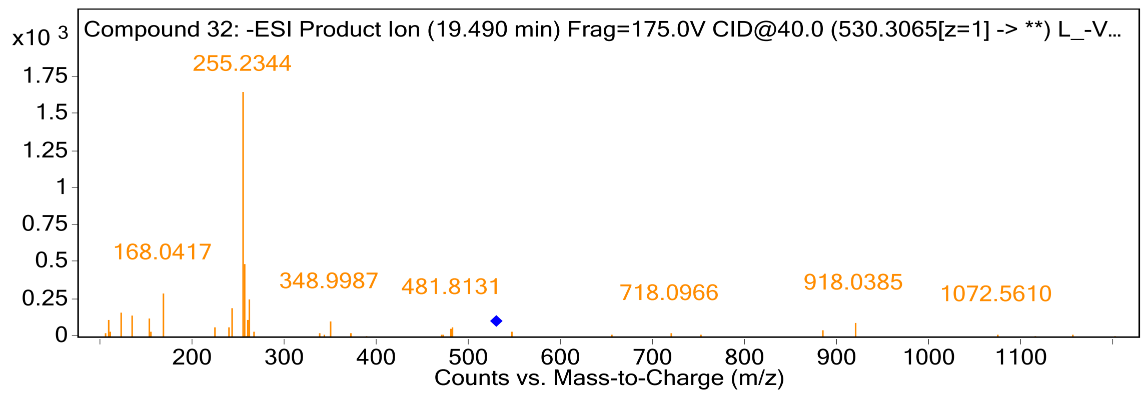

MS/MS Spectrum Peak List

m/z	z	Abund
122.9893		166.34
134.0282		145.34
152.9954		124.91
168.0417		299.5
242.0682		198.74
242.0848		154.2
255.2344	1	1653.37
256.2401	1	490.48
259.4157		121.19
261.7654		258.02

Compound Label	Name	*m/z*	RT	Algorithm	Mass
Cpd 33: Hexazinone; C12 H20 N4 O2	**Hexazinone**	311.1716	19.915	Auto MS/MS	252.1576

MS Spectrum

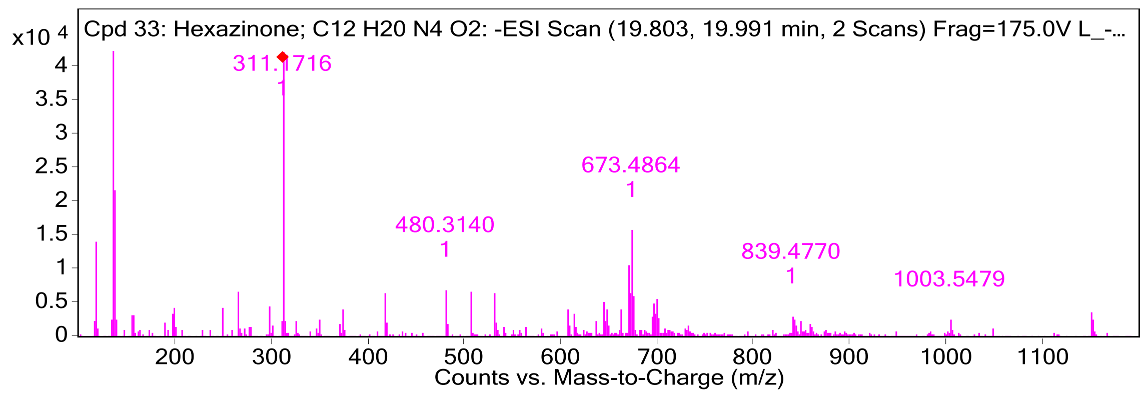

MS Zoomed Spectrum

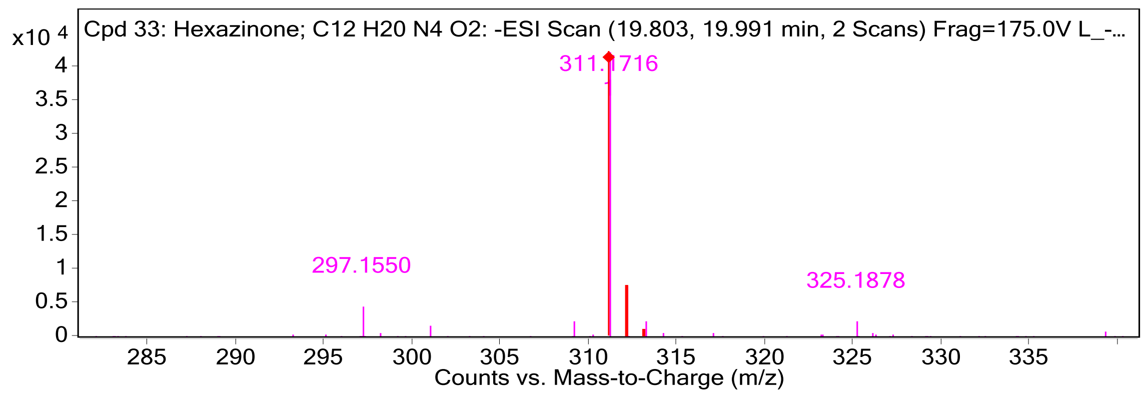

MS Spectrum Peak List

m/z	*Calc m/z*	Diff(ppm)	z	Abund	Formula	Ion
116.9297			1	13998.8		
134.8965			1	58282.32		
136.8935			1	21589.67		
311.1716	311.1725	2.72	1	42075.74	C12 H20 N4 O2	(M+CH3COO)-
312.175	312.1753	1.12	1	7573.39	C12 H20 N4 O2	(M+CH3COO)-
313.1703	313.1776	23.5	1	2388.13	C12 H20 N4 O2	(M+CH3COO)-
480.314			1	6911.74		
505.2612			1	6655.27		
669.455			1	10660.69		
673.4864			1	15762.09		

MSMS Spectrum

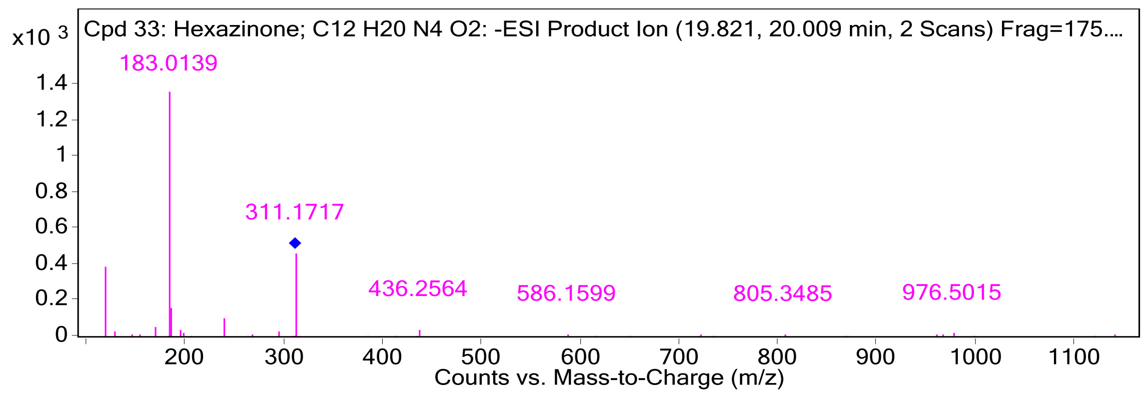

MS/MS Spectrum Peak List

m/z	z	Abund
119.051		391.69
170.0058		56.9
183.0139	1	1363.42
184.0132	1	371.55
184.9998		40.17
185.0124	1	164.16
239.0759		103.5
311.1717	1	467.72
312.1754	1	130.04
436.2564		41.44

Compound Structure

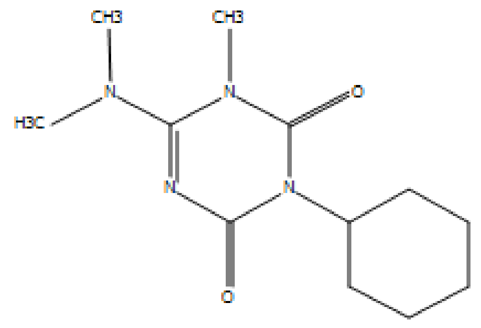

Compound Label	*m/z*	RT	Algorithm
Compound 34	530.3062	20.072	Auto MS/MS

MS Spectrum

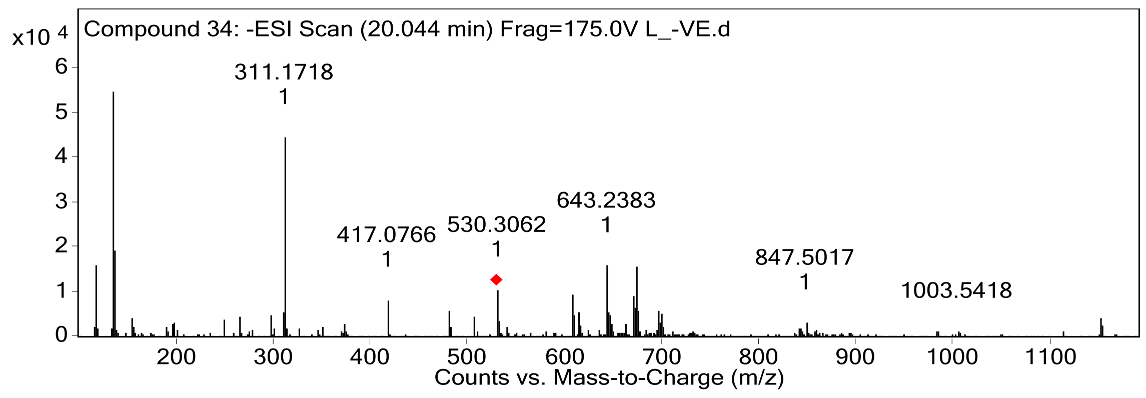

MS Zoomed Spectrum

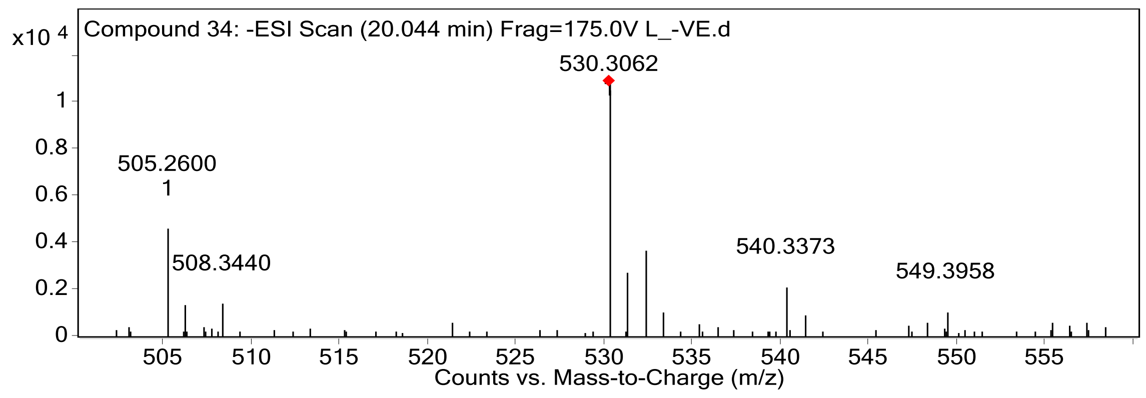

MS Spectrum Peak List

m/z	z	Abund
116.9302	1	15926.94
134.8962	1	54879.04
136.8935	1	19454.68
311.1718	1	44646.52
530.3062	1	10505.21
531.3083	1	2771.39
532.3026	1	3671.29
533.3062	1	1048.33
534.3015	1	226.22
643.2383	1	15937.13

MSMS Spectrum

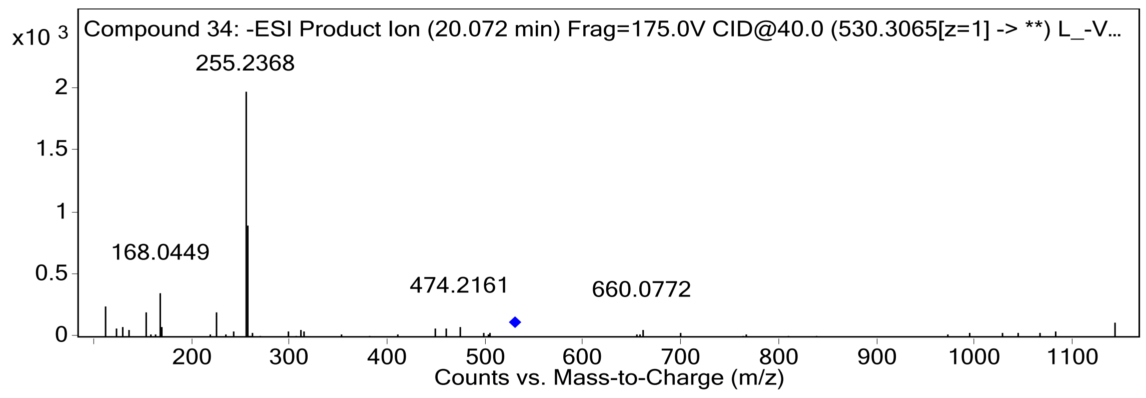

MS/MS Spectrum Peak List

m/z	z	Abund
111.7117		247.94
129.0374		86.96
152.9992		203.41
168.0449	1	354.02
224.0693		205.8
255.2368		1979.98
256.2369	1	900.21
257.2343	1	118.63
474.2161		87.56
1141.3076		118.84

Compound Label	Name	*m/z*	RT	Algorithm	Mass
Cpd 35: Magnesium protoporphyrin monomethyl ester; C35 H34 Mg N4 O4	**Magnesium protoporphyrin monomethyl ester**	643.2383	20.121	Auto MS/MS	598.2395

MS Spectrum

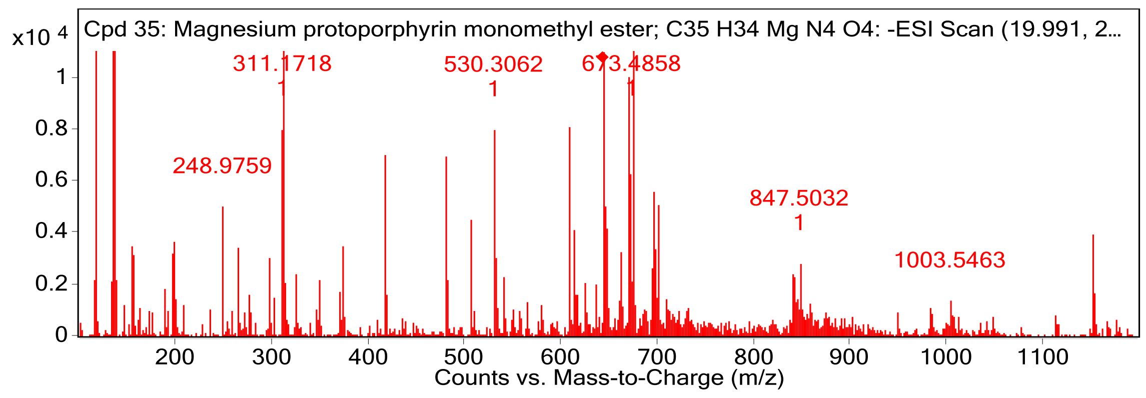

MS Zoomed Spectrum

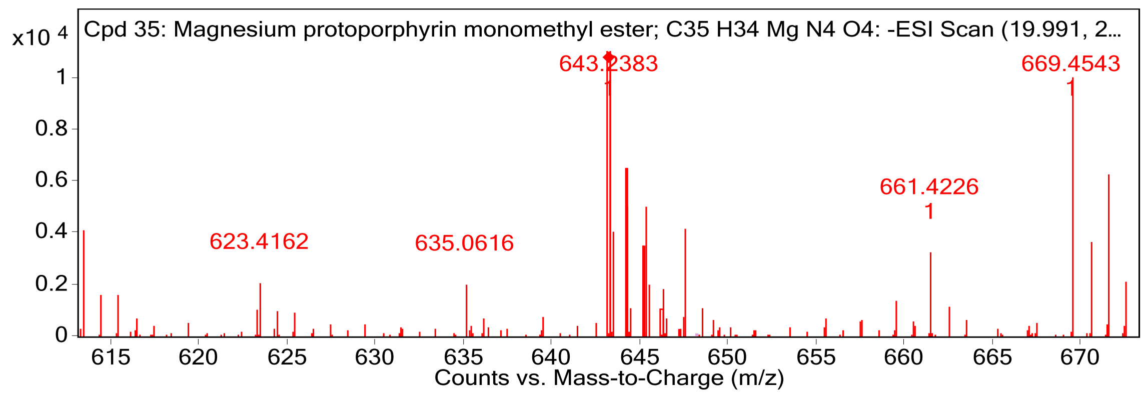

MS Spectrum Peak List

m/z	*Calc m/z*	Diff(ppm)	z	Abund	Formula	Ion
116.9299			1	13511.69		
134.8963			1	54449.67		
136.8933			1	19310.45		
311.1718			1	41644.15		
643.2383	643.2413	4.54	1	11004.22	C35 H34 Mg N4 O4	(M+HCOO)-
644.2418	644.2439	3.25	1	5022.35	C35 H34 Mg N4 O4	(M+HCOO)-
645.2372	645.2428	8.7	1	5064.39	C35 H34 Mg N4 O4	(M+HCOO)-
646.2387	646.2443	8.76	1	1846.78	C35 H34 Mg N4 O4	(M+HCOO)-
647.2398	647.2465	10.35	1	328.86	C35 H34 Mg N4 O4	(M+HCOO)-
673.4858			1	14759.6		

MSMS Spectrum

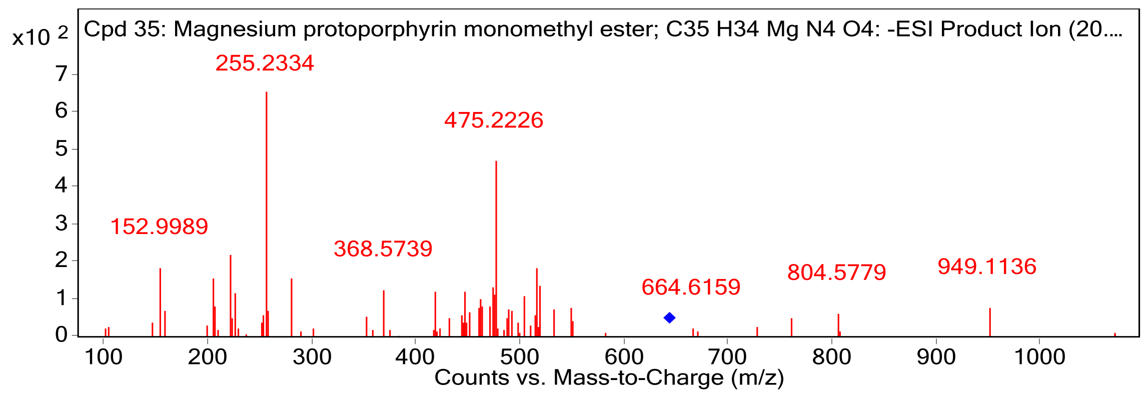

MSMS Spectrum

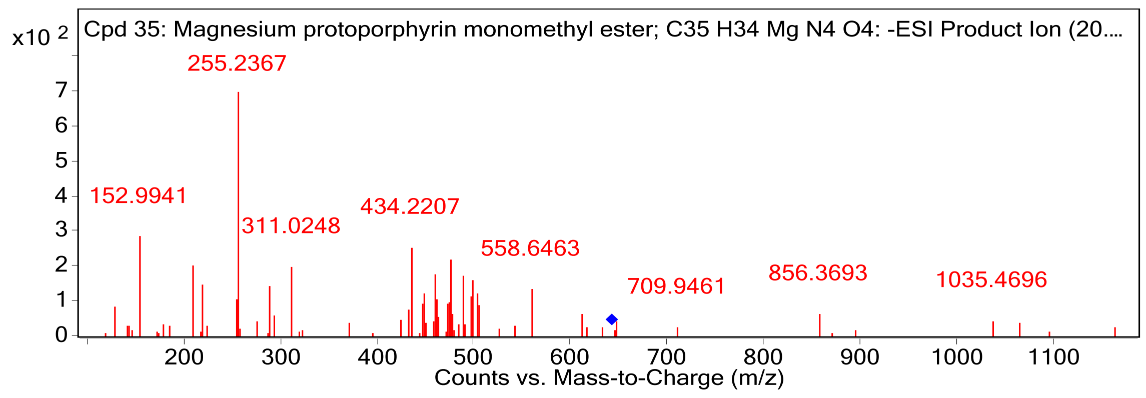

MS/MS Spectrum Peak List

m/z	z	Abund
152.9989		186.33
205.0236	2	156.23
221.0952		219.13
255.2334	1	658.14
278.9198		157.61
368.5739		126.54

473.2308		133.38
475.2226	1	472
514.2335		183.85
518.2493		138.62
152.9941		288
153.9886		241.88
208.6327		203.97
255.2367		702.26
311.0248		201.84
434.2207		257.13
459.2485		181.77
475.2169		221.06
487.2553		177.22
498.2		165.63

Compound Structure

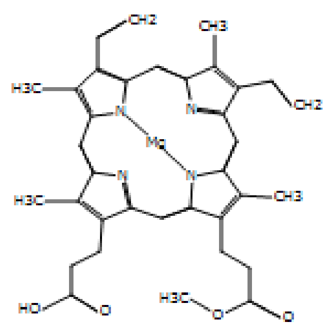

Compound Label	Name	*m/z*	RT	Algorithm	Mass
Cpd 36: Lamprolobine; C15 H24 N2 O2	**Lamprolobine**	309.177	20.351	Auto MS/MS	264.1787

MS Spectrum

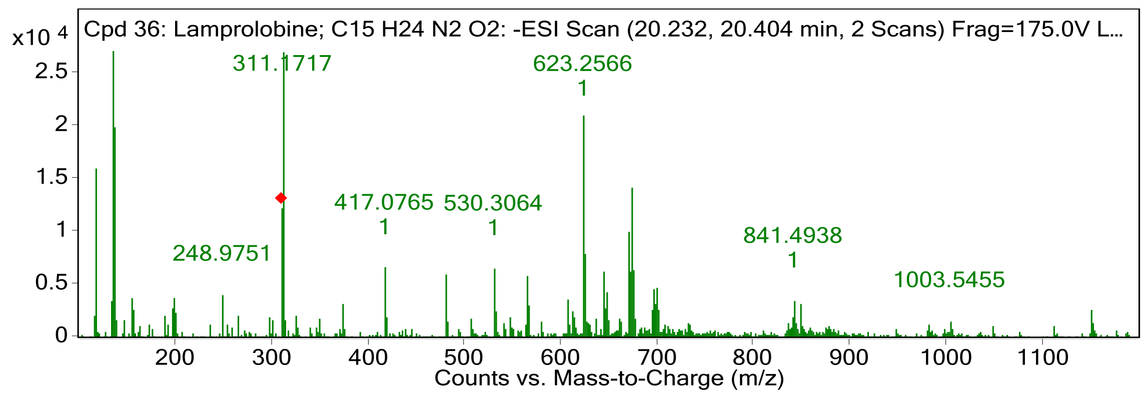

MS Zoomed Spectrum

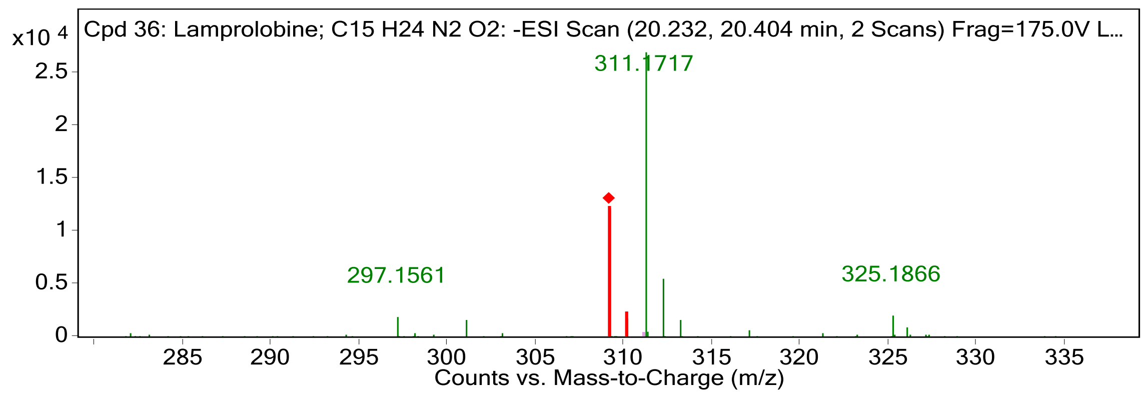

MS Spectrum Peak List

m/z	*Calc m/z*	Diff(ppm)	z	Abund	Formula	Ion
116.9298			1	15990.98		
134.8963			1	52527.34		
136.8936			1	19920.56		
309.177	309.182	16	1	12239.6	C15 H24 N2 O2	(M+HCOO)-
310.1795	310.1851	18.11	1	1790.13	C15 H24 N2 O2	(M+HCOO)-
311.1717				26948.15		
623.2566			1	20916.29		
624.2596			1	7852.98		
669.4545			1	9948.42		
673.4862			1	14193.05		

MSMS Spectrum

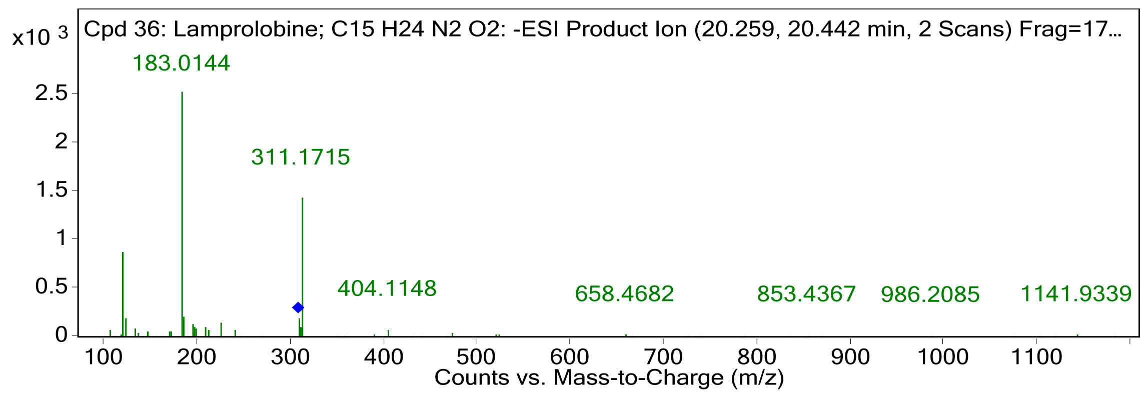

MS/MS Spectrum Peak List

m/z	z	Abund
119.052	1	872.71
122.978		204.09
183.0144	1	2535.99
184.0196	1	205.39
185.0337		115.38
195.016	1	138.53
197.0329		108.07
225.0591		147.4
309.1726		196.98
311.1715	1	1438.87

Compound Structure

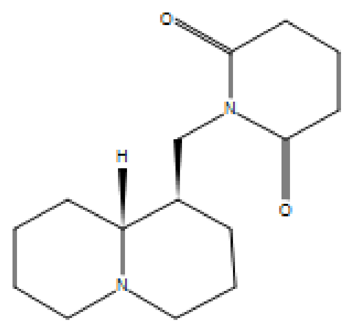

Compound Label	Name	*m/z*	RT	Algorithm	Mass
Cpd 37: Kanokoside D; C27 H44 O16	**Kanokoside D**	623.257	20.386	Auto MS/MS	624.2638

MS Spectrum

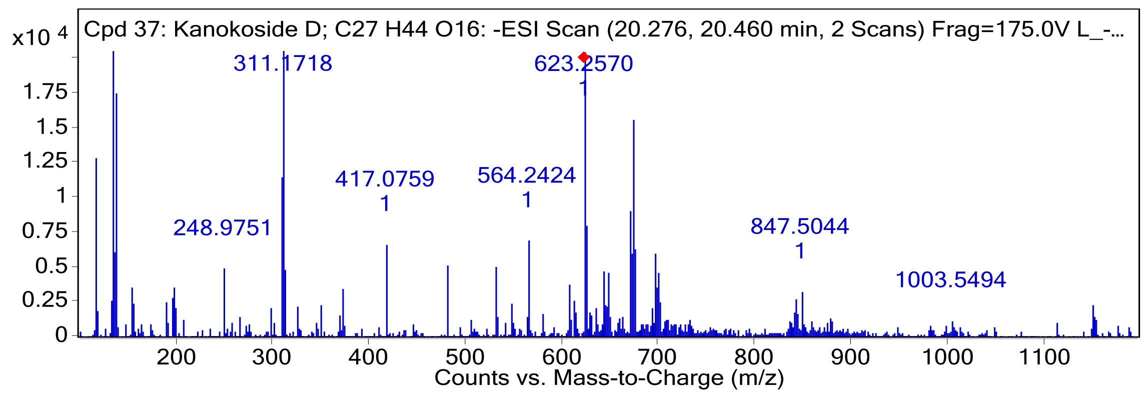

MS Zoomed Spectrum

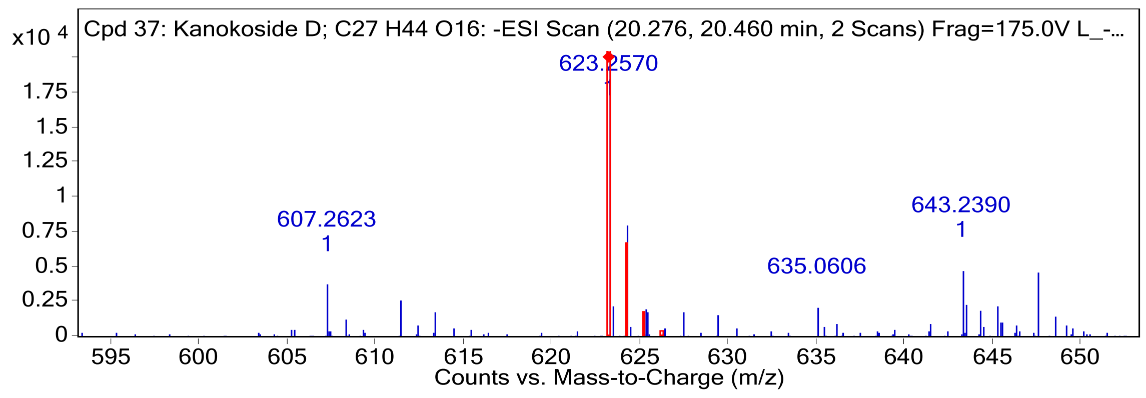

MS Spectrum Peak List

m/z	*Calc m/z*	Diff(ppm)	z	Abund	Formula	Ion
116.93			1	12830.55		
134.8964			1	55252.09		
136.8935			1	17526.79		
309.1769			1	11435.71		
311.1718				24812.04		
623.257	623.2557	-2.13	1	20434.3	C27 H44 O16	(M-H)-
624.2594	624.2591	-0.44	1	7999.91	C27 H44 O16	(M-H)-
625.26	625.2614	2.31	1	1971.49	C27 H44 O16	(M-H)-
626.2704	626.2641	-10	1	270.01	C27 H44 O16	(M-H)-
673.486			1	15614.41		

MSMS Spectrum

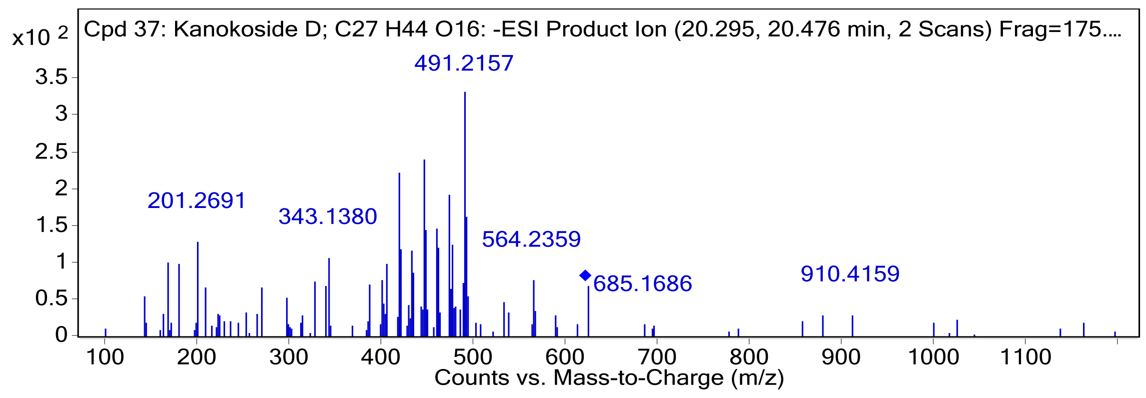

MS/MS Spectrum Peak List

m/z	z	Abund
201.2691		129.94
419.2278	1	222.49
447.2452		241.8
448.2652	1	145.38
459.1837	1	146.65
461.2324	1	120.69
474.2067	1	193.18
477.1831		124.66
491.2157	1	333.37
492.2066	1	162.76

Compound Structure

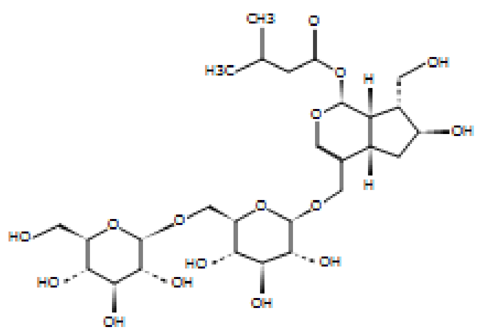

Compound Label	Name	*m/z*	RT	Algorithm	Mass
Cpd 38: 19-Hydroxycinnzeylanol 19-glucoside; C26 H42 O13	**19-Hydroxycinnzeylanol 19-glucoside**	607.2614	20.645	Auto MS/MS	562.2633

MS Spectrum

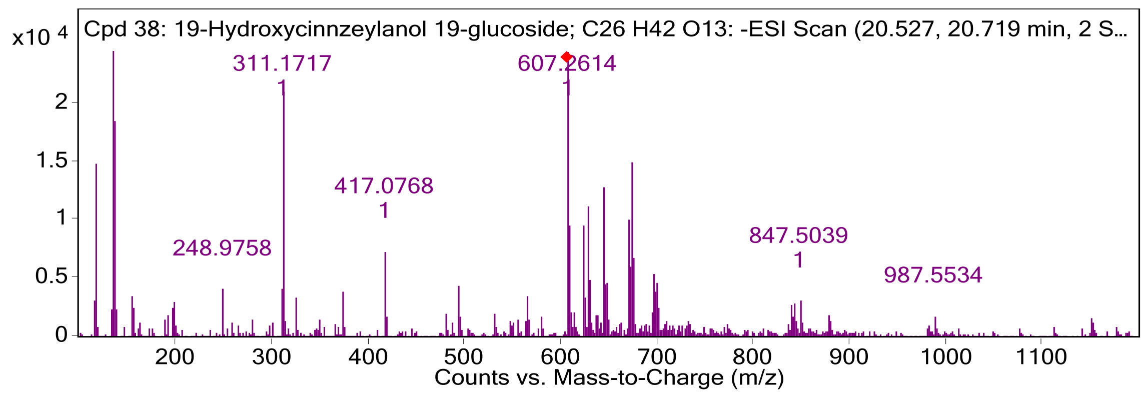

MS Zoomed Spectrum

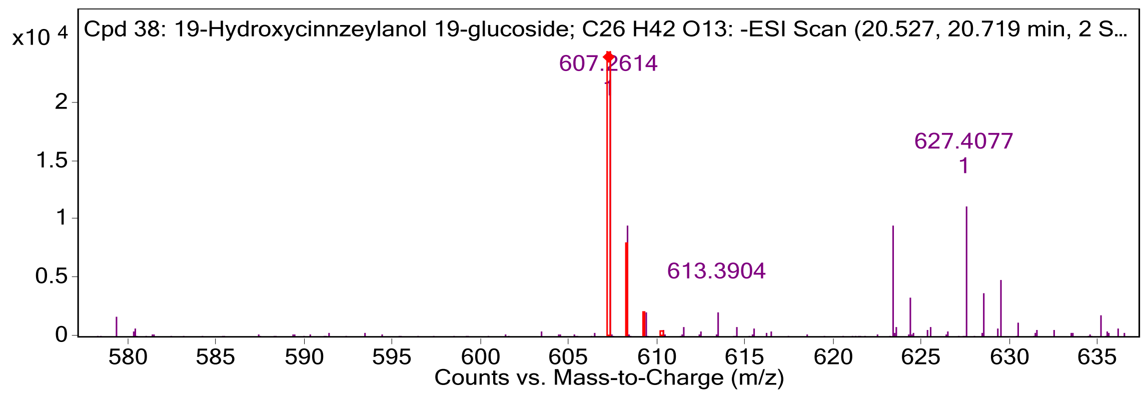

MS Spectrum Peak List

m/z	*Calc m/z*	Diff(ppm)	z	Abund	Formula	Ion
116.9302			1	14856.02		
134.8964			1	54504.54		
136.8935			1	18516.06		
311.1717			1	21015.16		
607.2614	607.2607	-1.13	1	24388.33	C26 H42 O13	(M+HCOO)-
608.2647	608.2642	-0.92	1	9534.31	C26 H42 O13	(M+HCOO)-
609.2675	609.2665	-1.68	1	2146.97	C26 H42 O13	(M+HCOO)-
610.2744	610.2692	-8.57	1	575.92	C26 H42 O13	(M+HCOO)-
643.2388			1	12861.8		
673.4861			1	14983.3		

MSMS Spectrum

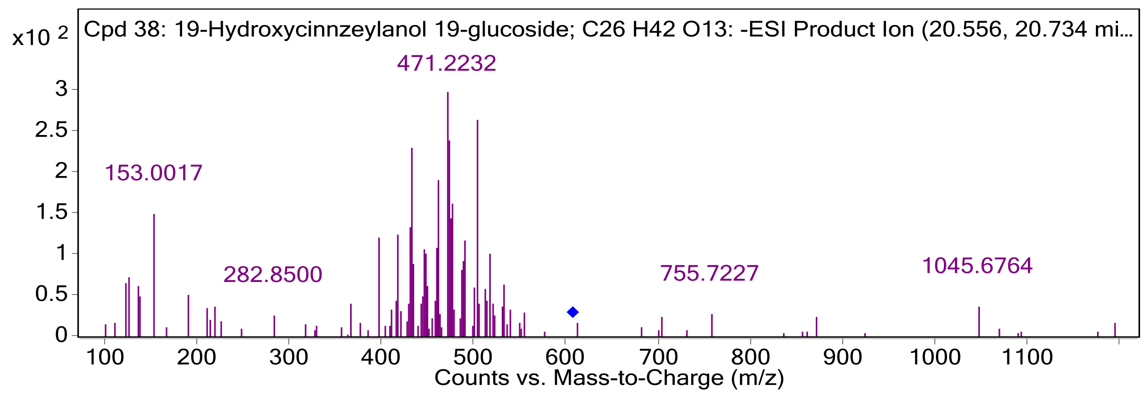

MS/MS Spectrum Peak List

m/z	z	Abund
153.0017		150.2
430.2151		134.42
432.2356	1	230.98
460.2036	1	190.84
460.2298	1	182.23
471.2232	1	298.54
472.2313		239.17
473.2312	1	143.66
475.223		162.02
502.2042	1	263.88

Compound Structure

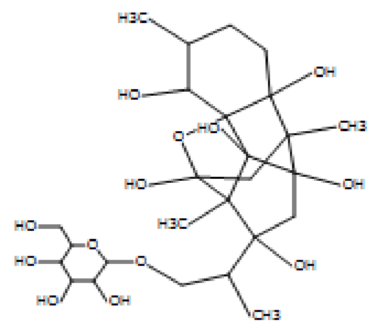

Compound Label	Name	*m/z*	RT	Algorithm	Mass
Cpd 39: Magnesium protoporphyrin monomethyl ester; C35 H34 Mg N4 O4	**Magnesium protoporphyrin monomethyl ester**	643.2384	20.74	Auto MS/MS	598.2393

MS Spectrum

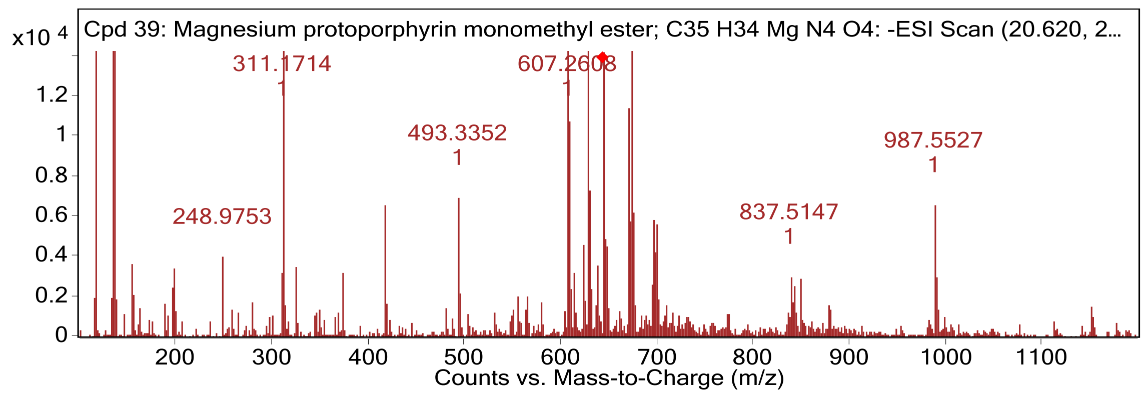

MS Zoomed Spectrum

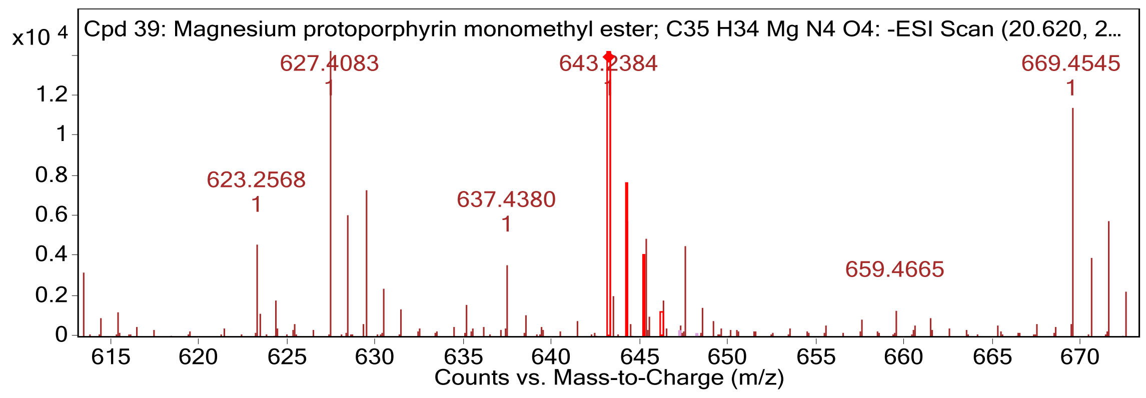

MS Spectrum Peak List

m/z	*Calc m/z*	Diff(ppm)	z	Abund	Formula	Ion
134.8964			1	53272.85		
136.8936			1	20391.29		
311.1714			1	21064.66		
607.2608			1	27816.96		
627.4083			1	17083.16		
643.2384	643.2413	4.46	1	14210.13	C35 H34 Mg N4 O4	(M+HCOO)-

644.2408	644.2439	4.82	1	5787.42	C35 H34 Mg N4 O4	(M+HCOO)-
645.2364	645.2428	9.89	1	4920.43	C35 H34 Mg N4 O4	(M+HCOO)-
646.238	646.2443	9.73	1	1822.65	C35 H34 Mg N4 O4	(M+HCOO)-
673.4859			1	14740.42		

MSMS Spectrum

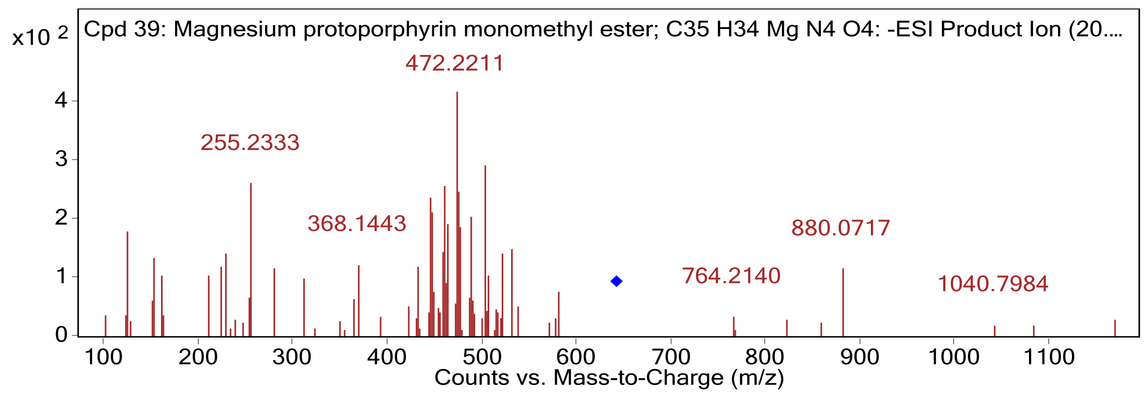

MSMS Spectrum

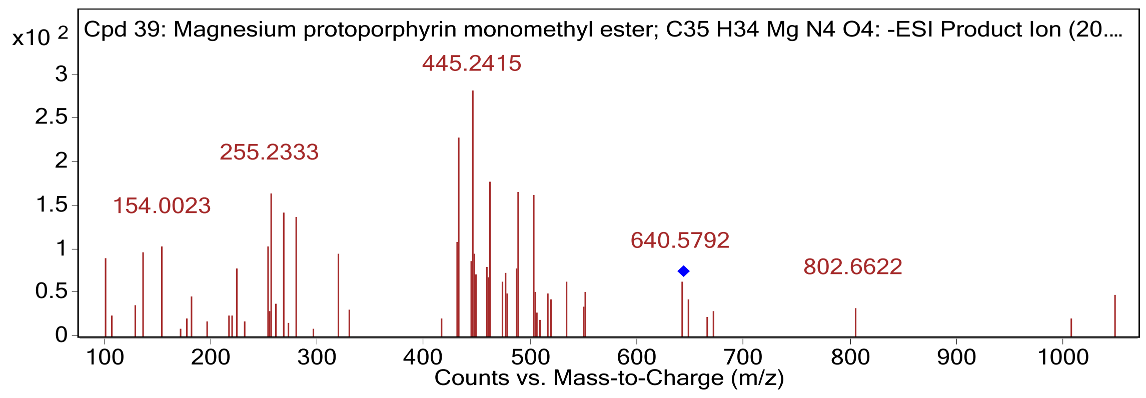

MS/MS Spectrum Peak List

m/z	z	Abund
255.2333		262.33
445.2437		237.41
446.2454	1	212.89
460.234	1	256.99
462.2099		191.66
472.2211	1	419.25
474.214	1	248.15
488.2557		206.31
502.1801		293.68
502.2056		190.55
255.2333		165.59
267.1161		143.79
279.2328		137.5
430.216		110
431.228	1	229.33
445.2415	1	282.01
461.1475		178.83
461.1899		109.88
487.2568		166.6
502.1995		162.69

Compound Structure

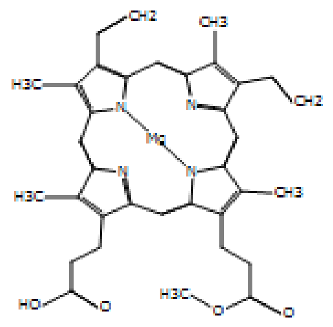

Compound Label	Name	*m/z*	RT	Algorithm	Mass
Cpd 40: Hexazinone; C12 H20 N4 O2	**Hexazinone**	311.1715	21.05	Auto MS/MS	252.1575

MS Spectrum

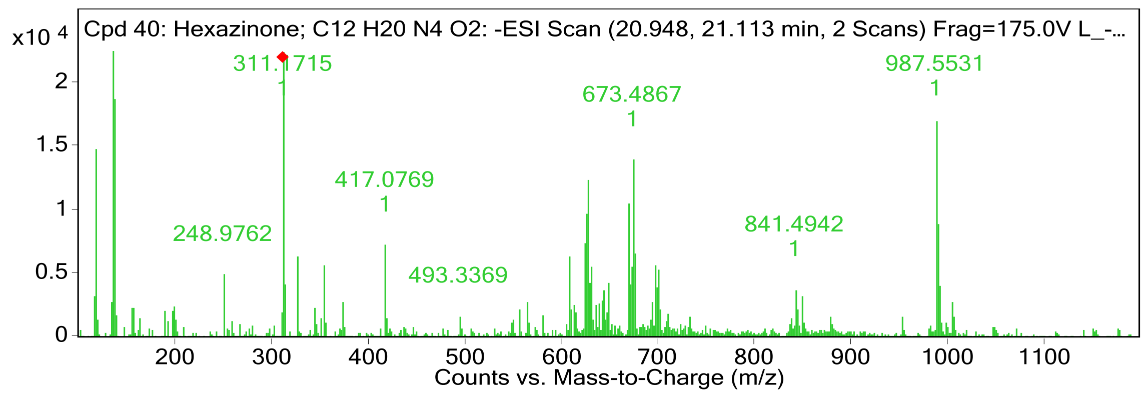

MS Zoomed Spectrum

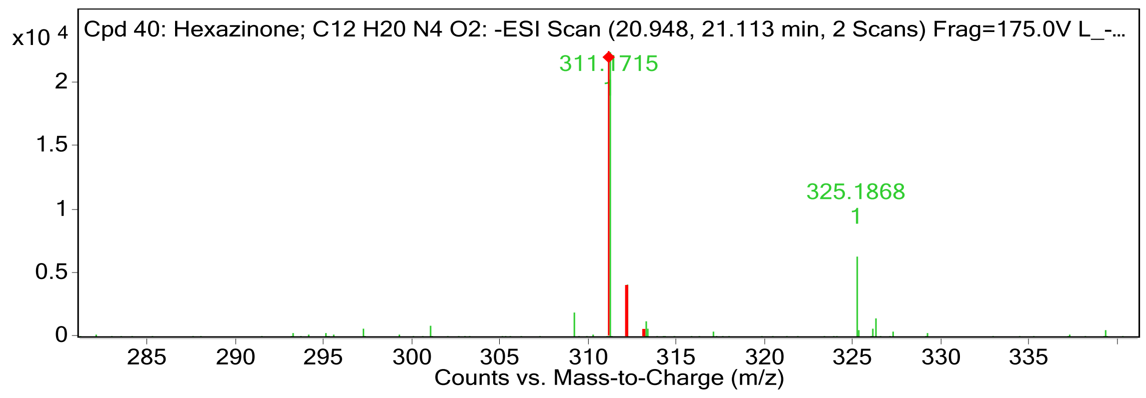

MS Spectrum Peak List

m/z	*Calc m/z*	Diff(ppm)	z	Abund	Formula	Ion
116.9302			1	14763.61		
134.8965			1	56143.23		
136.8936			1	18691.62		
311.1715	311.1725	3.08	1	22450.15	C12 H20 N4 O2	(M+CH3COO)-
312.1748	312.1753	1.88	1	4154.6	C12 H20 N4 O2	(M+CH3COO)-

313.1715	313.1776	19.7	1	1271.76	C12 H20 N4 O2	(M+CH3COO)-
627.4055			1	12349.01		
669.4561			1	10507.47		
673.4867			1	13996.05		
987.5531			1	16988.78		

MSMS Spectrum

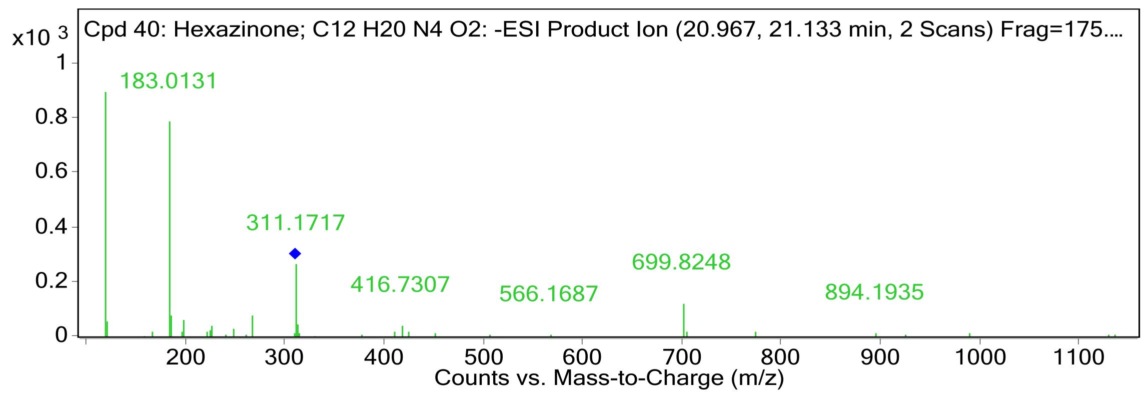

MS/MS Spectrum Peak List

m/z	z	Abund
119.0513	1	899.88
120.0475	1	61.75
182.9903		81.51
183.0131		791.08
184.0181	1	344.4
185.0102	1	80.63
197.0337		66.64
267.1027		81.35
311.1717	1	270.22
699.8248		125.97

Compound Structure

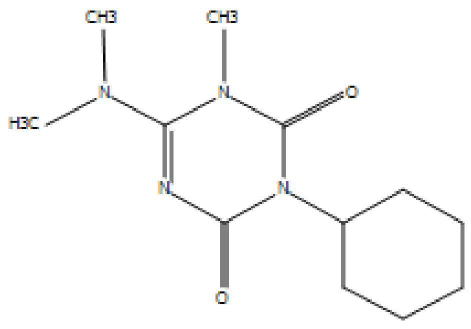

Compound Label	*m/z*	RT	Algorithm
Compound 41	668.4447	22.051	Auto MS/MS

MS Spectrum

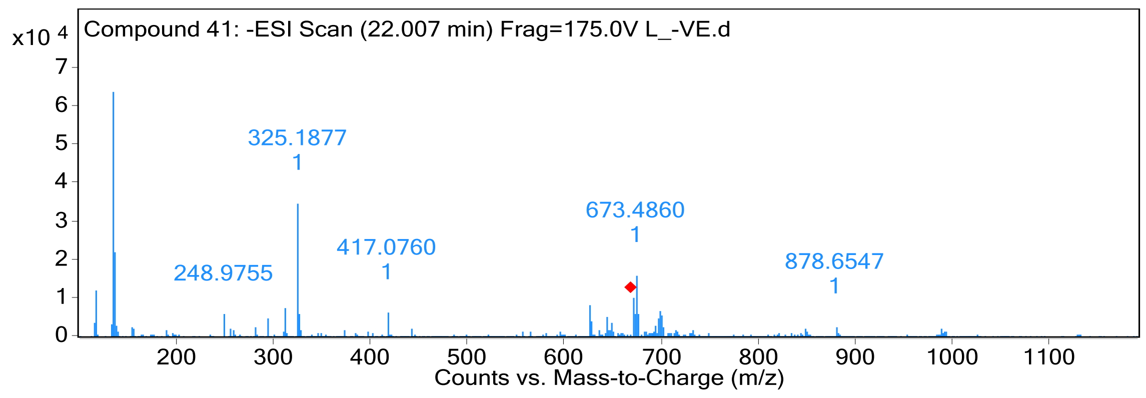

MS Zoomed Spectrum

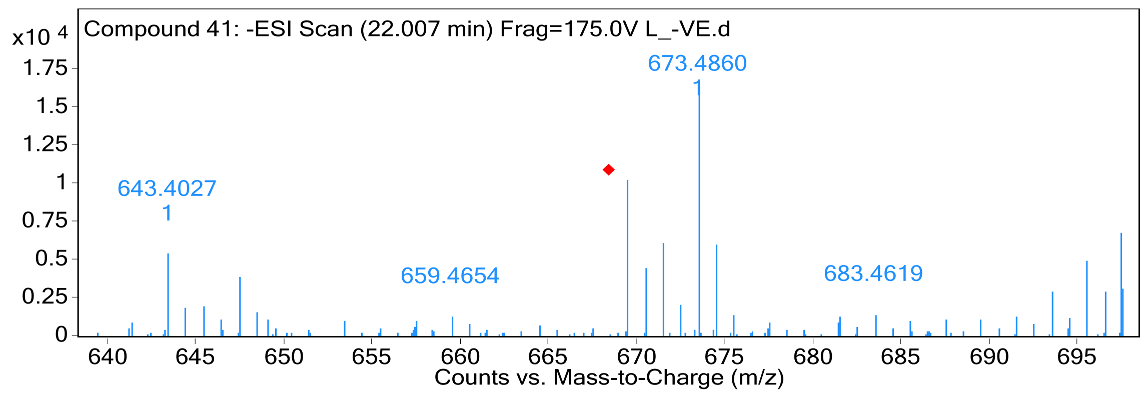

MS Spectrum Peak List

m/z	z	Abund
116.9298		12396.2
134.8963	1	63993.57
135.8969	1	8498.1
136.8934	1	22061.49
325.1877	1	34952.98
625.3922	1	8305.11
668.4447	2	209.57
668.9253	2	279.06
669.4544	1	10242.7
673.486	1	16097.2

MSMS Spectrum

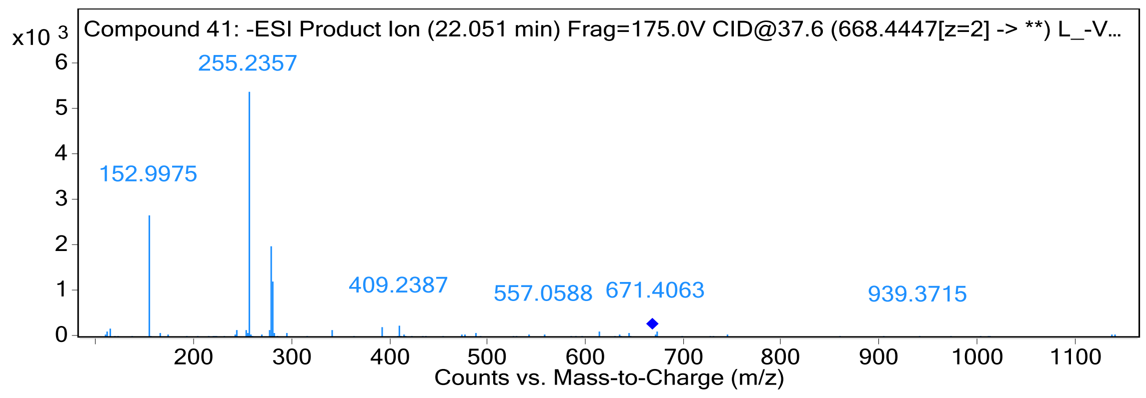

MS/MS Spectrum Peak List

m/z	z	Abund
114.0583		209.01
152.9776		263.06
152.9975	1	2685.51

255.2357	1	5379.84
256.2348	1	559.49
277.2194	1	1985.39
278.2223	1	299.75
279.2354		1216.36
391.2273	2	218
409.2387		243.15

Compound Label	Name	*m/z*	RT	Algorithm	Mass
Cpd 42: Muricatalin; C35 H64 O8	**Muricatalin**	671.4706	22.572	Auto MS/MS	612.4638

MS Spectrum

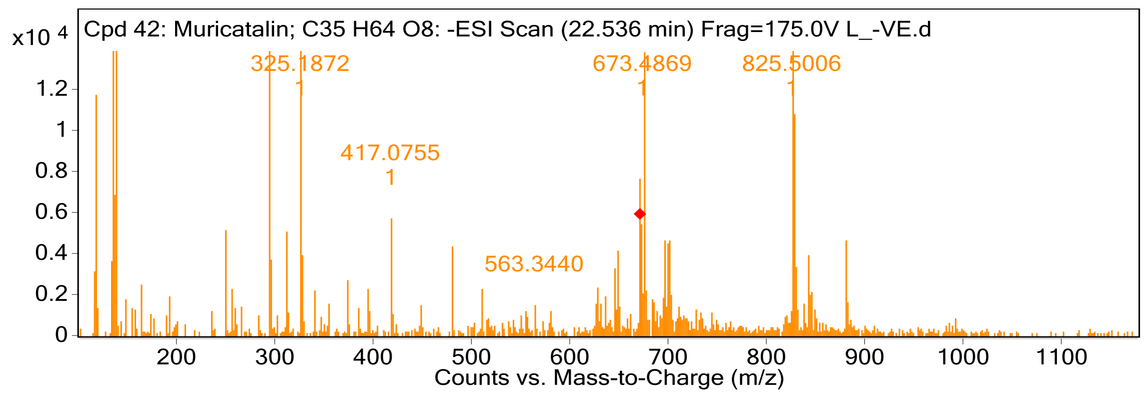

MS Zoomed Spectrum

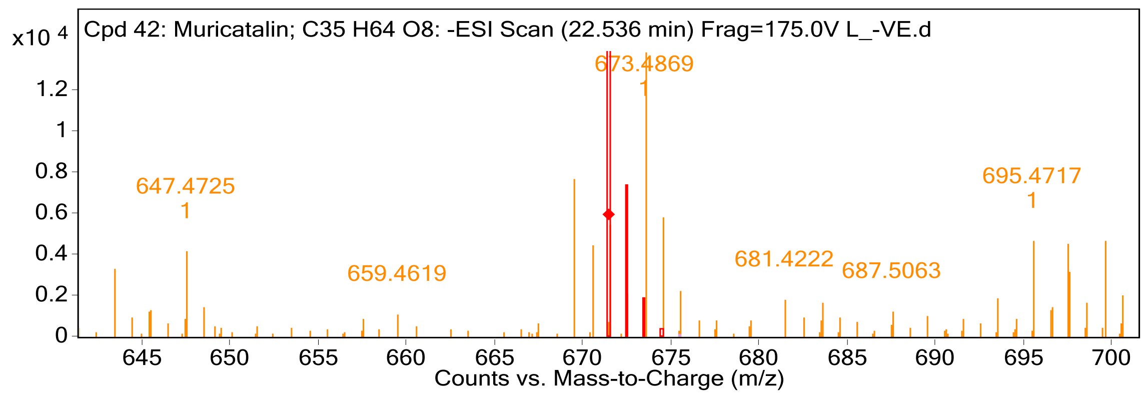

MS Spectrum Peak List

m/z	*Calc m/z*	Diff(ppm)	z	Abund	Formula	Ion
116.9299			1	11820.1		
134.8965			1	65017.07		
136.8934			1	20000.76		
293.1826			1	15250		
325.1872			1	15479.75		
671.4706	671.474	5.01	1	5517.38	C35 H64 O8	(M+CH3COO)-
672.4727	672.4774	6.91	1	2133.65	C35 H64 O8	(M+CH3COO)-
673.4869	673.4803	-9.83	1	13883.98	C35 H64 O8	(M+CH3COO)-
674.4897	674.4831	-9.73	1	5875.52	C35 H64 O8	(M+CH3COO)-
825.5006			1	23179.95		

MSMS Spectrum

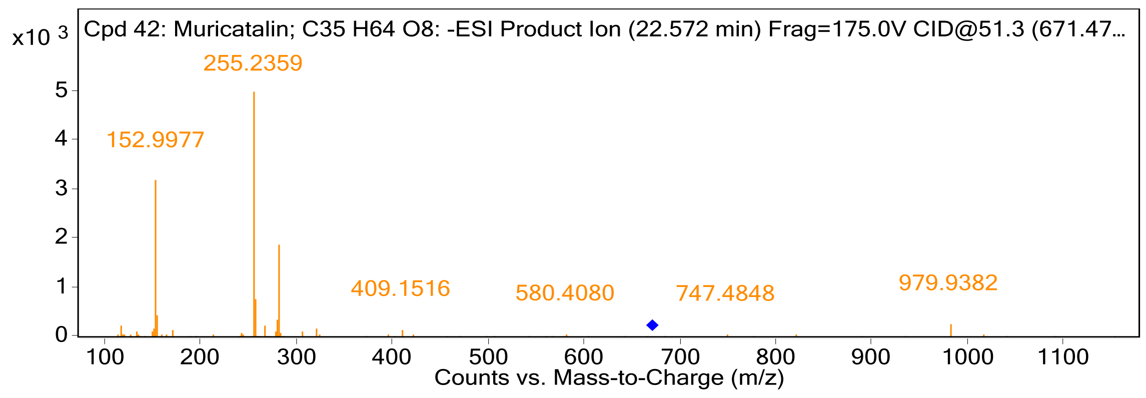

MS/MS Spectrum Peak List

m/z	z	Abund
117.0395		231.72
152.9977	1	3194.87
155.003	1	447.79
255.1882	1	301.21
255.2359	1	5006.8
256.2381	1	791.18
267.0721		228.02
279.2366		349.12
281.2517	1	1897.66
979.9382		272.25

Compound Structure

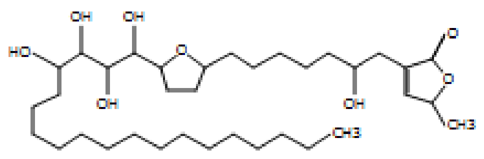

Compound Label	Name	*m/z*	RT	Algorithm	Mass
Cpd 43: 14,19-Dihydroaspidospermatine; C21 H28 N2 O2	**14,19-Dihydroaspidospermatine**	339.2031	23.787	Auto MS/MS	340.2102

MS Spectrum

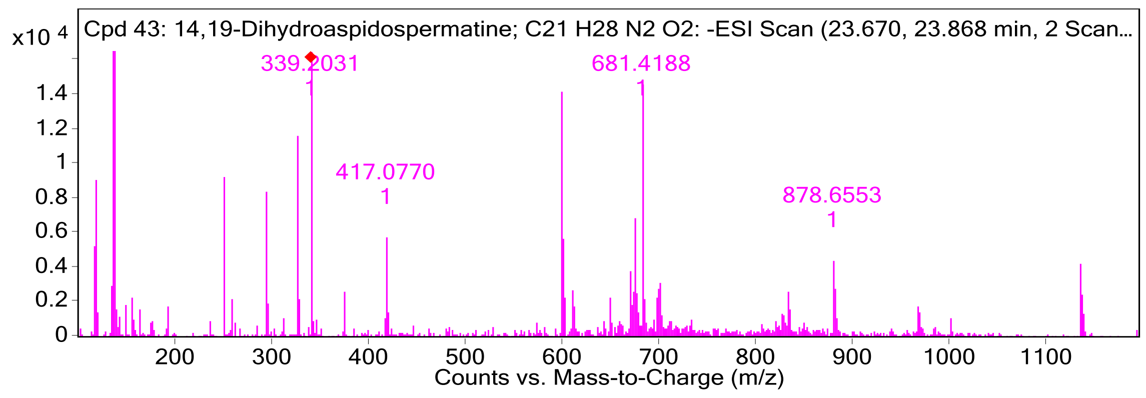

x10 4
Cpd 43: 14,19-Dihydroaspidospermatine; C21 H28 N2 O2: -ESI Scan (23.670, 23.868 min, 2 Scan...
339.2031
681.4188
417.0770
878.6553
Counts vs. Mass-to-Charge (m/z)

MS Zoomed Spectrum

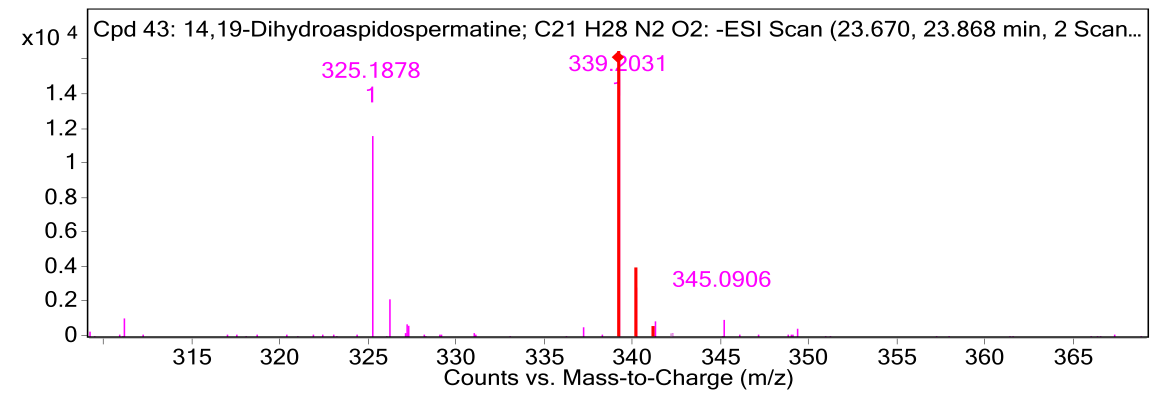

MS Spectrum Peak List

m/z	*Calc m/z*	Diff(ppm)	z	Abund	Formula	Ion
116.9301			1	9060.53		
134.8965			1	58111.59		
136.8939			1	20416.38		
248.9761				9227.04		
325.1878			1	11656.59		
339.2031	339.2078	13.74	1	16462.49	C21 H28 N2 O2	(M-H)-
340.2069	340.211	12.11	1	2888.7	C21 H28 N2 O2	(M-H)-
341.2029	341.2139	32.33	1	946.47	C21 H28 N2 O2	(M-H)-
597.3981			1	14135.35		
681.4188			1	14617.97		

MSMS Spectrum

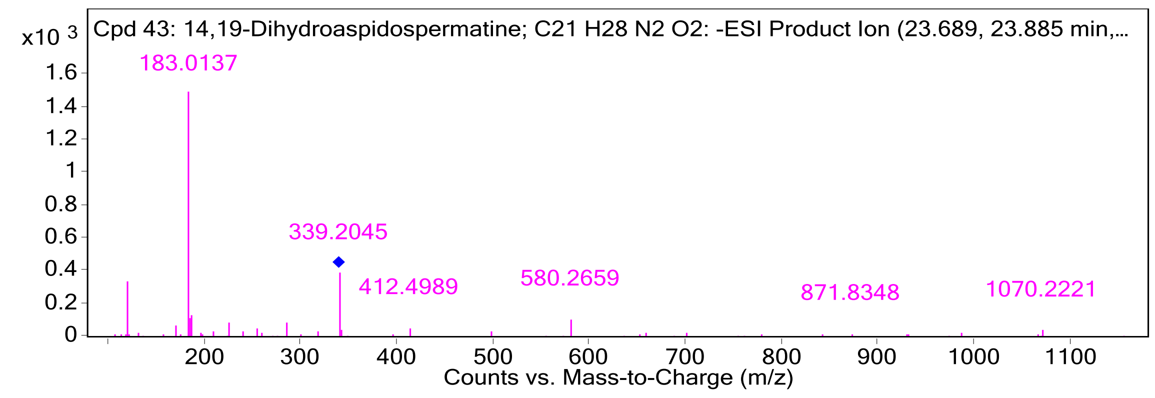

MS/MS Spectrum Peak List

m/z	z	Abund
119.0518	1	338.66
170.0041		72.44
183.0137	1	1497.7
184.0213	1	112.27
185.0107	1	130.09
225.0648		93.33
284.2683		85.23
339.1706		61.06
339.2045	1	392.22
580.2659		109.47

Compound Structure

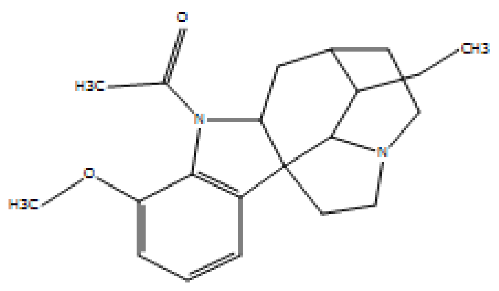

Compound Label	Name	*m/z*	RT	Algorithm	Mass
Cpd 44: 14,19-Dihydroaspidospermatine; C21 H28 N2 O2	**14,19-Dihydroaspidospermatine**	339.2034	24.069	Auto MS/MS	340.2105

MS Spectrum

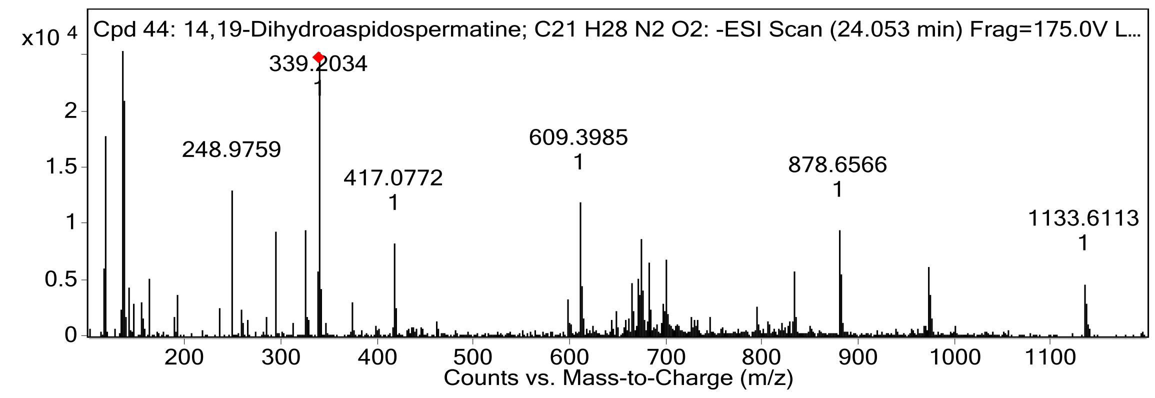

MS Zoomed Spectrum

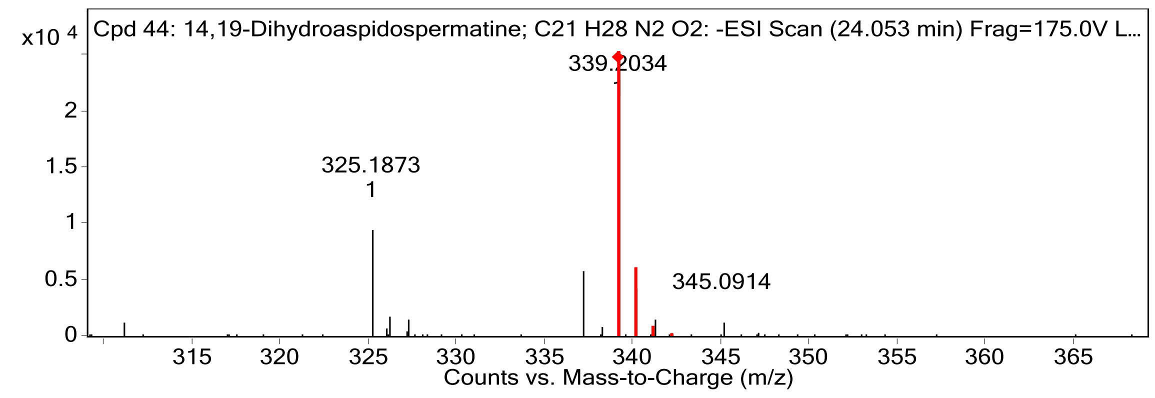

MS Spectrum Peak List

m/z	*Calc m/z*	Diff(ppm)	z	Abund	Formula	Ion
116.9299			1	17922.06		
134.8965			1	61716.58		
136.8936			1	21004.64		
248.9759				13066.62		
339.2034	339.2078	12.91	1	25299.17	C21 H28 N2 O2	(M-H)-
340.207	340.211	11.9	1	4250.72	C21 H28 N2 O2	(M-H)-

341.2053	341.2139	25.12	1	1579.77	C21 H28 N2 O2	(M-H)-
342.2122	342.2167	13.16	1	282.64	C21 H28 N2 O2	(M-H)-
609.3985			1	11954.64		
878.6566			1	9519.88		

MSMS Spectrum

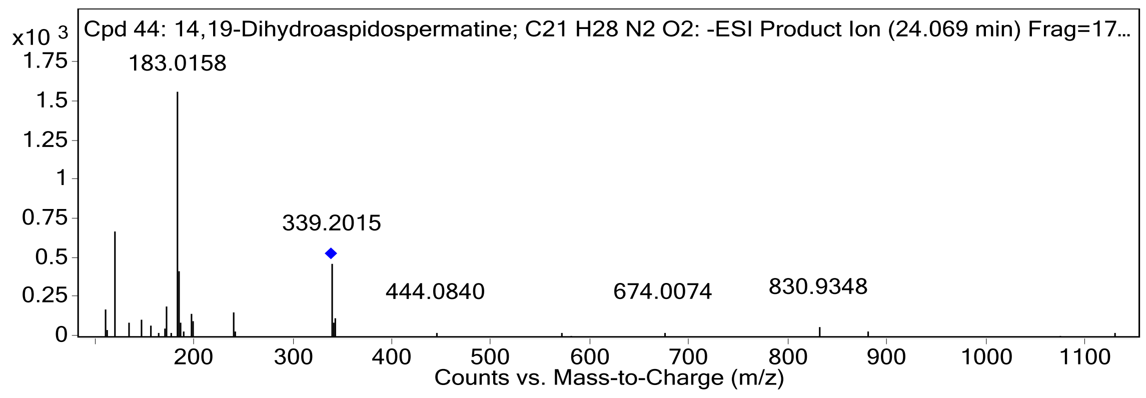

MS/MS Spectrum Peak List

m/z	z	Abund
110.059		174.46
119.0506	1	672.83
171.0057		199.48
183.0158	1	1568.2
184.0066		426.17
184.0202	1	367.09
197.0279		151.46
239.0474		160.81
339.2015	1	467.92
341.2012	1	120.06

Compound Structure

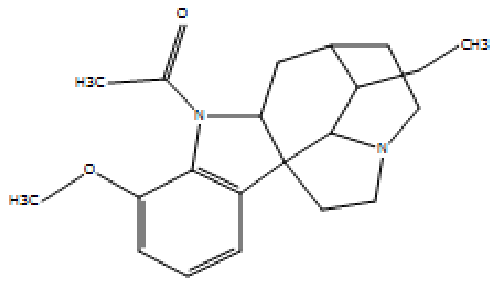

Compound Label	Name	*m/z*	RT	Algorithm	Mass
Cpd 45: Lycocernuine; C16 H26 N2 O2	**Lycocernuine**	337.2086	24.227	Auto MS/MS	278.1948

MS Spectrum

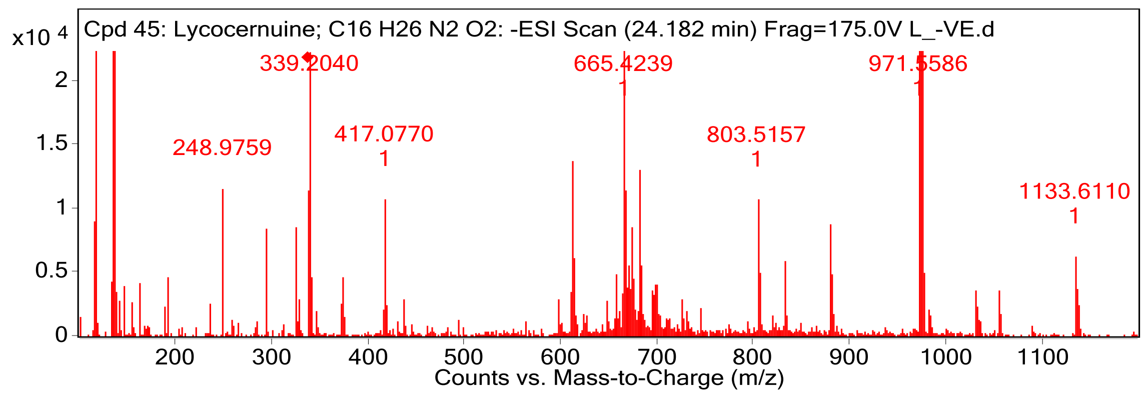

MS Zoomed Spectrum

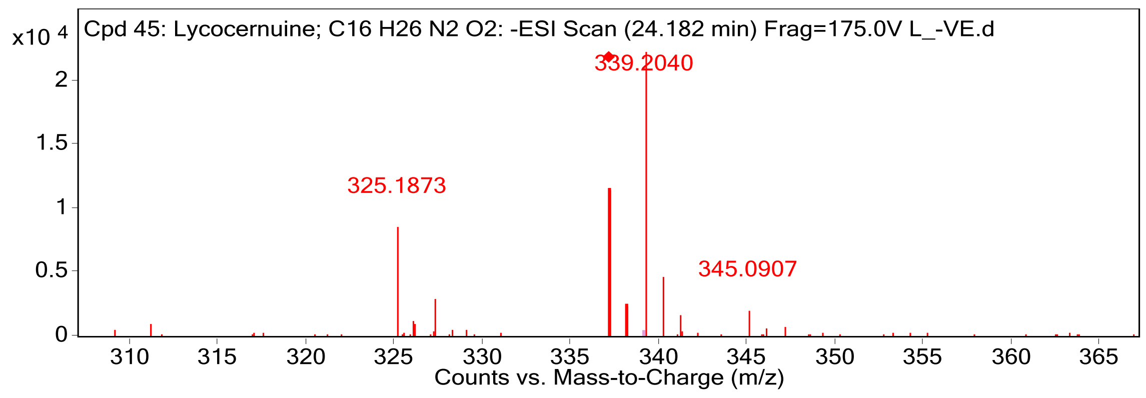

MS Spectrum Peak List

m/z	*Calc m/z*	Diff(ppm)	z	Abund	Formula	Ion
116.9304			1	26265.62		
134.8965			1	101247.31		
136.8938			1	36113.12		
337.2086	337.2133	13.79	1	11484.03	C16 H26 N2 O2	(M+CH3COO)-
338.2119	338.2165	13.51	1	2375.54	C16 H26 N2 O2	(M+CH3COO)-
339.204				22291.12		
665.4239			1	26440.11		
971.5586			1	73170.38		
972.5621			1	38905.89		
973.5598			1	29823.06		

MSMS Spectrum

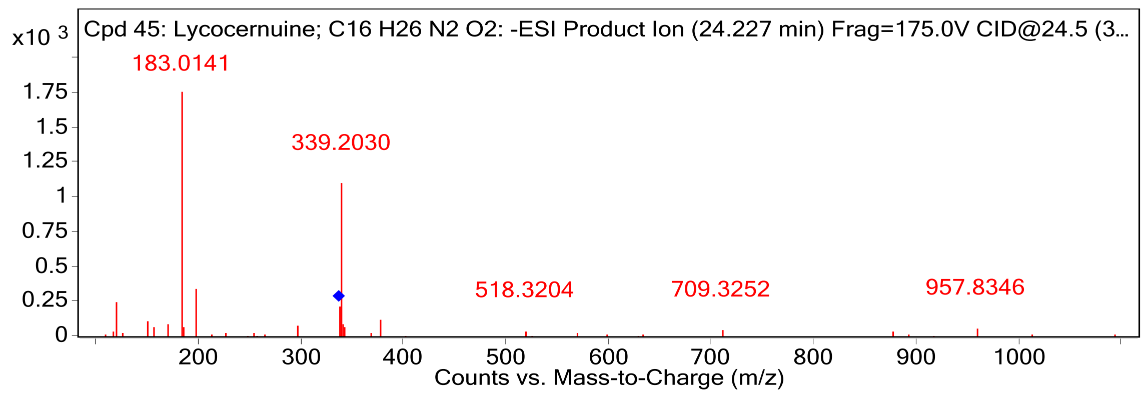

MS/MS Spectrum Peak List

m/z	z	Abund
119.0514		256.55
182.9818		172.68
183.0141	1	1762.37

184.0189	1	259.63
197.0301		348.11
337.1799		217.27
337.2067		120.36
338.2148		149.6
339.203	1	1105.52
377.2669		126.02

Compound Structure

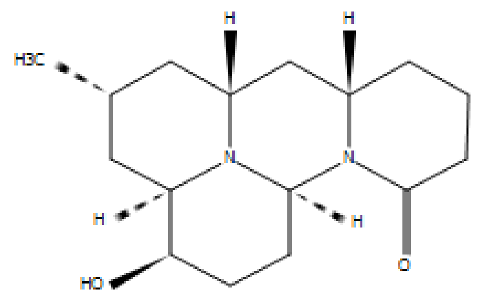

Compound Label	*m/z*	RT	Algorithm
Compound 46	665.4239	24.236	Auto MS/MS

MS Spectrum

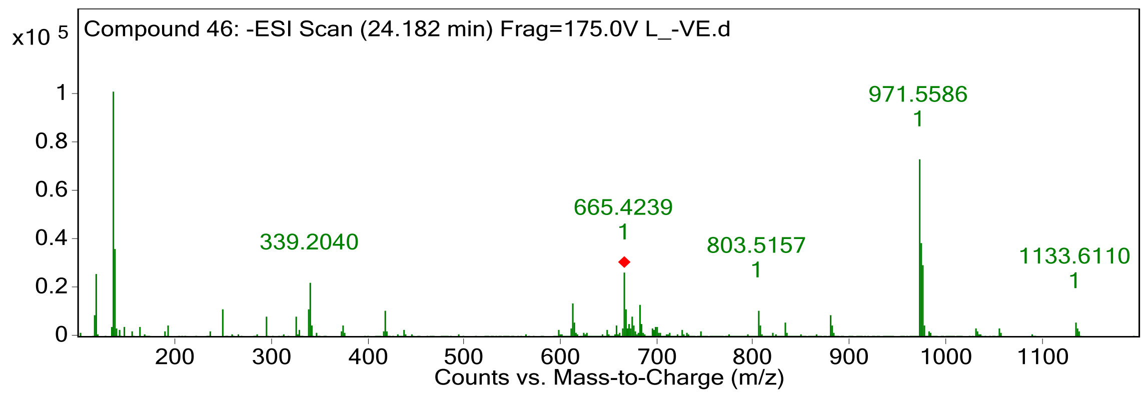

MS Zoomed Spectrum

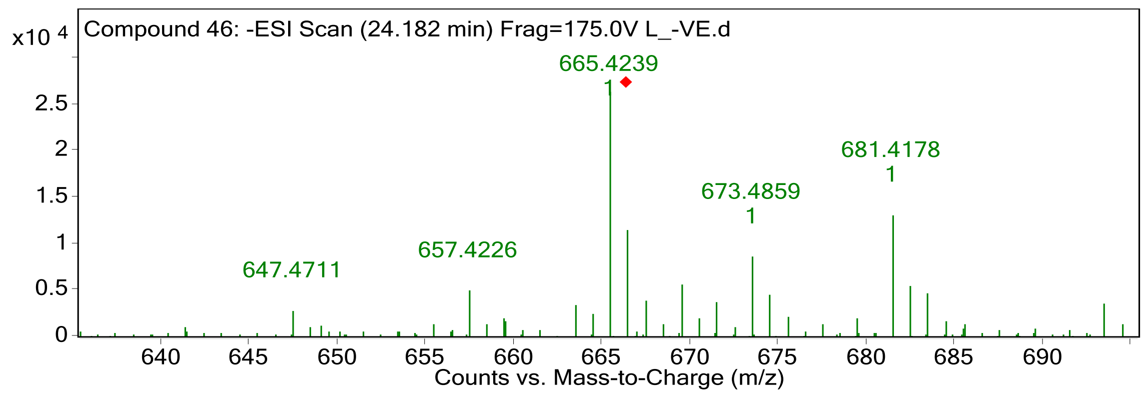

MS Spectrum Peak List

m/z	z	Abund
116.9304	1	26265.62
134.8965	1	101247.31
136.8938	1	36113.12
665.4239	1	26440.11
666.4257	1	11503.03
667.4244	1	3883.5
668.4083	1	1343.13
971.5586	1	73170.38
972.5621	1	38905.89
973.5598	1	29823.06

MSMS Spectrum

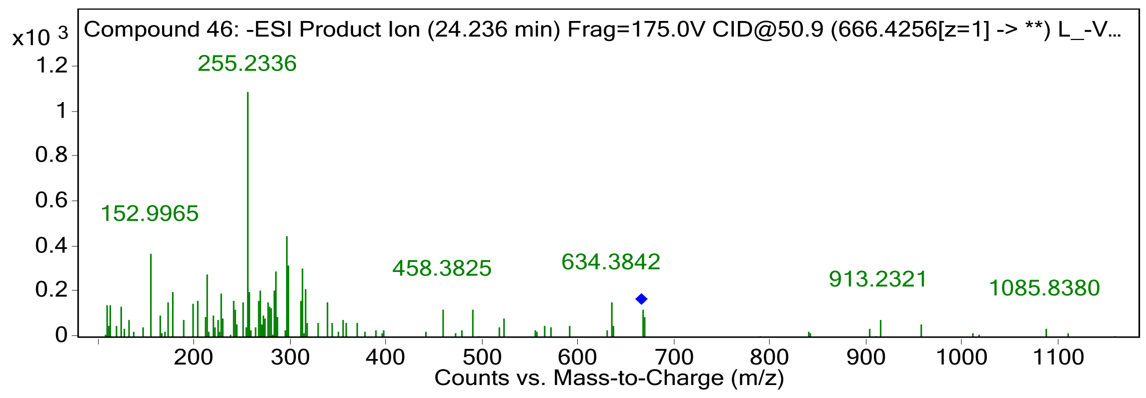

MS/MS Spectrum Peak List

m/z	z	Abund
152.9965		368.96
213.0433		277.36
255.2336	1	1091.66
282.0668		209.89
283.0758		285.61
283.1114		294.07
295.0724		449.64
296.0789		320.61
312.1109		309.6
315.0996	1	215.55

--- End Of Report ---
