# Supplementary material for: Metabolomic Profiling of Leptadenia reticulata: Unveiling Therapeutic Potential for Inflammatory Diseases through Network Pharmacology and Docking Studies
Source: Pharmaceuticals (Basel). 2024 Mar 26;17(4):423. doi: 10.3390/ph17040423 (PMC11054655; doi:10.3390/ph17040423)
Supplement: Supplementary file 1 [file pharmaceuticals-17-00423-s001.zip › HR LCMS chromatogram/L_CompoundReport.pdf]

Qualitative Compound Report

**Data File**	L.d	**Sample Name**	L
**Sample Type**	Sample	**Position**	P1-A3
**Instrument Name**	QTOF	**User Name**	
**Acq Method**	metabolite_ESI_+VE_MSMS.m	**Acquired Time**	5/4/2023 10:15:15 AM
**IRM Calibration Status**	Success	**DA Method**	default.m
**Comment**			

Sample Group **Info.**

**Acquisition SW Version**	6200 series TOF/6500 series Q-TOF B.05.01 (B5125.3)

Compound Table

Compound Label	RT	Mass	Abund	Name	Formula	MFG Formula	DB Formula	DB Diff (ppm)	Hits (DB)
Cpd 1: Brassilexin; C9 H6 N2 S	0.762	174.0277	32321	Brassilexin	C9 H6 N2 S	C9 H6 N2 S	C9 H6 N2 S	-14.39	10
Cpd 2: 1-Pyrenylsulfate; C16 H10 O4 S	0.826	298.0355		1-Pyrenylsulfate	C16 H10 O4 S	C16 H10 O4 S	C16 H10 O4 S	-18.44	1
Cpd 3: Chinomethionat; C10 H6 N2 O S2	0.833	233.9902		Chinomethionat	C10 H6 N2 O S2	C10 H6 N2 O S2	C10 H6 N2 O S2	8.22	1
Cpd 4: Neotussilagine; C10 H17 N O3	1.469	199.1209	214945	Neotussilagine	C10 H17 N O3	C10 H17 N O3	C10 H17 N O3	-0.32	7
Cpd 5: Neuraminic acid; C9 H17 N O8	1.623	267.0944	54770	Neuraminic acid	C9 H17 N O8	C9 H17 N O8	C9 H17 N O8	3.85	7
Cpd 6: Gabapentin; C9 H17 N O2	1.678	171.1245	243356	Gabapentin	C9 H17 N O2	C9 H17 N O2	C9 H17 N O2	8.3	1
Cpd 7: Neotussilagine; C10 H17 N O3	1.742	199.1215		Neotussilagine	C10 H17 N O3	C10 H17 N O3	C10 H17 N O3	-3.18	7
Compound 8	1.814								
Cpd 9: Gabapentin; C9 H17 N O2	2.006	171.1245		Gabapentin	C9 H17 N O2	C9 H17 N O2	C9 H17 N O2	8.42	1
Cpd 10: 1-Phenylbiguanide; C8 H11 N5	2.014	177.101		1-Phenylbiguanide	C8 H11 N5	C8 H11 N5	C8 H11 N5	2.73	4
Compound 11	2.135								
Compound 12	2.452								
Cpd 13: Methyl N-methylanthranilate; C9 H11 N	3.265	165.08		Methyl N-methylanthranilate	C9 H11 N O2	C9 H11 N O2	C9 H11 N O2	-5.98	10
Cpd 14: L-Tryptophan; C11 H12 N2 O2	3.286	204.0879		L-Tryptophan	C11 H12 N2 O2	C11 H12 N2 O2	C11 H12 N2 O2	9.87	9
Cpd 15: 6-Methylquinoline; C10 H9 N	3.33	143.0722	81959	6-Methylquinoline	C10 H9 N	C10 H9 N	C10 H9 N	9.01	10
Cpd 16: Isocarbostyril; C9 H7 N O	3.331	145.0517	82748	Isocarbostyril	C9 H7 N O	C9 H7 N O	C9 H7 N O	7.18	10
Cpd 17: Methyl N-methylanthranilate; C9 H11 N	3.536	165.08	276665	Methyl N-methylanthranilate	C9 H11 N O2	C9 H11 N O2	C9 H11 N O2	-6.02	10
Cpd 18: Isocarbostyril; C9 H7 N O	3.69	145.0517	70943	Isocarbostyril	C9 H7 N O	C9 H7 N O	C9 H7 N O	7.64	10
Cpd 19: Methyprylon; C10 H17 N O2	3.69	183.1269	402804	Methyprylon	C10 H17 N O2	C10 H17 N O2	C10 H17 N O2	-5.15	5
Cpd 20: 6-Methylquinoline; C10 H9 N	3.744	143.0723	46191	6-Methylquinoline	C10 H9 N	C10 H9 N	C10 H9 N	8.3	10
Cpd 21: Methyl N-methylanthranilate; C9 H11 N	3.849	165.0799		Methyl N-methylanthranilate	C9 H11 N O2	C9 H11 N O2	C9 H11 N O2	-5.7	10
Cpd 22: Methyprylon; C10 H17 N O2	4.023	183.1268	166539	Methyprylon	C10 H17 N O2	C10 H17 N O2	C10 H17 N O2	-4.76	5
Cpd 23: Pirbuterol; C12 H20 N2 O3	4.411	240.1476	35619	Pirbuterol	C12 H20 N2 O3	C12 H20 N2 O3	C12 H20 N2 O3	-0.87	6
Cpd 24: 2-Ethyl-5-methylpyridine; C8 H11 N	4.479	121.0903	76462	2-Ethyl-5-methylpyridine	C8 H11 N	C8 H11 N	C8 H11 N	-9.49	10
Compound 25	4.622		56495						
Cpd 26: Citrinin; C13 H14 O5	5.017	250.0835	18827	Citrinin	C13 H14 O5	C13 H14 O5	C13 H14 O5	2.5	10
Cpd 27: [2,2-bis(2-methylpropoxy)ethyl]benzene; C16 H26 O2	5.126	250.1891	16488	[2,2-bis(2-methylpropoxy)ethyl]benzene	C16 H26 O2	C16 H26 O2	C16 H26 O2	16.72	3
Cpd 28: Hexyl 2-furoate; C11 H16 O3	6.443	196.1087		Hexyl 2-furoate	C11 H16 O3	C11 H16 O3	C11 H16 O3	6.41	4
Cpd 29: 2,4,6-Triethyl-1,3,5-trioxane; C9 H18 O3	6.735	174.1264	90766	2,4,6-Triethyl-1,3,5-trioxane	C9 H18 O3	C9 H18 O3	C9 H18 O3	-4.39	4
Cpd 30: Maritimetin; C15 H10 O6	7.888	286.0455		Maritimetin	C15 H10 O6	C15 H10 O6	C15 H10 O6	7.93	10
Cpd 31: Ismine; C15 H15 N O3	8.808	257.1054	17728	Ismine	C15 H15 N O3	C15 H15 N O3	C15 H15 N O3	-0.68	6
Cpd 32: Lenacil; C13 H18 N2 O2	9.963	234.1368	57404	Lenacil	C13 H18 N2 O2	C13 H18 N2 O2	C13 H18 N2 O2	0.27	7
Compound 33	10.018		150216						
Cpd 34: 3-Hydroxynonyl acetate; C11 H22 O3	11.396	202.1574	32125	3-Hydroxynonyl acetate	C11 H22 O3	C11 H22 O3	C11 H22 O3	-2.53	10
Cpd 35: C16 Sphinganine; C16 H35 N O2	11.808	273.2653		C16 Sphinganine	C16 H35 N O2	C16 H35 N O2	C16 H35 N O2	5.48	1
Compound 36	12.272								
Cpd 37: Symlandine; C20 H31 N O6	12.348	381.214		Symlandine	C20 H31 N O6	C20 H31 N O6	C20 H31 N O6	2.86	2
Cpd 38: Lauroyl diethanolamide; C16 H33 N	12.449	287.2433		Lauroyl diethanolamide	C16 H33 N O3	C16 H33 N O3	C16 H33 N O3	9.53	1
Compound 39	12.457		138047						
Cpd 40: Gibberellin A74; C20 H28 O6	12.637	364.1875		Gibberellin A74	C20 H28 O6	C20 H28 O6	C20 H28 O6	3.03	10
Cpd 41: Thiamylal; C12 H18 N2 O2 S	12.854	254.1121	25481	Thiamylal	C12 H18 N2 O2 S	C12 H18 N2 O2 S	C12 H18 N2 O2 S	-12.65	2
Cpd 42: 2,6-Di-tert-butyl-4-ethylphenol; C16 H26 O	12.876	234.1986	29262	2,6-Di-tert-butyl-4-ethylphenol	C16 H26 O	C16 H26 O	C16 H26 O	-1.17	3
Cpd 43: Nigakilactone B; C22 H32 O6	13.531	392.2189		Nigakilactone B	C22 H32 O6	C22 H32 O6	C22 H32 O6	2.4	10
Cpd 44: Sphinganine; C18 H39 N O2	13.833	301.2954		Sphinganine	C18 H39 N O2	C18 H39 N O2	C18 H39 N O2	9.04	1

Cpd 45: 18-Nor-4(19),8,11,13 abietatetraene; C19 H26	16.38	254.2062		18-Nor-4(19),8,11,13-abietatetraene	C19 H26	C19 H26	C19 H26	-10.91	4
Cpd 46: Irinotecan; C33 H38 N4 O6	19.627	586.2768	180781	Irinotecan	C33 H38 N4 O6	C33 H38 N4 O6	C33 H38 N4 O6	4.03	5
Cpd 47: Oxidized dinoflagellate luciferin; C33 H38 N4 O7	19.985	602.2719	174585	Oxidized dinoflagellate luciferin	C33 H38 N4 O7	C33 H38 N4 O7	C33 H38 N4 O7	3.55	3
Cpd 48: Irinotecan; C33 H38 N4 O6	20.247	586.2771	365855	Irinotecan	C33 H38 N4 O6	C33 H38 N4 O6	C33 H38 N4 O6	3.43	5
Compound 49	23.149		95439						
Compound 50	23.787								

**Compound Label**	**Name**	***m/z***	**RT**	**Algorithm**	**Mass**
Cpd 1: Brassilexin; C9 H6 N2 S	**Brassilexin**	175.0354	0.762	Auto MS/MS	174.0277

MS Spectrum

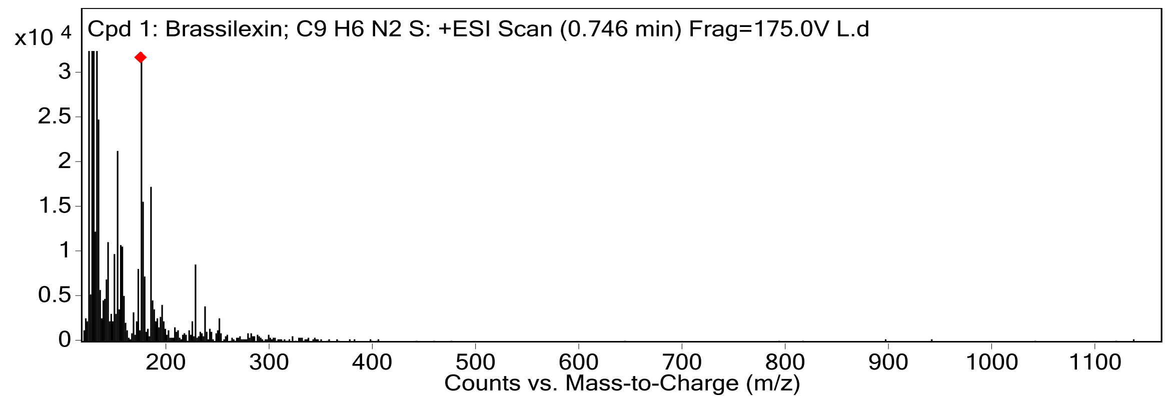

MS Zoomed Spectrum

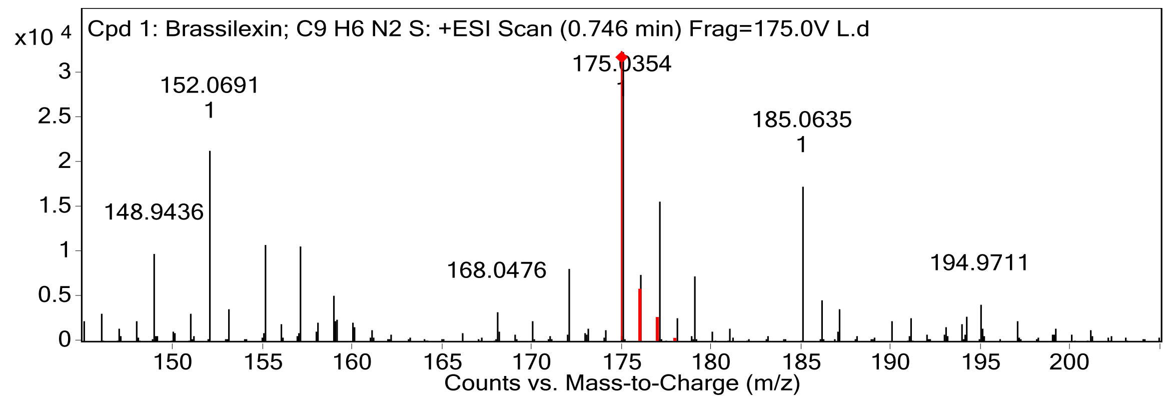

MS Spectrum Peak List

m/z	*Calc m/z*	Diff(ppm)	z	Abund	Formula	Ion
124.0858				51194.27		
125.0701			1	23736.42		
127.0228			1	49435.61		
129.9126			1	60697.67		
131.9098			1	68947.38		
133.9065				24858.7		
175.0354	175.0324	-17.14	1	32321.15	C9 H6 N2 S	(M+H)+
176.0361	176.0351	-5.38	1	7516.19	C9 H6 N2 S	(M+H)+
177.0319	177.0293	-14.38	1	15586.01	C9 H6 N2 S	(M+H)+
178.0322	178.0315	-3.73	1	2714.01	C9 H6 N2 S	(M+H)+

MSMS Spectrum

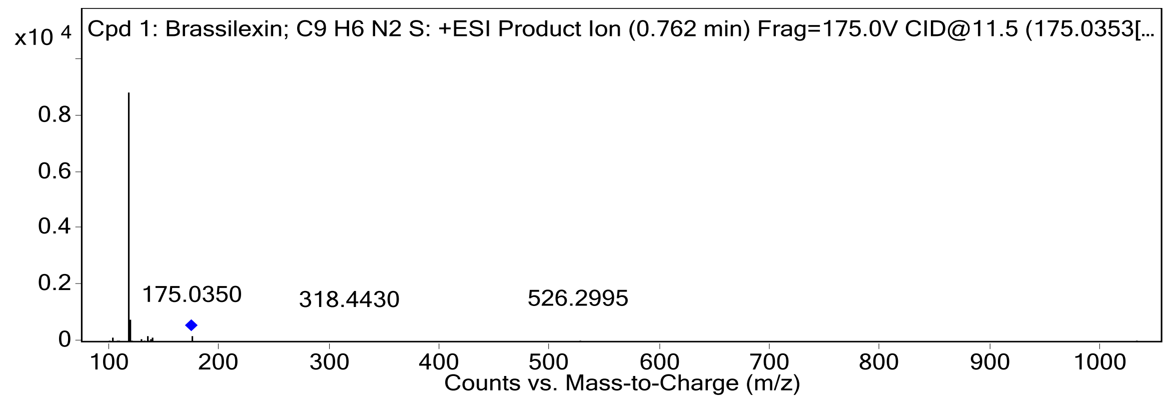

MS/MS Spectrum Peak List

m/z	z	Abund
102.035		148.04
116.6896		143.44
116.835		138.35
116.9946	1	8840.38
117.9957	1	809.68
118.9937	1	628.16
135.004	1	194.01
135.045		144.12
139.061		168.89
175.035	2	206.49

Compound Structure

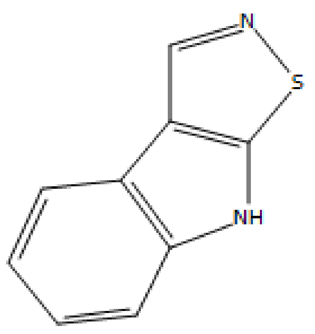

**Compound Label**	**Name**	***m/z***	**RT**	**Algorithm**	**Mass**

Cpd 2: 1-Pyrenylsulfate; C16 H10 O4 S	**1-Pyrenylsulfate**	299.0432	0.826	Auto MS/MS	298.0355

MS Spectrum

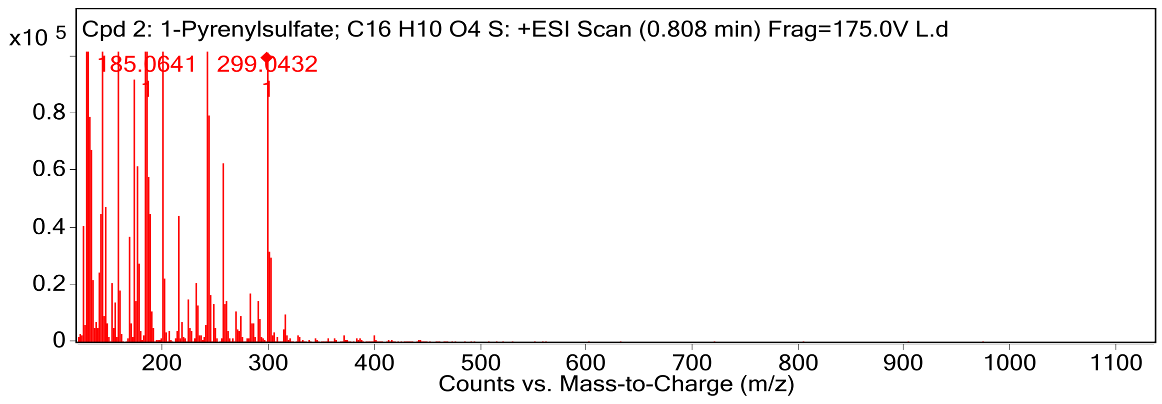

MS Zoomed Spectrum

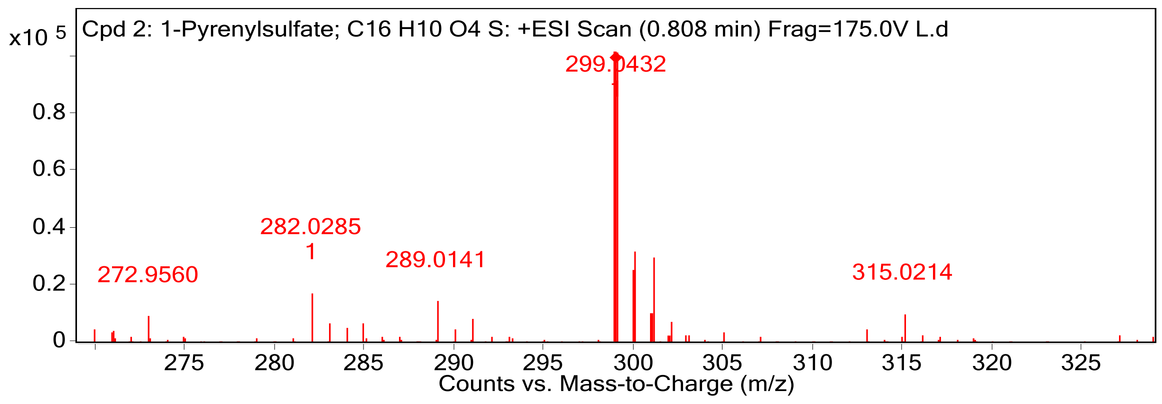

MS Spectrum Peak List

m/z	*Calc m/z*	Diff(ppm)	z	Abund	Formula	Ion
127.0229			1	887880.88		
128.0244			1	152307.16		
143			1	141332.92		
185.0641			1	286910.41		
198.9382			1	140320.86		
241.0022			1	257049.95		
299.0432	299.0373	-19.79	1	101559.71	C16 H10 O4 S	(M+H)+
300.0447	300.0405	-14.22	1	32144.92	C16 H10 O4 S	(M+H)+
301.042	301.0365	-18.28	1	29803.43	C16 H10 O4 S	(M+H)+
302.0434	302.0385	-15.96	1	7204.06	C16 H10 O4 S	(M+H)+

MSMS Spectrum

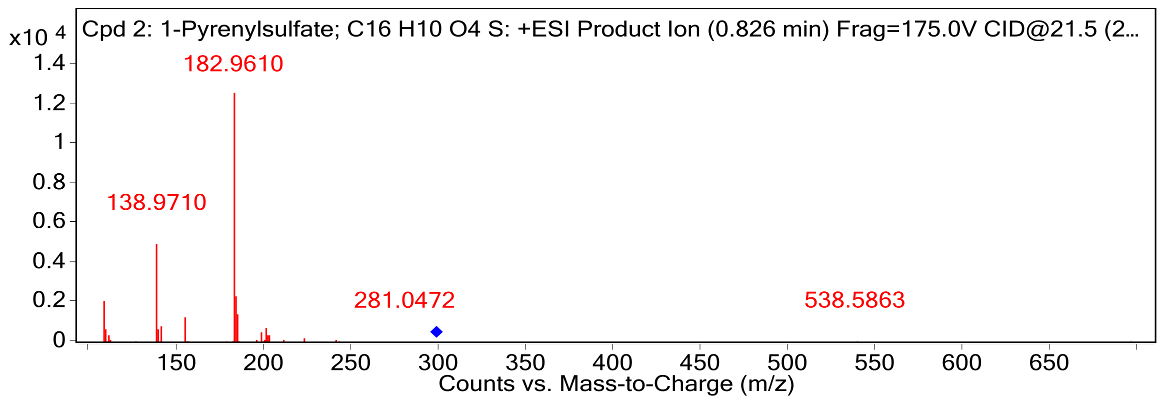

MS/MS Spectrum Peak List

m/z	z	Abund
108.9613		2126.14
109.9635		692.4
138.971	1	4979.72
139.9738	1	713.03
140.9664		801.71
154.9667	1	1304.02
182.961	1	12602.59
183.9619	1	2310.01
184.9605	1	1445.77
200.9704		729.29

Compound Structure

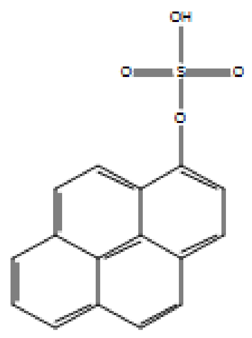

Compound Label	Name	*m/z*	RT	Algorithm	Mass
Cpd 3: Chinomethionat; C10 H6 N2 O S2	**Chinomethionat**	256.9793	0.833	Auto MS/MS	233.9902

MS Spectrum

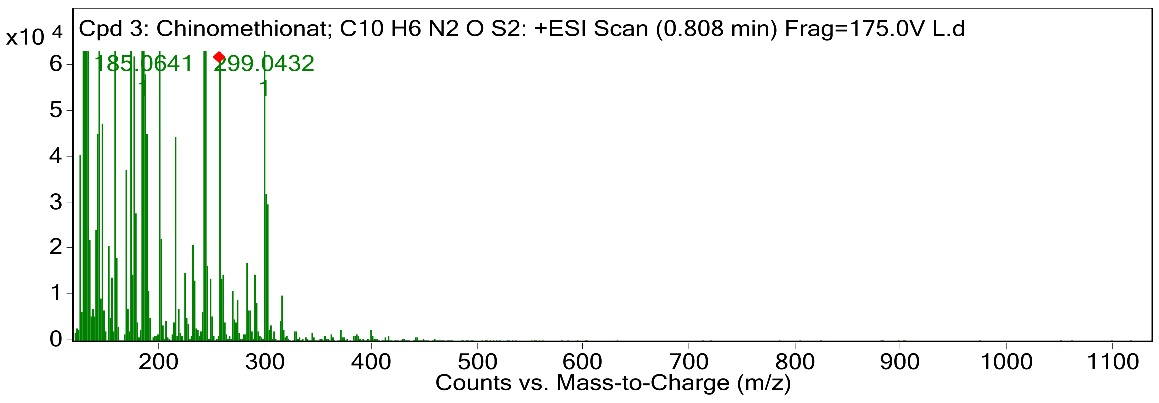

MS Zoomed Spectrum

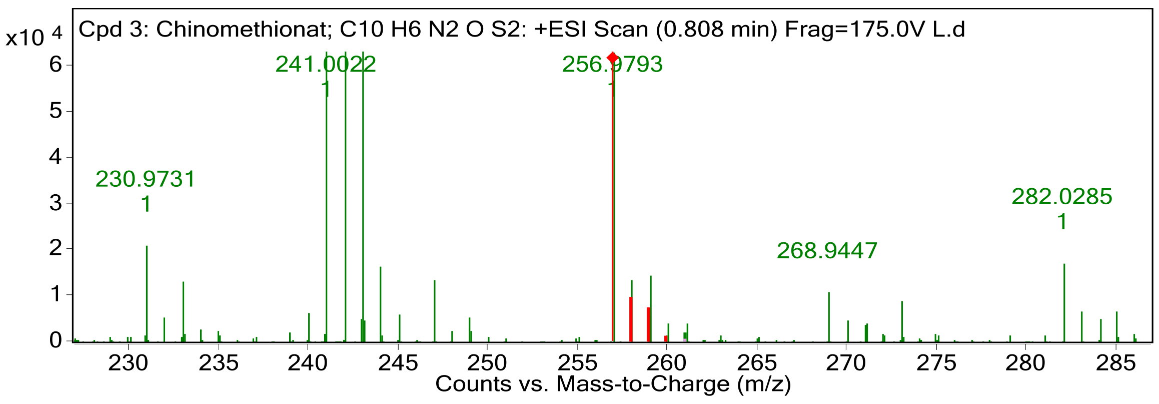

MS Spectrum Peak List

m/z	*Calc m/z*	Diff(ppm)	z	Abund	Formula	Ion
127.0229			1	887880.88		
128.0244			1	152307.16		
143			1	141332.92		
185.0641			1	286910.41		
198.9382			1	140320.86		
241.0022			1	257049.95		
256.9793	256.9814	8.11	1	62928.28	C10 H6 N2 O S2	(M+Na)+
257.9811	257.9839	11.08	1	13548.79	C10 H6 N2 O S2	(M+Na)+
258.9778	258.9781	1.3	1	10976.36	C10 H6 N2 O S2	(M+Na)+
259.9827	259.9803	-9.07	1	1440.57	C10 H6 N2 O S2	(M+Na)+

MSMS Spectrum

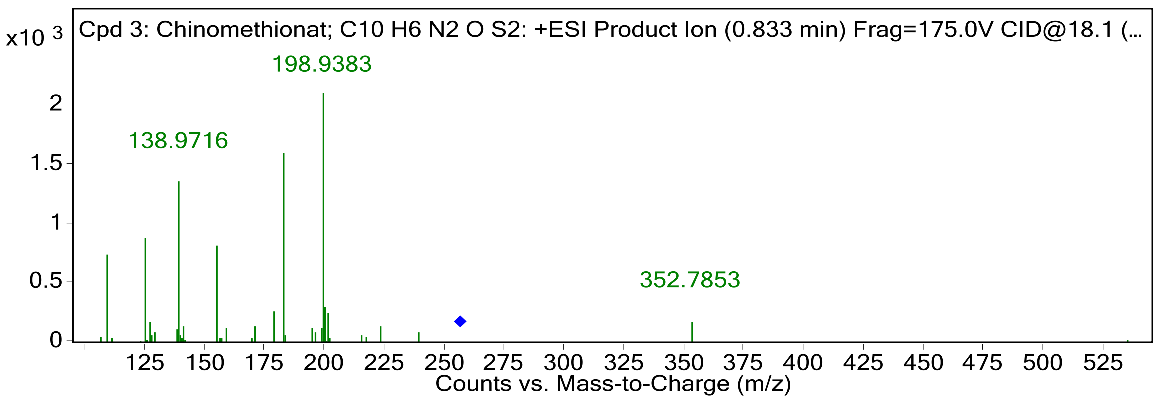

MS/MS Spectrum Peak List

m/z	z	Abund
108.9598		742.21
124.9393		885.9
138.9716		1354.3
154.9497		815.87
178.9502		265.32
182.9603		1598.07
198.9383	1	2104.41
199.9398	1	296.12
200.9372	1	253.63
352.7853		177.57

Compound Structure

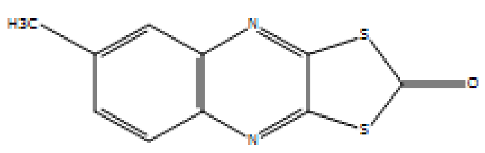

Compound Label	Name	*m/z*	RT	Algorithm	Mass
Cpd 4: Neotussilagine; C10 H17 N O3	**Neotussilagine**	222.1106	1.469	Auto MS/MS	199.1209

MS Spectrum

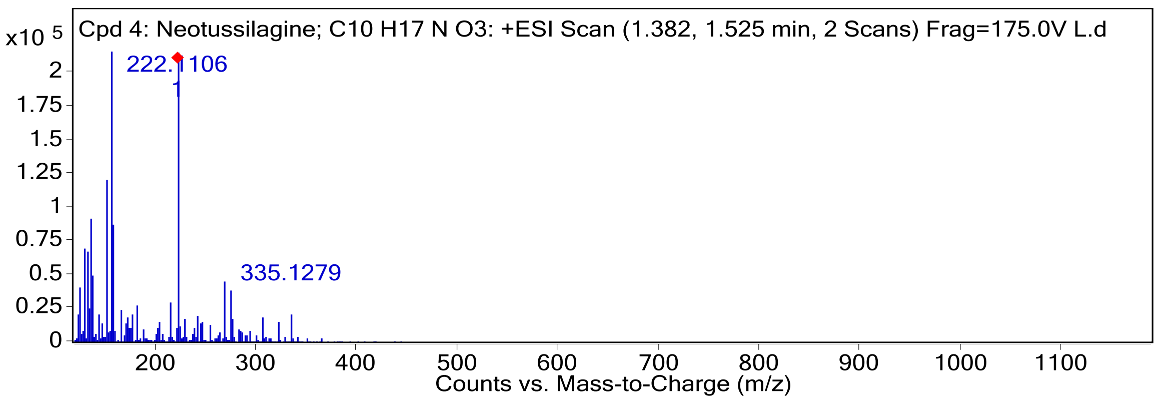

MS Zoomed Spectrum

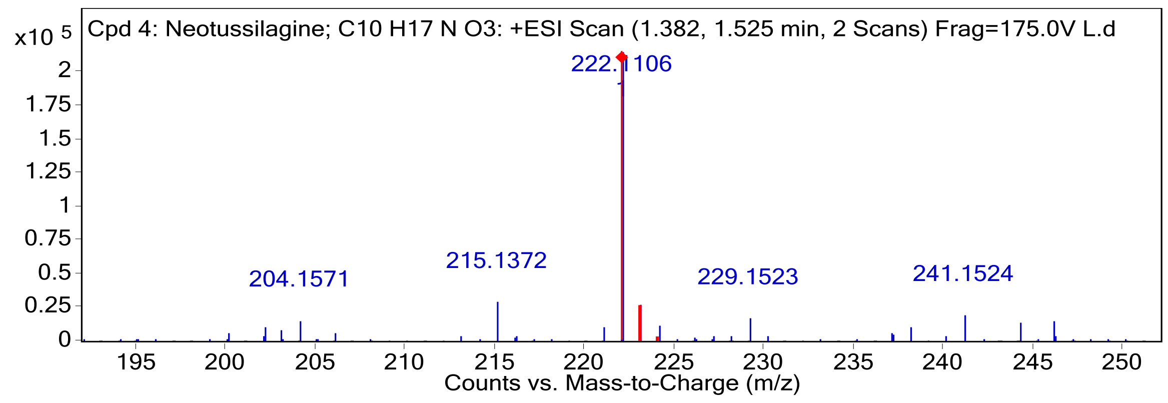

MS Spectrum Peak List

m/z	*Calc m/z*	Diff(ppm)	z	Abund	Formula	Ion
129.9125				70083.98		
131.9099				67375.28		
136.0605			1	92176		
152.0557			1	120556.88		
156.1005			1	1070959.63		
157.1037			1	87703.3		
158.116			1	85692.89		
222.1106	222.1101	-2.63	1	214944.95	C10 H17 N O3	(M+Na)+
223.1135	223.1133	-1.08	1	28104.19	C10 H17 N O3	(M+Na)+
224.1059	224.1154	42.17	1	12173.17	C10 H17 N O3	(M+Na)+

MSMS Spectrum

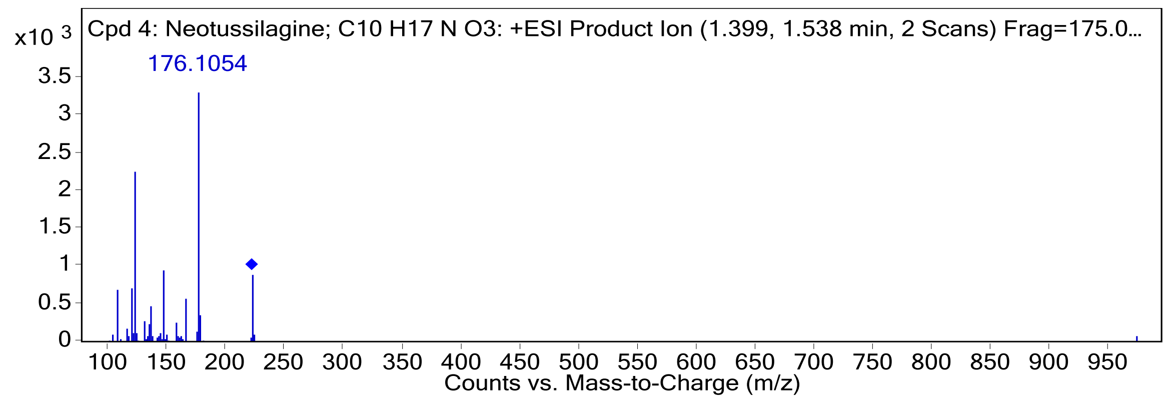

MS/MS Spectrum Peak List

m/z	z	Abund
107.0487	1	690.07
119.0479	1	714.09
123.0422	1	2256.61
130.0613		278.34
136.0729	1	478.79
147.0422	1	943.82
165.0524	1	569.99
176.1054	1	3304.6
177.1066	1	350.73
222.1099	1	893.78

Compound Structure

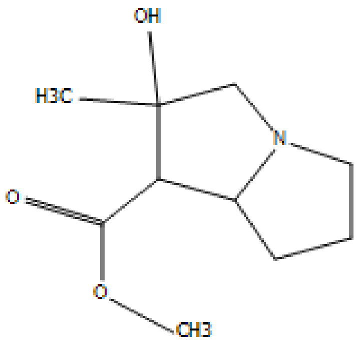

**Compound Label**	**Name**	***m/z***	**RT**	**Algorithm**	**Mass**
Cpd 5: Neuraminic acid; C9 H17 N O8	**Neuraminic acid**	268.1016	1.623	Auto MS/MS	267.0944

MS Spectrum

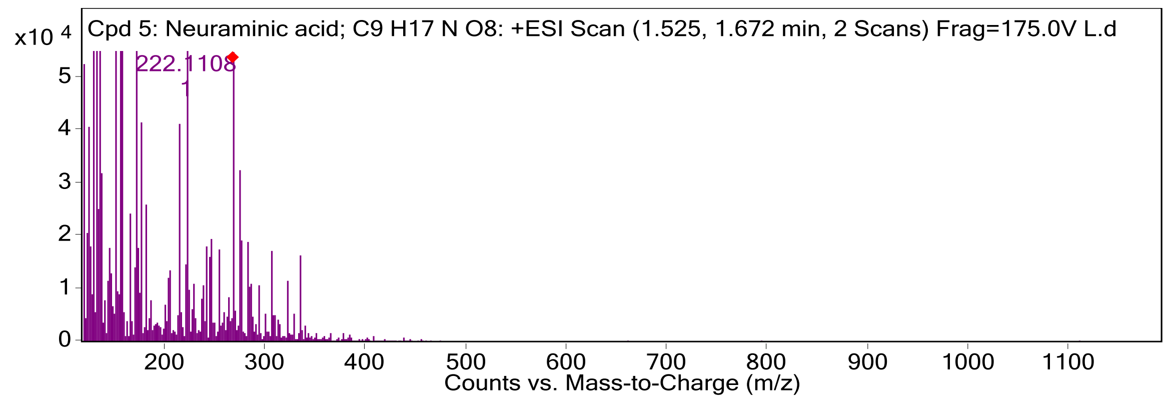

MS Zoomed Spectrum

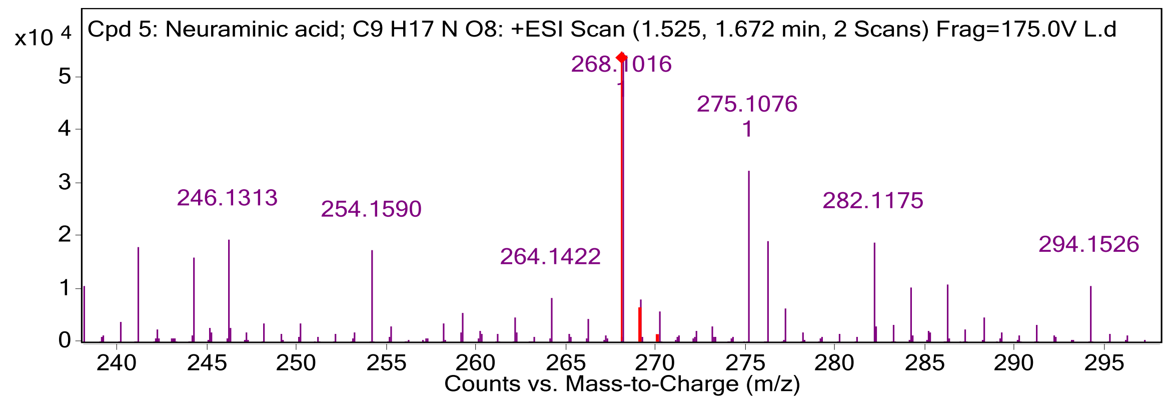

MS Spectrum Peak List

m/z	*Calc m/z*	Diff(ppm)	z	Abund	Formula	Ion
129.9127				65986.39		

131.9098				70380.52		
136.0608			1	113838.19		
152.0561			1	146592.44		
156.1008			1	290212.34		
172.1317			1	170525.64		
222.1108			1	410257.69		
268.1016	268.1027	4.13	1	54770.44	C9 H17 N O8	(M+H)+
269.105	269.1059	3.27	1	8205.13	C9 H17 N O8	(M+H)+
270.1079	270.1075	-1.51	1	1813.19	C9 H17 N O8	(M+H)+

MSMS Spectrum

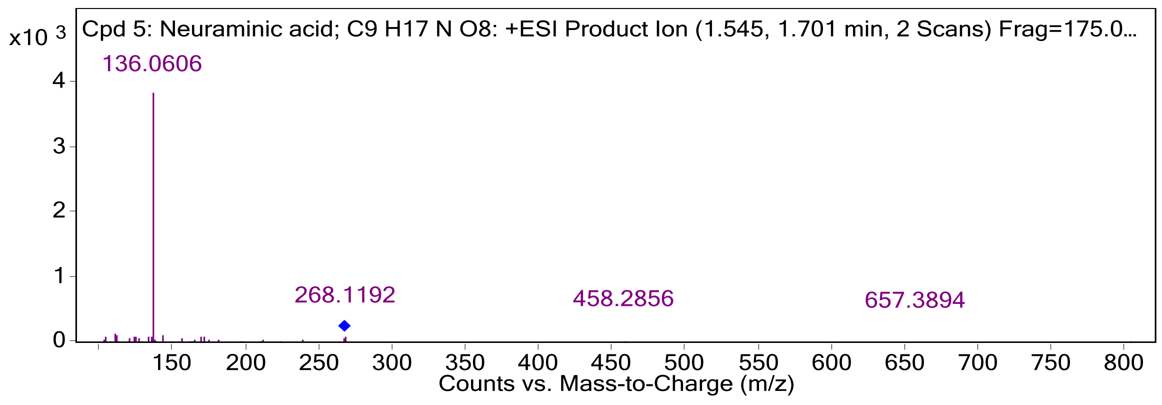

MS/MS Spectrum Peak List

m/z	z	Abund
110.0636		128.56
112.1078		119.86
124.0477		89.32
124.9646		91.79
133.0475		91.52
136.0078	1	98.43
136.0606	1	3852.9
137.0629	1	284.18
143.0315		112.54
268.1192		89.07

Compound Structure

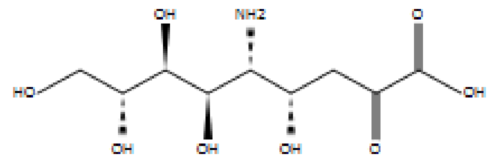

Compound Label	Name	*m/z*	RT	Algorithm	Mass
Cpd 6: Gabapentin; C9 H17 N O2	**Gabapentin**	172.1318	1.678	Auto MS/MS	171.1245

MS Spectrum

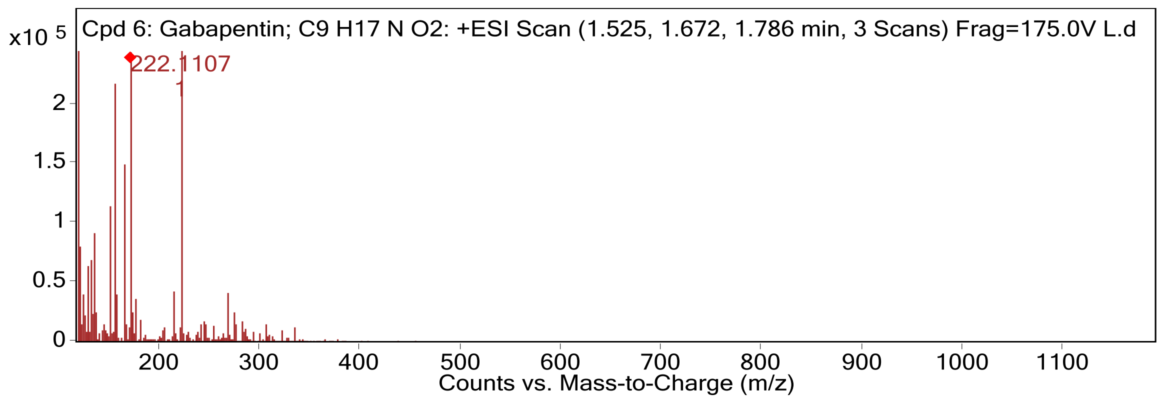

MS Zoomed Spectrum

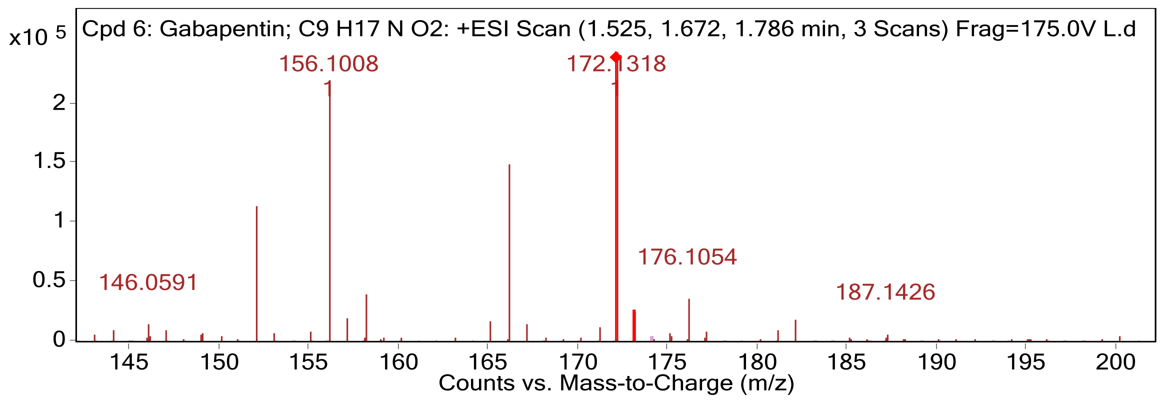

MS Spectrum Peak List

m/z	*Calc m/z*	Diff(ppm)	z	Abund	Formula	Ion
120.08			1	937651.06		
121.0833			1	79697.3		
131.9099				68421.41		
136.0609			1	91895.5		
152.056			1	114169.27		
156.1008			1	216400.83		
166.085			1	149748.41		
172.1318	172.1332	8.13	1	243356.45	C9 H17 N O2	(M+H)+
173.1348	173.1364	9.39	1	24762.95	C9 H17 N O2	(M+H)+
222.1107			1	351369.91		

MSMS Spectrum

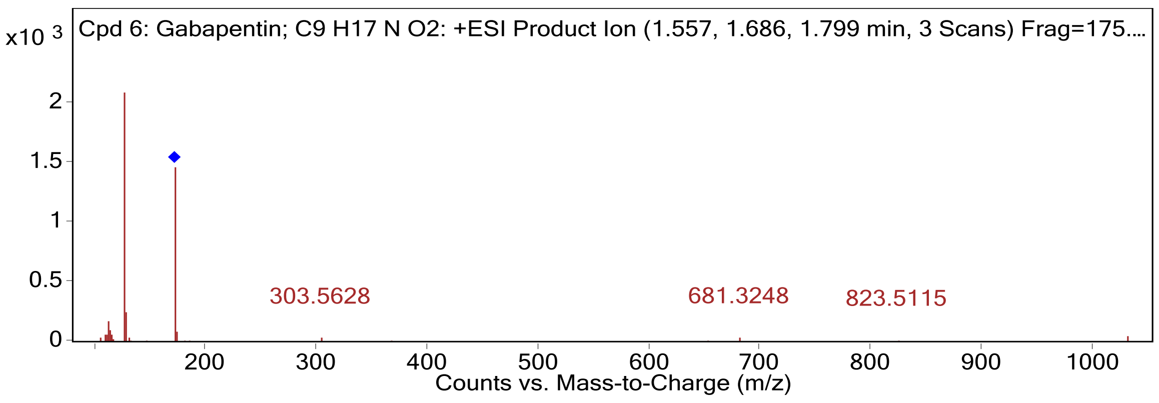

MS/MS Spectrum Peak List

m/z	z	Abund
109.1013		66.92
112.1111	1	171.77
113.0584		104.71
114.1026	1	78.26
126.1269	1	2086.15
127.1302	1	245.66
128.0689		69.63
172.1313	1	1464.23
173.0798		91.21
173.1365	1	65.63

Compound Structure

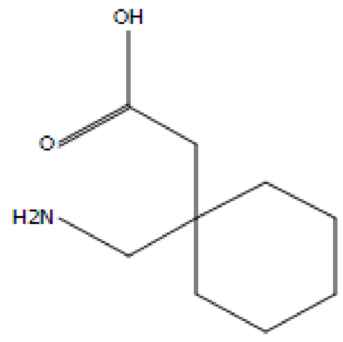

Compound Label	Name	*m/z*	RT	Algorithm	Mass
Cpd 7: Neotussilagine; C10 H17 N O3	**Neotussilagine**	222.1107	1.742	Auto MS/MS	199.1215

MS Spectrum

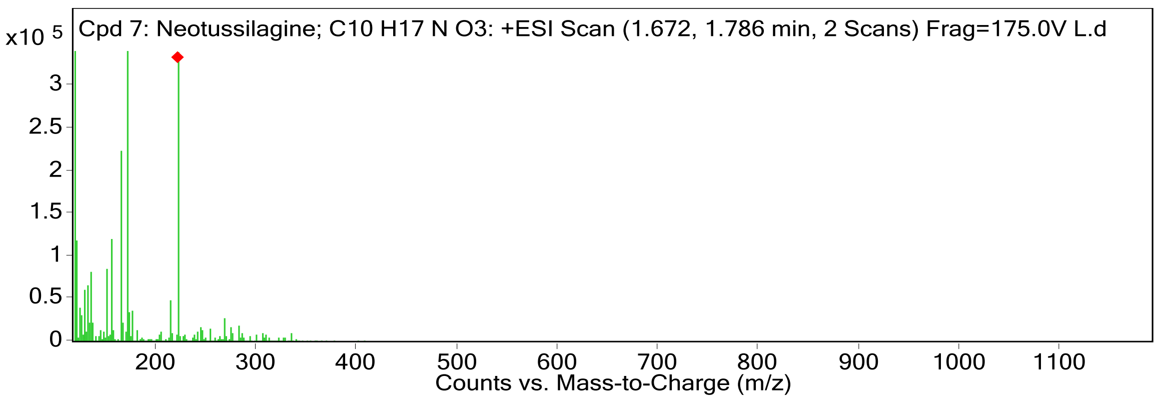

MS Zoomed Spectrum

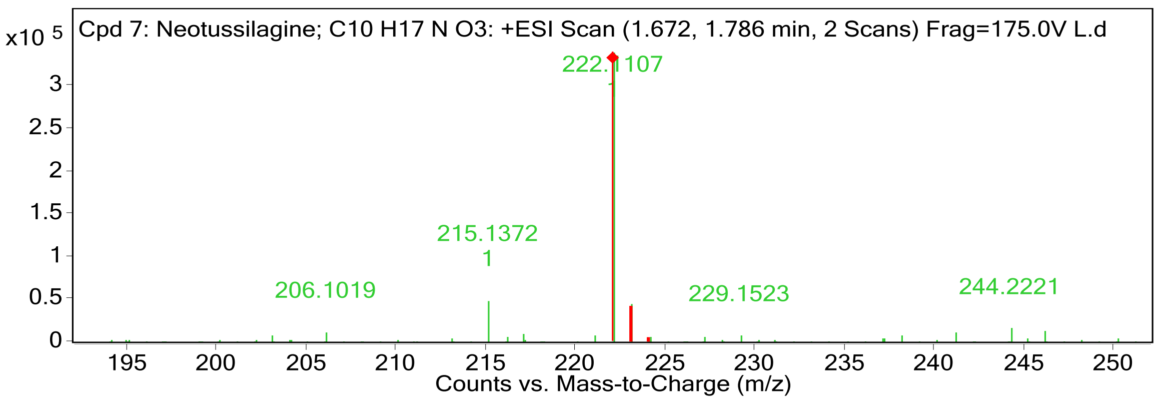

MS Spectrum Peak List

m/z	*Calc m/z*	Diff(ppm)	z	Abund	Formula	Ion
120.08			1	1406476.63		
121.0833			1	119545.95		
136.0612			1	81827.13		
152.0559			1	84985.74		
156.1006			1	121163.77		
166.085			1	224622.61		
172.1318			1	346650.41		
222.1107	222.1101	-3.04	1	339222.97	C10 H17 N O3	(M+Na)+
223.1136	223.1133	-1.43	1	44604.59	C10 H17 N O3	(M+Na)+
224.1155	224.1154	-0.38	1	6750.34	C10 H17 N O3	(M+Na)+

MSMS Spectrum

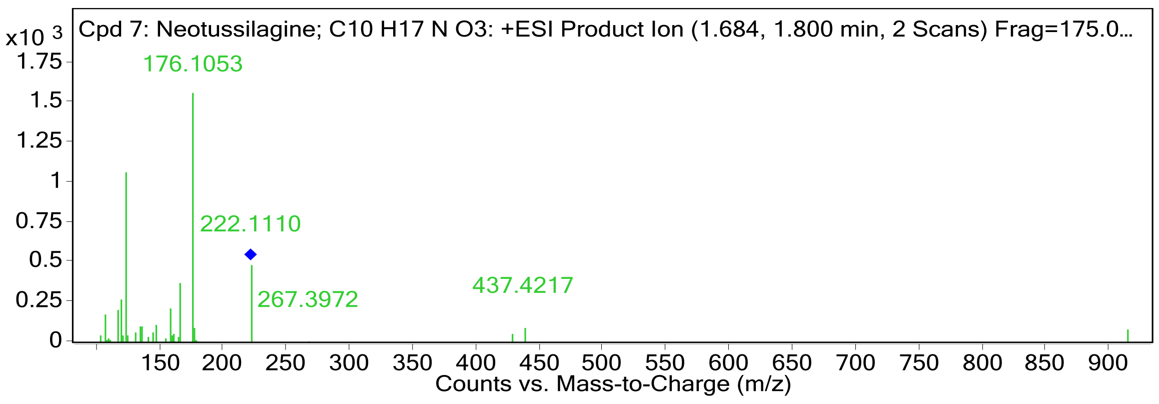

MS/MS Spectrum Peak List

m/z	z	Abund
107.0467	1	173.95

116.0698		205.87
119.0463	1	270.49
123.0426	1	1064.28
134.0619		106.48
147.0512		111.88
158.0587		212.86
165.0523		377.2
176.1053	1	1561.59
222.111		482.45

Compound Structure

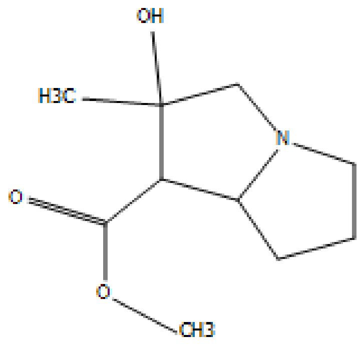

Compound Label	*m/z*	RT	Algorithm
Compound 8	120.08	1.814	Auto MS/MS

MS Spectrum

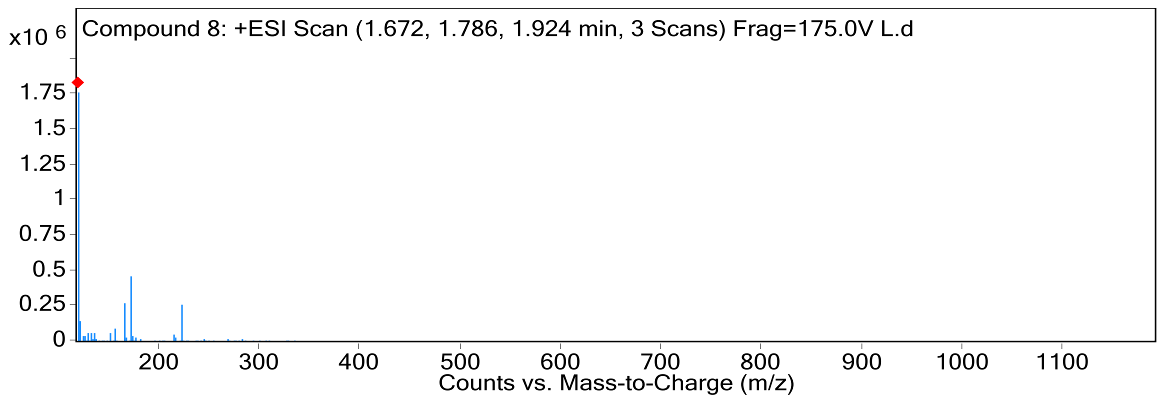

MS Zoomed Spectrum

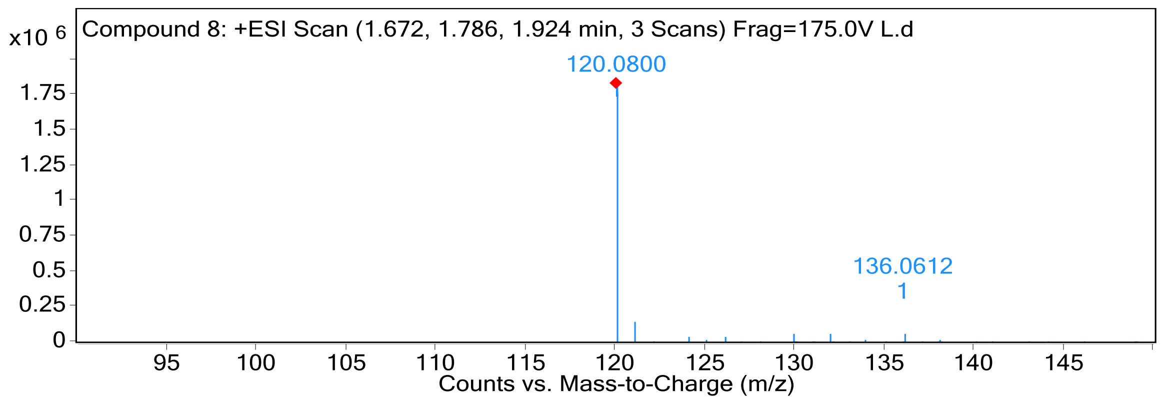

MS Spectrum Peak List

m/z	z	Abund
120.08	1	1765821
121.0833	1	147855
129.9126		62467.91
131.9098		67939.84
136.0612	1	68596.91
152.0558	1	64926.57
156.1006	1	94486.72
166.0851	1	277331.16
172.1318	1	460606.09
222.1107	1	266488

MSMS Spectrum

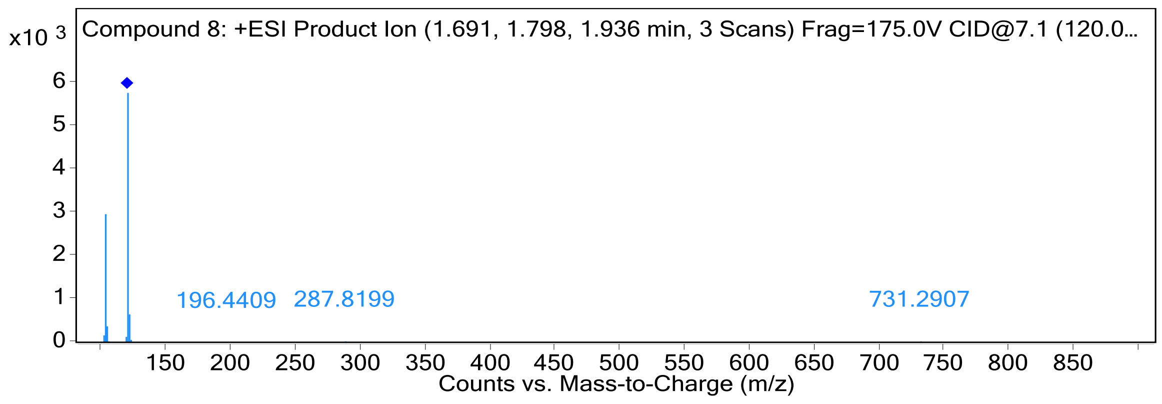

MS/MS Spectrum Peak List

m/z	z	Abund
102.0441		84.25
102.0513		164.26
103.0534	1	2980.97
104.0565		384.28
119.0513		103.95
119.0733		144.25
120.0603		177.68
120.0801	1	5776.02
120.9594	2	71.3
121.0823	1	662.28

Compound Label	Name	*m/z*	RT	Algorithm	Mass
Cpd 9: Gabapentin; C9 H17 N O2	**Gabapentin**	172.1318	2.006	Auto MS/MS	171.1245

MS Spectrum

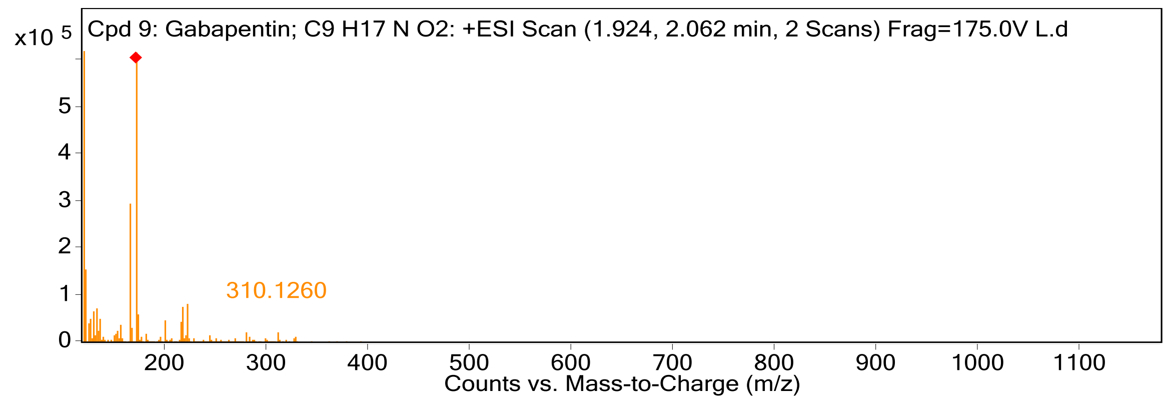

MS Zoomed Spectrum

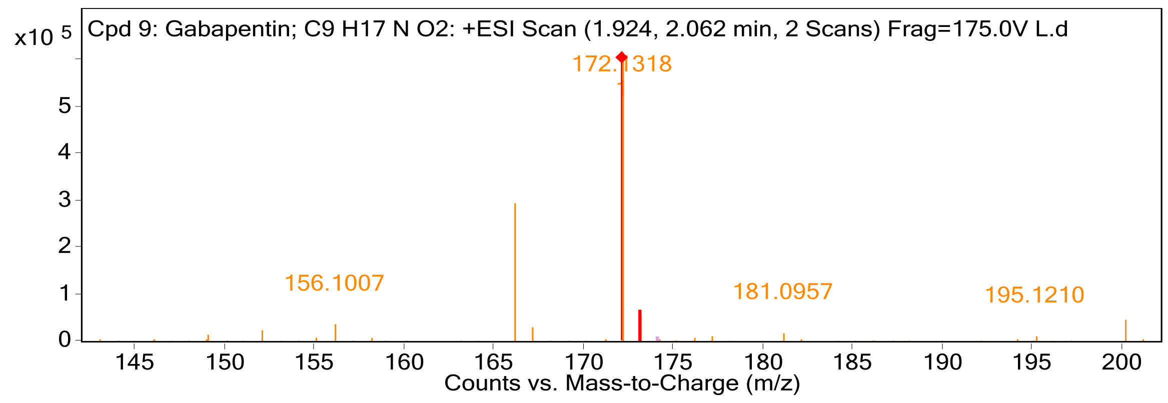

MS Spectrum Peak List

m/z	*Calc m/z*	Diff(ppm)	z	Abund	Formula	Ion
120.08			1	1840847.63		
121.0833			1	156116.39		
126.1269			1	52079.98		
129.9126				66200.61		
131.9099				71674.7		
166.0851			1	294911.47		
172.1318	172.1332	8.32	1	616171.88	C9 H17 N O2	(M+H)+
173.1349	173.1364	8.93	1	60945.78	C9 H17 N O2	(M+H)+
217.1029			1	77146.29		
222.1105			1	82058.63		

MSMS Spectrum

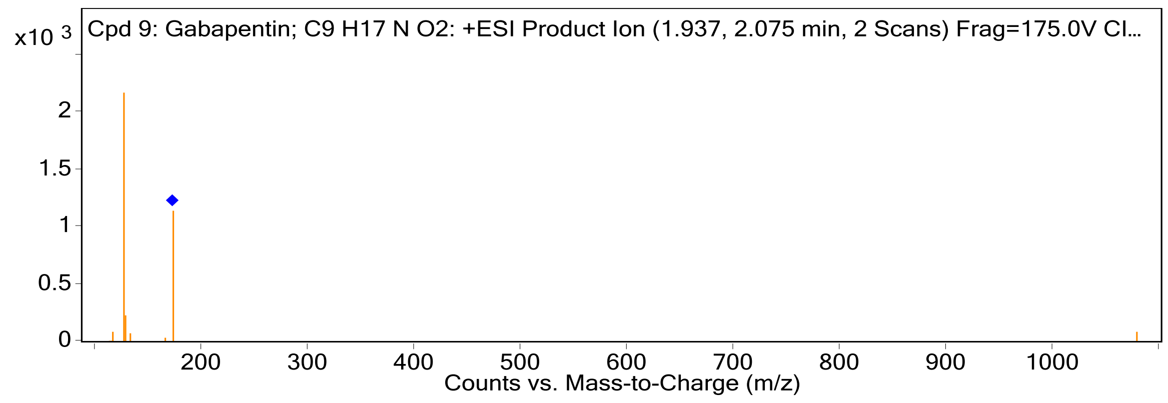

MS/MS Spectrum Peak List

m/z	z	Abund
116.0653		89.42
126.1086		153.47
126.1262	1	2176.27
127.1294	1	231.9
131.9236		79.03
172.0893		79.63
172.1306	1	1147.02
173.068	2	65.7
173.1352	1	149.42
1077.0452		86.11

Compound Structure

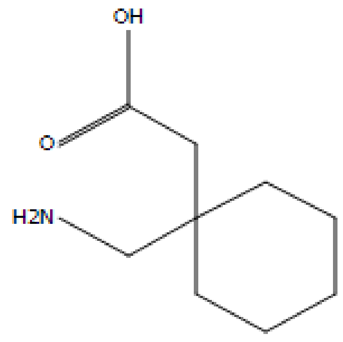

Compound Label	Name	*m/z*	RT	Algorithm	Mass
Cpd 10: 1-Phenylbiguanide; C8 H11	**1-Phenylbiguanide**	200.0901	2.014	Auto MS/MS	177.101

MS Spectrum

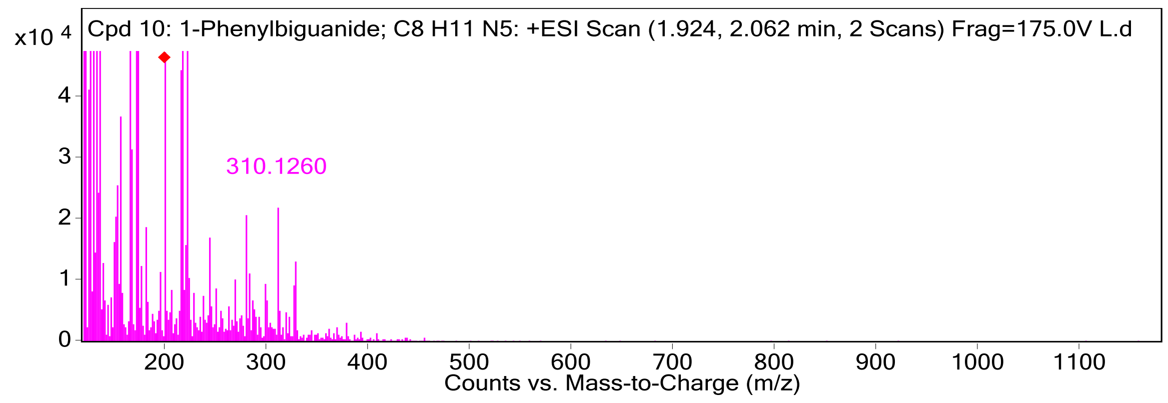

MS Zoomed Spectrum

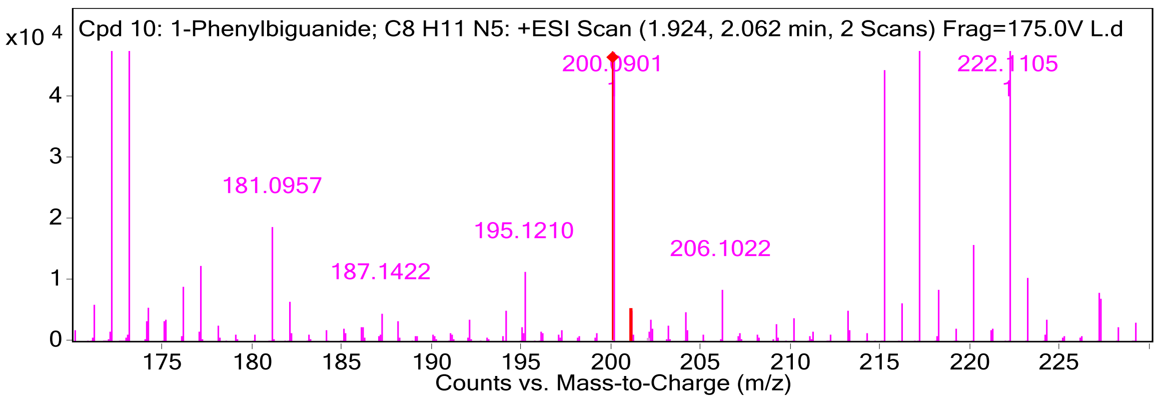

MS Spectrum Peak List

m/z	*Calc m/z*	Diff(ppm)	z	Abund	Formula	Ion
120.08			1	1840847.63		
121.0833			1	156116.39		
129.9126				66200.61		
131.9099				71674.7		
166.0851			1	294911.47		
172.1318			1	616171.88		
200.0901	200.0907	3.01	1	47390.32	C8 H11 N5	(M+Na)+
201.0936	201.093	-3.33	1	5108.17	C8 H11 N5	(M+Na)+
217.1029			1	77146.29		
222.1105			1	82058.63		

MSMS Spectrum

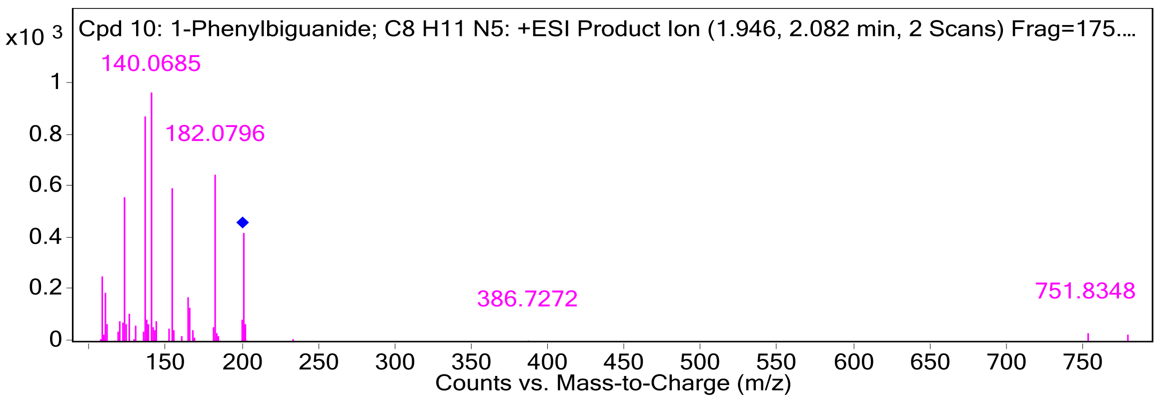

MS/MS Spectrum Peak List

m/z	z	Abund
108.0841		254.27
110.059		189.02
122.0589	1	563.86
136.0743	1	871.67
140.0685		968.31
154.086	1	595.36
164.0701		171.18
165.0526		132.02
182.0796		648.33
200.0909	1	420.44

Compound Structure

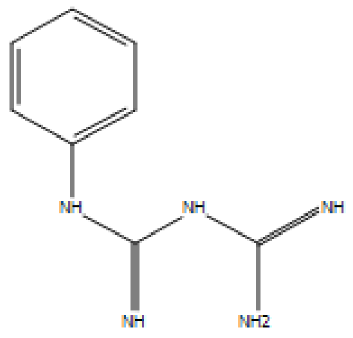

Compound Label	*m/z*	RT	Algorithm
Compound 11	120.08	2.135	Auto MS/MS

MS Spectrum

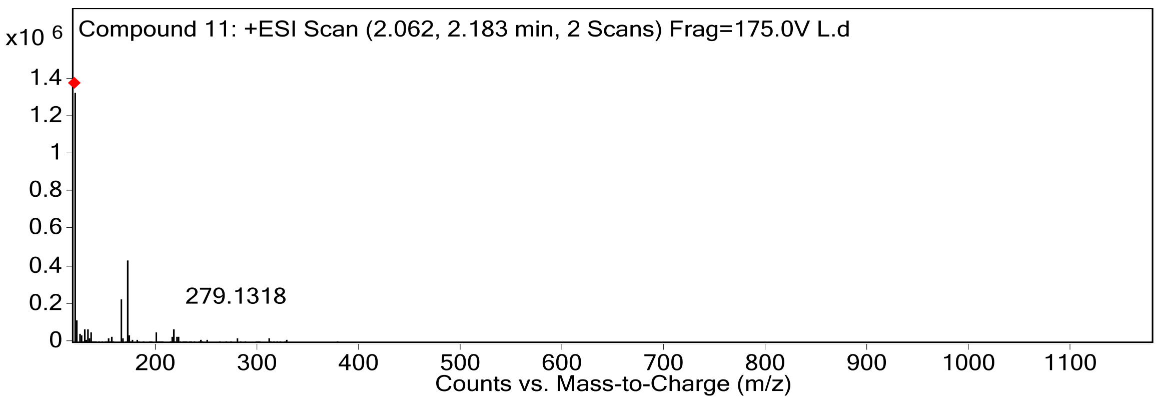

MS Zoomed Spectrum

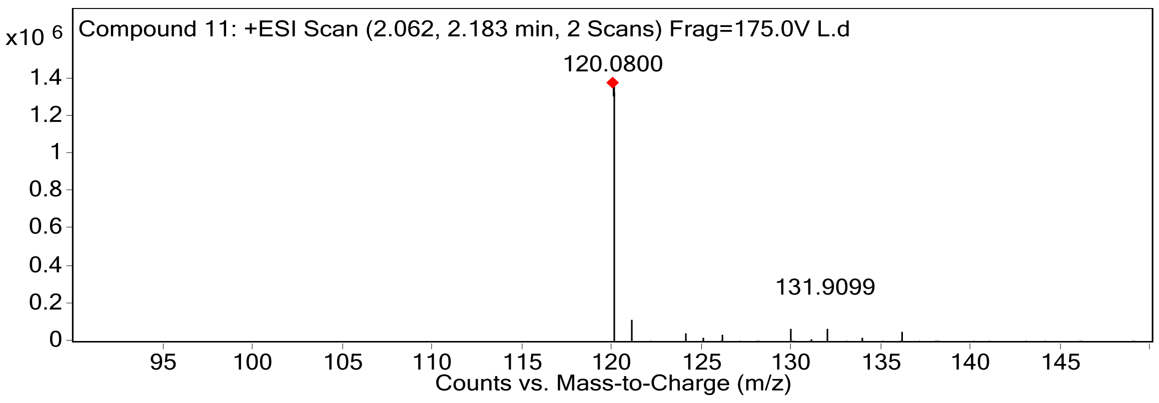

MS Spectrum Peak List

m/z	z	Abund
120.08	1	1327515
121.0833	1	119020.98

124.0859		44451.23
129.9126		68825.41
131.9099		70499.03
136.0615	1	51968.54
166.0849	1	227314.38
172.1318	1	433982.69
200.0902	1	53941.06
217.1029	1	72487.91

MSMS Spectrum

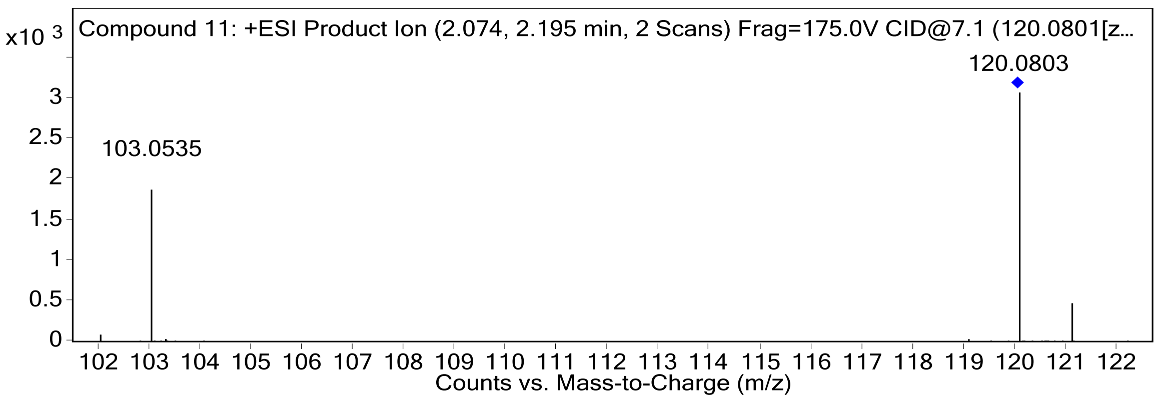

MS/MS Spectrum Peak List

m/z	z	Abund
102.0445		93.8
103.036		139.02
103.0535	1	1875.66
103.3152		32.09
119.0826		37.93
119.4932		26.54
119.8552		24.67
120.0803	1	3080.67
121.0822	1	476.93
121.132		25.5

Compound Label	*m/z*	RT	Algorithm
Compound 12	120.0802	2.452	Auto MS/MS

MS Spectrum

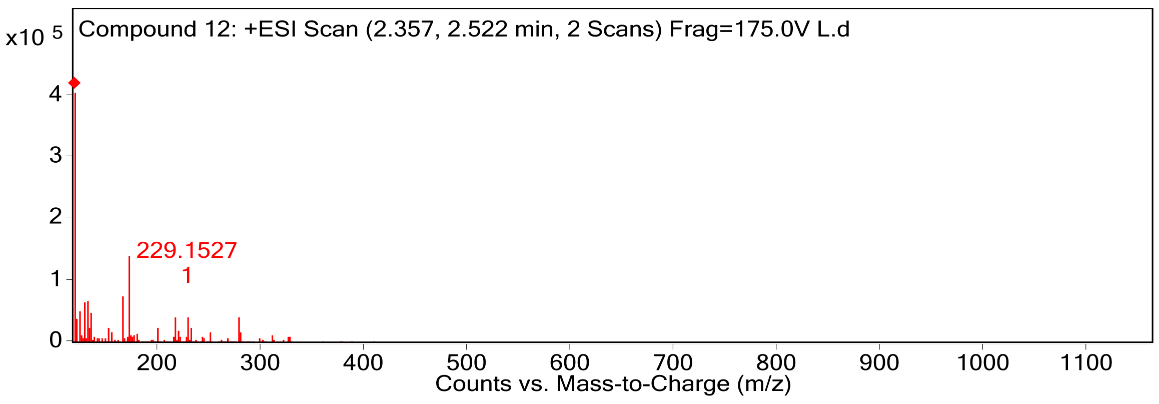

MS Zoomed Spectrum

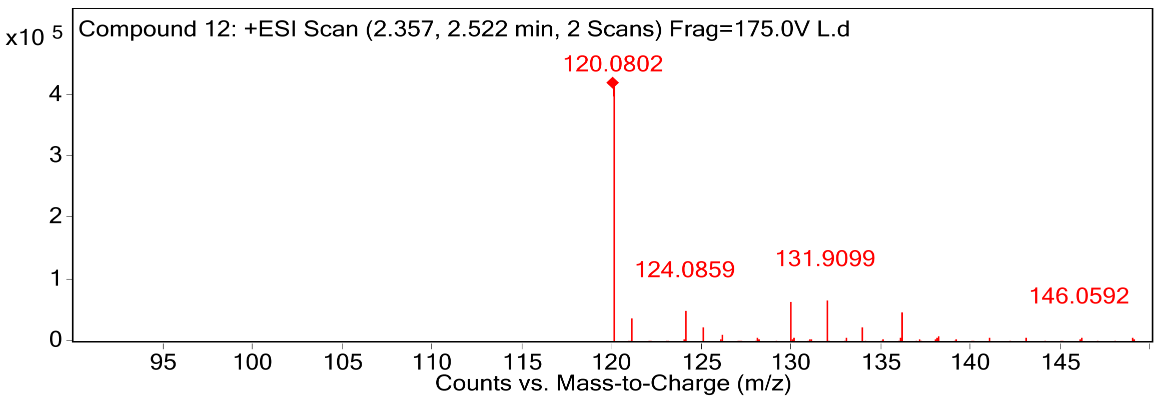

MS Spectrum Peak List

m/z	z	Abund
120.0802	1	404426.56
121.0832	1	38257.13
124.0859		49797.87
129.9126		66277.69
131.9099		67777.02
136.0607	1	48569.41
166.0849	1	75352.56
172.1316	1	139316.98
229.1527	1	41211.58
279.1315		40736.58

MSMS Spectrum

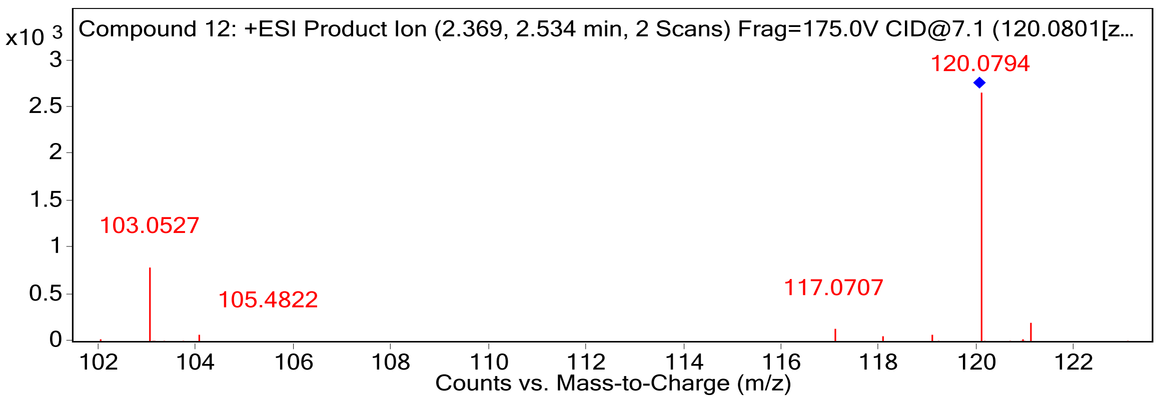

MS/MS Spectrum Peak List

m/z	z	Abund
103.037		58.34
103.0527	1	800.6
104.0556	1	86.18

m/z	z	Abund
117.0707	1	135.84
118.0402		68.92
118.0637	1	36.54
119.0725	1	84.72
120.0794	1	2662.47
121.0644		94.99
121.082	1	212.42

Compound Label	Name	*m/z*	RT	Algorithm	Mass
Cpd 13: Methyl N-methylanthranilate; C9 H11 N O2	**Methyl N-methylanthranilate**	188.0691	3.265	Auto MS/MS	165.08

MS Spectrum

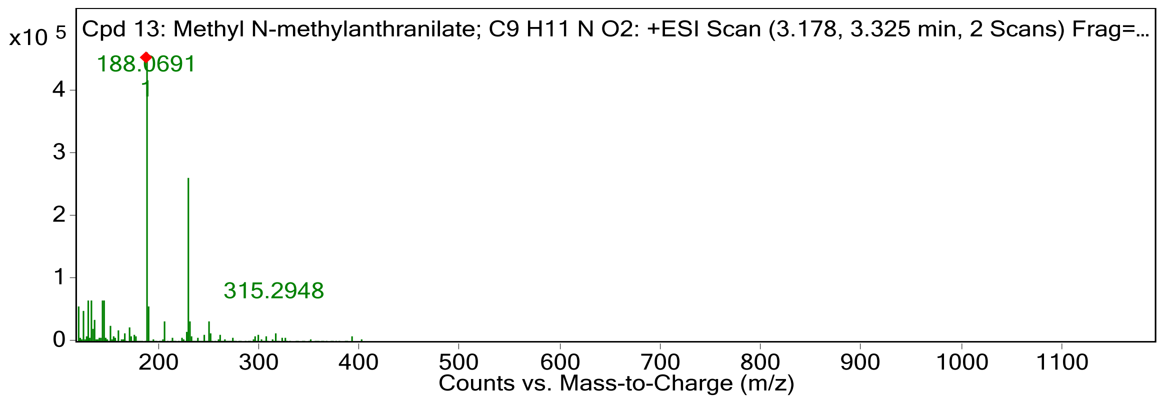

MS Zoomed Spectrum

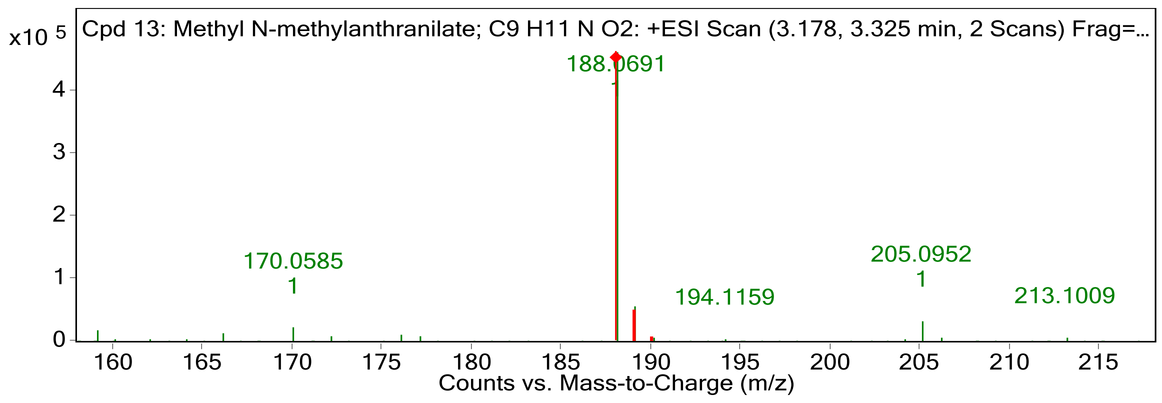

MS Spectrum Peak List

m/z	*Calc m/z*	Diff(ppm)	z	Abund	Formula	Ion
120.0799			1	57291.44		
124.0858			1	50315.51		
129.9126				66473.5		
131.9099				66210.7		
144.0796			1	65390.96		
146.0589			1	67067.8		
188.0691	188.0682	-4.98	1	461293.44	C9 H11 N O2	(M+Na)+
189.0723	189.0714	-4.66	1	55878.99	C9 H11 N O2	(M+Na)+
190.0786	190.0736	-26.37	1	7224.64	C9 H11 N O2	(M+Na)+
229.1527			1	261272.34		

MSMS Spectrum

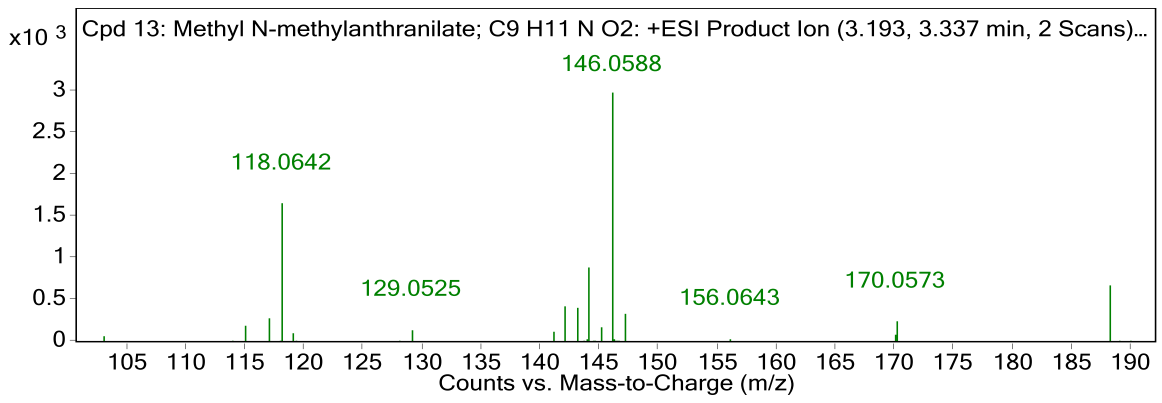

MS/MS Spectrum Peak List

m/z	z	Abund
115.0538		195.55
117.0673		290.39
118.0642	1	1655.48
142.0628		435.35
143.0731		411.41
144.079	1	897.6
146.0588	1	2981.31
147.0614	1	335.54
170.0573		241.57
188.0685		673.04

Compound Structure

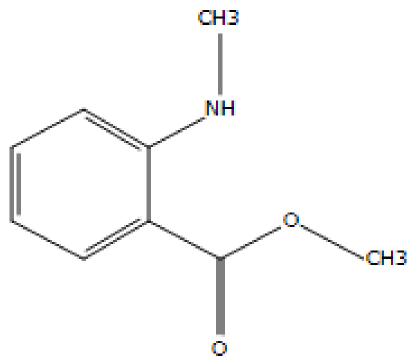

Compound Label	Name	*m/z*	RT	Algorithm	Mass
Cpd 14: L-Tryptophan; C11 H12 N2 O2	**L-Tryptophan**	205.0952	3.286	Auto MS/MS	204.0879

MS Spectrum

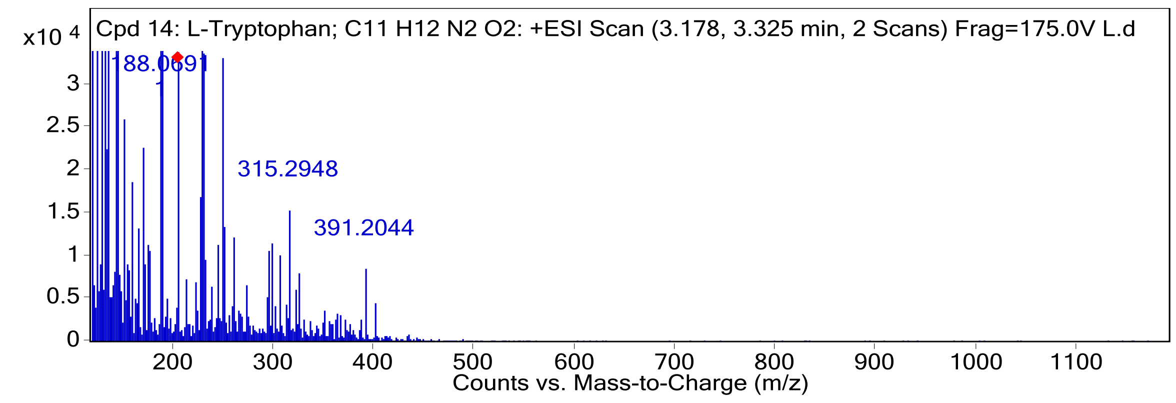

MS Zoomed Spectrum

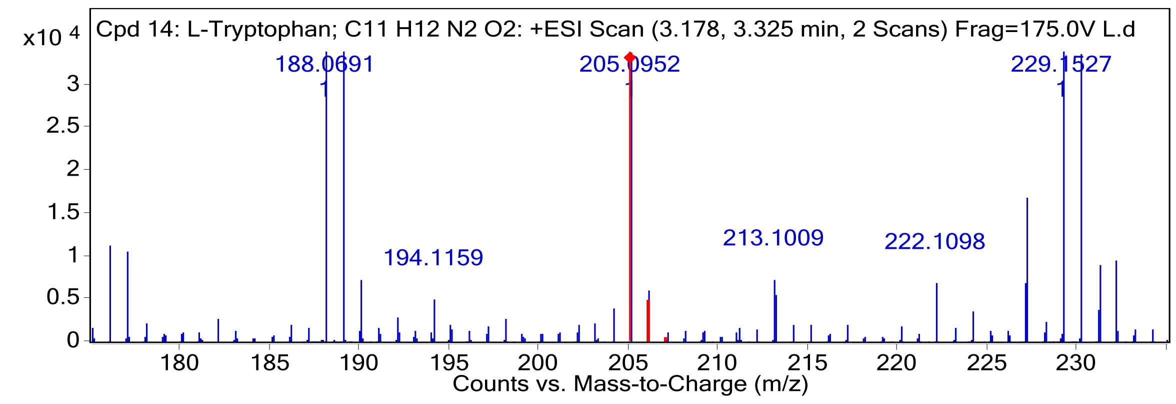

MS Spectrum Peak List

m/z	*Calc m/z*	Diff(ppm)	z	Abund	Formula	Ion
120.0799			1	57291.44		
129.9126				66473.5		
131.9099				66210.7		
144.0796			1	65390.96		
146.0589			1	67067.8		
188.0691			1	461293.44		
205.0952	205.0972	9.49	1	33743.45	C11 H12 N2 O2	(M+H)+
206.0983	206.1002	9.29	1	6124.57	C11 H12 N2 O2	(M+H)+
207.0978	207.1026	23	1	1163.97	C11 H12 N2 O2	(M+H)+
229.1527			1	261272.34		

MSMS Spectrum

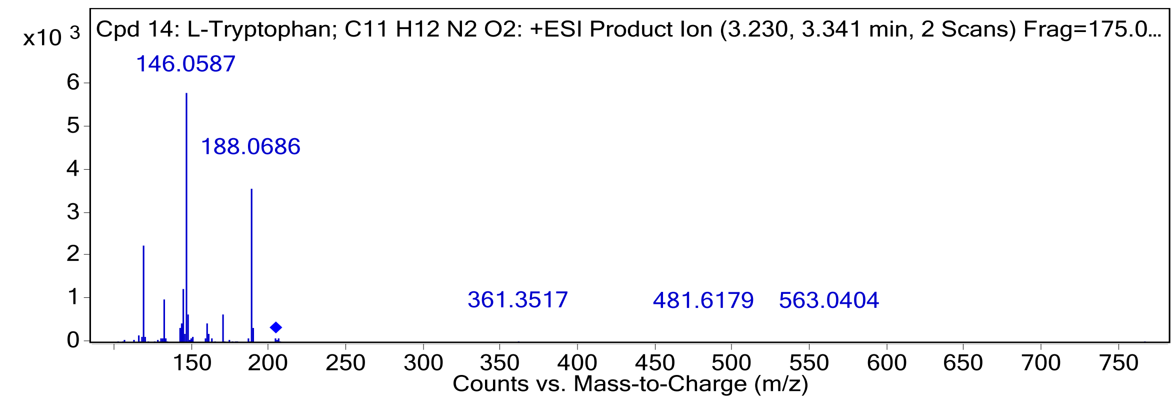

MS/MS Spectrum Peak List

m/z	z	Abund
118.0637	1	2253.11
132.0796	1	1008.48
143.0722		436.64
144.0796	1	1251.21
146.0587	1	5802.77
147.0634	1	675.75
159.087	1	437.43
170.0581		667.06
188.0686	1	3592.04
189.0734	1	348.84

Compound Structure

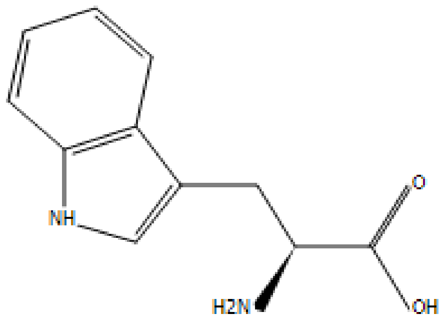

Compound Label	Name	*m/z*	RT	Algorithm	Mass
Cpd 15: 6-Methylquinoline; C10 H9 N	**6-Methylquinoline**	144.0795	3.33	Auto MS/MS	143.0722

MS Spectrum

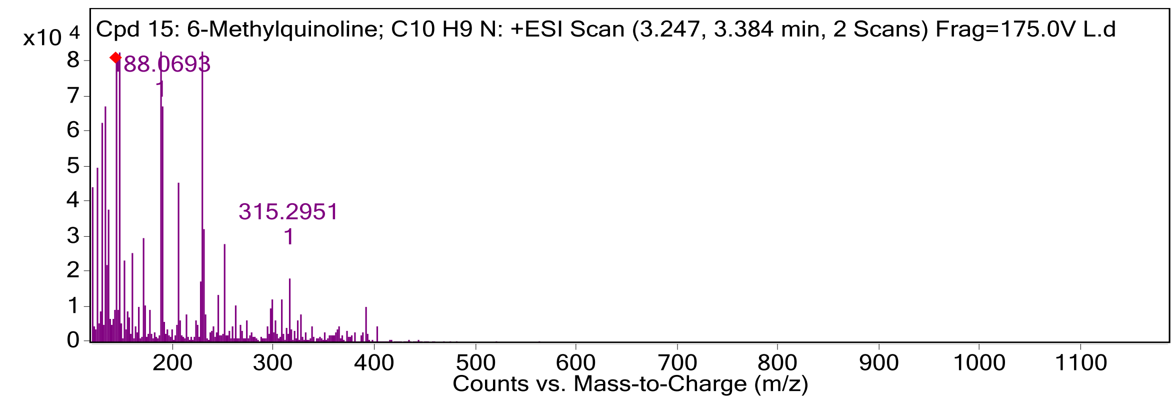

MS Zoomed Spectrum

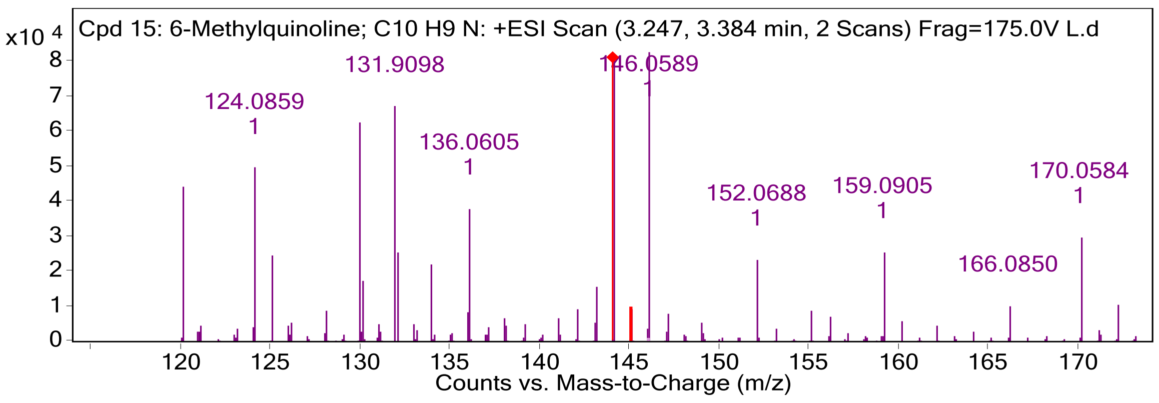

MS Spectrum Peak List

m/z	*Calc m/z*	Diff(ppm)	z	Abund	Formula	Ion
124.0859			1	49752.84		
129.9125				62527.44		
131.9098				67507.48		
144.0795	144.0808	9.07	1	81959.06	C10 H9 N	(M+H)+
145.0828	145.084	7.78	1	9498.06	C10 H9 N	(M+H)+
146.0589			1	82747.5		
188.0693			1	581243.38		
189.0725			1	67557.7		
205.0955			1	45515.89		
229.1527			1	245717.36		

MSMS Spectrum

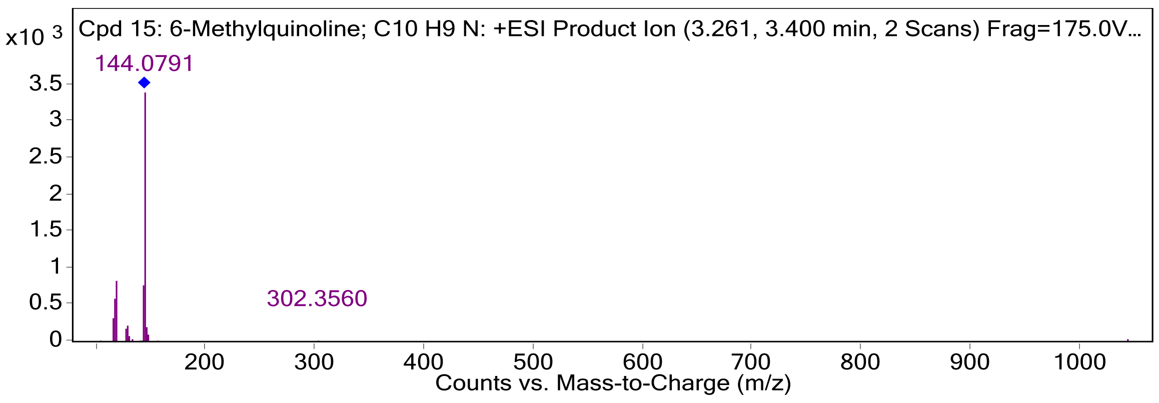

MS/MS Spectrum Peak List

m/z	z	Abund
115.0521		322.96
116.0471		205.06
117.0542		225.9
117.0685		595.15
118.0644		822.97
127.0507		186.92
128.0471		222.66
143.0709		777.75
144.0791	1	3395.52
145.0812	1	209.49

Compound Structure

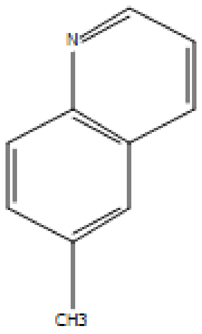

Compound Label	Name	*m/z*	RT	Algorithm	Mass
Cpd 16: Isocarbostyril; C9 H7 N O	**Isocarbostyril**	146.0589	3.331	Auto MS/MS	145.0517

MS Spectrum

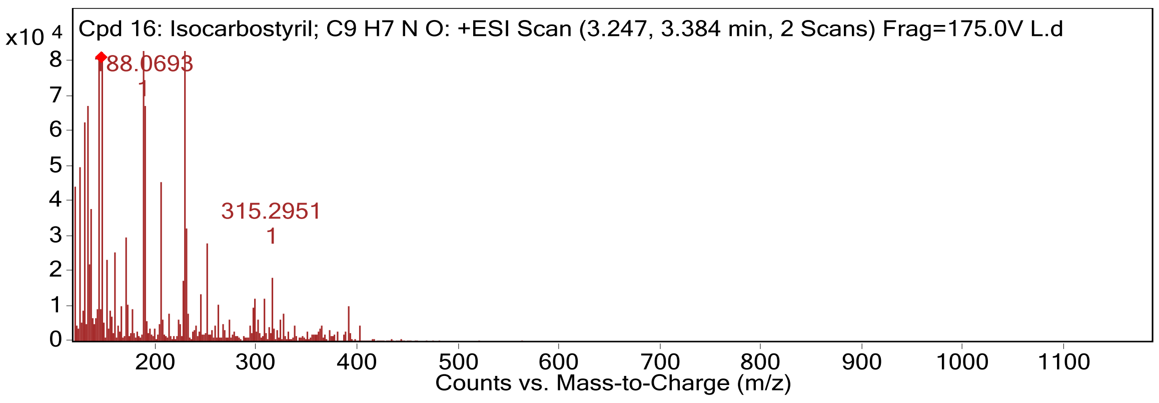

MS Zoomed Spectrum

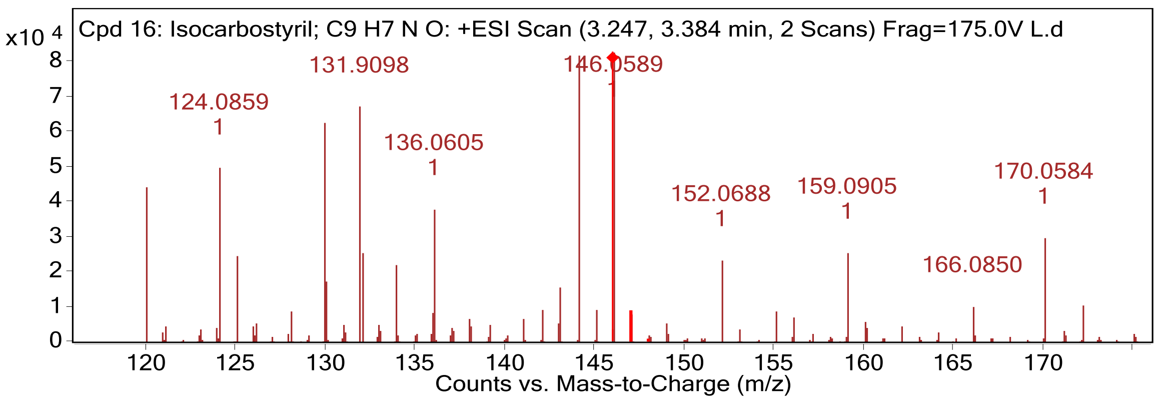

MS Spectrum Peak List

m/z	*Calc m/z*	Diff(ppm)	z	Abund	Formula	Ion
124.0859			1	49752.84		

129.9125				62527.44		
131.9098				67507.48		
144.0795			1	81959.06		
146.0589	146.06	8.1	1	82747.5	C9 H7 N O	(M+H)+
147.0618	147.0632	9.23	1	7965.14	C9 H7 N O	(M+H)+
148.0744	148.0657	-58.59	1	1497.91	C9 H7 N O	(M+H)+
188.0693			1	581243.38		
189.0725			1	67557.7		
229.1527			1	245717.36		

MSMS Spectrum

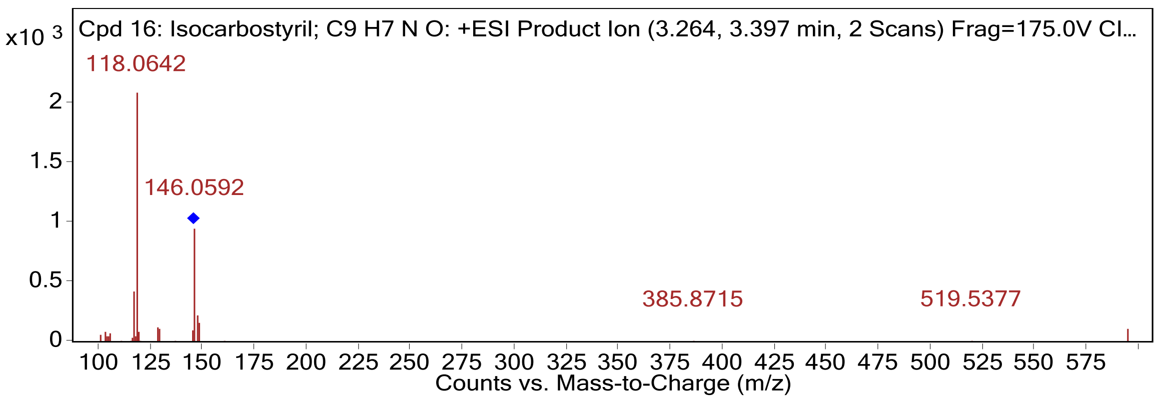

MS/MS Spectrum Peak List

m/z	z	Abund
117.058		428
118.0405		128.65
118.0642	1	2096.43
128.0468		125.37
129.062		107.51
145.9665		173.69
146.0592		950.13
147.0469		221.81
147.9594		157.63
593.8115		116.5

Compound Structure

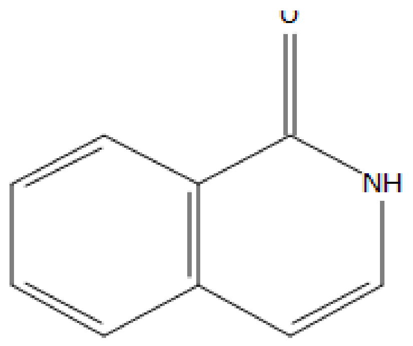

Compound Label	Name	*m/z*	RT	Algorithm	Mass
Cpd 17: Methyl N-methylanthranilate; C9 H11 N O2	**Methyl N-methylanthranilate**	188.0692	3.536	Auto MS/MS	165.08

MS Spectrum

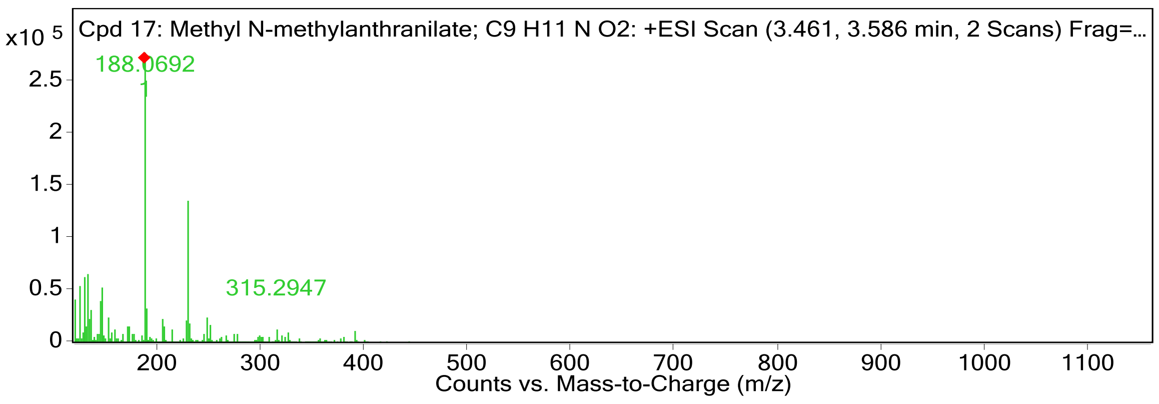

MS Zoomed Spectrum

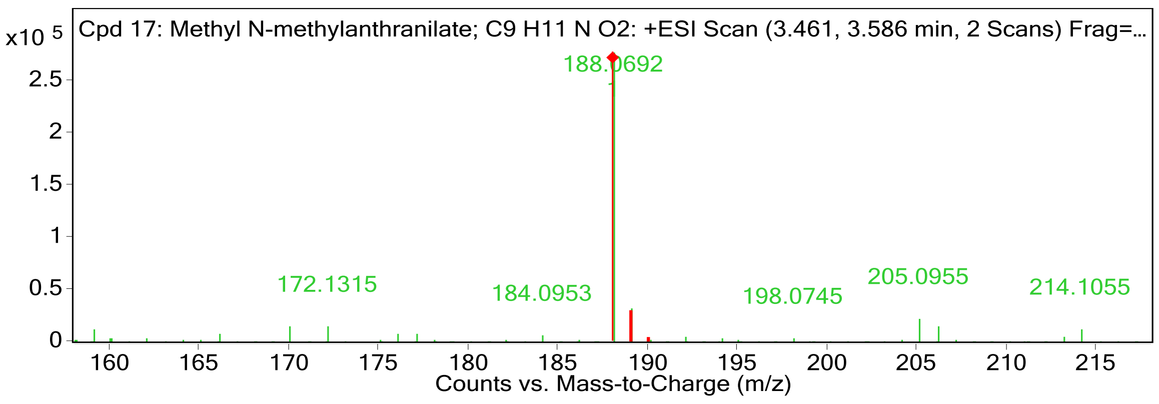

MS Spectrum Peak List

m/z	*Calc m/z*	Diff(ppm)	z	Abund	Formula	Ion
120.0798			1	41281.59		
124.0859				53493.8		
129.9125				63065.63		
131.9098				65407.93		
144.0797			1	40517.19		
146.0589			1	52949.3		
188.0692	188.0682	-5.05	1	276665.13	C9 H11 N O2	(M+Na)+
189.0727	189.0714	-6.86	1	32885.1	C9 H11 N O2	(M+Na)+
190.075	190.0736	-7.69	1	3246.34	C9 H11 N O2	(M+Na)+
229.1527			1	136097.03		

MSMS Spectrum

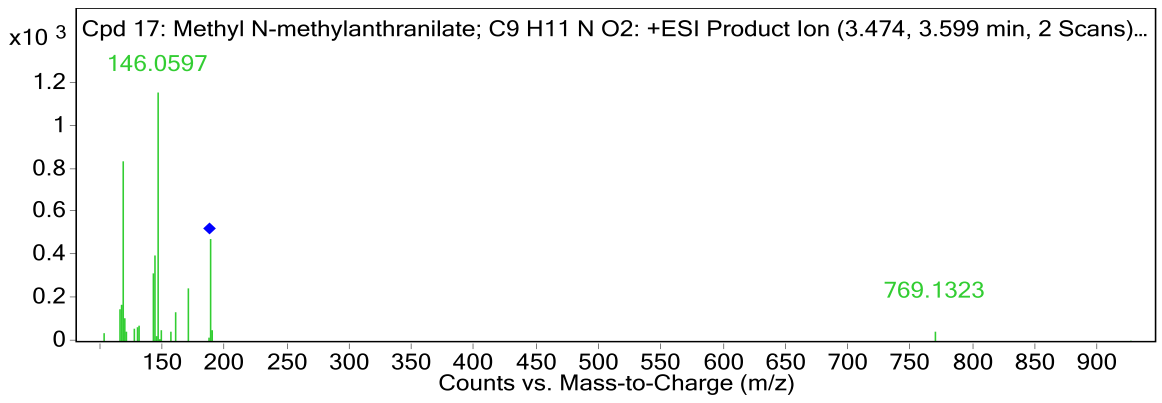

MS/MS Spectrum Peak List

m/z	z	Abund
115.0536		149.35
117.0682		170
118.0643	1	838.89
142.063		320.57
143.0719		389.91
144.0792	1	405.15
146.0354		147.91
146.0597	1	1159.54
170.0601		248.81
188.0686		481.72

Compound Structure

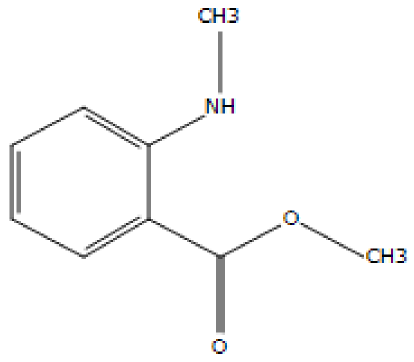

Compound Label	Name	*m/z*	RT	Algorithm	Mass
Cpd 18: Isocarbostyril; C9 H7 N O	**Isocarbostyril**	146.0589	3.69	Auto MS/MS	145.0517

MS Spectrum

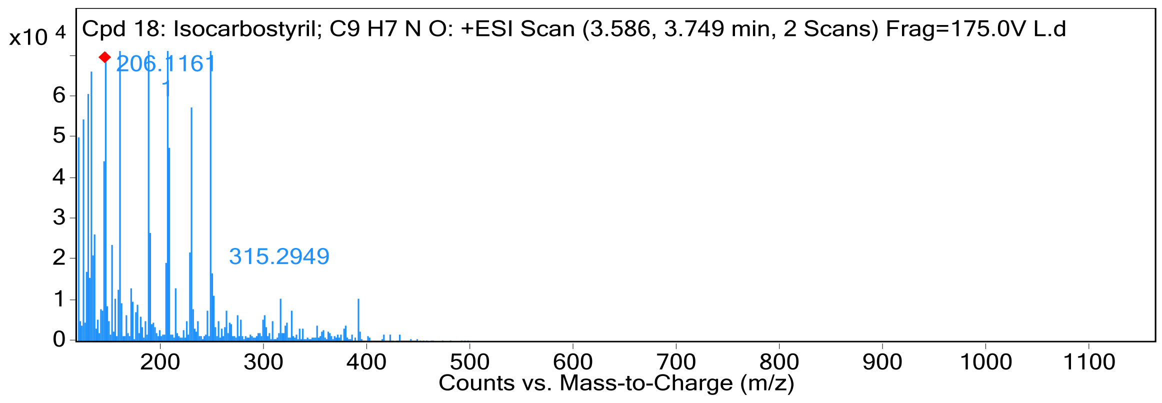

MS Zoomed Spectrum

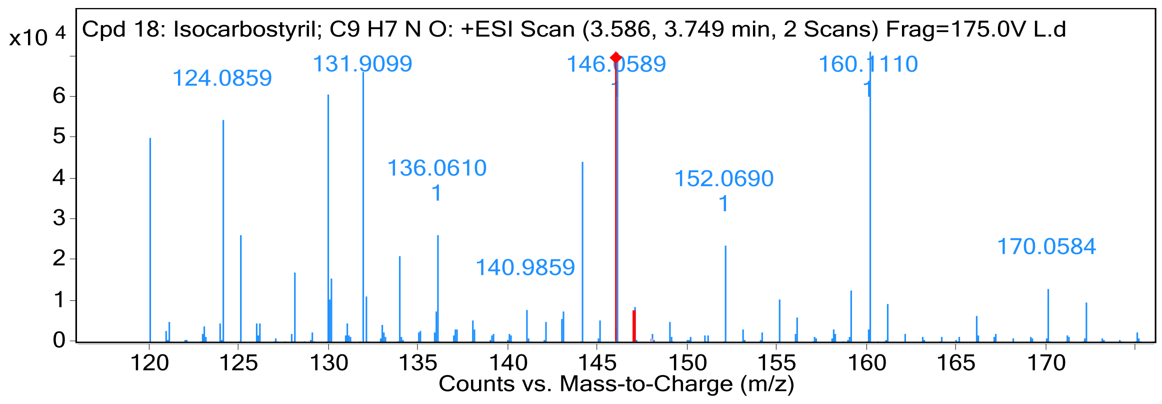

MS Spectrum Peak List

m/z	*Calc m/z*	Diff(ppm)	z	Abund	Formula	Ion
124.0859				54643.33		
129.9126				60856.05		
131.9099				66128.95		
146.0589	146.06	7.51	1	70942.85	C9 H7 N O	(M+H)+
147.062	147.0632	8.13	1	8823.69	C9 H7 N O	(M+H)+
160.111			1	79819.45		
188.0691			1	240896.25		
206.1161			1	402803.75		
229.1526			1	57580.45		
248.1735			1	99473.23		

MSMS Spectrum

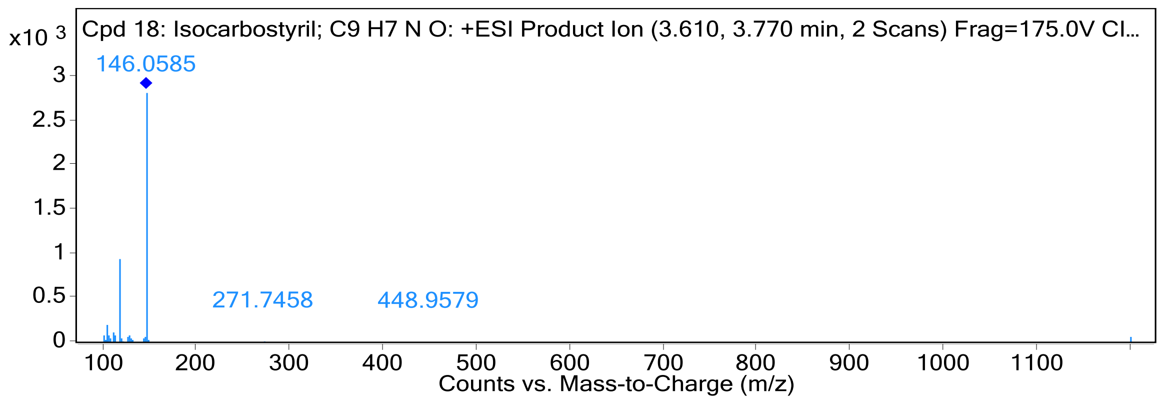

MS/MS Spectrum Peak List

m/z	z	Abund
104.0467		195.96

110.9984		111.01
117.0588		197.48
118.0639		940.18
128.0483		83.32
145.9667		418.96
146.0585	1	2820.54
147.0233		135.16
147.0633	1	382.02
147.0828		256.73

Compound Structure

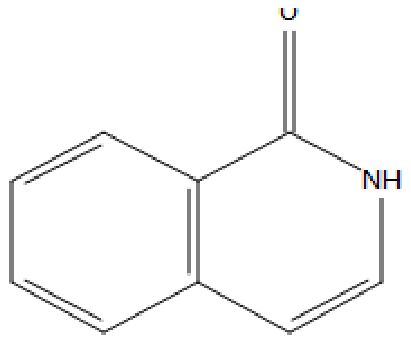

Compound Label	Name	*m/z*	RT	Algorithm	Mass
Cpd 19: Methyprylon; C10 H17 N O2	**Methyprylon**	206.1161	3.69	Auto MS/MS	183.1269

MS Spectrum

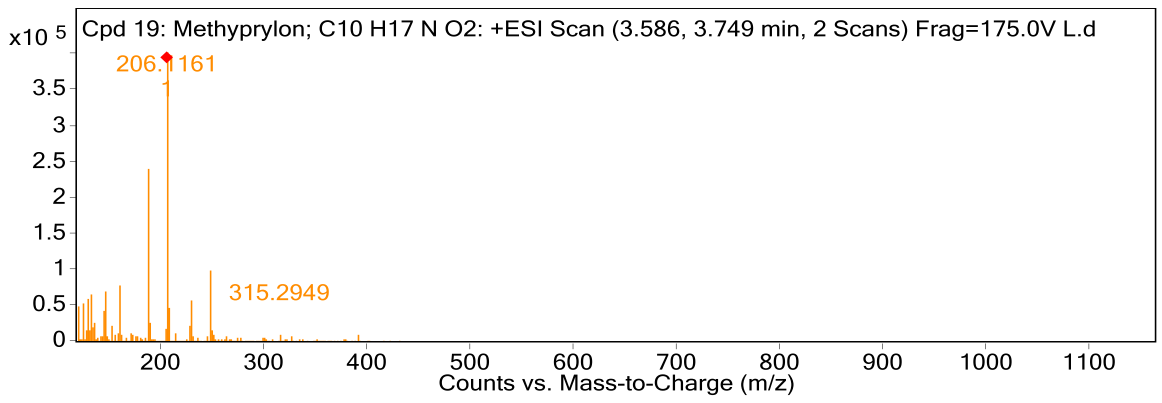

MS Zoomed Spectrum

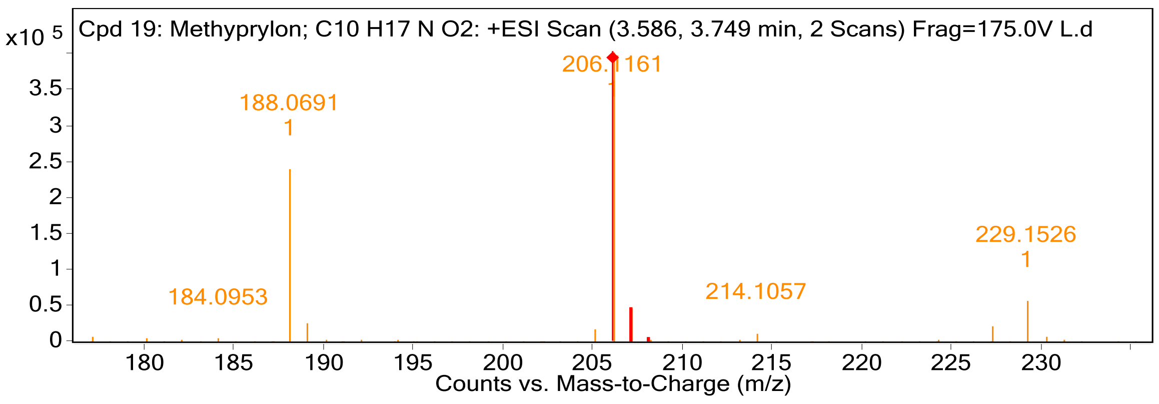

MS Spectrum Peak List

m/z	*Calc m/z*	Diff(ppm)	z	Abund	Formula	Ion
129.9126				60856.05		
131.9099				66128.95		
146.0589			1	70942.85		
160.111			1	79819.45		
188.0691			1	240896.25		
206.1161	206.1151	-4.85	1	402803.75	C10 H17 N O2	(M+Na)+
207.1187	207.1184	-1.58	1	47526.13	C10 H17 N O2	(M+Na)+
208.1229	208.1207	-10.58	1	5021.11	C10 H17 N O2	(M+Na)+
229.1526			1	57580.45		
248.1735			1	99473.23		

MSMS Spectrum

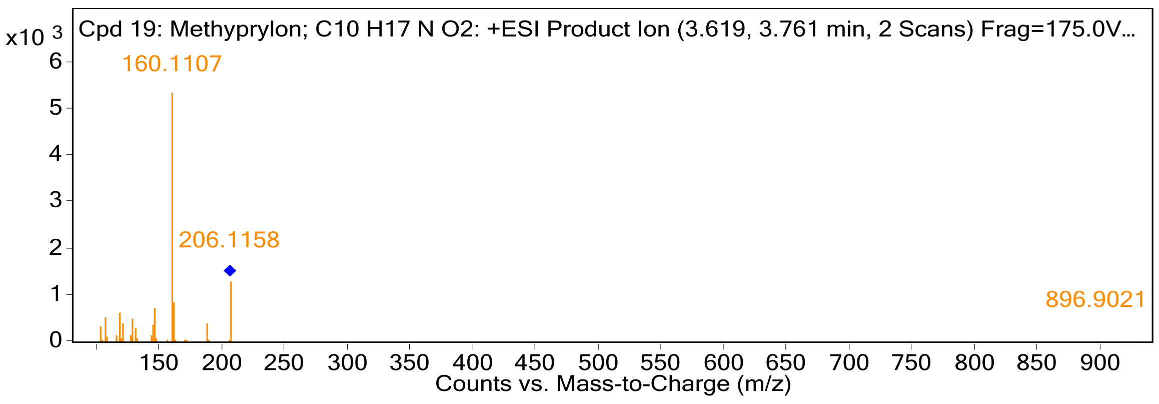

MS/MS Spectrum Peak List

m/z	z	Abund
107.0489		545.68
118.0646		651.15
120.0807	1	408.21
128.0611		503.83
144.0794		392.06
146.0594	1	753.45
160.1107	1	5361.37
161.1131	1	867.62
188.0719	1	427.15
206.1158		1309.85

Compound Structure

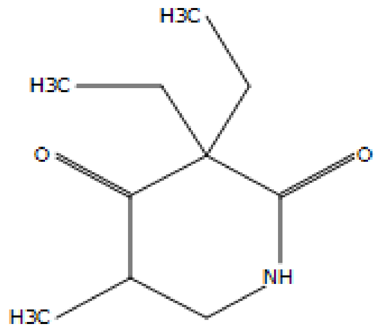

Compound Label	Name	*m/z*	RT	Algorithm	Mass
Cpd 20: 6-Methylquinoline; C10 H9 N	**6-Methylquinoline**	144.0795	3.744	Auto MS/MS	143.0723

MS Spectrum

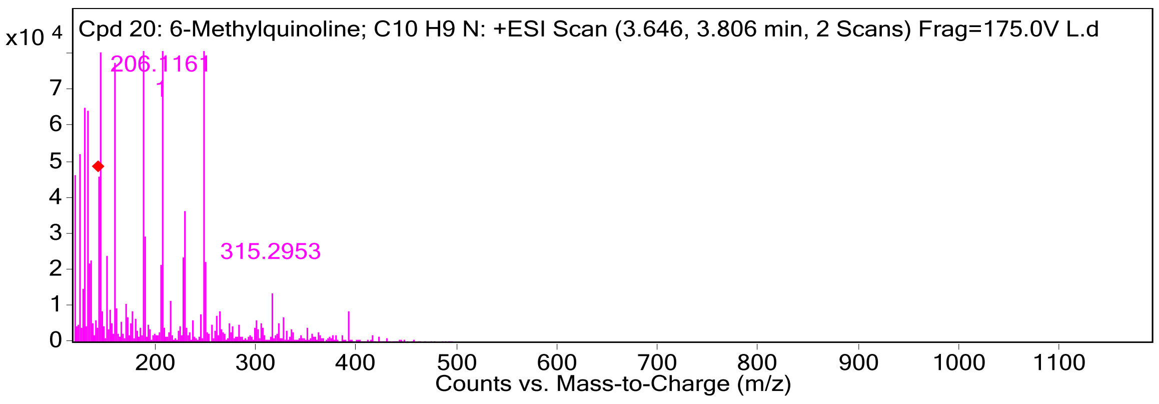

MS Zoomed Spectrum

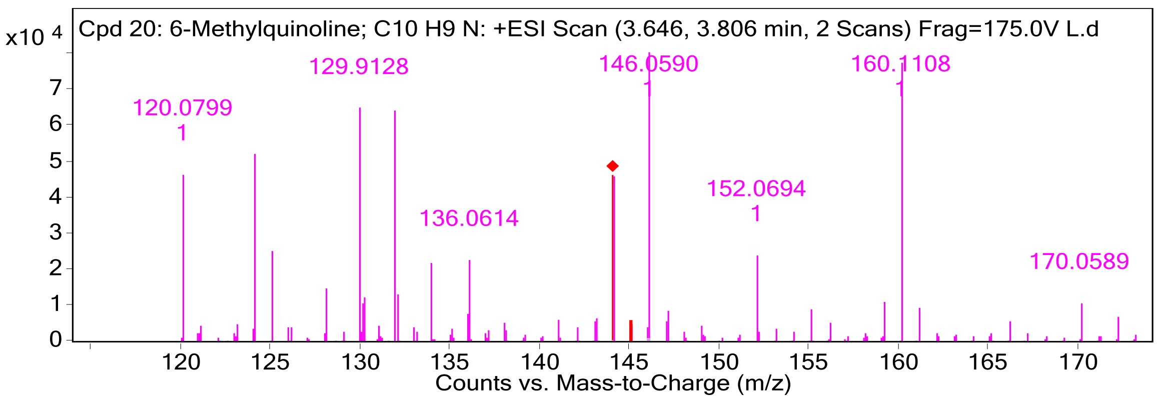

MS Spectrum Peak List

m/z	*Calc m/z*	Diff(ppm)	z	Abund	Formula	Ion

124.0859				52288.09		
129.9128				65162.91		
131.9099				64170.45		
144.0795	144.0808	9.01	1	46191.34	C10 H9 N	(M+H)+
145.0837	145.084	1.53	1	5004.45	C10 H9 N	(M+H)+
146.059			1	80537.26		
160.1108			1	77611.69		
188.0693			1	236325.09		
206.1161			1	372761.81		
248.1735			1	119919.38		

MSMS Spectrum

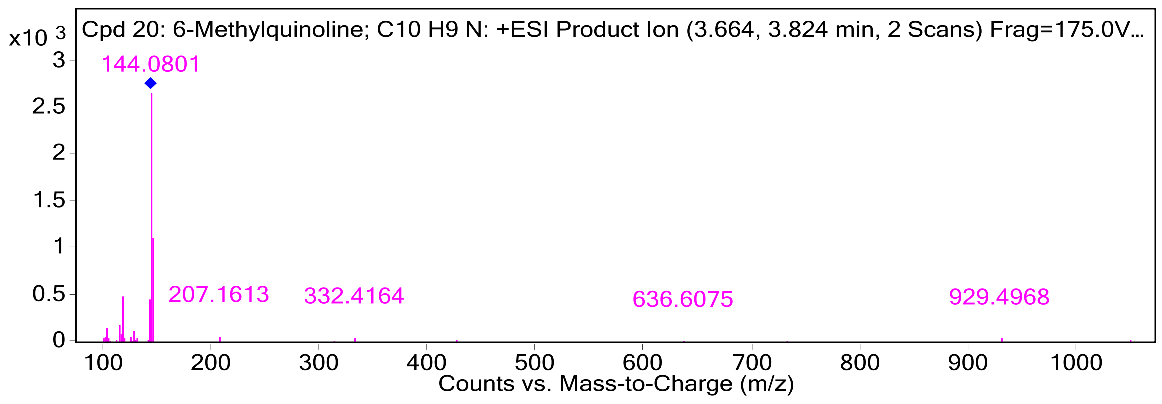

MS/MS Spectrum Peak List

m/z	z	Abund
103.0546		166.32
115.0573		196.64
117.0566		295.13
117.0696		322.23
118.0649	1	491.12
143.0707		464.08
144.0801	1	2663.04
144.0998		208.03
145.083	1	270.62
146.0581		1112.27

Compound Structure

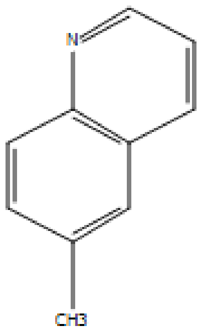

Compound Label	Name	*m/z*	RT	Algorithm	Mass
Cpd 21: Methyl N-methylanthranilate; C9 H11 N O2	**Methyl N-methylanthranilate**	188.0691	3.849	Auto MS/MS	165.0799

MS Spectrum

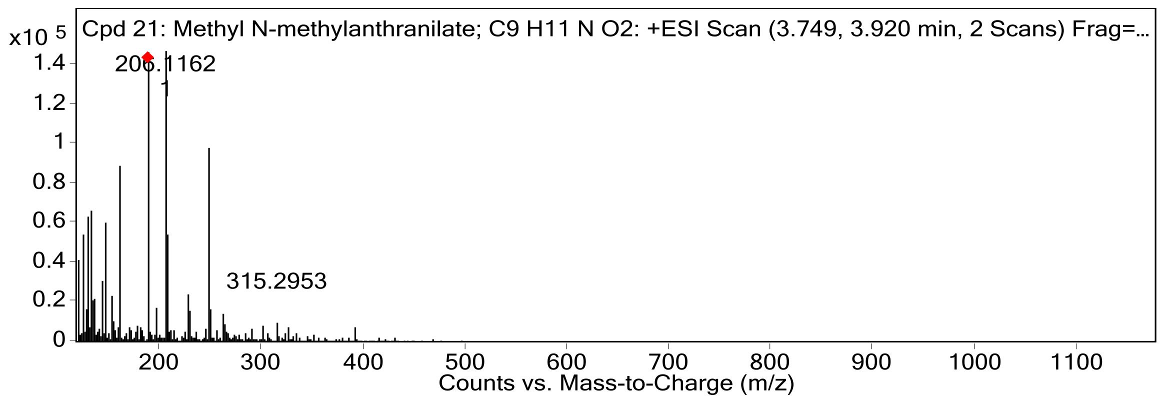

MS Zoomed Spectrum

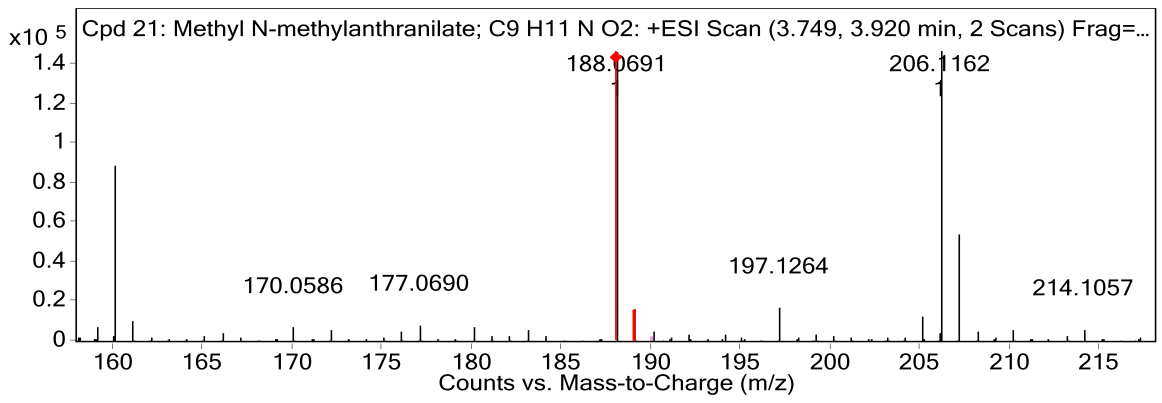

MS Spectrum Peak List

m/z	*Calc m/z*	Diff(ppm)	z	Abund	Formula	Ion
124.086				53800.73		
129.9128				62963.19		
131.9099				66582.36		
146.0589			1	59981.91		
160.111			1	88573.17		
188.0691	188.0682	-4.97	1	145973	C9 H11 N O2	(M+Na)+
189.0723	189.0714	-5.09	1	16524.41	C9 H11 N O2	(M+Na)+
206.1162			1	446268.38		
207.1189			1	54190.68		
248.1735			1	97958.83		

MSMS Spectrum

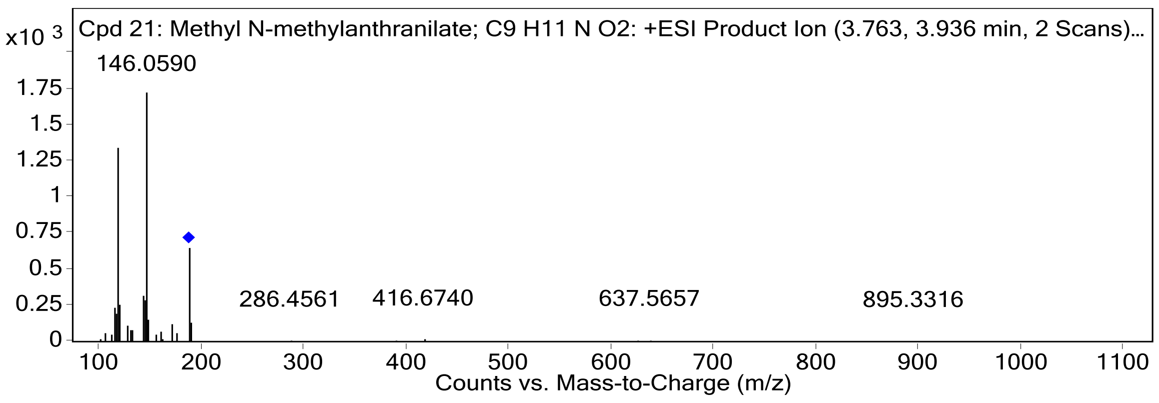

MS/MS Spectrum Peak List

m/z	z	Abund
115.0521		235.04
117.0595		193
118.0637	1	1337.75
119.0678	1	256.22
142.0642		316.6
143.0717		180.91
144.0806	2	284.48
146.059	1	1727.21
147.0615	1	154.02
188.0685	1	648.53

Compound Structure

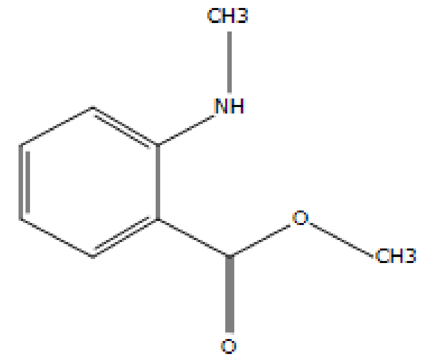

Compound Label	Name	*m/z*	RT	Algorithm	Mass
Cpd 22: Methyprylon; C10 H17 N O2	**Methyprylon**	206.116	4.023	Auto MS/MS	183.1268

MS Spectrum

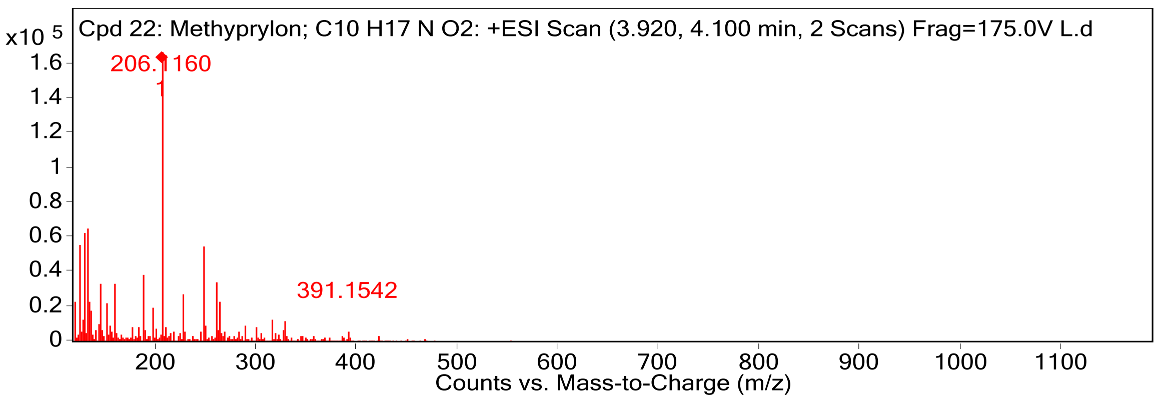

MS Zoomed Spectrum

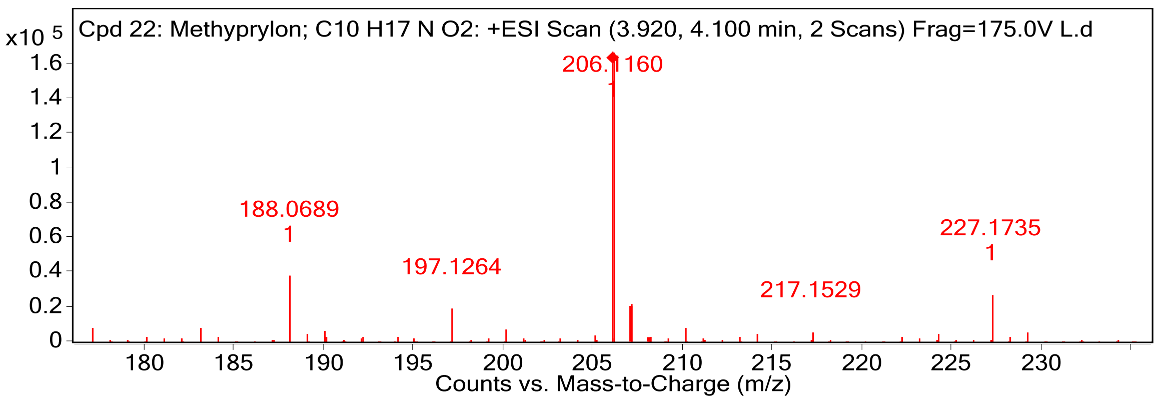

MS Spectrum Peak List

m/z	*Calc m/z*	Diff(ppm)	z	Abund	Formula	Ion
124.0859				56155.08		
129.9127				62928.63		
131.9099				65537.6		
146.0588				33899.33		
188.0689			1	38400.59		
206.116	206.1151	-4.22	1	166538.64	C10 H17 N O2	(M+Na)+
207.1189	207.1184	-2.68	1	22201.46	C10 H17 N O2	(M+Na)+
208.1235	208.1207	-13.36	1	3175.46	C10 H17 N O2	(M+Na)+
248.1735			1	54623.63		
261.1211			1	34608.04		

MSMS Spectrum

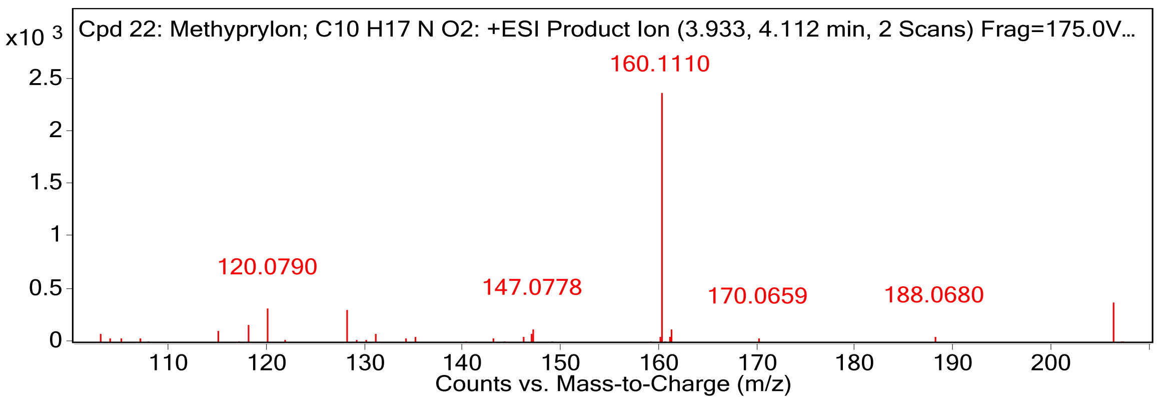

MS/MS Spectrum Peak List

m/z	z	Abund
103.0532		90.18

115.0618		117.53
118.0644		166.14
120.0613		167.44
120.079		323.95
128.06	1	312.66
147.0778		129.6
160.111	1	2370.95
161.1133	1	128.72
206.1152		376.14

Compound Structure

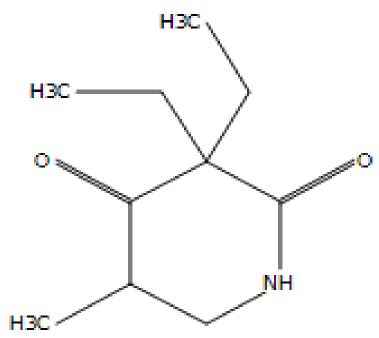

Compound Label	Name	*m/z*	RT	Algorithm	Mass
Cpd 23: Pirbuterol; C12 H20 N2 O3	**Pirbuterol**	263.1367	4.411	Auto MS/MS	240.1476

MS Spectrum

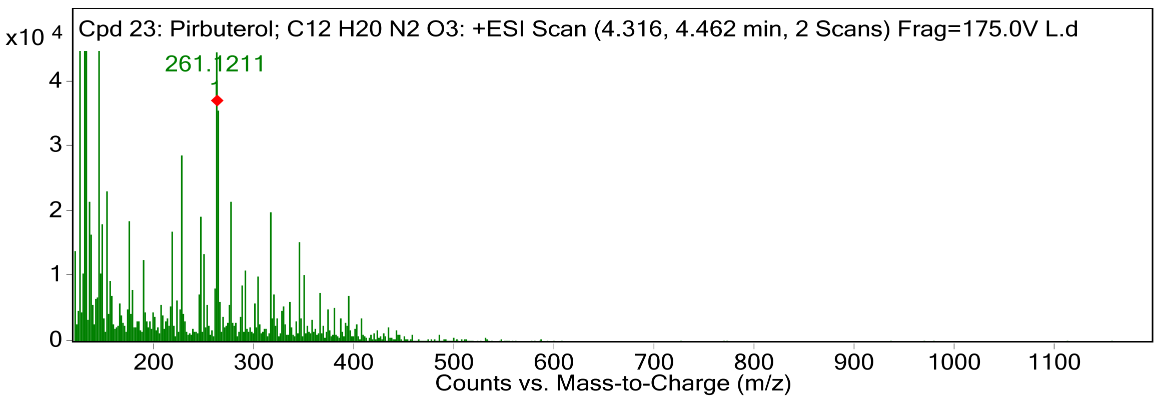

MS Zoomed Spectrum

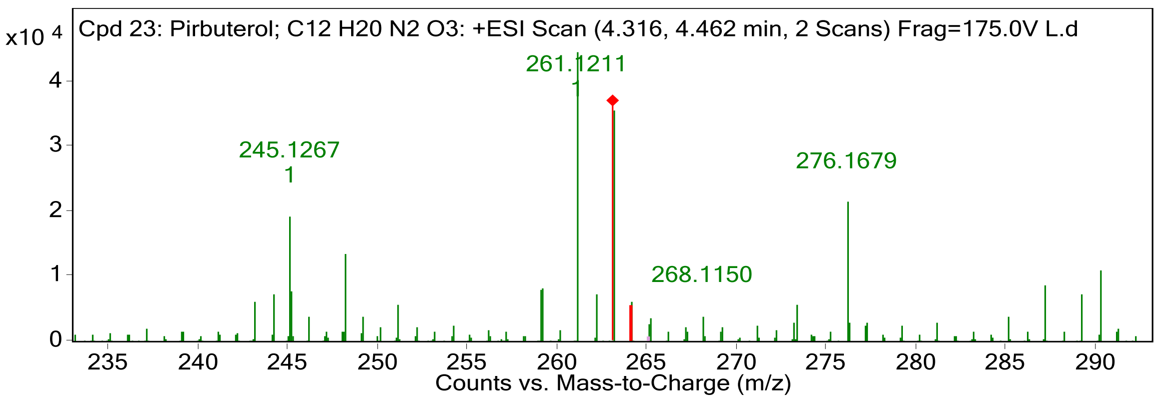

MS Spectrum Peak List

m/z	*Calc m/z*	Diff(ppm)	z	Abund	Formula	Ion
124.0859				54256.97		
125.0704				26180.88		
129.9128				61228.51		
131.9099				61080.51		
144.0795			1	63736.25		
152.0689			1	23247.82		
227.1733			1	28717.04		
261.1211			1	44490.43		
263.1367	263.1366	-0.45	1	35618.88	C12 H20 N2 O3	(M+Na)+
264.1405	264.1397	-3.08	1	6278.73	C12 H20 N2 O3	(M+Na)+

MSMS Spectrum

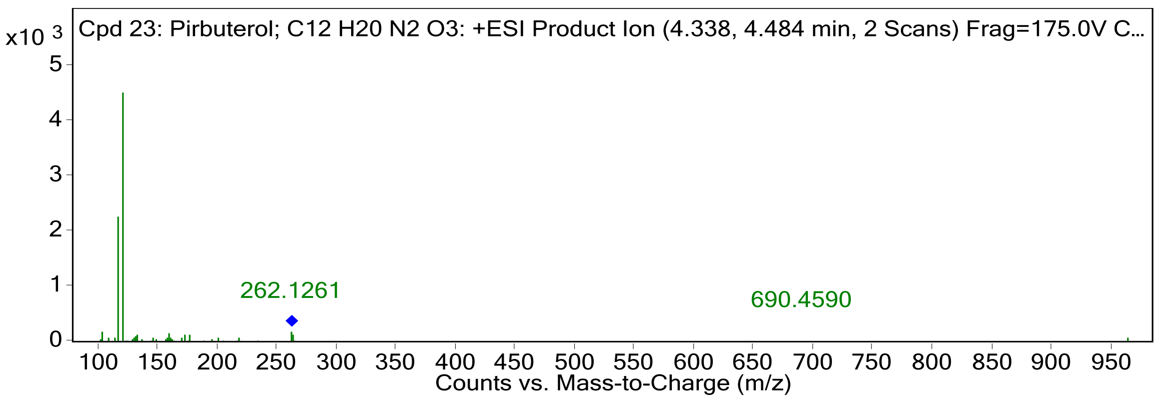

MS/MS Spectrum Peak List

m/z	z	Abund
103.0519		182.91
116.0701	1	2287.12
117.074	1	130.03
120.0802	1	4525.84
121.0826	1	297.32
133.0478		134.15
159.064	1	157.78
172.0709		129.57
262.1261	1	178.26
263.1064		139.81

Compound Structure

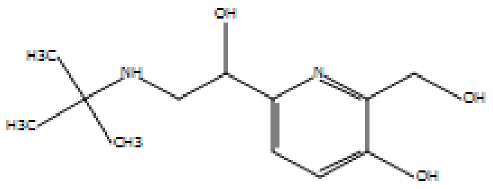

Compound Label	Name	*m/z*	RT	Algorithm	Mass
Cpd 24: 2-Ethyl-5-methylpyridine; C8 H11 N	**2-Ethyl-5-methylpyridine**	144.0794	4.479	Auto MS/MS	121.0903

MS Spectrum

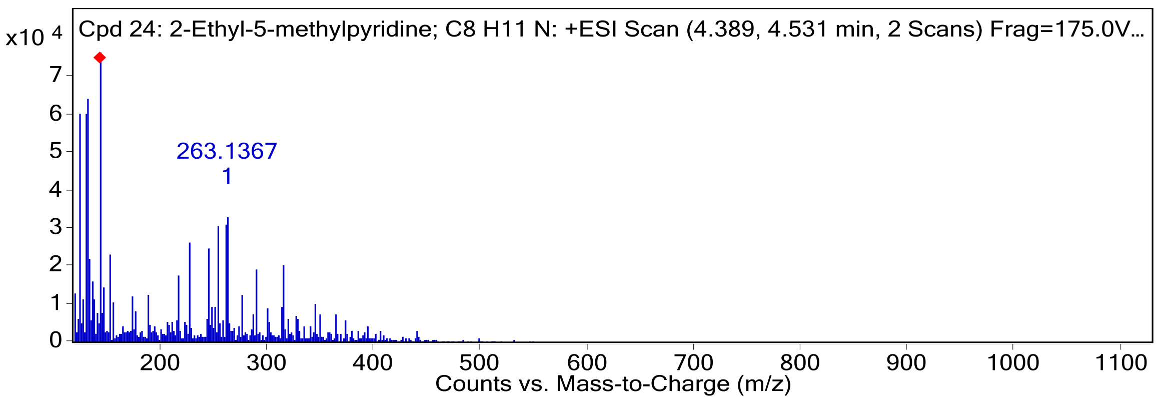

MS Zoomed Spectrum

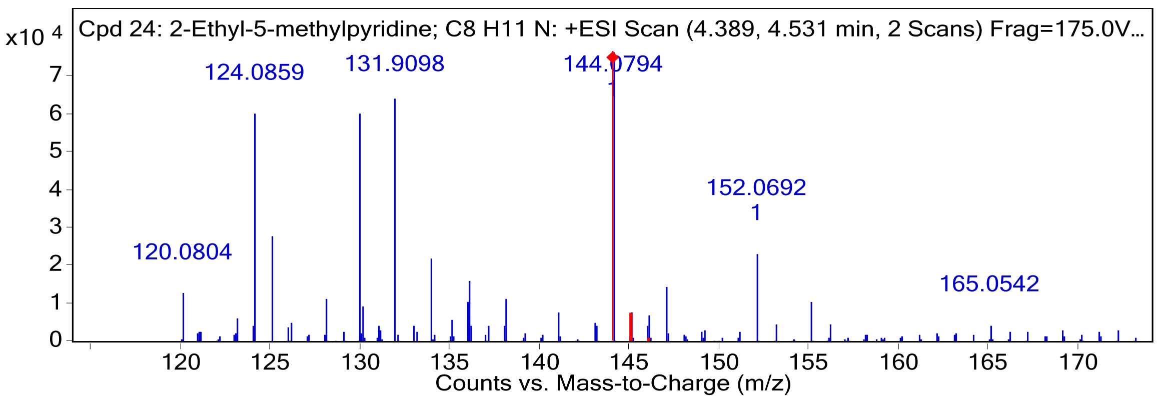

MS Spectrum Peak List

m/z	*Calc m/z*	Diff(ppm)	z	Abund	Formula	Ion
124.0859				60384.45		
125.0701				28055.39		
129.9126				60202.46		
131.9098				64291.52		
144.0794	144.0784	-7.43	1	76461.9	C8 H11 N	(M+Na)+
145.0827	145.0815	-8.46	1	7965.92	C8 H11 N	(M+Na)+
146.0906	146.0846	-40.72	1	1179.9	C8 H11 N	(M+Na)+
254.1726			1	30692.17		
261.1209			1	31199.71		
263.1367			1	32947.73		

MSMS Spectrum

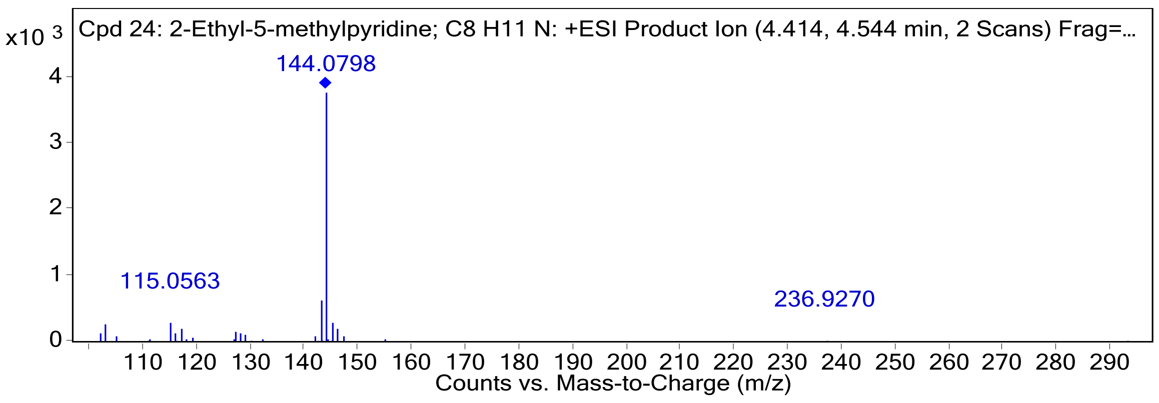

MS/MS Spectrum Peak List

m/z	z	Abund
102.088		134.46
103.0524		280.3
115.0563		292.99
117.0735		193.57
127.0517		160.51
143.0711		629.21
144.0798	1	3772.46
145.0839	1	292.13
145.1036		172.71
146.0615		192.78

Compound Structure

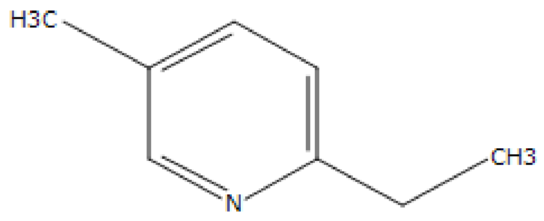

Compound Label	*m/z*	RT	Algorithm

Compound 25	254.1726	4.622	Auto MS/MS

MS Spectrum

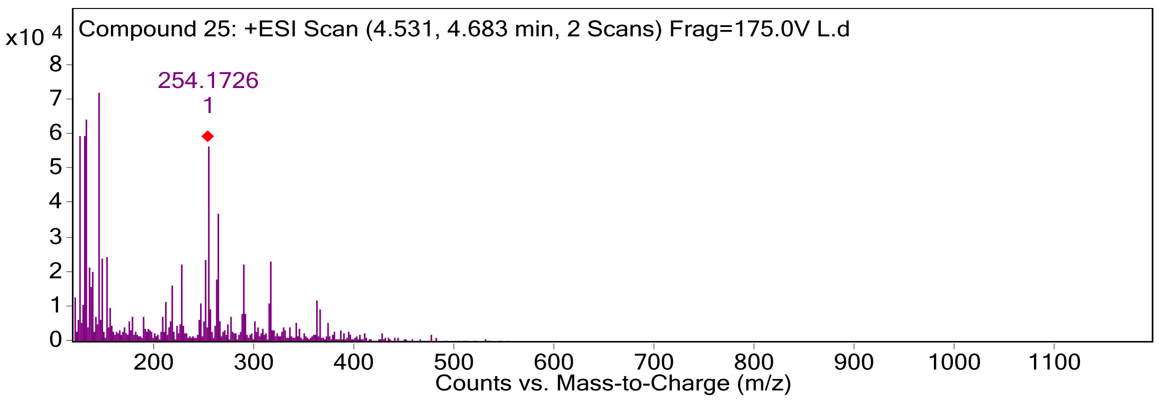

MS Zoomed Spectrum

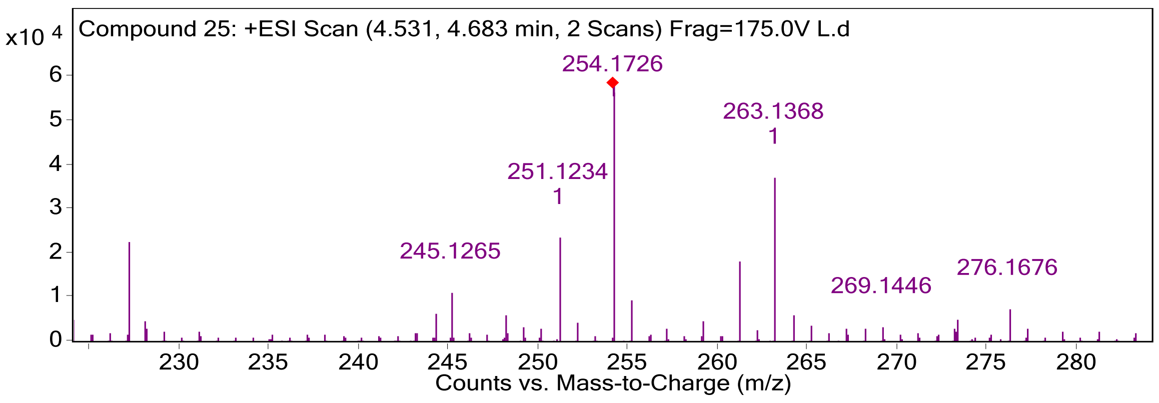

MS Spectrum Peak List

m/z	z	Abund
124.0858		59455.63
125.0704		27272.89
129.9126		59790.37
131.9099		64439.79
144.0795	1	72200.67
152.0689	1	24619.51
254.1726	1	56495.48
255.1762	1	9377.37
256.1789	1	1573.23
263.1368	1	37217.99

MSMS Spectrum

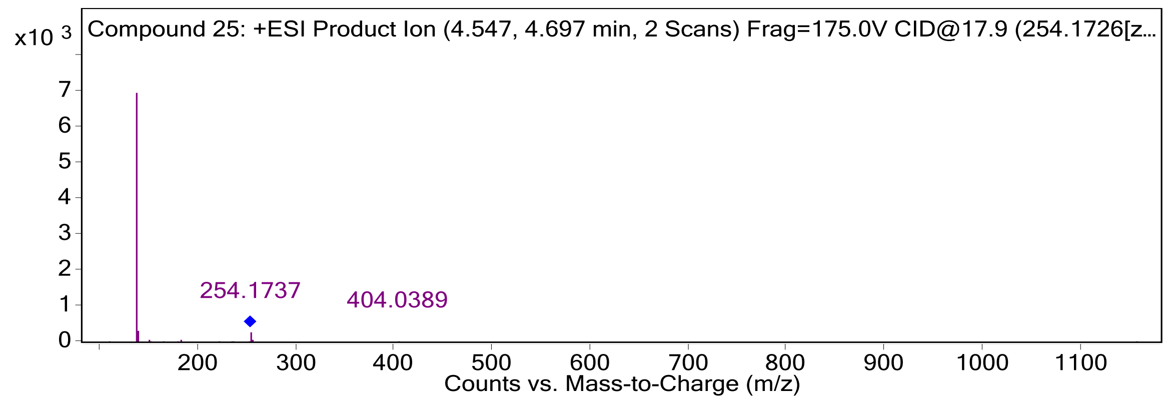

MS/MS Spectrum Peak List

m/z	z	Abund
136.9298		236.29
137.0803		60.5
137.9661		60.16
138.09	1	6982.34
139.093	1	335.29
150.0539		64.18
182.0805		68.28
253.1497		67.3
254.1737	1	288.55
255.1715	1	101.94

Compound Label	Name	*m/z*	RT	Algorithm	Mass
Cpd 26: Citrinin; C13 H14 O5	**Citrinin**	273.0726	5.017	Auto MS/MS	250.0835

MS Spectrum

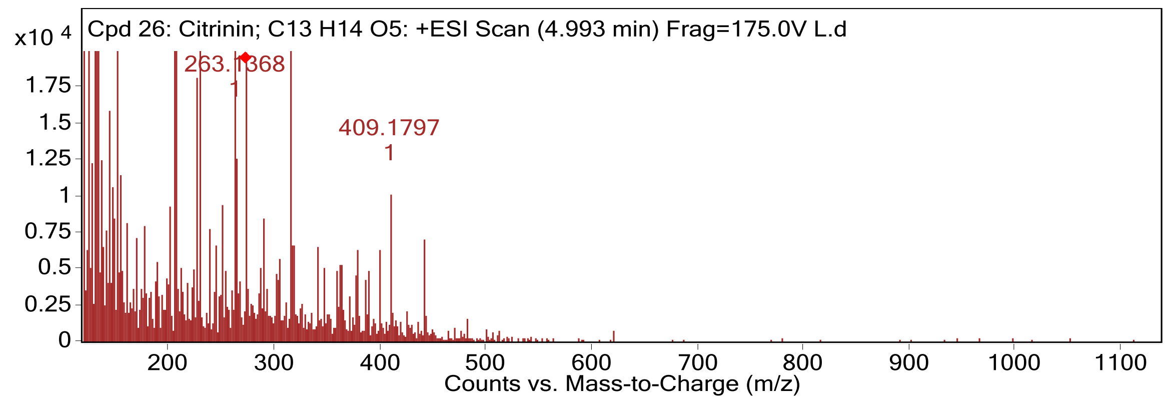

MS Zoomed Spectrum

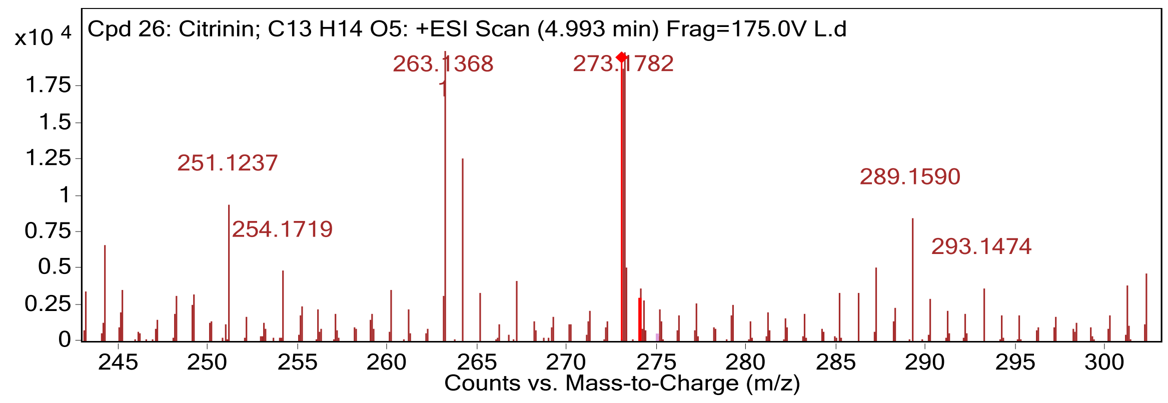

MS Spectrum Peak List

m/z	*Calc m/z*	Diff(ppm)	z	Abund	Formula	Ion
124.0859			1	69708.66		
125.0699				29158.16		
129.9126				59025.87		
131.9099				62975.67		
206.1159				44455.15		
229.0948			1	34140.89		
263.1368			1	47224.73		
273.0726	273.0733	2.88	1	18826.87	C13 H14 O5	(M+Na)+
274.0769	274.0767	-0.45	1	3687.05	C13 H14 O5	(M+Na)+
315.295			1	28308.41		

MSMS Spectrum

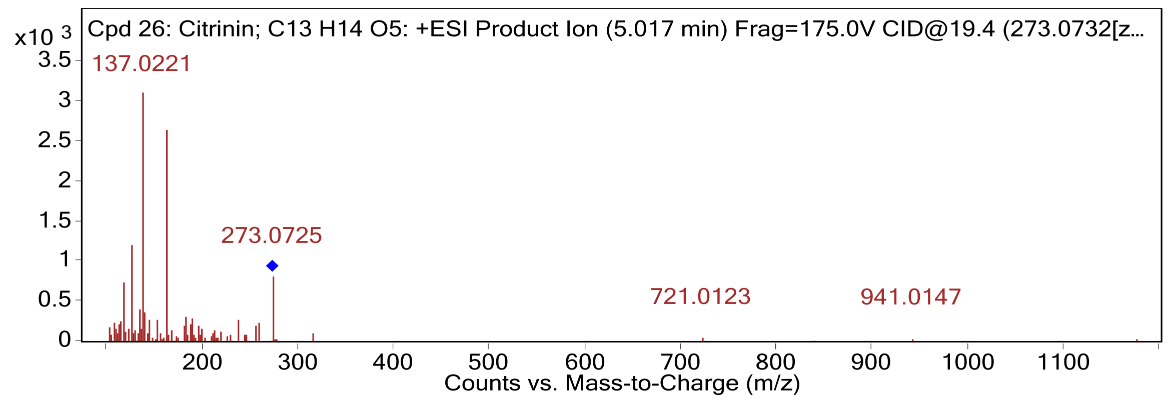

MS/MS Spectrum Peak List

m/z	z	Abund
117.0329		743.78
126.1268	1	1211.37
135.0416		408.08
137.0046		355.26
137.0221	1	3116.6
139.0348		378.67
163.0375	1	2642.57
182.0642		323.36
273.0725	1	812.35
273.1805		353.47

Compound Structure

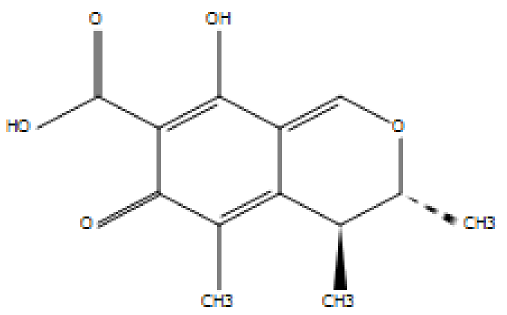

Compound Label	Name	*m/z*	RT	Algorithm	Mass
Cpd 27: [2,2-bis(2-methylpropoxy)ethyl]benzene; C16 H26 O2	**[2,2-bis(2-methylpropoxy)ethyl]benzene**	273.1781	5.126	Auto MS/MS	250.1891

MS Spectrum

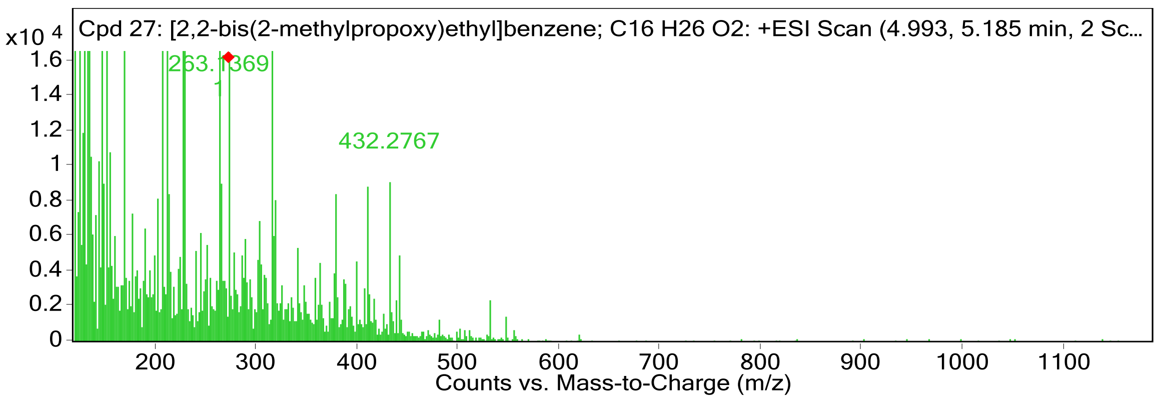

MS Zoomed Spectrum

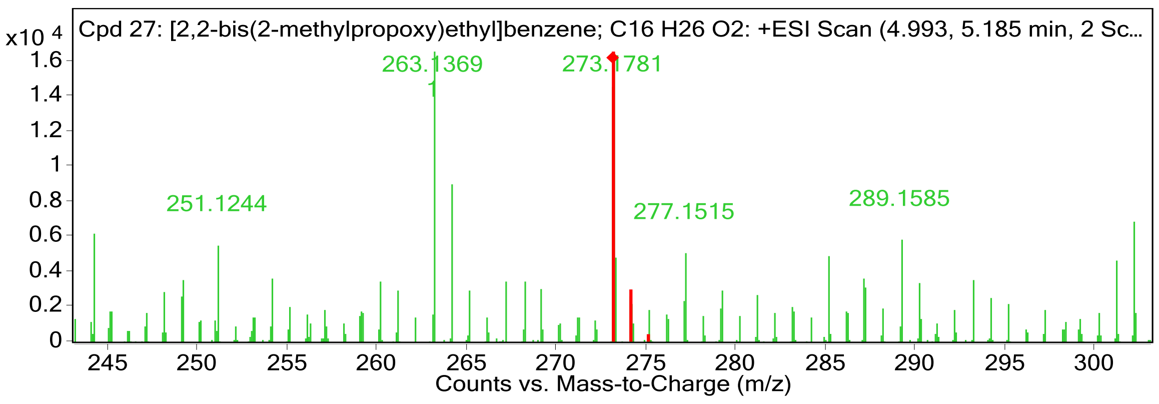

MS Spectrum Peak List

m/z	*Calc m/z*	Diff(ppm)	z	Abund	Formula	Ion
124.0859			1	67897.94		
125.0702				29542.29		
129.9126				57576.75		
131.9099				65434.28		
206.1158				30056.22		
263.1369			1	36796.36		
273.1781	273.1825	16.28		16488.1	C16 H26 O2	(M+Na)+
274.1829	274.1859	11.08	1	2249.85	C16 H26 O2	(M+Na)+
275.195	275.1888	-22.5	1	194.44	C16 H26 O2	(M+Na)+
315.2953			1	29584.11		

MSMS Spectrum

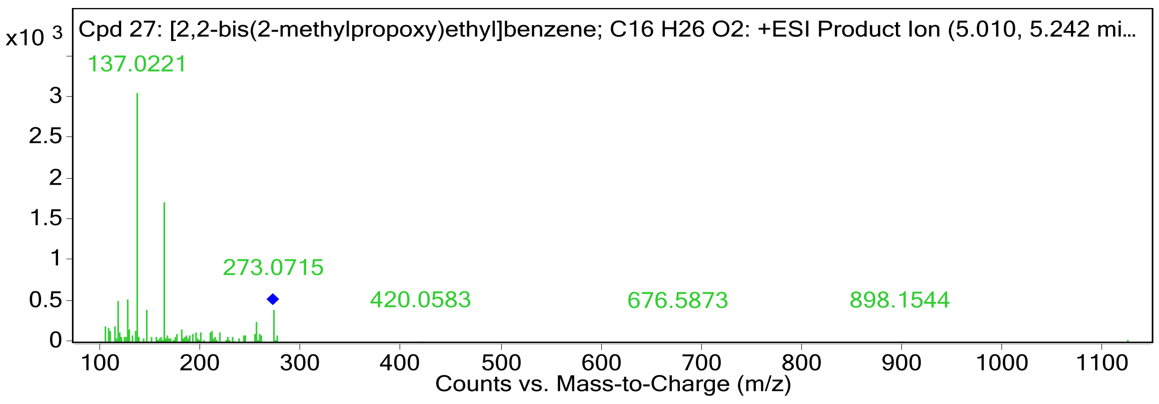

MS/MS Spectrum Peak List

m/z	z	Abund
105.0692		206.8
114.1011		192.78
117.0325		510.21
126.1283	1	525.3
137.0221	1	3061.92
145.026		394.28
163.0379	1	1712.91
255.0606		256.43
273.0715	1	410.53
273.1751		309.63

Compound Structure

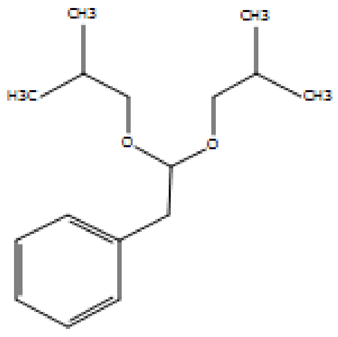

Compound Label	Name	*m/z*	RT	Algorithm	Mass
Cpd 28: Hexyl 2-furoate; C11 H16 O3	**Hexyl 2-furoate**	197.116	6.443	Auto MS/MS	196.1087

MS Spectrum

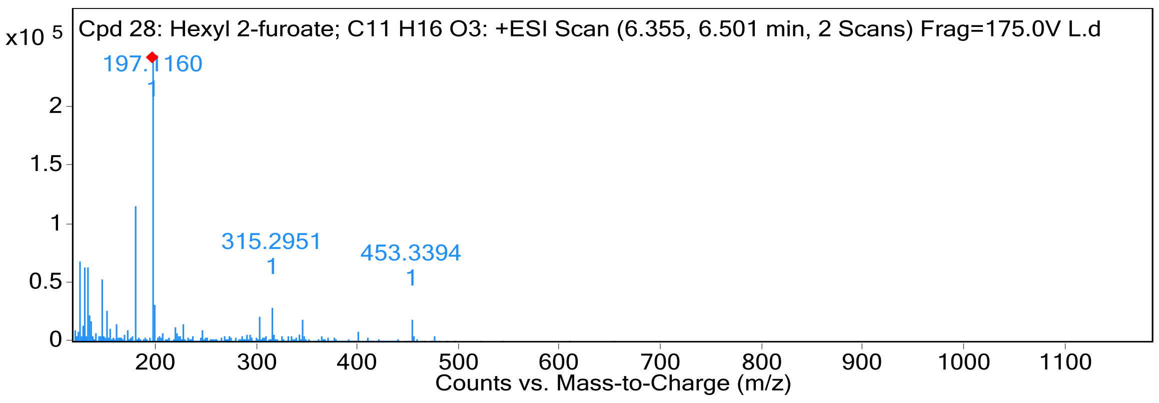

MS Zoomed Spectrum

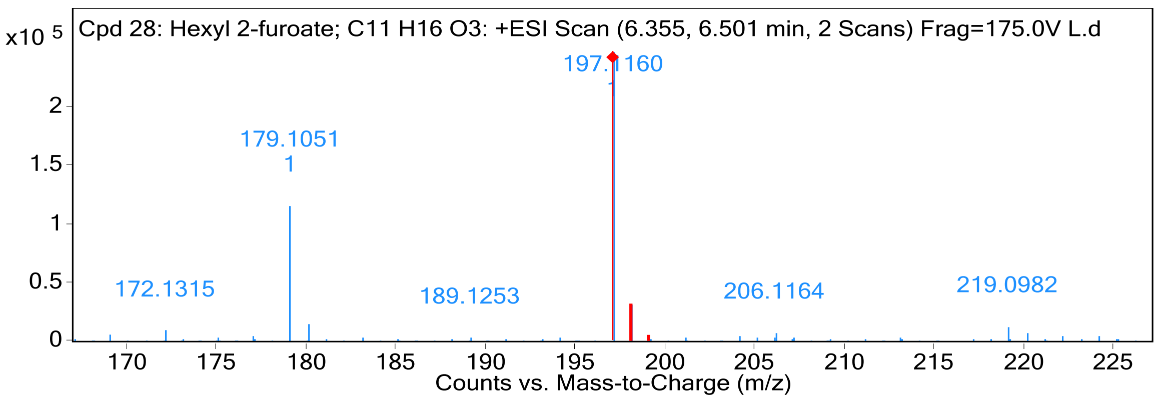

MS Spectrum Peak List

m/z	*Calc m/z*	Diff(ppm)	z	Abund	Formula	Ion
124.086				68729.53		
125.0703				32204.05		
129.9128				63843.44		
131.91				64075.36		
147.0427			1	53841.49		
179.1051			1	116593.11		
197.116	197.1172	6.04	1	247457.13	C11 H16 O3	(M+H)+
198.1189	198.1206	8.94	1	31876.16	C11 H16 O3	(M+H)+
199.1216	199.1228	6.25	1	2865.48	C11 H16 O3	(M+H)+
315.2951			1	28936.65		

MSMS Spectrum

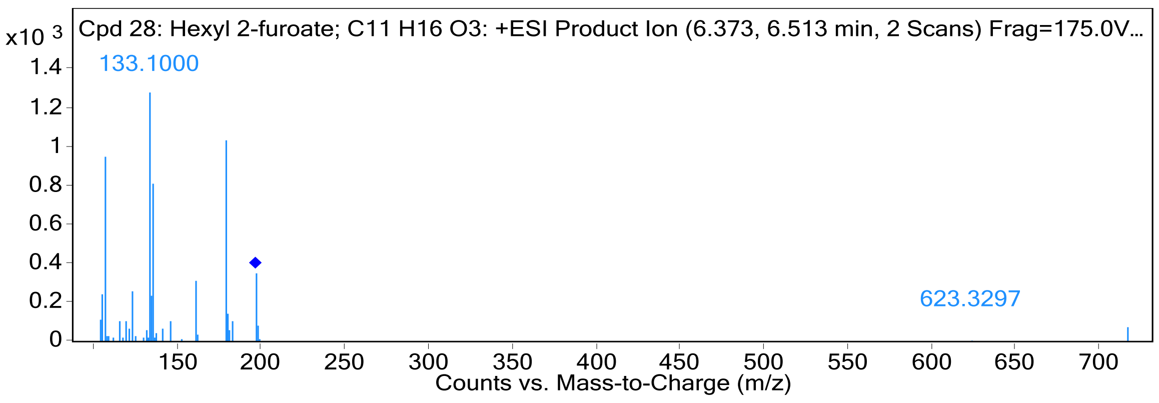

MS/MS Spectrum Peak List

m/z	z	Abund
105.0705		245.92
107.0843	2	953.5
123.1139		256.91
133.1	1	1282.2
134.101	1	234.96
135.1145	1	814.73
161.0959		316.46
179.105	1	1037.74
180.1062	1	149.13
197.1153	1	355.74

Compound Structure

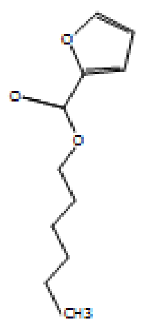

Compound Label	Name	*m/z*	RT	Algorithm	Mass
Cpd 29: 2,4,6-Triethyl-1,3,5-trioxane; C9 H18 O3	**2,4,6-Triethyl-1,3,5-trioxane**	197.1157	6.735	Auto MS/MS	174.1264

MS Spectrum

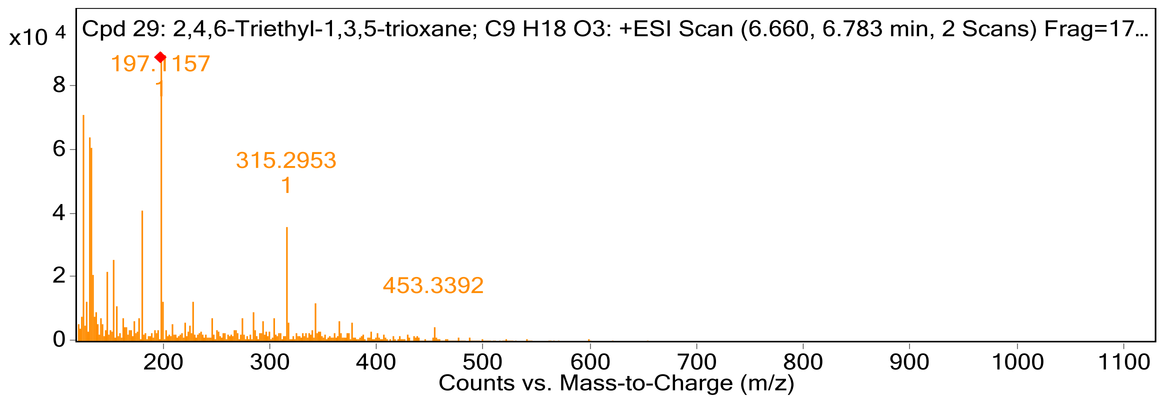

MS Zoomed Spectrum

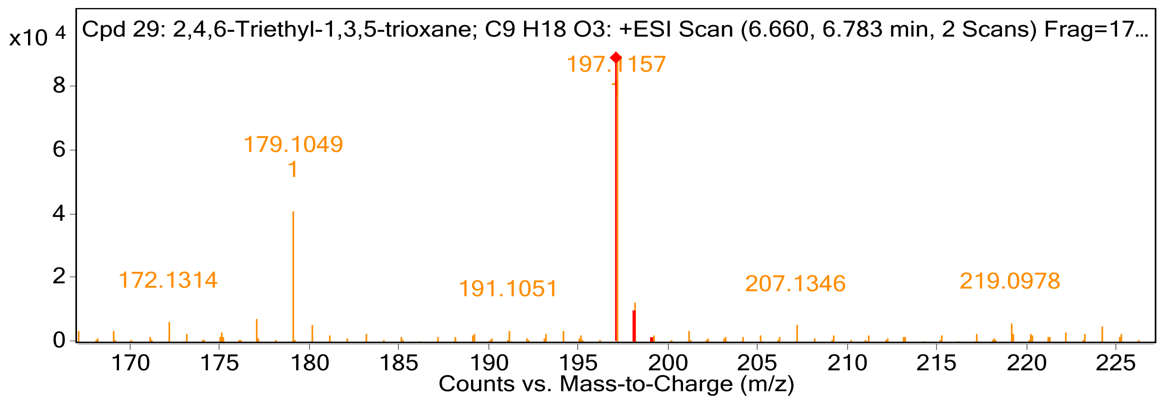

MS Spectrum Peak List

m/z	*Calc m/z*	Diff(ppm)	z	Abund	Formula	Ion
124.0861				71204.91		
125.0705			1	31251.04		
129.9127				64272.72		
131.9099				61056.38		
152.069			1	25958.91		
179.1049			1	41257.61		
197.1157	197.1148	-4.7	1	90766.2	C9 H18 O3	(M+Na)+
198.1182	198.1182	-0.02	1	12619.9	C9 H18 O3	(M+Na)+
199.1189	199.1202	6.55	1	2292.46	C9 H18 O3	(M+Na)+
315.2953			1	36061.25		

MSMS Spectrum

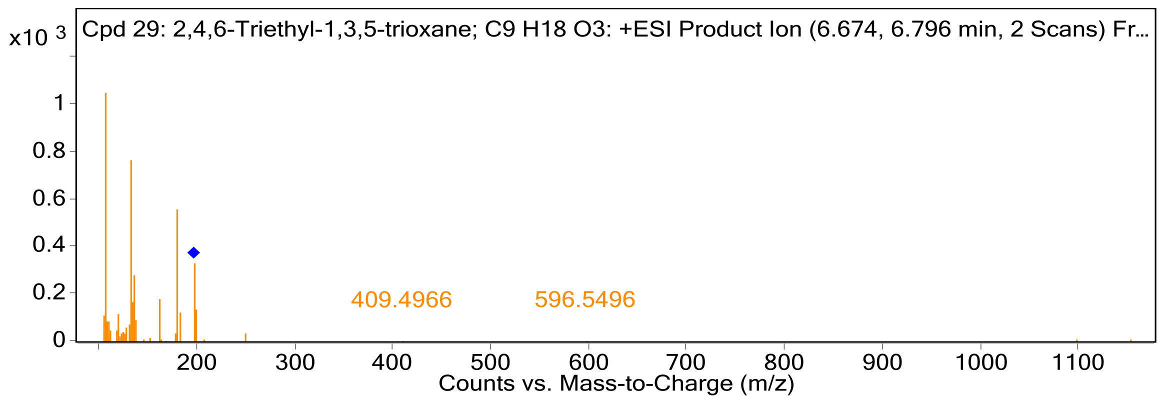

MS/MS Spectrum Peak List

m/z	z	Abund
107.0843	1	1051.47
133.099	1	764.97
134.1034	1	167.11
135.115	1	285.9
161.097	1	183.9
179.105	1	562.11
179.1213	2	152.73
182.0914		126.82
197.1137		331.69
198.1129		140.02

Compound Structure

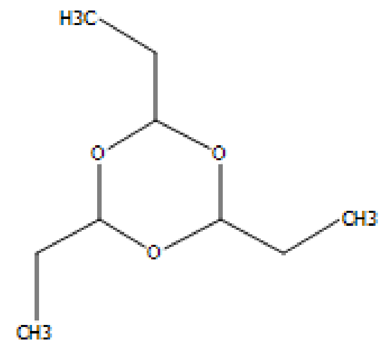

Compound Label	Name	*m/z*	RT	Algorithm	Mass
Cpd 30: Maritimetin; C15 H10 O6	**Maritimetin**	287.0528	7.888	Auto MS/MS	286.0455

MS Spectrum

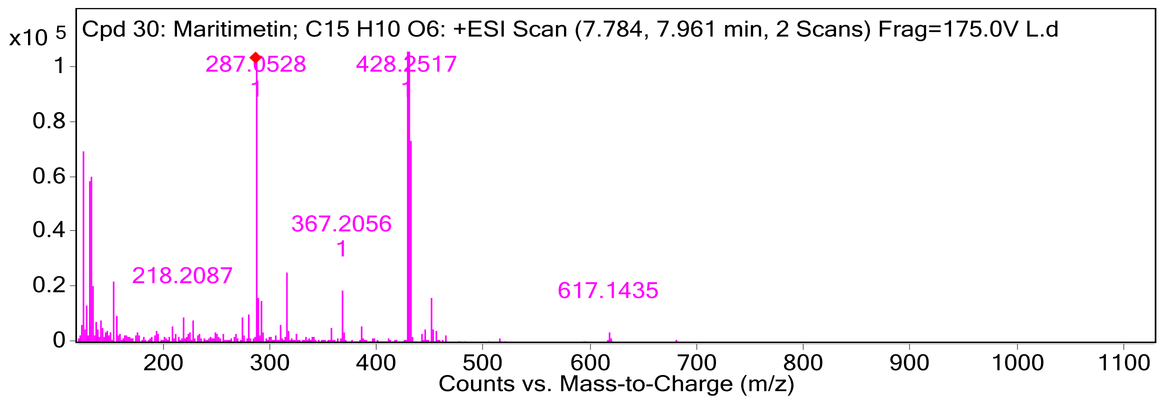

MS Zoomed Spectrum

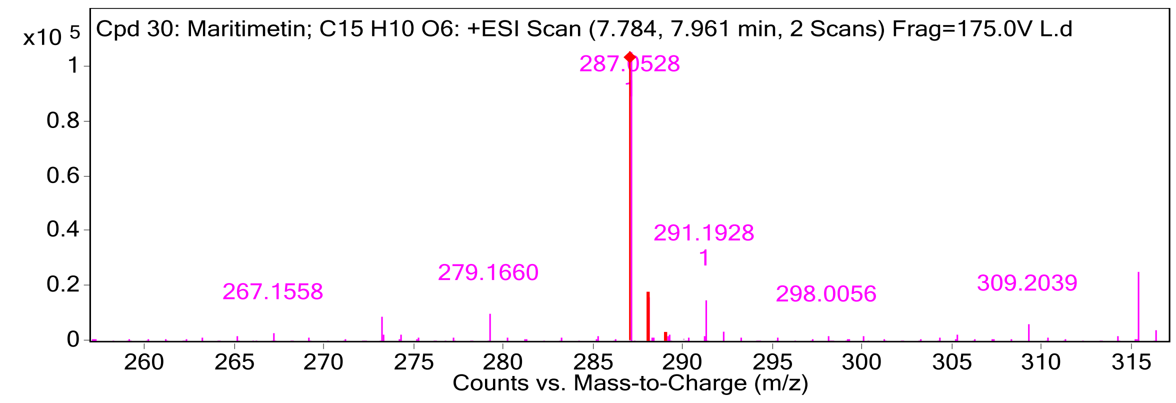

MS Spectrum Peak List

m/z	*Calc m/z*	Diff(ppm)	z	Abund	Formula	Ion
124.0861				69663.02		
125.0706				30070.57		
129.9128				58885.3		
131.91				60173.54		
287.0528	287.055	7.87	1	105392.27	C15 H10 O6	(M+H)+
288.0559	288.0584	8.86	1	16192.54	C15 H10 O6	(M+H)+
289.0595	289.0606	3.82	1	2324.72	C15 H10 O6	(M+H)+
428.2517			1	2120908.5		
429.2547			1	508964.84		
430.2574			1	73326.14		

MSMS Spectrum

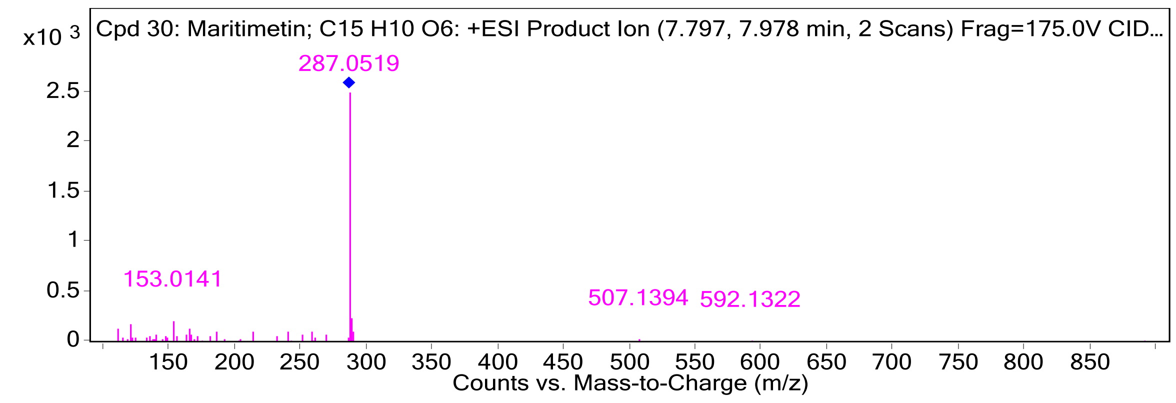

MS/MS Spectrum Peak List

m/z	z	Abund
111.0085		132.63
121.0254	1	174.07
153.0141		212.29
165.0157		136.87
186.1092		104.06
213.052		106.05
287.0195		120.9
287.0519	1	2500.49
288.0543	1	236.63
289.0604	1	111.01

Compound Structure

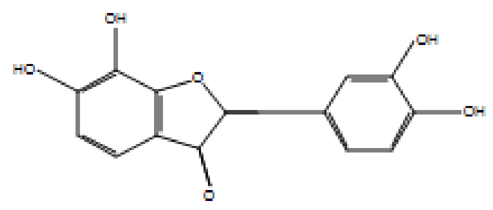

Compound Label	Name	*m/z*	RT	Algorithm	Mass
Cpd 31: Ismine; C15 H15 N O3	**Ismine**	258.1125	8.808	Auto MS/MS	257.1054

MS Spectrum

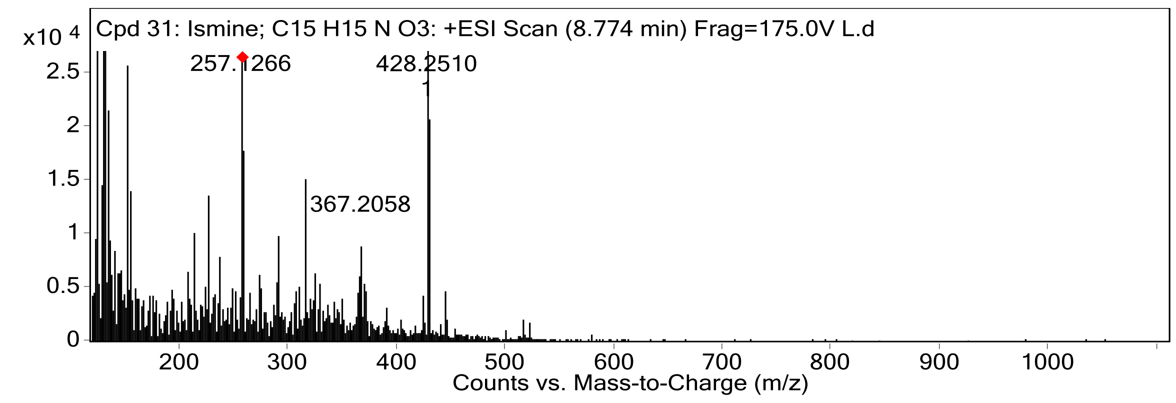

MS Zoomed Spectrum

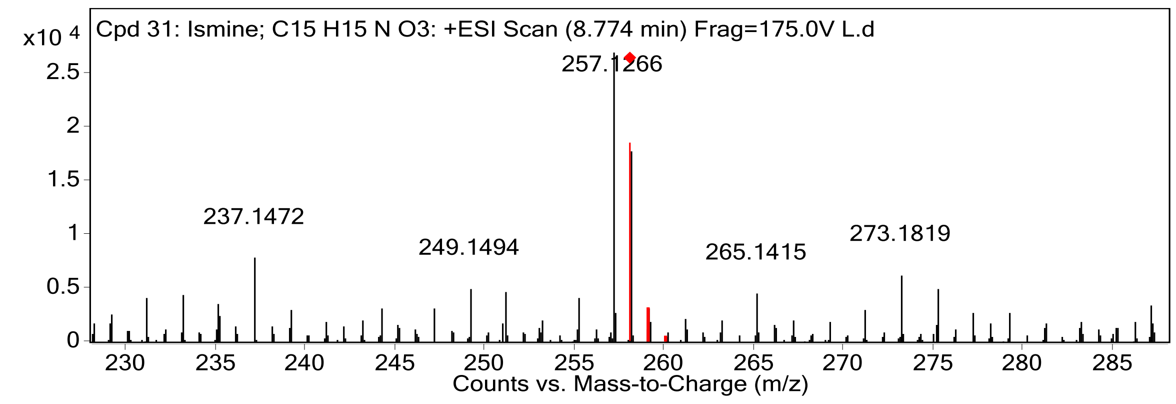

MS Spectrum Peak List

m/z	*Calc m/z*	Diff(ppm)	z	Abund	Formula	Ion
124.0861				97467.4		

125.0709				31540.09		
129.9128				49137.13		
131.9099				57402.24		
152.0692			1	25676.46		
257.1266				26885.15		
258.1125	258.1125	0.01	1	17727.81	C15 H15 N O3	(M+H)+
259.1161	259.1157	-1.44	1	3237.92	C15 H15 N O3	(M+H)+
260.1214	260.1183	-12.25	1	903.3	C15 H15 N O3	(M+H)+
428.251			1	75609.33		

MSMS Spectrum

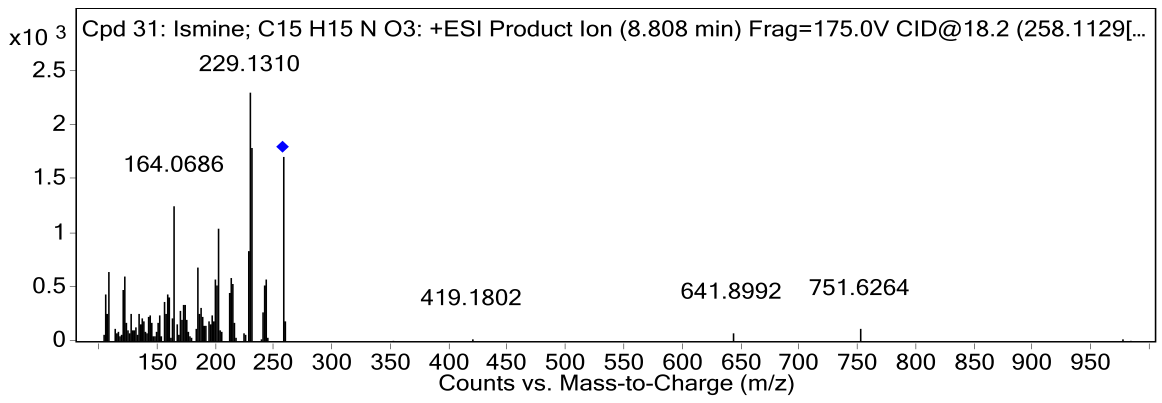

MS/MS Spectrum Peak List

m/z	z	Abund
108.0804		655.48
164.0686		1262.55
184.11	1	690.95
201.1361	1	1044.02
228.0865	1	848.45
229.131		2309.25
230.1161		1792.02
257.126		751.57
258.1097	1	1714.31
258.1288	2	898.61

Compound Structure

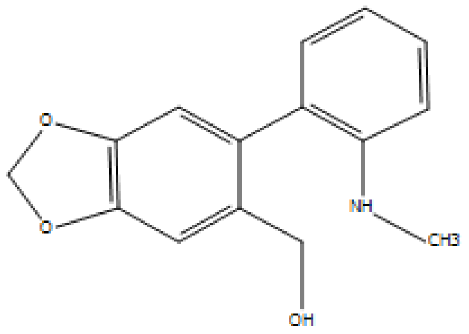

Compound Label	Name	*m/z*	RT	Algorithm	Mass
Cpd 32: Lenacil; C13 H18 N2 O2	**Lenacil**	257.1262	9.963	Auto MS/MS	234.1368

MS Spectrum

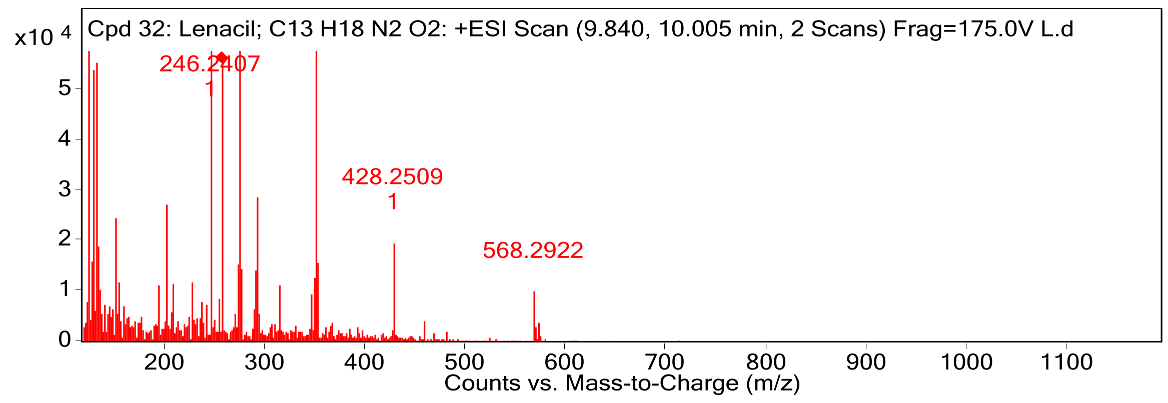

MS Zoomed Spectrum

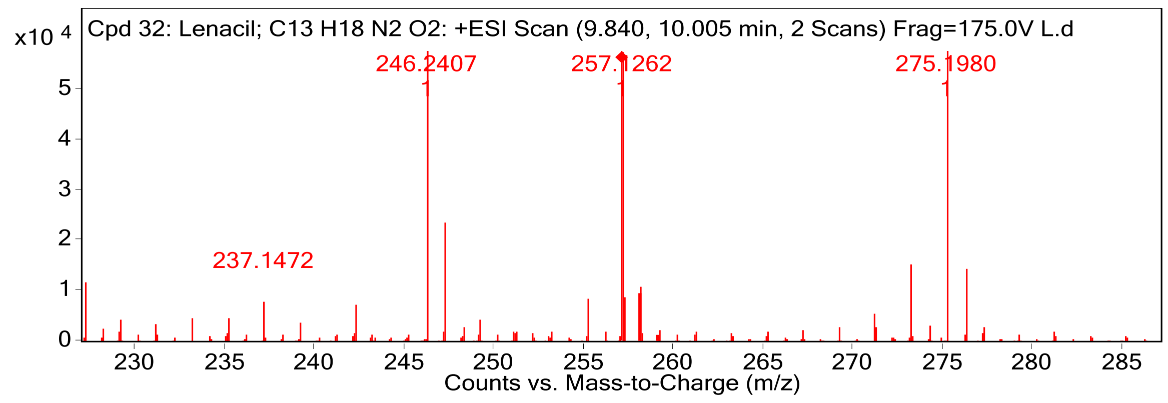

MS Spectrum Peak List

m/z	*Calc m/z*	Diff(ppm)	z	Abund	Formula	Ion
124.0861				86761.92		
125.0704				31960.71		
129.9126				53943.66		
131.9098				55407.24		
246.2407			1	170851.75		
257.1262	257.126	-0.72	1	57403.8	C13 H18 N2 O2	(M+Na)+
258.1286	258.1291	2.28	1	10822.33	C13 H18 N2 O2	(M+Na)+
259.1279	259.1317	14.57	1	2071.28	C13 H18 N2 O2	(M+Na)+
275.198			1	71349.67		
351.211			1	82434.17		

MSMS Spectrum

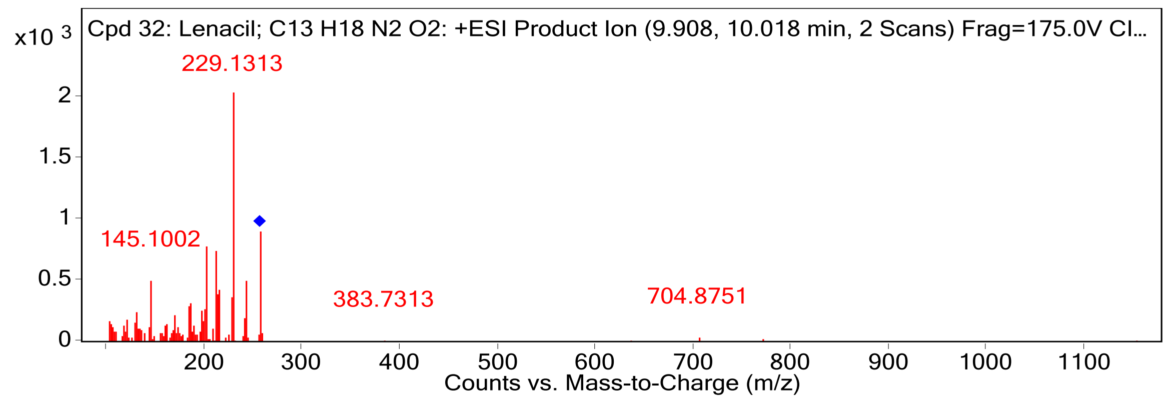

MS/MS Spectrum Peak List

m/z	z	Abund
145.1002		502.1
201.1373	1	783.7
212.105	1	741.17
213.0638		390.06
214.1152		425.27
228.0849		365.93
229.0961		797.11
229.1313	1	2043.86
242.1052	1	495.79
257.1256	1	899.38

Compound Structure

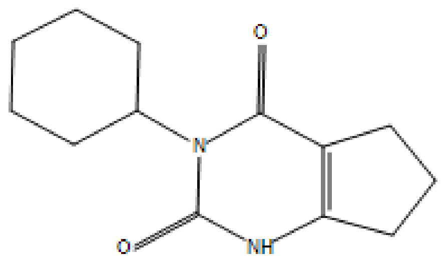

Compound Label	*m/z*	RT	Algorithm
Compound 33	246.2405	10.018	Auto MS/MS

MS Spectrum

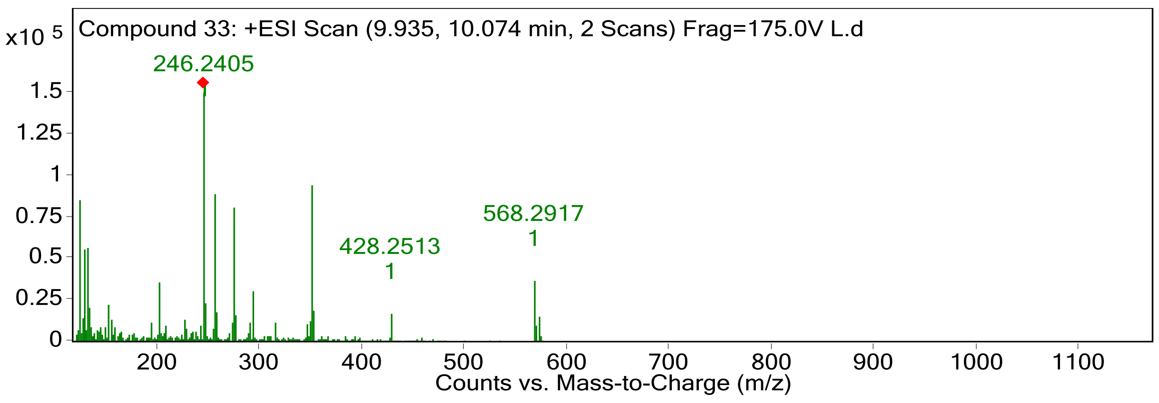

MS Zoomed Spectrum

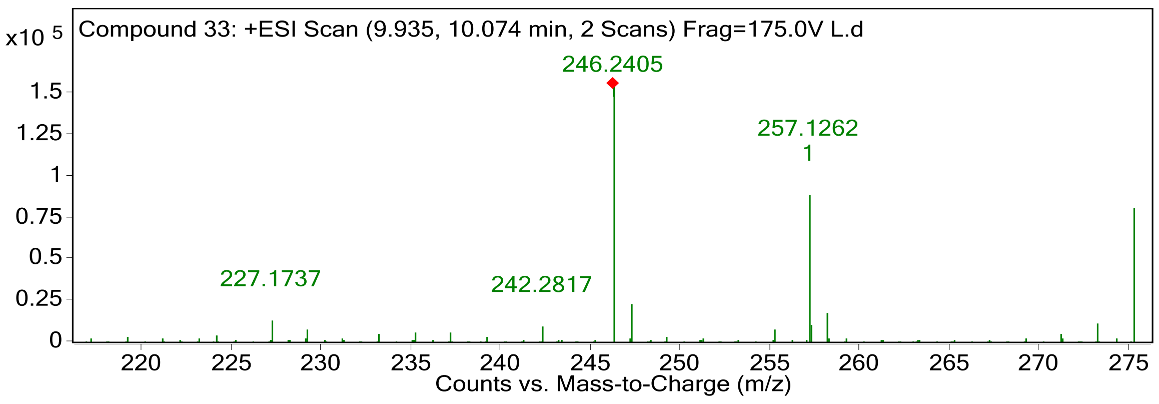

MS Spectrum Peak List

m/z	z	Abund
124.0859		85365.99
129.9127		55598.48
131.9098		56412.31
246.2405	1	150215.73
247.2441	1	23754.77
248.2466	1	2174.26
257.1262	1	89054.59
275.1983	1	80460.78
351.2113	1	94339.15
568.2917	1	37275.02

MSMS Spectrum

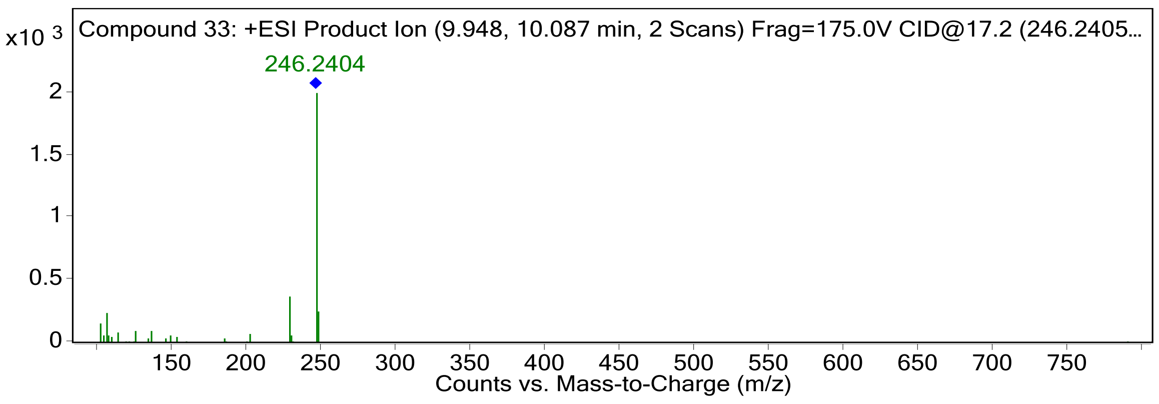

MS/MS Spectrum Peak List

m/z	z	Abund
102.0897		156.15
106.0837	1	243.41
114.0519		85.15
124.9985		101.08
136.0586		92.84
228.23	1	369.24
246.1585		145.99
246.2404	1	2002.3
247.1306		100.89
247.246	1	249.93

Compound Label	Name	*m/z*	RT	Algorithm	Mass
Cpd 34: 3-Hydroxynonyl acetate; C11 H22 O3	**3-Hydroxynonyl acetate**	225.1467	11.396	Auto MS/MS	202.1574

MS Spectrum

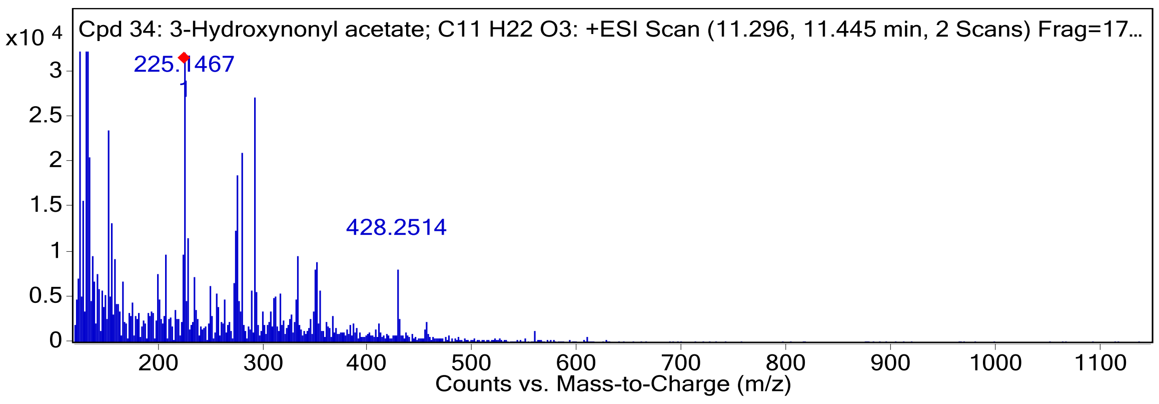

MS Zoomed Spectrum

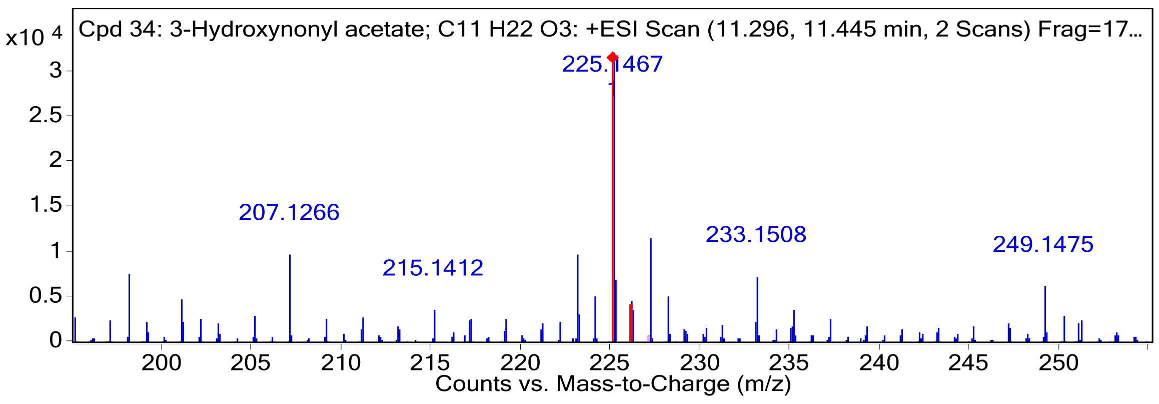

MS Spectrum Peak List

m/z	*Calc m/z*	Diff(ppm)	z	Abund	Formula	Ion

124.086				81528.69		
125.0705			1	34187.07		
129.9127				59032.69		
131.9098				64985.45		
133.9068				20609.4		
152.0693			1	23565.3		
225.1467	225.1461	-2.41	1	32124.64	C11 H22 O3	(M+Na)+
226.1499	226.1495	-1.55	1	4714.56	C11 H22 O3	(M+Na)+
279.0912			1	21014.51		
291.1927			1	27081.32		

MSMS Spectrum

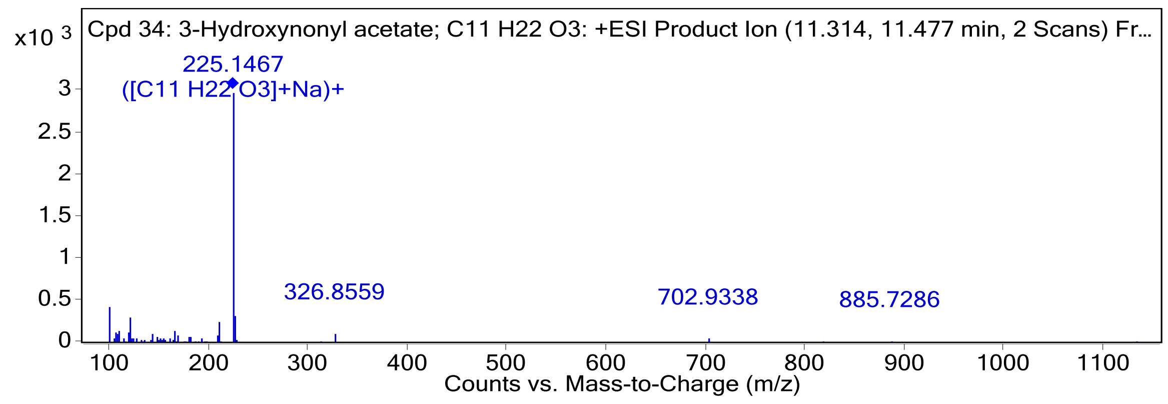

MS/MS Spectrum Peak List

m/z	*Calc m/z*	Diff (ppm)	z	Abund	Formula	Ion
100.1099				432.84		
109.0637				135.09		
121.0636			1	306.14		
165.0556				138.36		
210.1236				253.3		
224.1132				181.85		
224.1284				185.66		
225.1467	225.1461	-2.57	1	2976.41	C11 H22 O3	(M+Na)+
226.1493			1	320.23		
226.197			2	130.21		

Compound Structure

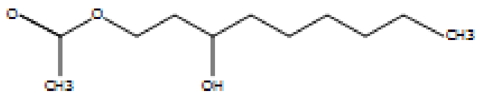

Compound Label	Name	*m/z*	RT	Algorithm	Mass
Cpd 35: C16 Sphinganine; C16 H35 N O2	**C16 Sphinganine**	274.2727	11.808	Auto MS/MS	273.2653

MS Spectrum

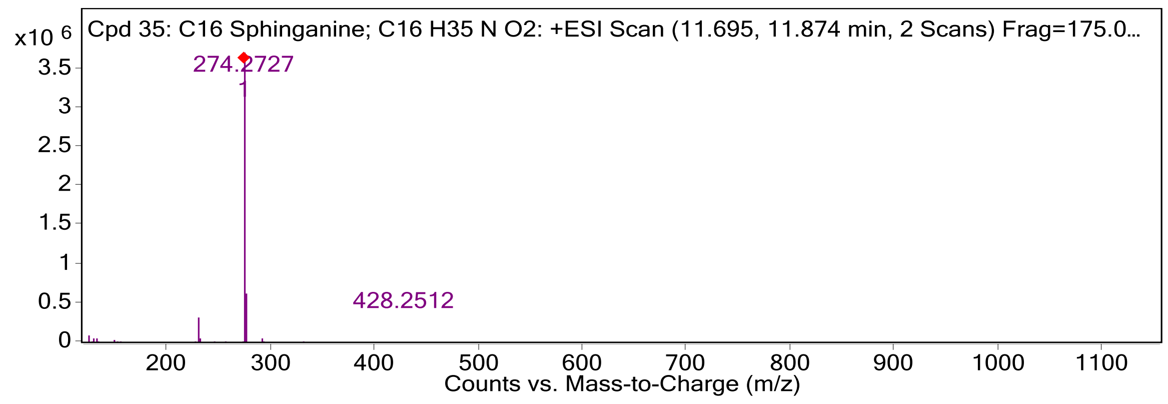

MS Zoomed Spectrum

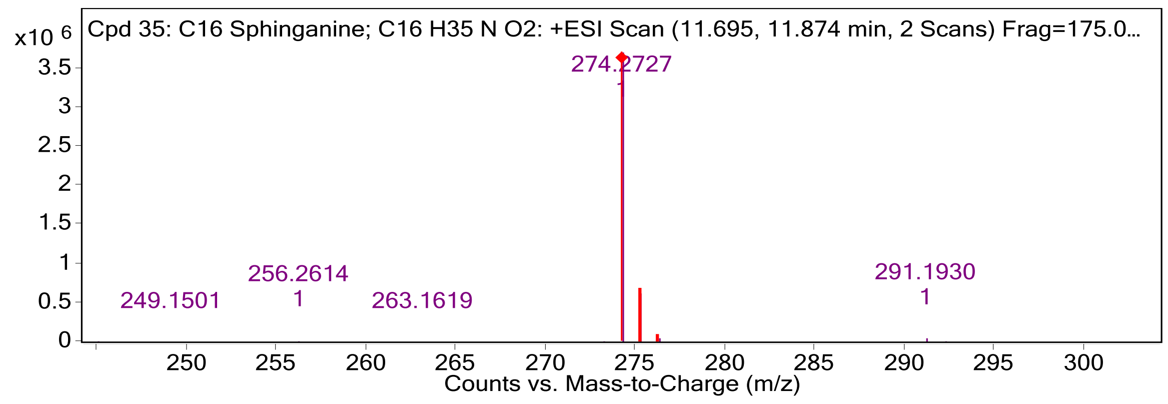

MS Spectrum Peak List

m/z	*Calc m/z*	Diff(ppm)	z	Abund	Formula	Ion
124.0861			1	90398.3		
129.9126				56363.63		
131.9099				61321.95		
149.022			1	36462.68		
230.2461			1	333540.13		
231.249			1	51578.4		
274.2727	274.2741	5.11	1	3704582	C16 H35 N O2	(M+H)+
275.2754	275.2774	7.07	1	630031.94	C16 H35 N O2	(M+H)+
276.2779	276.2802	8.02	1	60314.35	C16 H35 N O2	(M+H)+
291.193			1	51312.82		

MSMS Spectrum

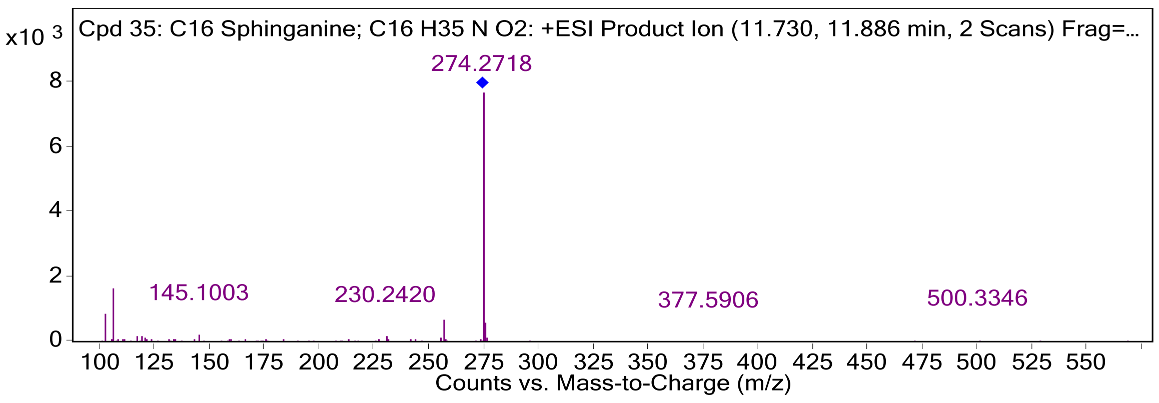

MS/MS Spectrum Peak List

m/z	z	Abund
102.0902		865.65
106.0856	1	1663.89
117.0686		188.24
119.0839		207.35
120.0883		152.49
145.1003		234.51
230.242		175.96
256.2605	1	684.07
274.2718	1	7707.91
275.2736	1	598.54

Compound Structure

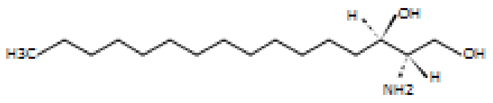

Compound Label	*m/z*	RT	Algorithm
Compound 36	272.2559	12.272	Auto MS/MS

MS Spectrum

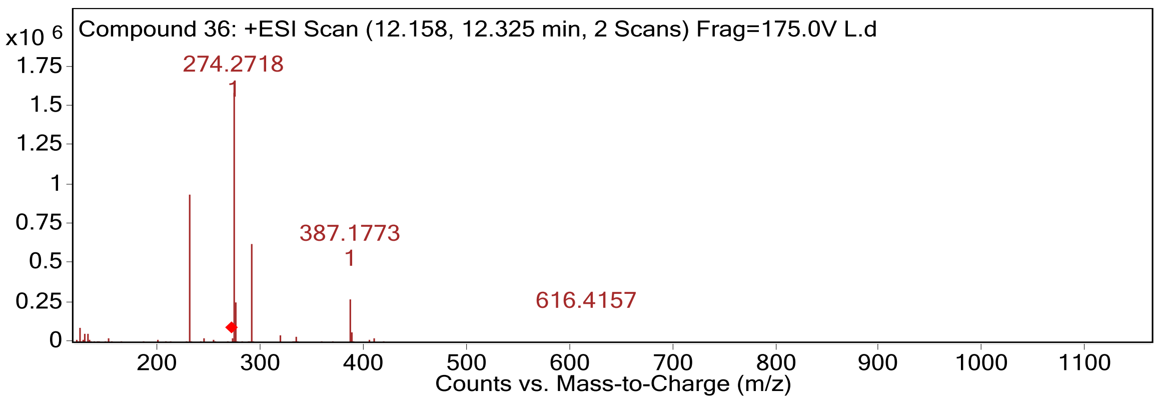

MS Zoomed Spectrum

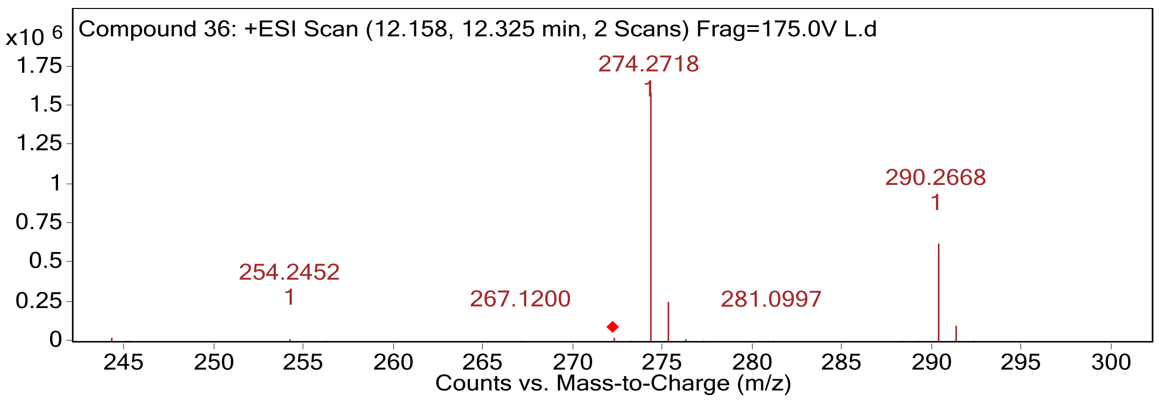

MS Spectrum Peak List

m/z	z	Abund
124.086		98404.8
230.2459	1	943300
231.2493	1	142994.8
272.2559	1	25779.09
273.2599	1	4980.48
274.2718	1	1586735.63
275.2755	1	254032.86
290.2668	1	623919.38
291.27	1	105328.98
387.1773	1	270811.38

MSMS Spectrum

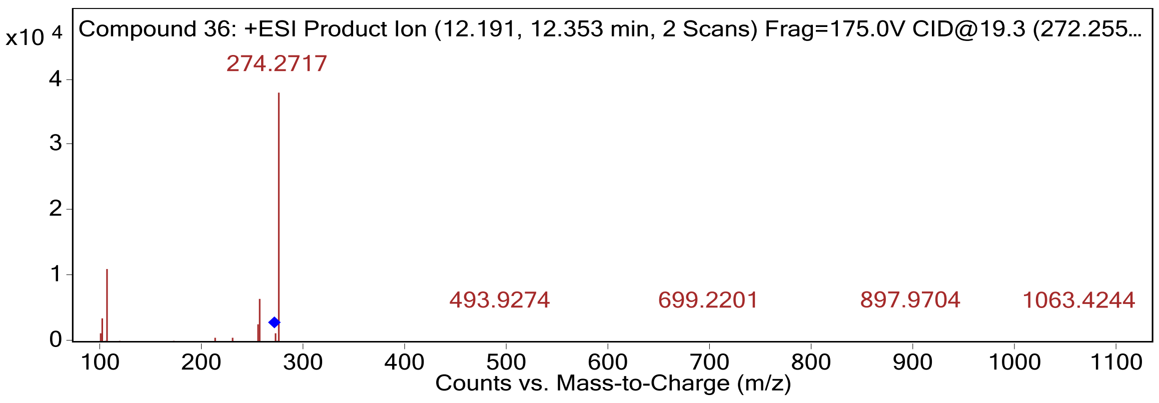

MS/MS Spectrum Peak List

m/z	z	Abund
100.0744		1284
102.0908		3607.26

106.0852		11118.94
212.2341	1	595.88
230.2452		796.11
254.1997		235.18
254.2447	1	2769.87
256.2613		6630.91
272.2552	1	1311.72
274.2717		38108.08

Compound Label	Name	*m/z*	RT	Algorithm	Mass
Cpd 37: Symlandine; C20 H31 N O6	**Symlandine**	404.2032	12.348	Auto MS/MS	381.214

MS Spectrum

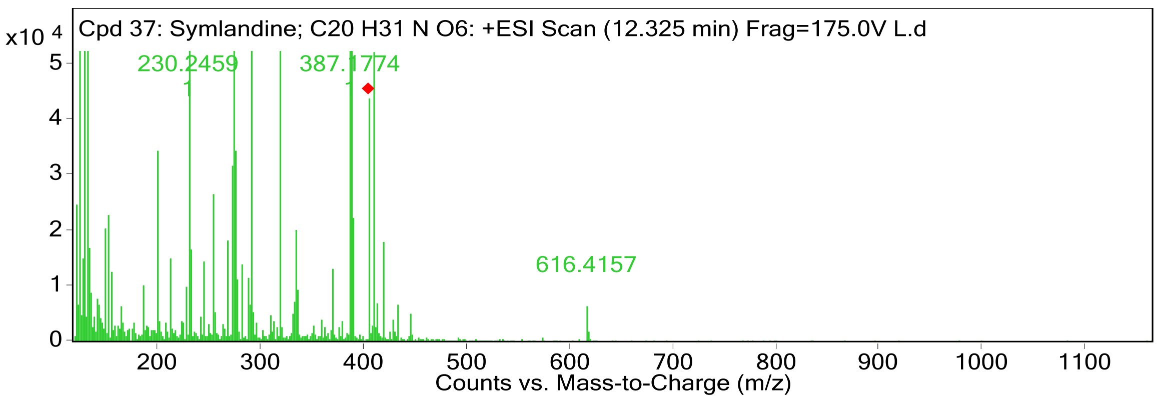

MS Zoomed Spectrum

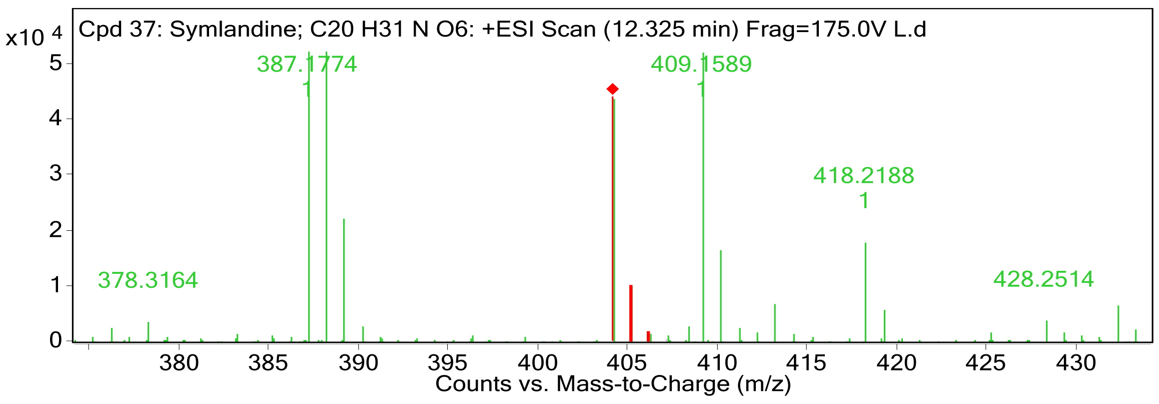

MS Spectrum Peak List

m/z	*Calc m/z*	Diff(ppm)	z	Abund	Formula	Ion
124.0861				96655.81		
230.2459			1	1137063.63		
231.2493			1	172327.92		
274.2718			1	183959.98		
290.2667			1	231026.58		
387.1774			1	535271.75		
388.1804			1	127420.34		
404.2032	404.2044	2.76	1	43956.36	C20 H31 N O6	(M+Na)+
405.2067	405.2077	2.45	1	10348.62	C20 H31 N O6	(M+Na)+
406.209	406.2102	2.78	1	1495.28	C20 H31 N O6	(M+Na)+

MSMS Spectrum

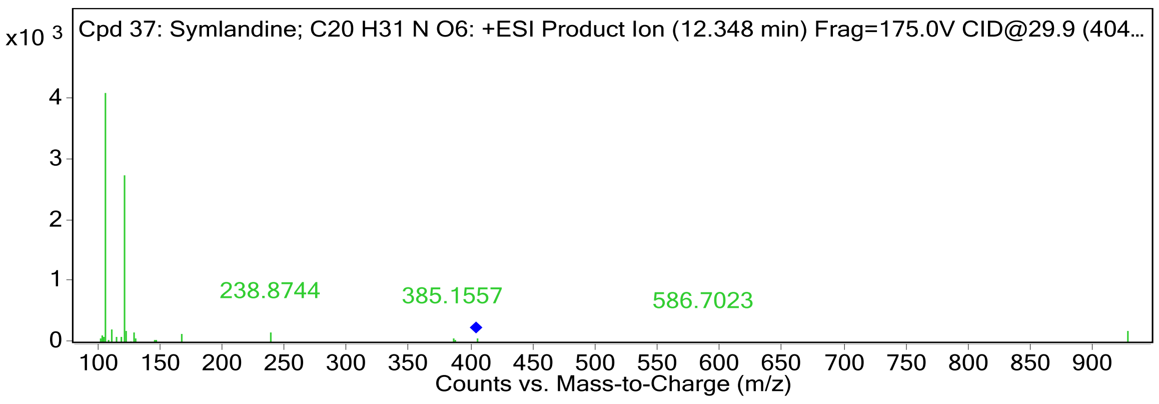

MS/MS Spectrum Peak List

m/z	z	Abund
103.05		132.72
105.0685	1	4104.82
106.0718	1	422.98
111.0423		218.53
121.0636	1	2746.4
122.0646	1	188.84
129.0533		176.98
167.0938		145.96
238.8744		170
926.2197		190.2

Compound Structure

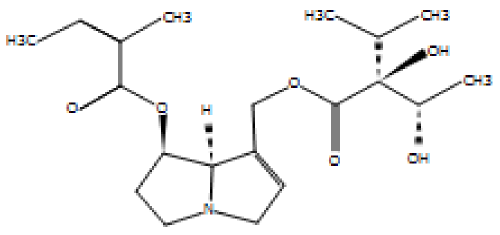

Compound Label	Name	*m/z*	RT	Algorithm	Mass
Cpd 38: Lauroyl diethanolamide; C16 H33 N O3	**Lauroyl diethanolamide**	288.2506	12.449	Auto MS/MS	287.2433

MS Spectrum

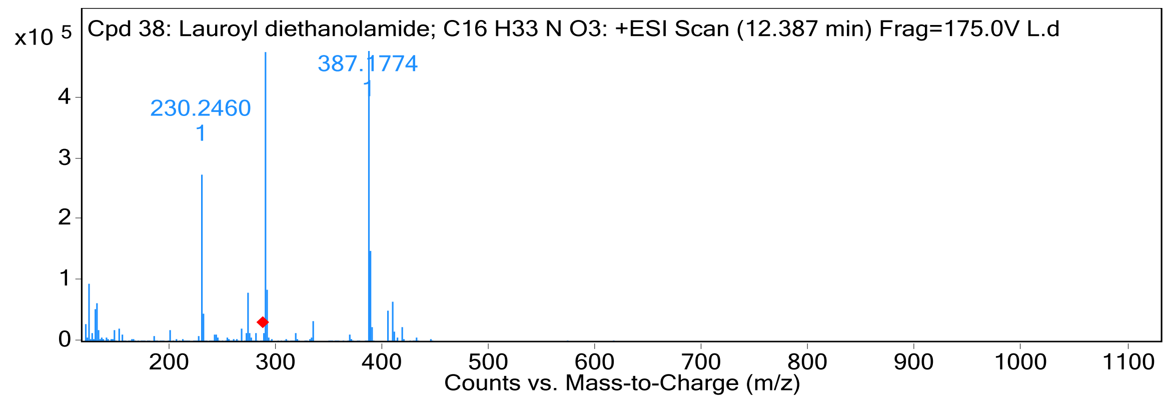

MS Zoomed Spectrum

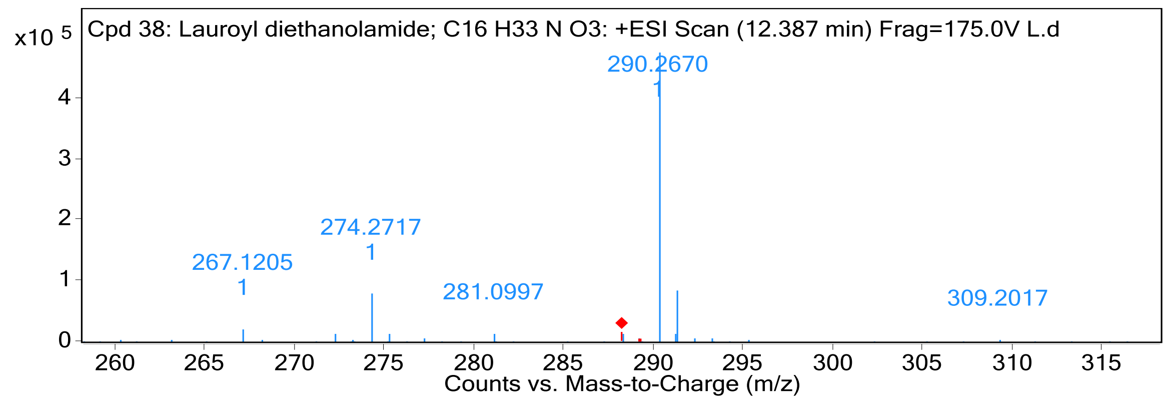

MS Spectrum Peak List

m/z	*Calc m/z*	Diff(ppm)	z	Abund	Formula	Ion
124.0861				95002.5		
230.246			1	274481.88		
274.2717			1	79895.82		
288.2506	288.2533	9.43		13553.91	C16 H33 N O3	(M+H)+
289.2537	289.2566	10.1	1	2067.76	C16 H33 N O3	(M+H)+
290.267			1	475166.34		
291.2697			1	85070.32		
387.1774			1	627109.56		
388.1806			1	148681.67		
409.1587			1	65677		

MSMS Spectrum

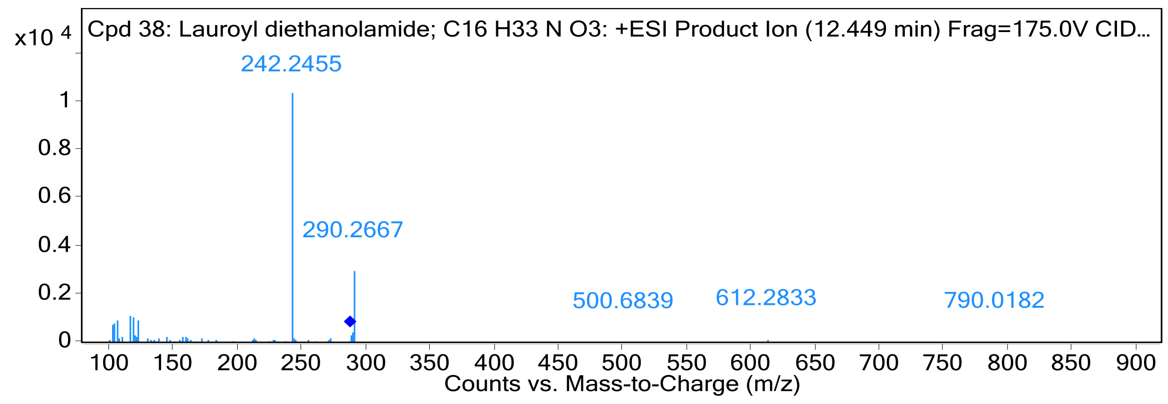

MS/MS Spectrum Peak List

m/z	z	Abund
102.0519		752.45

m/z	z	Abund
104.0694		831.12
106.0846	1	930.71
116.0711	1	1096.09
118.0862		1035.41
119.0831		340.94
122.0798		960.65
242.2455		10390.47
289.1791	1	452.71
290.2667		2965.13

Compound Structure

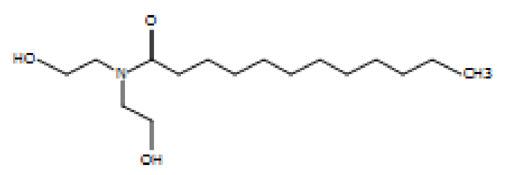

Compound Label	*m/z*	RT	Algorithm
Compound 39	290.2666	12.457	Auto MS/MS

MS Spectrum

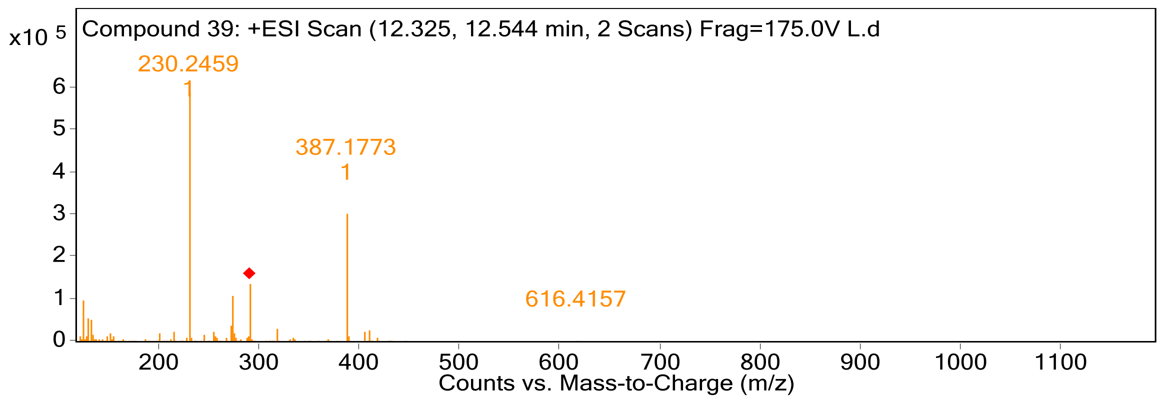

MS Zoomed Spectrum

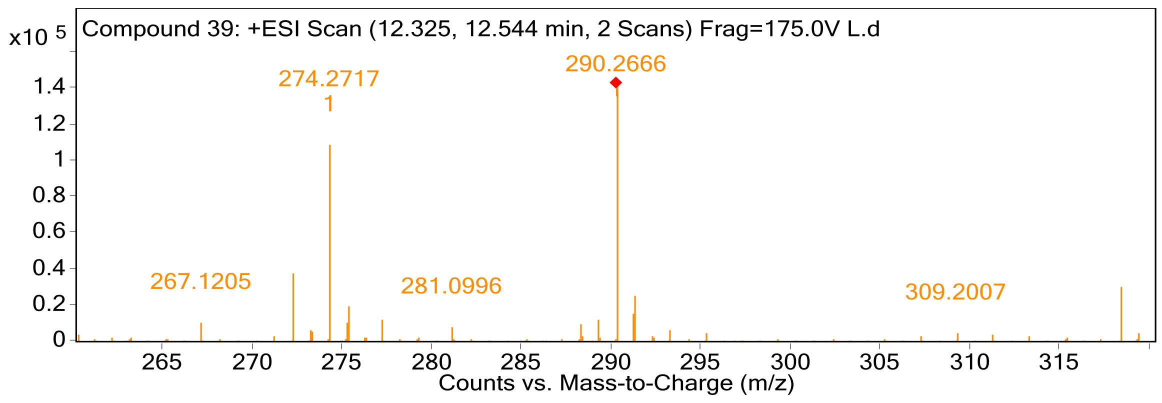

MS Spectrum Peak List

m/z	z	Abund
124.086		97561.41
129.9126		56128.28
230.2459	1	590596.25
231.2493	1	89885.59
274.2717	1	108961.43
290.2666	1	138047.14
291.2697	1	25804.08
292.2731	1	2501.33
387.1773	1	303931.09
388.1803	1	71714.76

MSMS Spectrum

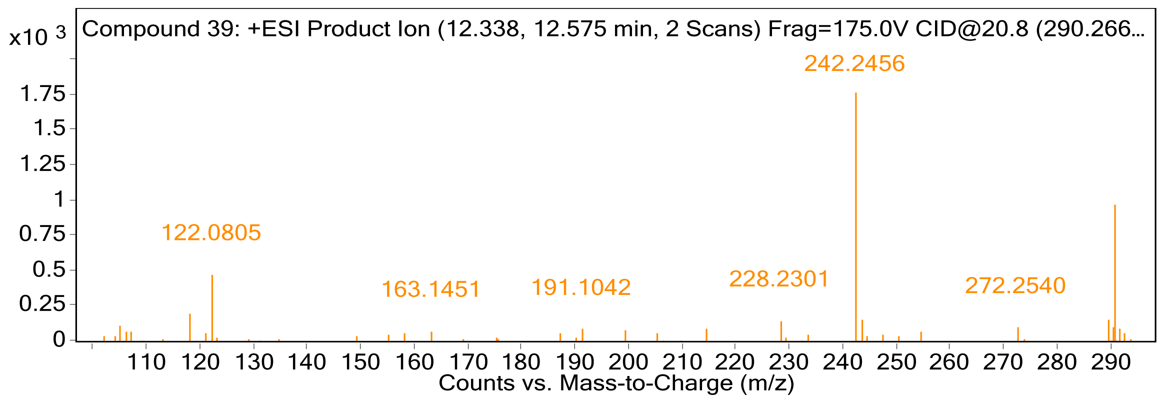

MS/MS Spectrum Peak List

m/z	z	Abund
105.0681		118.42
118.0821		196.78
122.0805	1	480.75
228.2301		151.65
242.2108		133.44
242.2456	1	1771.24
243.2475	1	159.46
289.125		107.27
289.1772		154.44
290.2662	1	978.65

Compound Label	Name	*m/z*	RT	Algorithm	Mass
Cpd 40: Gibberellin A74; C20 H28 O6	**Gibberellin A74**	387.1767	12.637	Auto MS/MS	364.1875

MS Spectrum

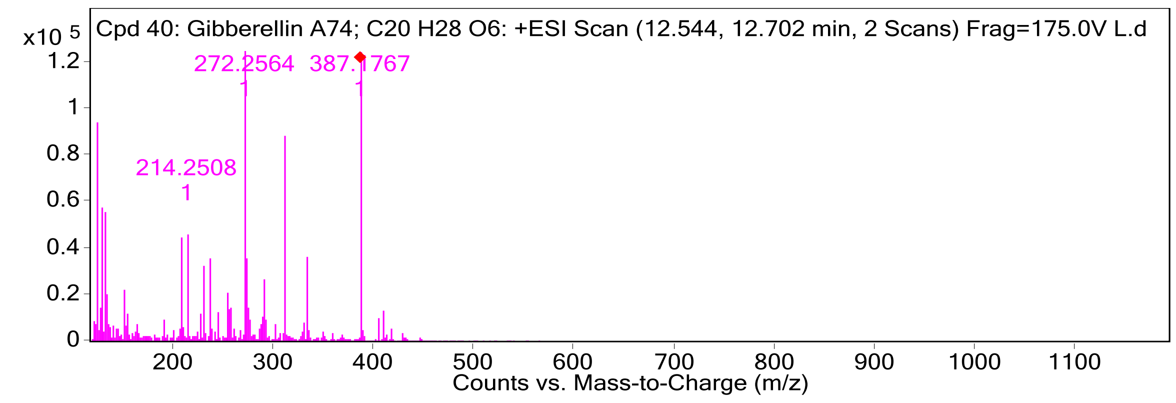

MS Zoomed Spectrum

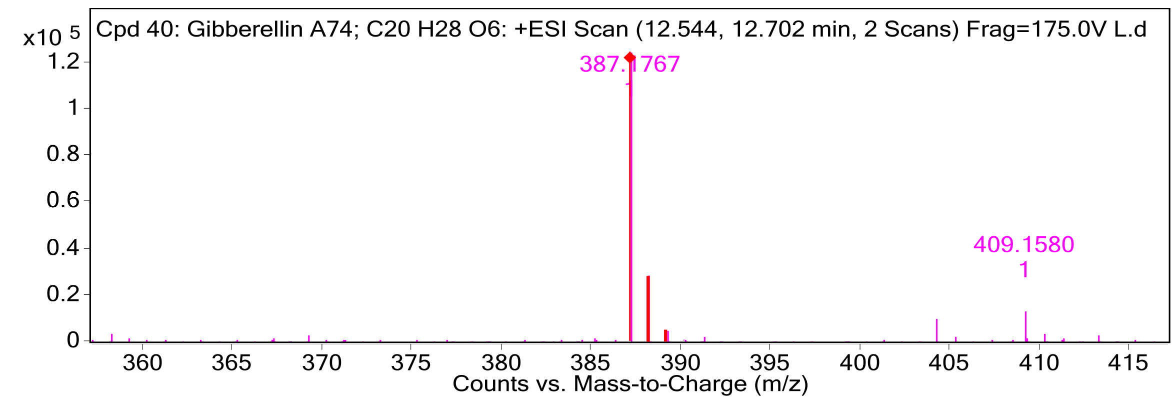

MS Spectrum Peak List

m/z	*Calc m/z*	Diff(ppm)	z	Abund	Formula	Ion
124.0859			1	94077.09		
129.9125				57479.05		
131.9098				55720.39		
209.1155			1	45113.66		
214.2508			1	46246.48		
272.2564			1	234602.39		
311.1832			1	88226.9		
387.1767	387.1778	2.85	1	124405.39	C20 H28 O6	(M+Na)+
388.18	388.1812	3.1	1	28652.91	C20 H28 O6	(M+Na)+
389.1834	389.1837	0.92	1	5002.35	C20 H28 O6	(M+Na)+

MSMS Spectrum

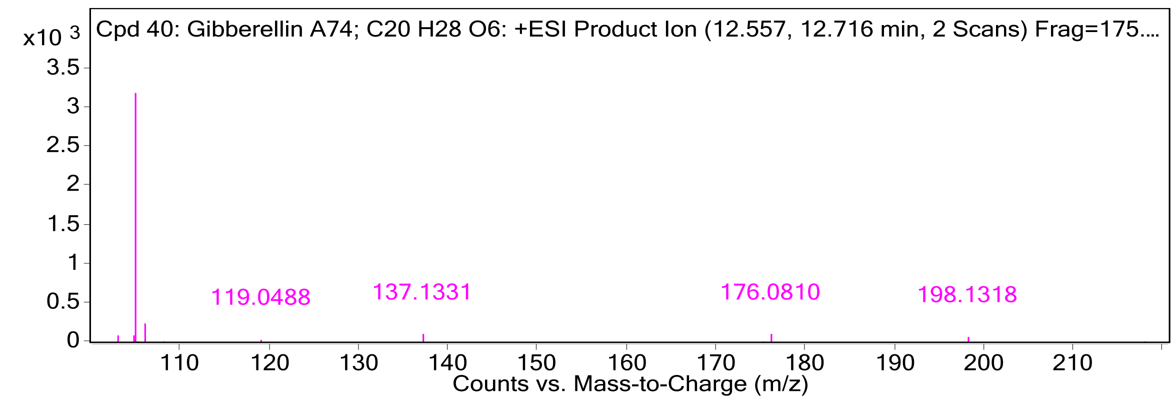

MS/MS Spectrum Peak List

m/z	z	Abund
103.0525		95.83
104.8575		94.31
104.9439		67.08
105.0071		65.58
105.0691	1	3197.82
106.0723	1	247.71
119.0488		46.51
137.1331		113.94
176.081		113.61
198.1318		80.2

Compound Structure

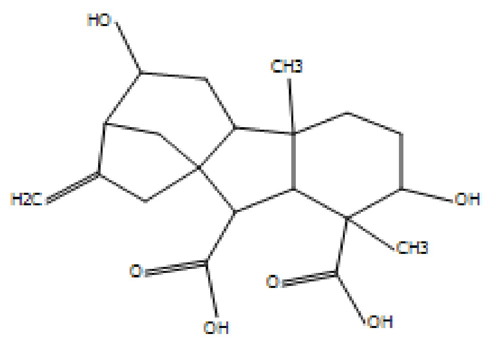

Compound Label	Name	*m/z*	RT	Algorithm	Mass
Cpd 41: Thiamylal; C12 H18 N2 O2 S	**Thiamylal**	255.1203	12.854	Auto MS/MS	254.1121

MS Spectrum

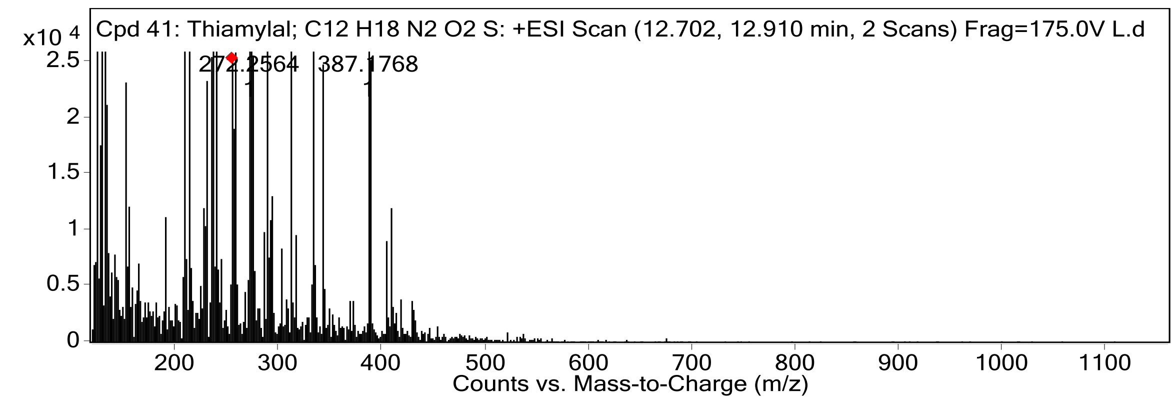

MS Zoomed Spectrum

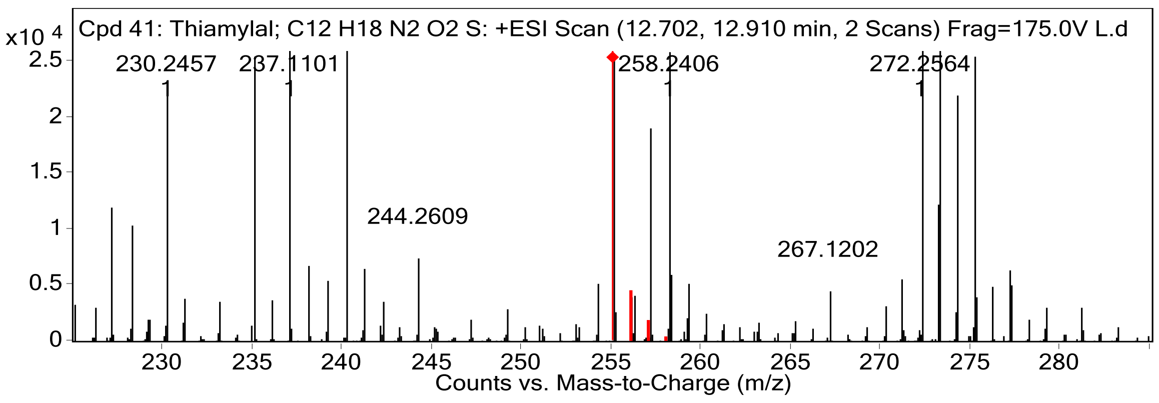

MS Spectrum Peak List

m/z	*Calc m/z*	Diff(ppm)	z	Abund	Formula	Ion
124.086			1	90224.06		
255.1203	255.1162	-16.02	1	25480.84	C12 H18 N2 O2 S	(M+H)+
256.1237	256.1191	-18.11	1	3943.12	C12 H18 N2 O2 S	(M+H)+
257.1128	257.1143	5.57	1	4884.84	C12 H18 N2 O2 S	(M+H)+
258.1154	258.1163	3.61	1	1204.23	C12 H18 N2 O2 S	(M+H)+
259.1204	259.1173	-12.1	1	316.76	C12 H18 N2 O2 S	(M+H)+
272.2564			1	242884.45		
288.2513			1	114834.34		
311.183			1	104480.17		
387.1768			1	105999.15		

MSMS Spectrum

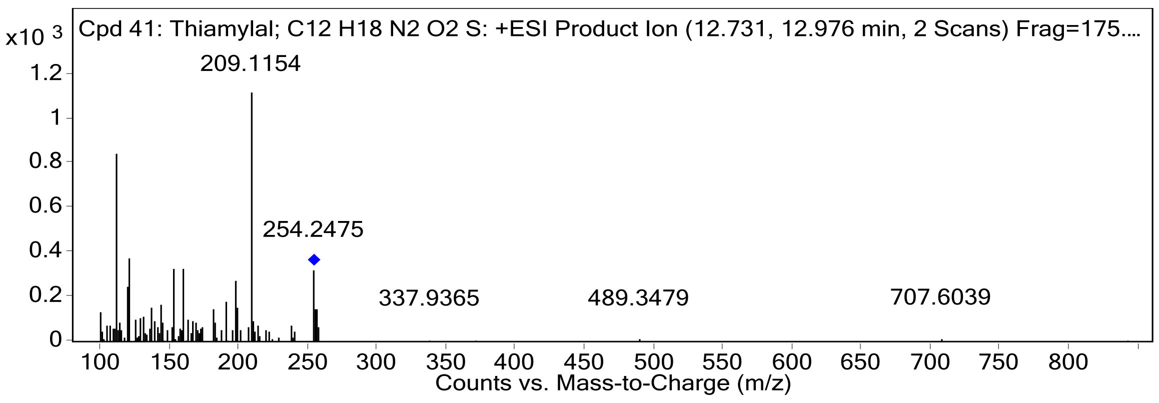

MS/MS Spectrum Peak List

m/z	z	Abund
111.0417		843.17
119.0826		246.23
121.0989		373.3
143.082		166.98
153.0529	1	330.94
159.1145		329.75
191.1041		180.29
197.129		271.5
209.1154	1	1119.13
254.2475	1	318.41

Compound Structure

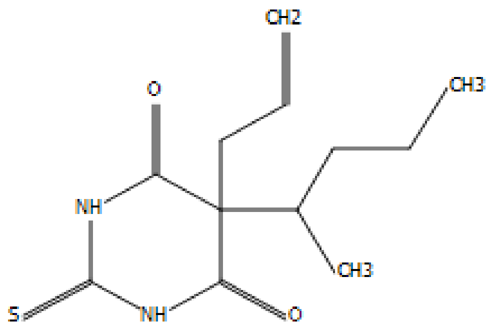

Compound Label	Name	*m/z*	RT	Algorithm	Mass
Cpd 42: 2,6-Di-tert-butyl-4-ethylphenol; C16 H26 O	**2,6-Di-tert-butyl-4-ethylphenol**	257.1877	12.876	Auto MS/MS	234.1986

MS Spectrum

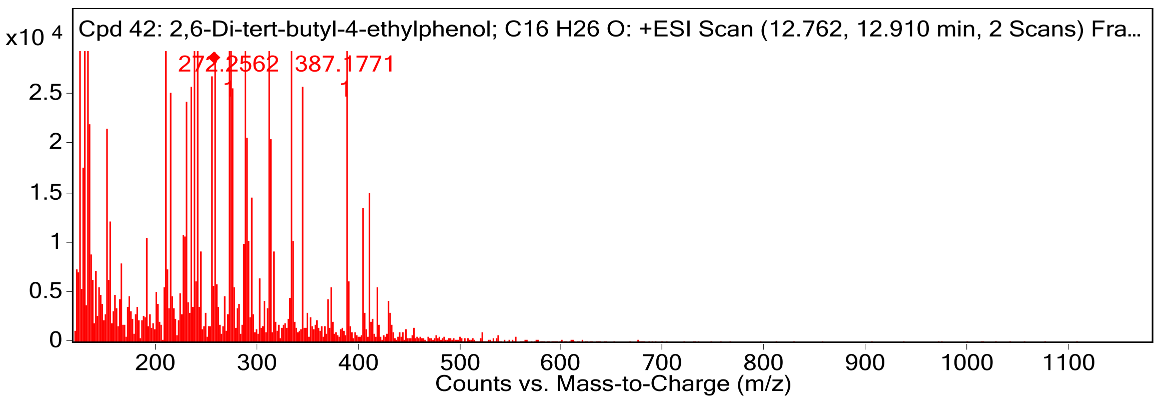

MS Zoomed Spectrum

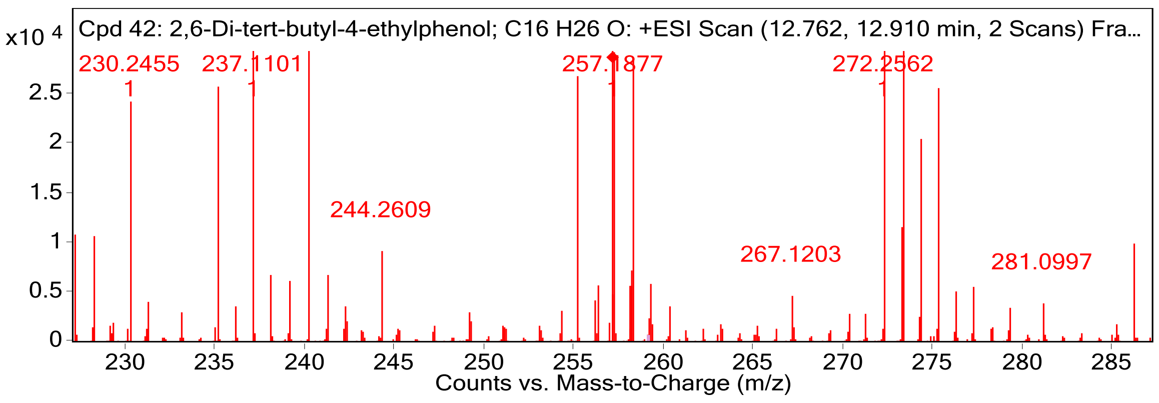

MS Spectrum Peak List

m/z	*Calc m/z*	Diff(ppm)	z	Abund	Formula	Ion

124.0861			1	90511.34		
129.9127				60973.67		
131.9098				58763.47		
209.1156			1	55054.17		
257.1877	257.1876	-0.39	1	29261.67	C16 H26 O	(M+Na)+
258.192	258.191	-3.88	1	7206.41	C16 H26 O	(M+Na)+
272.2562			1	196349.02		
288.2512			1	117831.11		
311.183			1	105110.73		
387.1771			1	135812.88		

MSMS Spectrum

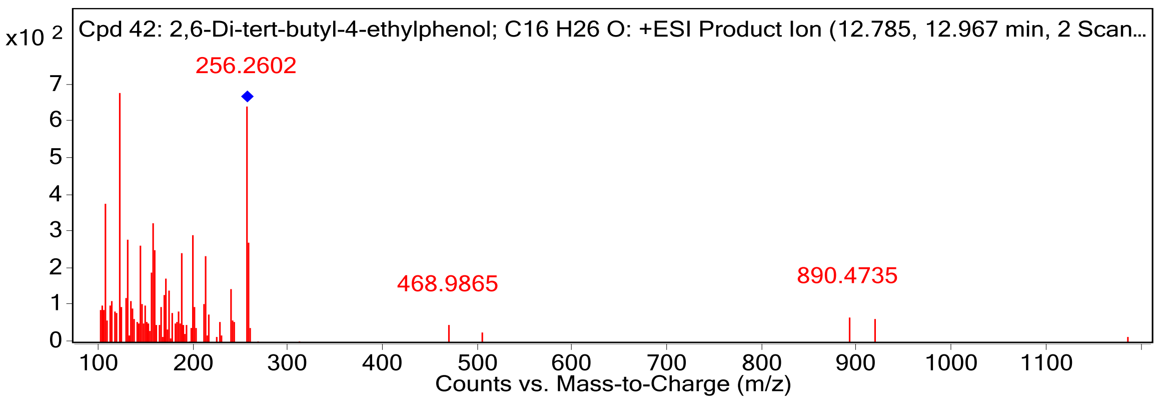

MS/MS Spectrum Peak List

m/z	z	Abund
107.0839		376.11
121.0998	1	679.91
131.0852		281.42
143.0853		263.54
157.0989	1	325.97
159.114		250.94
187.1101		242.31
199.1483		291.07
256.2602	2	641.72
257.1907		271

Compound Structure

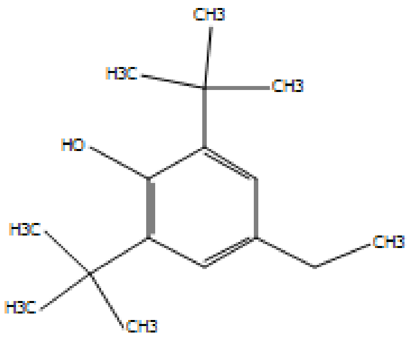

**Compound Label**	**Name**	***m/z***	**RT**	**Algorithm**	**Mass**
Cpd 43: Nigakilactone B; C22 H32 O6	**Nigakilactone B**	415.2082	13.531	Auto MS/MS	392.2189

MS Spectrum

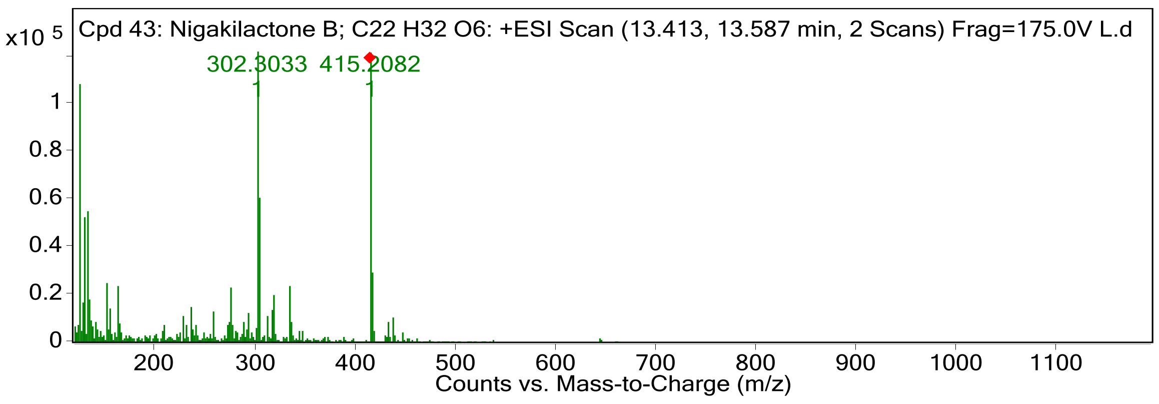

MS Zoomed Spectrum

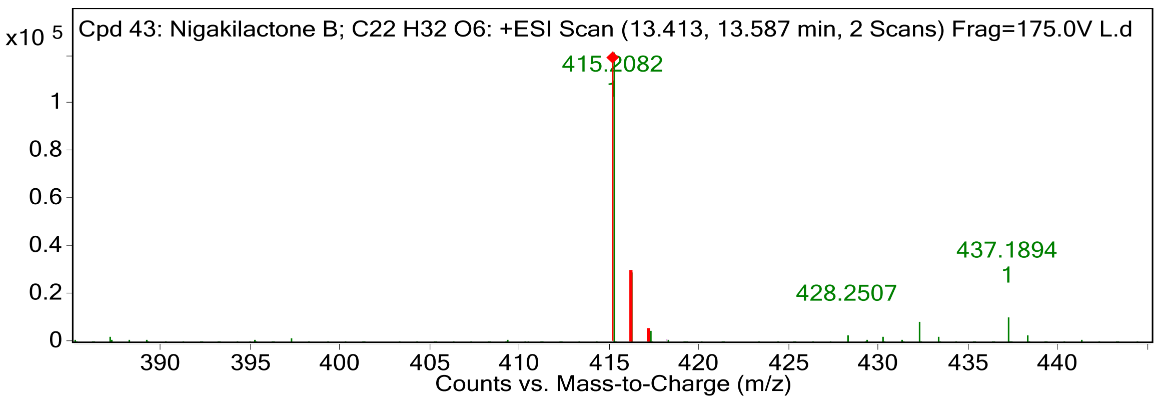

MS Spectrum Peak List

m/z	*Calc m/z*	Diff(ppm)	z	Abund	Formula	Ion
124.0859			1	108262.2		
125.0703			1	35828.19		
129.9126				52751.23		
131.9099				55048.37		
152.069			1	24941.36		
302.3033			1	320695.75		
303.306			1	60793.45		
415.2082	415.2091	2.24	1	121567.3	C22 H32 O6	(M+Na)+
416.2113	416.2125	2.84	1	29560.23	C22 H32 O6	(M+Na)+
417.2151	417.2152	0.15	1	5081.46	C22 H32 O6	(M+Na)+

MSMS Spectrum

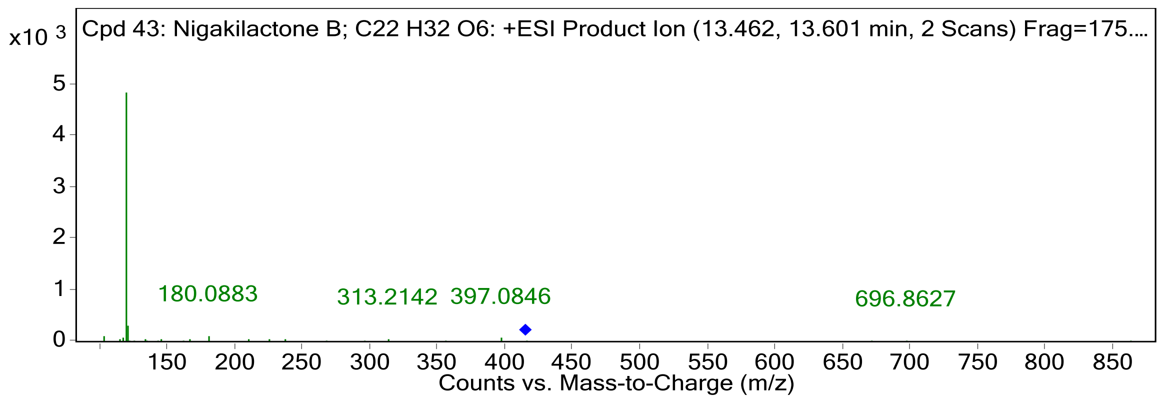

MS/MS Spectrum Peak List

m/z	z	Abund
103.0519		123.07
117.0694		91.82
118.847	1	69.54
119.028	2	104.94
119.0844	1	4857.3
120.0895	1	315.17
166.1199		70.71
180.0883		125.03
313.2142		62.5
397.0846		75.35

Compound Structure

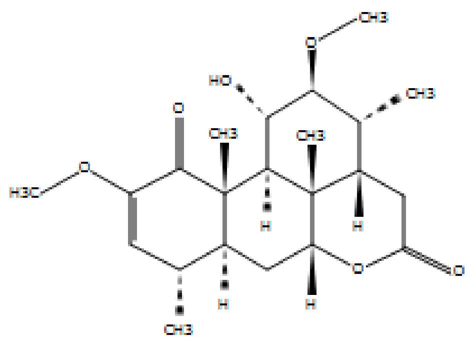

Compound Label	Name	*m/z*	RT	Algorithm	Mass
Cpd 44: Sphinganine; C18 H39 N O2	**Sphinganine**	302.3027	13.833	Auto MS/MS	301.2954

MS Spectrum

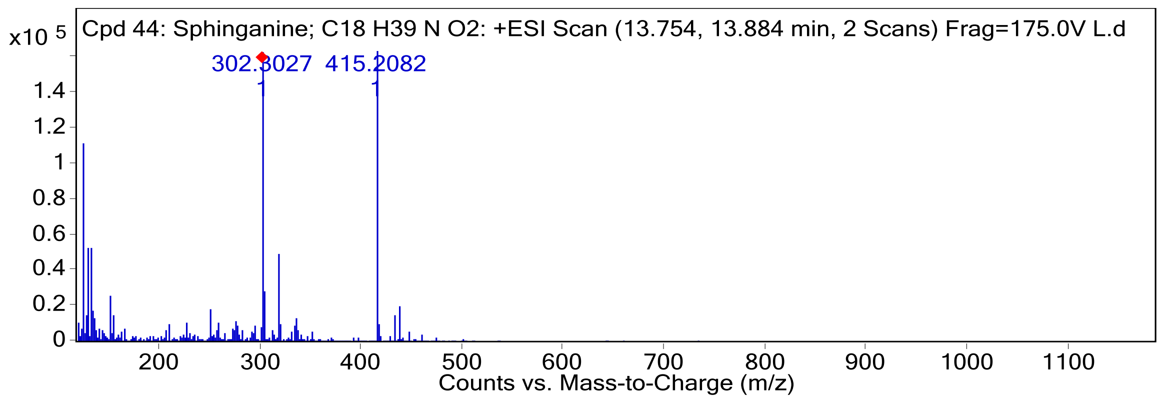

MS Zoomed Spectrum

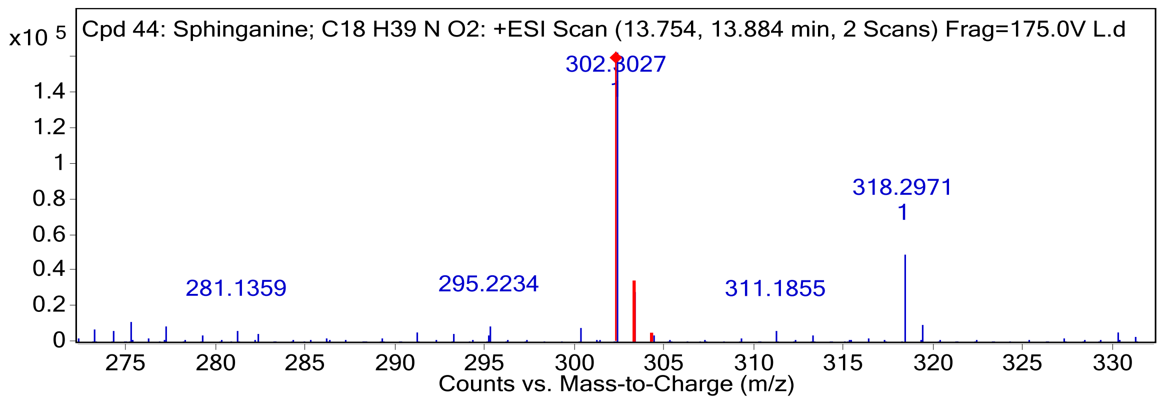

MS Spectrum Peak List

m/z	*Calc m/z*	Diff(ppm)	z	Abund	Formula	Ion
124.0859				111544.52		
125.0705				37886.02		
129.9125				52500.86		
131.9097				52820.42		
302.3027	302.3054	8.8	1	162831.2	C18 H39 N O2	(M+H)+
303.3058	303.3087	9.29	1	28474.8	C18 H39 N O2	(M+H)+
304.3065	304.3116	16.58	1	3898.64	C18 H39 N O2	(M+H)+
318.2971			1	49880.94		
415.2082			1	224244.72		
416.2111			1	56632.77		

MSMS Spectrum

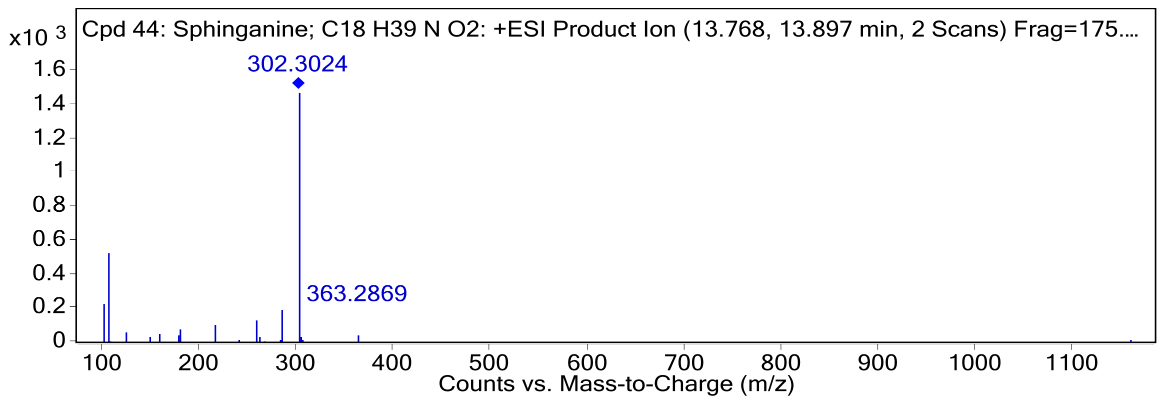

MS/MS Spectrum Peak List

m/z	z	Abund
102.0898		228.42

106.0852		525.25
181.0785		80.27
217.1026		105.54
258.2732		130.9
284.2919		192.4
285.3135		69.56
302.3024	1	1473.09
303.2982	1	116.78
303.317		85.12

Compound Structure

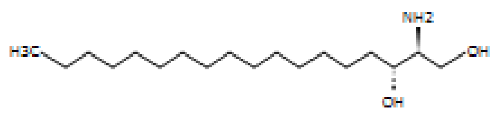

Compound Label	Name	*m/z*	RT	Algorithm	Mass
Cpd 45: 18-Nor-4(19),8,11,13-abietatetraene; C19 H26	**18-Nor-4(19),8,11,13-abietatetraene**	277.1954	16.38	Auto MS/MS	254.2062

MS Spectrum

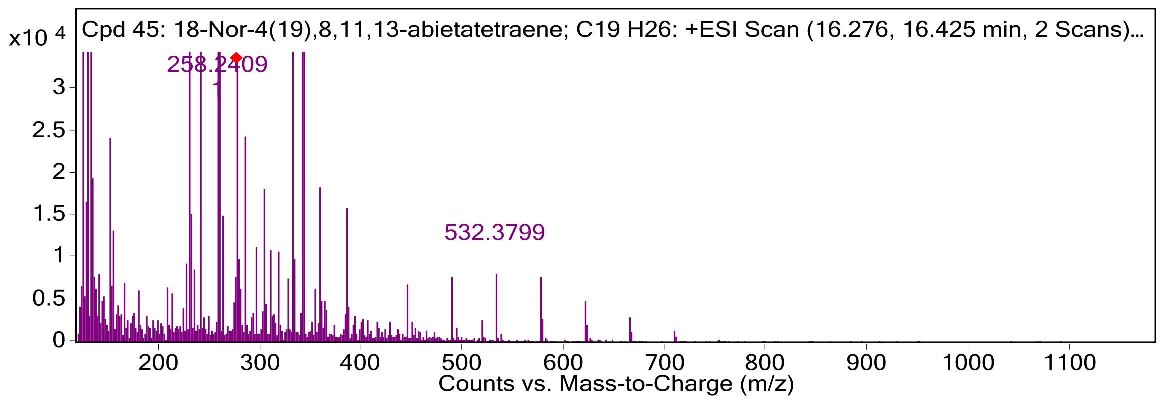

MS Zoomed Spectrum

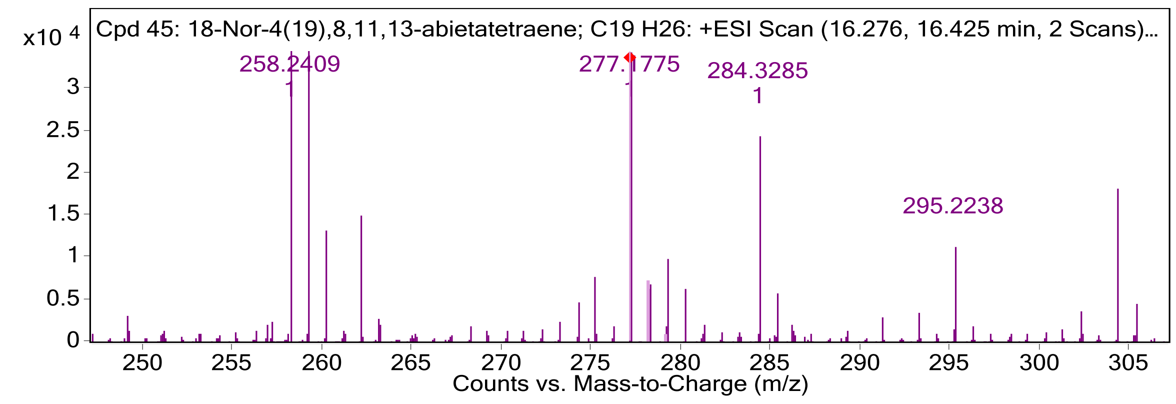

MS Spectrum Peak List

m/z	z	Abund
124.0859	1	115647.84
129.9125		51649.66
131.9097		56313.42
230.2455	1	90177.47
240.2303	1	39958.91
258.2409	1	856588.25
259.2438	1	139308.16
332.3282	1	40242.75
341.0909	1	181445.73
342.0939	1	40853.18

MSMS Spectrum

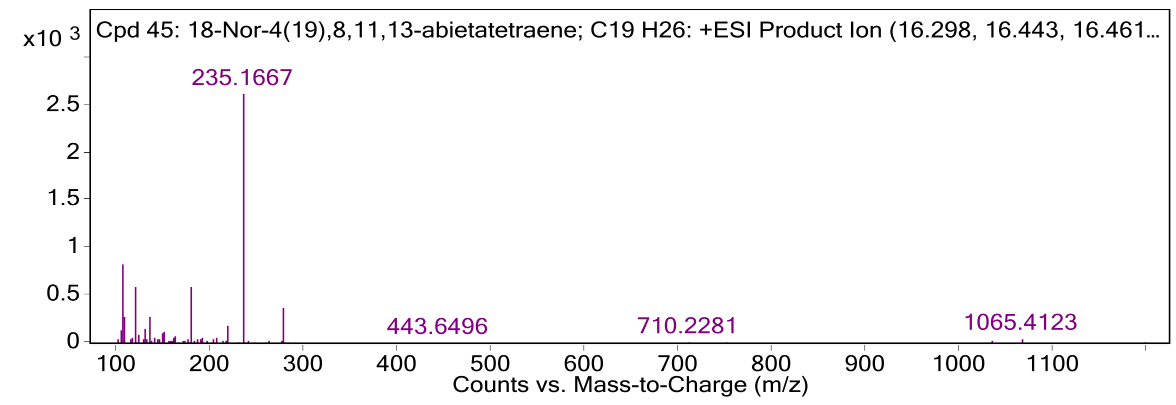

MS/MS Spectrum Peak List

m/z	z	Abund
105.069		139.81
107.084	1	835.6
109.0999		284.43
121.0992	1	601.49
131.0827		163.44
135.1153		282.1
179.1043	1	591.95
219.1362		195.87
235.1667	1	2623.19
277.1783		371.18

Compound Structure

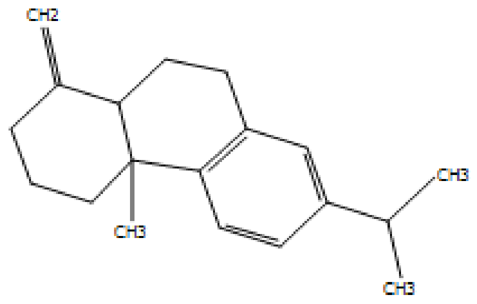

Compound Label	Name	*m/z*	RT	Algorithm	Mass
Cpd 46: Irinotecan; C33 H38 N4 O6	**Irinotecan**	609.2662	19.627	Auto MS/MS	586.2768

MS Spectrum

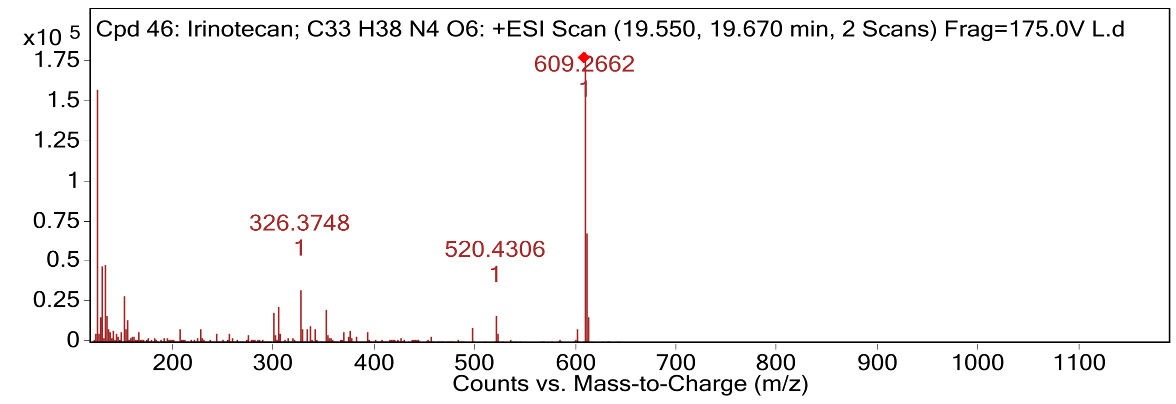

MS Zoomed Spectrum

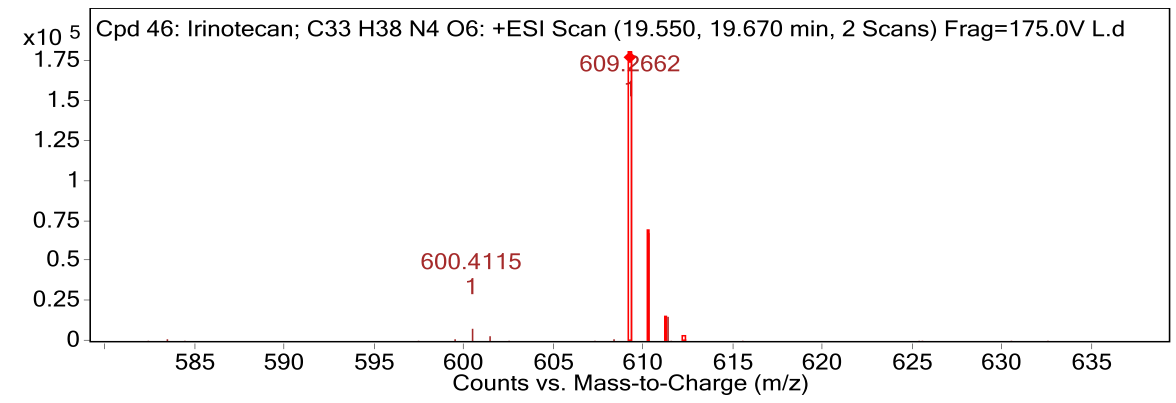

MS Spectrum Peak List

m/z	*Calc m/z*	Diff(ppm)	z	Abund	Formula	Ion

124.0859			1	157260.44		
125.0704			1	46550.24		
129.9124				47702.1		
131.9097				47993.73		
152.069			1	28562.25		
326.3748			1	32922.8		
609.2662	609.2684	3.61	1	180780.81	C33 H38 N4 O6	(M+Na)+
610.2689	610.2715	4.33	1	67671.37	C33 H38 N4 O6	(M+Na)+
611.2711	611.2743	5.31	1	16252.28	C33 H38 N4 O6	(M+Na)+
612.2751	612.2771	3.24	1	2209.46	C33 H38 N4 O6	(M+Na)+

MSMS Spectrum

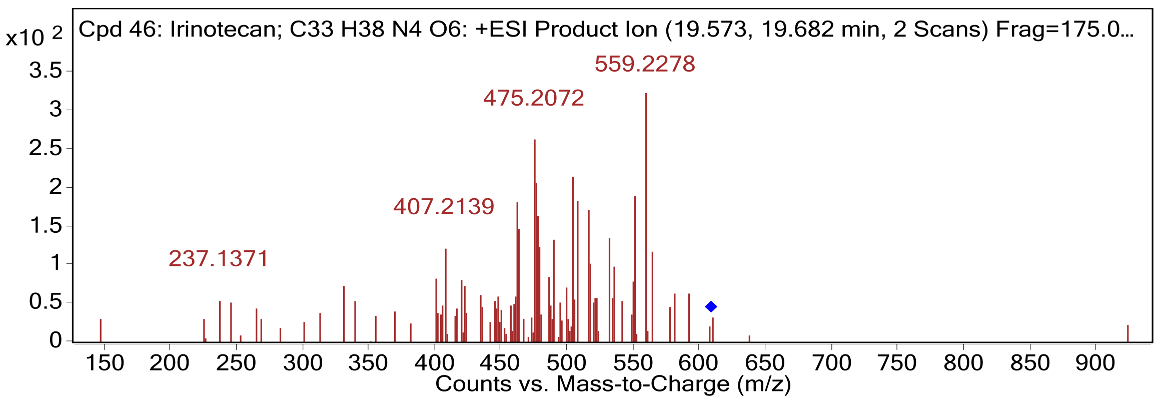

MS/MS Spectrum Peak List

m/z	z	Abund
461.1843		182.57
462.2068	1	147.3
475.2072		263.6
476.2122		207.64
477.2362		165.45
503.239	1	214.61
507.2297		183.73
515.2363		172.68
550.2554	2	190.08
559.2278		324.06

Compound Structure

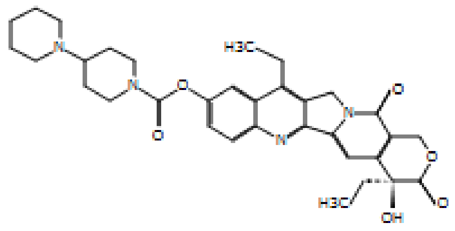

Compound Label	Name	*m/z*	RT	Algorithm	Mass
Cpd 47: Oxidized dinoflagellate luciferin; C33 H38 N4 O7	**Oxidized dinoflagellate luciferin**	625.2612	19.985	Auto MS/MS	602.2719

MS Spectrum

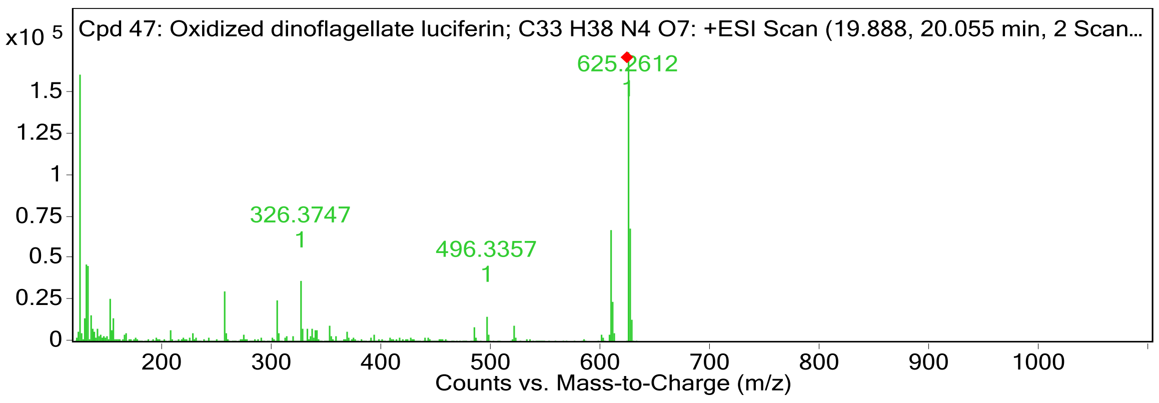

MS Zoomed Spectrum

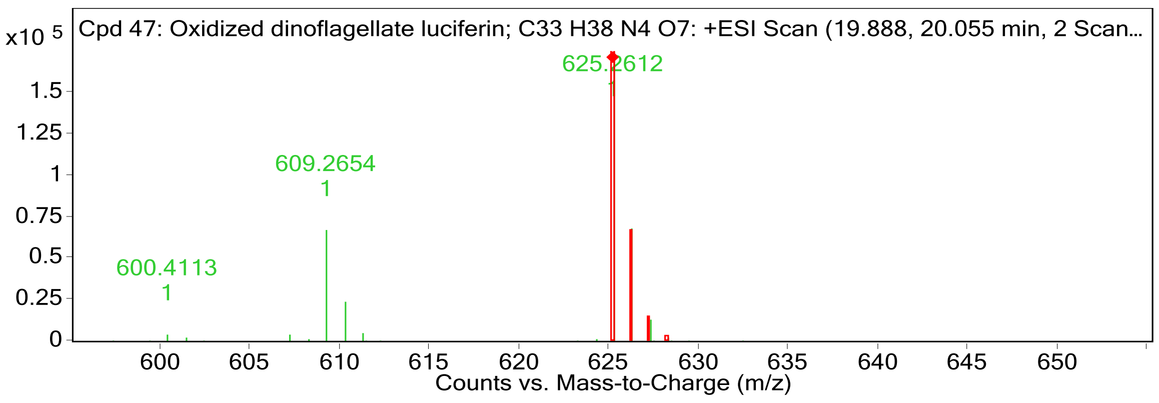

MS Spectrum Peak List

m/z	*Calc m/z*	Diff(ppm)	z	Abund	Formula	Ion
124.0859			1	161175.22		
125.0705			1	48975.73		
129.9125				46912.89		
131.9097				45577.61		
326.3747			1	36475.81		
609.2654			1	67421.86		
625.2612	625.2633	3.3	1	174584.91	C33 H38 N4 O7	(M+Na)+
626.2641	626.2664	3.65	1	67963.42	C33 H38 N4 O7	(M+Na)+
627.2666	627.2692	4.14	1	13581.64	C33 H38 N4 O7	(M+Na)+
628.2688	628.2719	4.91	1	2361.07	C33 H38 N4 O7	(M+Na)+

MSMS Spectrum

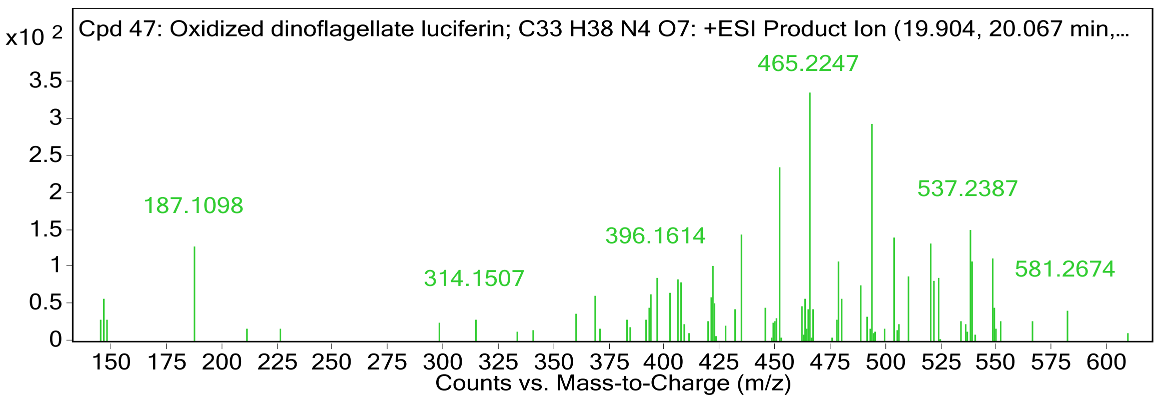

MS/MS Spectrum Peak List

m/z	z	Abund
187.1098		128.17
434.2342		145.39
451.2062		236.58
465.2247	1	337.47
478.2297	1	109.28
493.255		294.3
503.2472	1	140.62
519.2332		133.99
537.2387		151.15
547.2291	1	112.65

Compound Structure

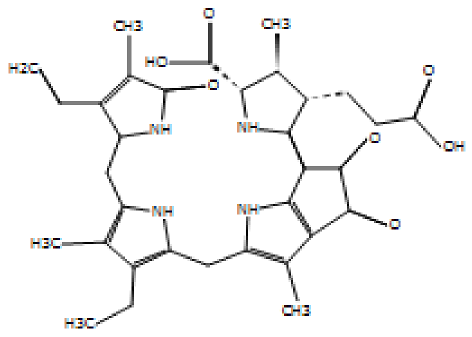

Compound Label	Name	*m/z*	RT	Algorithm	Mass
Cpd 48: Irinotecan; C33 H38 N4 O6	**Irinotecan**	609.2665	20.247	Auto MS/MS	586.2771

MS Spectrum

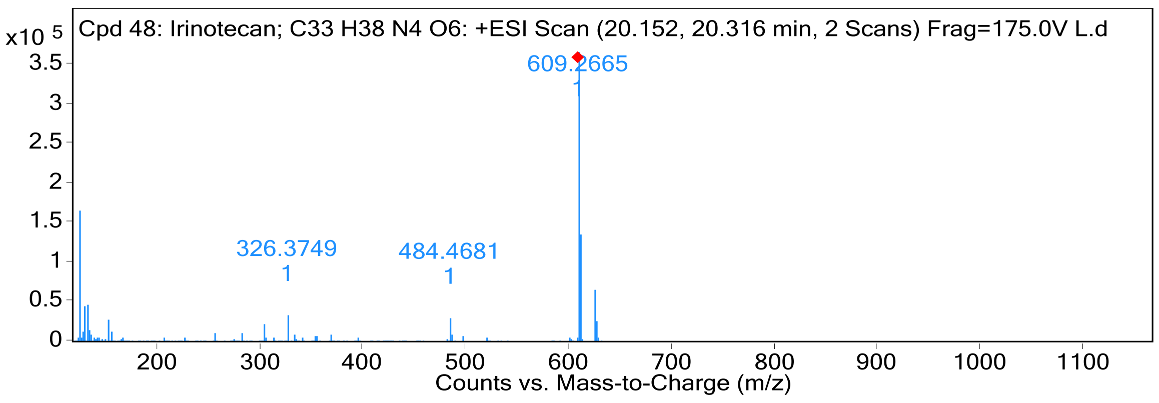

MS Zoomed Spectrum

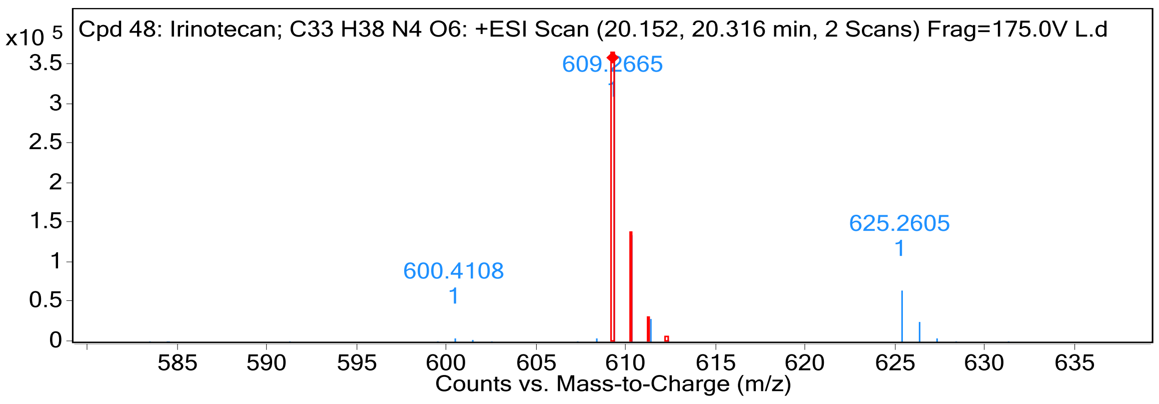

MS Spectrum Peak List

m/z	*Calc m/z*	Diff(ppm)	z	Abund	Formula	Ion
124.0859			1	165827.97		
125.0705			1	44403.75		
129.9126				44945.3		
131.9097				46827.45		
326.3749			1	33744.71		
609.2665	609.2684	3.07	1	365854.66	C33 H38 N4 O6	(M+Na)+
610.2694	610.2715	3.39	1	135608.95	C33 H38 N4 O6	(M+Na)+
611.2712	611.2743	5.07	1	29332.77	C33 H38 N4 O6	(M+Na)+
612.2739	612.2771	5.23	1	4419.05	C33 H38 N4 O6	(M+Na)+
625.2605			1	66425.27		

MSMS Spectrum

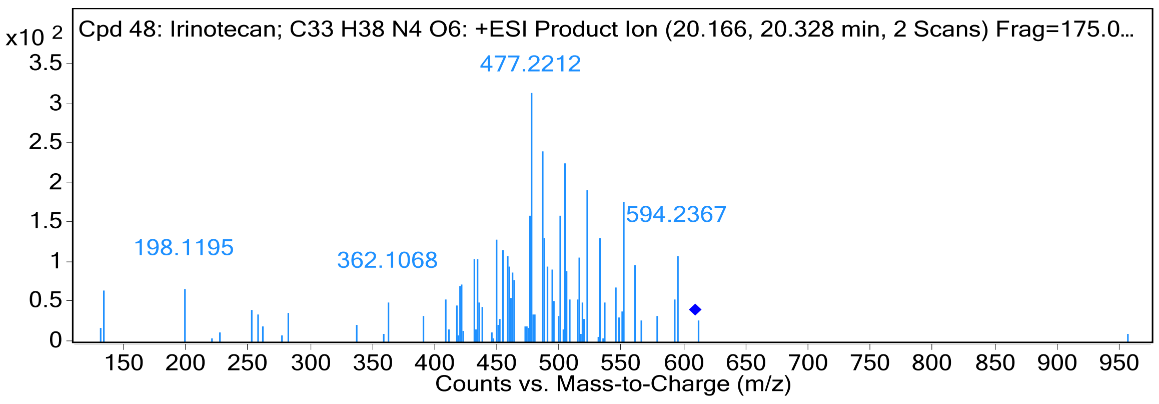

MS/MS Spectrum Peak List

m/z	z	Abund
449.2263	1	130.22

475.2078		160.05
477.2212	1	315.22
485.23	1	240.31
487.2481		130.94
500.2166		160.07
503.2379	1	226.12
521.2516	2	191.09
531.2444		132.05
550.2606		177.17

Compound Structure

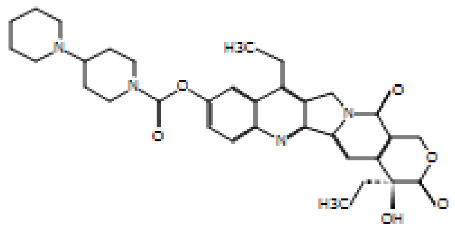

Compound Label	*m/z*	RT	Algorithm
Compound 49	568.4225	23.149	Auto MS/MS

MS Spectrum

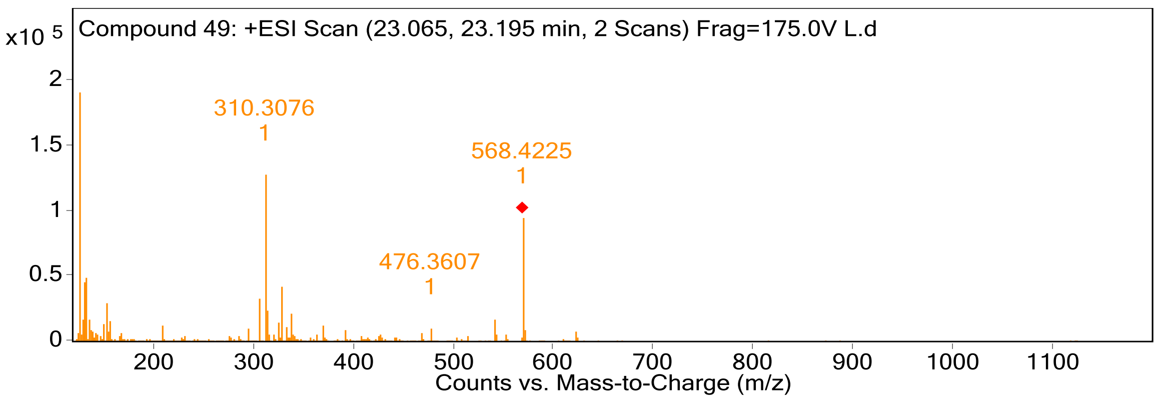

MS Zoomed Spectrum

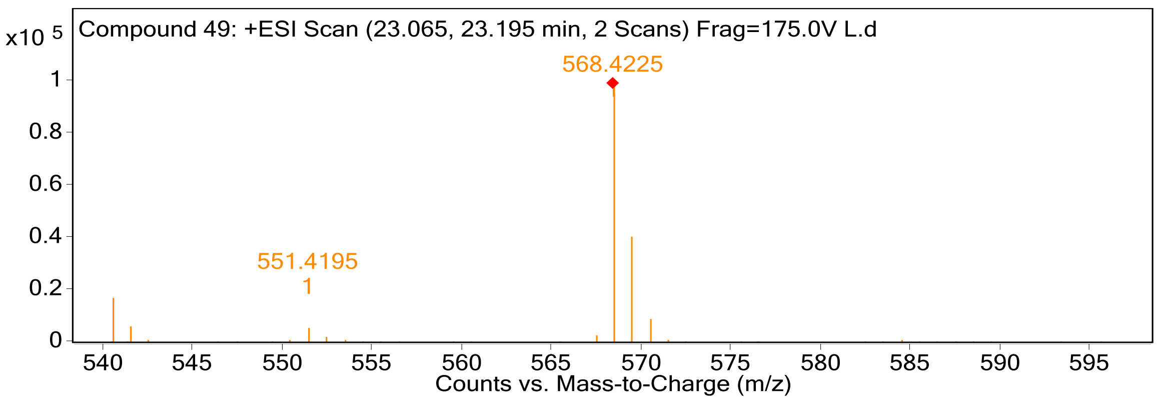

MS Spectrum Peak List

m/z	z	Abund
124.086	1	191180.97
125.0704	1	52272.62
129.9125		45270.61
131.9097		49661.47
310.3076	1	128342.39
326.375	1	42368.04
568.4225	1	95438.86
569.4259	1	40617.28
570.4288	1	9203.41
571.4335	1	1269.39

MSMS Spectrum

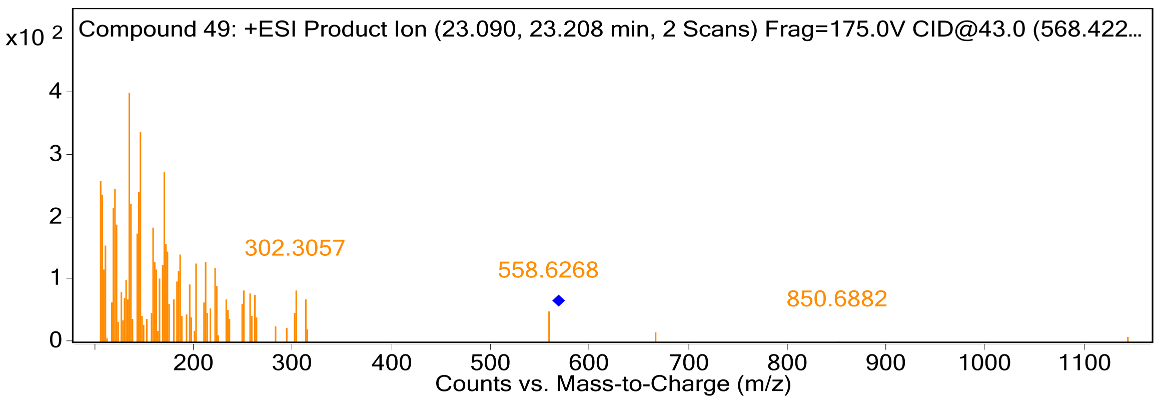

MS/MS Spectrum Peak List

m/z	z	Abund
105.0683		258.32
107.0897		236.81
117.0693		216.02
119.0831	2	245.81
121.1003		190.03
133.099		400.81
135.1152		222.39
143.0846		241.28
145.0994	1	337.46
169.1003	1	272.94

Compound Label	*m/z*	RT	Algorithm
Compound 50	568.4227	23.787	Auto MS/MS

MS Spectrum

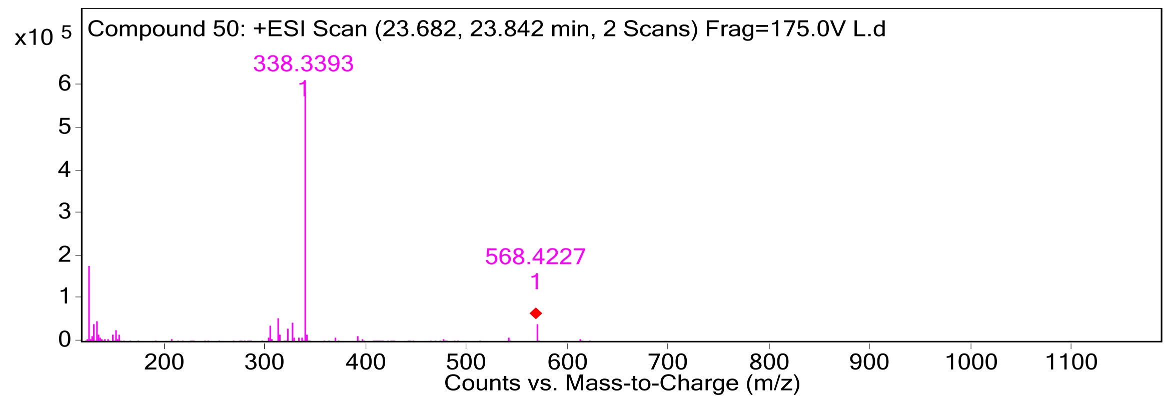

MS Zoomed Spectrum

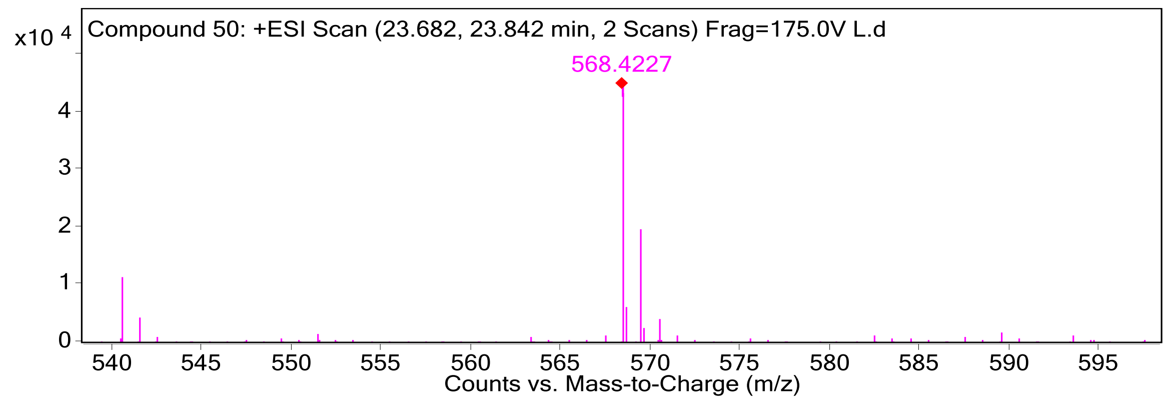

MS Spectrum Peak List

m/z	z	Abund
124.086	1	180061.02
125.0702	1	49308.72
131.9097		48080.26
312.3232	1	55261.02
338.3393	1	584651.94
339.3426	1	134043.84
568.4227	1	43430.94
569.4257	1	19717.06
570.43	1	4249.69
571.4321	1	1172.2

MSMS Spectrum

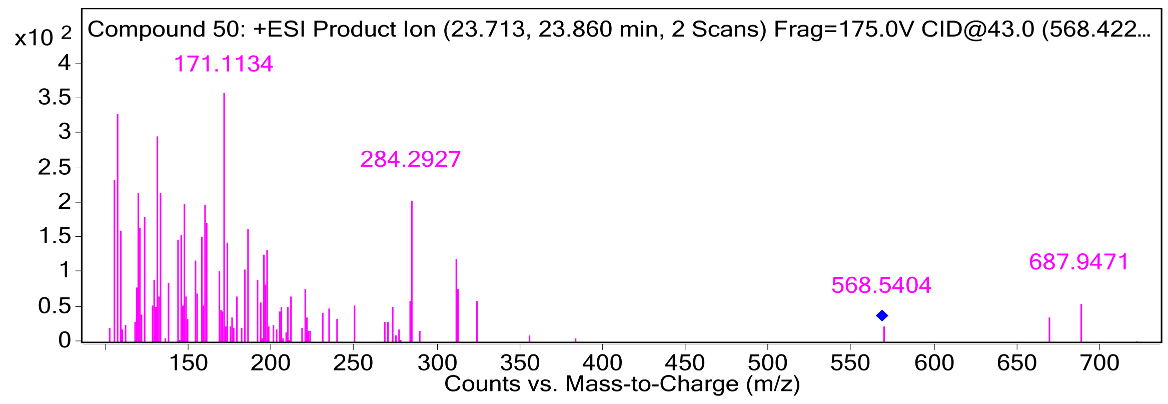

MS/MS Spectrum Peak List

m/z	z	Abund
105.0683		232.94
107.0836		328.27
119.0828		213.64
123.1143		180.51
131.0836	1	295.47
133.0994		214
147.1154		200.65
159.1143		198.17
171.1134		359.19
284.2927		204.04

--- End Of Report ---
