# Supplementary material for: Metabolomic Profiling of Leptadenia reticulata: Unveiling Therapeutic Potential for Inflammatory Diseases through Network Pharmacology and Docking Studies
Source: Pharmaceuticals (Basel). 2024 Mar 26;17(4):423. doi: 10.3390/ph17040423 (PMC11054655; doi:10.3390/ph17040423)
Supplement: Supplementary file 1 [file pharmaceuticals-17-00423-s001.zip › HR LCMS chromatogram/method.pdf]

Acquisition Method Report

Agilent Technologies

Acquisition Method Info

**Method Name**	metabolite_ESI_+VE_MSMS.m
**Method Path**	D:\MassHunter\methods\2022\metabolite_ESI_+VE_MSMS.m
**Method Description**	Default Method

Device List

- HiP Sampler
- Binary Pump
- Column Comp.
- DAD
- Q-TOF

TOF/Q-TOF Mass Spectrometer

**Component Name**	MS Q-TOF	**Component Model**	G6550A
**Ion Source**	Dual AJS ESI	**Stop Time (min)**	30.00
**Can wait for temp.**	Enable	**Fast Polarity**	N/A
**MS Abs. threshold**	200	**MS Rel. threshold(%)**	0.010
**MS/MS Abs. threshold**	5	**MS/MS Rel. threshold(%)**	0.010
**Tune File**	AutoTune (3).tun		

Time Segments

Time Segment #	Start Time (min)	Diverter Valve State	Storage Mode	Ion Mode
1	0	MS	Both	Dual AJS ESI

Time Segment 1

Acquisition Mode AutoMS2

**MS Min Range (m/z)**	120
**MS Max Range (m/z)**	1200
**MS Scan Rate (spectra/sec)**	1.00
**MS/MS Scan Rate (spectra/sec)**	1.00
**Isolation Width MS/MS**	Medium (~4 amu)

Ramped Collision Energy

Charge	Slope	Offset
1	8	-2.6
2	6	-2.6
3	4	-2.6

Auto MS/MS Preferred/Exclude Table

Mass	Delta Mass (ppm)	Charge	Type	Retention Time (min)	Delta Ret. Time (min)	Isolation Width	Collision Energy
197.8075	500	1	Exclude	0		Medium (~4 amu)	

Precursor Selection

**Max Precursors Per Cycle**	10
**Threshold (Abs)**	10000
**Threshold (Rel)(%)**	0.010
**Precursor abundance based scan speed**	Yes
**Target (counts/spectrum)**	25000.000
**Use MS/MS accumulation time limit**	Yes
**Use dynamic precursor rejection**	No
**Purity Stringency (%)**	100.000
**Purity Cutoff (%)**	30.000
**Isotope Model**	Common
**Active exclusion enabled**	Yes
**Active exclusion excluded after (spectra)**	1
**Active exclusion released after (min)**	0.20
**Sort precursors**	By abundance only

Charge State Preference

Selected Charges
1
2
Unk

Source Parameters

Parameter	Value
Gas Temp (°C)	250
Gas Flow (l/min)	13
Nebulizer (psig)	35
SheathGasTemp	300
SheathGasFlow	11

Scan Segments

Scan Seg #	Ion Polarity	Collision Energy
1	Positive	0

Scan Segment 1

Scan Source Parameters

Parameter	Value
VCap	3500
Nozzle Voltage (V)	1000
Fragmentor	175
Skimmer1	65
OctopoleRFPeak	750

ReferenceMasses

**Ref Mass Enabled**	Disabled
**Ref Nebulizer (psig)**	

Chromatograms

Chrom Type	Label	Offset	Y-Range
TIC	TIC	15	10000000

Name: HiP Sampler	Model: G4226A
**Auxiliary**	
Draw Speed	100.0 µL/min
Eject Speed	100.0 µL/min
Draw Position Offset	0.0 mm
Wait Time After Drawing	2.0 s
Sample Flush Out Factor	5.0
Vial/Well bottom sensing	Yes
**Injection**	
Injection Mode	Injection with needle wash
Injection Volume	5.00 µL
**Needle Wash**	
Needle Wash Location	Flush Port
Wash Time	3.0 s
**High throughput**	
Automatic Delay Volume Reduction	No
**Overlapped Injection**	
Enable Overlapped Injection	No
**Valve Switching**	
Valve Movements	0
**Valve Switch Time 1**	
Switch Time 1 Enabled	Yes
Switch Time 1	0.01 min
**Valve Switch Time 2**	
Switch Time 2 Enabled	No
**Valve Switch Time 3**	
Switch Time 3 Enabled	No
**Valve Switch Time 4**	
Switch Time 4 Enabled	No
**Stop Time**	
Stoptime Mode	As pump/No limit
**Post Time**	
Posttime Mode	Off

Name: Binary Pump Model: G4220B

**Flow**	0.300 mL/min
**Use Solvent Types**	Yes
**Stroke Mode**	Synchronized
**Low Pressure Limit**	0.00 bar
**High Pressure Limit**	1200.00 bar
**Max. Flow Ramp Up**	100.000 mL/min²
**Max. Flow Ramp Down**	100.000 mL/min²
**Expected Mixer**	No check

Stroke A

**Automatic Stroke Calculation A**	Yes

Stop Time

**Stoptime Mode**	Time set
**Stoptime**	35.00 min

Post Time

**Posttime Mode**	Off

Solvent Composition

	Channel	Ch. 1 Solv.	Name 1	Ch2 Solv.	Name 2	Selected	Used	Percent
1	A	100.0 % Water V.02	0.1% FA in water	100.0 % Water V.02	0.1% FA in water	Ch. 2	Yes	95.00 %
2	B	100.0 % Methanol V.03		100.0 % Acetonitrile V.02		Ch. 2	Yes	5.00 %

Timetable

	Time	A	B	Flow	Pressure
1	1.00 min	95.00 %	5.00 %	0.300 mL/min	1200.00 bar
2	25.00 min	0.00 %	100.00 %	0.300 mL/min	1200.00 bar
3	30.00 min	0.00 %	100.00 %	0.300 mL/min	1200.00 bar
4	31.00 min	95.00 %	5.00 %	0.300 mL/min	1200.00 bar
5	35.00 min	95.00 %	5.00 %	0.300 mL/min	1200.00 bar

Name: Column Comp. Model: G1316C

**Ready when front door open**	Yes

Left Temperature Control

**Temperature Control Mode**	Temperature Set
**Temperature**	40.00 °C

Enable Analysis Left Temperature

**Enable Analysis Left Temperature On**	Yes
**Enable Analysis Left Temperature Value**	0.80 °C

Right Temperature Control

**Right temperature Control Mode**	Temperature Set
**Right temperature**	40.00 °C

Enable Analysis Right Temperature

**Enable Analysis Right Temperature On**	Yes
**Enable Analysis Right Temperature Value**	0.80 °C

Stop Time

**Stoptime Mode**	As pump/injector

Post Time

**Posttime Mode**	Off

Name: DAD **Model:** G4212B

**Peakwidth**	>0.10 min (2.0 s response time) (2.5 Hz)
**UV Lamp Required**	No

Analog Output 1

**Analog 1 Zero Offset**	5 %
**Analog 1 Attenuation**	1000 mAU

Signals

Prepare Mode

**Margin for negative Absorbance**	100 mAU

Autobalance

**Autobalance Prerun**	Yes
**Autobalance Postrun**	No

Spectrum

**Spectrum Range WL from**	190.0 nm
**Spectrum Range WL to**	600.0 nm
**Spectrum Step**	2.0 nm
**Spectrum Store**	All

Stoptime

**Stoptime Mode**	As pump/injector

Posttime

**Posttime Mode**	Off

Signals

Signal table

	Use Sig.	Signal	Wavelength	Bandwidth	Use Ref.
1	Yes	Signal A	280.0 nm	4.0 nm	No
2	Yes	Signal B	254.0 nm	4.0 nm	No
3	Yes	Signal C	387.0 nm	4.0 nm	No
4	Yes	Signal D	340.0 nm	4.0 nm	No
5	Yes	Signal E	360.0 nm	4.0 nm	No
6	Yes	Signal F	420.0 nm	4.0 nm	No
7	Yes	Signal G	460.0 nm	4.0 nm	No
8	No	Signal H			
