# Supplementary material for: Metabolomic Profiling of Leptadenia reticulata: Unveiling Therapeutic Potential for Inflammatory Diseases through Network Pharmacology and Docking Studies
Source: Pharmaceuticals (Basel). 2024 Mar 26;17(4):423. doi: 10.3390/ph17040423 (PMC11054655; doi:10.3390/ph17040423)
Supplement: Supplementary file 1 [file pharmaceuticals-17-00423-s001.zip › HR LCMS chromatogram/M_-VE_CompoundReport.pdf]

Qualitative Compound Report

**Data File**	M_-VE.d	**Sample Name**	M
**Sample Type**	Sample	**Position**	P1-A6
**Instrument Name**	QTOF	**User Name**	
**Acq Method**	metabolite_ESI_-VE_MSMS.m	**Acquired Time**	5/8/2023 4:42:44 AM
**IRM Calibration Status**	Success	**DA Method**	default.m
**Comment**			

**Sample Group**		**Info.**
**Acquisition SW Version**	6200 series TOF/6500 series Q-TOF B.05.01 (B5125.3)	

Compound Table

Compound Label	RT	Mass	Abund	Name	Formula	Tgt Mass	Diff (ppm)	MFG Formula	DB Formula	DB Diff (ppm)	Hits (DB)
Cpd 1: Xanthyletine; C14 H12 O3	2.068	228.0788	409	Xanthyletine	C14 H12 O3			C14 H12 O3	C14 H12 O3	-0.68	
Cpd 2: C19 H14 O2	5.273	274.0992	291		C19 H14 O2	274.0994	-0.58	C19 H14 O2	C19 H14 O2		
Cpd 3: Citric acid; C6 H8 O7	16.404	192.0276	611	Citric acid	C6 H8 O7			C6 H8 O7	C6 H8 O7	-2.96	
Compound 4	1.461		15120								
Compound 5	1.495		12246								
Cpd 6: Ribose-1-arsenate; C5 H11 As O8	1.526	273.9678		Ribose-1-arsenate	C5 H11 As O8			C5 H11 As O8	C5 H11 As O8	-3.06	2
Cpd 7: 2,3,5,7,9-Pentathiadecane 2,2-dioxide; C5 H12 O2 S5	1.534	263.942	20574	2,3,5,7,9-Pentathiadecane 2,2-dioxide	C5 H12 O2 S5			C5 H12 O2 S5	C5 H12 O2 S5	7.95	1
Compound 8	1.539		20330								
Cpd 9: (-)-epicatechin-3'-O-glucuronide; C21 H24 O11	1.733	452.1332	12115	(-)-epicatechin-3'-O-glucuronide	C21 H24 O11			C21 H24 O11	C21 H24 O11	-3.04	10
Cpd 10: Tosyllysine Chloromethyl Ketone; C14 H21 Cl N2 O3 S	1.789	332.0935		Tosyllysine Chloromethyl Ketone	C14 H21 Cl N2 O3 S			C14 H21 Cl N2 O3 S	C14 H21 Cl N2 O3 S	7.99	10
Cpd 11: 5-Dehydro-4-deoxy-D-glucarate; C6 H8 O7	1.843	192.0304		5-Dehydro-4-deoxy-D-glucarate	C6 H8 O7			C6 H8 O7	C6 H8 O7	-17.82	10
Cpd 12: 3,7-Dimethyluric acid; C7 H8 N4 O3	1.92	196.0617	153630	3,7-Dimethyluric acid	C7 H8 N4 O3			C7 H8 N4 O3	C7 H8 N4 O3	-10.29	10
Cpd 13: GYKI 52466; C17 H15 N3 O2	1.94	293.1155		GYKI 52466	C17 H15 N3 O2			C17 H15 N3 O2	C17 H15 N3 O2	3.09	8
Cpd 14: 3,7-Dimethyluric acid; C7 H8 N4 O3	2.135	196.0614	29108	3,7-Dimethyluric acid	C7 H8 N4 O3			C7 H8 N4 O3	C7 H8 N4 O3	-8.82	10
Cpd 15: Ketamine; C13 H16 Cl N O	2.378	237.0905	18153	Ketamine	C13 H16 Cl N O			C13 H16 Cl N O	C13 H16 Cl N O	6.71	10
Cpd 16: Aprepitant; C23 H21 F7 N4 O3	6.011	534.1456		Aprepitant	C23 H21 F7 N4 O3			C23 H21 F7 N4 O3	C23 H21 F7 N4 O3	8.57	10
Cpd 17: CMP-N-glycoloylneuraminate; C20 H31 N4 O17 P	6.062	630.1417		CMP-N-glycoloylneuraminate	C20 H31 N4 O17 P			C20 H31 N4 O17 P	C20 H31 N4 O17 P	0.73	1
Cpd 18: Aprepitant; C23 H21 F7 N4 O3	6.391	534.1456		Aprepitant	C23 H21 F7 N4 O3			C23 H21 F7 N4 O3	C23 H21 F7 N4 O3	8.52	10
Cpd 19: CMP-N-glycoloylneuraminate; C20 H31 N4 O17 P	6.441	630.1414		CMP-N-glycoloylneuraminate	C20 H31 N4 O17 P			C20 H31 N4 O17 P	C20 H31 N4 O17 P	1.21	1
Cpd 20: Aprepitant; C23 H21 F7 N4 O3	6.686	534.1455	21347	Aprepitant	C23 H21 F7 N4 O3			C23 H21 F7 N4 O3	C23 H21 F7 N4 O3	8.85	10
Cpd 21: Polyethylene, oxidized; C12 H20 O5	8.44	244.1348		Polyethylene, oxidized	C12 H20 O5			C12 H20 O5	C12 H20 O5	-15.21	1
Cpd 22: Hemibrevetoxin B; C28 H42 O7	9.682	490.285	10058	Hemibrevetoxin B	C28 H42 O7			C28 H42 O7	C28 H42 O7	16.42	4
Cpd 23: (3b,6b,8a,12a)-8,12-Epoxy-7(11)-eremophilene-6,8,12-trimethoxy-3-ol; C18 H30 O5	11.121	326.2146		(3b,6b,8a,12a)-8,12-Epoxy-7(11)-eremophilene-6,8,12-trimethoxy-3-ol	C18 H30 O5			C18 H30 O5	C18 H30 O5	-16.12	10
Cpd 24: (3b,6b,8a,12a)-8,12-Epoxy-7(11)-eremophilene-6,8,12-trimethoxy-3-ol; C18 H30 O5	11.51	326.2145		(3b,6b,8a,12a)-8,12-Epoxy-7(11)-eremophilene-6,8,12-trimethoxy-3-ol	C18 H30 O5			C18 H30 O5	C18 H30 O5	-15.79	10
Cpd 25: (3b,6b,8a,12a)-8,12-Epoxy-7(11)-eremophilene-6,8,12-trimethoxy-3-ol; C18 H30 O5	11.895	326.2144	15067	(3b,6b,8a,12a)-8,12-Epoxy-7(11)-eremophilene-6,8,12-trimethoxy-3-ol	C18 H30 O5			C18 H30 O5	C18 H30 O5	-15.48	10
Cpd 26: Saponin H; C36 H58 O10	17.125	650.3962	14104	Saponin H	C36 H58 O10			C36 H58 O10	C36 H58 O10	10.42	8
Cpd 27: Hexazinone; C12 H20 N4 O2	18.076	252.1596		Hexazinone	C12 H20 N4 O2			C12 H20 N4 O2	C12 H20 N4 O2	-4.01	5
Cpd 28: Hexazinone; C12 H20 N4 O2	18.346	252.1595		Hexazinone	C12 H20 N4 O2			C12 H20 N4 O2	C12 H20 N4 O2	-3.62	5
Cpd 29: Hydroquinidine; C20 H26 N2 O2	21.674	326.1968		Hydroquinidine	C20 H26 N2 O2			C20 H26 N2 O2	C20 H26 N2 O2	7.95	10
Cpd 30: Hydroquinidine; C20 H26 N2 O2	22.039	326.1965		Hydroquinidine	C20 H26 N2 O2			C20 H26 N2 O2	C20 H26 N2 O2	8.9	10
Cpd 31: Hydroquinidine; C20 H26 N2 O2	22.372	326.1966		Hydroquinidine	C20 H26 N2 O2			C20 H26 N2 O2	C20 H26 N2 O2	8.71	10
Compound 32	22.494										
Compound 33	22.845										

Compound Label	Name	*m/z*	RT	Algorithm	Mass
Cpd 1: Xanthyletine; C14 H12 O3	**Xanthyletine**	287.0924	2.068	Find By Formula	228.0788

MS Spectrum

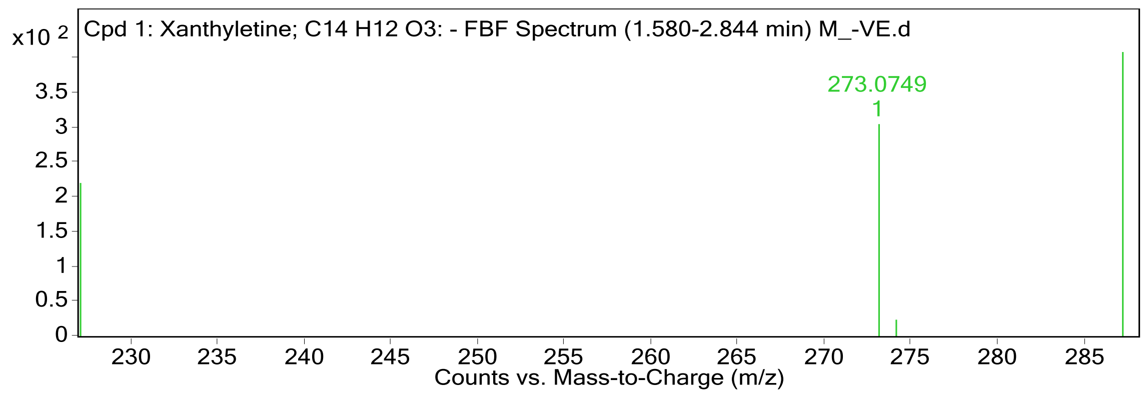

MS Zoomed Spectrum

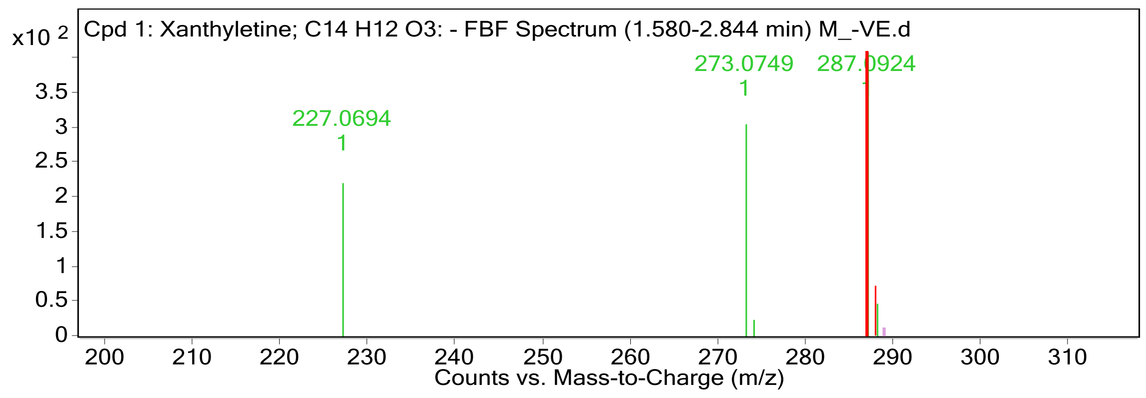

MS Spectrum Peak List

m/z	z	Abund	Formula	Ion
227.0694	1	220.58	C14 H12 O3	(M-H)-
273.0749	1	304.95	C14 H12 O3	(M+HCOO)-
274.0847	1	26.36	C14 H12 O3	(M+HCOO)-
287.0924	1	409.24	C14 H12 O3	(M+CH3COO)-
288.0981	1	48.49	C14 H12 O3	(M+CH3COO)-

Compound Structure

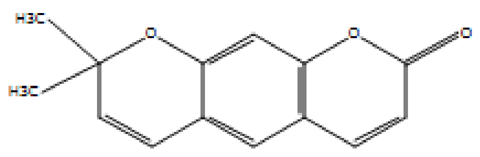

Compound Label	*m/z*	RT	Algorithm	Mass
Cpd 2: C19 H14 O2	319.0966	5.273	Find By Formula	274.0992

MS Spectrum

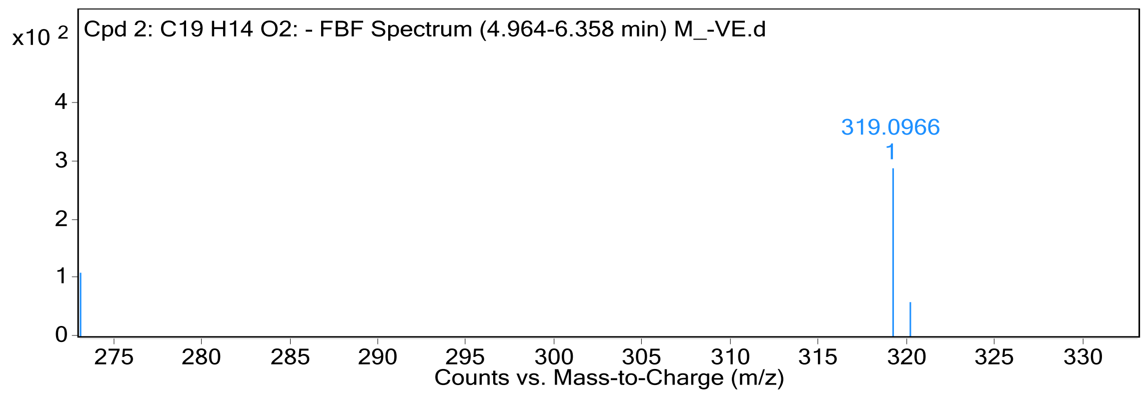

MS Zoomed Spectrum

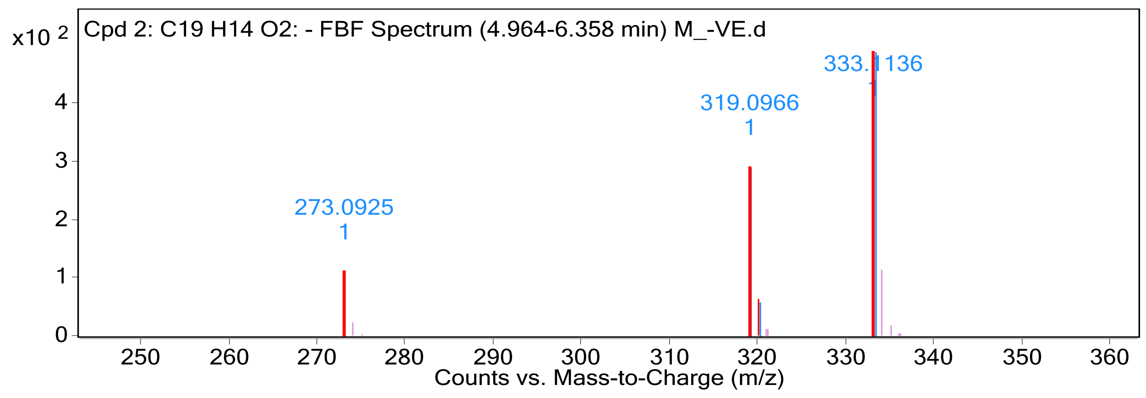

MS Spectrum Peak List

m/z	z	Abund	Formula	Ion
273.0925	1	110.8	C19 H14 O2	(M-H)-
319.0966	1	290.84	C19 H14 O2	(M+HCOO)-
320.0997	1	61.32	C19 H14 O2	(M+HCOO)-
333.1136	1	489.43	C19 H14 O2	(M+CH3COO)-

Compound Label	Name	*m/z*	RT	Algorithm	Mass
Cpd 3: Citric acid; C6 H8 O7	**Citric acid**	191.02	16.404	Find By Formula	192.0276

MS Spectrum

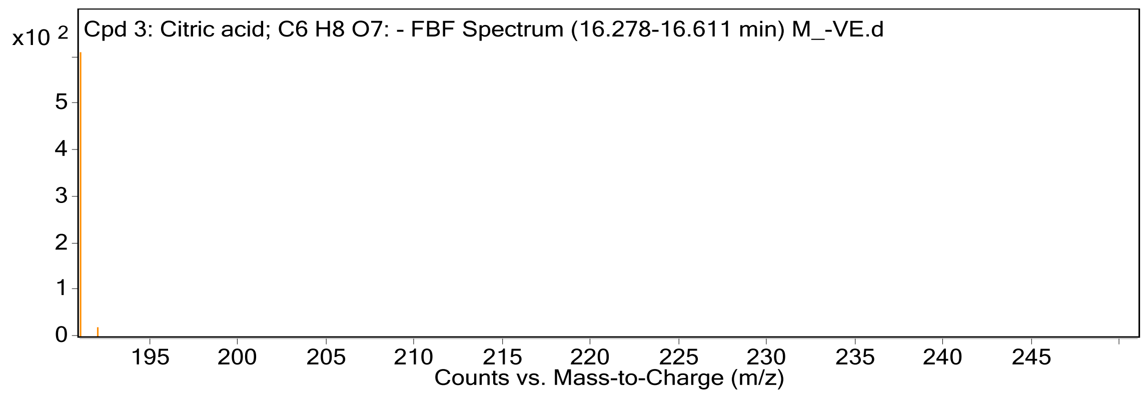

MS Zoomed Spectrum

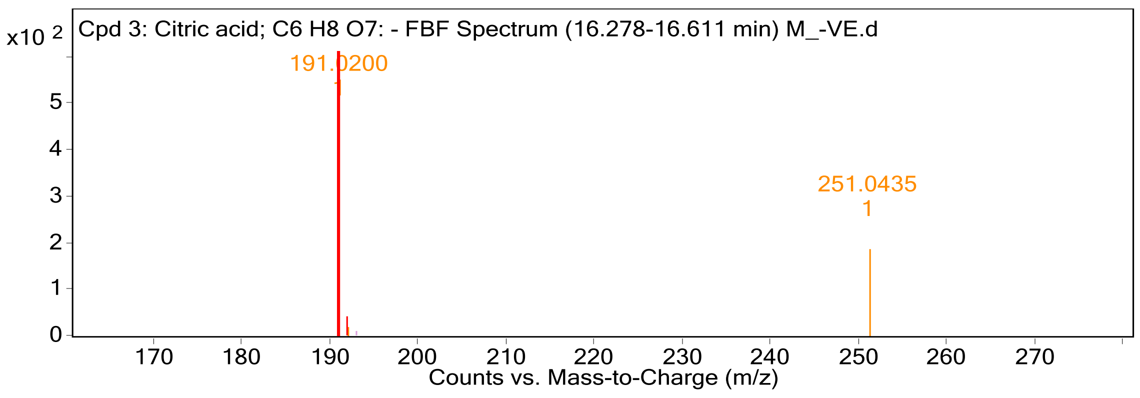

MS Spectrum Peak List

m/z	z	Abund	Formula	Ion
191.02	1	611.09	C6 H8 O7	(M-H)-
192.0331	1	20.51	C6 H8 O7	(M-H)-
251.0435	1	188.05	C6 H8 O7	(M+CH3COO)-

Compound Structure

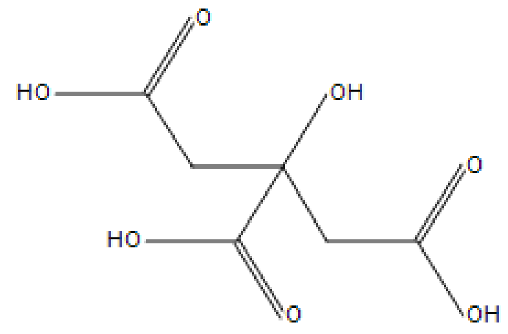

Compound Label	*m/z*	RT	Algorithm
Compound 4	392.8937	1.461	Auto MS/MS

MS Spectrum

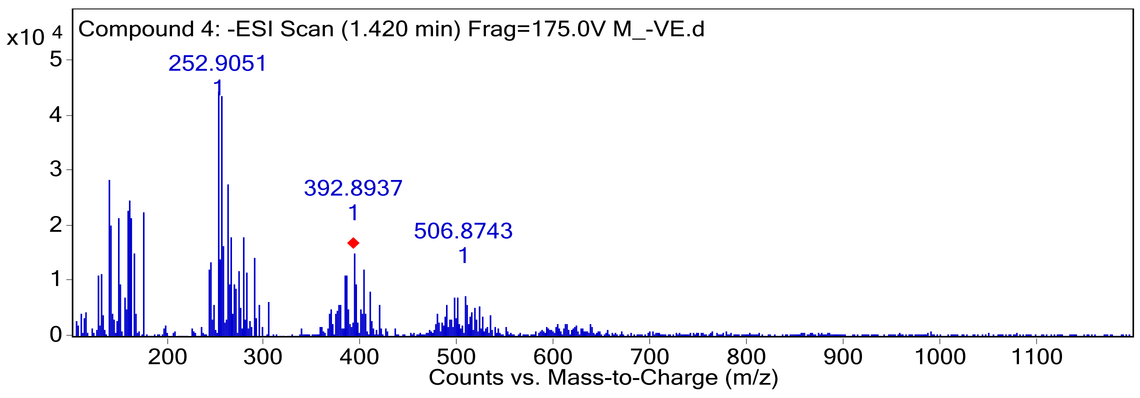

MS Zoomed Spectrum

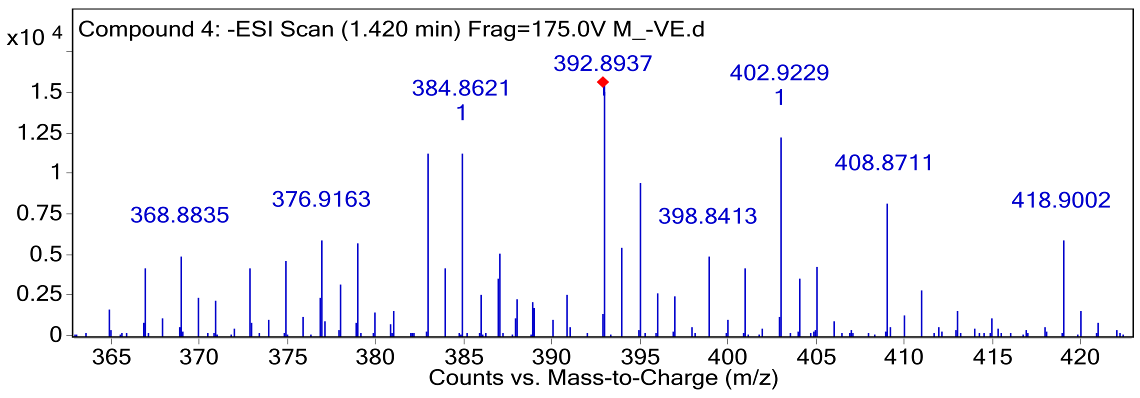

MS Spectrum Peak List

m/z	z	Abund
138.9234	1	28352.99
160.8449		24879.58
252.9051	1	44531.95
254.9023	1	43736.31
262.9343	1	27639.73
392.8937	1	15119.76
393.8946	1	5526
394.8918	1	9452.51
395.8921	1	2713.56
396.8879	1	2553.25

MSMS Spectrum

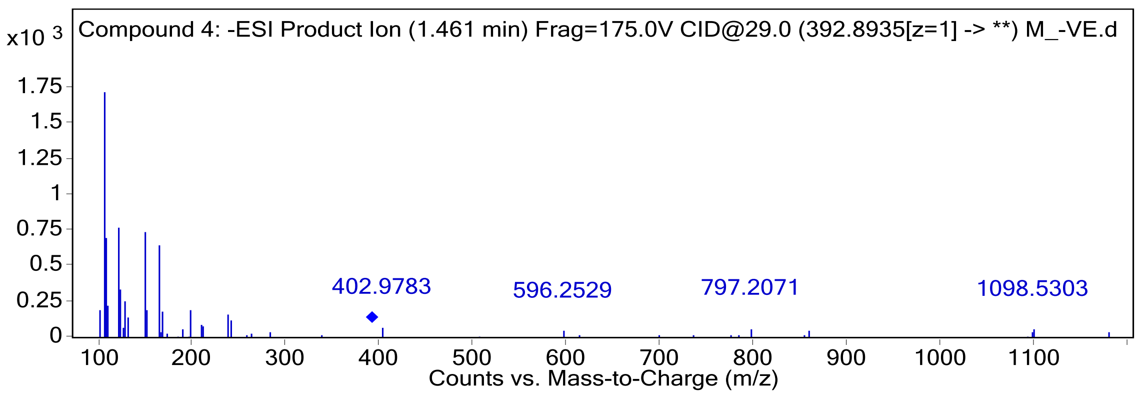

MS/MS Spectrum Peak List

m/z	z	Abund
104.9614	1	1720.61
106.9578	1	695.1
109.0294		224.12
120.9384		775.02
122.9368		339.62
126.992		256.04
148.9525	1	745.46
150.9405	1	195.08
164.9284		653.22
196.9652		194.79

Compound Label	*m/z*	RT	Algorithm
Compound 5	402.9229	1.495	Auto MS/MS

MS Spectrum

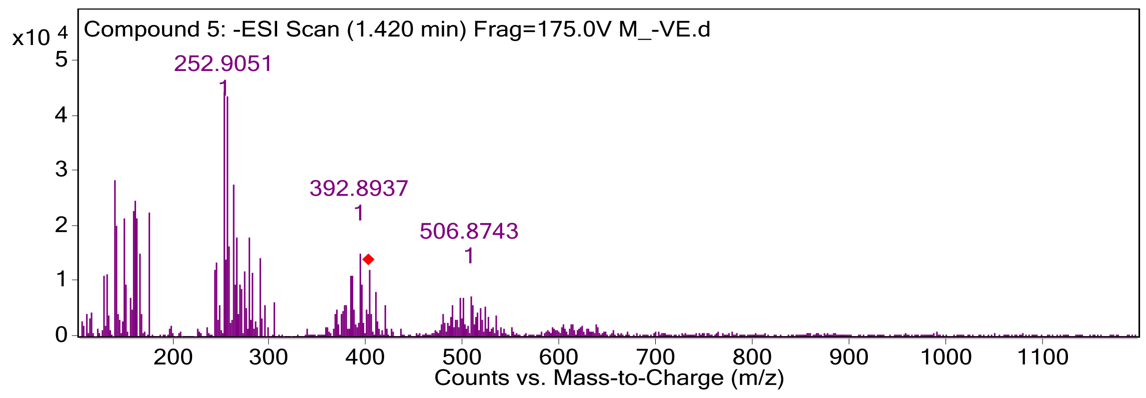

MS Zoomed Spectrum

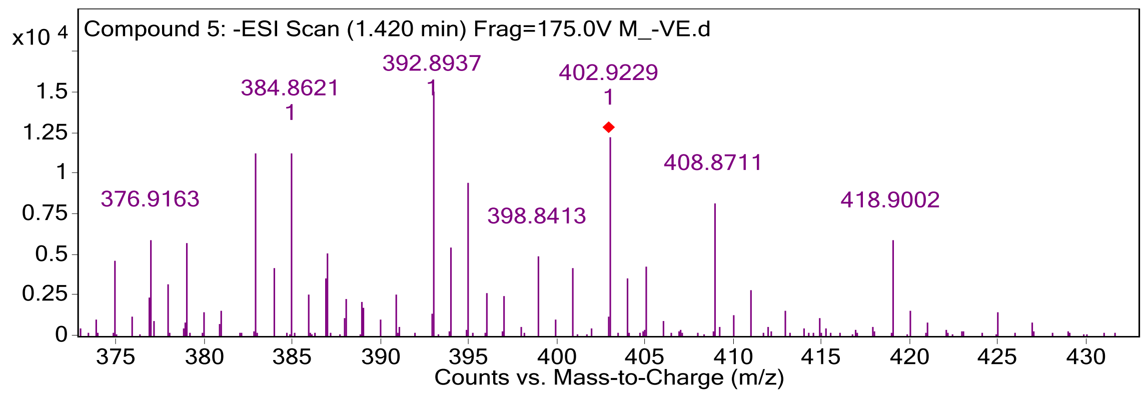

MS Spectrum Peak List

m/z	z	Abund
138.9234	1	28352.99
160.8449		24879.58
252.9051	1	44531.95
254.9023	1	43736.31
262.9343	1	27639.73
402.9229	1	12246.27
403.9237	1	3594.57
404.9217	1	4312.65
405.9229	1	1033.49
406.9244	1	209.28

MSMS Spectrum

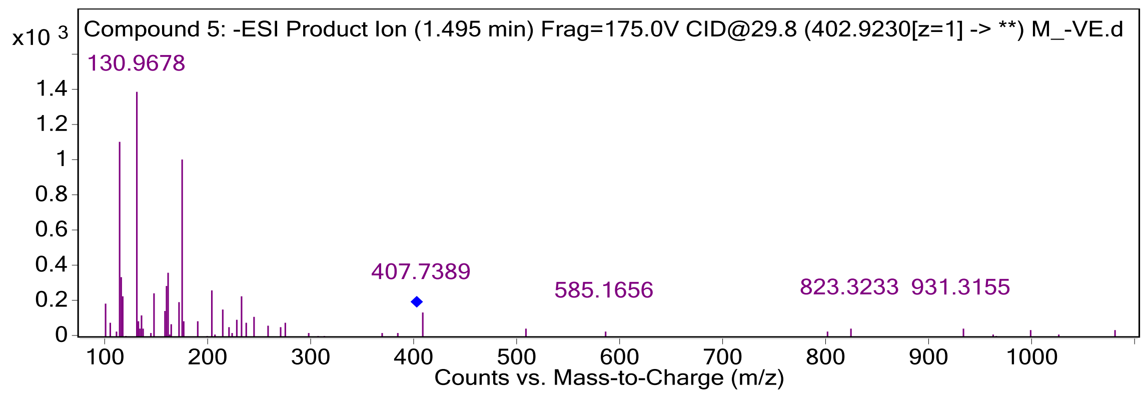

MS/MS Spectrum Peak List

m/z	z	Abund
114.9792		323.78
114.9909		1104.73
115.9921		340.48
116.9897		230.56
130.9678		1394.63
146.9628		248.64
159.983		294.03
160.981		363.83
174.9581	1	1011.91
203.9843		269.1

Compound Label	Name	*m/z*	RT	Algorithm	Mass
Cpd 6: Ribose-1-arsenate; C5 H11 As O8	**Ribose-1-arsenate**	272.9627	1.526	Auto MS/MS	273.9678

MS Spectrum

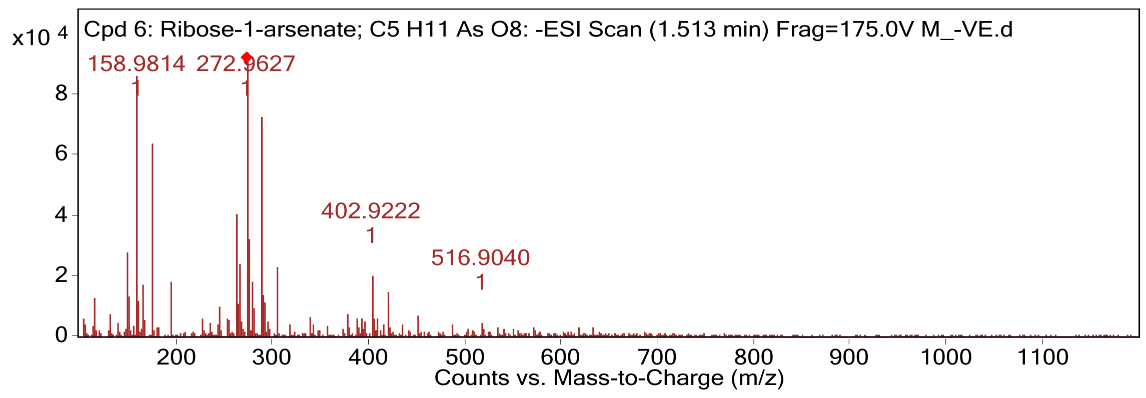

MS Zoomed Spectrum

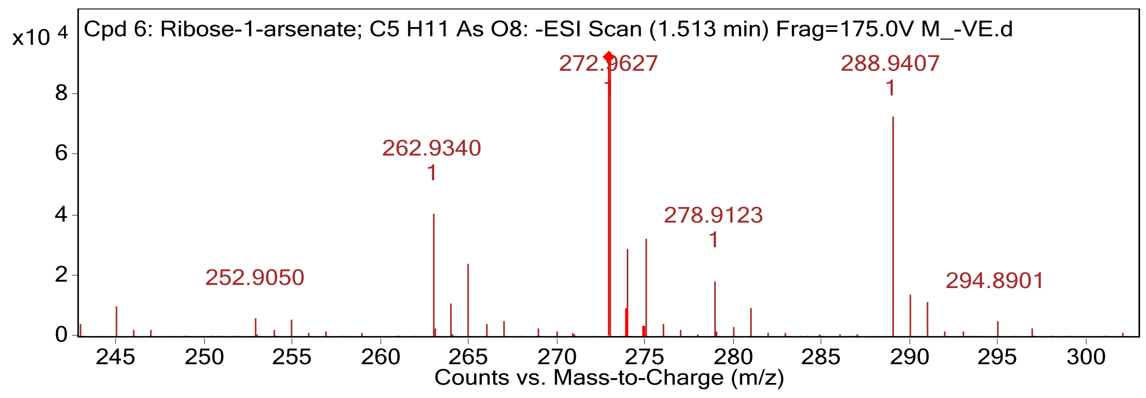

MS Spectrum Peak List

m/z	*Calc m/z*	Diff(ppm)	z	Abund	Formula	Ion
148.9523			1	28234.22		
158.9814			1	86237.42		
174.9589			1	64155.92		
262.934			1	40808.8		
272.9627	272.9597	-10.95	1	94104.98	C5 H11 As O8	(M-H)-
273.9637	273.9632	-1.85	1	29161.06	C5 H11 As O8	(M-H)-
274.961	274.9642	11.71	1	32530.18	C5 H11 As O8	(M-H)-

275.9628	275.9675	16.86	1	4353.73	C5 H11 As O8	(M-H)-
276.9553	276.9686	48.29	1	2616.45	C5 H11 As O8	(M-H)-
288.9407			1	72644.41		

MSMS Spectrum

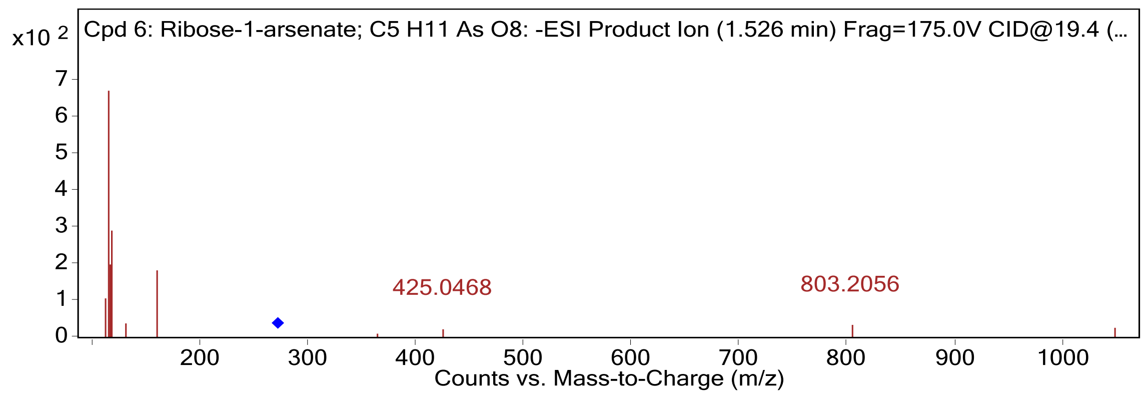

MS/MS Spectrum Peak List

m/z	z	Abund
111.9834		106.54
114.9734	2	131
114.9908		670.76
115.9913		200.32
116.9878		293.4
130.9659		41.42
158.95		38.08
158.9804		183.33
159.9795		80.8
803.2056		34.81

Compound Structure

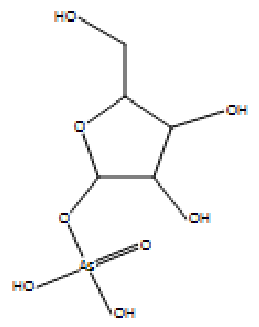

Compound Label	Name	*m/z*	RT	Algorithm	Mass
Cpd 7: 2,3,5,7,9-Pentathiadecane 2,2-dioxide; C5 H12 O2 S5	**2,3,5,7,9-Pentathiadecane 2,2-dioxide**	262.9344	1.534	Auto MS/MS	263.942

MS Spectrum

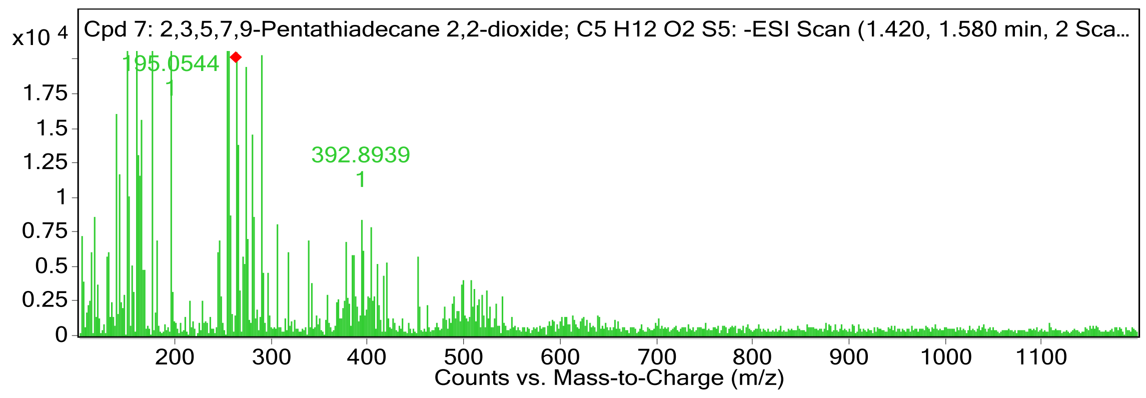

MS Zoomed Spectrum

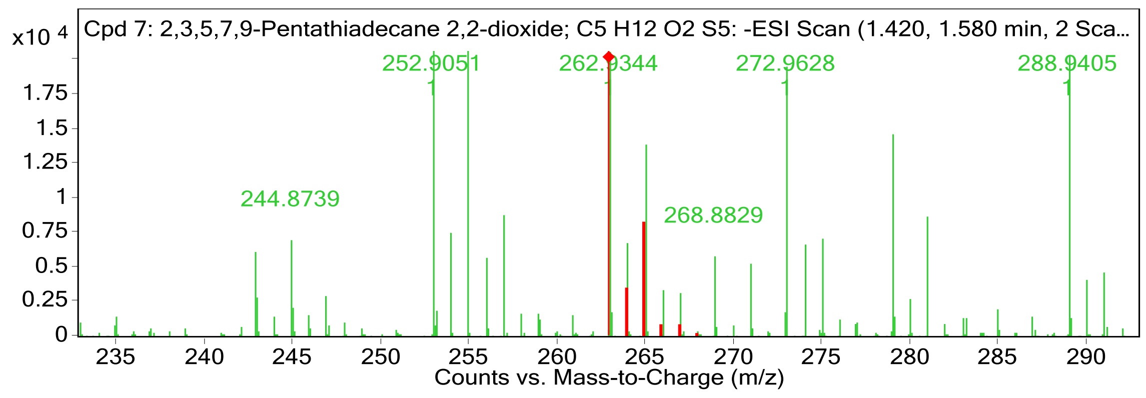

MS Spectrum Peak List

m/z	*Calc m/z*	Diff(ppm)	z	Abund	Formula	Ion
158.9815			1	36613.02		
174.959				31915.17		
195.0544			1	39217.92		
252.9051			1	24148.58		
262.9344	262.9368	9.31	1	20573.81	C5 H12 O2 S5	(M-H)-
263.9349	263.9386	13.86	1	6800.34	C5 H12 O2 S5	(M-H)-
264.9316	264.9329	4.69	1	13843.59	C5 H12 O2 S5	(M-H)-
265.9326	265.9348	8.37	1	3425.66	C5 H12 O2 S5	(M-H)-
266.9285	266.9291	2.47	1	3133.52	C5 H12 O2 S5	(M-H)-
267.9339	267.9312	-10.31	1	405.98	C5 H12 O2 S5	(M-H)-

MSMS Spectrum

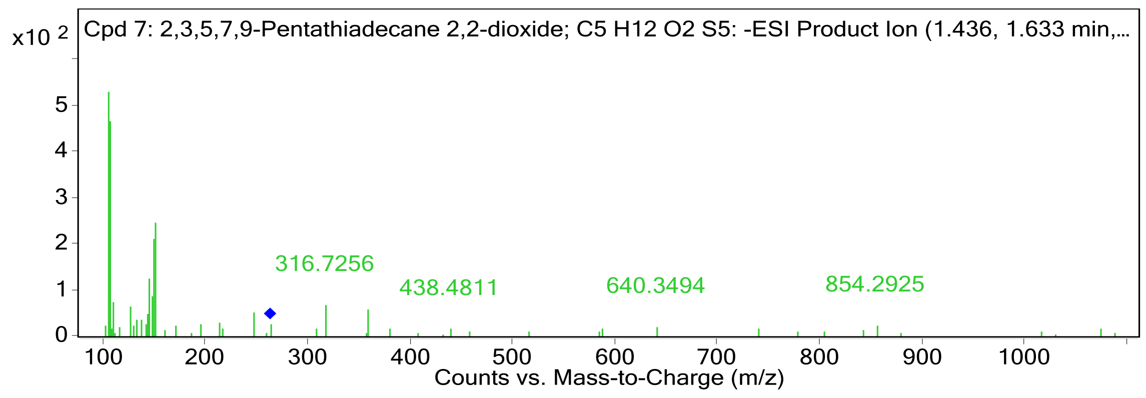

MS/MS Spectrum Peak List

m/z	z	Abund
104.9622		531.24
106.9573	1	468.18
108.9559		75.52

126.0311		65.52
144.9618		128.39
146.9434		90.32
148.9525		211.36
150.9488		248.25
316.7256		70.55
357.8628		60.74

Compound Structure

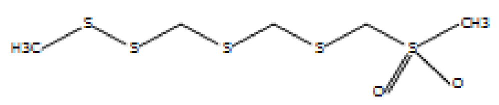

**Compound Label**	***m/z***	**RT**	**Algorithm**
Compound 8	288.9405	1.539	Auto MS/MS

MS Spectrum

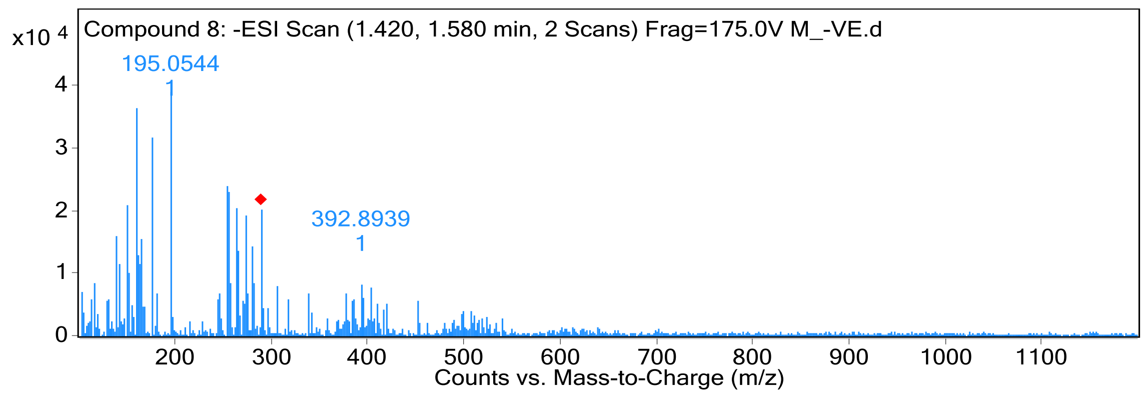

MS Zoomed Spectrum

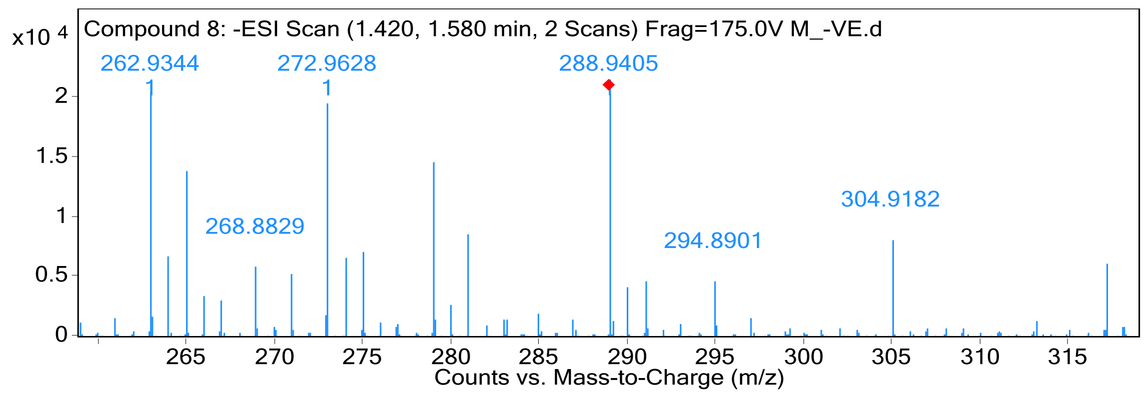

MS Spectrum Peak List

m/z	z	Abund
148.9524	1	21036.75
158.9815	1	36613.02
174.959		31915.17
195.0544	1	39217.92
252.9051	1	24148.58
254.9022	1	23242.72
288.9405	1	20330.36
289.9438	1	4120.27
290.9395	1	4620.32
291.9451	1	628.39

MSMS Spectrum

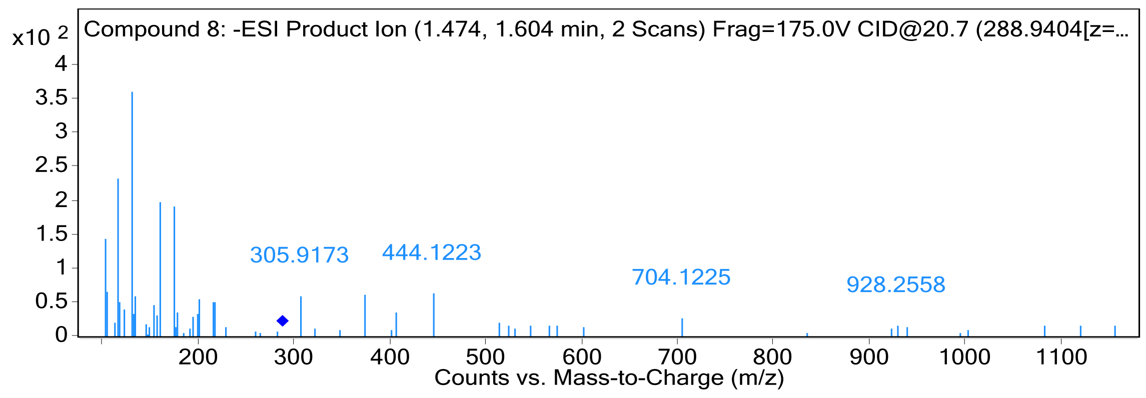

MS/MS Spectrum Peak List

m/z	z	Abund
102.9734		144.33
103.9464		68.1
114.9736		82.86
114.9905		232.46
115.9774		74.11
115.9899		163.04
130.9678		361.82
158.9819		197.89
174.9467		74.43
174.9588	1	193.44

**Compound Label**	**Name**	***m/z***	**RT**	**Algorithm**	**Mass**
Cpd 9: (-)-epicatechin-3'-O-glucuronide; C21 H24 O11	**(-)-epicatechin-3'-O-glucuronide**	451.128	1.733	Auto MS/MS	452.1332

MS Spectrum

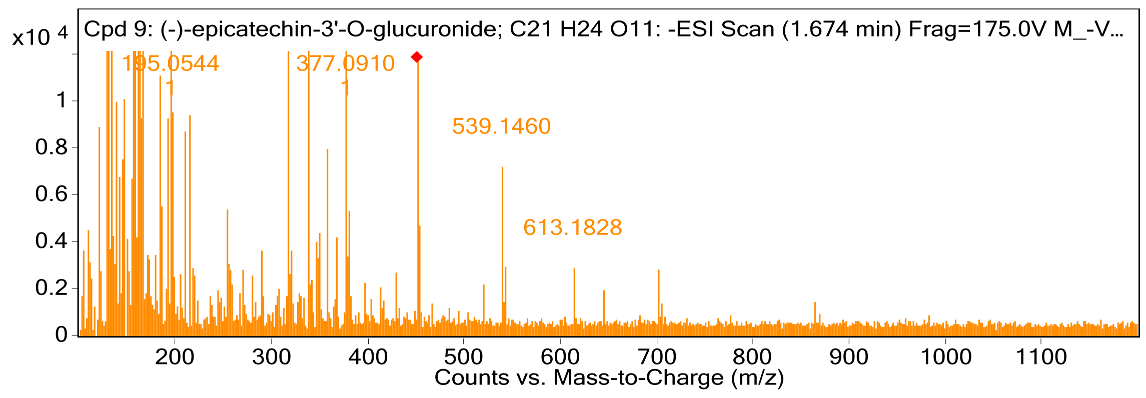

MS Zoomed Spectrum

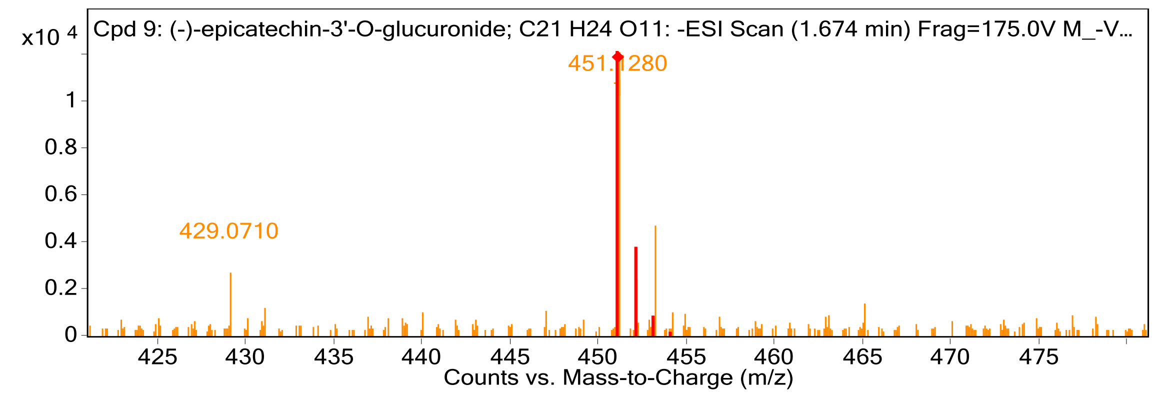

MS Spectrum Peak List

m/z	*Calc m/z*	Diff(ppm)	z	Abund	Formula	Ion
130.8915			1	21904.1		
134.0495				31859.6		
160.8446				30306.67		
162.842				32247.87		
195.0544			1	131420.23		
451.128	451.1246	-7.63	1	12114.71	C21 H24 O11	(M-H)-
452.1326	452.128	-10.12	1	2476.76	C21 H24 O11	(M-H)-
453.1268	453.1302	7.56	1	4734.66	C21 H24 O11	(M-H)-
454.1286	454.1329	9.47	1	1045.4	C21 H24 O11	(M-H)-
455.1268	455.1353	18.49	1	424.99	C21 H24 O11	(M-H)-

MSMS Spectrum

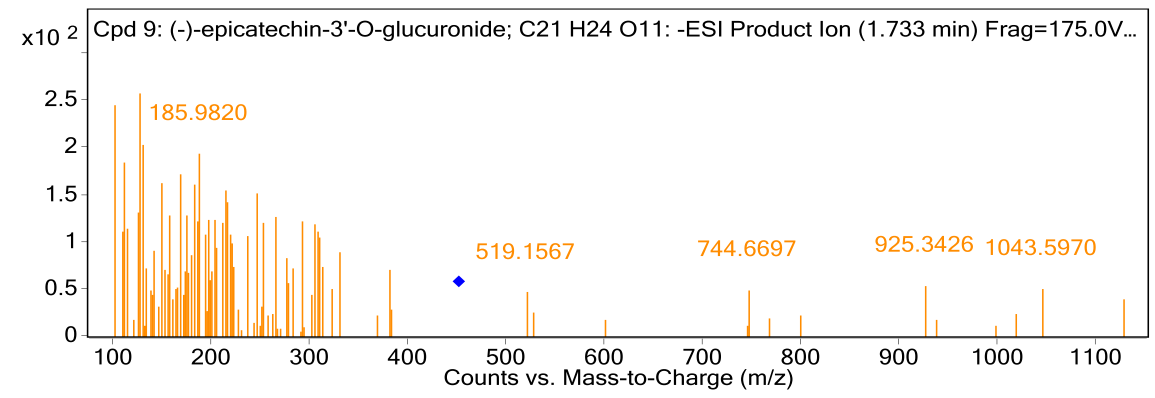

MS/MS Spectrum Peak List

m/z	Abund
101.0237	245.34
110.9024	185.54
126.9898	258.58
128.8875	203.56
148.8658	163.28
166.9777	173.03
180.9998	162.1
185.982	194.93
213	154.8
244.8004	152

Compound Structure

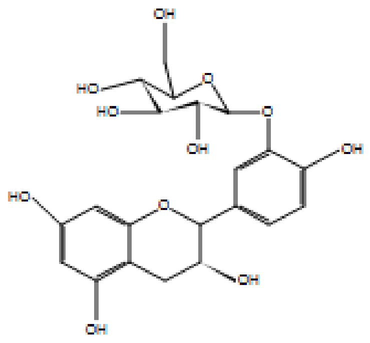

Compound Label	Name	*m/z*	RT	Algorithm	Mass
Cpd 10: Tosyllysine Chloromethyl Ketone; C14 H21 Cl N2 O3 S	**Tosyllysine Chloromethyl Ketone**	377.0907	1.789	Auto MS/MS	332.0935

MS Spectrum

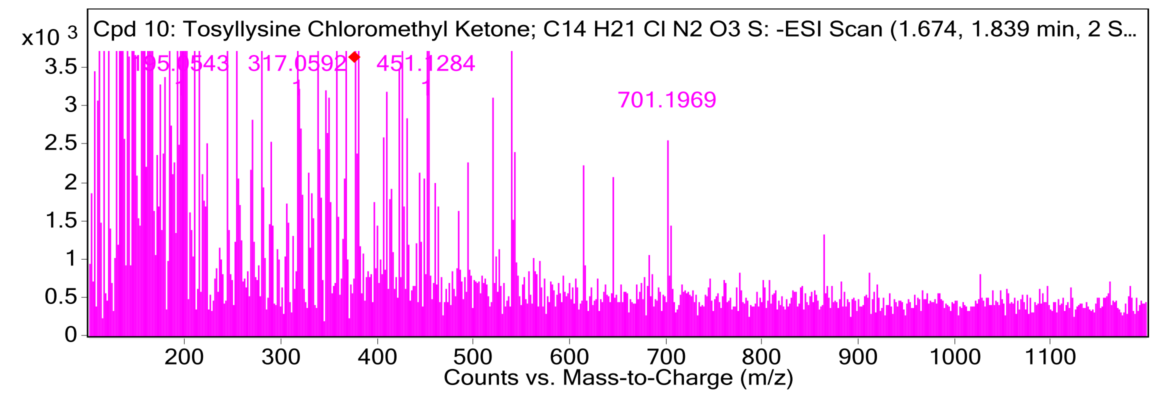

MS Zoomed Spectrum

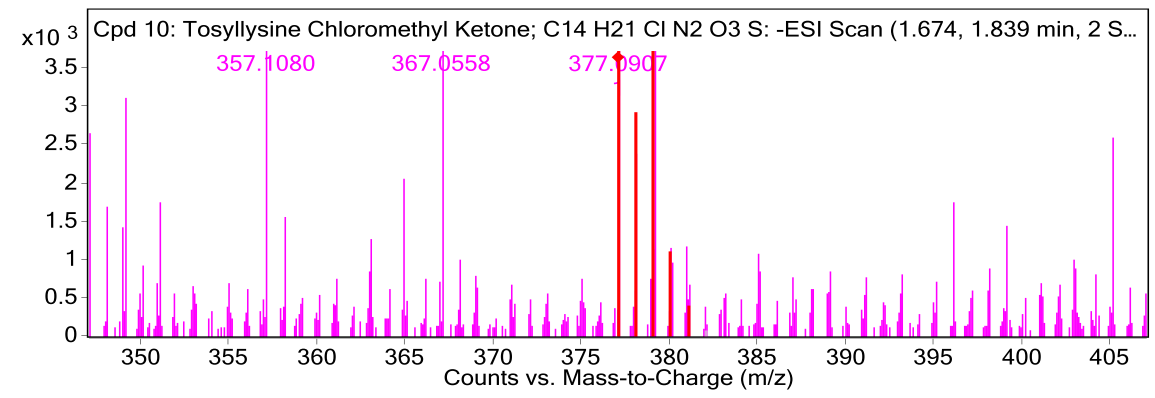

MS Spectrum Peak List

m/z	*Calc m/z*	Diff(ppm)	z	Abund	Formula	Ion
160.8446				29663.94		
162.842				28865		
191.0229			1	104287.8		
195.0543			1	177275.09		
197.8112				35444.09		
377.0907	377.0943	9.54	1	15961.59	C14 H21 Cl N2 O3 S	(M+HCOO)-
378.0946	378.0973	7.14	1	2394.64	C14 H21 Cl N2 O3 S	(M+HCOO)-

379.0904	379.0918	3.7	1	4815.63	C14 H21 Cl N2 O3 S	(M+HCOO)-
380.0912	380.0946	8.93	1	970.11	C14 H21 Cl N2 O3 S	(M+HCOO)-
381.1024	381.091	-29.94	1	683.33	C14 H21 Cl N2 O3 S	(M+HCOO)-

MSMS Spectrum

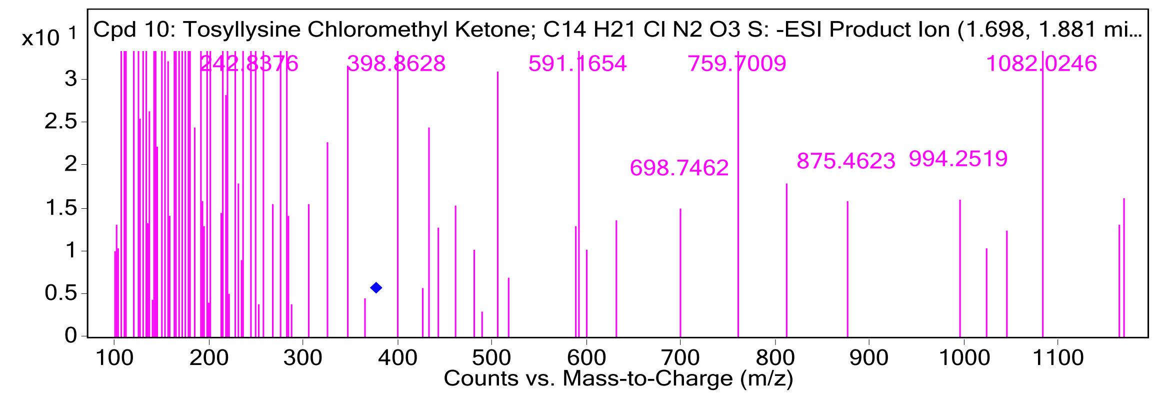

MS/MS Spectrum Peak List

m/z	Abund
110.9076	130.85
124.0377	79.4
132.9402	93.36
132.9919	98.27
142.0538	102.5
148.9332	77.88
163.0503	77.11
190.9443	80.22
196.8751	76.9
242.8376	90.5

Compound Structure

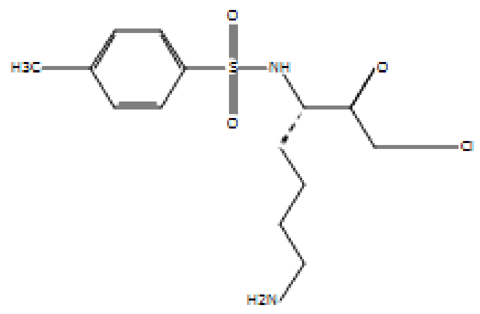

Compound Label	Name	*m/z*	RT	Algorithm	Mass
Cpd 11: 5-Dehydro-4-deoxy-D-glucarate; C6 H8 O7	**5-Dehydro-4-deoxy-D-glucarate**	191.0231	1.843	Auto MS/MS	192.0304

MS Spectrum

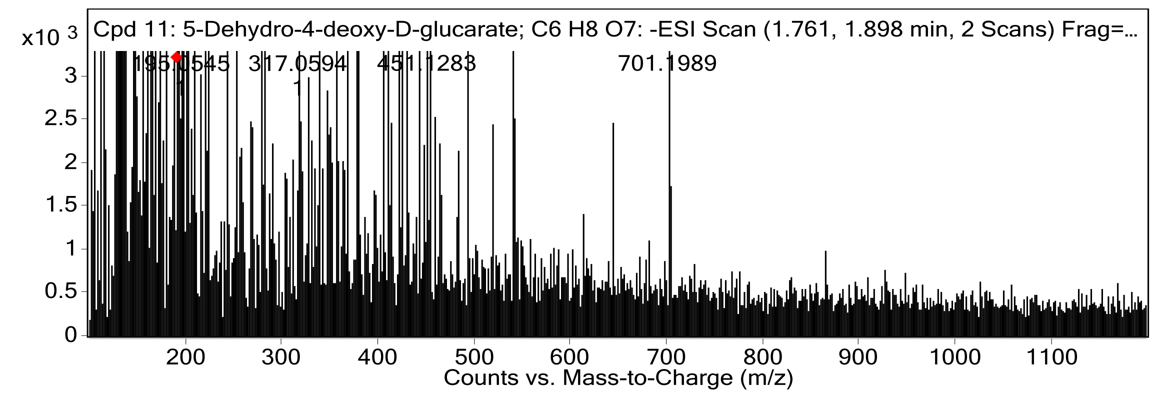

MS Zoomed Spectrum

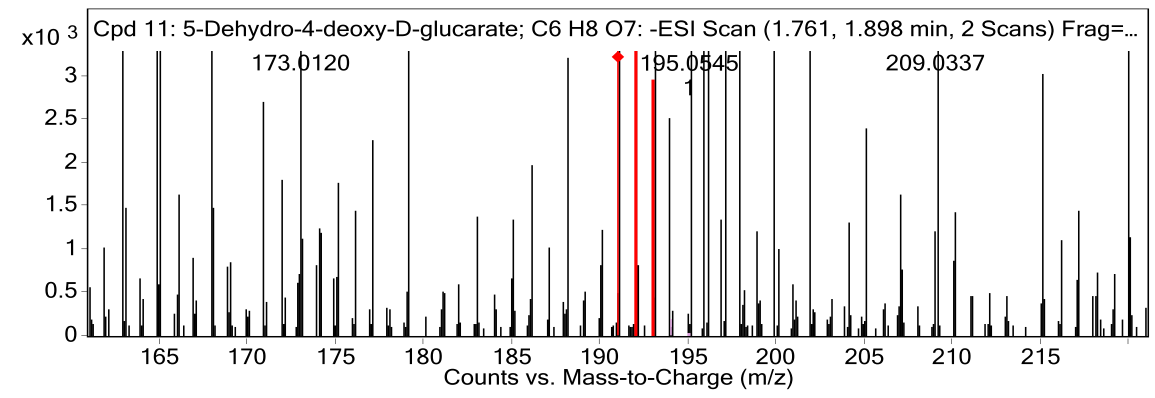

MS Spectrum Peak List

m/z	*Calc m/z*	Diff(ppm)	z	Abund	Formula	Ion
111.0109				35530.44		
133.0169				26970.5		
160.8449				37584.14		
162.8419				37631.22		
191.0231	191.0197	-17.8	1	178493.91	C6 H8 O7	(M-H)-
192.0265	192.0231	-17.29	1	12598.54	C6 H8 O7	(M-H)-
193.0287	193.0243	-23.1	1	4147.09	C6 H8 O7	(M-H)-
195.0545			1	192114.97		
195.8143				42255.59		
197.8114				48753.3		

MSMS Spectrum

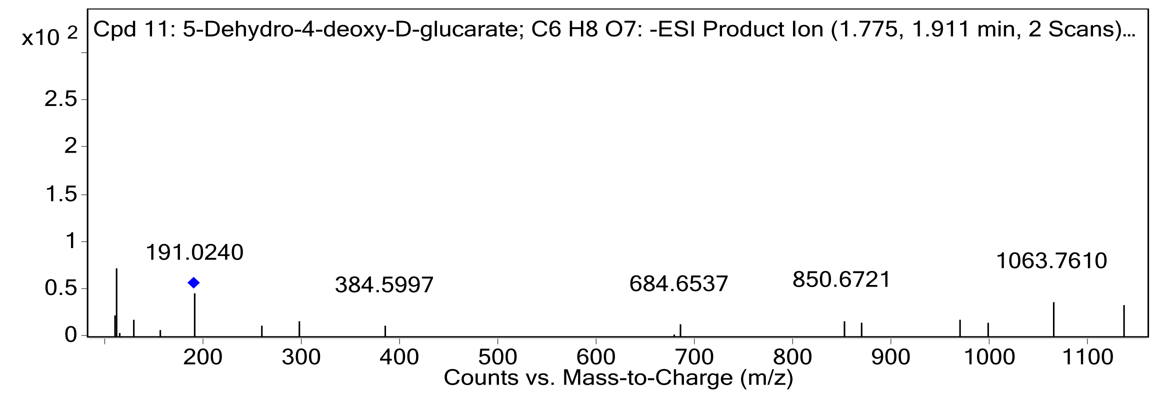

MS/MS Spectrum Peak List

m/z	Abund
109.689	22.99
111.0097	72.32
129.0206	19.4

191.024	46.49
296.7608	16.64
850.6721	17.76
968.0328	18.29
997.7244	15.69
1063.761	37.58
1135.29	34.88

Compound Structure

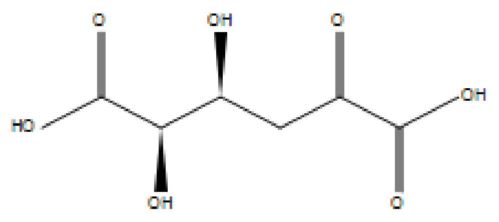

Compound Label	Name	*m/z*	RT	Algorithm	Mass
Cpd 12: 3,7-Dimethyluric acid; C7 H8 N4 O3	**3,7-Dimethyluric acid**	195.0543	1.92	Auto MS/MS	196.0617

MS Spectrum

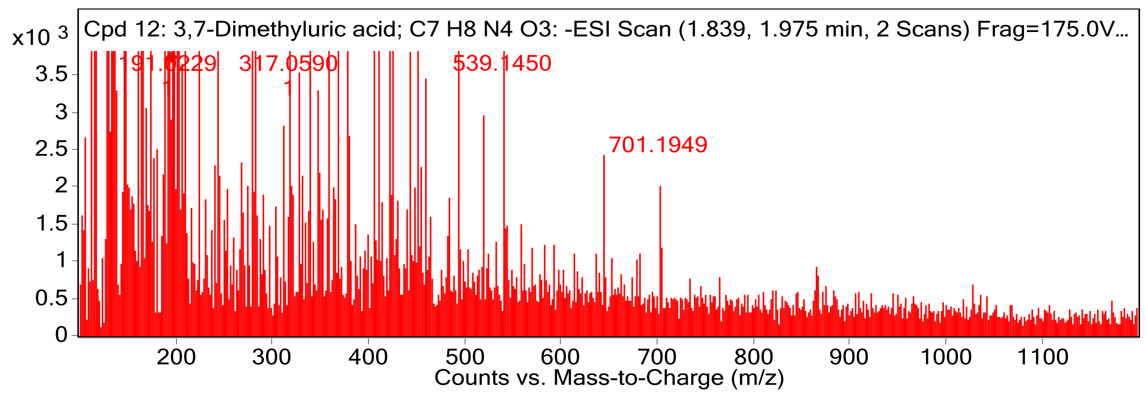

MS Zoomed Spectrum

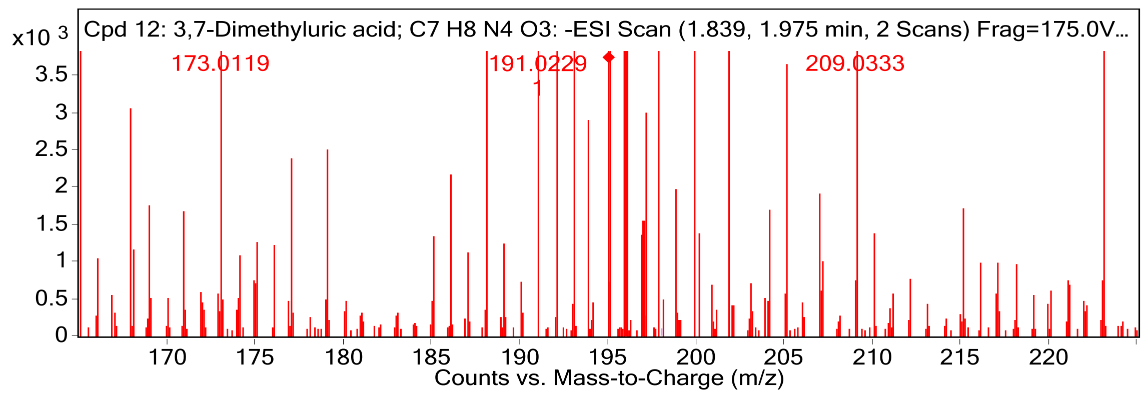

MS Spectrum Peak List

m/z	*Calc m/z*	Diff(ppm)	z	Abund	Formula	Ion
111.0109			1	47077.43		
133.0167			1	26168.03		
191.0229			1	241768.34		
195.0543	195.0524	-9.92	1	153630.13	C7 H8 N4 O3	(M-H)-
195.8141				50938.63		
196.0582	196.0548	-17.45	1	10227.12	C7 H8 N4 O3	(M-H)-
197.0586	197.0568	-9.42	1	3018.51	C7 H8 N4 O3	(M-H)-
197.8112			1	64256.45		
199.8081			1	29641.21		
317.059			1	25418.35		

MSMS Spectrum

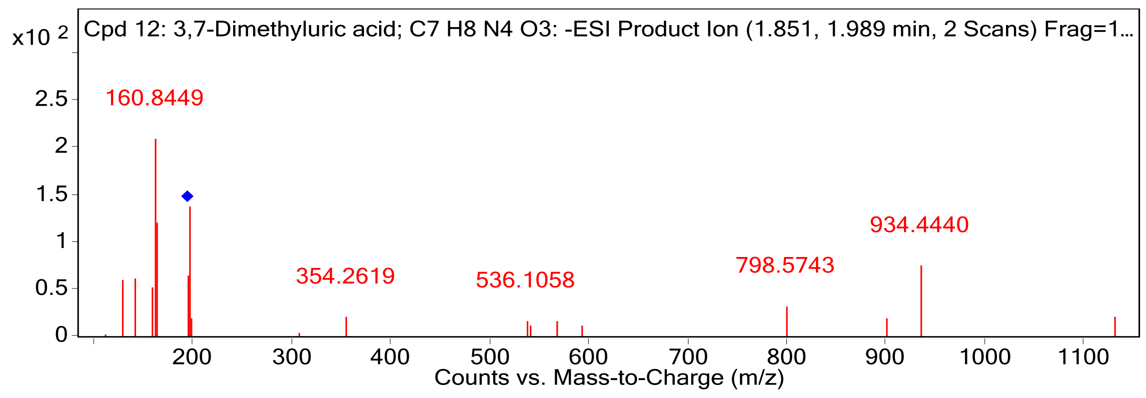

MS/MS Spectrum Peak List

m/z	Abund
129.0196	61.06
141.0234	61.46
158.8494	52.38
160.8319	32.98
160.8449	210.35
162.8416	120.68
195.0503	65.68
195.8109	138.65
196.8146	69.66
934.444	75.72

Compound Structure

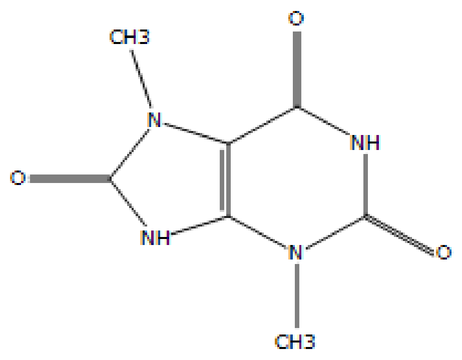

Compound Label	Name	*m/z*	RT	Algorithm	Mass
Cpd 13: GYKI 52466; C17 H15 N3 O2	**GYKI 52466**	338.1139	1.94	Auto MS/MS	293.1155

MS Spectrum

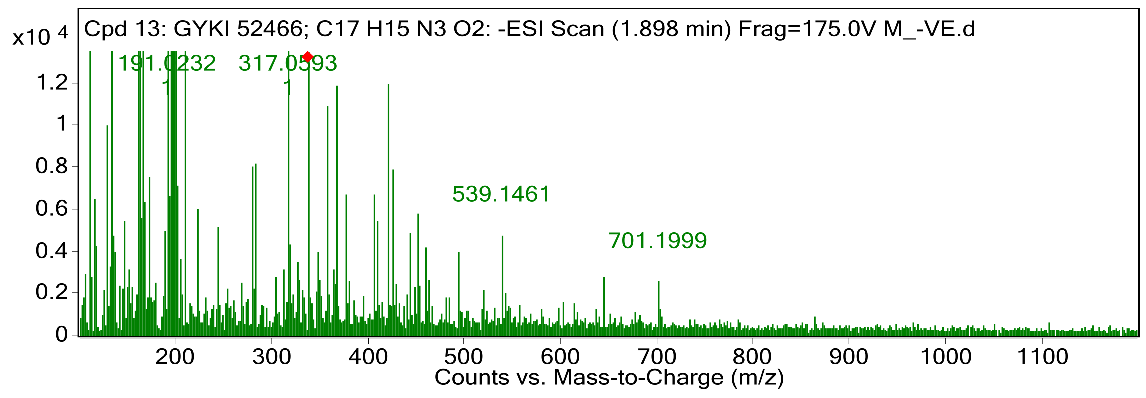

MS Zoomed Spectrum

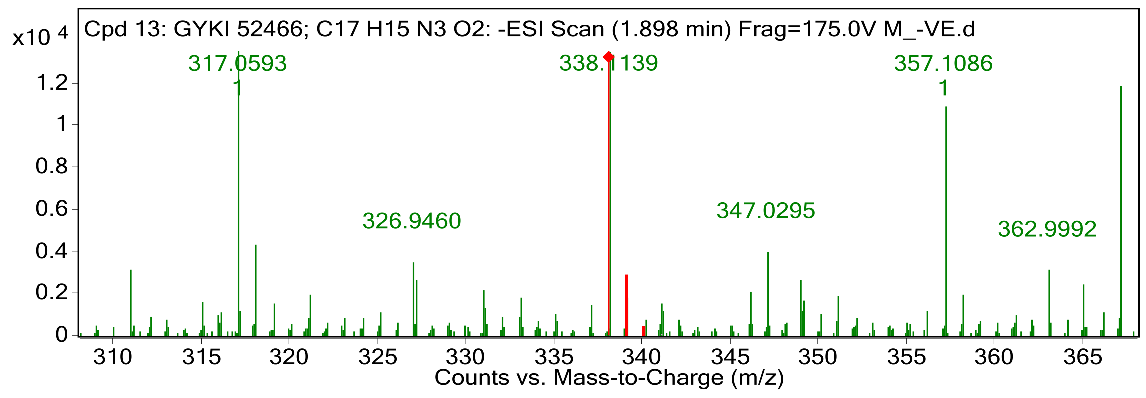

MS Spectrum Peak List

m/z	*Calc m/z*	Diff(ppm)	z	Abund	Formula	Ion
111.0109			1	57600.65		
133.017				28906.47		
191.0232			1	288738.66		
195.0543			1	153612.11		
195.8143			1	58961.74		
197.8114			1	67424.88		
199.8079				30559.71		
338.1139	338.1146	2.24		13502.18	C17 H15 N3 O2	(M+HCOO)-
339.1157	339.1177	5.74	1	1865.04	C17 H15 N3 O2	(M+HCOO)-
340.1194	340.1202	2.47	1	810.52	C17 H15 N3 O2	(M+HCOO)-

MSMS Spectrum

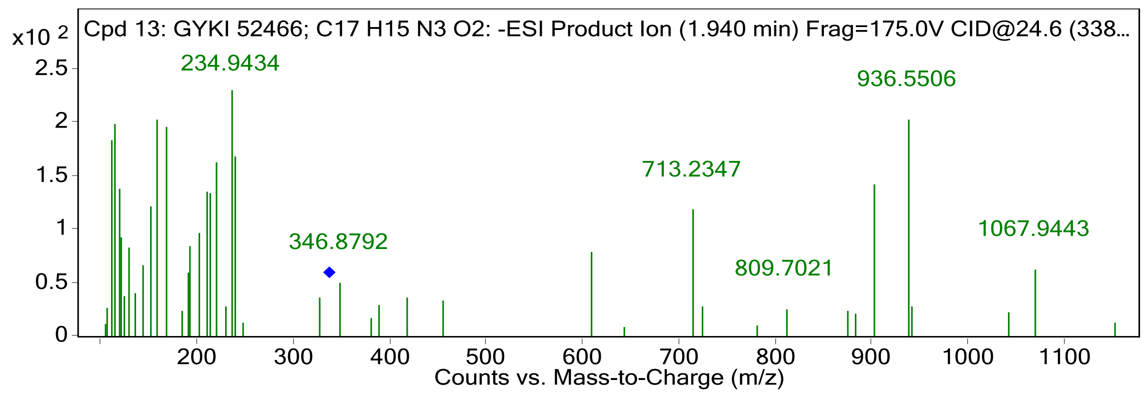

MS/MS Spectrum Peak List

m/z	Abund
111.0242	184
114.9296	198.37
119.0367	138.94
158.0476	202.35
167.086	196.56
218.9106	162.61
234.9434	230.34
238.0216	169.04
901.2922	143.09
936.5506	203.27

Compound Structure

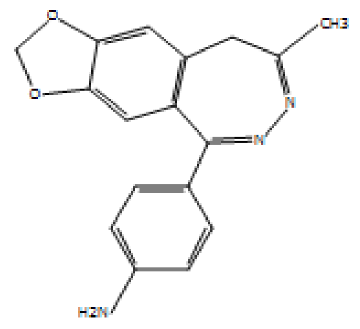

Compound Label	Name	*m/z*	RT	Algorithm	Mass
Cpd 14: 3,7-Dimethyluric acid; C7 H8 N4 O3	**3,7-Dimethyluric acid**	195.0541	2.135	Auto MS/MS	196.0614

MS Spectrum

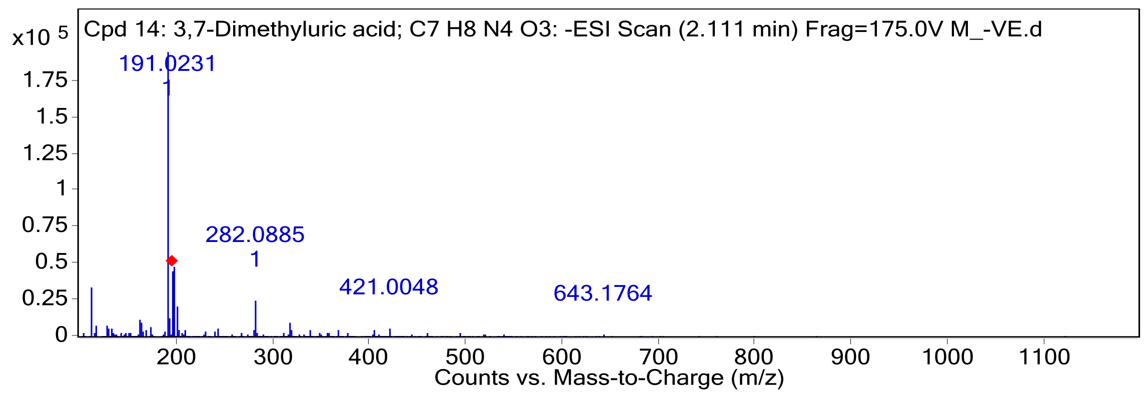

MS Zoomed Spectrum

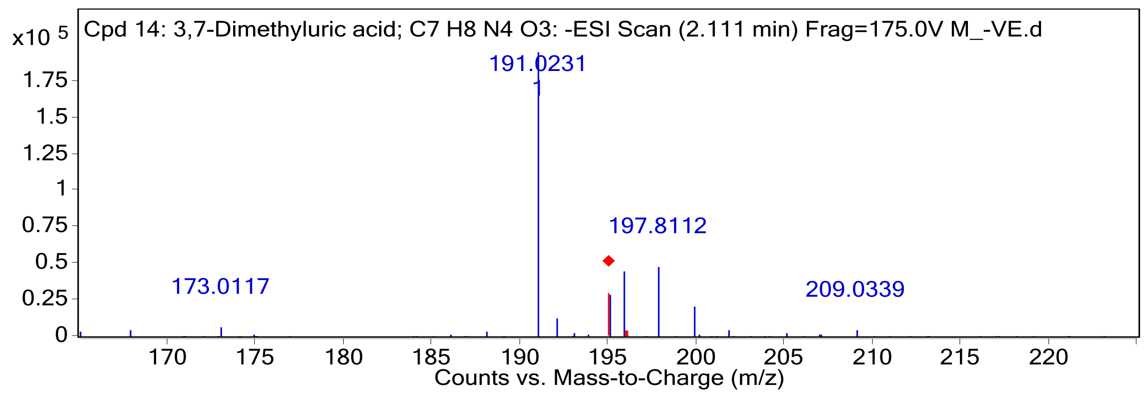

MS Spectrum Peak List

m/z	*Calc m/z*	Diff(ppm)	z	Abund	Formula	Ion
111.0107			1	34047.82		
191.0231			1	194865.56		
192.0259			1	13305.98		
195.0541	195.0524	-8.68	1	29107.85	C7 H8 N4 O3	(M-H)-
195.8143				45282.57		
196.0583	196.0548	-18.27	1	1739.22	C7 H8 N4 O3	(M-H)-
197.0555	197.0568	6.59	1	768.5	C7 H8 N4 O3	(M-H)-
197.8112				48322.53		
199.8081				21315.21		
282.0885			1	25322.46		

MSMS Spectrum

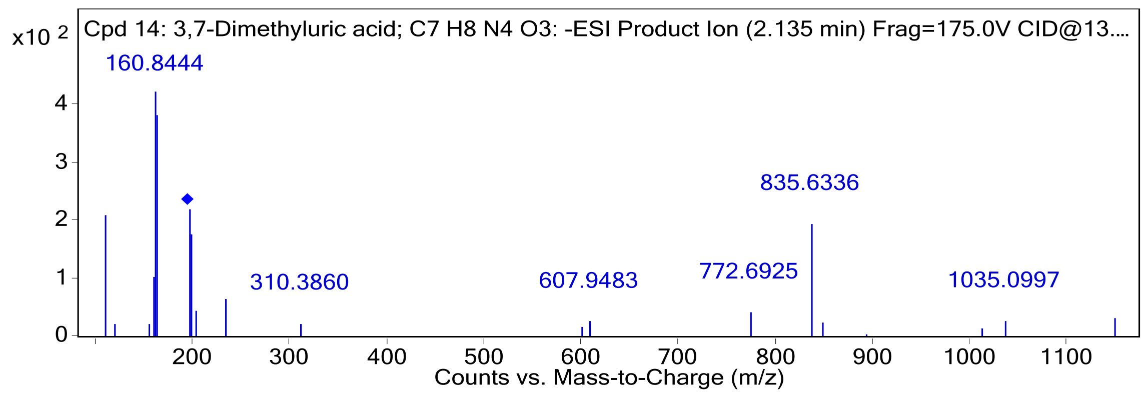

MS/MS Spectrum Peak List

m/z	z	Abund
109.99		210.02
158.8491		104
160.8444		425.16
162.8398	2	382.81
195.8103		219.81
197.8088		176.84
202.6556		44.58
233.8903		65.08
772.6925		43.16
835.6336		196.54

Compound Structure

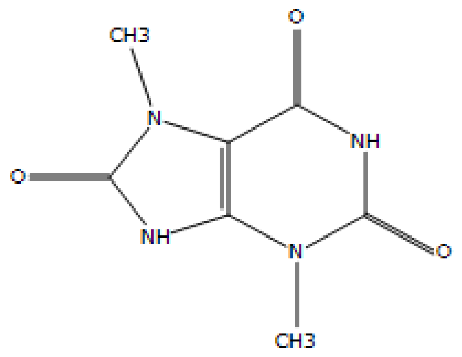

Compound Label	Name	*m/z*	RT	Algorithm	Mass
Cpd 15: Ketamine; C13 H16 Cl N O	**Ketamine**	282.0886	2.378	Auto MS/MS	237.0905

MS Spectrum

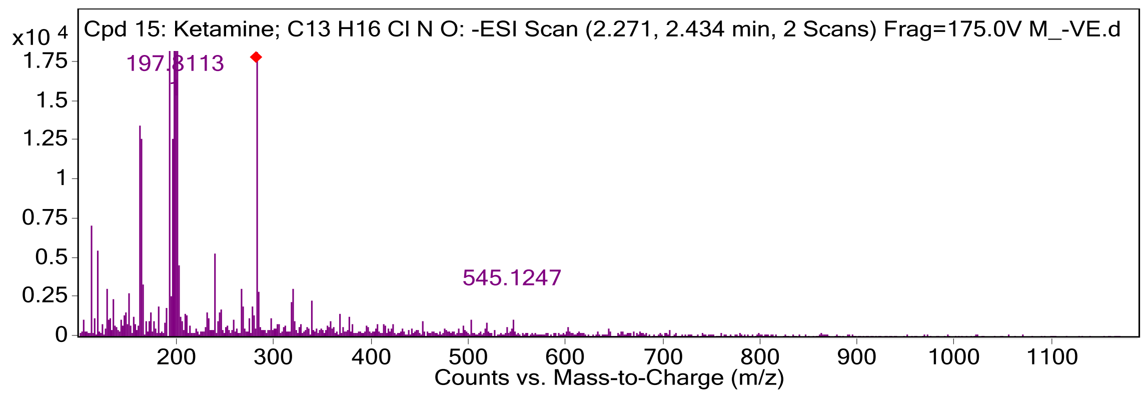

MS Zoomed Spectrum

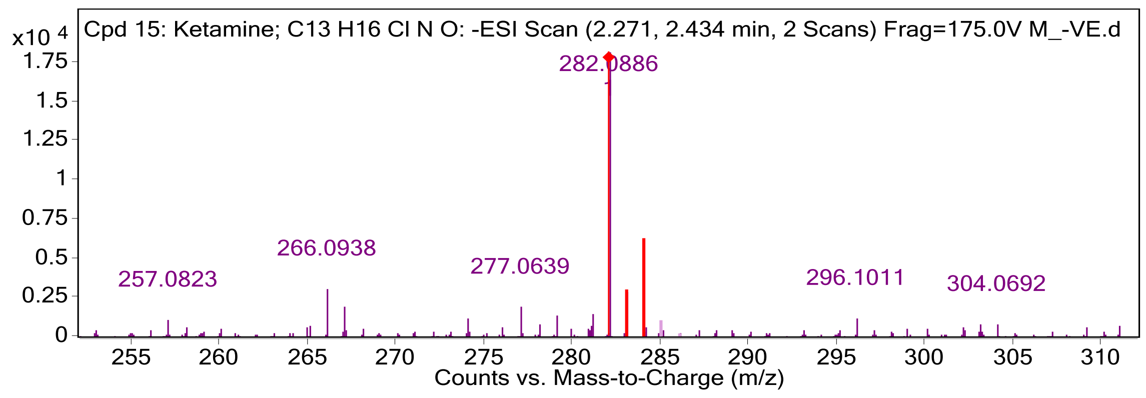

MS Spectrum Peak List

m/z	*Calc m/z*	Diff(ppm)	z	Abund	Formula	Ion
160.8445				13495.47		
162.8418				12598.92		
191.0231			1	33515.28		
195.0542				12656.4		
195.8142			1	41069.52		
197.8113			1	49318.73		
199.8082			1	21685.95		

282.0886	282.0902	5.92	1	18152.67	C13 H16 Cl N O	(M+HCOO)-
283.092	283.0935	5.37	1	2873.91	C13 H16 Cl N O	(M+HCOO)-
284.0883	284.0878	-2.06	1	659.81	C13 H16 Cl N O	(M+HCOO)-

MSMS Spectrum

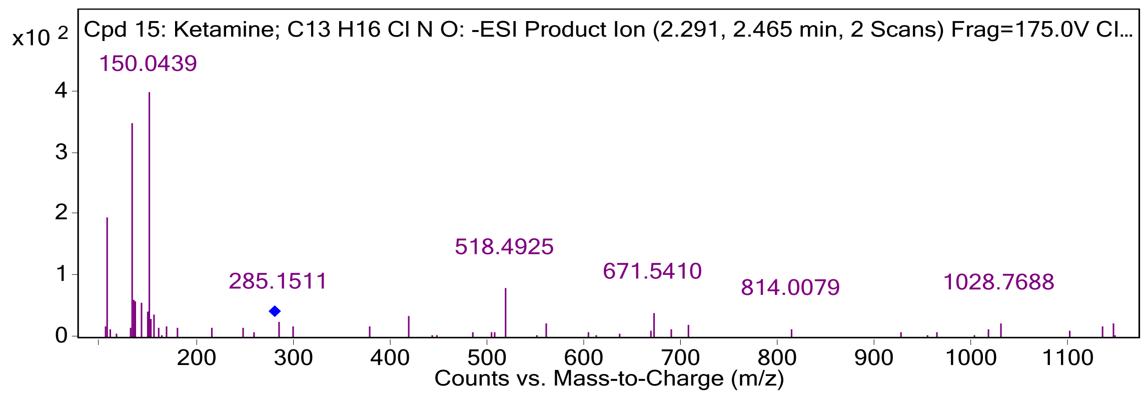

MS/MS Spectrum Peak List

m/z	Abund
108.0126	141.22
108.0222	195.64
133.0182	350.28
135.0253	63.02
136.0344	60.52
143.039	57.63
150.0226	44
150.0439	400.51
518.4925	81.62
671.541	41.75

Compound Structure

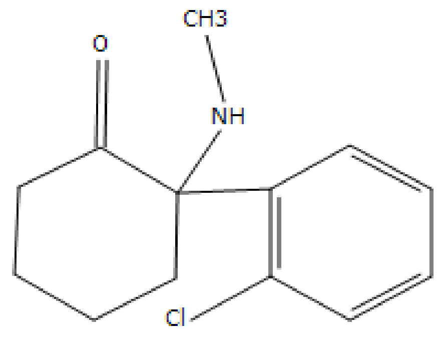

Compound Label	Name	*m/z*	RT	Algorithm	Mass
Cpd 16: Aprepitant; C23 H21 F7 N4 O3	**Aprepitant**	593.1594	6.011	Auto MS/MS	534.1456

MS Spectrum

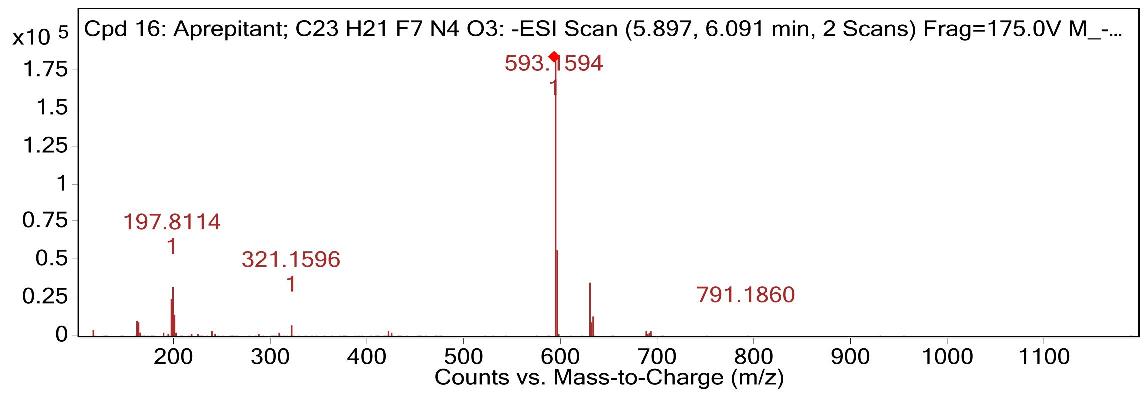

MS Zoomed Spectrum

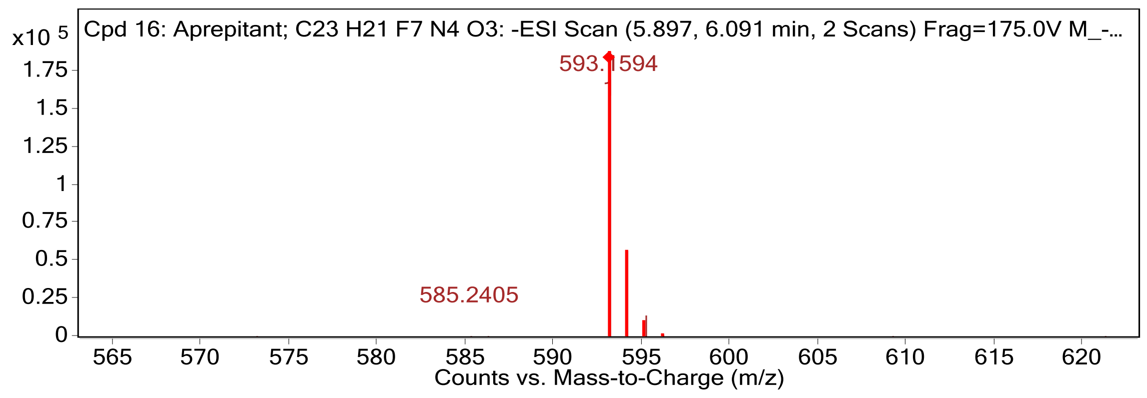

MS Spectrum Peak List

m/z	*Calc m/z*	Diff(ppm)	z	Abund	Formula	Ion
160.8448				10269.4		
195.8143			1	25178.86		
197.8114			1	32503.51		
199.8081			1	14950.45		
593.1594	593.164	7.76	1	187515.98	C23 H21 F7 N4 O3	(M+CH3COO)-
594.1626	594.1671	7.64	1	57169.07	C23 H21 F7 N4 O3	(M+CH3COO)-
595.1653	595.1698	7.57	1	14568.71	C23 H21 F7 N4 O3	(M+CH3COO)-
596.1691	596.1724	5.44	1	2160.55	C23 H21 F7 N4 O3	(M+CH3COO)-
629.136			1	35282.97		
631.1361			1	13856.98		

MSMS Spectrum

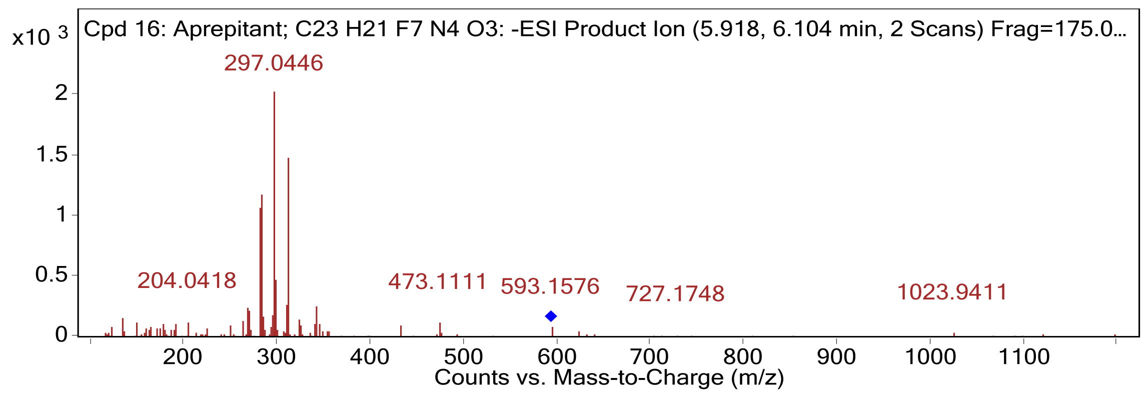

MS/MS Spectrum Peak List

m/z	z	Abund
268.045		242.47
281.0508		244.55
282.0581		1072.23
283.0643	1	1176.53

297.0446	1	2031.23
298.0478	1	471.6
310.0506		264.42
311.0603	1	1486.52
312.0641	1	525.89
341.0731	1	257.76

Compound Structure

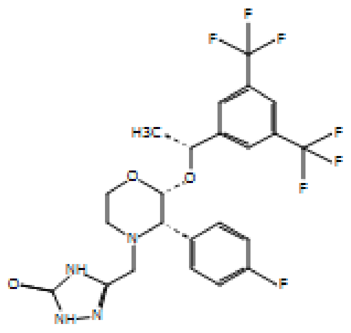

Compound Label	Name	*m/z*	RT	Algorithm	Mass
Cpd 17: CMP-N-glycoloylneuraminate; C20 H31 N4 O17 P	**CMP-N-glycoloylneuraminate**	629.1361	6.062	Auto MS/MS	630.1417

MS Spectrum

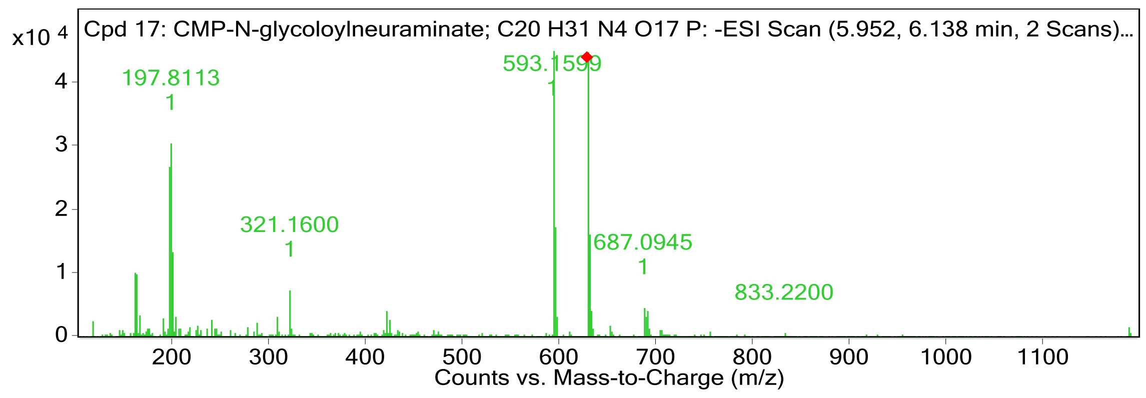

MS Zoomed Spectrum

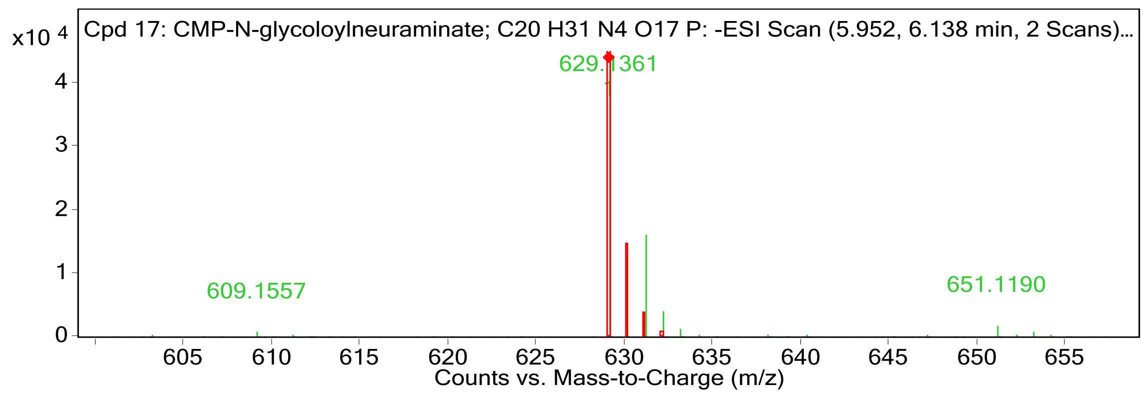

MS Spectrum Peak List

m/z	*Calc m/z*	Diff(ppm)	z	Abund	Formula	Ion
195.8144			1	26823.03		
197.8113			1	30606.71		
593.1599			1	239224.86		
594.163			1	69386.24		
595.165			1	17331.76		
629.1361	629.1349	-1.96	1	44913.45	C20 H31 N4 O17 P	(M-H)-
630.1397	630.1379	-2.82	1	12920.46	C20 H31 N4 O17 P	(M-H)-
631.1348	631.14	8.21	1	16097.97	C20 H31 N4 O17 P	(M-H)-
632.1372	632.1425	8.43	1	4279.12	C20 H31 N4 O17 P	(M-H)-
633.1395	633.1447	8.26	1	1340.47	C20 H31 N4 O17 P	(M-H)-

MSMS Spectrum

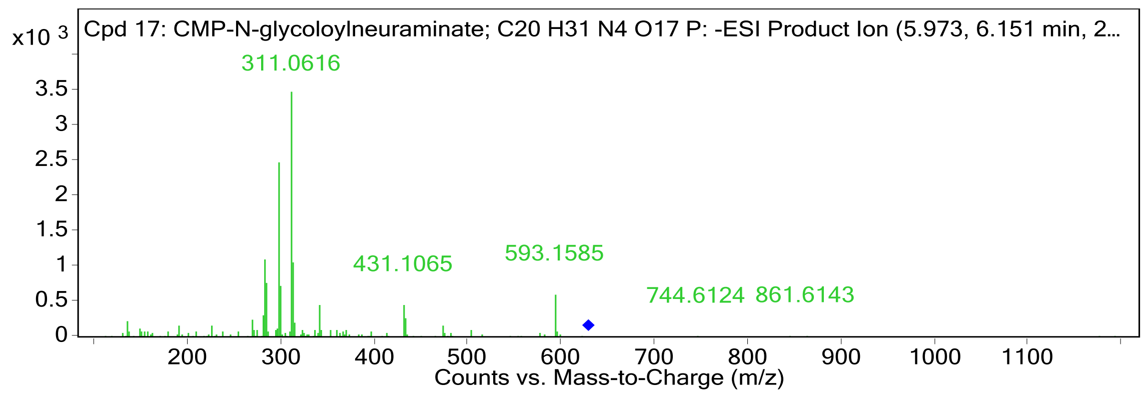

MS/MS Spectrum Peak List

m/z	z	Abund
281.0483		307.05
282.0578		1112.64
283.065	1	770.61
297.0447	1	2483.48
298.0498	1	733.37
311.0616	1	3491.05
312.0639	1	1054.92
341.0715		451.69
431.1065		460.52
593.1585	1	612.62

Compound Structure

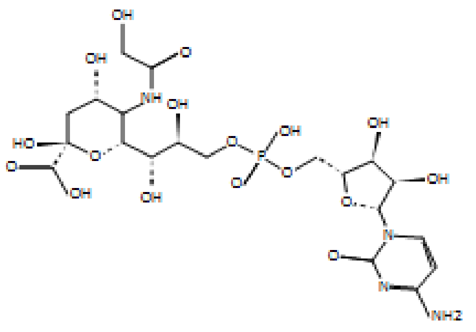

Compound Label	Name	*m/z*	RT	Algorithm	Mass
Cpd 18: Aprepitant; C23 H21 F7 N4 O3	**Aprepitant**	593.1595	6.391	Auto MS/MS	534.1456

MS Spectrum

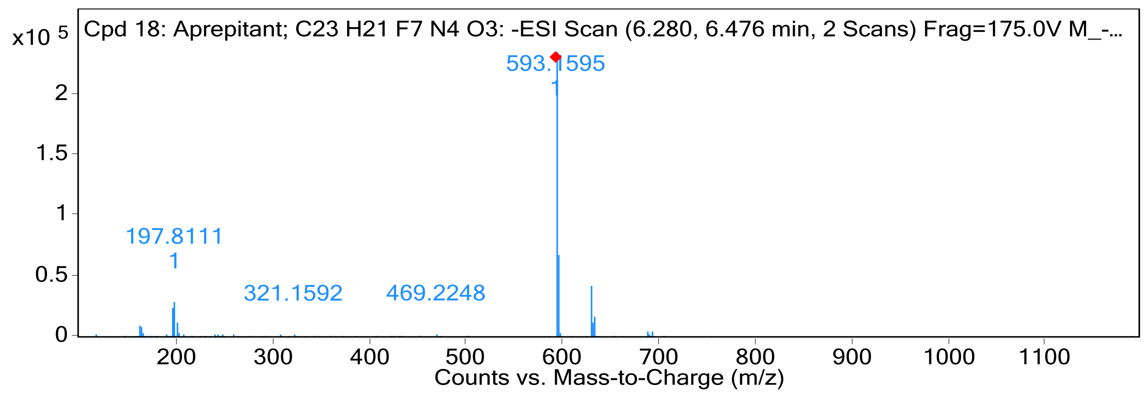

MS Zoomed Spectrum

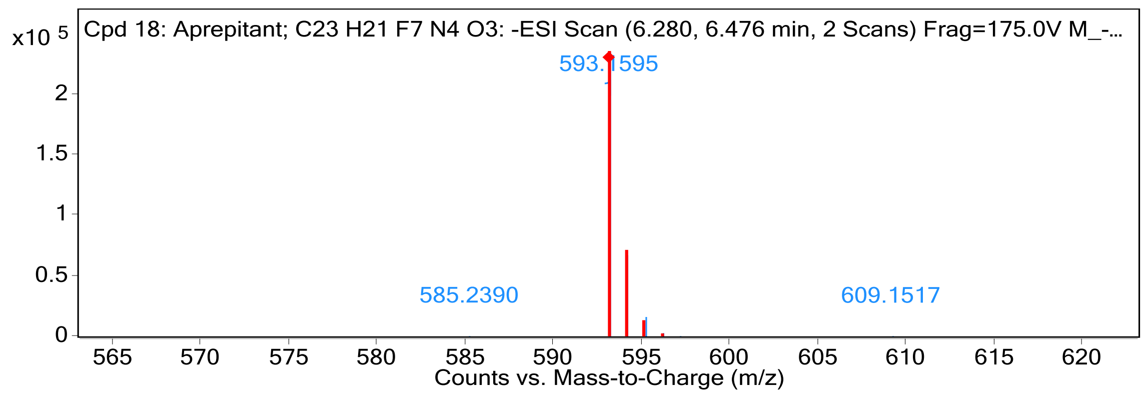

MS Spectrum Peak List

m/z	*Calc m/z*	Diff(ppm)	z	Abund	Formula	Ion
195.8143				24323.7		
197.8111			1	29069.69		
199.8082			1	12140.94		
593.1595	593.164	7.7	1	234970.86	C23 H21 F7 N4 O3	(M+CH3COO)-
594.1625	594.1671	7.72	1	68250.33	C23 H21 F7 N4 O3	(M+CH3COO)-
595.1654	595.1698	7.41	1	17246.52	C23 H21 F7 N4 O3	(M+CH3COO)-
596.1683	596.1724	6.9	1	3518.66	C23 H21 F7 N4 O3	(M+CH3COO)-
629.1358			1	42143.39		
630.139			1	12571.26		
631.1342			1	17195.97		

MSMS Spectrum

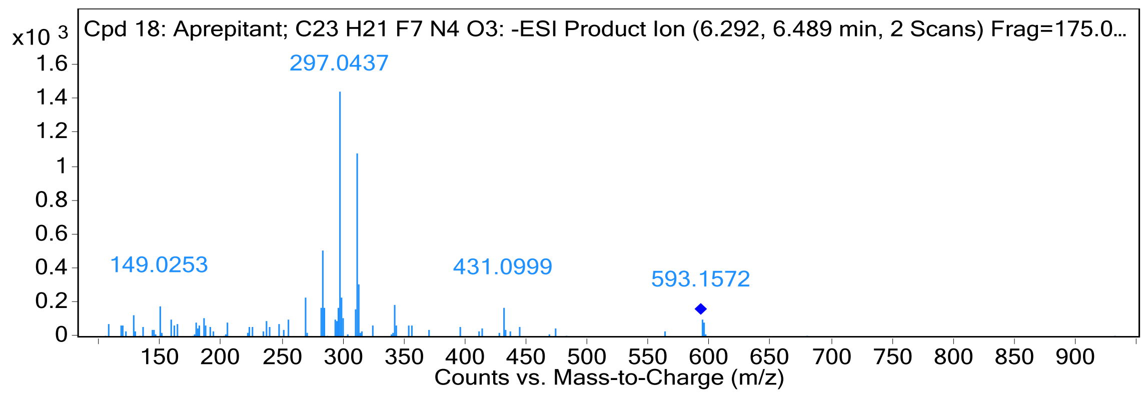

MS/MS Spectrum Peak List

m/z	z	Abund
149.0253		184.59
269.0497		230.9
282.0572		508.21
283.0651	1	478.54
295.0267	2	176.3
297.0437	1	1448.34
298.0482	1	237.27
311.0611	1	1080.92
312.0606	1	314.8
341.0541	1	192.52

Compound Structure

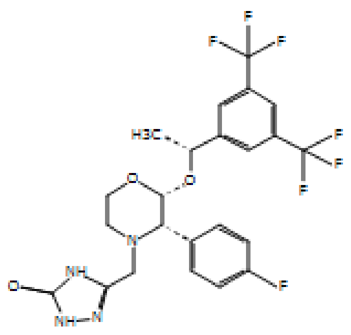

Compound Label	Name	*m/z*	RT	Algorithm	Mass
Cpd 19: CMP-N-glycoloylneuraminate; C20 H31 N4 O17 P	**CMP-N-glycoloylneuraminate**	629.1358	6.441	Auto MS/MS	630.1414

MS Spectrum

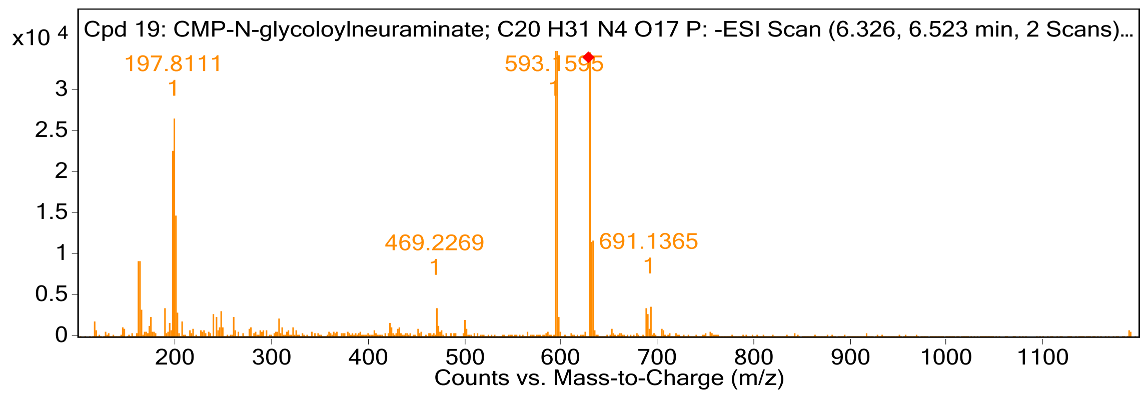

MS Zoomed Spectrum

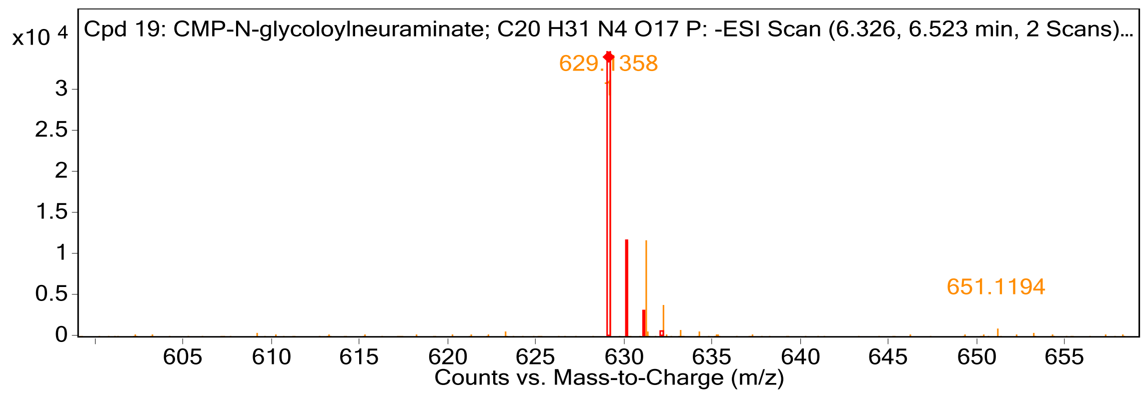

MS Spectrum Peak List

m/z	*Calc m/z*	Diff(ppm)	z	Abund	Formula	Ion
195.8143				22648.67		
197.8111			1	26598.34		
199.8083			1	14815.83		
593.1595			1	196446.97		
594.1628			1	56205.06		
629.1358	629.1349	-1.37	1	34675.7	C20 H31 N4 O17 P	(M-H)-
630.1391	630.1379	-1.78	1	11653.16	C20 H31 N4 O17 P	(M-H)-
631.1351	631.14	7.75	1	11816.2	C20 H31 N4 O17 P	(M-H)-
632.1356	632.1425	10.98	1	4013.13	C20 H31 N4 O17 P	(M-H)-
633.1398	633.1447	7.78	1	897.66	C20 H31 N4 O17 P	(M-H)-

MSMS Spectrum

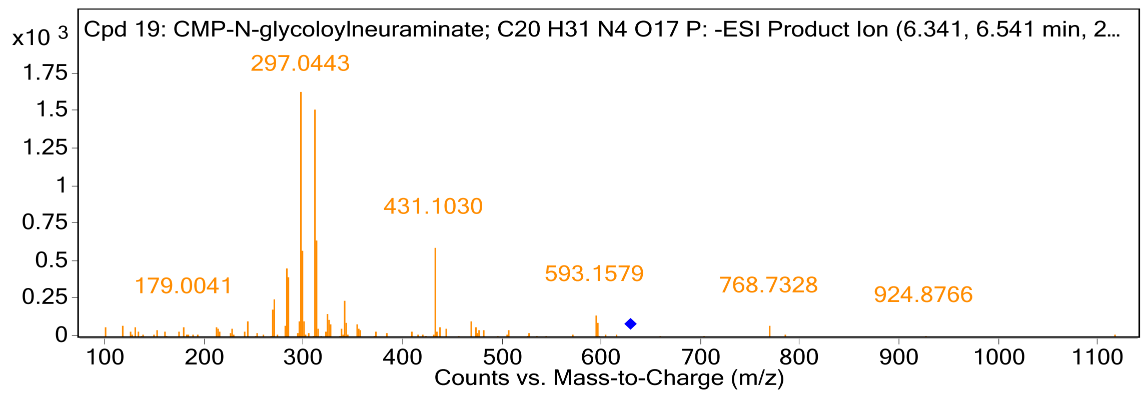

MS/MS Spectrum Peak List

m/z	z	Abund
268.0446		180.83
269.0452		258.4
282.0602		460.41
283.066	1	404.42
297.0443	1	1633.03
298.0479	1	574.76
311.0601	1	1517.32
312.0638	1	645.98
341.0711	1	244.26
431.103	1	599.68

Compound Structure

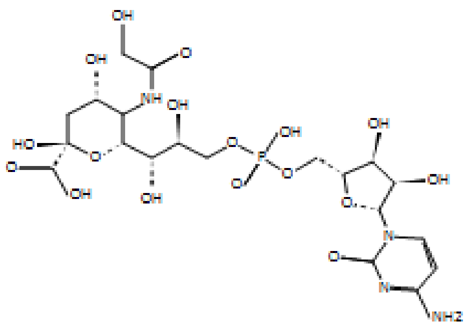

Compound Label	Name	*m/z*	RT	Algorithm	Mass
Cpd 20: Aprepitant; C23 H21 F7 N4 O3	**Aprepitant**	593.1591	6.686	Auto MS/MS	534.1455

MS Spectrum

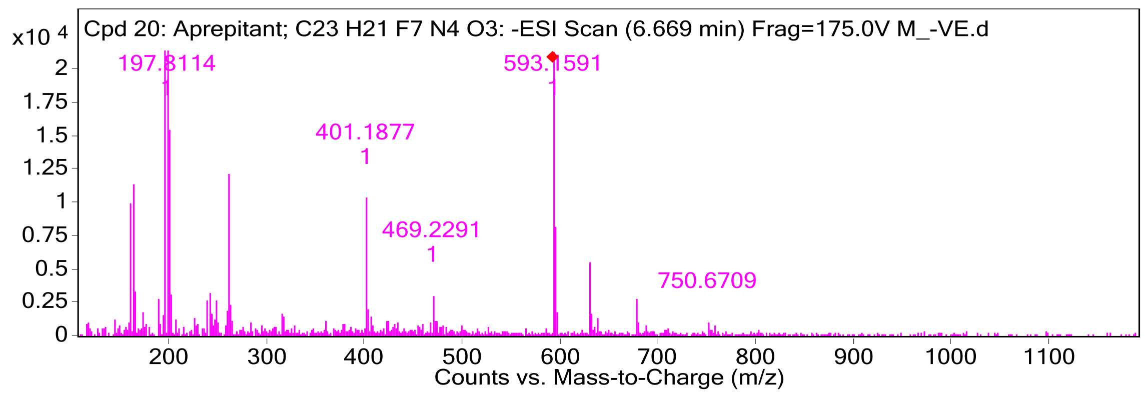

MS Zoomed Spectrum

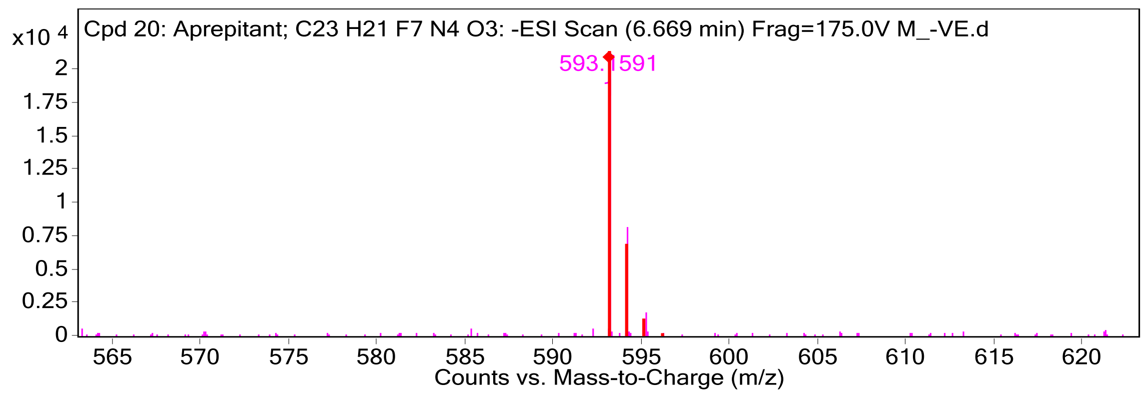

MS Spectrum Peak List

m/z	*Calc m/z*	Diff(ppm)	z	Abund	Formula	Ion
162.8422				11390.46		
195.8148				26219.04		
197.8114			1	30471.63		
199.8081			1	15529.44		
261.1378			1	12174		
401.1877			1	10464.68		
593.1591	593.164	8.31	1	21347.47	C23 H21 F7 N4 O3	(M+CH3COO)-
594.1635	594.1671	6.03	1	8272.9	C23 H21 F7 N4 O3	(M+CH3COO)-

595.1634	595.1698	10.72	1	1903.52	C23 H21 F7 N4 O3	(M+CH3COO)-
596.1606	596.1724	19.77	1	289.48	C23 H21 F7 N4 O3	(M+CH3COO)-

MSMS Spectrum

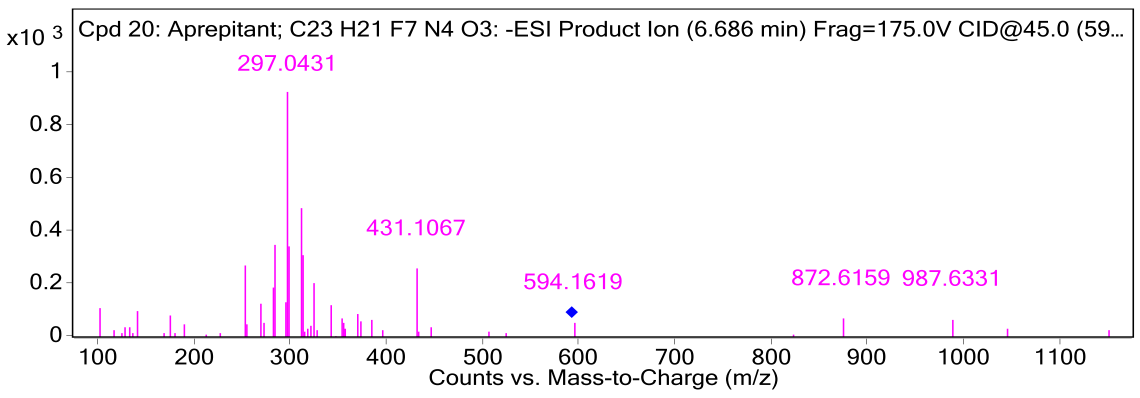

MS/MS Spectrum Peak List

m/z	z	Abund
253.0534		269.96
282.0598		188.13
283.0639	1	348.78
296.0359		150.96
297.0431	1	929.36
298.0529	1	345.88
311.0605		489.76
312.0628	1	312.36
324.0294		203.52
431.1067		259.22

Compound Structure

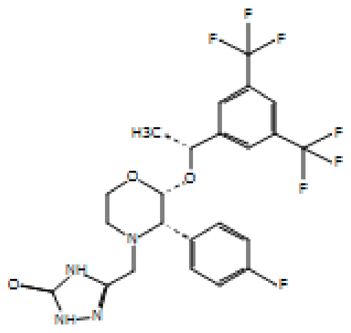

**Compound Label**	**Name**	***m/z***	**RT**	**Algorithm**	**Mass**
Cpd 21: Polyethylene, oxidized; C12 H20 O5	**Polyethylene, oxidized**	243.1275	8.44	Auto MS/MS	244.1348

MS Spectrum

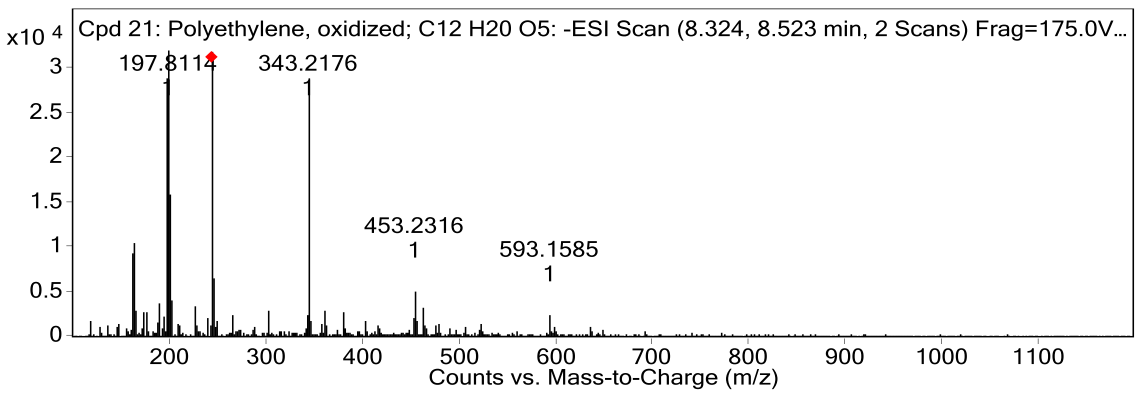

MS Zoomed Spectrum

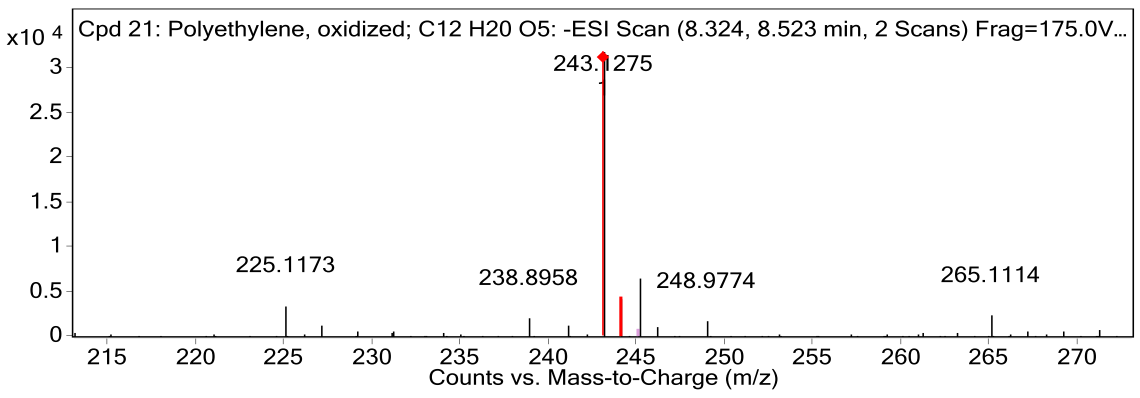

MS Spectrum Peak List

m/z	*Calc m/z*	Diff(ppm)	z	Abund	Formula	Ion
160.8445				9398.72		
162.8417				10518.82		
195.8142				28838.34		
197.8114			1	34797.68		
199.8085			1	15854.88		
243.1275	243.1238	-15.28	1	31855.99	C12 H20 O5	(M-H)-
244.1309	244.1272	-14.99	1	4247.65	C12 H20 O5	(M-H)-
245.1431			1	6608.24		
343.2176			1	28840.75		
344.2211			1	6575.52		

MSMS Spectrum

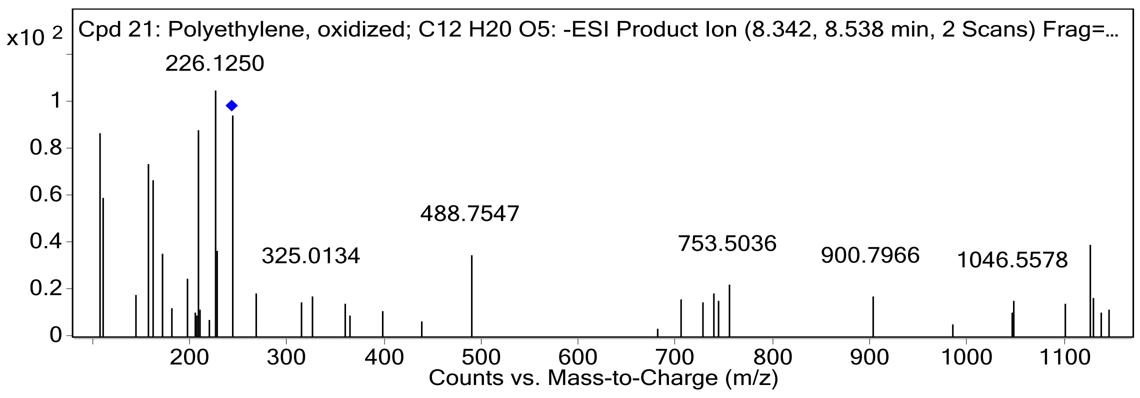

MS/MS Spectrum Peak List

m/z	z	Abund
107.0899		86.51
110.043		59.51
156.1442		73.51
161.1002		66.57
208.0863		88.26
225.0839		51.55
226.125	1	104.91

227.1366	1	36.82
243.1249	1	94.25
1125.4797		39.51

Compound Structure

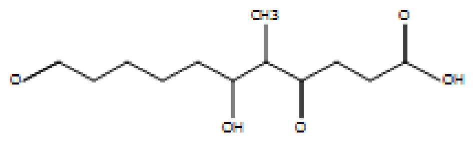

Compound Label	Name	*m/z*	RT	Algorithm	Mass
Cpd 22: Hemibrevetoxin B; C28 H42 O7	**Hemibrevetoxin B**	489.2776	9.682	Auto MS/MS	490.285

MS Spectrum

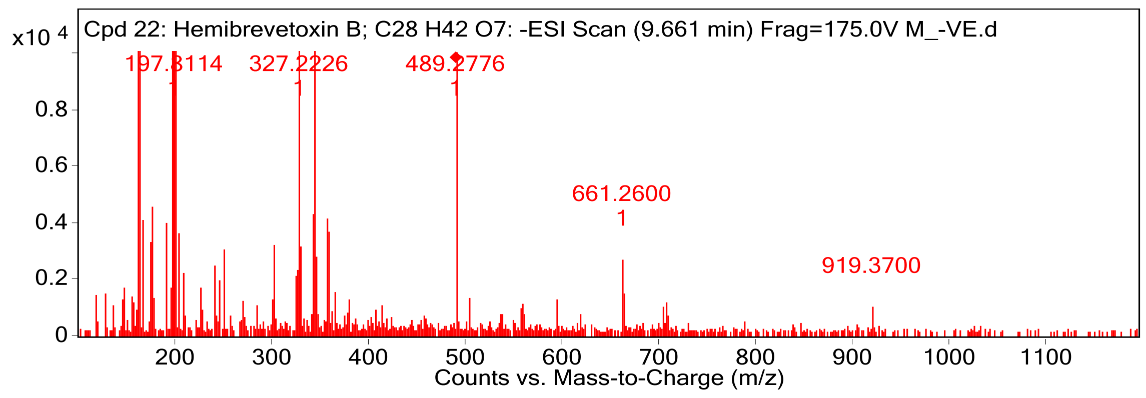

MS Zoomed Spectrum

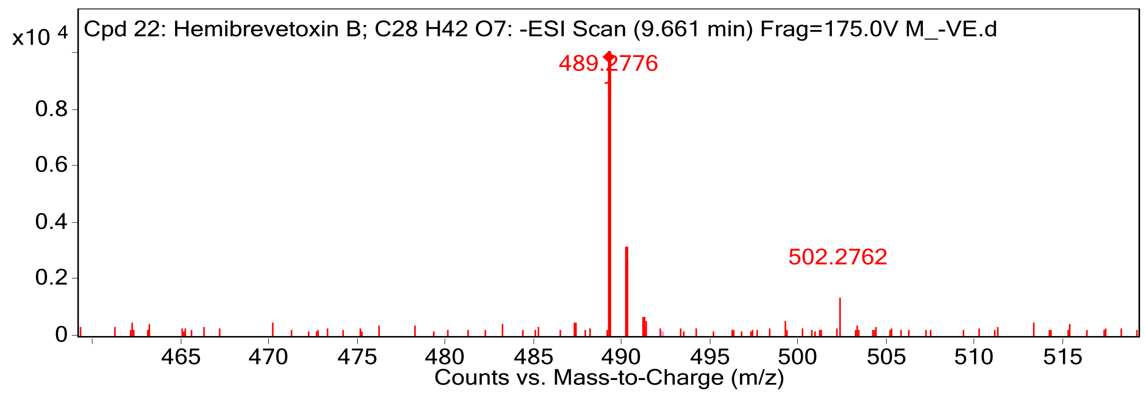

MS Spectrum Peak List

m/z	*Calc m/z*	Diff(ppm)	z	Abund	Formula	Ion
160.8448				13238.44		
162.8418				10874.79		
195.8145			1	29426.41		
197.8114			1	33874.52		
199.8087			1	16675.57		
327.2226			1	15607.21		
343.2175			1	12020.89		
489.2776	489.2858	16.67	1	10058.26	C28 H42 O7	(M-H)-
490.2815	490.2892	15.58	1	2744.13	C28 H42 O7	(M-H)-
491.2838	491.292	16.59	1	588.58	C28 H42 O7	(M-H)-

MSMS Spectrum

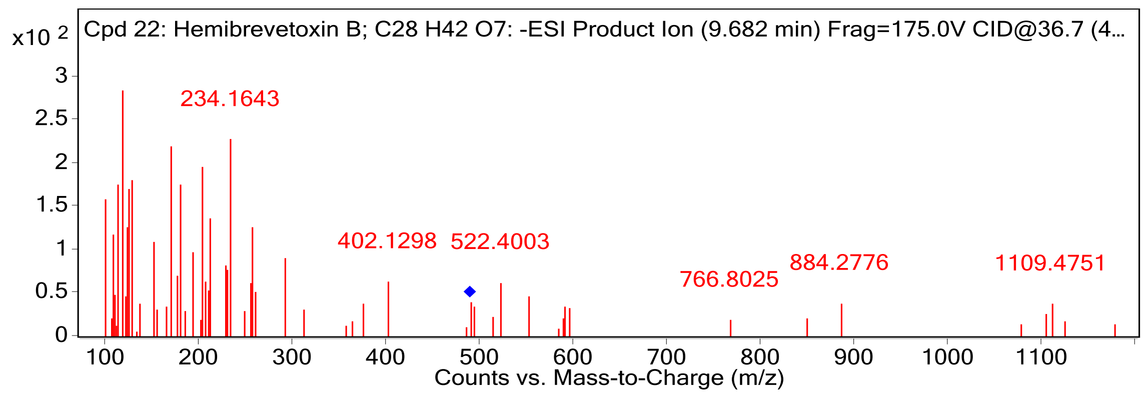

MS/MS Spectrum Peak List

m/z	Abund
101.0061	159.39
114.4964	175.53
119.0379	283.54
125.0963	171.15
129.0918	181.02
171.1046	219.4
180.1074	176.18
204.0101	195.97
212.1429	136.78
234.1643	228.06

Compound Structure

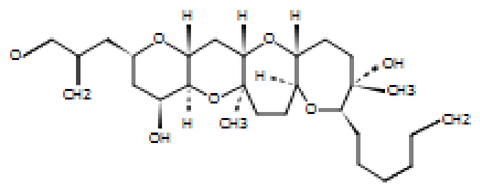

Compound Label	Name	*m/z*	RT	Algorithm	Mass

Cpd 23: (3b,6b,8a,12a)-8,12-Epoxy-7(11)-eremophilene-6,8,12-trimethoxy-3-ol; C18 H30 O5	**(3b,6b,8a,12a)-8,12-Epoxy-7(11)-eremophilene-6,8,12-trimethoxy-3-ol**	325.2072	11.121	Auto MS/MS	326.2146

MS Spectrum

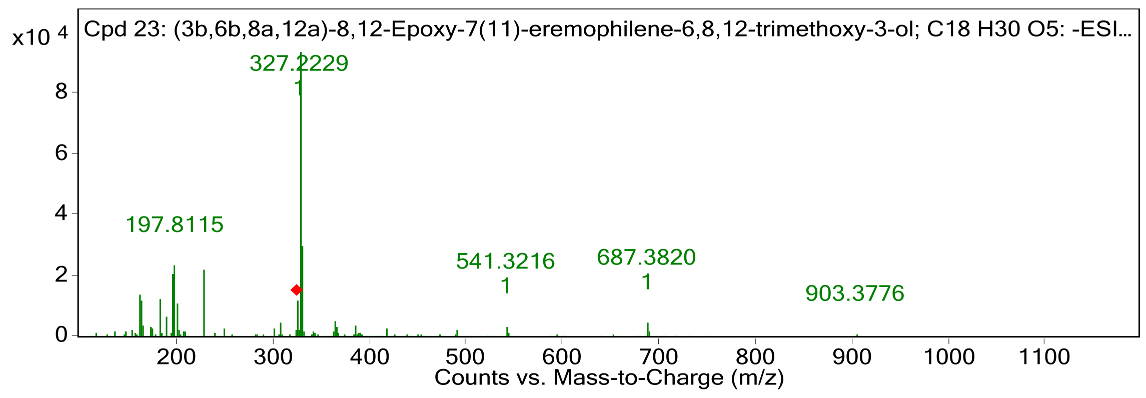

MS Zoomed Spectrum

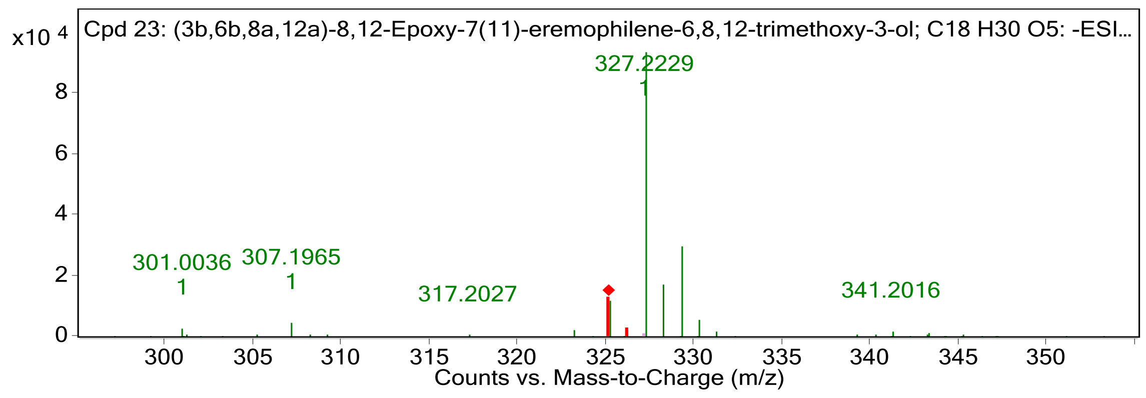

MS Spectrum Peak List

m/z	*Calc m/z*	Diff(ppm)	z	Abund	Formula	Ion
160.8449				14074.72		
183.1421			1	12517.68		
195.8145				20735.8		
197.8115				23528.47		
227.1328			1	22407.19		
325.2072	325.202	-15.86	1	12214.76	C18 H30 O5	(M-H)-
326.2111	326.2055	-17.42	1	2598.46	C18 H30 O5	(M-H)-
327.2229			1	93587.59		
328.2262			1	17443.43		
329.2388			1	29955.4		

MSMS Spectrum

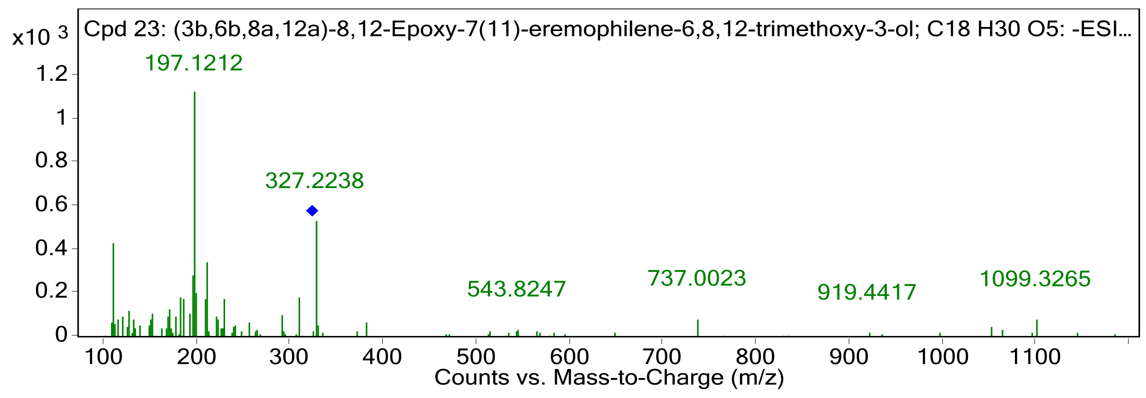

MS/MS Spectrum Peak List

m/z	z	Abund
111.0847	1	434.16
183.1405		182.19
186.1249		176.03
195.1429		281.5
197.1212	1	1127.43
199.1379		203.63
211.1358	1	346.78
229.1466		173.71
309.206		179.1
327.2238		530.39

Compound Structure

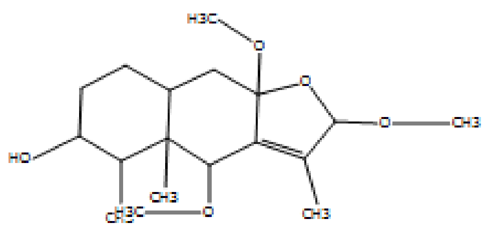

Compound Label	Name	*m/z*	RT	Algorithm	Mass
Cpd 24: (3b,6b,8a,12a)-8,12-Epoxy-7(11)-eremophilene-6,8,12-trimethoxy-3-ol; C18 H30 O5	**(3b,6b,8a,12a)-8,12-Epoxy-7(11)-eremophilene-6,8,12-trimethoxy-3-ol**	325.2073	11.51	Auto MS/MS	326.2145

MS Spectrum

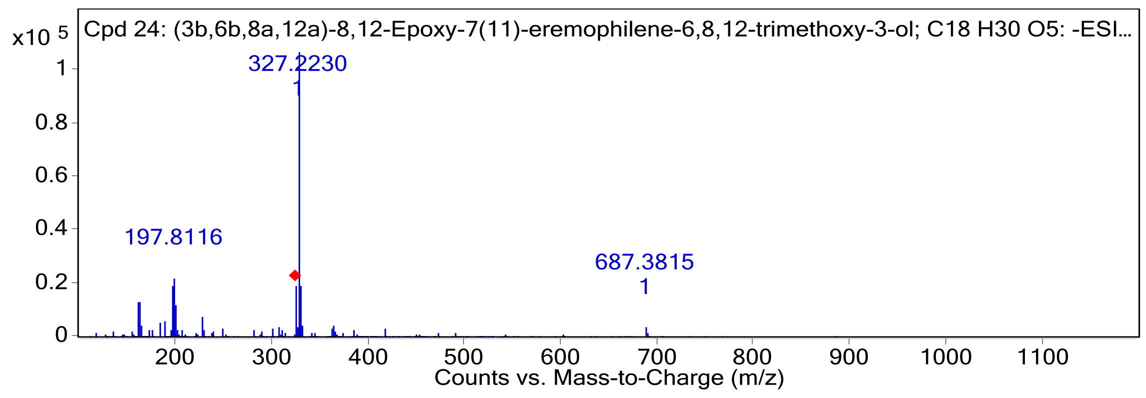

MS Zoomed Spectrum

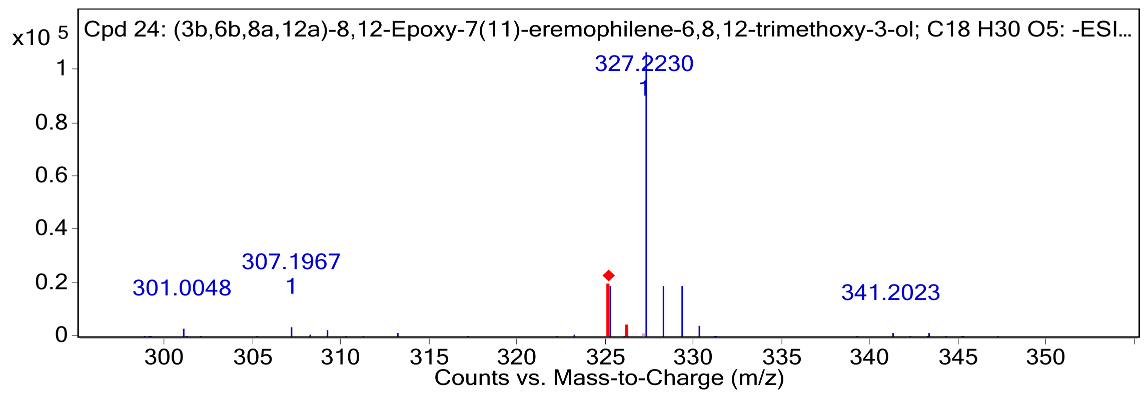

MS Spectrum Peak List

m/z	*Calc m/z*	Diff(ppm)	z	Abund	Formula	Ion
160.845				13441.61		
162.8419				13054.08		
195.8149				19209.41		
197.8116				22235.05		
199.808				11930.77		
325.2073	325.202	-16.1	1	19439.14	C18 H30 O5	(M-H)-
326.2102	326.2055	-14.62	1	3994.06	C18 H30 O5	(M-H)-
327.223			1	106832.79		
328.2266			1	19185.46		
329.2379			1	19528.62		

MSMS Spectrum

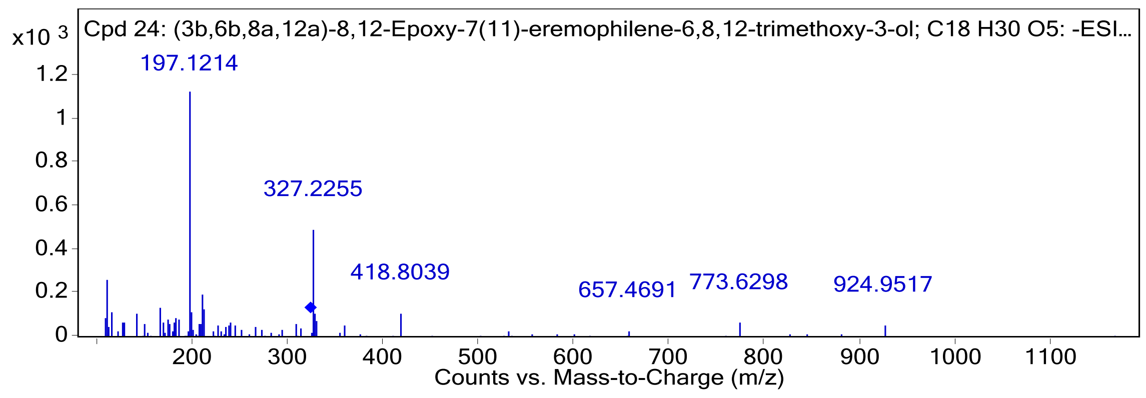

MS/MS Spectrum Peak List

m/z	z	Abund
111.0822	1	263.97
116.0639		114.95
165.5444		132.63
197.1214		1127.42
199.1373		113.42
210.1262		195.27
211.1373		130.48
327.2255	1	493.74
328.2301	1	110.65
418.8039		110.31

Compound Structure

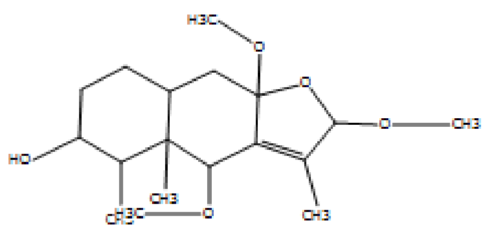

Compound Label	Name	*m/z*	RT	Algorithm	Mass
Cpd 25: (3b,6b,8a,12a)-8,12-Epoxy-7(11)-eremophilene-6,8,12-trimethoxy-3-ol; C18 H30 O5	**(3b,6b,8a,12a)-8,12-Epoxy-7(11)-eremophilene-6,8,12-trimethoxy-3-ol**	325.2071	11.895	Auto MS/MS	326.2144

MS Spectrum

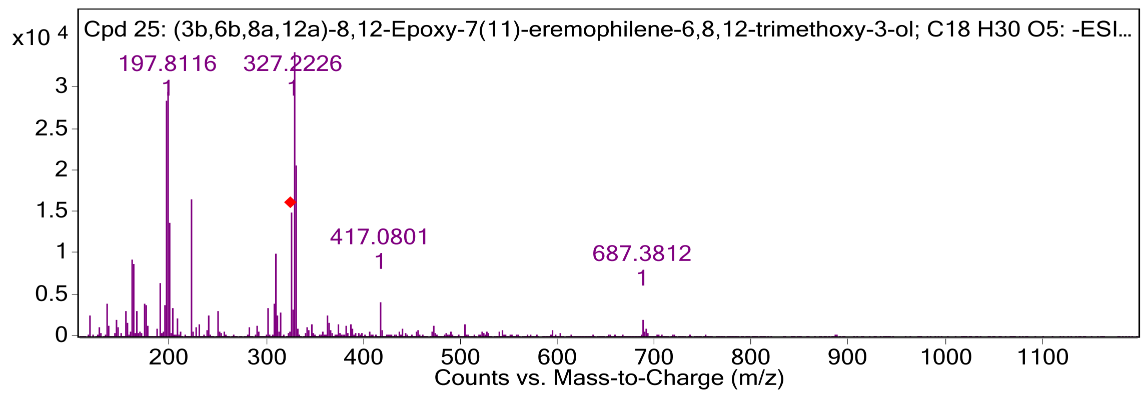

MS Zoomed Spectrum

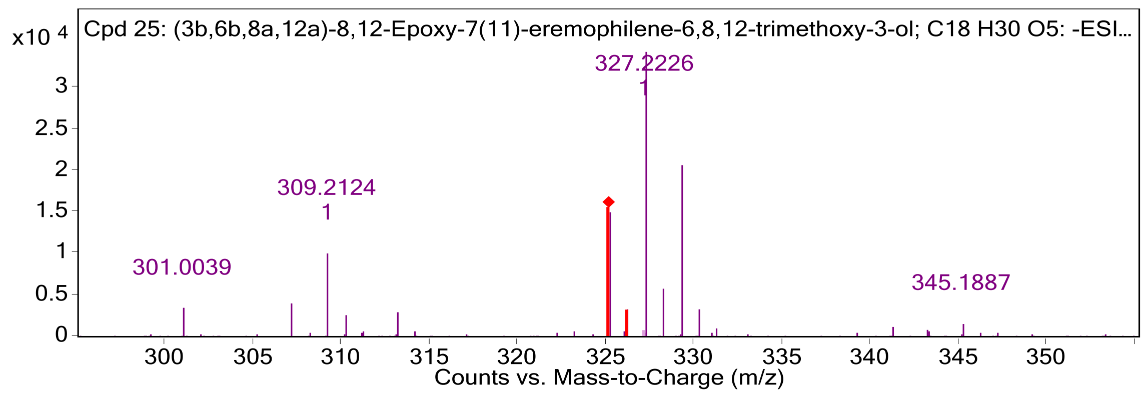

MS Spectrum Peak List

m/z	*Calc m/z*	Diff(ppm)	z	Abund	Formula	Ion
160.845				9393.13		
195.8147				28497.75		
197.8116			1	29610.03		
199.8082			1	13755.2		

221.1221			1	16680.98		
309.2124			1	10125.04		
325.2071	325.202	-15.45	1	15067.16	C18 H30 O5	(M-H)-
326.2107	326.2055	-15.97	1	3443.46	C18 H30 O5	(M-H)-
327.2226			1	34334.53		
329.2384			1	20696.46		

MSMS Spectrum

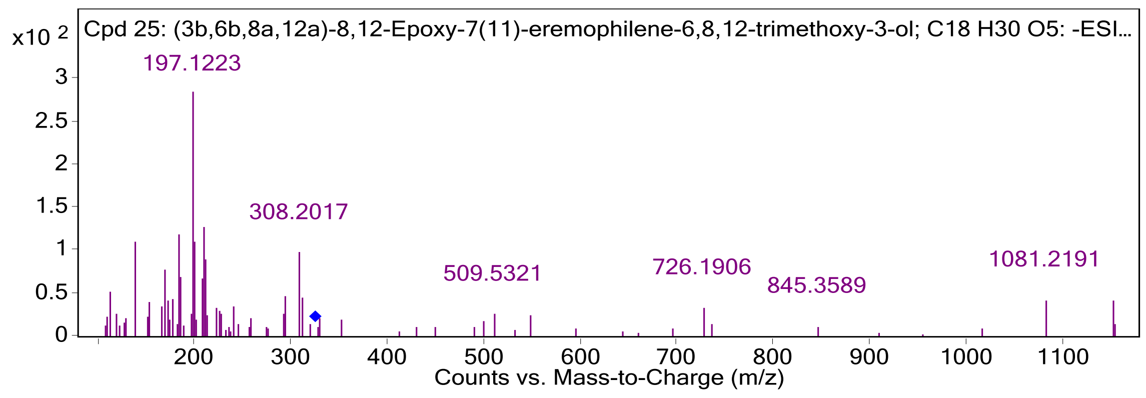

MS/MS Spectrum Peak List

m/z	z	Abund
137.1013		110.24
169.1165		78.15
183.0098		120.21
197.1223	1	286.17
198.038		73.6
198.1221	1	101.19
199.137		111.1
209.1129		128.07
211.124		89.93
308.2017		98.28

Compound Structure

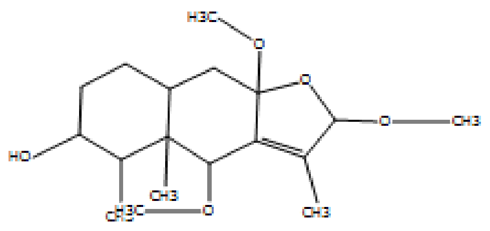

Compound Label	Name	*m/z*	RT	Algorithm	Mass
Cpd 26: Saponin H; C36 H58 O10	**Saponin H**	649.3886	17.125	Auto MS/MS	650.3962

MS Spectrum

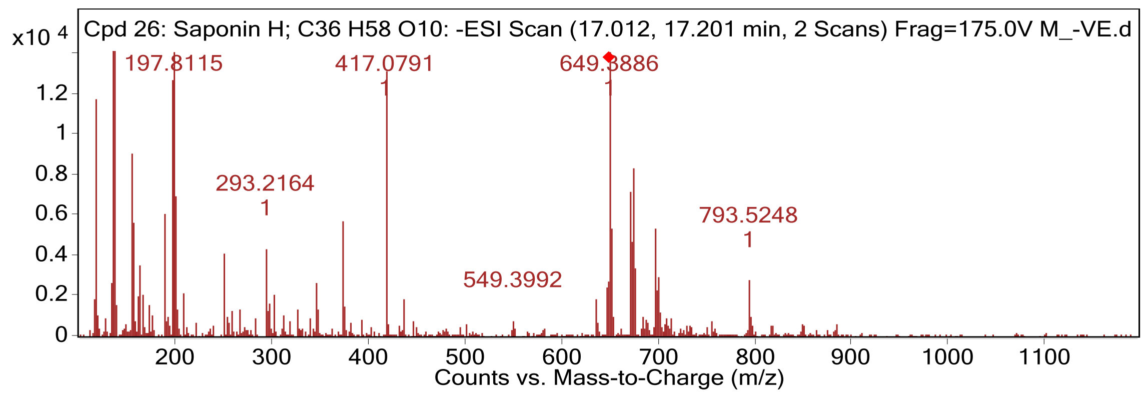

MS Zoomed Spectrum

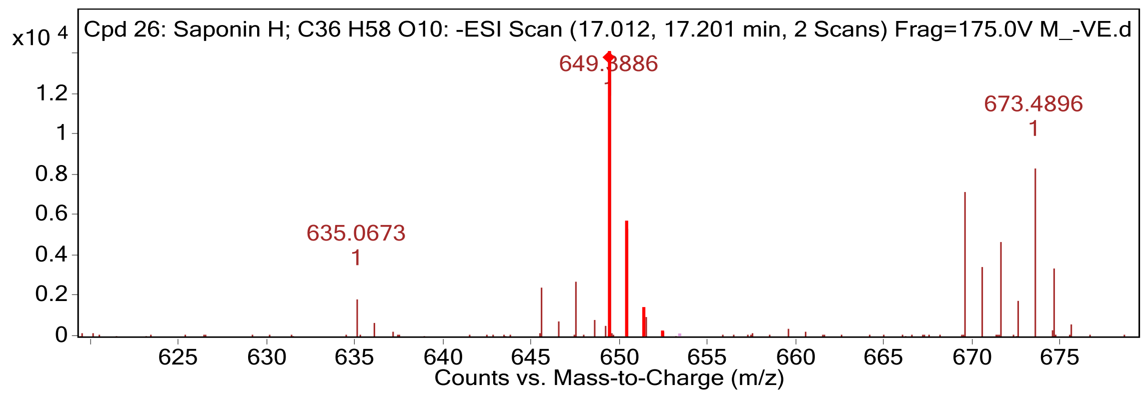

MS Spectrum Peak List

m/z	*Calc m/z*	Diff(ppm)	z	Abund	Formula	Ion
116.9312			1	11805.89		
134.8973			1	50802.94		
136.8945			1	17787.77		
195.8146				12750.57		
197.8115				14127.26		
417.0791			1	13169.6		
649.3886	649.3957	10.94	1	14103.76	C36 H58 O10	(M-H)-
650.3935	650.3991	8.6	1	5352.2	C36 H58 O10	(M-H)-
651.3927	651.402	14.23	1	991.35	C36 H58 O10	(M-H)-
652.4005	652.4048	6.65	1	305.31	C36 H58 O10	(M-H)-

MSMS Spectrum

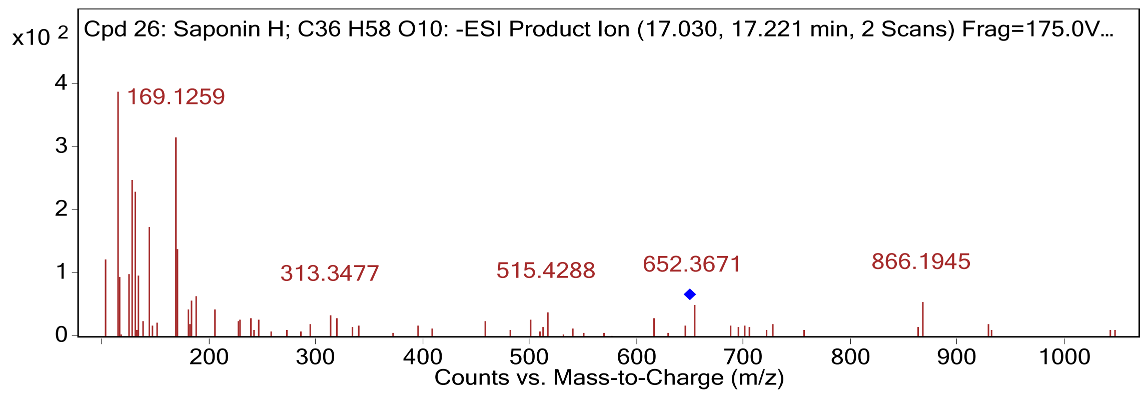

MS/MS Spectrum Peak List

m/z	z	Abund
103.0424		123.17

115.0431	1	389.82
116.0468	1	94.81
125.1004		99.11
127.0427	1	250.36
131.0365		229.8
133.0425		97.93

m/z	z	Abund
143.037		175.67
169.1259		316.89
170.1293		139.19

Compound Structure

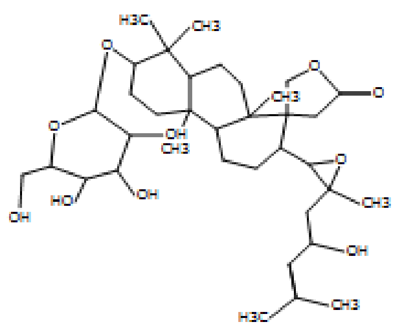

Compound Label	Name	*m/z*	RT	Algorithm	Mass
Cpd 27: Hexazinone; C12 H20 N4 O2	**Hexazinone**	297.1579	18.076	Auto MS/MS	252.1596

MS Spectrum

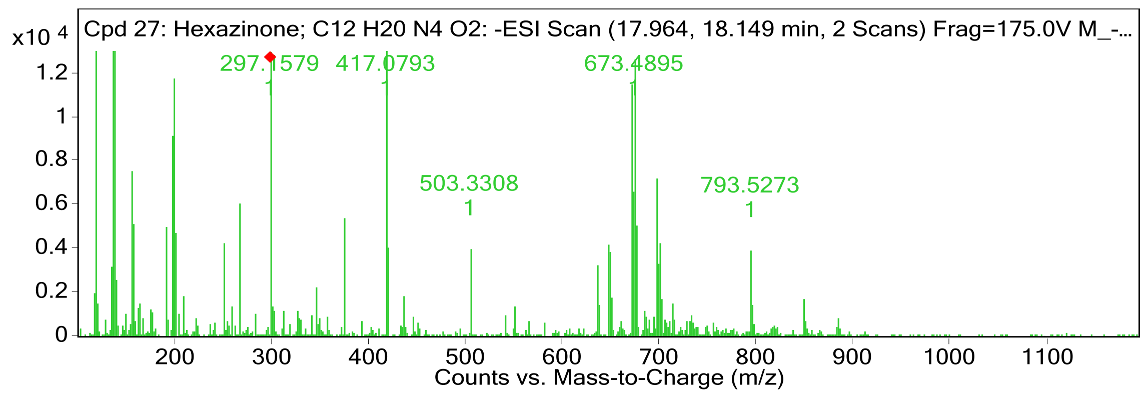

MS Zoomed Spectrum

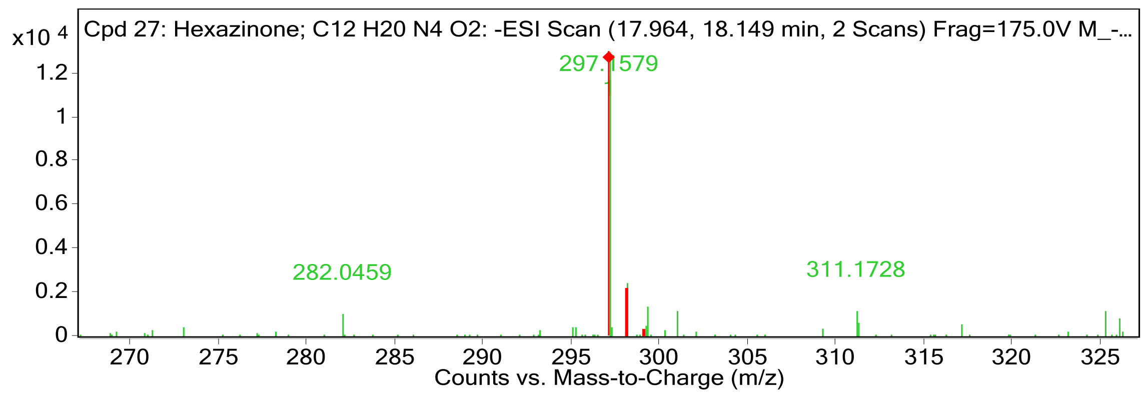

MS Spectrum Peak List

m/z	*Calc m/z*	Diff(ppm)	z	Abund	Formula	Ion
116.931			1	17410.61		
134.8973			1	64953.45		
136.8944			1	23506.8		
197.8119				11759.04		
297.1579	297.1568	-3.62	1	12979.01	C12 H20 N4 O2	(M+HCOO)-
298.1618	298.1597	-7.11	1	2465.88	C12 H20 N4 O2	(M+HCOO)-
299.1566	299.1619	17.76	1	547.07	C12 H20 N4 O2	(M+HCOO)-
417.0793			1	13066.1		
669.4585			1	11530.61		
673.4895			1	12621.34		

MSMS Spectrum

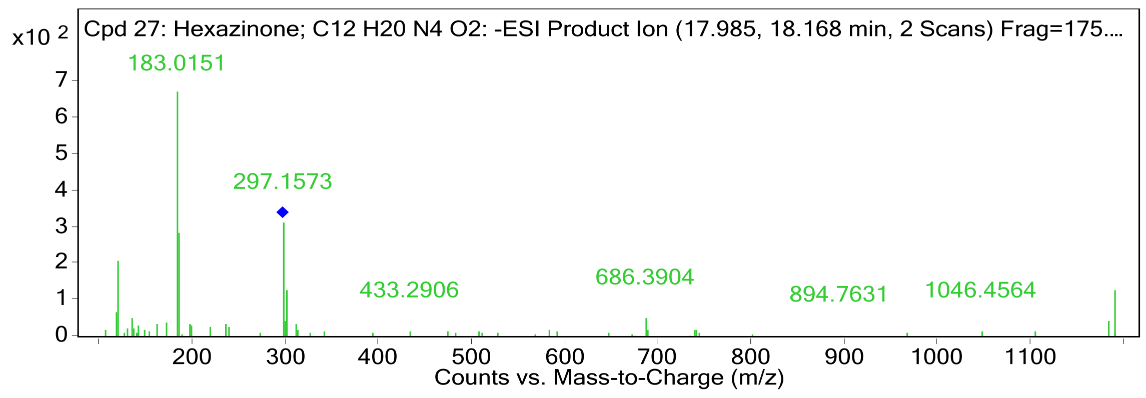

MS/MS Spectrum Peak List

m/z	z	Abund
117.0283		66.62
119.043		92.25
119.0533		207.87
183.0151		674.11
184.0159	1	286.74
297.1573		314.93
300.9791		56.13
301.0068		127.94
686.3904		52.2
1188.4838		127.99

Compound Structure

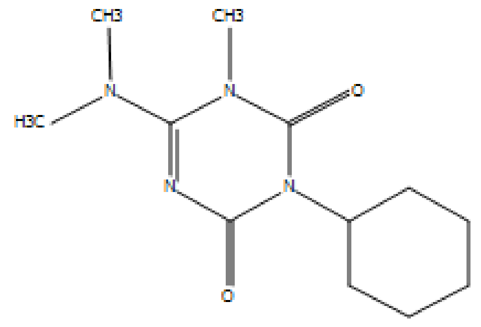

Compound Label	Name	*m/z*	RT	Algorithm	Mass
Cpd 28: Hexazinone; C12 H20 N4 O2	**Hexazinone**	297.1581	18.346	Auto MS/MS	252.1595

MS Spectrum

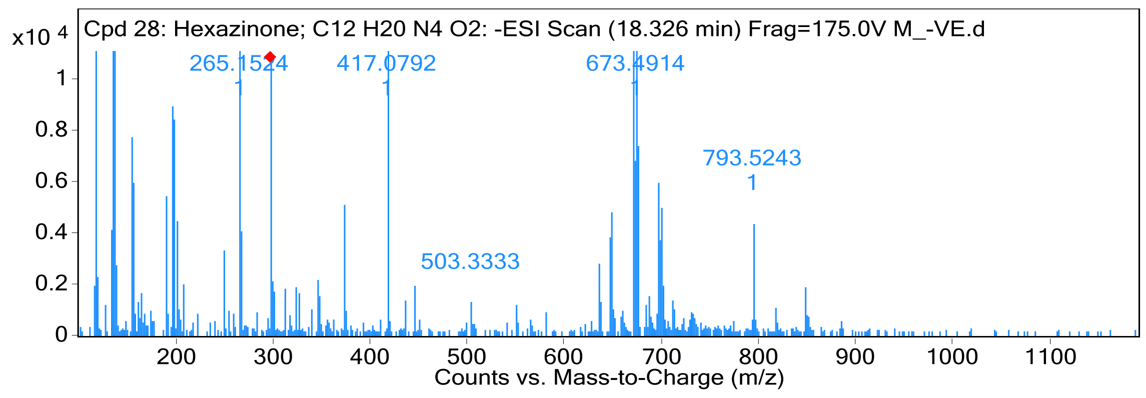

MS Zoomed Spectrum

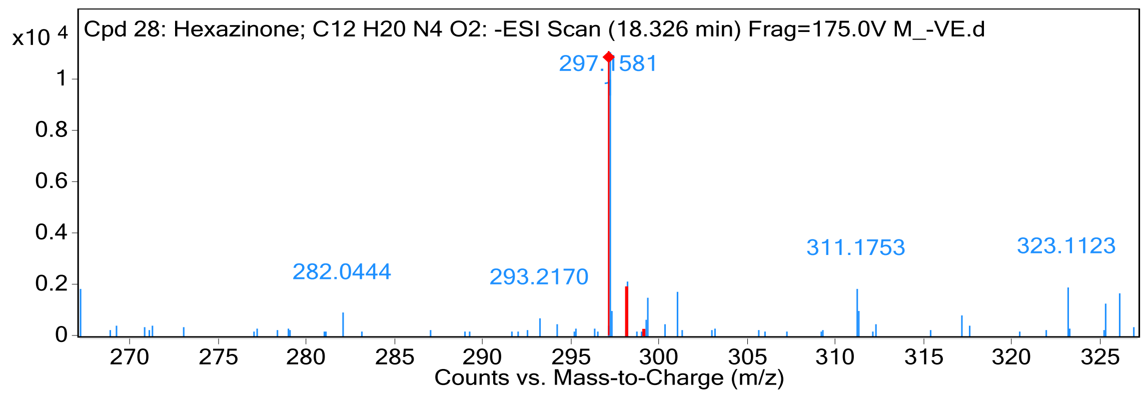

MS Spectrum Peak List

m/z	*Calc m/z*	Diff(ppm)	z	Abund	Formula	Ion
116.931			1	14324.27		
134.8973			1	75637.02		
136.8943			1	23729.52		
265.1524			1	29455.7		
297.1581	297.1568	-4.4	1	11086.9	C12 H20 N4 O2	(M+HCOO)-
298.1603	298.1597	-2.19	1	2197	C12 H20 N4 O2	(M+HCOO)-
299.1571	299.1619	16.13	1	674.83	C12 H20 N4 O2	(M+HCOO)-
417.0792			1	14369		
669.459			1	13854.76		
673.4914			1	14106.79		

MSMS Spectrum

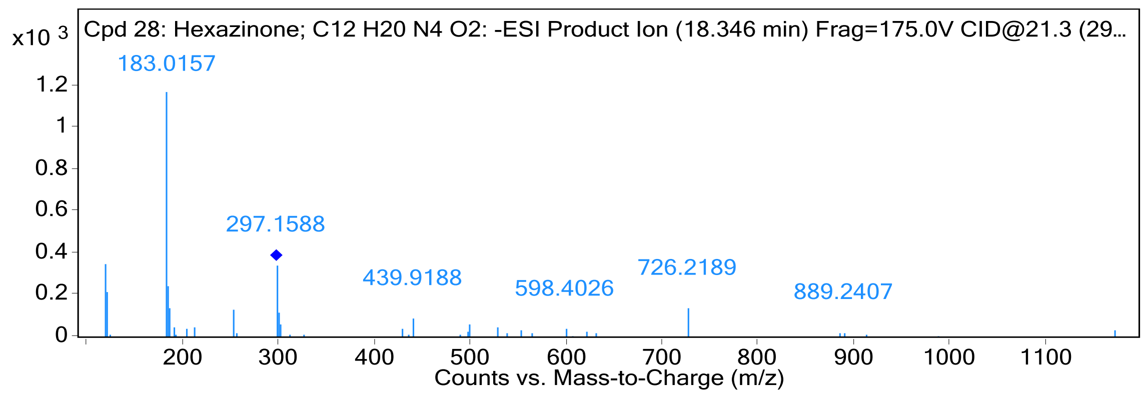

MS/MS Spectrum Peak List

m/z	z	Abund
119.0537		352.51
120.0348		218.47
183.0157	1	1170.72
184.0185	1	246.7
185.0162	1	140.42
253.0908		134.5
297.1588	1	345.31
299.2522	1	117.04
439.9188		89.19
726.2189		138.41

Compound Structure

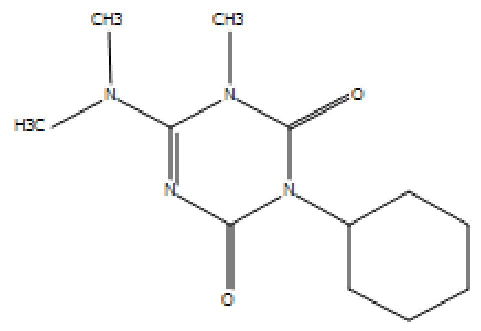

Compound Label	Name	*m/z*	RT	Algorithm	Mass
Cpd 29: Hydroquinidine; C20 H26 N2 O2	**Hydroquinidine**	325.1899	21.674	Auto MS/MS	326.1968

MS Spectrum

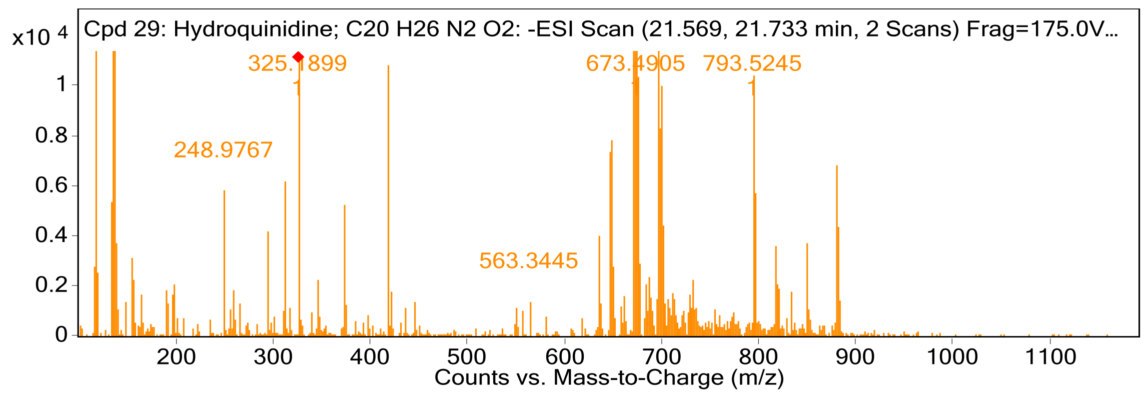

MS Zoomed Spectrum

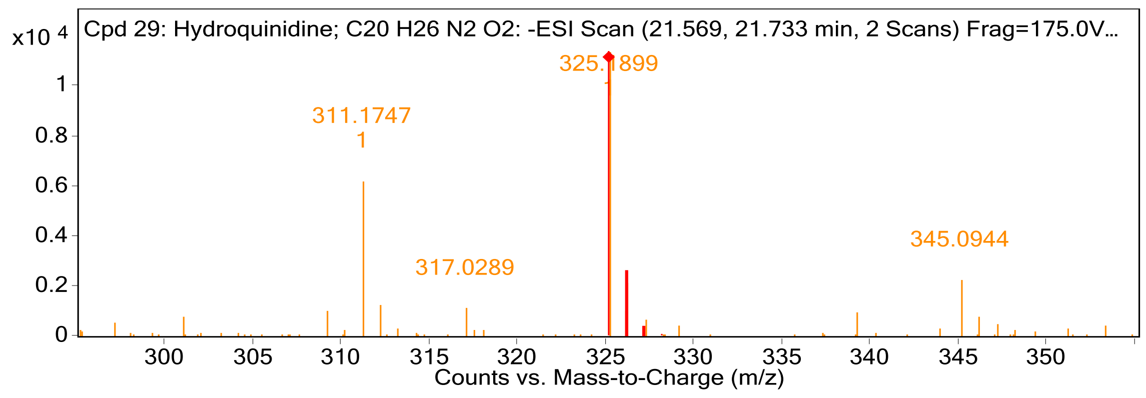

MS Spectrum Peak List

m/z	*Calc m/z*	Diff(ppm)	z	Abund	Formula	Ion
116.931			1	18504.14		
134.8974			1	100140.8		
136.8946			1	33324.49		
325.1899	325.1922	6.83	1	11371.83	C20 H26 N2 O2	(M-H)-
326.193	326.1953	7.15	1	2150.34	C20 H26 N2 O2	(M-H)-
327.1904	327.1982	23.84	1	722.61	C20 H26 N2 O2	(M-H)-
328.19	328.2009	33.5	1	142.58	C20 H26 N2 O2	(M-H)-
669.4595			1	23033.7		
671.4736			1	16143.19		
673.4905			1	27820.27		

MSMS Spectrum

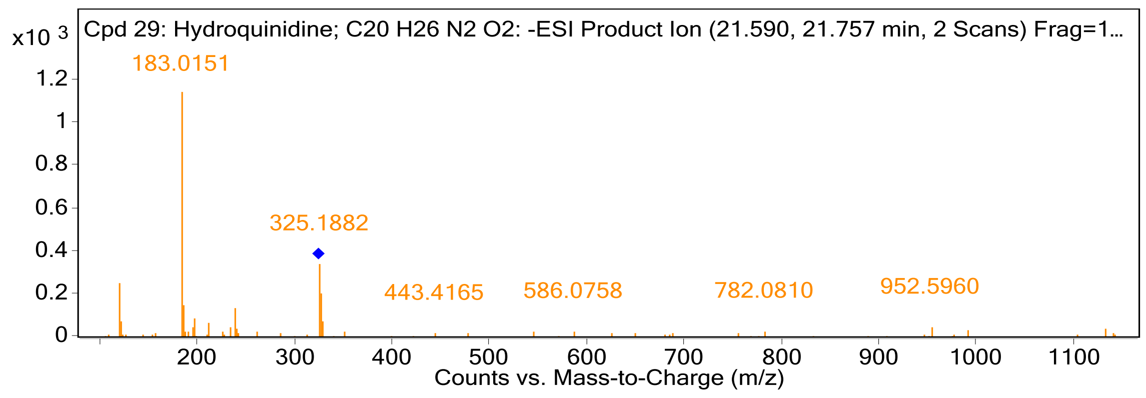

MS/MS Spectrum Peak List

m/z	z	Abund
119.0523	1	251.95
121.0822		73.59
183.0151		1147.73
184.02	1	515.94
185.02	1	148.53
197.0279		87.63
238.0536	1	136.59
325.1882		343.53
326.1911		207.15
327.1324		78.6

Compound Structure

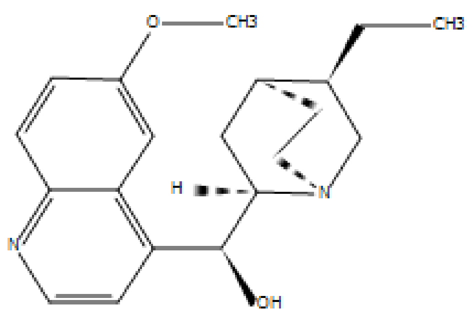

Compound Label	Name	*m/z*	RT	Algorithm	Mass
Cpd 30: Hydroquinidine; C20 H26 N2 O2	**Hydroquinidine**	325.1897	22.039	Auto MS/MS	326.1965

MS Spectrum

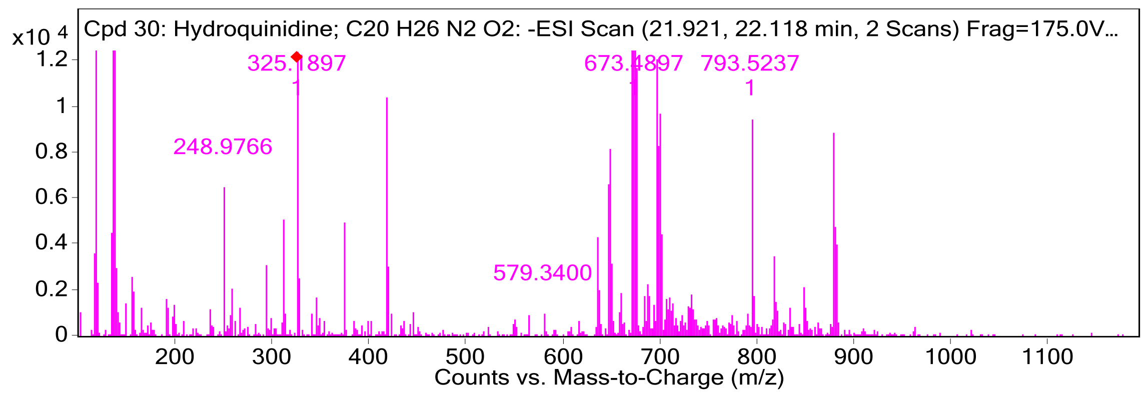

MS Zoomed Spectrum

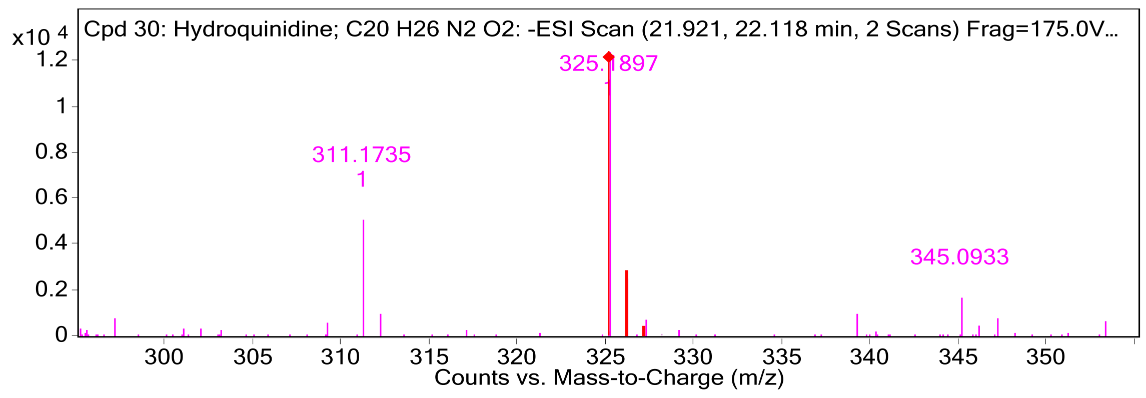

MS Spectrum Peak List

m/z	*Calc m/z*	Diff(ppm)	z	Abund	Formula	Ion
116.9311			1	17321.5		
134.8974			1	101988.34		
136.8945			1	33715.11		
325.1897	325.1922	7.61	1	12398.76	C20 H26 N2 O2	(M-H)-
326.1916	326.1953	11.47	1	2534.42	C20 H26 N2 O2	(M-H)-
327.1913	327.1982	21.2	1	755.25	C20 H26 N2 O2	(M-H)-
669.4584			1	21216.01		
671.4737			1	16291.51		

673.4897			1	26733.68		
695.4748			1	12076.31		

MSMS Spectrum

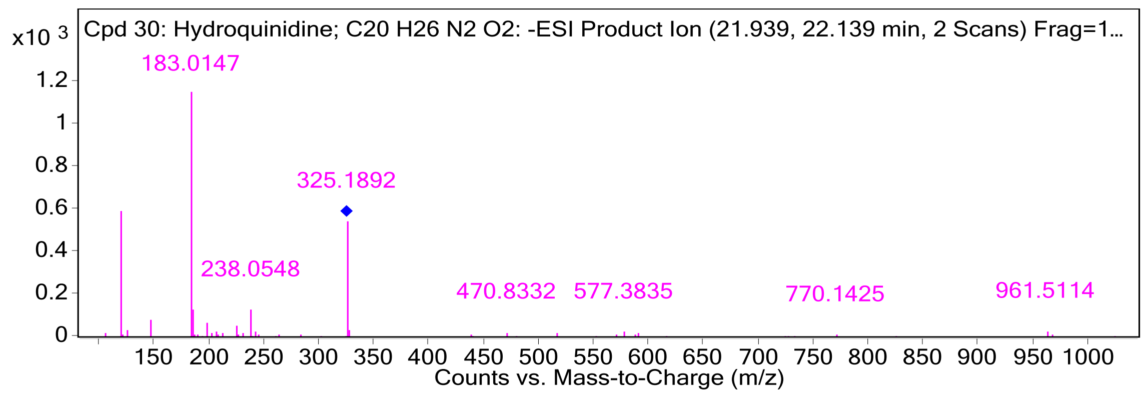

MS/MS Spectrum Peak List

m/z	z	Abund
119.0412		106.42
119.0521		590.94
146.5253		79.56
182.9833	2	107.87
183.0147	1	1155.19
183.9832	2	72.01
184.9539		131.77
185.014	1	75.08
238.0548		128.57
325.1892	1	546.58

Compound Structure

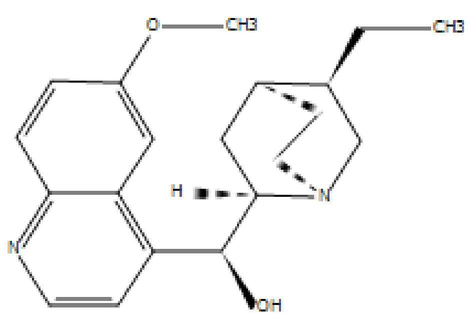

Compound Label	Name	*m/z*	RT	Algorithm	Mass
Cpd 31: Hydroquinidine; C20 H26 N2 O2	**Hydroquinidine**	325.1901	22.372	Auto MS/MS	326.1966

MS Spectrum

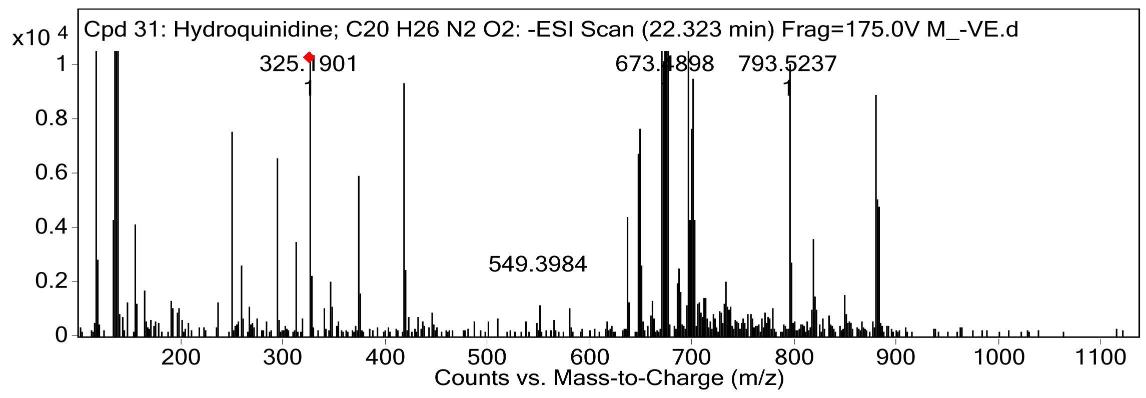

MS Zoomed Spectrum

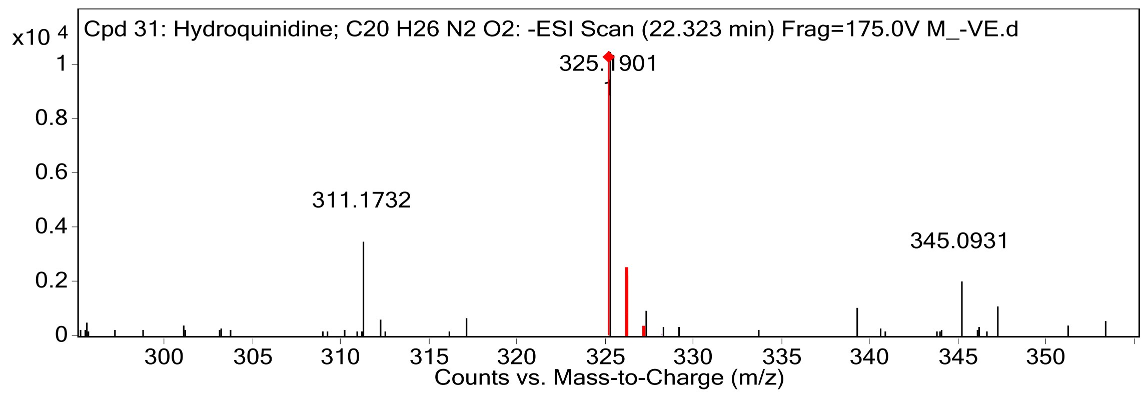

MS Spectrum Peak List

m/z	*Calc m/z*	Diff(ppm)	z	Abund	Formula	Ion
116.9308			1	17778.61		
134.8973			1	100796.41		
136.8945			1	32582.1		
325.1901	325.1922	6.39	1	10488.24	C20 H26 N2 O2	(M-H)-
326.1931	326.1953	6.84	1	2279.1	C20 H26 N2 O2	(M-H)-
327.1857	327.1982	38.24	1	959.63	C20 H26 N2 O2	(M-H)-
669.459			1	20933.58		
671.4747			1	15067.32		
673.4898			1	25512.18		
695.4743			1	12829.21		

MSMS Spectrum

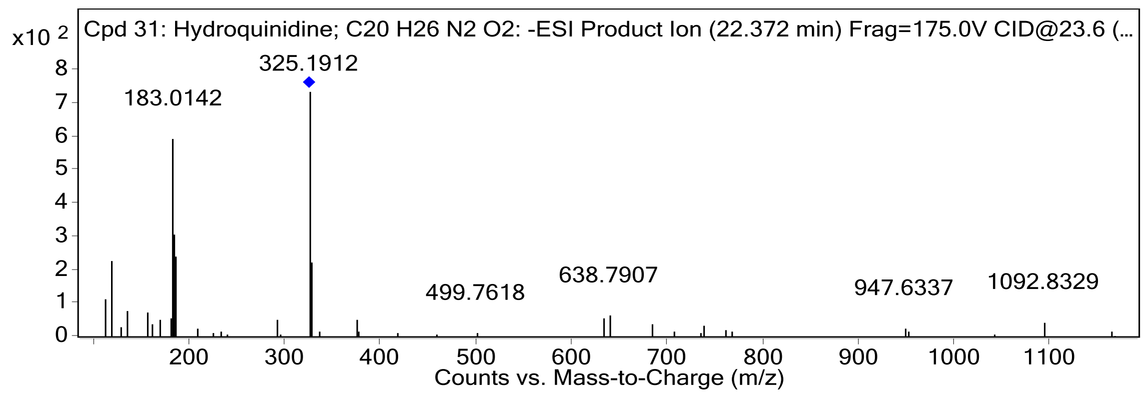

MS/MS Spectrum Peak List

m/z	z	Abund
112.312		114.16
119.0507		230.91
135.0304		77.16
183.0142		595.67
184.0154		307.57

185.0286		180.2
186.0164		240.17
325.1912	1	736.74
326.1957	1	213.63
327.1957	1	225.62

Compound Structure

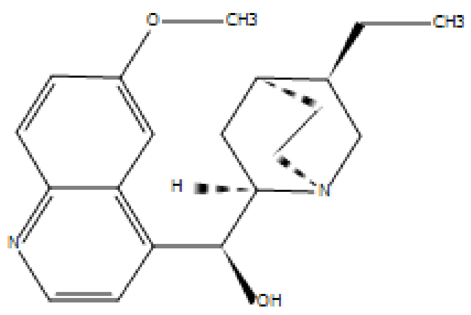

Compound Label	*m/z*	RT	Algorithm
Compound 32	694.4618	22.494	Auto MS/MS

MS Spectrum

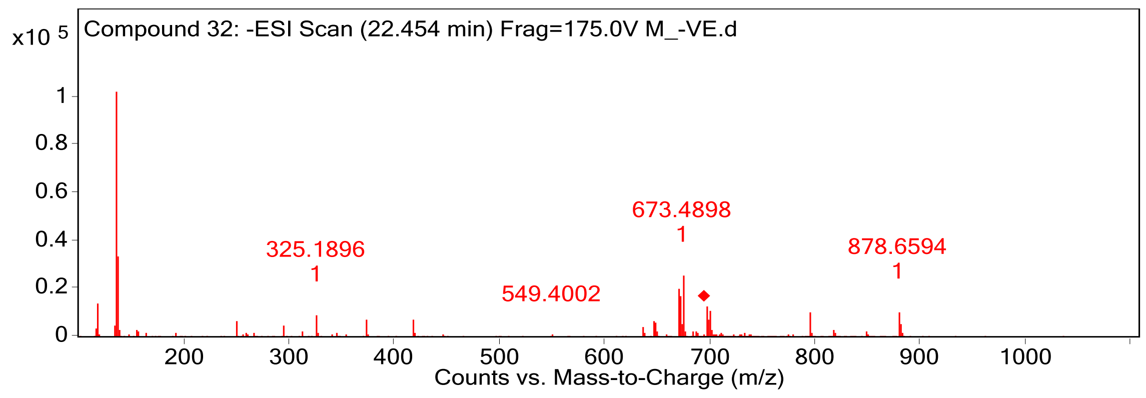

MS Zoomed Spectrum

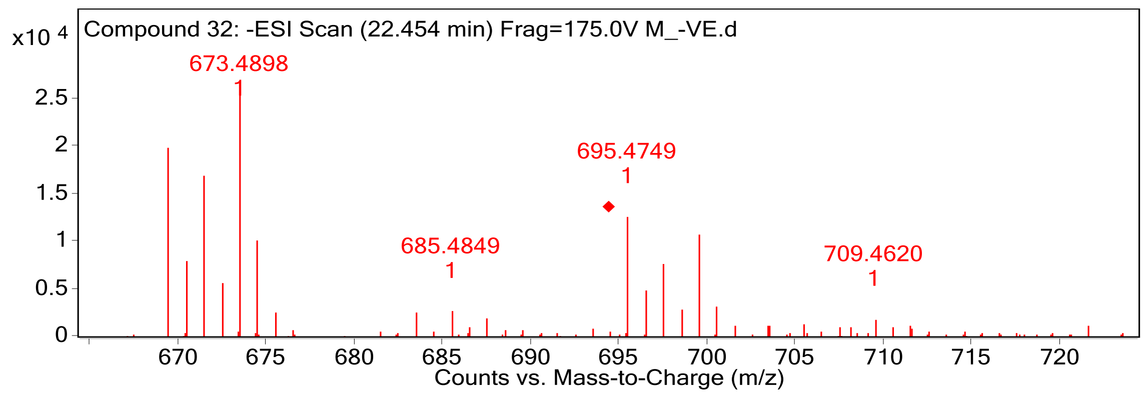

MS Spectrum Peak List

m/z	z	Abund
116.931	1	14290.77
134.8973	1	102333.19
135.8973	1	12284.94
136.8945	1	33585.71
669.4588	1	19913.49
671.4734	1	16910.32
673.4898	1	25770.55
694.4618	2	627.41
694.9629	2	359
695.4749	1	12667.61

MSMS Spectrum

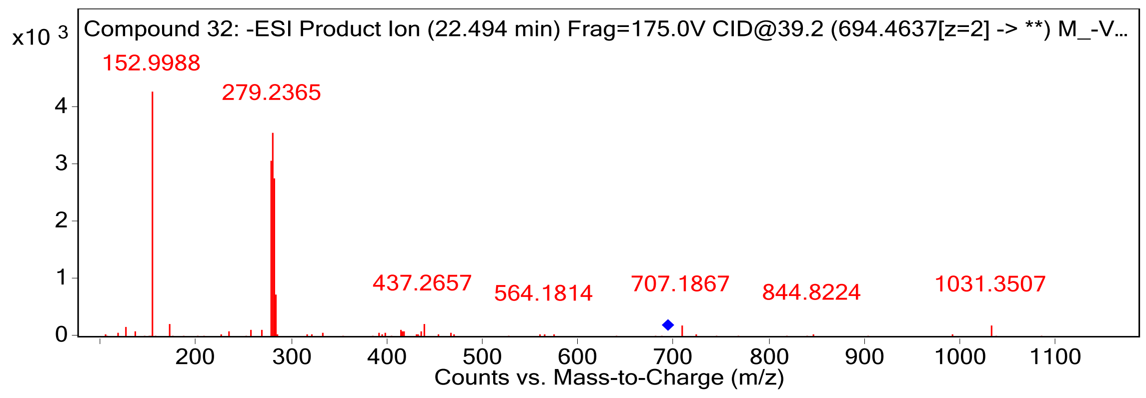

MS/MS Spectrum Peak List

m/z	z	Abund
152.9988	1	4279.45
171.0064		231.93
277.2211	1	3077.62
278.224	1	626.2
279.2365	1	3554.25
280.2408	1	581.25
281.253	1	2768.37
282.2523	1	749.15
283.2669	1	597.94
437.2657		225.79

Compound Label	*m/z*	RT	Algorithm
Compound 33	694.4656	22.845	Auto MS/MS

MS Spectrum

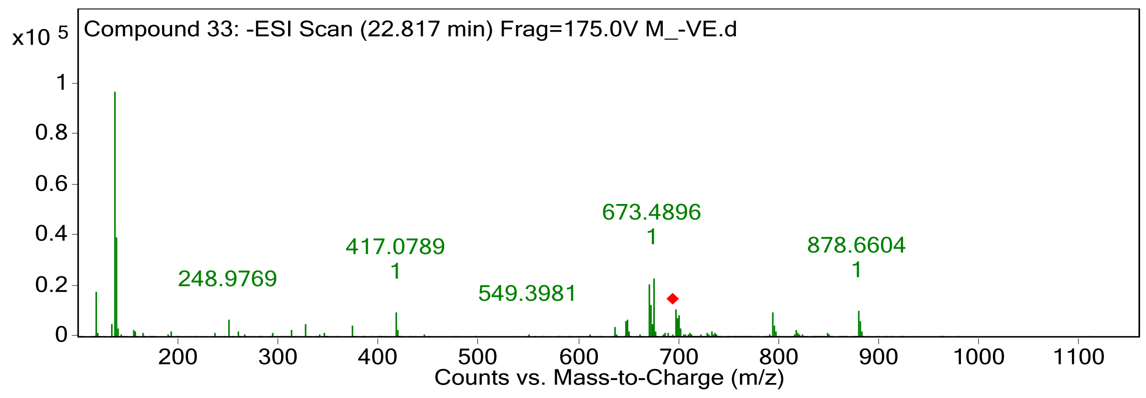
x10 5
Compound 33: -ESI Scan (22.817 min) Frag=175.0V M_-VE.d
673.4896
1
417.0789
1
878.6604
1
248.9769
549.3981
Counts vs. Mass-to-Charge (m/z)

MS Zoomed Spectrum

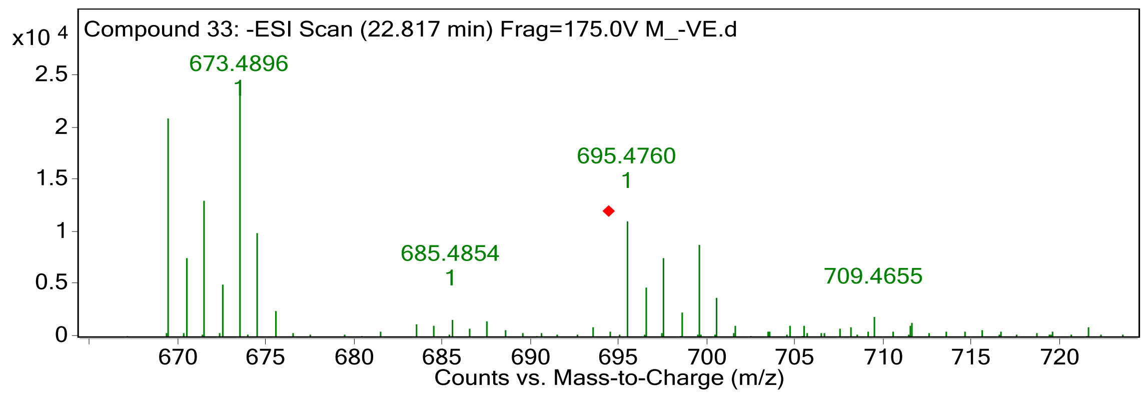

MS Spectrum Peak List

m/z	z	Abund
116.931	1	17854.47
134.8973	1	97170.83
135.8974	1	12817.05
136.8944	1	39606.31
669.4585	1	20877.97
671.4732	1	12990
673.4896	1	23500.88
694.4656	2	605.93
694.9715	2	234
695.476	1	11088.64

MSMS Spectrum

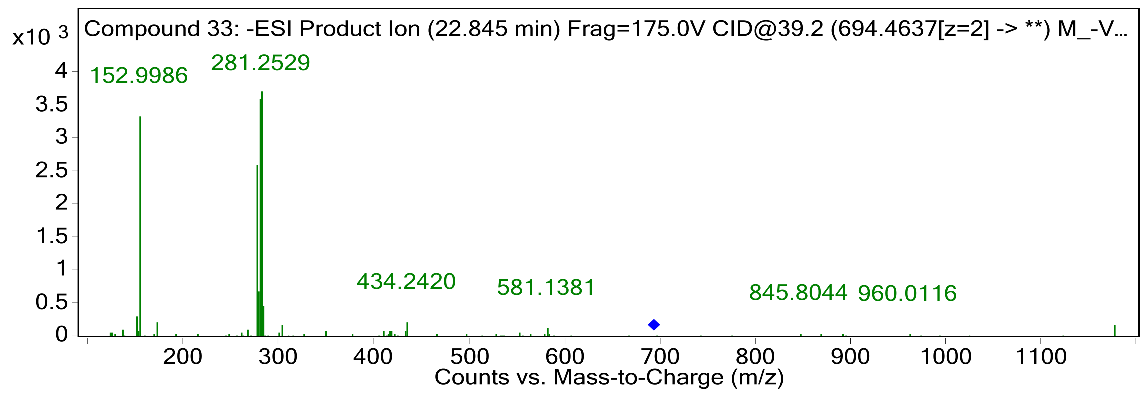

MS/MS Spectrum Peak List

m/z	z	Abund
150.9824		301.95
152.9986	1	3346.54
277.2228	1	2601.03
278.2242	1	685.74
279.2376	1	3604.2
280.2359	1	488.92
281.2298	1	248.01
281.2529	1	3722.03
282.256	1	476.81
283.2689		421.38

--- End Of Report ---
