# Supplementary material for: Metabolomic Profiling of Leptadenia reticulata: Unveiling Therapeutic Potential for Inflammatory Diseases through Network Pharmacology and Docking Studies
Source: Pharmaceuticals (Basel). 2024 Mar 26;17(4):423. doi: 10.3390/ph17040423 (PMC11054655; doi:10.3390/ph17040423)
Supplement: Supplementary file 1 [file pharmaceuticals-17-00423-s001.zip › HR LCMS chromatogram/M_CompoundReport.pdf]

Qualitative Compound Report

**Data File**	M.d	**Sample Name**	M
**Sample Type**	Sample	**Position**	P1-A6
**Instrument Name**	QTOF	**User Name**	
**Acq Method**	metabolite_ESI_+VE_MSMS.m	**Acquired Time**	5/7/2023 7:10:20 PM
**IRM Calibration Status**	Success	**DA Method**	default.m
**Comment**			

Sample Group **Info.**

Acquisition SW Version 6200 series TOF/6500 series Q-TOF B.05.01 (B5125.3)

Compound Table

Compound Label	RT	Mass	Abund	Name	Formula	MFG Formula	DB Formula	DB Diff (ppm)	Hits (DB)
Cpd 1: Brassilexin; C9 H6 N2 S	1.516	174.025	13602	Brassilexin	C9 H6 N2 S	C9 H6 N2 S	C9 H6 N2 S	0.73	3
Cpd 2: 2-Methyl-1,3-dithiolane; C4 H8 S2	1.551	120.0086	19961	2-Methyl-1,3-dithiolane	C4 H8 S2	C4 H8 S2	C4 H8 S2	-15.31	7
Compound 3	1.59								
Cpd 4: Lansiumamide C; C18 H19 N O	1.628	265.1466		Lansiumamide C	C18 H19 N O	C18 H19 N O	C18 H19 N O	0.29	5
Compound 5	2.082		10090						
Compound 6	2.6		16197						
Compound 7	2.88		13766						
Cpd 8: Methyl N-methylanthranilate; C9 H11 N [illegible]	3.929	165.0773		Methyl N-methylanthranilate	C9 H11 N O2	C9 H11 N O2	C9 H11 N O2	10.38	10
Cpd 9: D6-Ambrettolide; C16 H28 O2	10.657	252.2052		D6-Ambrettolide	C16 H28 O2	C16 H28 O2	C16 H28 O2	14.92	9
Cpd 10: D6-Ambrettolide; C16 H28 O2	10.938	252.2054		D6-Ambrettolide	C16 H28 O2	C16 H28 O2	C16 H28 O2	13.85	9
Cpd 11: Vinpocetine; C22 H26 N2 O2	11.532	350.1991		Vinpocetine	C22 H26 N2 O2	C22 H26 N2 O2	C22 H26 N2 O2	0.95	6
Cpd 12: Azatadine; C20 H22 N2	12.391	290.182		Azatadine	C20 H22 N2	C20 H22 N2	C20 H22 N2	-12.75	1
Compound 13	12.533		12572						
Cpd 14: Azatadine; C20 H22 N2	12.704	290.1816	10764	Azatadine	C20 H22 N2	C20 H22 N2	C20 H22 N2	-11.24	1
Cpd 15: 16-Oxo-palmitate; C16 H30 O3	13.115	270.2158	12397	16-Oxo-palmitate	C16 H30 O3	C16 H30 O3	C16 H30 O3	13.73	2
Cpd 16: Dimethindene; C20 H24 N2	13.413	292.1971	10519	Dimethindene	C20 H24 N2	C20 H24 N2	C20 H24 N2	-10.77	2
Cpd 17: D6-Ambrettolide; C16 H28 O2	13.907	252.2057		D6-Ambrettolide	C16 H28 O2	C16 H28 O2	C16 H28 O2	12.76	9
Cpd 18: Dimethindene; C20 H24 N2	13.983	292.1973	10544	Dimethindene	C20 H24 N2	C20 H24 N2	C20 H24 N2	-11.48	2
Cpd 19: D6-Ambrettolide; C16 H28 O2	14.193	252.2056		D6-Ambrettolide	C16 H28 O2	C16 H28 O2	C16 H28 O2	13.37	9
Cpd 20: (E)-3-Hexadecenoic acid; C16 H30 O2	15.385	254.2209		(E)-3-Hexadecenoic acid	C16 H30 O2	C16 H30 O2	C16 H30 O2	14.32	8
Cpd 21: (E)-3-Hexadecenoic acid; C16 H30 O2	15.765	254.2209		(E)-3-Hexadecenoic acid	C16 H30 O2	C16 H30 O2	C16 H30 O2	14.53	8
Cpd 22: (E)-3-Hexadecenoic acid; C16 H30 O2	16.166	254.221	59767	(E)-3-Hexadecenoic acid	C16 H30 O2	C16 H30 O2	C16 H30 O2	13.99	8
Cpd 23: Dodecyl butyrate; C16 H32 O2	16.542	256.2366	14428	Dodecyl butyrate	C16 H32 O2	C16 H32 O2	C16 H32 O2	14	8
Cpd 24: (E)-3-Hexadecenoic acid; C16 H30 O2	16.898	254.221		(E)-3-Hexadecenoic acid	C16 H30 O2	C16 H30 O2	C16 H30 O2	14.17	8
Cpd 25: (E)-3-Hexadecenoic acid; C16 H30 O2	17.153	254.2211	15755	(E)-3-Hexadecenoic acid	C16 H30 O2	C16 H30 O2	C16 H30 O2	13.85	8
Cpd 26: Methyl 2-furoate; C6 H6 O3	17.297	126.0302	24892	Methyl 2-furoate	C6 H6 O3	C6 H6 O3	C6 H6 O3	12.1	9
Cpd 27: Methyl 2-furoate; C6 H6 O3	17.643	126.0303	21573	Methyl 2-furoate	C6 H6 O3	C6 H6 O3	C6 H6 O3	11.03	9
Cpd 28: Methyl 2-furoate; C6 H6 O3	17.99	126.0301	17518	Methyl 2-furoate	C6 H6 O3	C6 H6 O3	C6 H6 O3	12.88	9
Cpd 29: Methyl 2-furoate; C6 H6 O3	18.413	126.0303	11532	Methyl 2-furoate	C6 H6 O3	C6 H6 O3	C6 H6 O3	10.98	9
Compound 30	24.215								

Compound Label	Name	*m/z*	RT	Algorithm	Mass
Cpd 1: Brassilexin; C9 H6 N2 S	**Brassilexin**	175.0323	1.516	Auto MS/MS	174.025

MS Spectrum

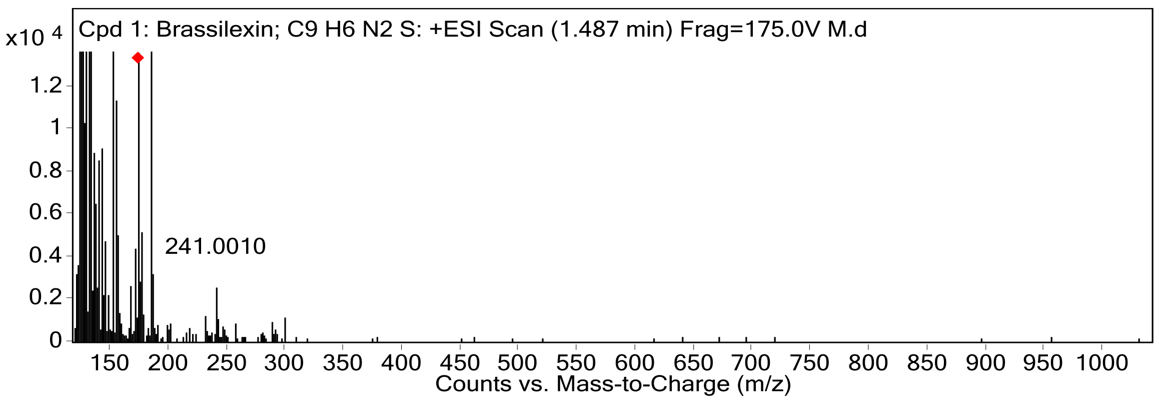

MS Zoomed Spectrum

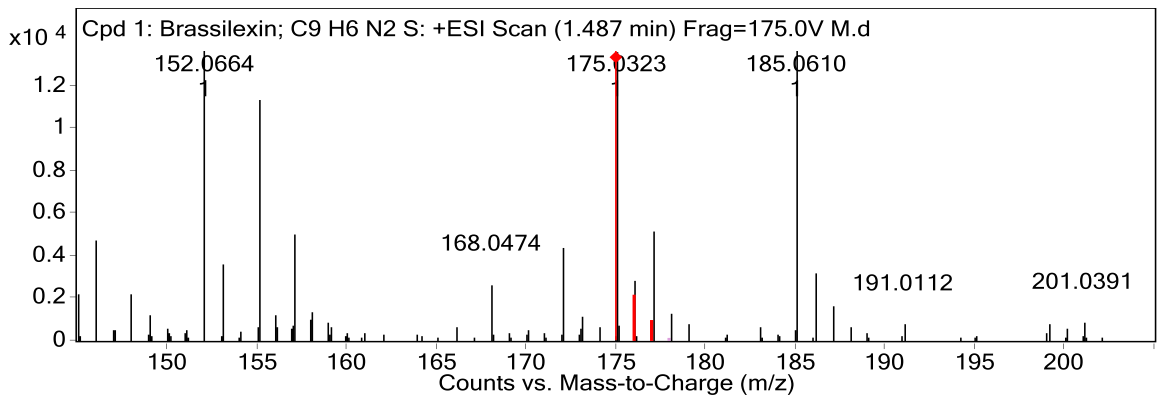

MS Spectrum Peak List

m/z	*Calc m/z*	Diff(ppm)	z	Abund	Formula	Ion
124.084				70196.3		
125.068			1	36191.43		
127.0204			1	44362.46		
129.9104			1	56473.39		
131.9077			1	57385.58		
133.9043				18195.35		
152.0664			1	33411.17		
175.0323	175.0324	0.69	1	13602.15	C9 H6 N2 S	(M+H)+
176.0345	176.0351	3.64	1	2845.49	C9 H6 N2 S	(M+H)+
177.0295	177.0293	-0.83	1	5177.38	C9 H6 N2 S	(M+H)+

MSMS Spectrum

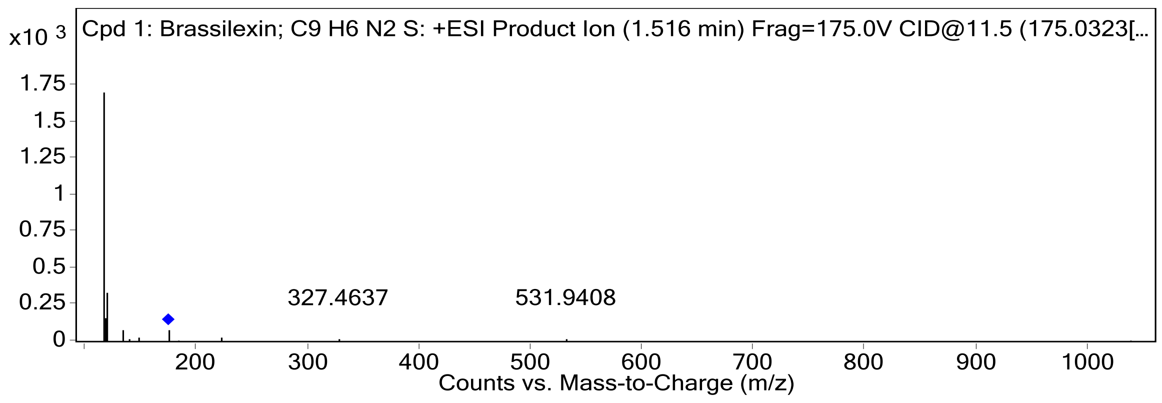

MS/MS Spectrum Peak List

m/z	z	Abund
116.8448		138.09
116.9722		112
116.992	1	1701.1
117.0338		128.28
117.1991		29.13
117.9929	1	163.06
118.9895		336.8
133.9718		82
148.1128		33.47
175.0333		81.85

Compound Structure

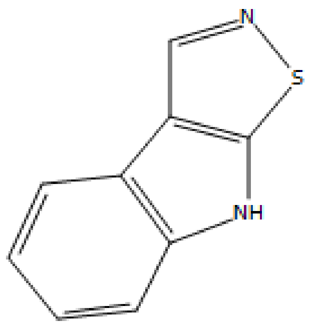

Compound Label	Name	*m/z*	RT	Algorithm	Mass
Cpd 2: 2-Methyl-1,3-dithiolane; C4 H8 S2	**2-Methyl-1,3-dithiolane**	142.9978	1.551	Auto MS/MS	120.0086

MS Spectrum

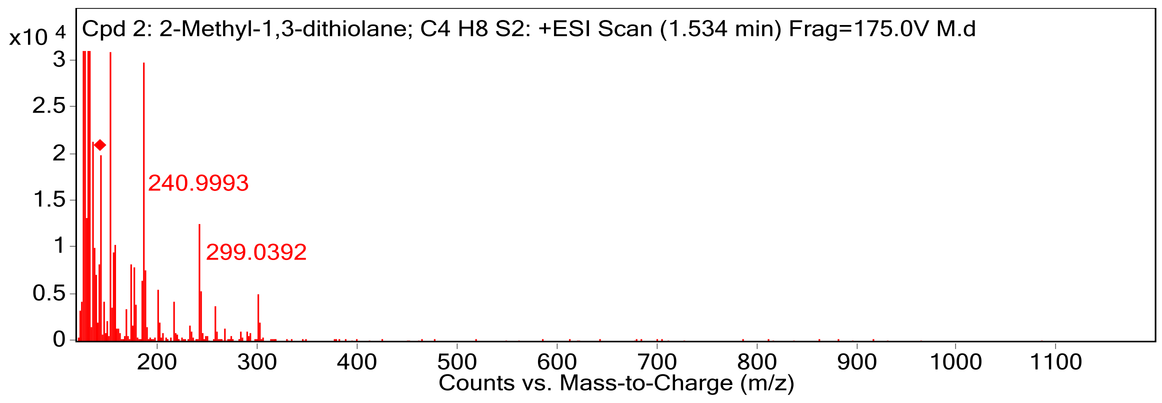

MS Zoomed Spectrum

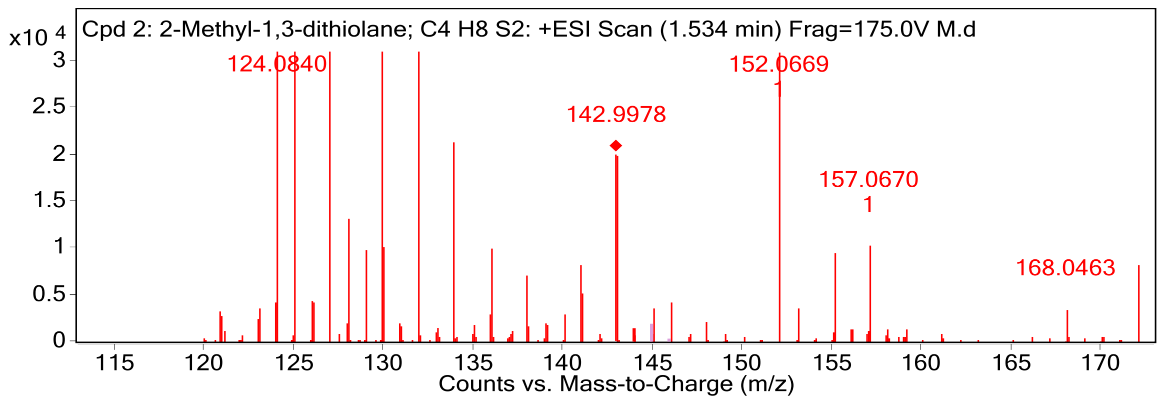

MS Spectrum Peak List

m/z	*Calc m/z*	Diff(ppm)	z	Abund	Formula	Ion
124.084				71064.01		

125.068			1	43598.94		
127.0206			1	70471.74		
129.9106			1	60751.57		
131.9078			1	56781.73		
133.9045				21425.2		
142.9978	142.996	-12.55		19961.33	C4 H8 S2	(M+Na)+
144.0013	143.9983	-20.99	1	725.63	C4 H8 S2	(M+Na)+
152.0669			1	30957.5		
185.0613			1	29898.19		

MSMS Spectrum

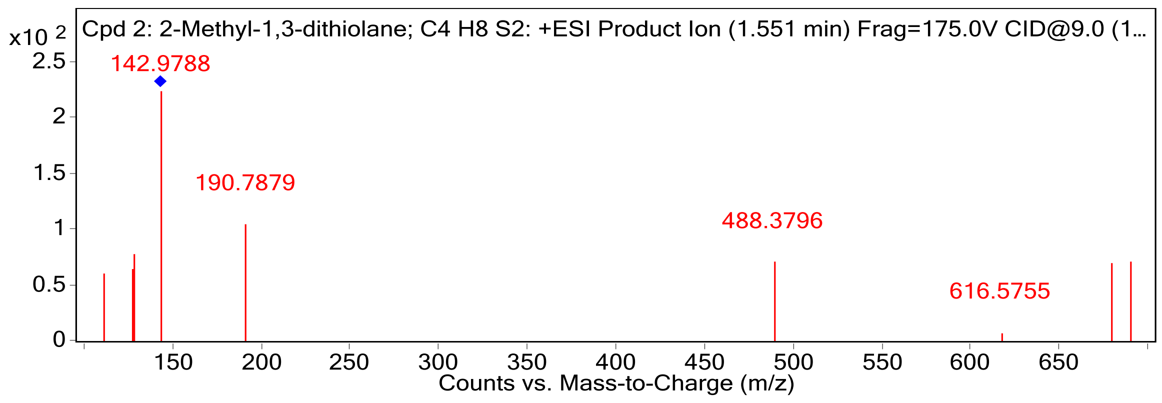

MS/MS Spectrum Peak List

m/z	Abund
111.0405	61.3
127.0193	65.34
128.0565	78.22
142.9788	224.35
142.9992	120.11
190.7879	105.48
488.3796	71.45
616.5755	8.35
678.6875	71.02
689.0159	72

Compound Structure

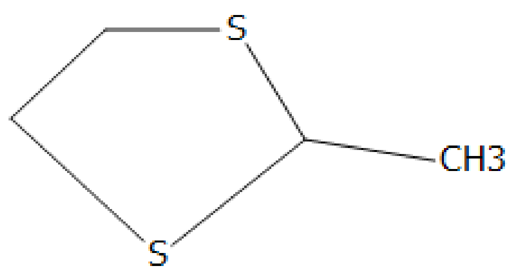

Compound Label	*m/z*	RT	Algorithm
Compound 3	198.9359	1.59	Auto MS/MS

MS Spectrum

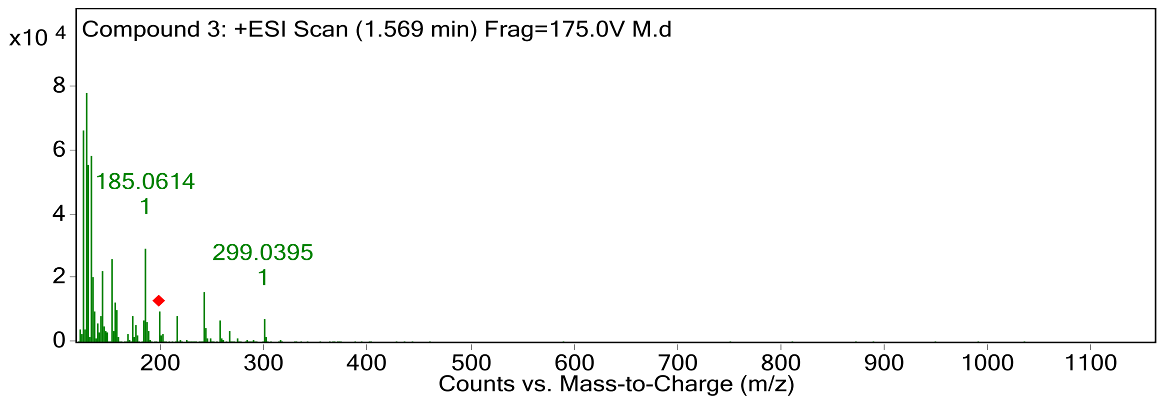

MS Zoomed Spectrum

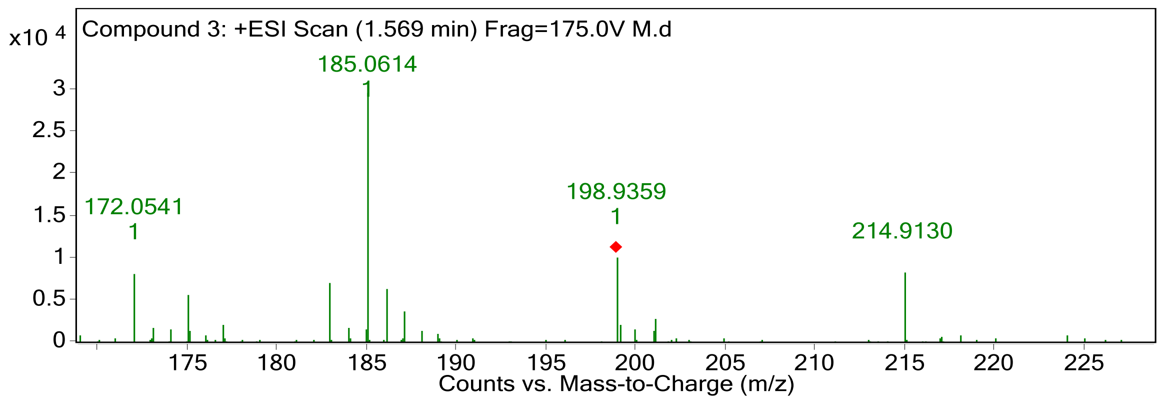

MS Spectrum Peak List

m/z	z	Abund
124.084		66759.42
125.0679	1	42154.89
127.0207	1	78432.02
129.9104	1	55623.93
131.9077	1	58399.28
152.0671	1	26031.51
185.0614	1	29709.51
198.9359	1	10037.33
199.9357	1	1596.64
200.9333	1	1445.1

MSMS Spectrum

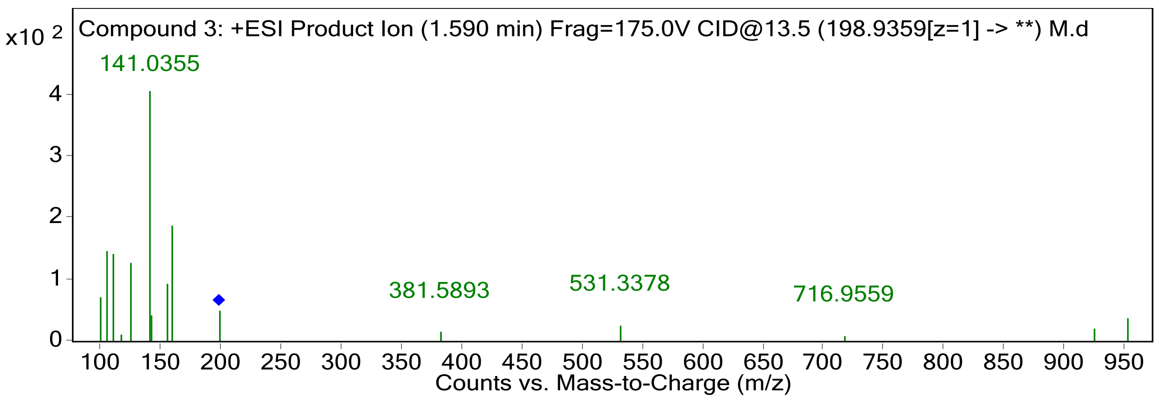

MS/MS Spectrum Peak List

m/z	Abund
100.0709	72.2
104.936	147.89
111.0016	142.15
124.9353	127.21
141.0355	405.62
141.9685	43.52
154.9597	94.07
159.0502	188.9
198.9346	49.92
950.835	38

Compound Label	Name	*m/z*	RT	Algorithm	Mass
Cpd 4: Lansiumamide C; C18 H19 N O	**Lansiumamide C**	266.1539	1.628	Auto MS/MS	265.1466

MS Spectrum

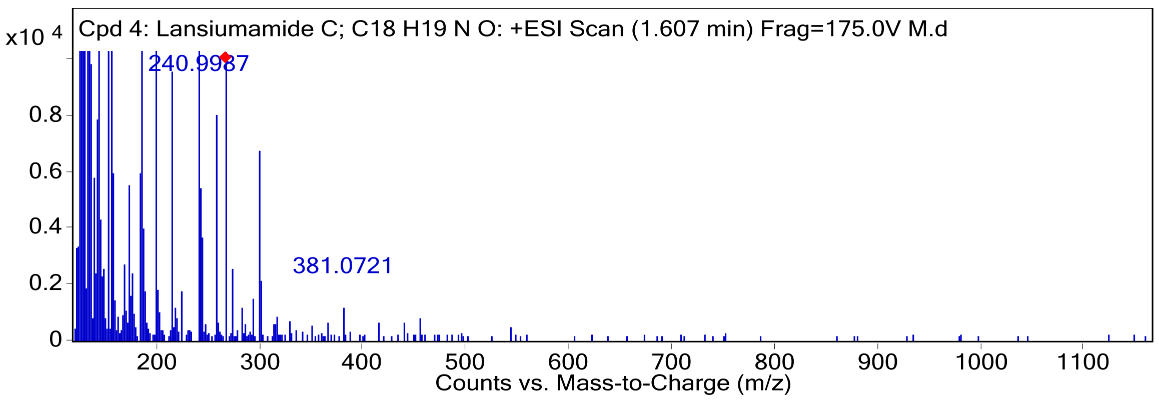

MS Zoomed Spectrum

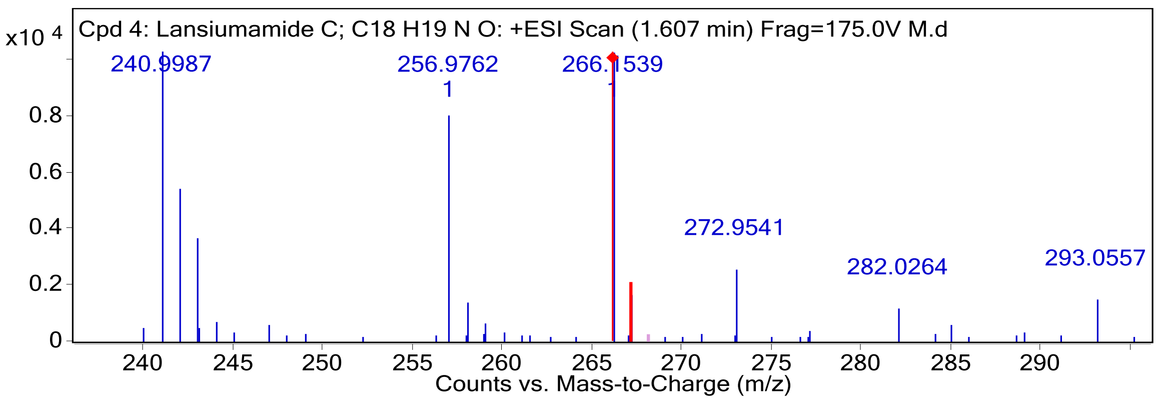

MS Spectrum Peak List

m/z	*Calc m/z*	Diff(ppm)	z	Abund	Formula	Ion
124.0838				71717.76		
125.0679			1	41102.53		
127.021			1	58485.88		
129.9104			1	54825.15		
131.9076			1	60250.94		
133.9046				20364.38		
152.0671			1	26807.25		
185.0609			1	24571.55		
266.1539	266.1539	0.33	1	10255.72	C18 H19 N O	(M+H)+
267.1573	267.1572	-0.28	1	1672.32	C18 H19 N O	(M+H)+

MSMS Spectrum

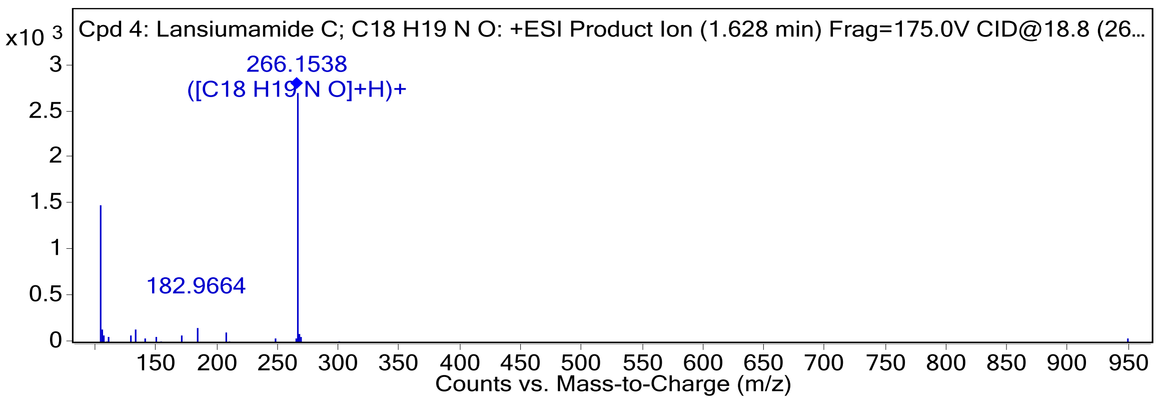

MS/MS Spectrum Peak List

m/z	*Calc m/z*	Diff (ppm)	z	Abund	Formula	Ion
104.0866				173.4		
104.1047			1	1483.64		
105.1086			1	144.01		
133.0512				145.02		
182.9664				163.82		
206.9962				118.66		
265.6257				109.48		
265.983				103.04		
266.1538	266.1539	0.65		2707.04	C18 H19 N O	(M+H)+
267.104				95.48		

Compound Structure

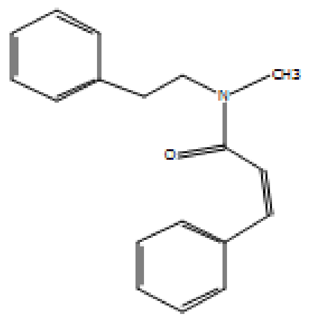

Compound Label	*m/z*	RT	Algorithm
Compound 5	144.0982	2.082	Auto MS/MS

MS Spectrum

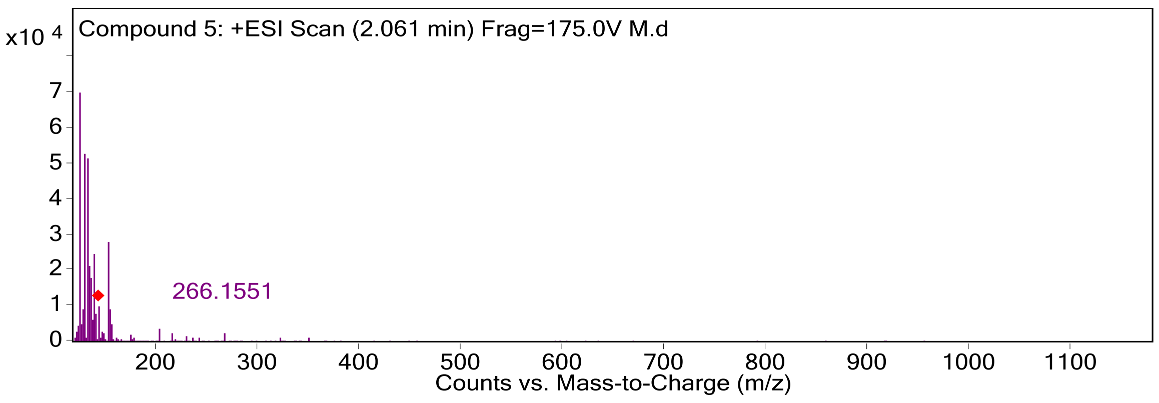

MS Zoomed Spectrum

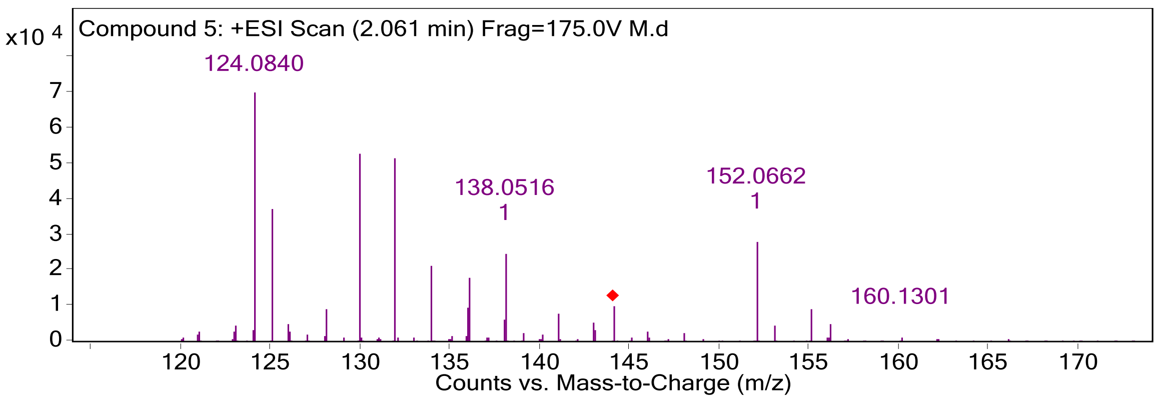

MS Spectrum Peak List

m/z	z	Abund
124.084		70196.94
125.0682	1	37492.88
129.9105	1	52918.42
131.9074	1	51747.71
133.9045	1	21361.39
138.0516	1	24705.07
144.0982	1	10090.04
145.1018	1	1454.68
146.0879	1	1231.72
152.0662	1	27936.88

MSMS Spectrum

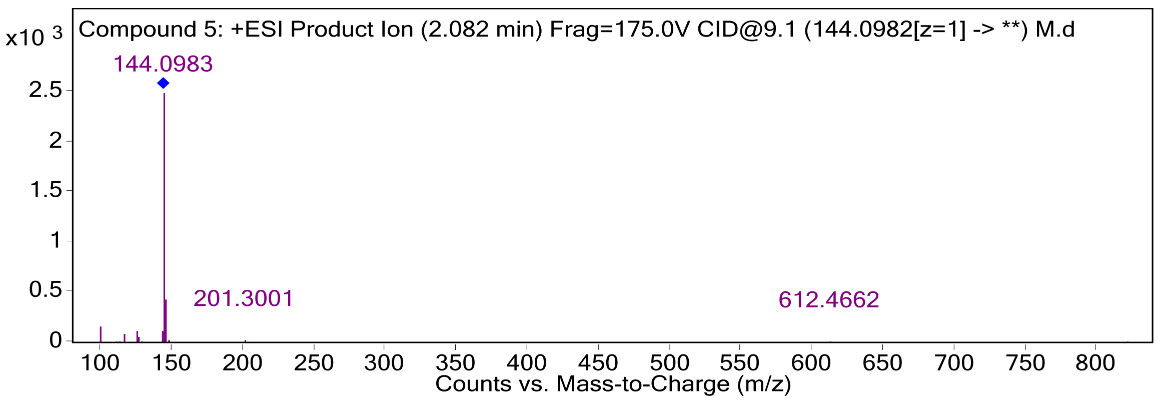

MS/MS Spectrum Peak List

m/z	z	Abund
100.0313		162.03
117.0664		89
126.0871		113.93
127.02		55.54
143.0788		119.32
144.0693	2	222.84
144.0983	1	2492.69
144.578	2	44.04
145.099	1	184.99
145.9637		433.91

Compound Label	*m/z*	RT	Algorithm
Compound 6	120.0781	2.6	Auto MS/MS

MS Spectrum

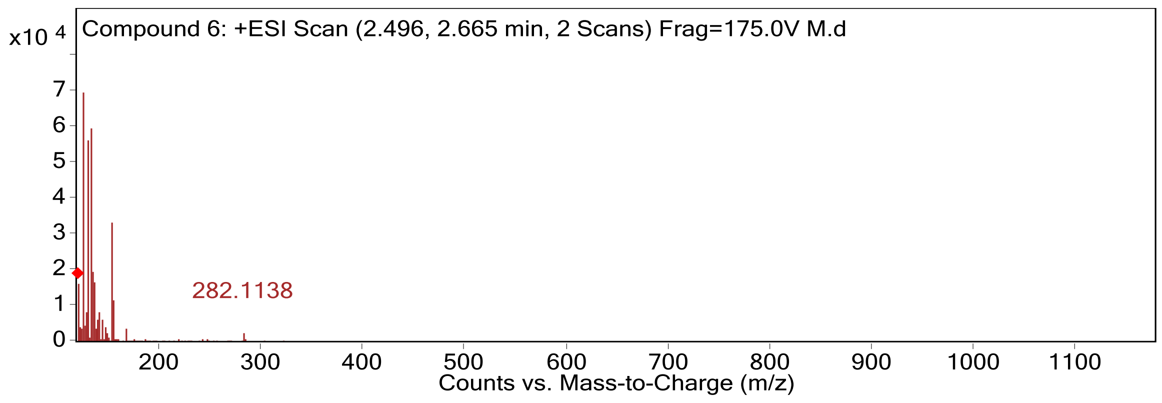

MS Zoomed Spectrum

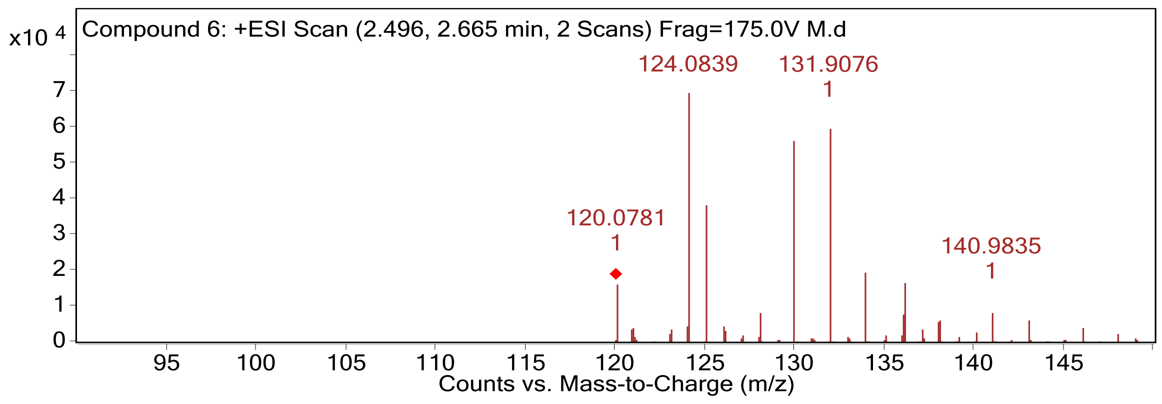

MS Spectrum Peak List

m/z	z	Abund
120.0781	1	16197.4
121.0808	1	1807.04
124.0839		69559.41
125.0681	1	38290.04
129.9103	1	56051.52
131.9076	1	59644.12
133.9044		19690.3
136.0583		16573.89
152.0529		11893.94
152.0659	1	33430.43

MSMS Spectrum

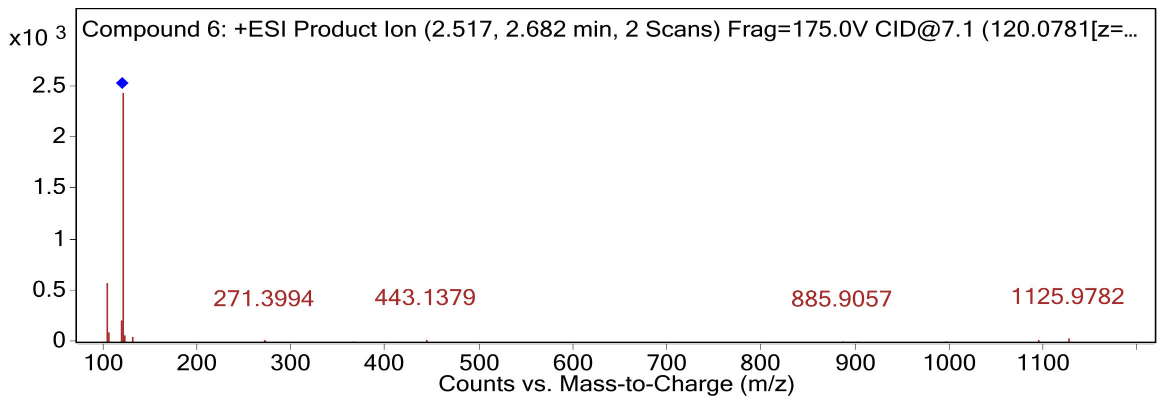

MS/MS Spectrum Peak List

m/z	z	Abund
103.0519		578.68
104.0548		170.19
105.066		99.43
119.0335		90.74
119.0816		213.96
120.0785	1	2443.57
120.8965		744.13
120.957		333.66
121.0827	1	475.15
121.0964	2	189.33

Compound Label	*m/z*	RT	Algorithm
Compound 7	120.078	2.88	Auto MS/MS

MS Spectrum

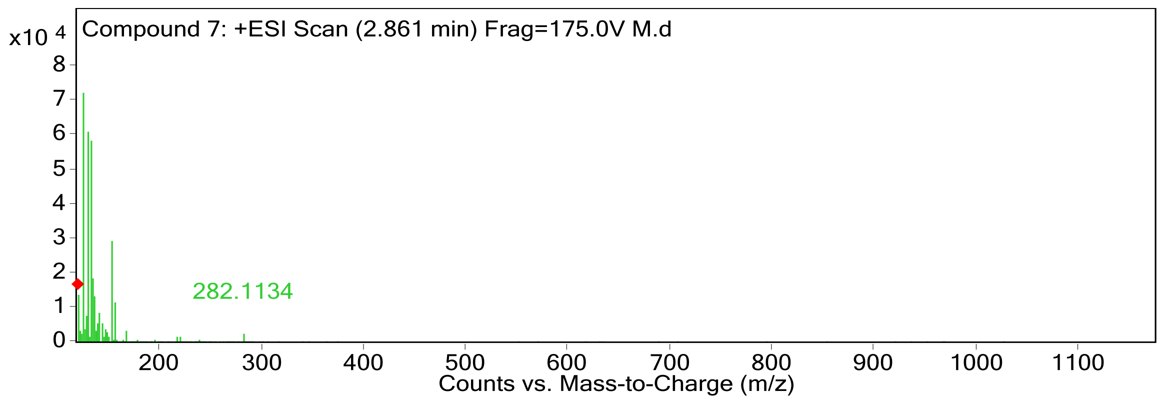

MS Zoomed Spectrum

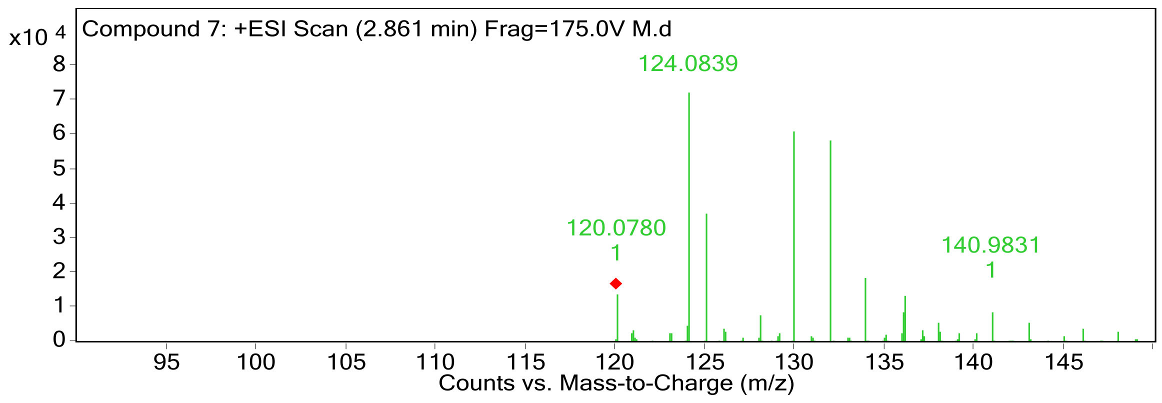

MS Spectrum Peak List

m/z	z	Abund
120.078	1	13766.11

121.082	1	1311.3
124.0839		72406.02
125.0683	1	37066.64
129.9102	1	61214.56
131.9076	1	58480.39
133.9044	1	18795.14
136.0583	1	13350.12
152.0666	1	29581.13
155.0772	1	11780.1

MSMS Spectrum

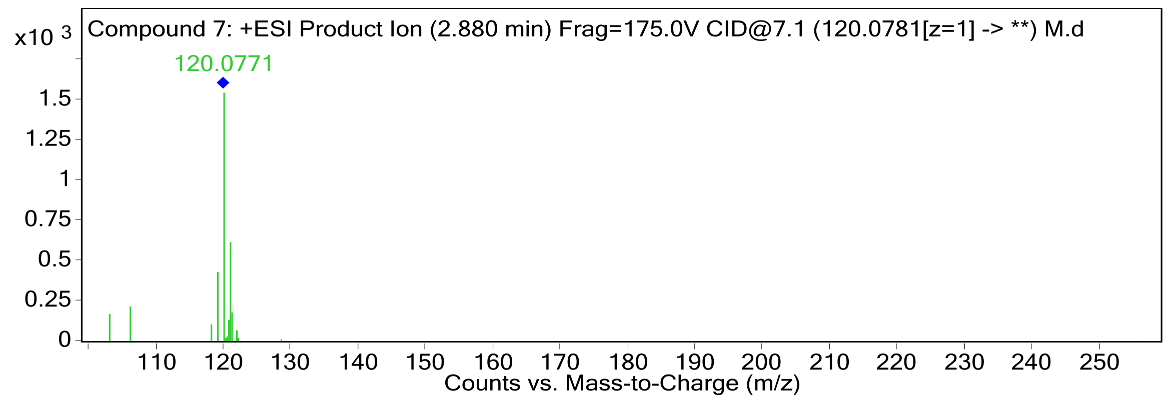

MS/MS Spectrum Peak List

m/z	z	Abund
103.0511		179.67
106.071		218.53
118.0639		108.01
119.0826		432.25
120.0771	1	1551
120.6753		135.33
120.9011		264.61
120.958	1	620.62
121.0453		144.32
121.0719	1	185.5

Compound Label	Name	*m/z*	RT	Algorithm	Mass
Cpd 8: Methyl N-methylanthranilate; C9 H11 N O2	**Methyl N-methylanthranilate**	188.0666	3.929	Auto MS/MS	165.0773

MS Spectrum

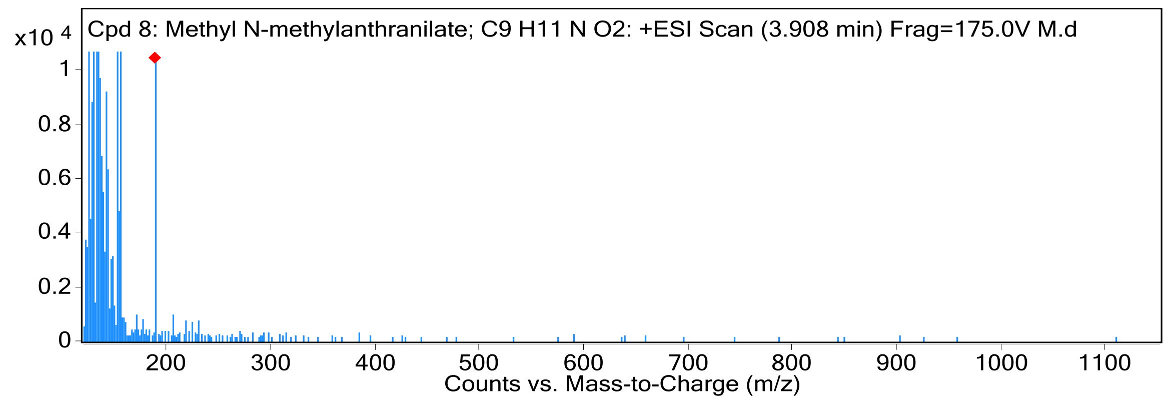

MS Zoomed Spectrum

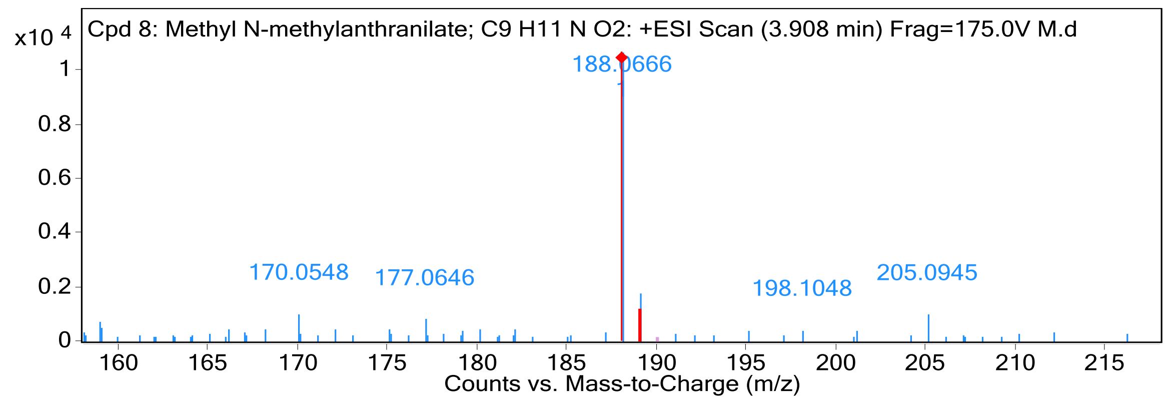

MS Spectrum Peak List

m/z	*Calc m/z*	Diff(ppm)	z	Abund	Formula	Ion
124.0839				66709.52		
125.0683			1	35646.84		
129.9103			1	56243.61		
131.9076			1	62402.02		
133.9045			1	18413.2		
135.968			1	9754.04		
152.0667			1	29229.7		
155.0776			1	12506.94		
188.0666	188.0682	8.75	1	10681.26	C9 H11 N O2	(M+Na)+
189.0692	189.0714	11.32	1	1794.66	C9 H11 N O2	(M+Na)+

MSMS Spectrum

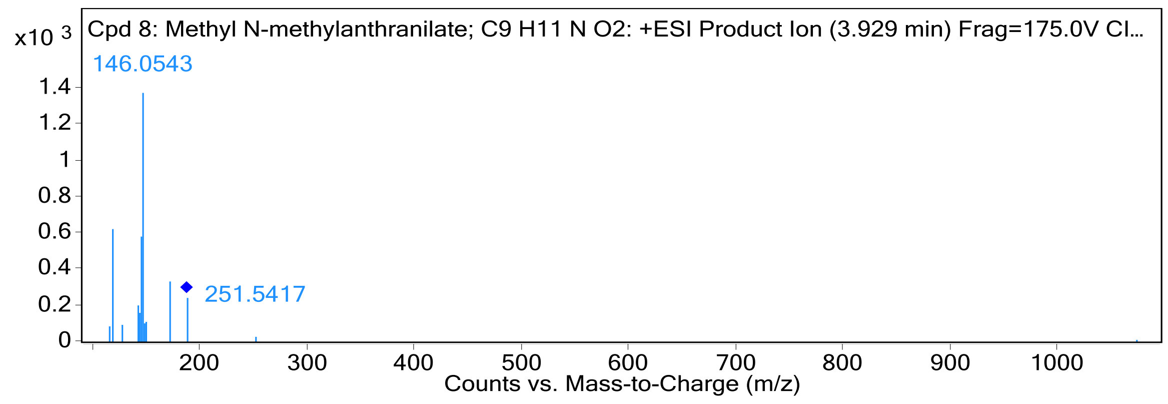

MS/MS Spectrum Peak List

m/z	z	Abund
117.0637		115.2
118.0619		625.43
118.1968		146.88

142.0781		202.45
143.0673		168.07
144.0776		583.01
146.0543	1	1379.31
148.9426		117.39
171.061		334.19
188.0656		246.46

Compound Structure

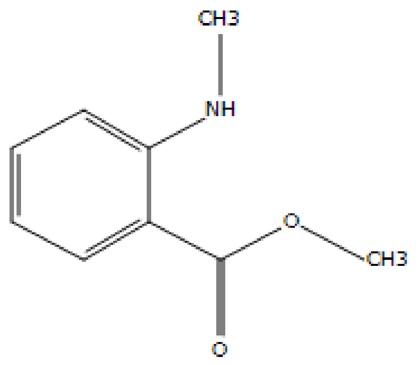

Compound Label	Name	*m/z*	RT	Algorithm	Mass
Cpd 9: D6-Ambrettolide; C16 H28 O2	**D6-Ambrettolide**	275.1943	10.657	Auto MS/MS	252.2052

MS Spectrum

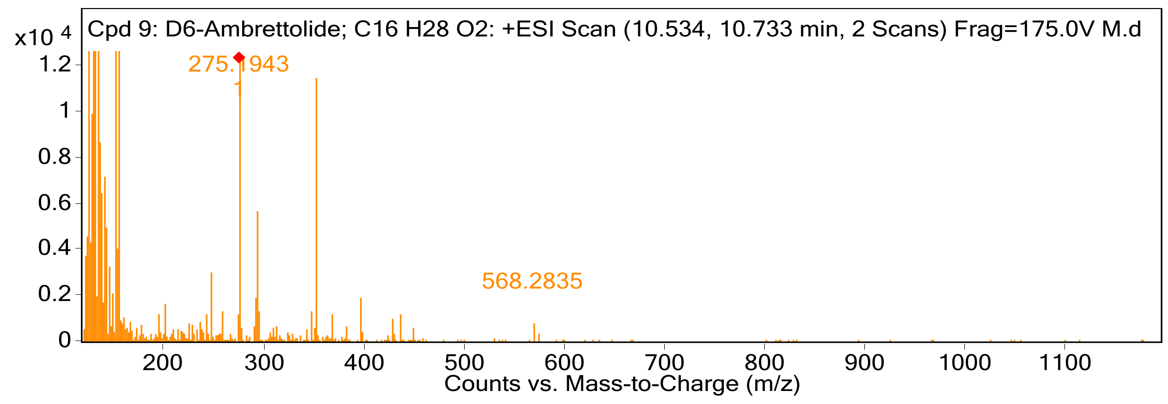

MS Zoomed Spectrum

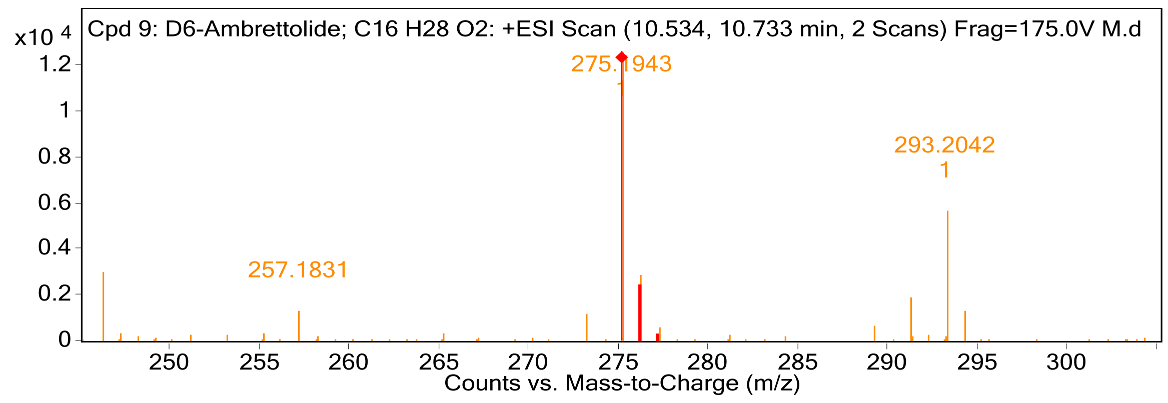

MS Spectrum Peak List

m/z	*Calc m/z*	Diff(ppm)	z	Abund	Formula	Ion
124.0839				68336.67		
125.0681			1	40030.66		
129.9105			1	61958.57		
131.9076			1	59885.3		
133.9045				21924.82		
152.0668			1	28775.83		
155.0773			1	13823.51		
275.1943	275.1982	13.9	1	12584.77	C16 H28 O2	(M+Na)+
276.1979	276.2016	13.14	1	2927.97	C16 H28 O2	(M+Na)+
277.2011	277.2044	12.09	1	639.52	C16 H28 O2	(M+Na)+

MSMS Spectrum

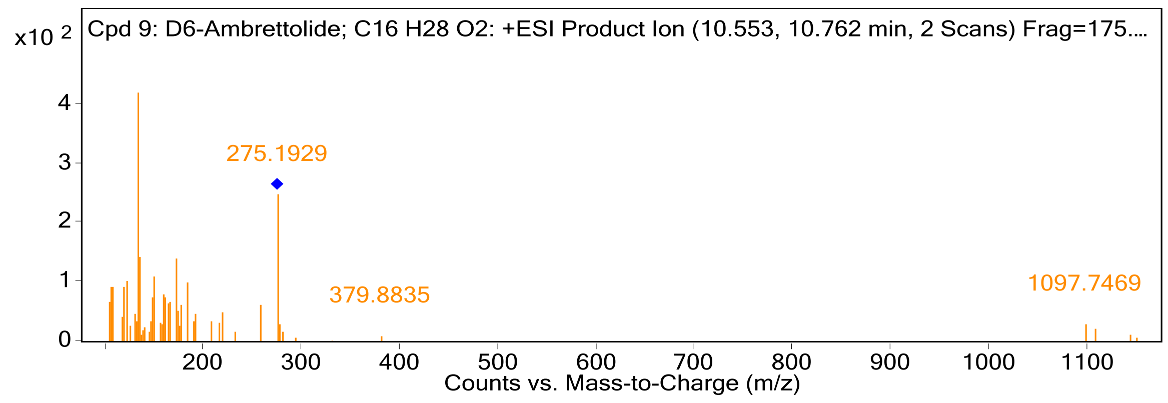

MS/MS Spectrum Peak List

m/z	Abund
105.0668	93.27
107.0753	92.74
121.0599	102.14
133.0981	420.16
135.0785	144.32
135.1118	92.59
149.0778	111.61
171.112	139.36
183.1125	99.64
275.1929	247.59

Compound Structure

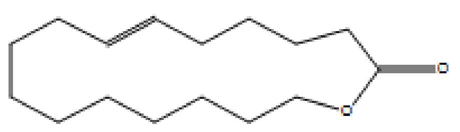

Compound Label	Name	*m/z*	RT	Algorithm	Mass
Cpd 10: D6-Ambrettolide; C16 H28 O2	**D6-Ambrettolide**	275.1947	10.938	Auto MS/MS	252.2054

MS Spectrum

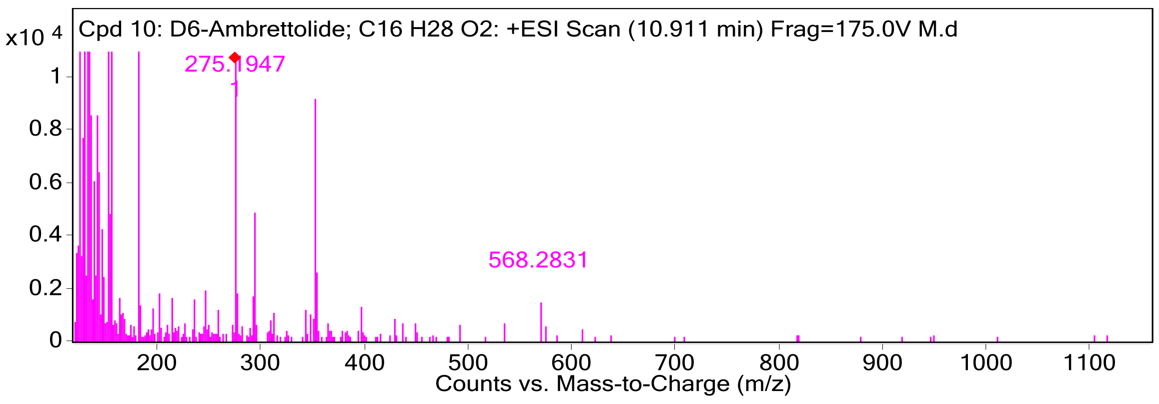

MS Zoomed Spectrum

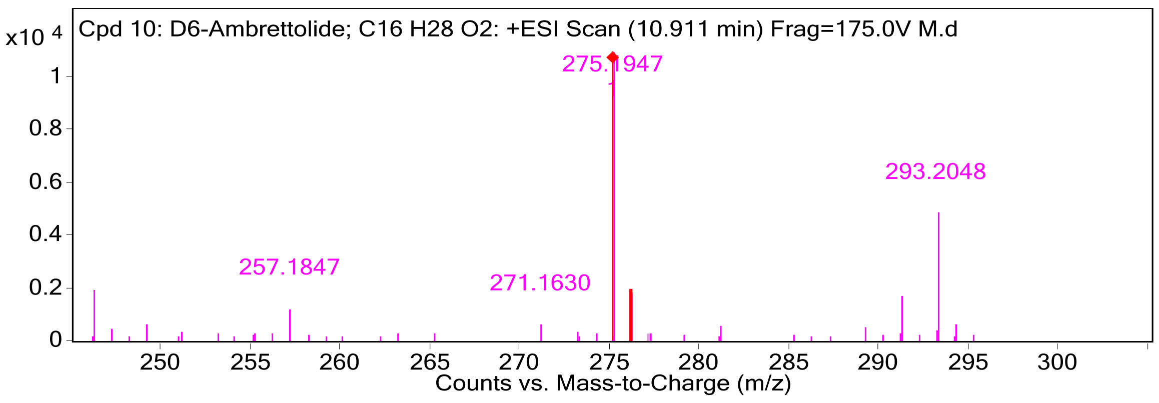

MS Spectrum Peak List

m/z	*Calc m/z*	Diff(ppm)	z	Abund	Formula	Ion
124.0839				69676.81		
125.0683			1	39935.58		
129.9107			1	60187.32		
131.9075			1	57079.88		
133.9044			1	19894.93		
152.0666			1	29035.81		
155.078				11429.11		
181.1178			1	15825.7		
275.1947	275.1982	12.62	1	10930.73	C16 H28 O2	(M+Na)+
276.198	276.2016	12.94	1	1879.78	C16 H28 O2	(M+Na)+

MSMS Spectrum

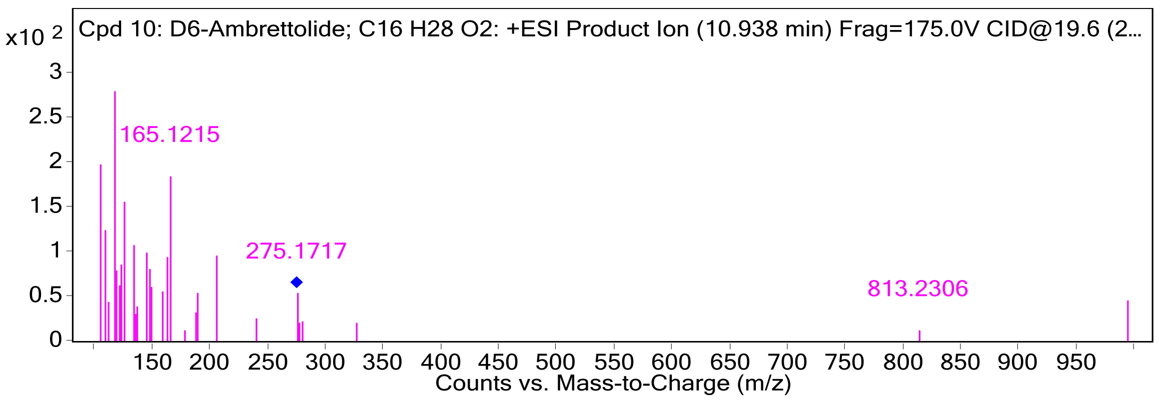

MS/MS Spectrum Peak List

m/z	Abund
105.0697	197.98
109.0957	125.08
117.0649	280.07
123.0756	87.25
125.0865	157.31
133.0703	107.53
145.0968	99.31
163.1054	95.11
165.1215	185.5
205.1915	97.19

Compound Structure

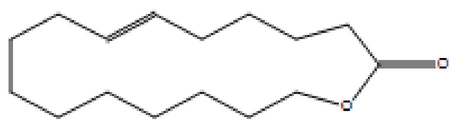

Compound Label	Name	*m/z*	RT	Algorithm	Mass

Cpd 11: Vinpocetine; C22 H26 N2 O2	**Vinpocetine**	351.2063	11.532	Auto MS/MS	350.1991

MS Spectrum

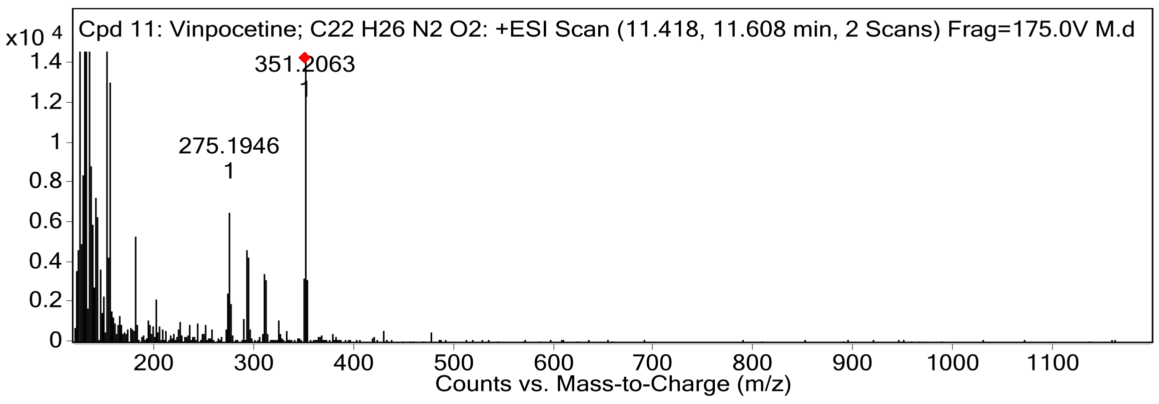

MS Zoomed Spectrum

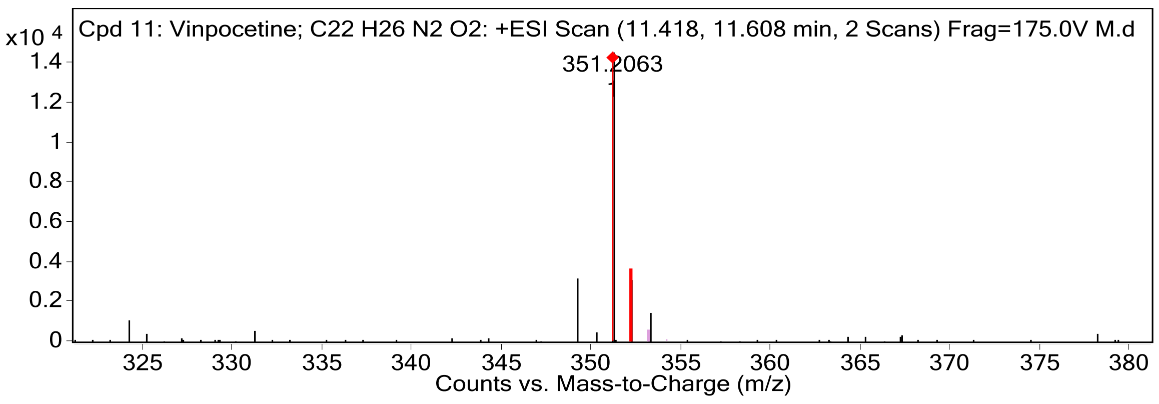

MS Spectrum Peak List

m/z	*Calc m/z*	Diff(ppm)	z	Abund	Formula	Ion
124.0839				68372.41		
125.0679			1	39993.8		
129.9103			1	59804.68		
131.9075			1	62486.45		
133.9044				22075.91		
135.9678				8821.21		
152.0668			1	29627.54		
155.0773			1	13033.21		
351.2063	351.2067	1.23	1	14559.31	C22 H26 N2 O2	(M+H)+
352.21	352.2099	-0.38	1	3186.58	C22 H26 N2 O2	(M+H)+

MSMS Spectrum

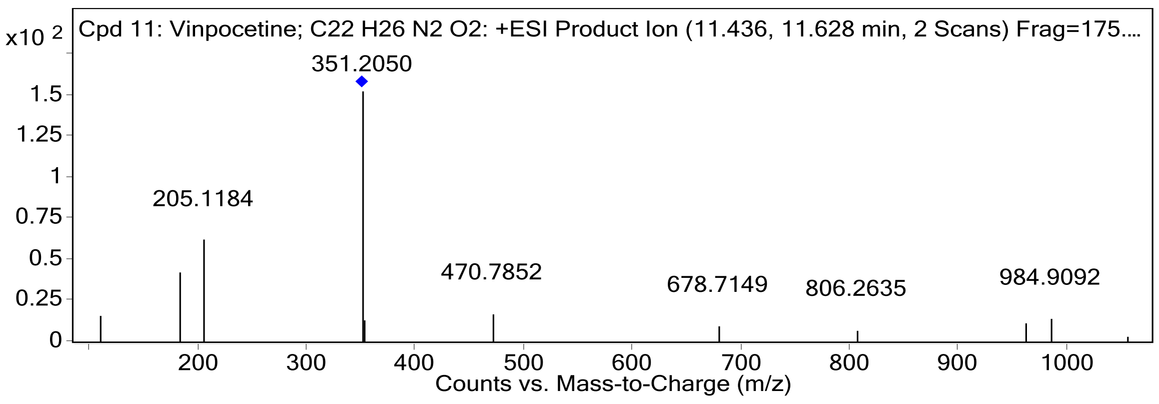

MS/MS Spectrum Peak List

m/z	z	Abund
110.0572		16.1
183.0519		42.79
205.1184		62.13
351.205	1	152.1
351.6807		20.94
352.2096	1	13.92
470.7852		17.59
678.7149		10
961.68		12
984.9092		14.4

Compound Structure

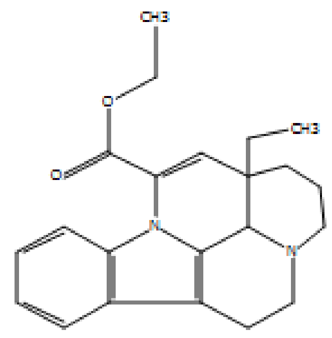

Compound Label	Name	*m/z*	RT	Algorithm	Mass
Cpd 12: Azatadine; C20 H22 N2	**Azatadine**	291.1892	12.391	Auto MS/MS	290.182

MS Spectrum

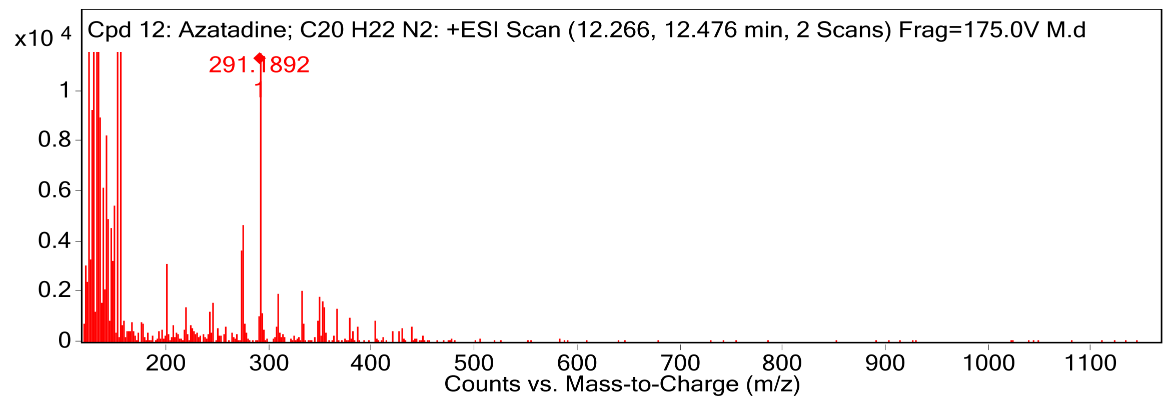

MS Zoomed Spectrum

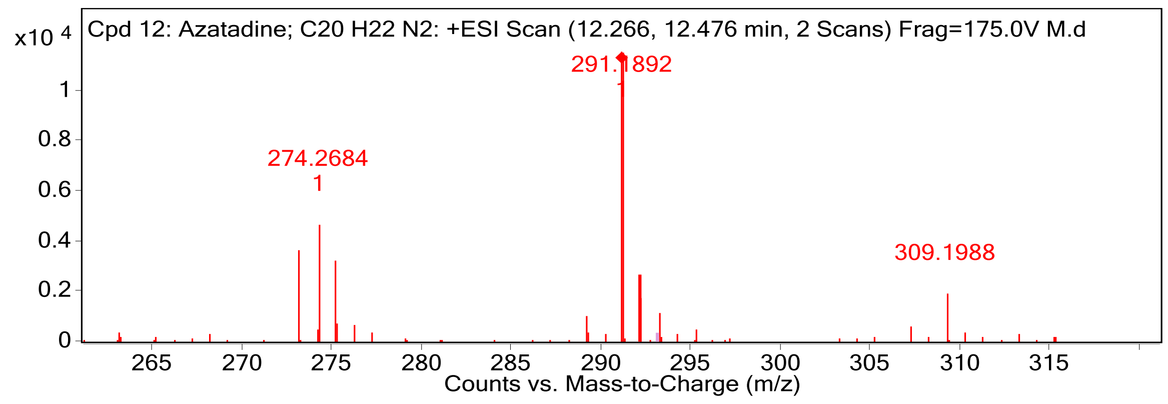

MS Spectrum Peak List

m/z	*Calc m/z*	Diff(ppm)	z	Abund	Formula	Ion
124.0838				68975.05		
125.068			1	36885.23		
128.0587			1	9242.92		
129.9103			1	60315.02		
131.9074			1	62603.88		
133.9044			1	21355.97		
152.0668			1	29087.53		
155.0774			1	12478.65		
291.1892	291.1856	-12.47	1	11525.16	C20 H22 N2	(M+H)+
292.1929	292.1888	-14.13	1	1766.24	C20 H22 N2	(M+H)+

MSMS Spectrum

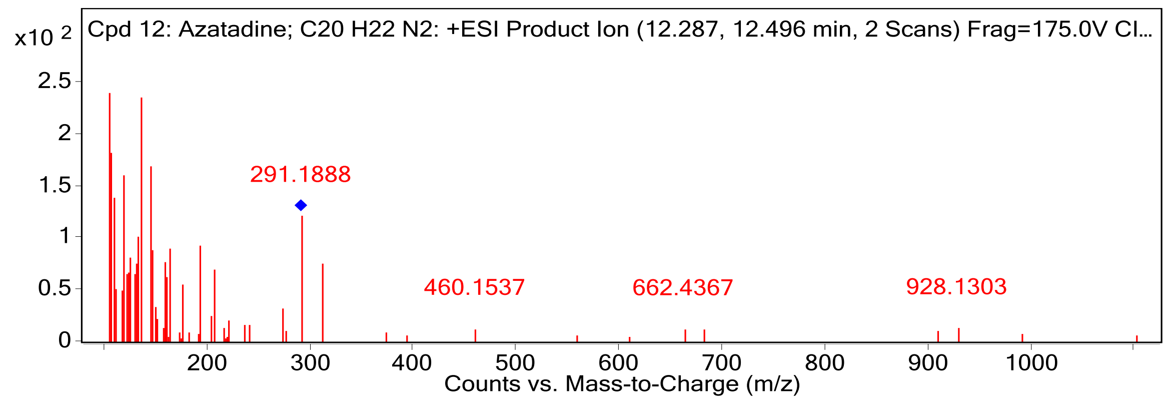

MS/MS Spectrum Peak List

m/z	z	Abund
105.0664		240.45
107.0818		182.14
109.0624		138.9
119.0808		160.73
133.0505		101.5
135.077		235.74
145.0949		170.07
163.1055		91.01
192.1092		93.61
291.1888	2	121.88

Compound Structure

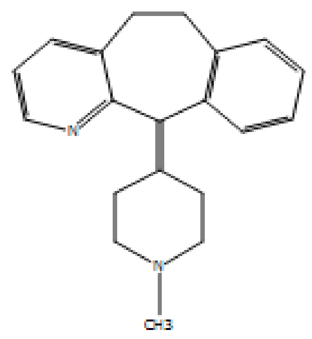

Compound Label	*m/z*	RT	Algorithm
Compound 13	274.2679	12.533	Auto MS/MS

MS Spectrum

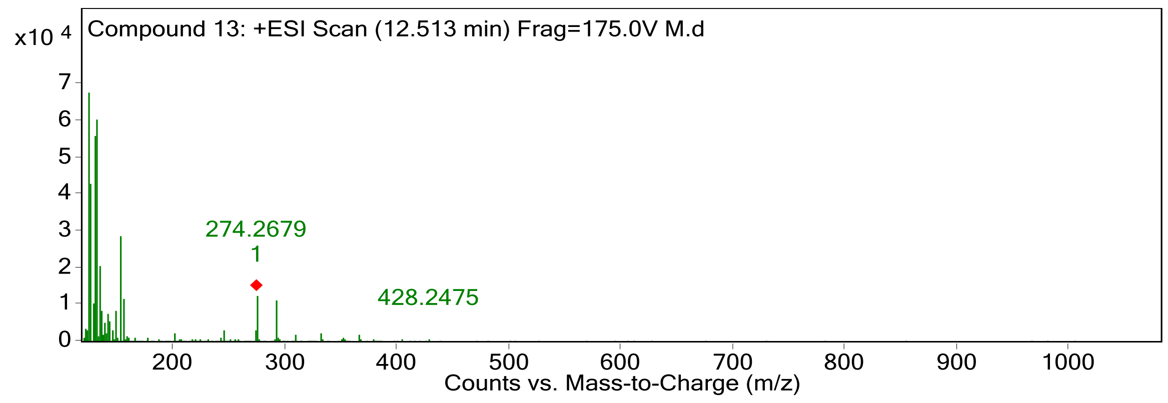

MS Zoomed Spectrum

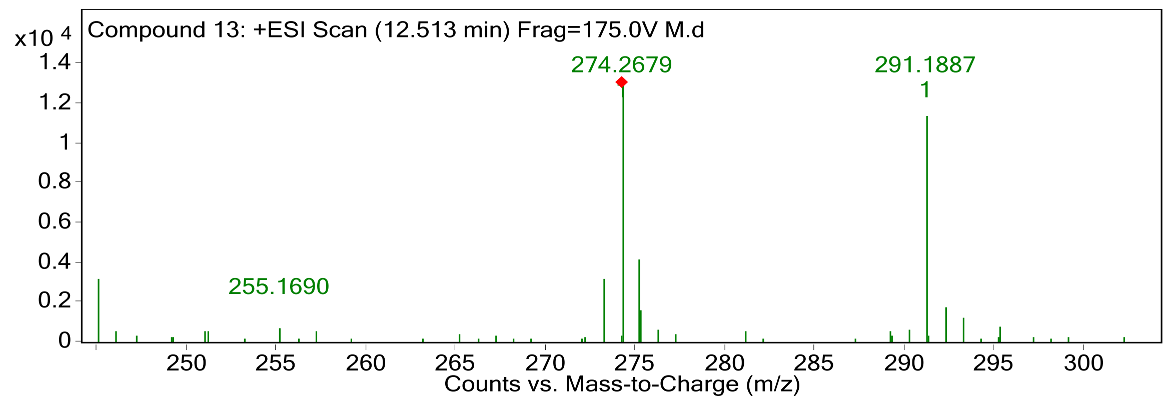

MS Spectrum Peak List

m/z	z	Abund
124.0839		67828.84
125.0679	1	42962.98
129.9105	1	56050.1
131.9075	1	60526.42
133.9045		20837.14
152.0667	1	28703.63
155.078	1	11918.59
274.2679	1	12571.64
275.272	1	1664.07
291.1887	1	11390.88

MSMS Spectrum

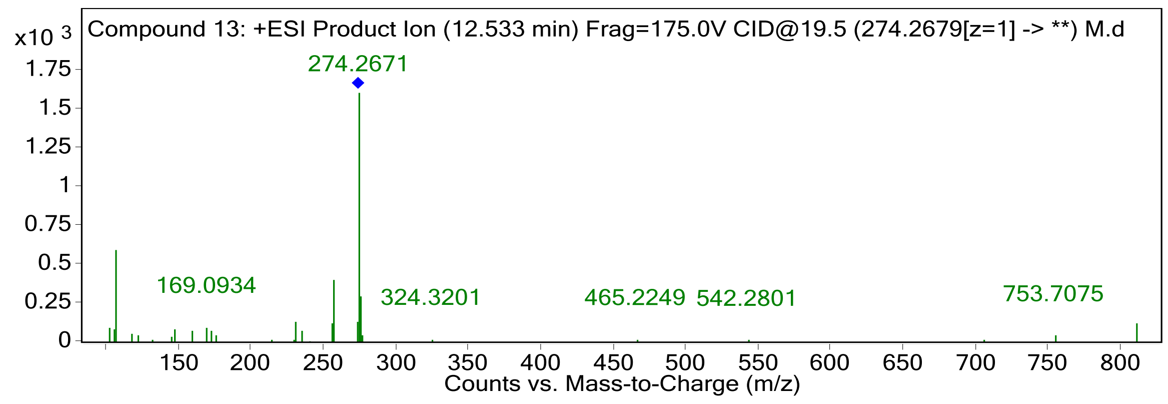

MS/MS Spectrum Peak List

m/z	z	Abund
106.083		598.82
169.0934		99.7
230.2493		133.42
255.1682		128.62
256.2567		401.75
273.1822		135.77
274.2671	1	1610.46
274.3		183
275.2704	1	298.41
809.7089		121.84

Compound Label	Name	*m/z*	RT	Algorithm	Mass
Cpd 14: Azatadine; C20 H22 N2	**Azatadine**	291.1888	12.704	Auto MS/MS	290.1816

MS Spectrum

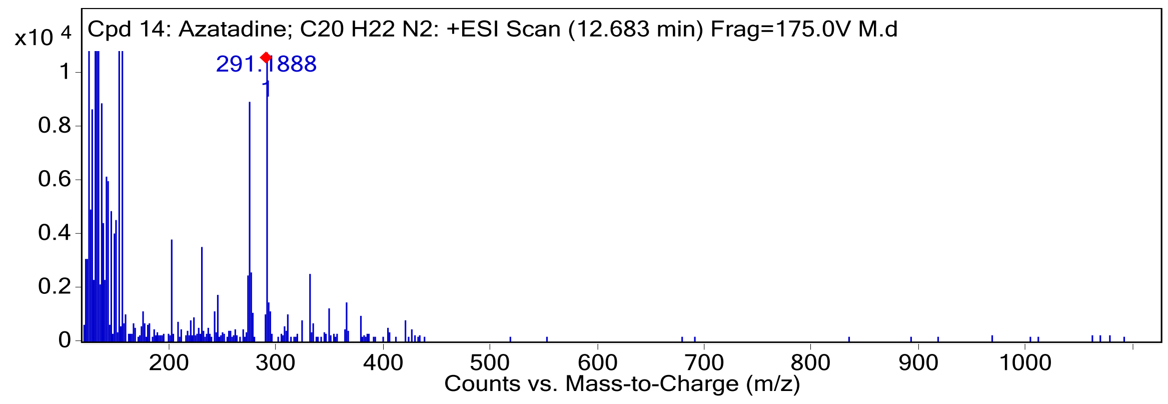

MS Zoomed Spectrum

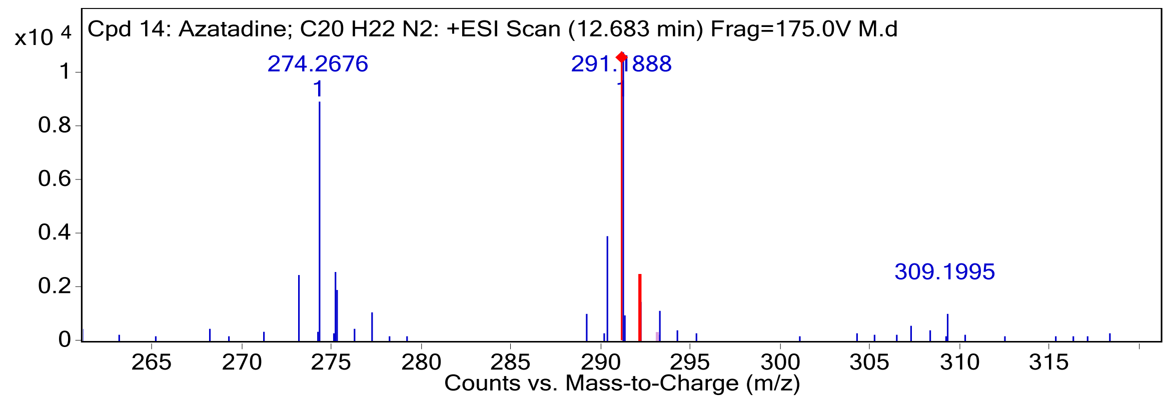

MS Spectrum Peak List

m/z	*Calc m/z*	Diff(ppm)	z	Abund	Formula	Ion
124.0837				68192.09		
125.068			1	39868.91		
129.9103			1	58797.59		
131.9076			1	64532.02		
133.9044			1	20367.7		
152.0668			1	28978.2		
155.0776			1	12499.61		
274.2676			1	8956.05		
291.1888	291.1856	-11.24	1	10764.1	C20 H22 N2	(M+H)+
292.192	292.1888	-11.06	1	1512.21	C20 H22 N2	(M+H)+

MSMS Spectrum

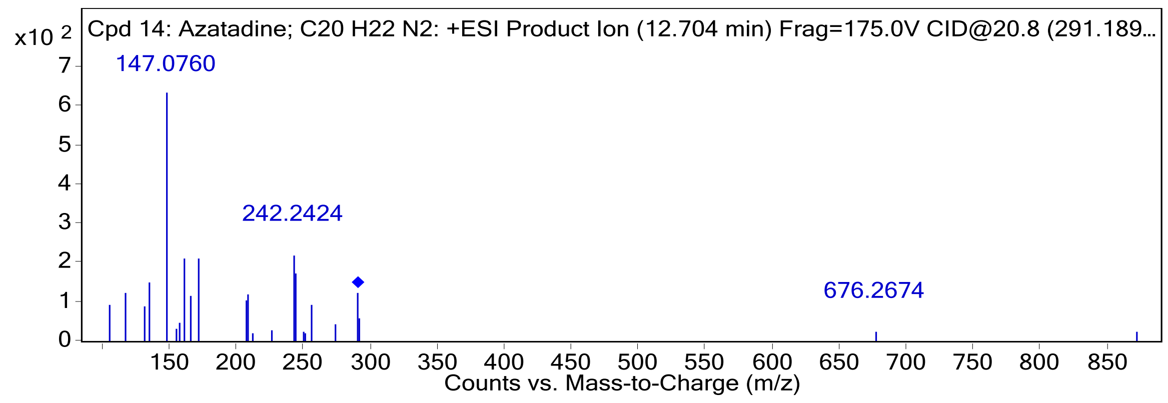

MS/MS Spectrum Peak List

m/z	Abund
117.0644	124.67
135.0786	151.04
147.076	637.62
161.0891	213.28
165.0642	119.74
171.1112	212.73
208.1179	123.34
242.2424	222.4
243.2449	174.17
290.2846	127.06

Compound Structure

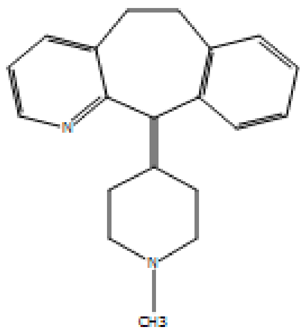

Compound Label	Name	*m/z*	RT	Algorithm	Mass
Cpd 15: 16-Oxo-palmitate; C16 H30 O3	**16-Oxo-palmitate**	293.2049	13.115	Auto MS/MS	270.2158

MS Spectrum

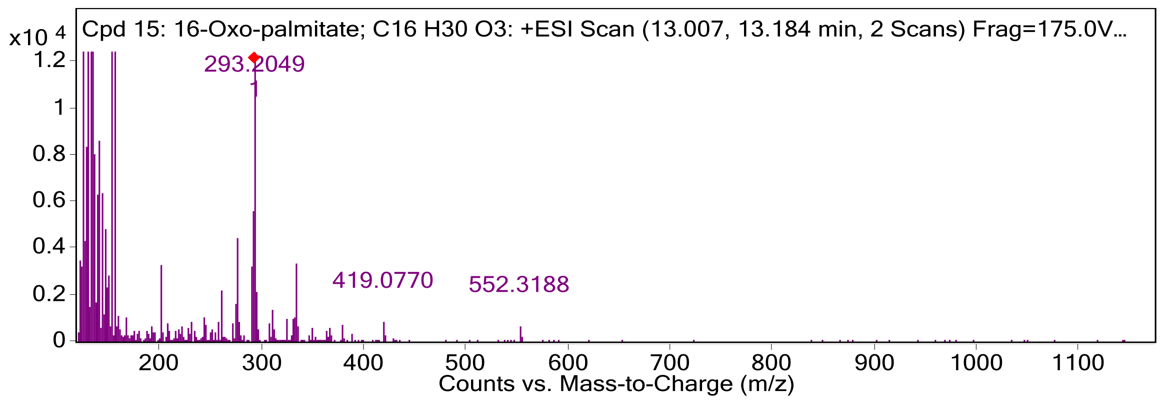

MS Zoomed Spectrum

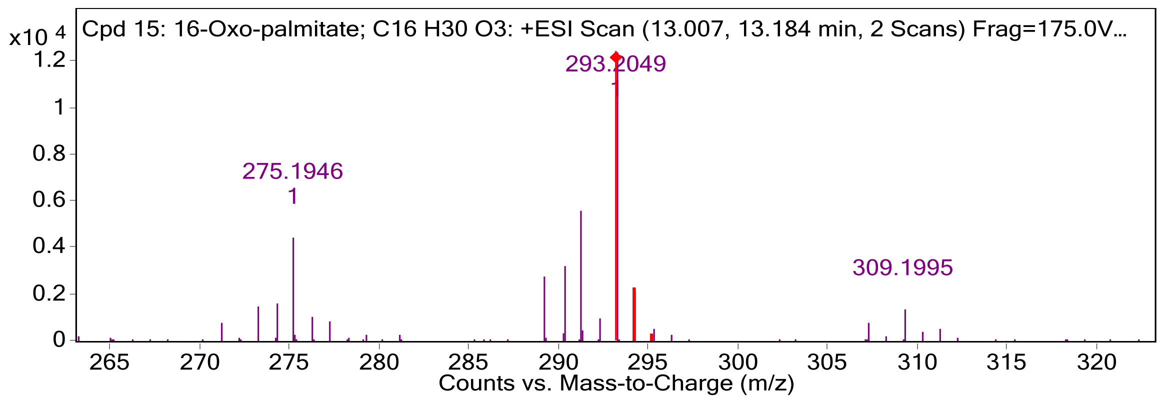

MS Spectrum Peak List

m/z	*Calc m/z*	Diff(ppm)	z	Abund	Formula	Ion
124.0838				68739.91		
125.068			1	42459.39		
129.9105			1	59441.84		
131.9076			1	63583.53		
133.9043				23050.63		
152.0667			1	29674.38		
155.0775			1	13690.87		
293.2049	293.2087	12.88	1	12397.32	C16 H30 O3	(M+Na)+
294.2083	294.2121	12.91	1	2202.4	C16 H30 O3	(M+Na)+
295.2133	295.2148	5.2	1	544.25	C16 H30 O3	(M+Na)+

MSMS Spectrum

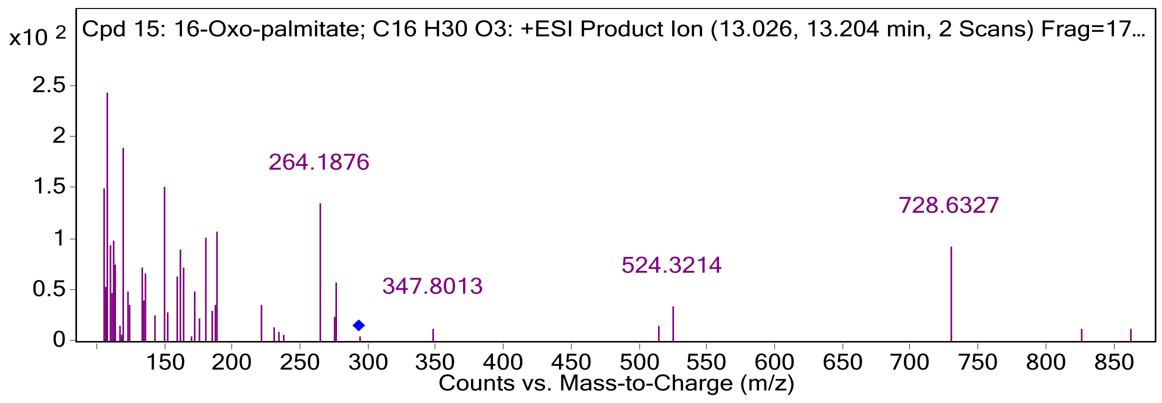

MS/MS Spectrum Peak List

m/z	Abund
105.0681	150.32
107.0458	220.01
107.0816	244.92
109.0673	95.74
112.1314	99.28
119.0819	190.27
149.1257	151.5
180.1552	101.97
188.1123	108.74
264.1876	135.78

Compound Structure

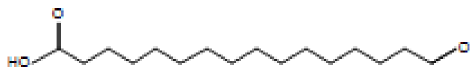

Compound Label	Name	*m/z*	RT	Algorithm	Mass

Cpd 16: Dimethindene; C20 H24 N2	**Dimethindene**	293.2045	13.413	Auto MS/MS	292.1971

MS Spectrum

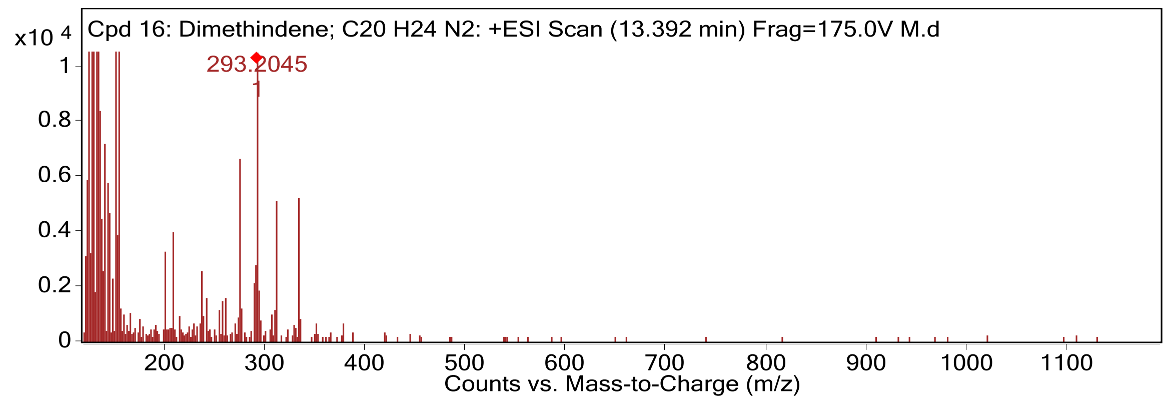

MS Zoomed Spectrum

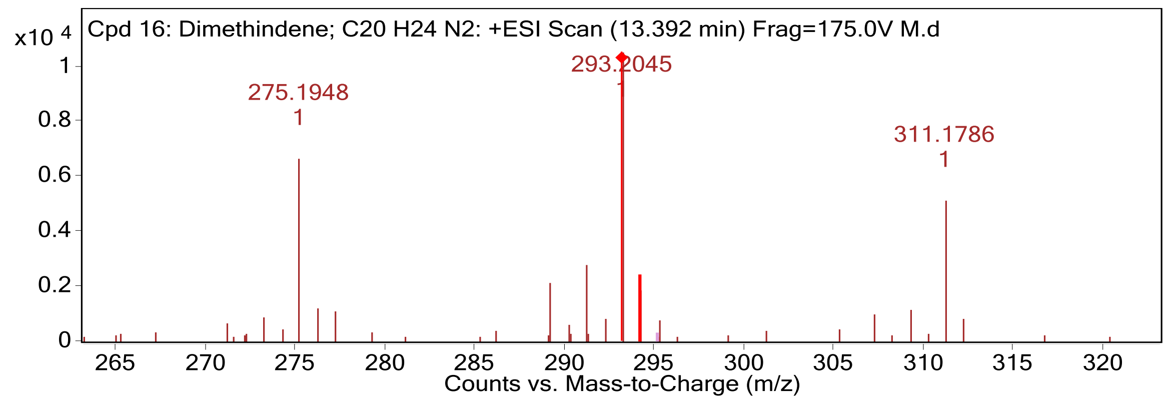

MS Spectrum Peak List

m/z	*Calc m/z*	Diff(ppm)	z	Abund	Formula	Ion
124.0839				70140.1		
125.0685			1	39674.76		
128.0583			1	11948.72		
129.9103			1	61790.66		
131.9076			1	61481.25		
133.9045				23275.24		
152.0669			1	30962.68		
155.0782			1	11627.32		
293.2045	293.2012	-11.01	1	10518.62	C20 H24 N2	(M+H)+
294.207	294.2044	-8.94	1	1893.7	C20 H24 N2	(M+H)+

MSMS Spectrum

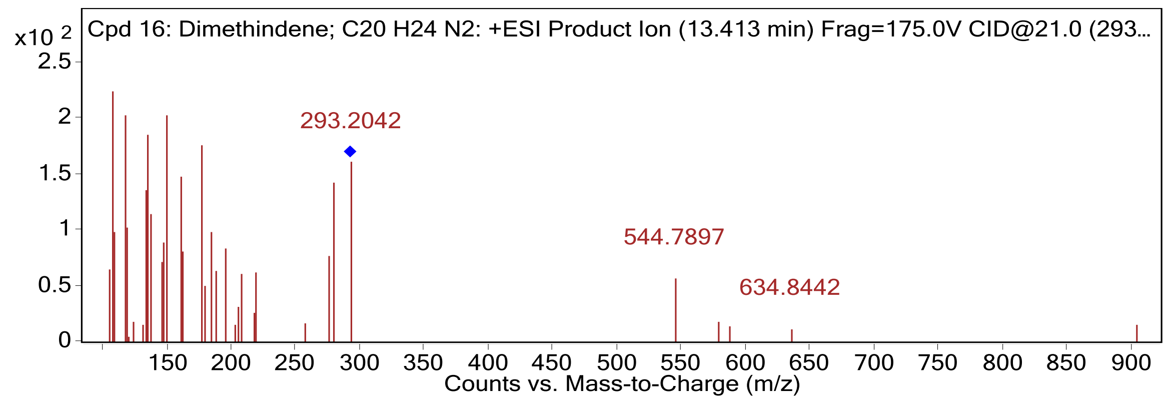

MS/MS Spectrum Peak List

m/z	Abund
107.046	207.35
107.0825	223.7
117.0674	203.03
133.0948	135.98
135.1106	185.41
149.0909	202.47
161.0881	147.97
177.1021	176.3
279.1264	143.27
293.2042	161.16

Compound Structure

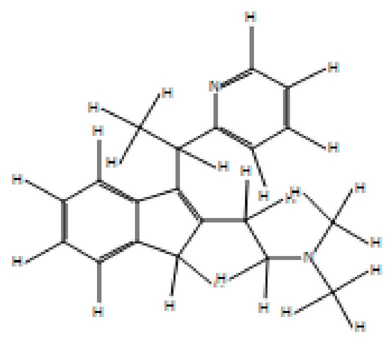

Compound Label	Name	*m/z*	RT	Algorithm	Mass
Cpd 17: D6-Ambrettolide; C16 H28 O2	**D6-Ambrettolide**	275.195	13.907	Auto MS/MS	252.2057

MS Spectrum

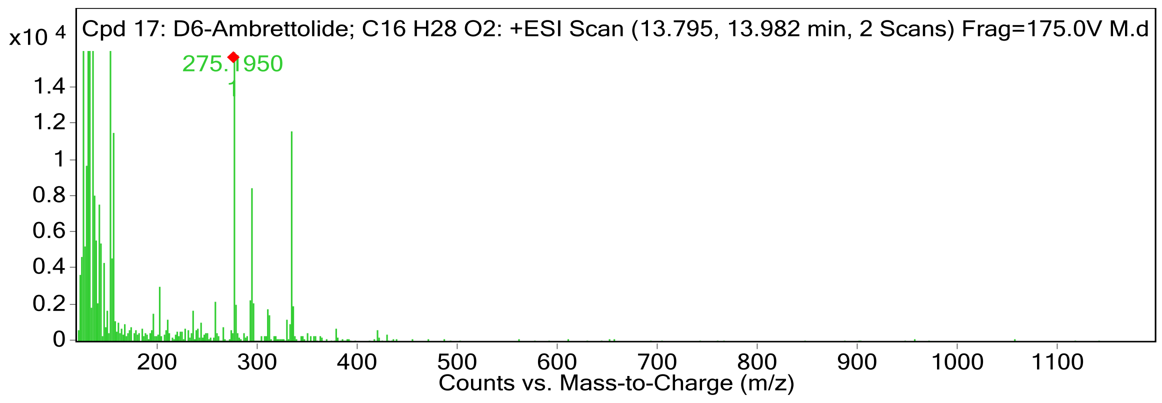

MS Zoomed Spectrum

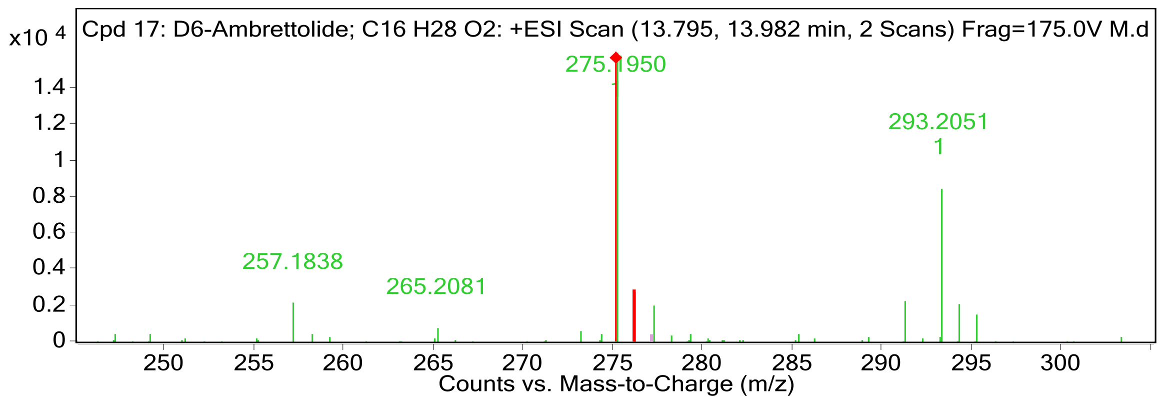

MS Spectrum Peak List

m/z	*Calc m/z*	Diff(ppm)	z	Abund	Formula	Ion
124.0839				68352.01		
125.0681			1	40300.39		
129.9104			1	61839.8		
131.9075			1	62488.7		
133.9044				21576.43		
152.0666			1	31053.5		
155.0775			1	11519.9		
275.195	275.1982	11.55	1	15972.75	C16 H28 O2	(M+Na)+
276.1981	276.2016	12.6	1	2831.47	C16 H28 O2	(M+Na)+
333.1965			1	11611.08		

MSMS Spectrum

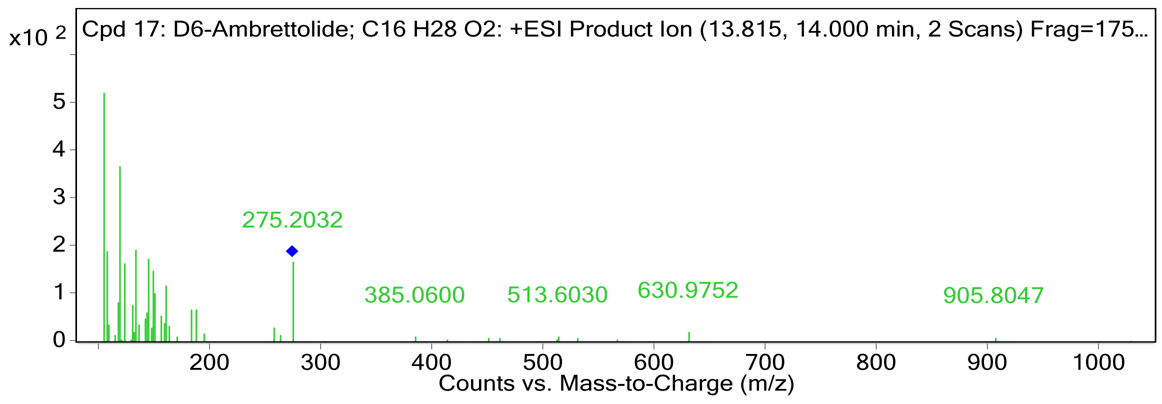

MS/MS Spectrum Peak List

m/z	z	Abund
105.0672		522.24
107.0838		191.91
119.0827		367.45
123.0765		163.98
123.1117		158.16
124.1162		123.24
133.1039		194.84
145.0977		173.89
149.0918	1	148.64
275.2032		168.8

Compound Structure

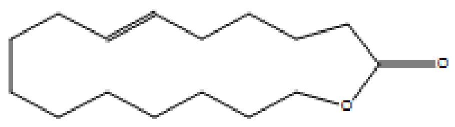

Compound Label	Name	*m/z*	RT	Algorithm	Mass
Cpd 18: Dimethindene; C20 H24 N2	**Dimethindene**	293.2046	13.983	Auto MS/MS	292.1973

MS Spectrum

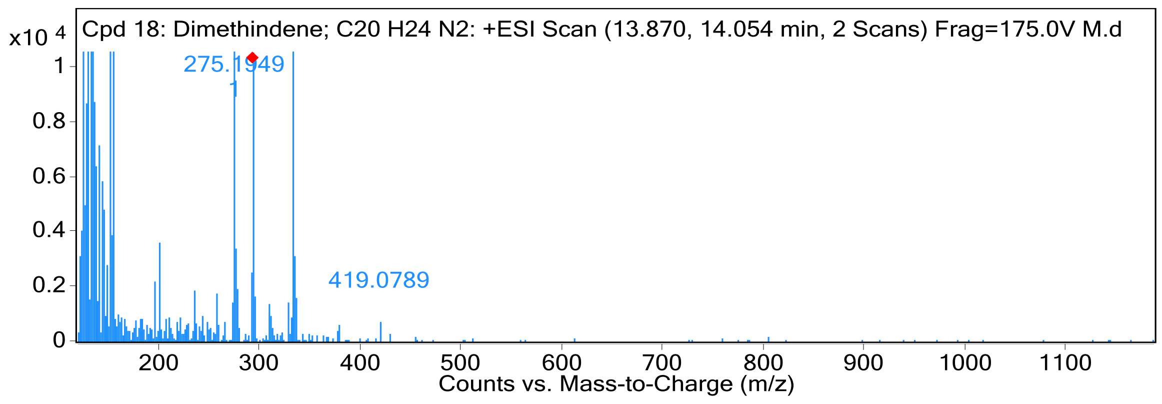

MS Zoomed Spectrum

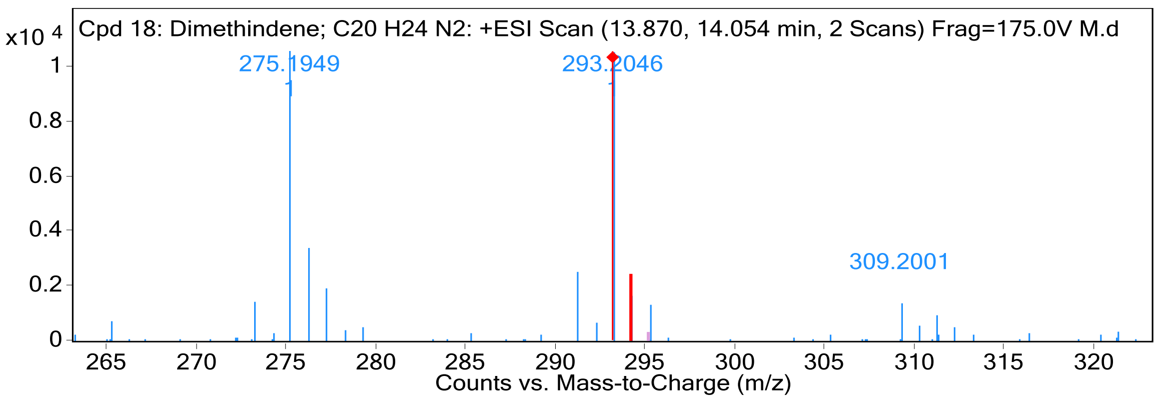

MS Spectrum Peak List

m/z	*Calc m/z*	Diff(ppm)	z	Abund	Formula	Ion
124.0838				67083.45		
125.0682			1	38681.62		
129.9105			1	62600.29		
131.9075			1	62076.98		
133.9046				21445.32		
152.067			1	27746.07		
275.1949			1	17179.8		
293.2046	293.2012	-11.56	1	10544.11	C20 H24 N2	(M+H)+
294.2075	294.2044	-10.53	1	1679.88	C20 H24 N2	(M+H)+
333.1964			1	13649.43		

MSMS Spectrum

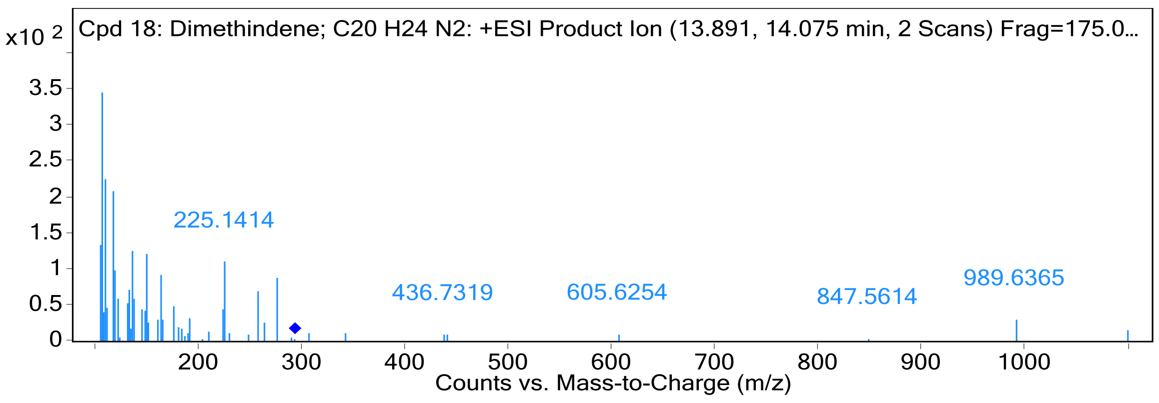

MS/MS Spectrum Peak List

m/z	z	Abund
105.065		135.77
107.0823		347.04
109.0987	1	225.26
117.0675		209.17
119.0847		100.06
135.0756		126.86
149.0917		123.23
163.1075		94.26
225.1414		111.63
275.1944		89.73

Compound Structure

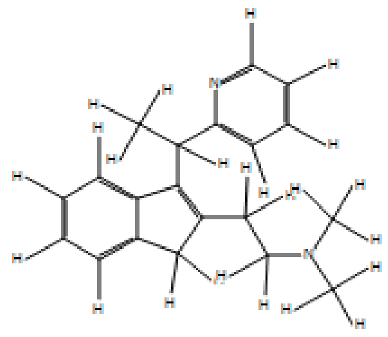

Compound Label	Name	*m/z*	RT	Algorithm	Mass
Cpd 19: D6-Ambrettolide; C16 H28 O2	**D6-Ambrettolide**	275.1948	14.193	Auto MS/MS	252.2056

MS Spectrum

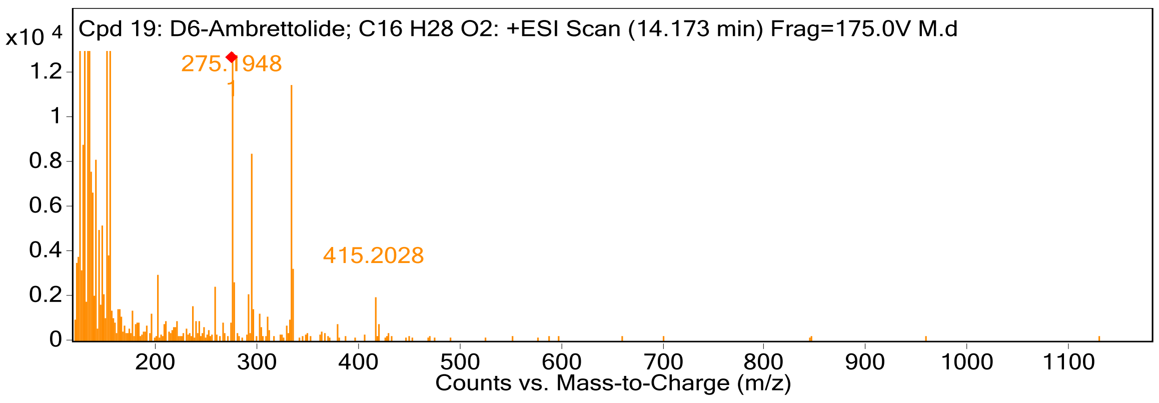

MS Zoomed Spectrum

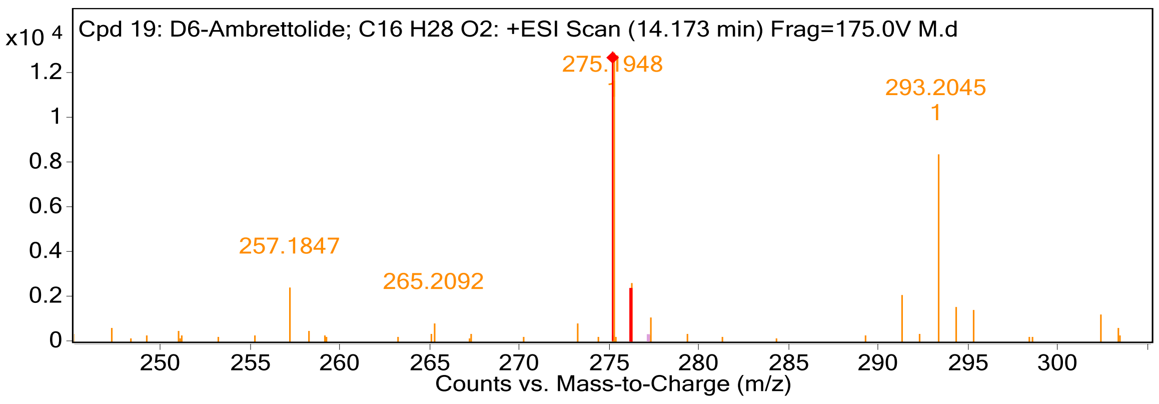

MS Spectrum Peak List

m/z	*Calc m/z*	Diff(ppm)	z	Abund	Formula	Ion
124.0838				71962.19		

125.0681			1	39819.52		
129.9104			1	60398.04		
131.9077			1	68315.27		
133.9044				20713.81		
152.0669			1	25322		
155.0778			1	13598.77		
275.1948	275.1982	12.29	1	12960.06	C16 H28 O2	(M+Na)+
276.1983	276.2016	11.9	1	2687.4	C16 H28 O2	(M+Na)+
333.196			1	11490.68		

MSMS Spectrum

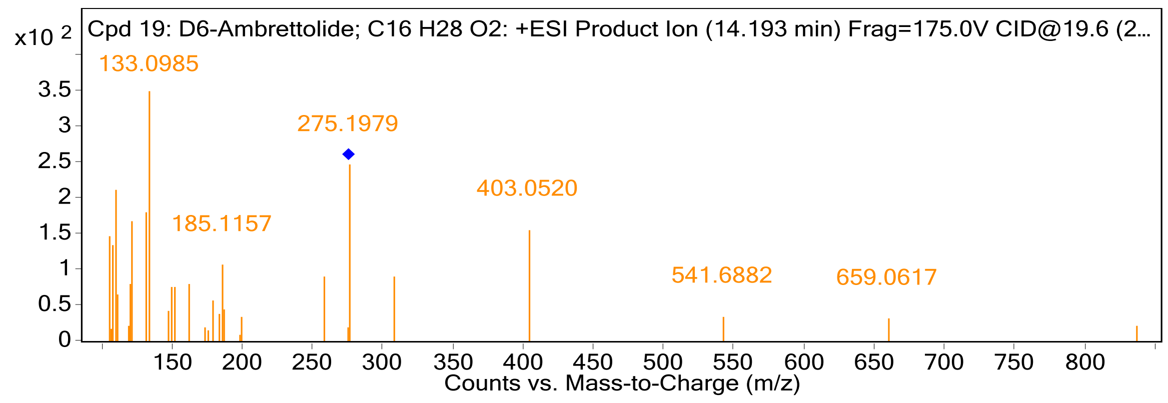

MS/MS Spectrum Peak List

m/z	Abund
105.067	148.51
107.0815	136.01
109.0987	212.56
121.0961	168.61
131.0826	181.97
133.0985	349.12
185.1157	107.44
275.1979	247.4
307.1435	90.85
403.052	157.01

Compound Structure

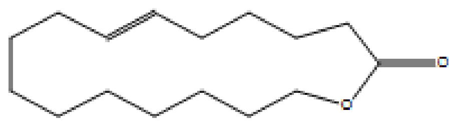

Compound Label	Name	*m/z*	RT	Algorithm	Mass
Cpd 20: (E)-3-Hexadecenoic acid; C16	**(E)-3-Hexadecenoic acid**	277.2101	15.385	Auto MS/MS	254.2209

MS Spectrum

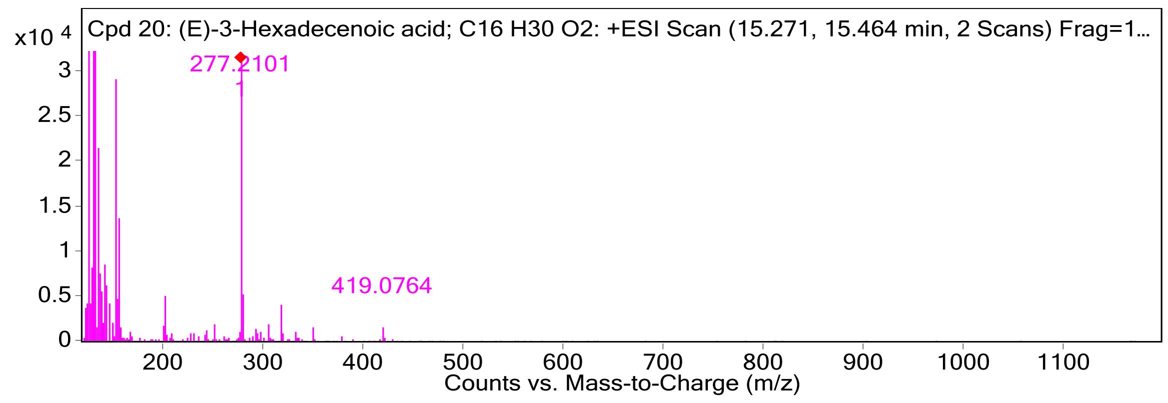

MS Zoomed Spectrum

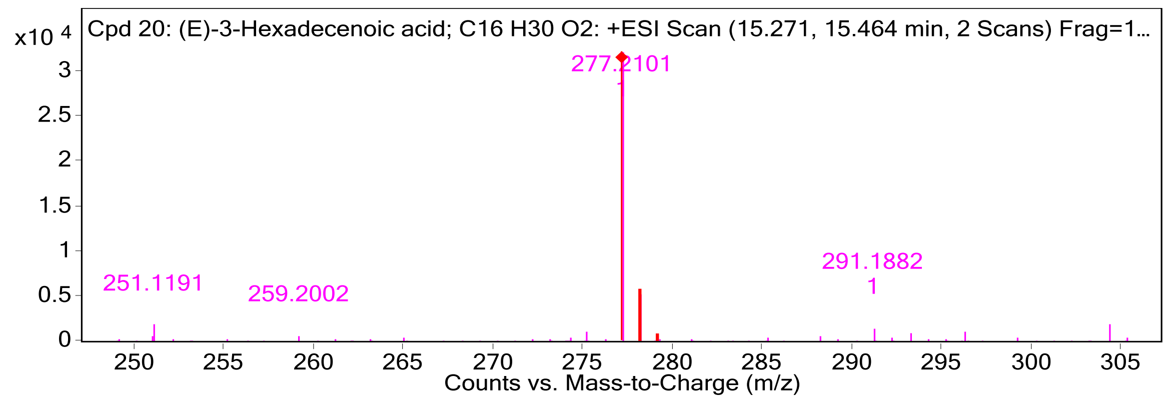

MS Spectrum Peak List

m/z	*Calc m/z*	Diff(ppm)	z	Abund	Formula	Ion
124.0839				73463.54		
125.0682			1	41634.45		
129.9105			1	59518.81		
131.9076			1	64148.18		
133.9044				21479.62		
152.067			1	29165.87		
155.0777			1	13792.51		
277.2101	277.2138	13.32	1	32155.81	C16 H30 O2	(M+Na)+
278.2136	278.2172	13.08	1	5384.3	C16 H30 O2	(M+Na)+
279.2201	279.2201	-0.16	1	394.79	C16 H30 O2	(M+Na)+

MSMS Spectrum

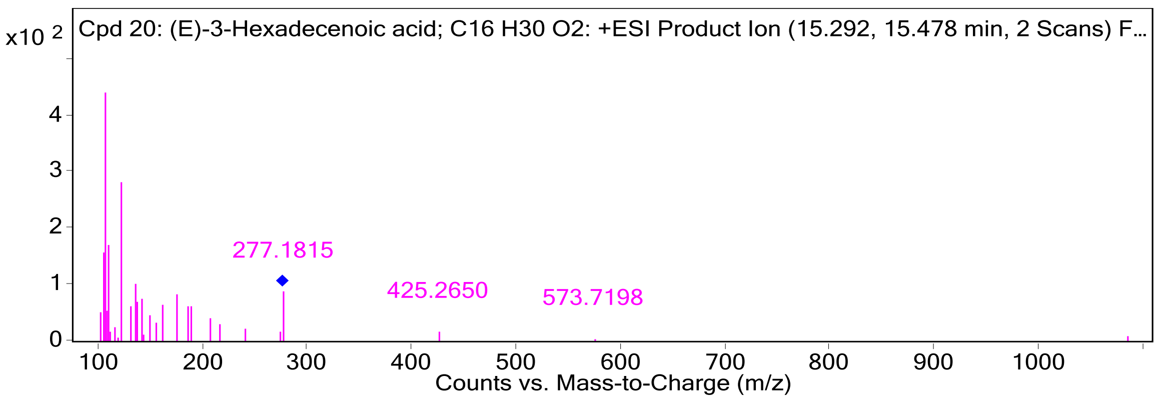

MS/MS Spectrum Peak List

m/z	z	Abund
105.0667		157.73
107.0818	1	442.22
109.0597		82.33
109.0985		171.16
121.0976		283.07
135.1038		99.87
135.1178		102.41
141.0698		75.49
175.0585		83.24
277.1815		88.93

Compound Structure

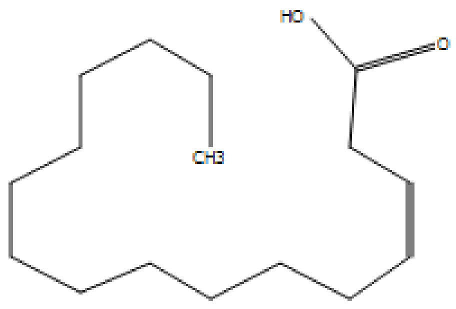

Compound Label	Name	*m/z*	RT	Algorithm	Mass
Cpd 21: (E)-3-Hexadecenoic acid; C16	**(E)-3-Hexadecenoic acid**	277.2101	15.765	Auto MS/MS	254.2209

MS Spectrum

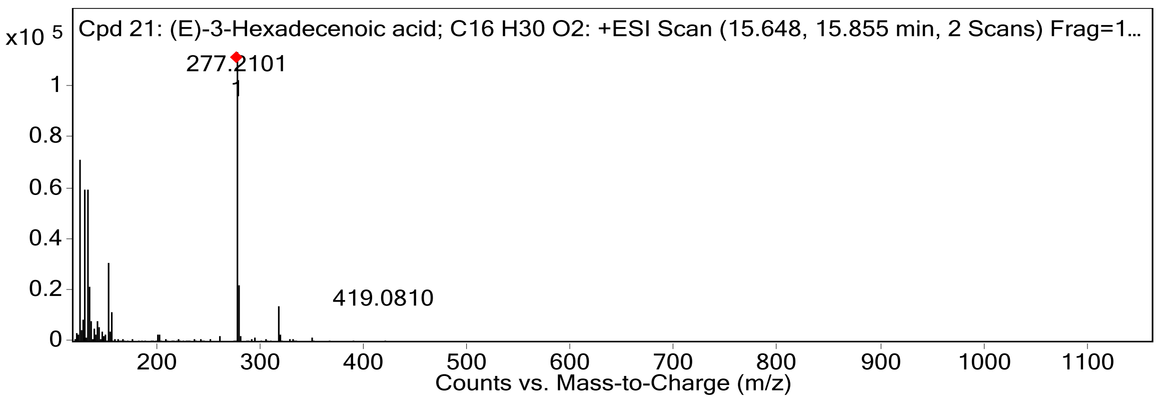

MS Zoomed Spectrum

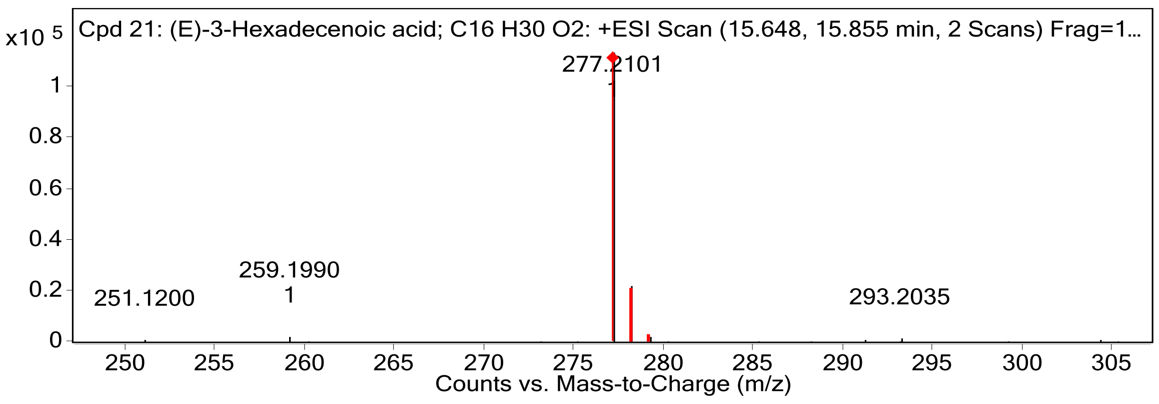

MS Spectrum Peak List

m/z	*Calc m/z*	Diff(ppm)	z	Abund	Formula	Ion
124.0839				71229.91		
125.0681			1	42261.17		
129.9104			1	59878.59		
131.9077			1	59820.49		
133.9044				21544.21		
152.0667			1	31214.92		
277.2101	277.2138	13.27	1	113697.97	C16 H30 O2	(M+Na)+
278.2134	278.2172	13.74	1	22160.74	C16 H30 O2	(M+Na)+
279.2172	279.2201	10.28	1	2257.48	C16 H30 O2	(M+Na)+
317.2015			1	13926.88		

MSMS Spectrum

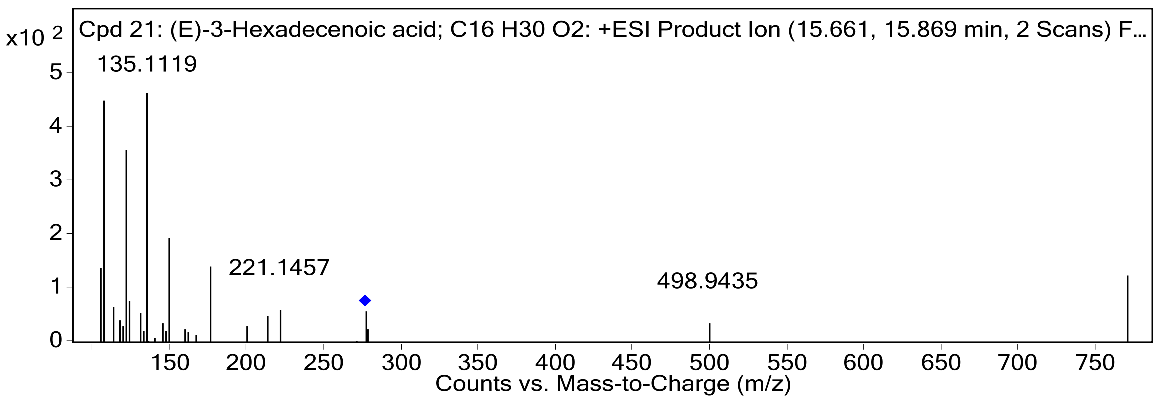

MS/MS Spectrum Peak List

m/z	Abund
105.0256	99.69

105.0658	140.03
107.0613	95.46
107.0833	448.46
121.0977	356.72
123.1129	76.98
135.1119	463.87
149.1276	193.03
176.0952	140.85
769.5311	125.64

Compound Structure

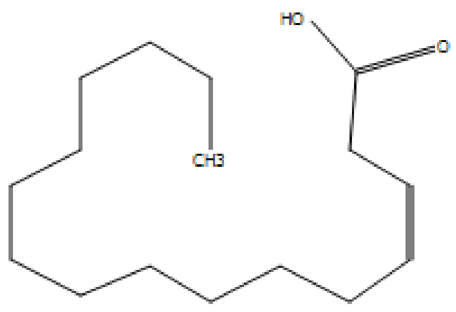

Compound Label	Name	*m/z*	RT	Algorithm	Mass
Cpd 22: (E)-3-Hexadecenoic acid; C16	**(E)-3-Hexadecenoic acid**	277.2102	16.166	Auto MS/MS	254.221

MS Spectrum

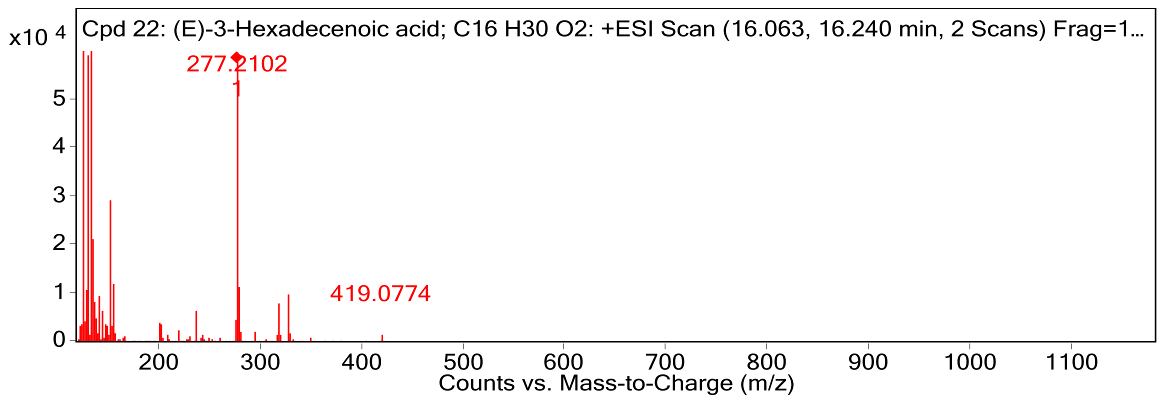

MS Zoomed Spectrum

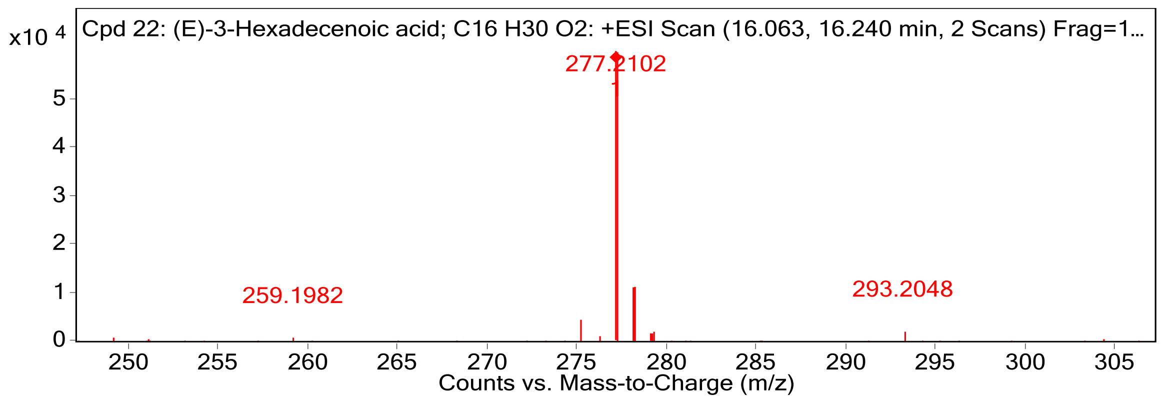

MS Spectrum Peak List

m/z	*Calc m/z*	Diff(ppm)	z	Abund	Formula	Ion
124.0837				75858.09		
125.0681			1	44811.83		
129.9105			1	59077.26		
131.9076			1	60777.27		
133.9045			1	21265.11		
152.0667			1	29219.24		
155.0776			1	12097.79		
277.2102	277.2138	13.05	1	59767.23	C16 H30 O2	(M+Na)+
278.2133	278.2172	14.05	1	11435.29	C16 H30 O2	(M+Na)+
279.2203	279.2201	-0.85	1	2155.55	C16 H30 O2	(M+Na)+

MSMS Spectrum

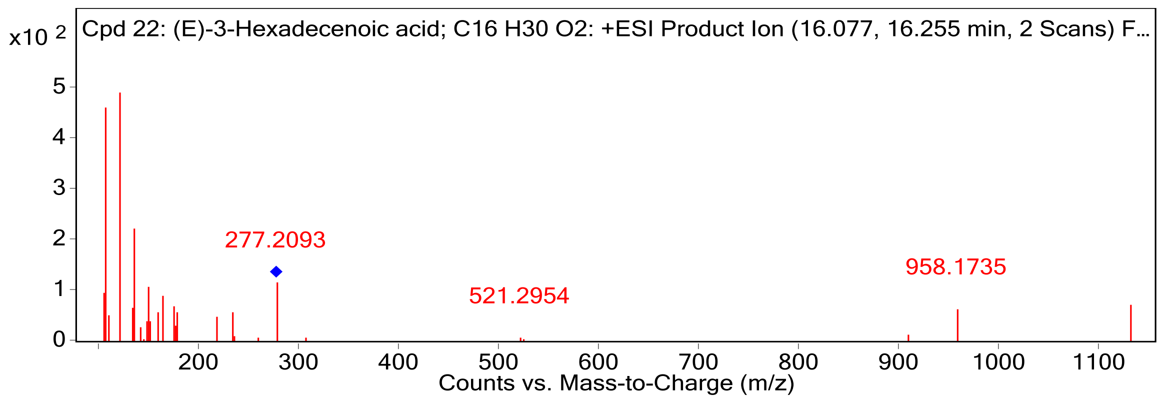

MS/MS Spectrum Peak List

m/z	Abund
105.0661	98.35
107.0818	462.76
121.0975	492.71
133.0962	67.93
135.1114	222.77
149.1299	109.28
163.1442	92.48
175.143	70.39
277.2093	119.26
1130.6228	73.71

Compound Structure

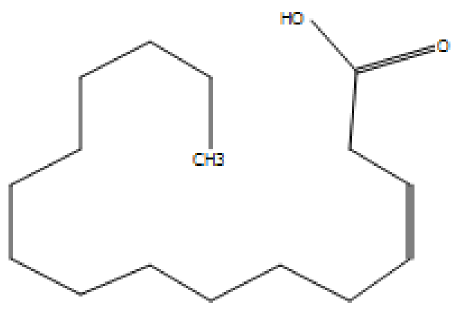

Compound Label	Name	*m/z*	RT	Algorithm	Mass
Cpd 23: Dodecyl butyrate; C16 H32 O2	**Dodecyl butyrate**	279.2259	16.542	Auto MS/MS	256.2366

MS Spectrum

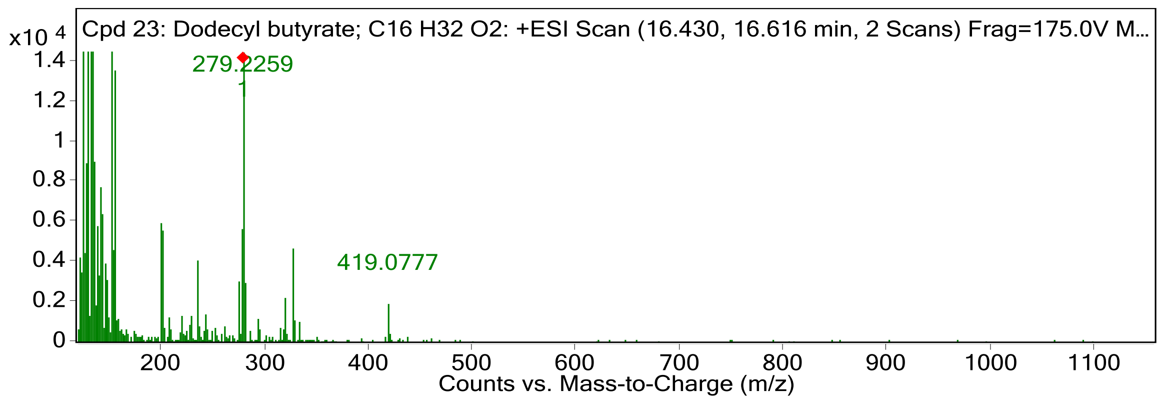

MS Zoomed Spectrum

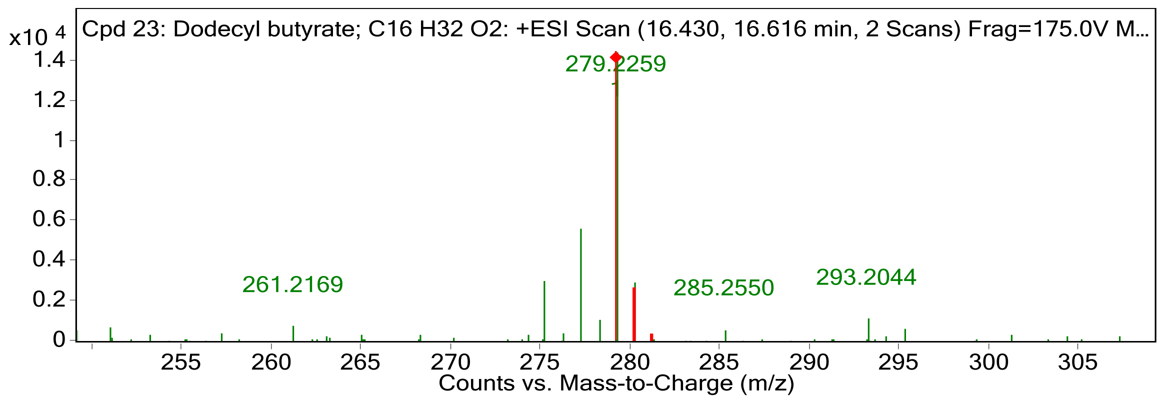

MS Spectrum Peak List

m/z	*Calc m/z*	Diff(ppm)	z	Abund	Formula	Ion
124.0839				72615.34		
125.0681			1	39371.65		
129.9104			1	60357.27		
131.9077			1	57476.33		
133.9042				22887.29		
152.0667			1	30217.78		
155.0774			1	13521.17		
279.2259	279.2295	12.69	1	14427.51	C16 H32 O2	(M+Na)+
280.2287	280.2329	14.72	1	2944.95	C16 H32 O2	(M+Na)+
281.2363	281.2357	-2.16	1	171.05	C16 H32 O2	(M+Na)+

MSMS Spectrum

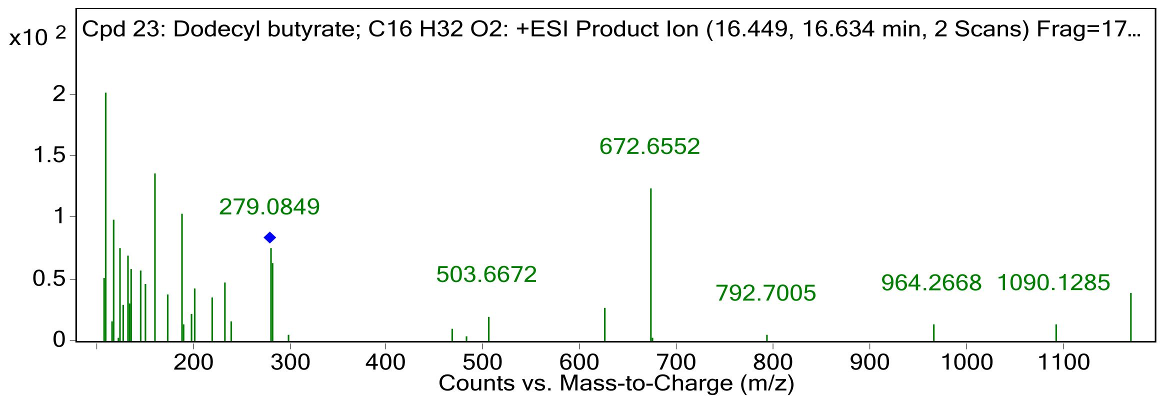

MS/MS Spectrum Peak List

m/z	Abund
108.0862	127.76
109.0989	202.98
117.0674	99.77
123.0784	76.11
131.0778	70
159.1152	137.21
187.1302	104.01
279.0849	76.11
281.0858	64.77
672.6552	125.3

Compound Structure

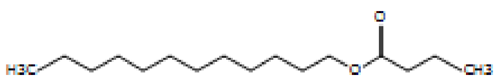

Compound Label	Name	*m/z*	RT	Algorithm	Mass

Cpd 24: (E)-3-Hexadecenoic acid; C16	**(E)-3-Hexadecenoic acid**	277.2101	16.898	Auto MS/MS	254.221

MS Spectrum

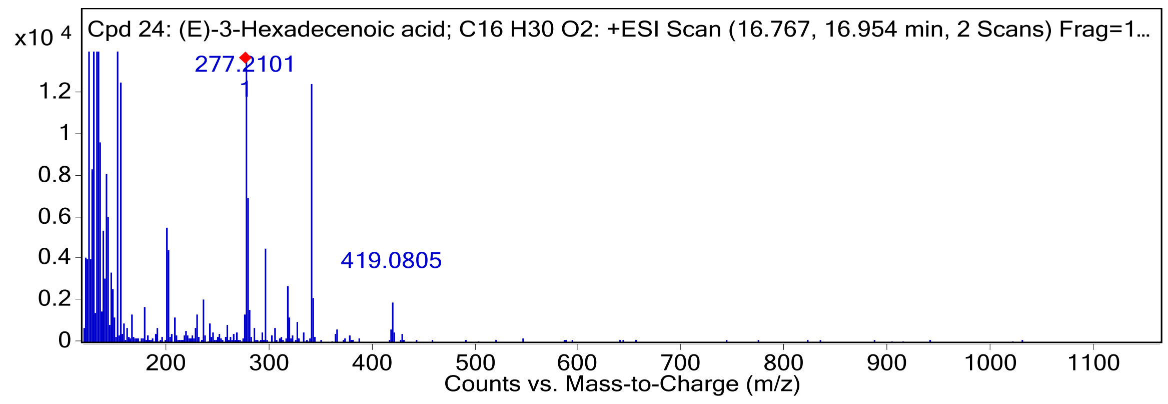

MS Zoomed Spectrum

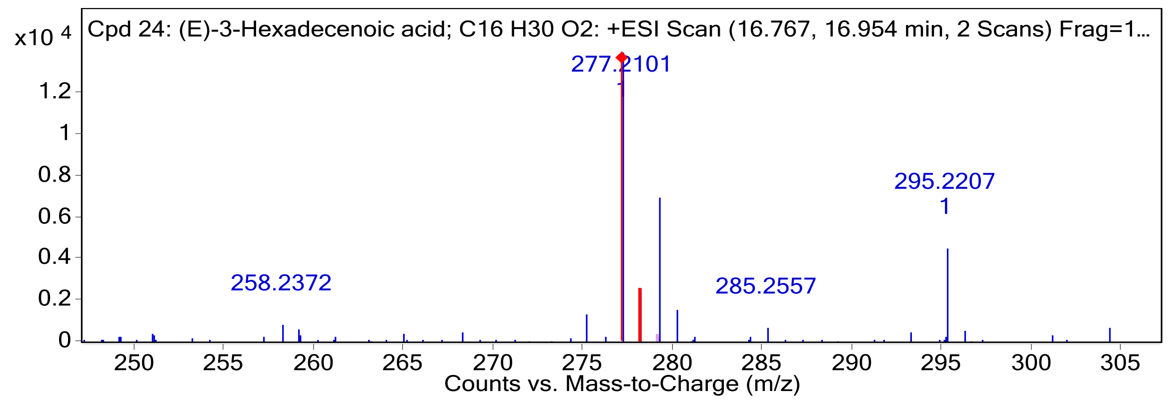

MS Spectrum Peak List

m/z	*Calc m/z*	Diff(ppm)	z	Abund	Formula	Ion
124.0839				74171.3		
125.068			1	42133.13		
129.9105			1	59361.58		
131.9078			1	60131.51		
133.9043				21475.62		
152.067			1	30608.84		
155.0776			1	12528.83		
277.2101	277.2138	13.27	1	13979.07	C16 H30 O2	(M+Na)+
278.2141	278.2172	11.26	1	2591.24	C16 H30 O2	(M+Na)+
341.0864			1	12484.39		

MSMS Spectrum

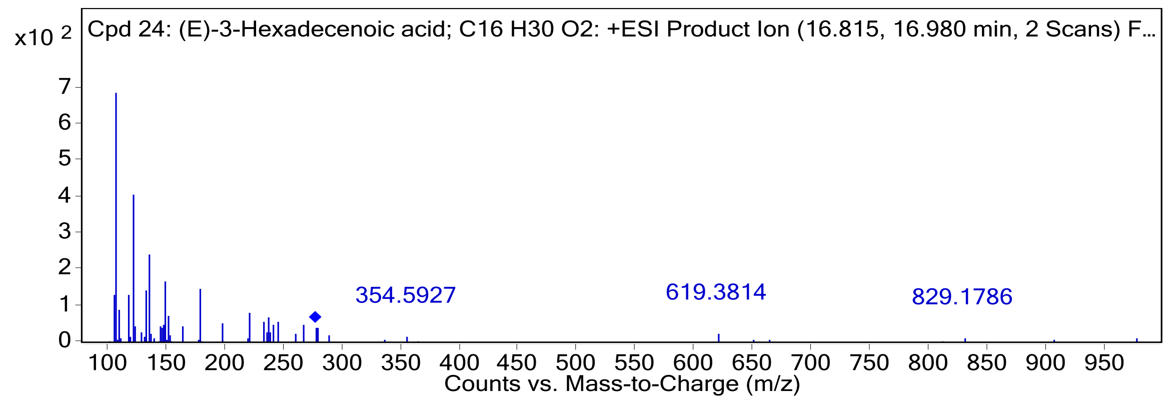

MS/MS Spectrum Peak List

m/z	z	Abund
105.0676		130.3
107.083		687.33
109.0989		90
117.067		132.06
121.0974		405.64
133.0977		143.02
135.1158		242.54
149.129	1	166.57
179.1385		146.1
220.0955		81.31

Compound Structure

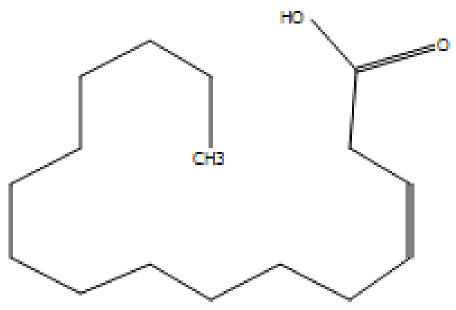

Compound Label	Name	*m/z*	RT	Algorithm	Mass
Cpd 25: (E)-3-Hexadecenoic acid; C16	**(E)-3-Hexadecenoic acid**	277.2102	17.153	Auto MS/MS	254.2211

MS Spectrum

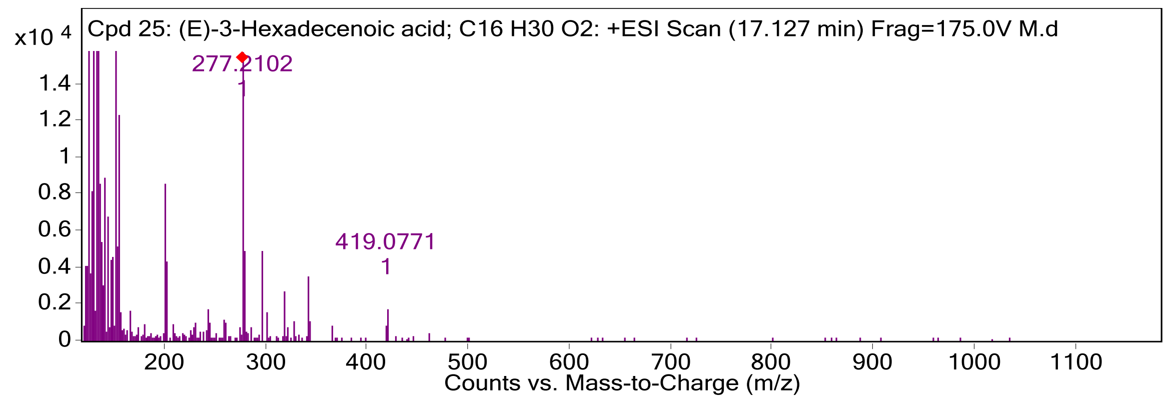

MS Zoomed Spectrum

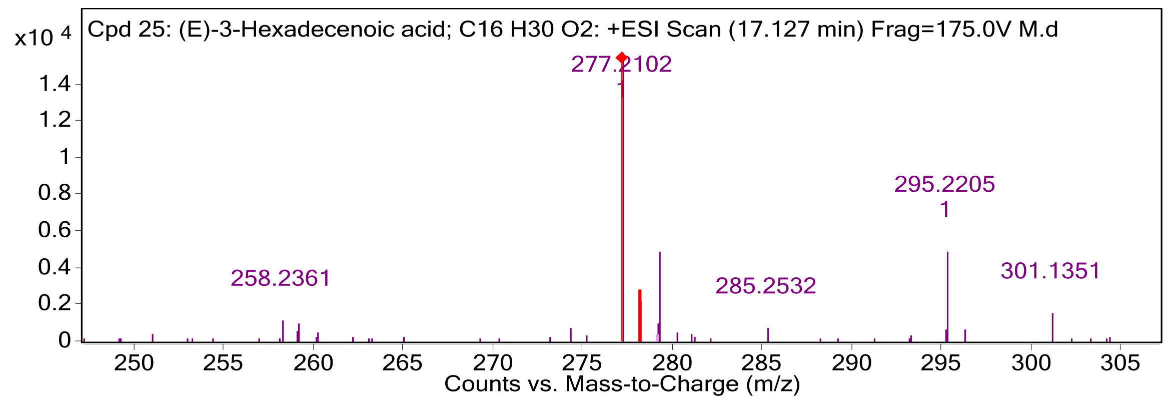

MS Spectrum Peak List

m/z	*Calc m/z*	Diff(ppm)	z	Abund	Formula	Ion
124.0839				72876.48		
125.0684			1	40780.93		
129.9105			1	62310.19		
131.9076			1	58458.9		
133.9044			1	24503.55		
140.984				8959.1		
152.0668			1	28992.62		
155.0774			1	12315.76		
277.2102	277.2138	13.04	1	15754.78	C16 H30 O2	(M+Na)+
278.2144	278.2172	10.09	1	2281.08	C16 H30 O2	(M+Na)+

MSMS Spectrum

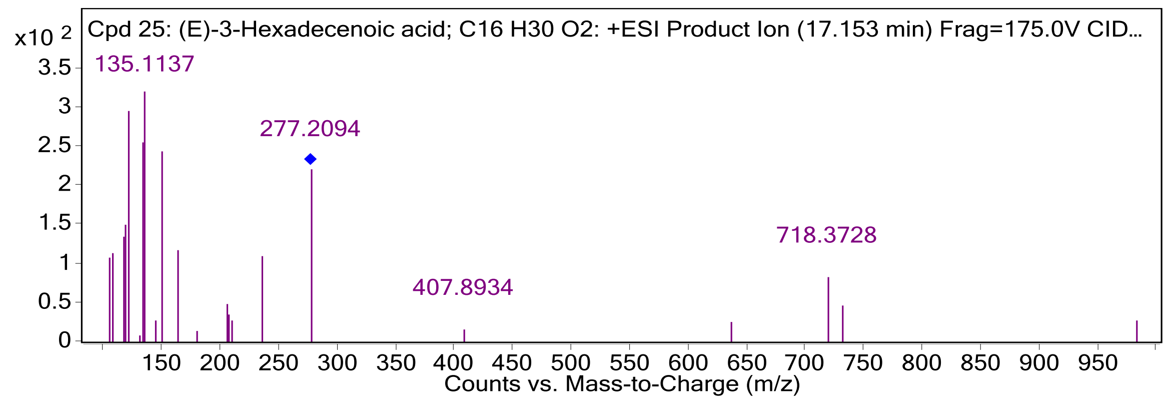

MS/MS Spectrum Peak List

m/z	Abund
107.0869	115.29
117.0671	136.02
119.0814	151.54
121.098	295.36
133.0995	255.68
135.1137	320.56
149.0184	243.47
163.1062	117.97
235.167	111.16
277.2094	220.67

Compound Structure

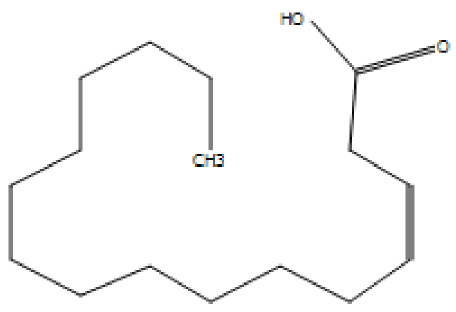

Compound Label	Name	*m/z*	RT	Algorithm	Mass
Cpd 26: Methyl 2-furoate; C6 H6 O3	**Methyl 2-furoate**	149.0194	17.297	Auto MS/MS	126.0302

MS Spectrum

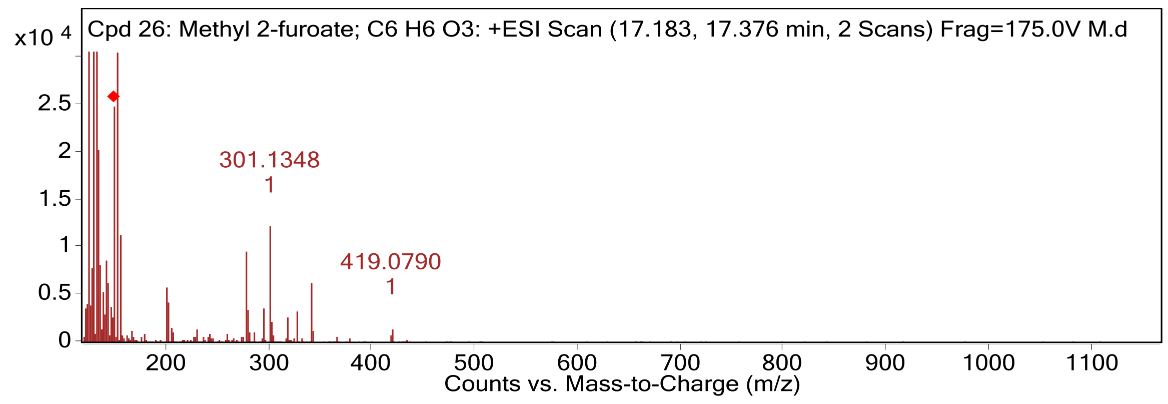

MS Zoomed Spectrum

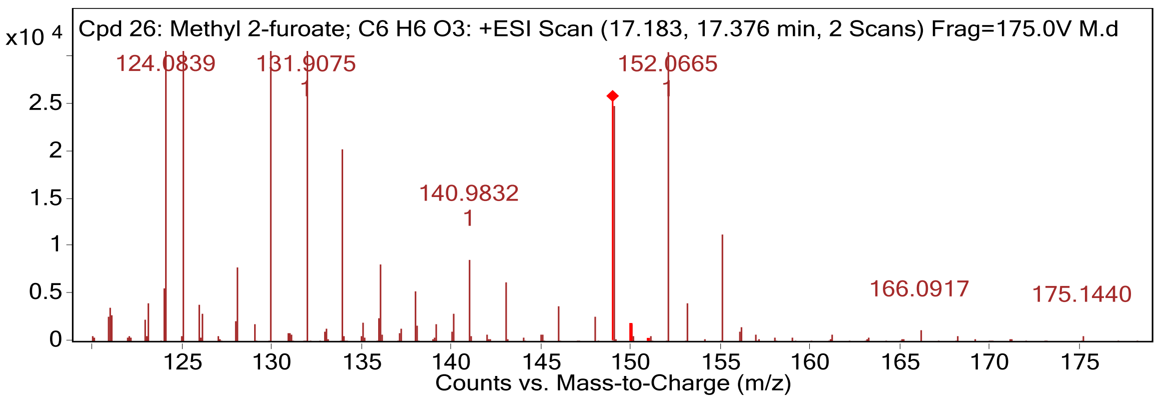

MS Spectrum Peak List

m/z	*Calc m/z*	Diff(ppm)	z	Abund	Formula	Ion
124.0839				78486.23		
125.0681			1	39103.32		
129.9104			1	57446.63		

m/z	*Calc m/z*	Diff(ppm)	z	Abund	Formula	Ion
131.9075			1	58776.06		
133.9044				20355.54		
149.0194	149.0209	10.45	1	24891.51	C6 H6 O3	(M+Na)+
150.0237	150.0243	3.87	1	1888.2	C6 H6 O3	(M+Na)+
151.0222	151.0258	23.35	1	559.82	C6 H6 O3	(M+Na)+
152.0665			1	30498.21		
301.1348			1	12228.15		

MSMS Spectrum

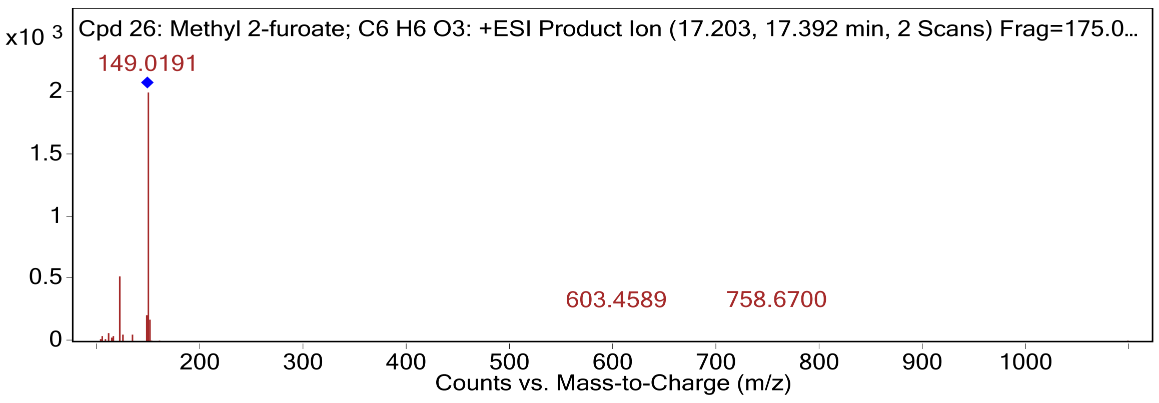

MS/MS Spectrum Peak List

m/z	z	Abund
110.0559		62.09
110.9982		71.53
121.0254	1	529.2
124.9687		62.41
133.0082		56.66
147.9613		210.83
148.9922		59.56
149.0191	1	2003.39
149.1235		63.19
150.0223	1	176.33

Compound Structure

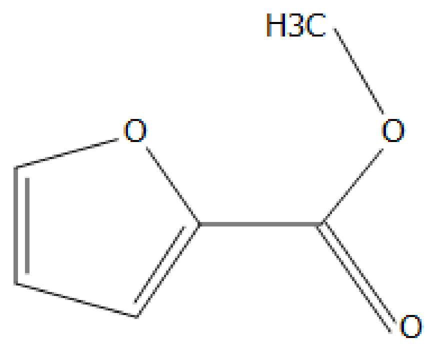

Compound Label	Name	*m/z*	RT	Algorithm	Mass
Cpd 27: Methyl 2-furoate; C6 H6 O3	**Methyl 2-furoate**	149.0195	17.643	Auto MS/MS	126.0303

MS Spectrum

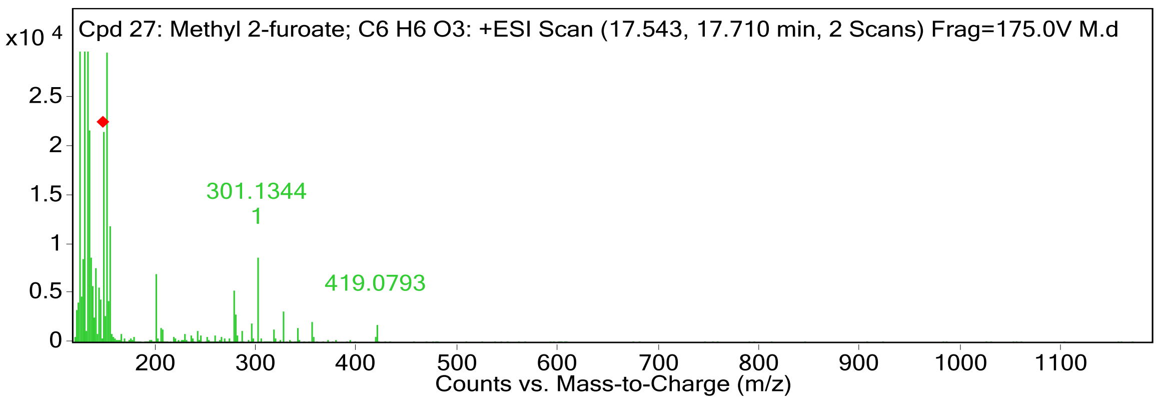

MS Zoomed Spectrum

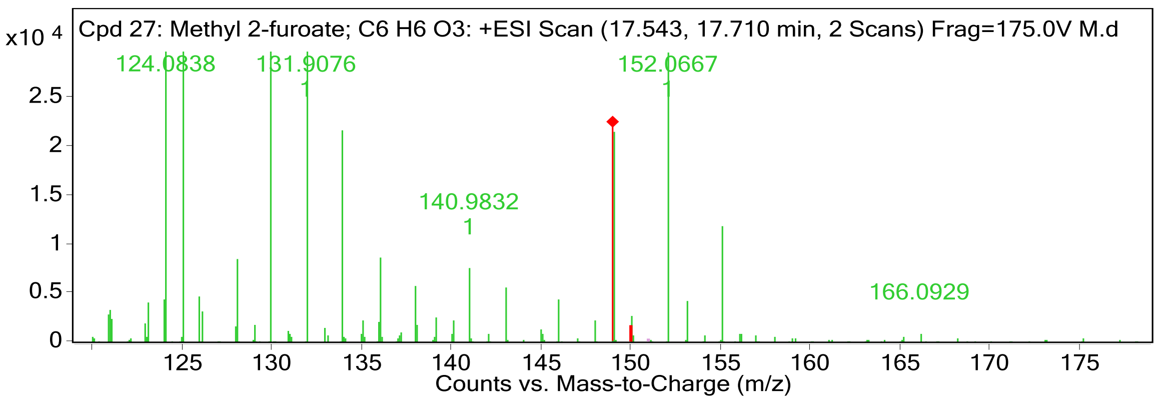

MS Spectrum Peak List

m/z	*Calc m/z*	Diff(ppm)	z	Abund	Formula	Ion
124.0838				72353.3		
125.0682			1	40856.92		
129.9104			1	58822.99		
131.9076			1	59960.64		
133.9044				21755.26		
135.9678				8722.32		
149.0195	149.0209	9.72	1	21573.25	C6 H6 O3	(M+Na)+
150.0234	150.0243	6.33	1	2722.49	C6 H6 O3	(M+Na)+
152.0667			1	29642.45		
155.0775			1	11910.88		

MSMS Spectrum

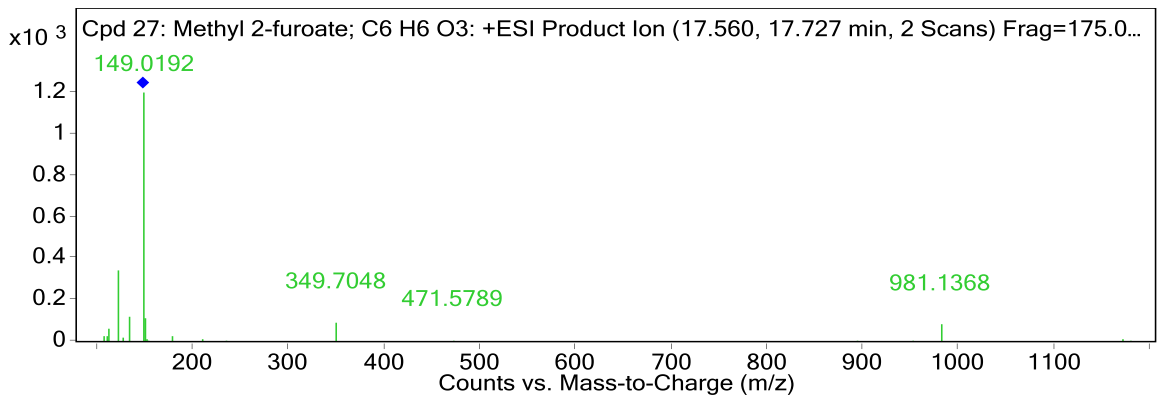

MS/MS Spectrum Peak List

m/z	z	Abund
121.0244	1	344.01
133.0086		120.56
134.0566		68.58
147.9591	1	105.57
149.0192	1	1204.73
149.0549		70.36
150.0225	1	116.39
150.1245		78.51
349.7048		93.73
981.1368		84.04

Compound Structure

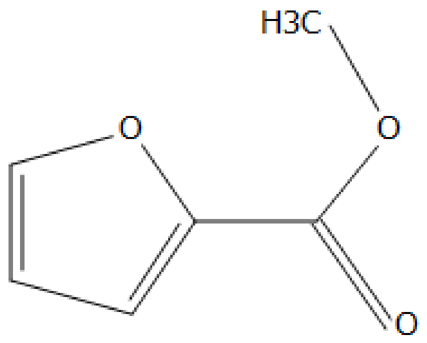

Compound Label	Name	*m/z*	RT	Algorithm	Mass
Cpd 28: Methyl 2-furoate; C6 H6 O3	**Methyl 2-furoate**	149.0194	17.99	Auto MS/MS	126.0301

MS Spectrum

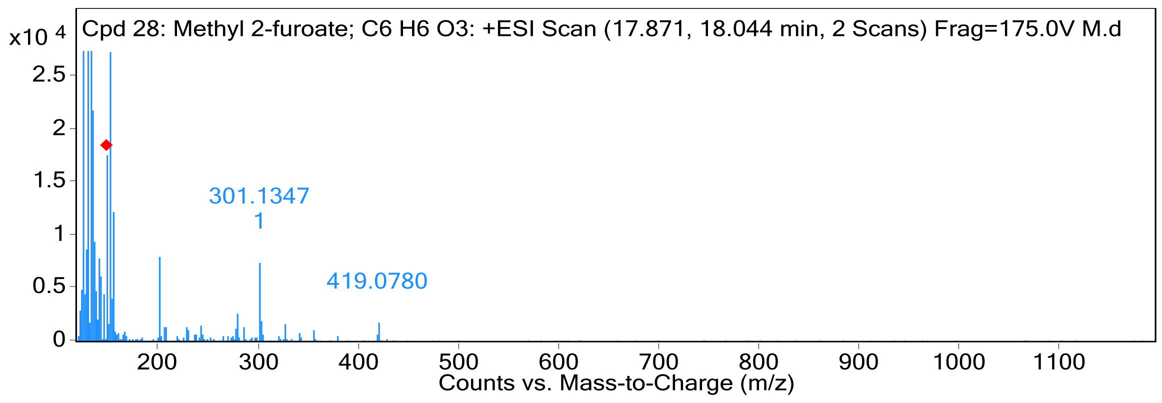

MS Zoomed Spectrum

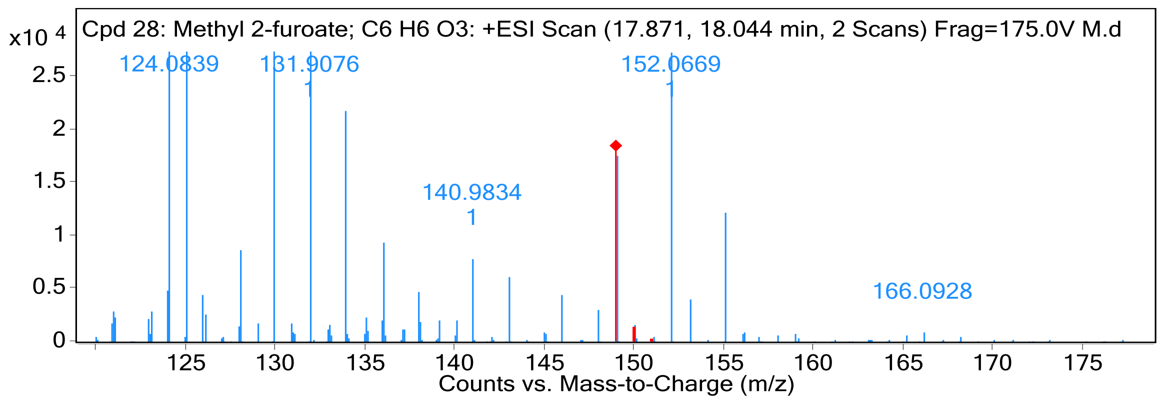

MS Spectrum Peak List

m/z	*Calc m/z*	Diff(ppm)	z	Abund	Formula	Ion
124.0839				70240.72		
125.068			1	38970.88		
129.9106			1	57862.68		
131.9076			1	64086.23		
133.9043			1	21736.7		
149.0194	149.0209	10.49	1	17518.05	C6 H6 O3	(M+Na)+
150.023	150.0243	8.88	1	1709.59	C6 H6 O3	(M+Na)+
151.0213	151.0258	29.66	1	516.56	C6 H6 O3	(M+Na)+
152.0669			1	27249.2		
155.0778			1	12267.01		

MSMS Spectrum

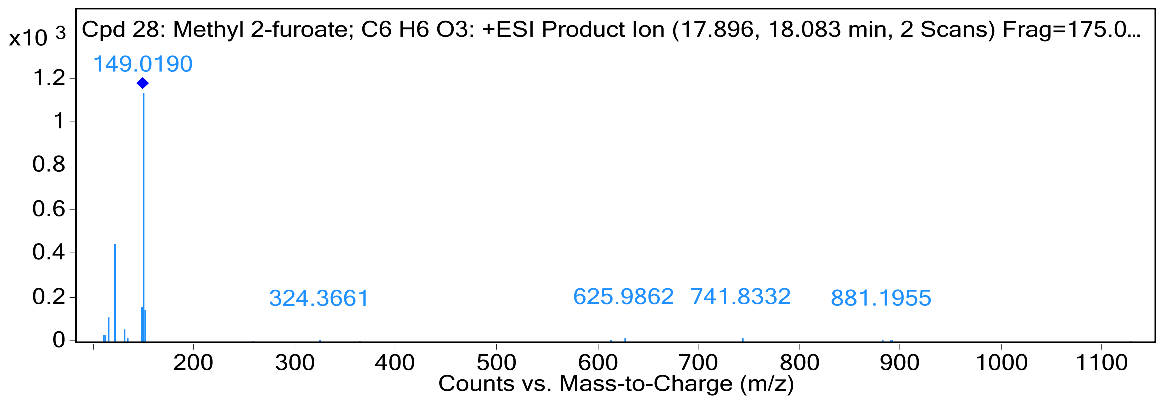

MS/MS Spectrum Peak List

m/z	z	Abund
114.8535		112.28

120.9866		108.03
121.0241		446.72
121.0353		286.92
147.962	1	165.44
149.019	1	1137.79
149.0641		85.28
149.9596	1	130.89
150.02	1	152.76
151.0818		80.51

Compound Structure

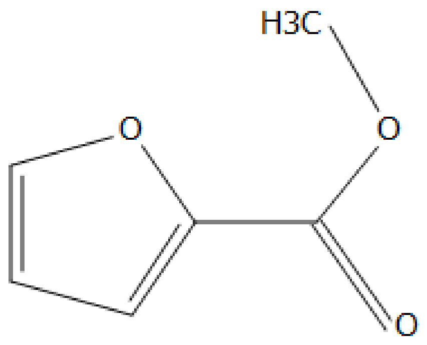

Compound Label	Name	*m/z*	RT	Algorithm	Mass
Cpd 29: Methyl 2-furoate; C6 H6 O3	**Methyl 2-furoate**	149.0194	18.413	Auto MS/MS	126.0303

MS Spectrum

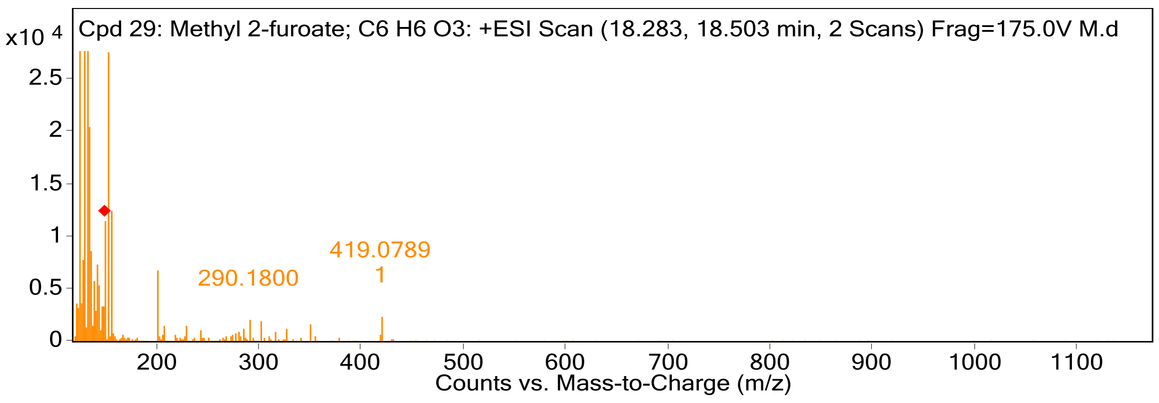

MS Zoomed Spectrum

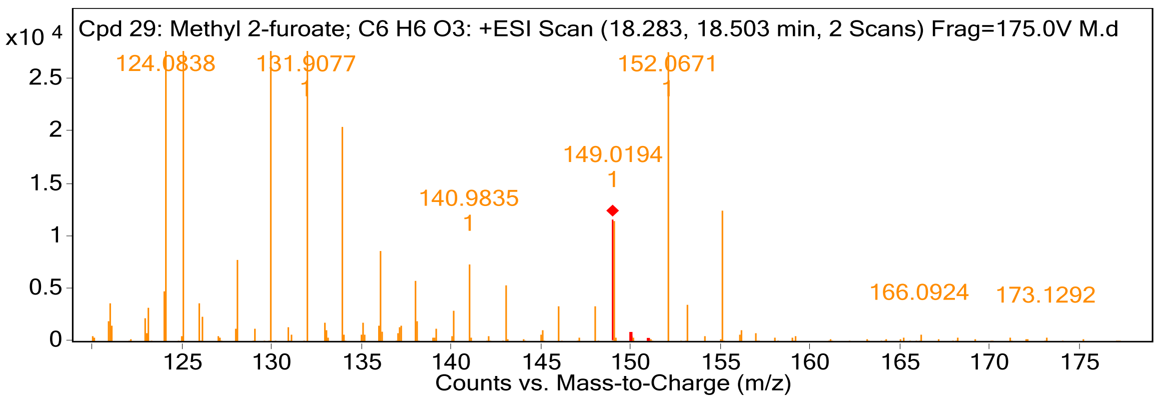

MS Spectrum Peak List

m/z	*Calc m/z*	Diff(ppm)	z	Abund	Formula	Ion
124.0838				71870.3		
125.068			1	39899.23		
129.9104			1	59476.63		
131.9077			1	60361.86		
133.9045				20439.95		
149.0194	149.0209	10.05	1	11531.54	C6 H6 O3	(M+Na)+
150.0254	150.0243	-7.2	1	625.64	C6 H6 O3	(M+Na)+
151.022	151.0258	25.09	1	130.02	C6 H6 O3	(M+Na)+
152.0671			1	27600.64		
155.0775			1	12500.15		

MSMS Spectrum

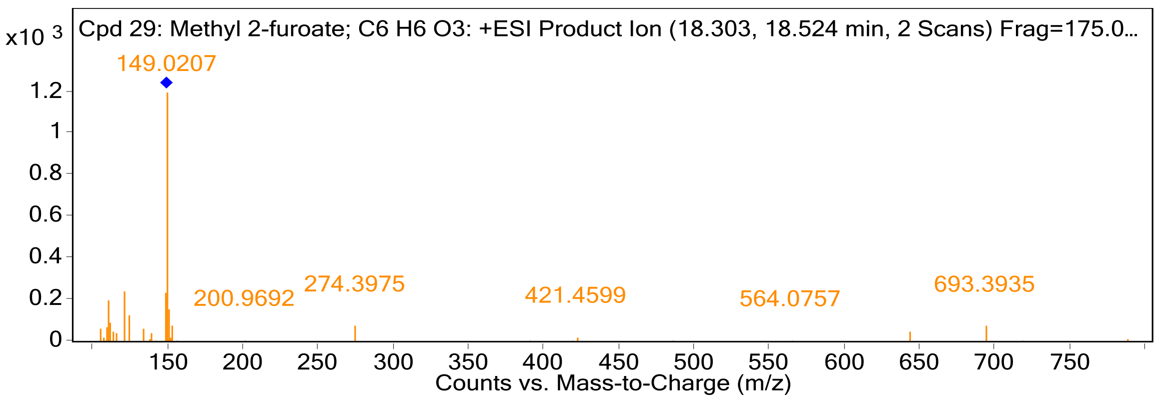

MS/MS Spectrum Peak List

m/z	z	Abund
110.0567		198.89
110.9947		92.88
121.0249		246
124.0704		129.53
147.9625	1	236.58
148.9907		152.12
149.0207	2	1198.39
149.9586	1	161.13
150.0222	2	91.03
150.1239		160.43

Compound Structure

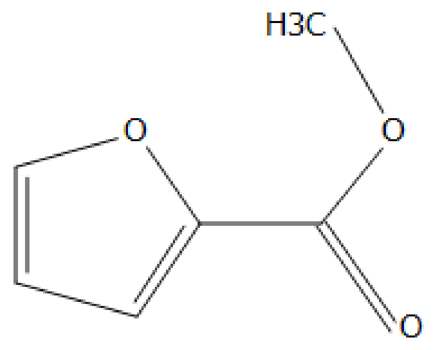

**Compound Label**	***m/z***	**RT**	**Algorithm**
Compound 30	338.3351	24.215	Auto MS/MS

MS Spectrum

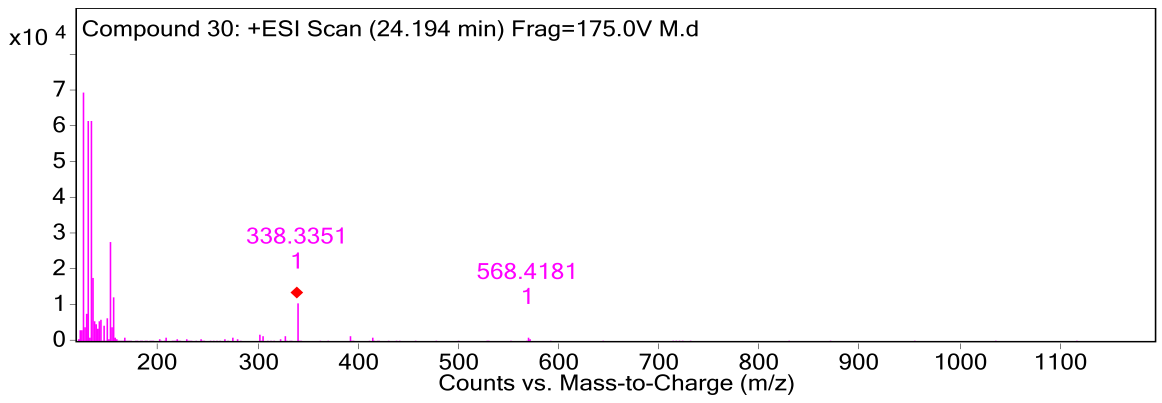

MS Zoomed Spectrum

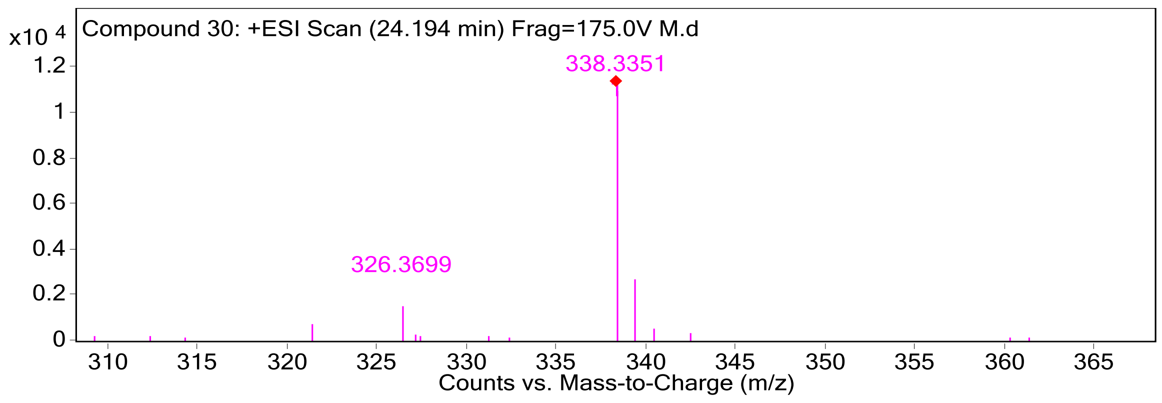

MS Spectrum Peak List

m/z	z	Abund
124.0838		69584.82
125.0682	1	34538.63
129.9102	1	61535.01
131.9076	1	61781.97
133.9047		18016.52
152.067	1	28072.91
155.0775	1	12381.8
338.3351	1	10951.89
339.3376	1	2752.66
340.3449	1	603.64

MSMS Spectrum

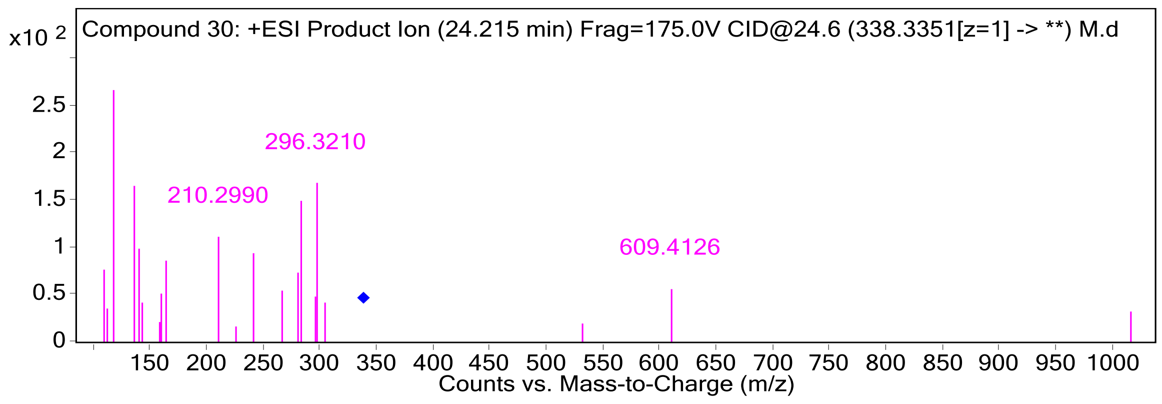

MS/MS Spectrum Peak List

m/z	Abund
109.0934	78
117.0644	266.45
135.1119	166.11
139.1157	98.9
163.1419	86.33
210.299	112.13
240.2324	95.02
279.2571	73.8
282.0491	150.35
296.321	168.91

--- End Of Report ---
