# Supplementary material for: Metabolomic Profiling of Leptadenia reticulata: Unveiling Therapeutic Potential for Inflammatory Diseases through Network Pharmacology and Docking Studies
Source: Pharmaceuticals (Basel). 2024 Mar 26;17(4):423. doi: 10.3390/ph17040423 (PMC11054655; doi:10.3390/ph17040423)
Supplement: Supplementary file 1 [file pharmaceuticals-17-00423-s001.zip › Supplementary Figure.pdf]

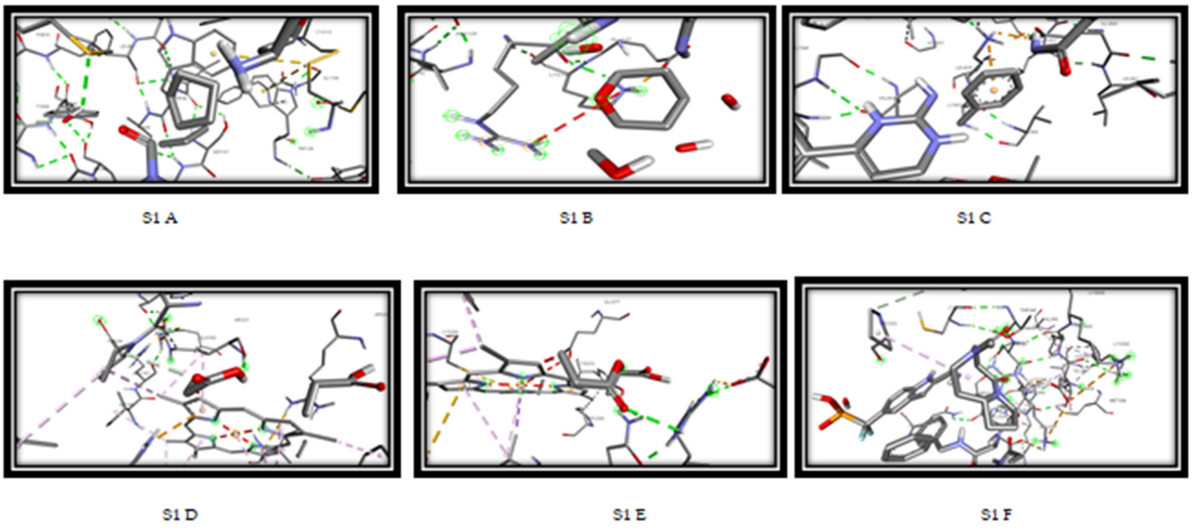

Supplementary Figure S1: S1A) Interaction of CCR2 and ligand; S1 B) Interaction of ICAM-1 and ligand; S1 C) Interaction of KIT and ligand; S1 D) Interaction of MPO and ligand; S1 E) Interaction of NOS2 and ligand; S1 F) Interaction of STAT3 and ligand.

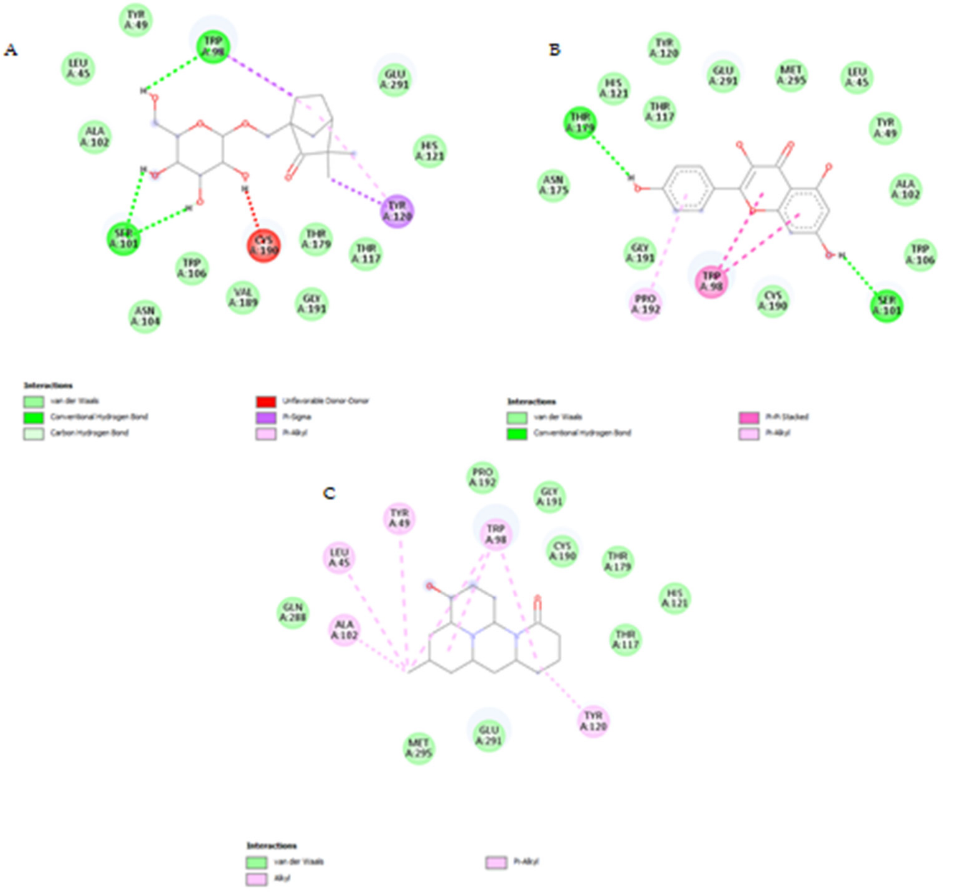

Supplementary Figure S2: A) Interaction residue of (1S,4R)-10-Hydroxyfenchone glucoside with CCR2; B) Interaction residue of Kaempferol with CCR2; C) Interaction residue of Lycocernuine with CCR2.

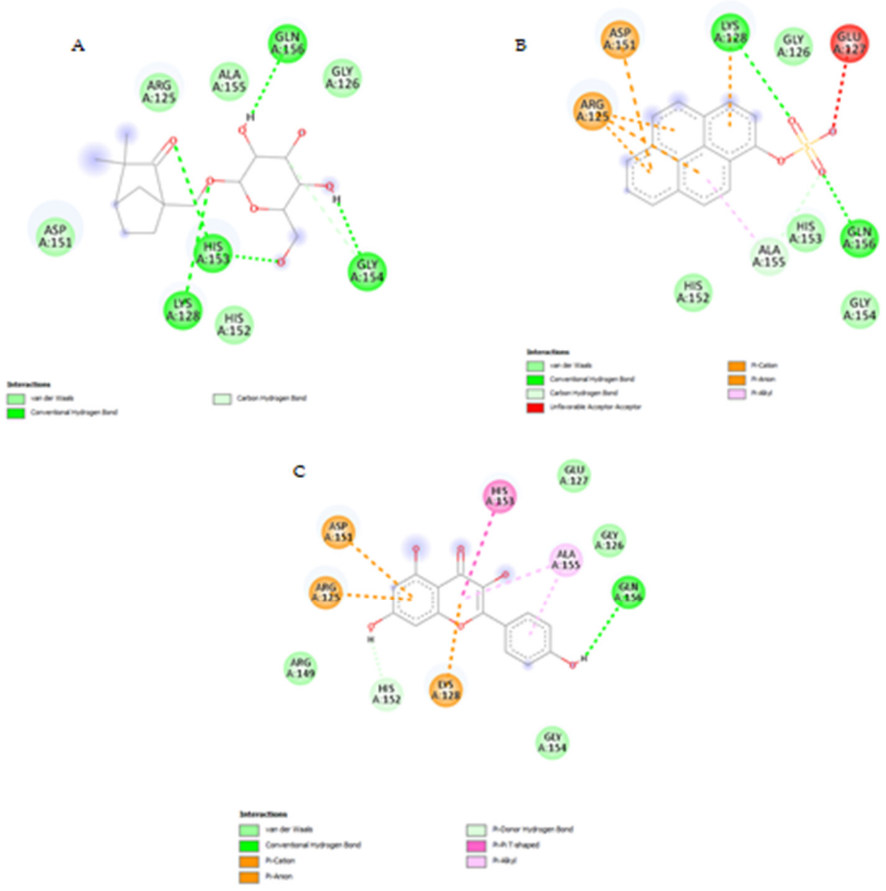

Supplementary Figure S3: A) Interaction residue of (1S,4R)-10-Hydroxyfenchone glucoside with ICAM-1; B) Interaction residue of 1-Pyrenylsulfate with ICAM-1; C) Interaction residue of Kaempferol with ICAM-1.

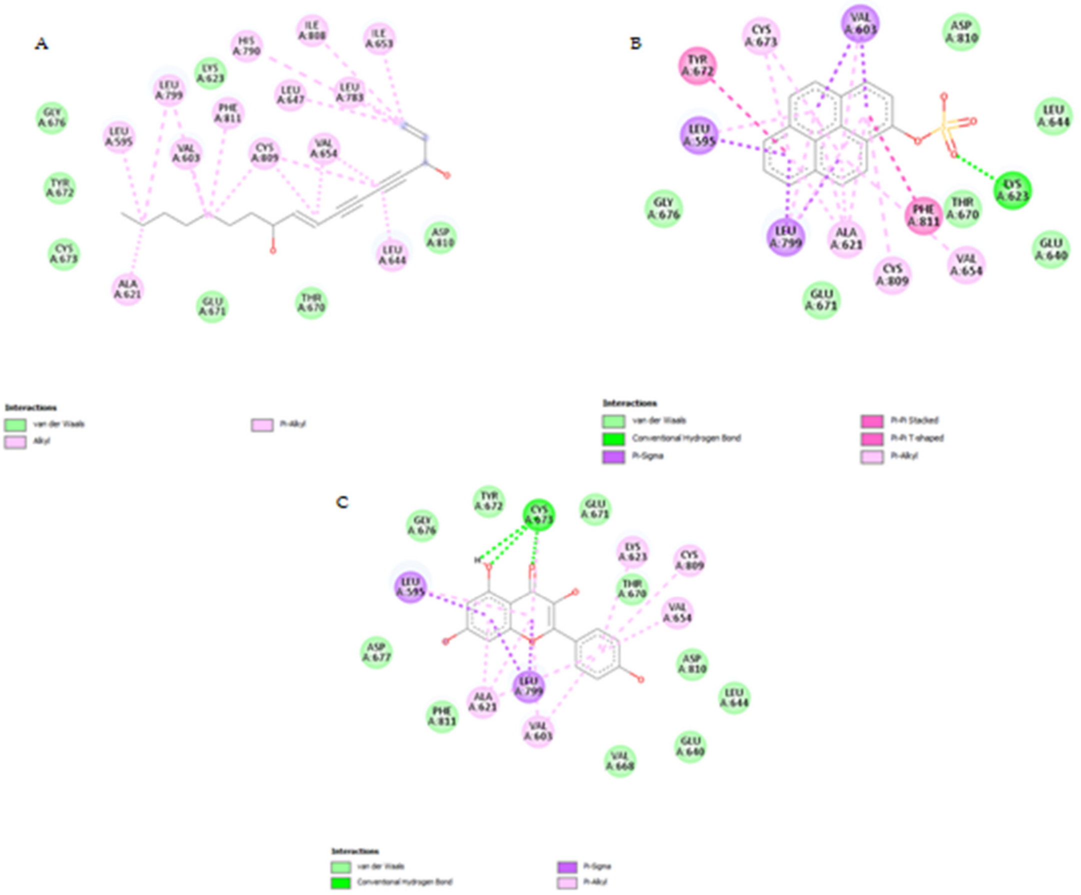

Supplementary Figure S4: A) Interaction residue of 1,8-Heptadecadiene-4,6-diyne-3,10-diol with KIT; B) Interaction residue of 1-Pyrenylsulfate with KIT; C) Interaction residue of Kaempferol with KIT.

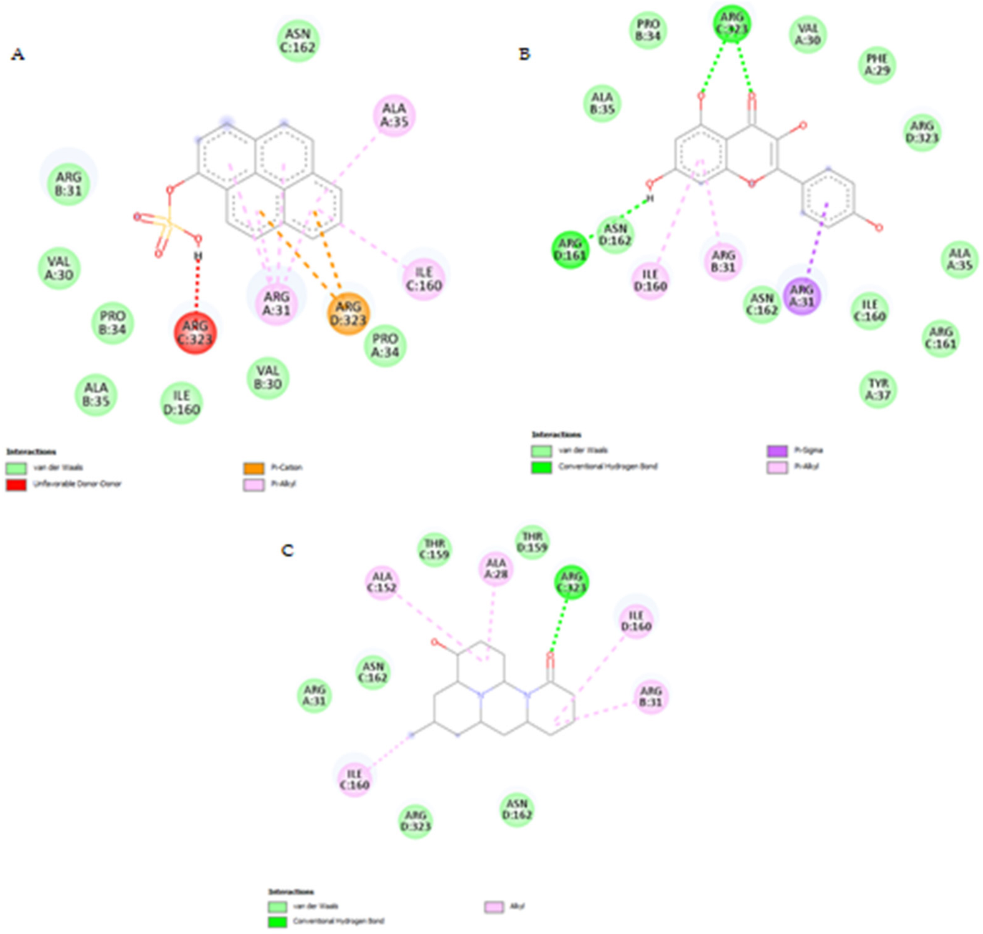

Supplementary Figure S5: A) Interaction residue of 1-Pyrenylsulfate with MPO; B) Interaction residue of Kaempferol with MPO; C) Interaction residue of Lycocernuine with MPO.

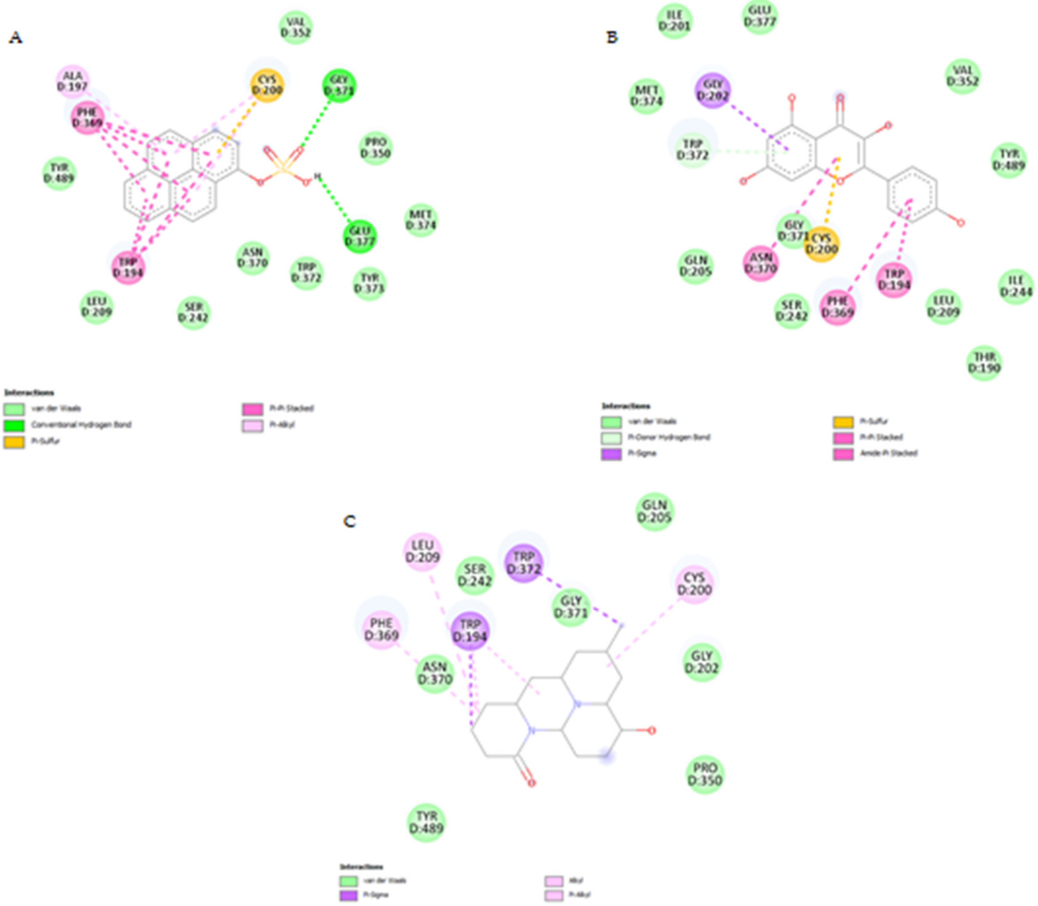

Supplementary Figure S6: A) Interaction residue of 1-Pyrenylsulfate with NOS2; B) Interaction residue of Kaempferol with NOS2; C) Interaction residue of Lycocernuine with NOS2.

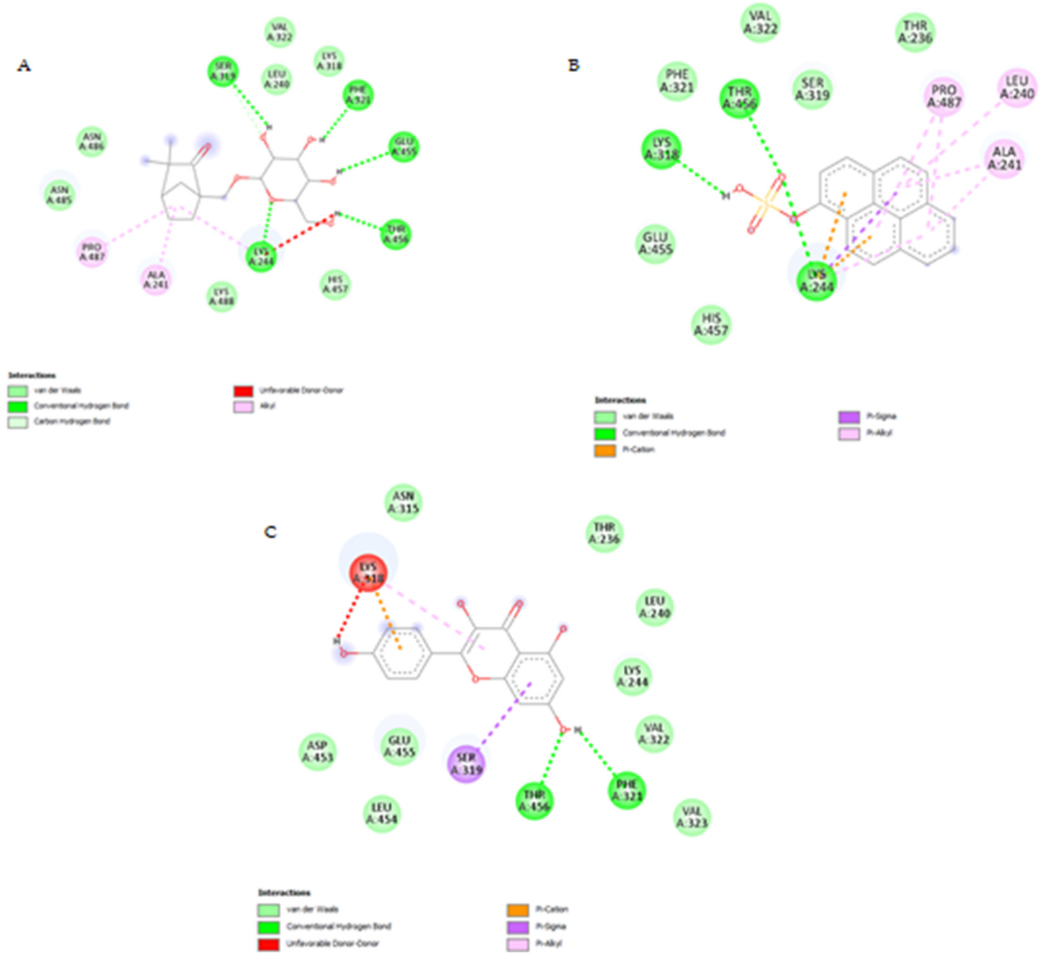

Supplementary Figure S7: A) Interaction residue of (1S,4R)-10-Hydroxyfenchone with STAT3; B) Interaction residue of glucoside1-Pyrenylsulfate with STAT3; C) Interaction residue of Kaempferol with STAT3.

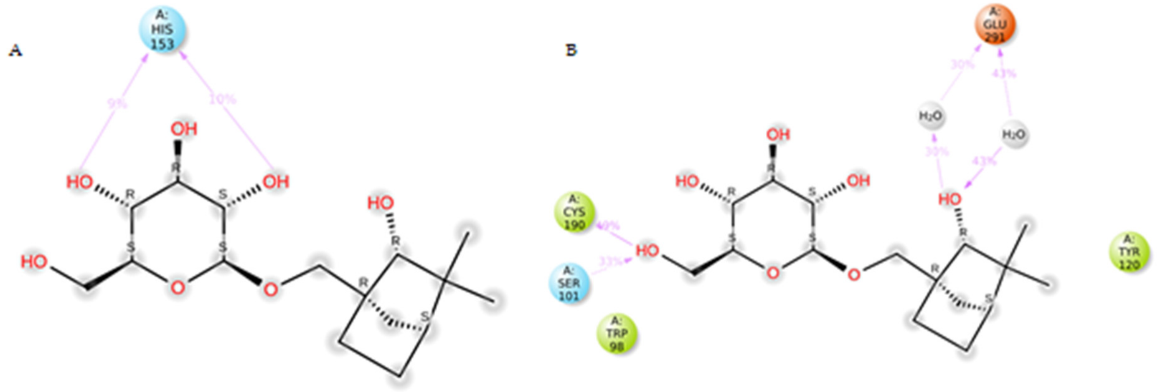

Supplementary Figure S8: A) Interactions of (1S,4R)-10-Hydroxyfenchone glucoside with ICAM1; B) Interactions of (1S,4R)-10-Hydroxyfenchone glucoside with CCR2.

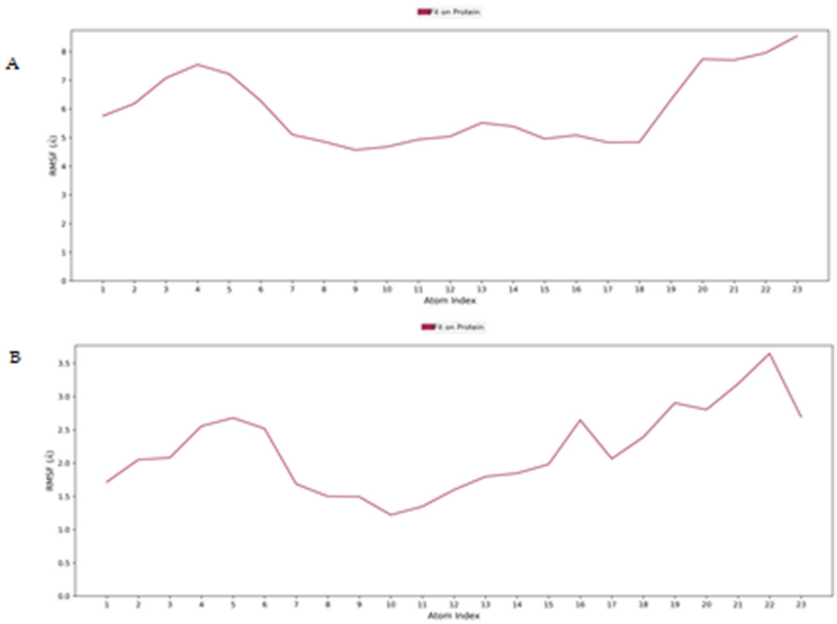

Supplementary Figure S9: A) ICAM1 Protein RMSF; B) CCR2 Protein RMSF.

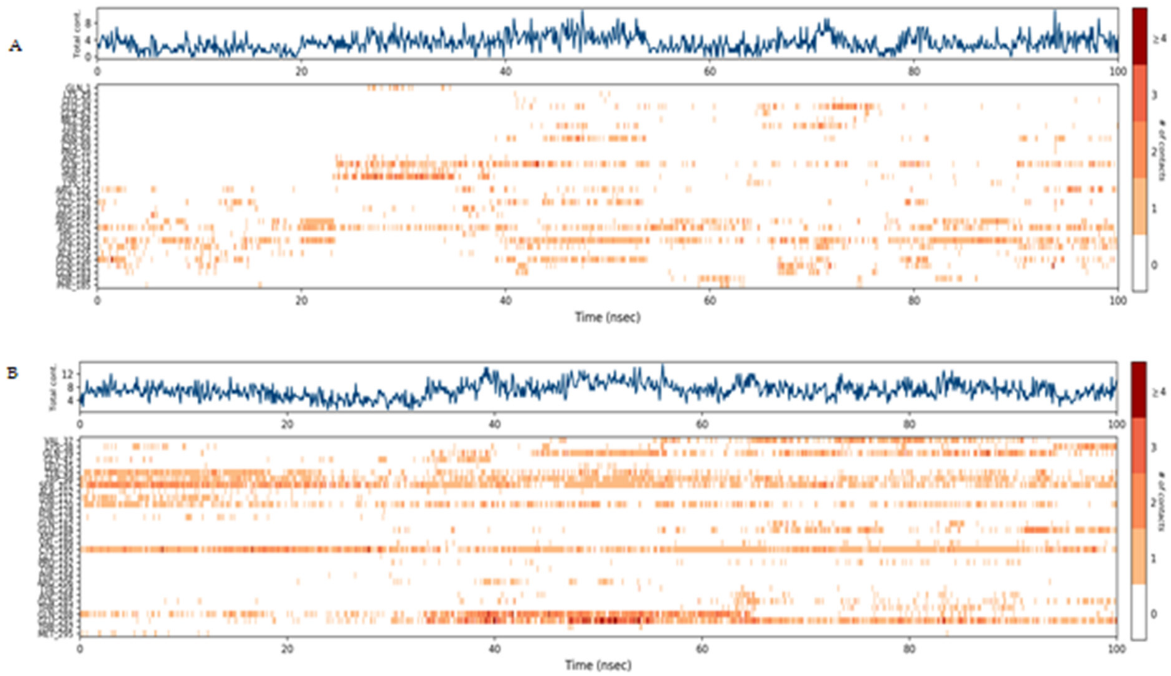

Supplementary Figure S10: A) The number of ligand contacts with ICAM1 protein and the amino acid sites; B) The number of ligand contacts with CCR2 protein and the amino acid sites.
